# Global, regional, and national burden of digestive diseases: findings from the global burden of disease study 2019

**DOI:** 10.3389/fpubh.2023.1202980

**Published:** 2023-08-24

**Authors:** Fang Wang, Dingtao Hu, Hongyu Sun, Ziye Yan, Yuhua Wang, Linlin Wang, Tingyu Zhang, Nana Meng, Chunxia Zhai, Qiqun Zong, Wanqin Hu, Guanghui Yu, Yanfeng Zou

**Affiliations:** ^1^Department of Oncology, The First Affiliated Hospital of Anhui Medical University, Hefei, Anhui, China; ^2^Clinical Cancer Institute, Center for Translational Medicine, Second Military Medical University, Shanghai, China; ^3^Department of Epidemiology and Biostatistics, School of Public Health, Anhui Medical University, Hefei, Anhui, China; ^4^Department of Quality Management Office, The Second Affiliated Hospital of Anhui Medical University, Hefei, Anhui, China

**Keywords:** digestive diseases, global burden of disease, incidence, deaths, DALYs

## Abstract

**Background:**

The global burden of digestive diseases has been rising in the last 30 years. The rates and trends of incidence, deaths, and disability-adjusted life-years (DALYs) for digestive diseases need to be investigated.

**Methods:**

We extracted the data on overall digestive diseases and by cause between 1990–2019 from the Global Burden of Diseases 2019 website, including the absolute number and the corresponding age-standardized rates of incidence (ASIR), deaths (ASDR), and DALYs (ASDALYs).

**Results:**

Globally, the incident cases, deaths, and DALYs of digestive diseases in 2019 increased by 74.44, 37.85, and 23.46%, respectively, compared with that in 1990, with an increasing ASIR of 0.09%, as well as decreasing ASDR and ASDALYs of 1.38 and 1.32% annually. The sociodemographic index (SDI) of overall digestive diseases showed a slight increase in ASIR from low to middle-low regions. The downtrend in ASDR and ASDALYs was found in all SDI regions. The burden of incidence was higher in females, while the burden of deaths and DALYs was higher in males for the overall digestive diseases and most causes. The estimated annual percentage changes were significantly associated with the baseline ASIR, ASDR, and ASDALYs for the overall digestive diseases, and the negative correlations between ASDR, ASDALYs, and human development index both in 1990 (*R* = −0.68, *R* = −0.69) and 2019 (*R* = −0.71, *R* = −0.73) were noticed.

**Conclusion:**

The findings indicate that digestive diseases remain a significant public health burden, with substantial variation across countries, sexes, and age groups. Therefore, implementing age, gender, and country-specific policies for early screening and targeted interventions could significantly reduce the global burden of digestive diseases.

## Introduction

The global burden of disease has shifted from perinatal, maternal, nutritional, and communicable diseases to non-communicable diseases, including digestive diseases, due to changes in global demographics and socio-economic factors such as improvements in health care, sanitation, and nutrition, an aging population, changes in dietary habits, and increasing urbanization (1). As an important part of non-communicable diseases, the global burden of digestive diseases has been on the rise in the last few decades (2). Based on available data, the incidence of digestive diseases in 2017 was over 4.6 billion worldwide, with the percentage of change of years lived with disability increased by 31.1% from 1990 to 2007 and 20.5% from 2007 to 2017 (3). Meanwhile, the counts of all-age disability-adjusted life-years (DALYs) also increased by 4.1% from 2006–2016 (3). Although the epidemiological characteristics of the global and regional level of several specific digestive diseases, such as cirrhosis (4), inflammatory bowel disease (5), and pancreatitis (6) have been reported, the studies that focus on the characterization of the burden and distribution of all digestive diseases in diverse countries and territories are still lacking. Therefore, a systematic analysis of the comparable epidemiological statistics of digestive diseases would help to evaluate the global burden of these diseases in diverse countries at different economic development levels and facilitate the formation of standard healthcare policy, which could decrease the burden of digestive diseases over time.

Based on the Global Burden of Disease (GBD) study (7), all digestive diseases were classified into ten categories, including appendicitis, pancreatitis, cirrhosis and other chronic liver diseases, inflammatory bowel disease, upper digestive system diseases, paralytic ileus and intestinal obstruction, inguinal femoral and abdominal hernia, vascular intestinal disorders, gallbladder and biliary diseases, and other digestive diseases. In the current research, we retrieved data on digestive diseases from the GBD 2019 study, ranging from 1990–2019, to analyze the burden of digestive diseases caused by nine major causes at the national, regional, and global levels. The association of these comparable statistics with the human development index (HDI) of different countries and the baseline age-standardized incidence rate (ASIR), death rate (ASDR), and DALYs rate (ASDALYs) was also explored. Since the GBD study did not provide any specific data on other digestive diseases in 2019 globally, we did not include this category in the study.

## Materials and methods

### Overview

Data about the burden of digestive diseases were downloaded from the GBD 2019 study, which provides estimations comprehensively on the burden of 369 diseases among 204 countries and territories based on all available information, such as the clinical and hospital data, survey data, surveillance data, and published literature (7). To assess and correct the potential bias of different data sources for the assessment of model performance and standardized statistical evaluation, the DisMod-MR V.2.1 meta-regression tool was used. The detailed information about the methodology for processing and estimating disease burden levels and trends in the GBD 2019 studies has been reported extensively (8).

### Data sources

Comparable statistics of digestive diseases from 1990 to 2019, including the numbers and age-standardized rates, which were sorted by cause, location, gender, and age, were obtained from the GBD 2019 study (7). 204 countries and regions were zoned as five SDI quintiles based on a combination of regional *per capita* income, average educational attainment, and fertility rankings. Meanwhile, these countries and territories were also separated into 21 regions on the basis of geographical contiguity. The list of five SDI quintiles, 21 regions, and all 204 countries and territories are presented in Table 1 and Supplementary Table S10. HDI Data of 193 countries and territories in 1990 and 2019, which was available from the Human Development Report 2019^1^ and can be matched with the data from the GBD 2019 study, was obtained for further study. Eleven countries and territories, including the United States Virgin Islands, Cook Islands, Tokelau, American Samoa, Taiwan (China), Northern Mariana Islands, Guam, Puerto Rico, Niue, Greenland, and Bermuda, were excluded.

**Table 1:** The incidence, death, and DALYs of digestive diseases in 1990 and 2019.

Characteristics	1990		2019		1990–2019	1990		2019		1990–2019	1990		2019		1990–2019
	Incidence cases No×10^6^ (95%UI)	ASR per 100,000 No×10^3^ (95% UI)	Incidence cases No×10^6^ (95%UI)	ASR per 100,000 No×10^3^ (95% UI)	EAPC No (95% CI)	Death cases No×10^4^ (95%UI)	ASR per 100,000 No (95% UI)	Death cases No×10^4^ (95%UI)	ASR per 100,000 No (95% UI)	EAPC No (95% CI)	DALYs No×10^6^ (95%UI)	ASR per 100,000 No×10^3^ (95% UI)	DALYs No×10^6^ (95%UI)	ASR per 100,000 No×10^3^ (95% UI)	EAPC No (95% CI)
Global	254.25 (231.57–277.83)	5.32 (4.87–5.80)	443.53 (405.58–484.42)	5.45 (4.99–5.94)	0.09 (0.05 to 0.12)	185.54 (175.45–193.02)	46.67 (44.06–48.76)	255.77 (238.99–271.63)	32.07 (29.87–34.05)	−1.38 (−1.44 to −1.31)	72.08 (66.88–77.58)	1.57 (1.47–1.68)	88.99 (81.41–97.58)	1.10 (1.00–1.20)	−1.32 (−1.36 to −1.27)
Sex															
Female	134.15 (122.24–146.45)	5.56 (5.09–6.06)	236.26 (216.39–258.15)	5.73 (5.24–6.25)	0.12 (0.09 to 0.15)	73.82 (68.15–79.00)	34.92 (32.24–37.24)	103.35 (94.05–111.43)	24.01 (21.88–25.90)	−1.38 (−1.44 to −1.31)	28.11 (25.31–31.16)	1.19 (1.07–1.31)	34.49 (30.95–38.79)	0.83 (0.74–0.94)	−1.31 (−1.35 to −1.27)
Male	120.10 (109.18–131.50)	5.08 (4.65–5.56)	207.26 (188.91–227.15)	5.17 (4.73–5.65)	0.05 (0.02 to 0.09)	111.72 (104.19–117.46)	59.80 (55.66–63.05)	152.42 (141.71–163.67)	40.80 (38.02–43.82)	−1.40 (−1.45 to −1.34)	43.97 (40.72–46.85)	1.97 (1.84–2.09)	54.50 (49.90–59.47)	1.37 (1.25–1.50)	−1.33 (−1.38 to −1.28)
SDI															
Low SDI	21.22 (19.04–23.58)	5.78 (5.21–6.40)	46.76 (41.94–51.84)	5.63 (5.10–6.21)	0.08 (0.03 to 0.14)	21.61 (18.99–24.06)	82.86 (73.27–91.85)	32.62 (28.76–36.75)	58.46 (52.22–65.25)	−1.30 (−1.40 to −1.19)	9.22 (7.86–10.50)	2.58 (2.29–2.85)	13.69 (11.92–15.66)	1.81 (1.60–2.04)	−1.30 (−1.39 to −1.21)
Low-middle SDI	52.57 (47.48–57.94)	5.98 (5.44–6.55)	102.49 (92.94–112.41)	6.14 (5.61–6.71)	0.12 (0.10 to 0.14)	45.36 (41.78–48.27)	70.53 (64.24–76.42)	63.12 (57.99–69.01)	46.21 (42.50–50.41)	−1.56 (−1.64 to −1.48)	19.30 (17.72–20.93)	2.32 (2.13–2.48)	24.13 (21.78–26.58)	1.54 (1.39–1.69)	−1.49 (−1.55 to −1.44)
Middle SDI	69.46 (63.17–76.02)	4.74 (4.35–5.16)	127.75 (116.71–139.78)	4.94 (4.51–5.37)	0.30 (0.24 to 0.36)	52.40 (49.05–55.26)	51.75 (48.07–55.14)	72.66 (66.69–79.45)	31.42 (28.66–34.35)	−1.77 (−1.81 to −1.73)	21.21 (19.69–22.74)	1.62 (1.50–1.73)	25.15 (22.83–27.8)	0.99 (0.90–1.09)	−1.75 (−1.78 to −1.72)
High-middle SDI	60.66 (55.48–66.24)	5.27 (4.84–5.74)	90.82 (83.58–99.50)	5.20 (4.77–5.66)	−0.07 (−0.11 to −0.03)	36.16 (34.60–37.59)	35.47 (33.79–36.93)	46.58 (43.28–49.48)	23.92 (22.20–25.42)	−1.45 (−1.61 to −1.28)	13.22 (12.24–14.41)	1.19 (1.11–1.30)	15.45 (14.17–17.13)	0.83 (0.76–0.93)	−1.36 (−1.52 to −1.20)
High SDI	50.20 (46.10–54.80)	5.41 (4.96–5.90)	68.28 (62.79–74.60)	5.24 (4.81–5.74)	−0.11 (−0.15 to −0.06)	29.91 (28.50–30.66)	29.58 (28.16–30.33)	40.63 (36.67–42.90)	21.02 (19.33–22.03)	−1.26 (−1.30 to −1.22)	9.09 (8.43–9.88)	0.94 (0.87–1.03)	10.52 (9.60–11.53)	0.69 (0.63–0.76)	−1.17 (−1.22 to −1.13)
Region															
Andean Latin America	2.73 (2.50–2.97)	8.42 (7.75–9.11)	5.10 (4.67–5.54)	8.11 (7.45–8.80)	0.07 (0.07 to 0.08)	1.77 (1.58–1.96)	69.43 (62.58–76.57)	2.44 (1.97–2.99)	43.84 (35.54–53.62)	−1.59 (−1.70 to −1.47)	0.92 (0.81–1.03)	2.67 (2.39–2.96)	0.80 (0.66–0.97)	1.35 (1.12–1.64)	−2.45 (−2.66 to −2.23)
Australasia	1.12 (1.02–1.24)	5.07 (4.61–5.59)	1.82 (1.67–2.00)	5.14 (4.68–5.65)	−0.21 (−0.27 to −0.16)	0.47 (0.44–0.49)	21.15 (19.72–22.02)	0.77 (0.68–0.83)	14.75 (13.18–15.82)	−1.12 (−1.28 to −0.95)	0.14 (0.12–0.15)	0.61 (0.55–0.68)	0.20 (0.17–0.22)	0.47 (0.41–0.54)	−0.79 (−0.93 to −0.66)
Caribbean	2.35 (2.12–2.58)	7.36 (6.70–8.05)	3.75 (3.42–4.09)	7.53 (6.86–8.22)	0.05 (−0.02 to 0.11)	1.19 (1.05–1.31)	44.10 (39.40–47.51)	1.72 (1.42–2.01)	33.59 (27.75–39.45)	−1.08 (−1.29 to −0.86)	0.50 (0.41–0.59)	1.62 (1.37–1.87)	0.62 (0.50–0.74)	1.25 (1.00–1.49)	−0.98 (−1.19 to −0.76)
Central Asia	3.16 (2.84–3.49)	5.35 (4.85–5.92)	5.01 (4.51–5.57)	5.45 (4.95–6.02)	0.07 (0.06 to 0.09)	2.06 (2.00–2.11)	41.22 (39.93–42.31)	4.18 (3.77–4.64)	54.18 (48.90–59.76)	0.79 (0.43 to 1.16)	0.86 (0.81–0.92)	1.50 (1.42–1.60)	1.62 (1.46–1.80)	1.83 (1.66–2.02)	0.50 (0.14 to 0.85)
Central Europe	8.64 (7.93–9.48)	6.38 (5.86–7.00)	9.61 (8.83–10.48)	6.41 (5.89–7.02)	0.02 (−0.01 to 0.05)	5.76 (5.60–5.87)	41.01 (39.73–41.87)	6.16 (5.46–6.91)	30.99 (27.34–34.82)	−1.22 (−1.35 to −1.08)	2.03 (1.90–2.19)	1.45 (1.36–1.57)	1.90 (1.67–2.13)	1.10 (0.97–1.24)	−1.25 (−1.38 to −1.12)
Central Latin America	10.73 (9.73–11.73)	8.16 (7.49–8.84)	21.07 (19.29–22.90)	8.27 (7.59–8.97)	0.09 (0.07 to 0.11)	5.78 (5.61–5.90)	63.78 (61.16–65.32)	11.28 (9.81–12.81)	47.97 (41.86–54.41)	−1.17 (−1.28 to −1.05)	2.50 (2.35–2.70)	2.15 (2.03–2.30)	3.87 (3.41–4.41)	1.57 (1.39–1.79)	−1.22 (−1.36 to −1.09)
Central Sub-Saharan Africa	2.07 (1.85–2.31)	5.48 (4.95–6.08)	5.43 (4.86–6.05)	5.78 (5.24–6.40)	0.18 (0.17 to 0.19)	2.22 (1.88–2.59)	88.80 (76.46–101.71)	3.83 (2.94–4.83)	65.80 (50.45–81.32)	−1.04 (−1.10 to −0.98)	0.96 (0.81–1.13)	2.69 (2.29–3.10)	1.63 (1.26–2.08)	2.01 (1.58–2.51)	−0.99 (−1.05 to −0.94)
East Asia	42.30 (38.81–45.92)	3.76 (3.47–4.07)	70.14 (64.44–76.41)	3.77 (3.48–4.09)	−0.01 (−0.09 to 0.07)	33.76 (29.79–37.48)	40.81 (36.5–45.07)	29.81 (25.89–34.26)	15.99 (14.03–18.24)	−3.31 (−3.45 to −3.17)	13.64 (12.19–15.21)	1.34 (1.20–1.49)	10.62 (9.14–12.12)	0.55 (0.48–0.63)	−3.19 (−3.27 to −3.11)
Eastern Europe	16.11 (14.72–17.71)	6.37 (5.82–6.98)	17.02 (15.53–18.66)	6.47 (5.92–7.08)	−0.03 (−0.11 to 0.04)	6.88 (6.73–7.15)	25.85 (25.18–26.87)	13.22 (11.90–14.65)	42.68 (38.45–47.30)	1.77 (1.24 to 2.31)	2.61 (2.42–2.88)	1.00 (0.92–1.10)	4.66 (4.19–5.20)	1.65 (1.48–1.84)	1.76 (1.19 to 2.33)
Eastern Sub-Saharan Africa	6.61 (5.89–7.39)	5.34 (4.78–5.94)	15.88 (14.13–17.73)	5.51 (4.96–6.11)	0.10 (0.09 to 0.12)	7.75 (6.39–9.01)	93.94 (80.53–106.78)	12.60 (10.96–14.66)	73.14 (64.79–83.44)	−0.94 (−1.01 to −0.88)	3.15 (2.50–3.74)	2.75 (2.30–3.16)	4.94 (4.21–5.83)	2.07 (1.80–2.40)	−1.06 (−1.11 to −1.01)
High-income Asia Pacific	9.57 (8.81–10.46)	5.00 (4.60–5.44)	13.51 (12.46–14.72)	5.21 (4.81–5.67)	0.27 (0.18 to 0.36)	6.33 (6.06–6.48)	33.60 (31.86–34.52)	7.88 (6.59–8.61)	15.77 (13.91–16.92)	−2.74 (−2.91 to −2.57)	2.07 (1.93–2.23)	1.05 (0.98–1.13)	1.84 (1.64–2.06)	0.57 (0.49–0.65)	−2.28 (−2.42 to −2.15)
High-income North America	20.15 (18.40–22.09)	6.40 (5.85–7.02)	25.27 (23.11–27.73)	5.54 (5.05–6.10)	−0.53 (−0.64 to −0.41)	8.56 (8.09–8.81)	24.67 (23.41–25.33)	14.22 (13.20–14.81)	23.13 (21.76–23.97)	−0.17 (−0.21 to −0.13)	2.80 (2.57–3.10)	0.87 (0.79–0.97)	4.00 (3.72–4.36)	0.77 (0.71–0.85)	−0.25 (−0.32 to −0.17)
North Africa and Middle East	15.09 (13.41–16.8)	5.69 (5.11–6.30)	34.78 (31.19–38.5)	5.91 (5.36–6.51)	0.15 (0.14 to 0.15)	9.28 (8.55–9.87)	58.88 (53.6–64.47)	14.57 (11.26–17.59)	37.48 (29.66–44.82)	−1.50 (−1.57 to −1.42)	3.29 (2.87–3.69)	1.50 (1.38–1.63)	4.84 (3.85–5.80)	0.99 (0.80–1.18)	−1.37 (−1.42 to −1.32)
Oceania	0.15 (0.13–0.17)	2.99 (2.69–3.33)	0.34 (0.30–0.37)	3.02 (2.73–3.37)	0.03 (0.01 to 0.04)	0.12 (0.10–0.14)	37.52 (31.92–44.41)	0.22 (0.18–0.28)	29.04 (24.03–35.26)	−0.87 (−0.91 to −0.83)	0.05 (0.05–0.06)	1.22 (1.05–1.42)	0.10 (0.08–0.12)	0.97 (0.81–1.17)	−0.76 (−0.79 to −0.73)
South Asia	55.23 (49.52–61.19)	6.45 (5.85–7.09)	115.67 (104.59–127.26)	6.69 (6.09–7.33)	0.14 (0.11 to 0.17)	41.17 (37.53–44.46)	68.45 (60.96–75.75)	58.06 (51.76–66.06)	41.33 (36.75–47.02)	−1.94 (−2.11 to −1.78)	17.78 (16.03–19.36)	2.23 (2.02–2.40)	23.23 (20.53–26.28)	1.42 (1.26–1.61)	−1.68 (−1.79 to −1.57)
Southeast Asia	12.42 (11.20–13.7)	3.24 (2.95–3.58)	23.33 (21.21–25.80)	3.36 (3.06–3.70)	0.12 (0.10 to 0.13)	18.61 (16.86–19.99)	68.89 (62.15–75.88)	27.55 (24.70–30.34)	47.79 (43.09–52.39)	−1.37 (−1.41 to −1.33)	7.40 (6.44–8.21)	2.13 (1.93–2.29)	9.06 (8.11–10.09)	1.38 (1.24–1.52)	−1.62 (−1.66 to −1.58)
Southern Latin America	2.86 (2.57–3.19)	5.97 (5.36–6.65)	4.52 (4.08–5.01)	6.12 (5.52–6.81)	−0.04 (−0.09 to 0.01)	1.94 (1.88–1.99)	43.59 (41.88–44.76)	2.78 (2.58–2.92)	33.43 (31.23–35.10)	−0.82 (−0.94 to −0.69)	0.61 (0.57–0.66)	1.30 (1.23–1.40)	0.76 (0.70–0.83)	0.97 (0.89–1.06)	−0.95 (−1.08 to −0.82)
Southern Sub-Saharan Africa	2.34 (2.10–2.58)	5.69 (5.13–6.31)	4.28 (3.83–4.74)	5.81 (5.24–6.41)	0.06 (0.04 to 0.08)	1.34 (1.19–1.60)	45.19 (39.47–54.59)	2.05 (1.87–2.24)	36.92 (33.77–40.07)	−0.77 (−1.11 to −0.44)	0.57 (0.51–0.64)	1.51 (1.34–1.75)	0.79 (0.70–0.88)	1.17 (1.05–1.30)	−0.95 (−1.29 to −0.62)
Tropical Latin America	10.59 (9.58–11.55)	8.14 (7.47–8.81)	19.98 (18.26–21.64)	8.15 (7.46–8.79)	−0.03 (−0.09 to 0.04)	4.47 (4.33–4.59)	47.00 (44.87–48.42)	8.02 (7.50–8.38)	33.60 (31.28–35.17)	−1.08 (−1.14 to −1.02)	1.96 (1.82–2.14)	1.67 (1.56–1.81)	2.88 (2.63–3.20)	1.18 (1.08–1.31)	−1.20 (−1.23 to −1.17)
Western Europe	22.93 (21.08–25.10)	4.98 (4.56–5.45)	29.45 (27.07–32.15)	5.13 (4.69–5.61)	0.10 (0.07 to 0.13)	17.69 (16.91–18.15)	31.75 (30.26–32.56)	20.51 (18.49–21.68)	21.12 (19.38–22.14)	−1.55 (−1.61 to −1.50)	4.78 (4.48–5.15)	0.94 (0.88–1.02)	4.72 (4.32–5.18)	0.65 (0.59–0.73)	−1.48 (−1.55 to −1.42)
Western Sub-Saharan Africa	7.09 (6.33–7.90)	5.27 (4.72–5.87)	17.58 (15.67–19.60)	5.40 (4.85–6.01)	0.07 (0.06 to 0.08)	8.37 (6.77–10.36)	86.15 (68.77–108.73)	13.89 (10.90–17.55)	66.12 (53.56–81.88)	−0.82 (−0.92 to −0.73)	3.45 (2.90–4.12)	2.60 (2.11–3.21)	5.92 (4.64–7.47)	1.98 (1.58–2.48)	−0.87 (−0.94 to −0.81)

ASR, age-standardized incidence rate; EAPC, estimated annual percentage change; UI, uncertainty interval.

### Statistical analysis

Consistent with the previous studies, the burden levels and trends of digestive diseases were estimated by the ASRs (including ASIR, ASDR, and ASDALYs) and estimated annual percentage change (EAPC) (8). To compare the populations across various locations over time, the potential confounding of age structure needed to be adjusted, and the data was thus standardized. Considered *a_i_* as the age-specific rate for the *i*th age class, *wi* denotes the numbers (or weights) for the same age class *i* of the selected reference standard population, the truncated ASR could be calculated as the following formula:


$$\text{ASR}=\frac{\sum_{i=1}^{A}aiwi}{\Sigma_{i=1}^{A}wi}\times 100,000$$

In addition, the trends of ASRs over a specified time interval were summarized and evaluated quantitatively by EAPC, which was counted by a regression model fitted to the natural logarithm of ASRs, and the details for calculating EAPC have been described in detail elsewhere (8).

An optimistic EAPC estimation indicates an increasing trend of ASR while being minus means a decreasing trend. Moreover, we also performed a correlation analysis between the EAPC and ASRs in 1990 and between ASRs and HDI in 1990 and 2019 for overall digestive diseases and causes. According to the EAPC and values of 95%CI, we also classified the 204 countries and territories by hierarchical cluster analysis. Depicting with maps, we presented the global incidence, death, and DALYs of overall, appendicitis, pancreatitis, cirrhosis and other chronic liver diseases, inflammatory bowel disease, upper digestive system diseases, paralytic ileus and intestinal obstruction, inguinal femoral and abdominal hernia, vascular intestinal disorders, and gallbladder and biliary diseases by locations for all ages, both sexes combined, including the numbers in 2019, ASRs in 2019, and EAPCs of ASRs in 1990–2019. The R-index and value of *p* of the relationship between the variables were probed using Pearson correlation analysis with the R version 3.5.3 of the utilization. Statistical meaningfulness is deemed when the *value of p* is less than 0.05.

## Results

### Number of incidences, deaths, and DALYs of digestive diseases

**Figure 1:**
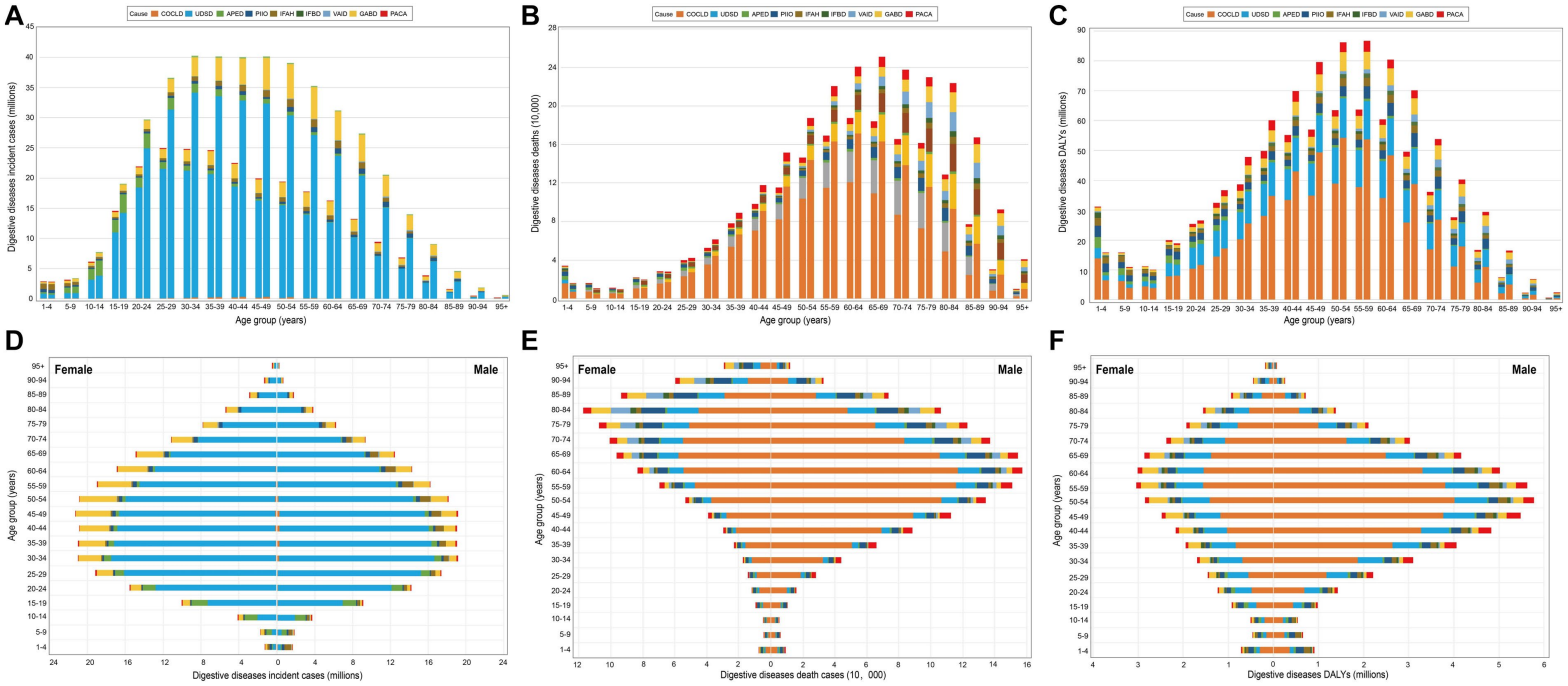
Global cases of incidence, deaths, and DALYs of digestive diseases by nine causes and 23 GBD age groups. **(A–C)** Global cases of incidence, deaths, and DALYs of digestive diseases by age for both sexes combined in 1990 and 2019. For each group, the left column shows case data in 1990 and the right column shows data in 2019. **(D–F)** Sex difference in global cases of incidence, deaths, and DALYs of digestive diseases by age in 2019. DALYs, disability-adjusted life-years; GBD, Global Burden of Disease; COCLD, Cirrhosis and other chronic liver diseases; UDSD, Upper digestive system diseases; APED, Appendicitis; PIIO, Paralytic ileus and intestinal obstruction; IFAH, Inguinal, femoral, and abdominal hernia; IFBD, Inflammatory bowel disease; VAID, Vascular intestinal disorders; GABD, Gallbladder and biliary diseases; PACA, Pancreatitis.

Table 1 and Supplementary Tables S1–S9 showed the absolute number, ASRs, and the EAPC of the incidences, deaths, and DALYs of the overall digestive diseases and by causes in 1990 and 2019. Globally, the incident cases, deaths, and DALYs of digestive diseases in 2019 were 443.53 million, 2.56 million, and 88.99 million, increased by 74.44, 37.85, and 23.46%, respectively, compared with that in 1990 (Table 1). Of notice, we found India had the prominent incidences, deaths, and DALYs in 2019, with the corresponding present change in absolute numbers as 109.11, 47.48, and 34.42%, respectively. The country-specific contribution of the incidence, deaths, and DALYs presented in Supplementary Table S10. The incidence, deaths, and DALYs of digestive diseases increased particularly in the 25–59-year class, 45–79-year class, and 39–69-year class, respectively, with the peak shifting from 25–29 years in 1990 to 30–34 years in 2019 for incidence, from 60–64 years in 1990 to 65–69 years in 2019 for deaths, and peak for DALYs was occurred both in 55–59 years in 1990 and 2019 (Figures 1A–C). Of interest, we observed relatively higher deaths and DALYs in 1–4 years than that in 5–19 years, both in 1990 and 2019.

**Figure 2:**
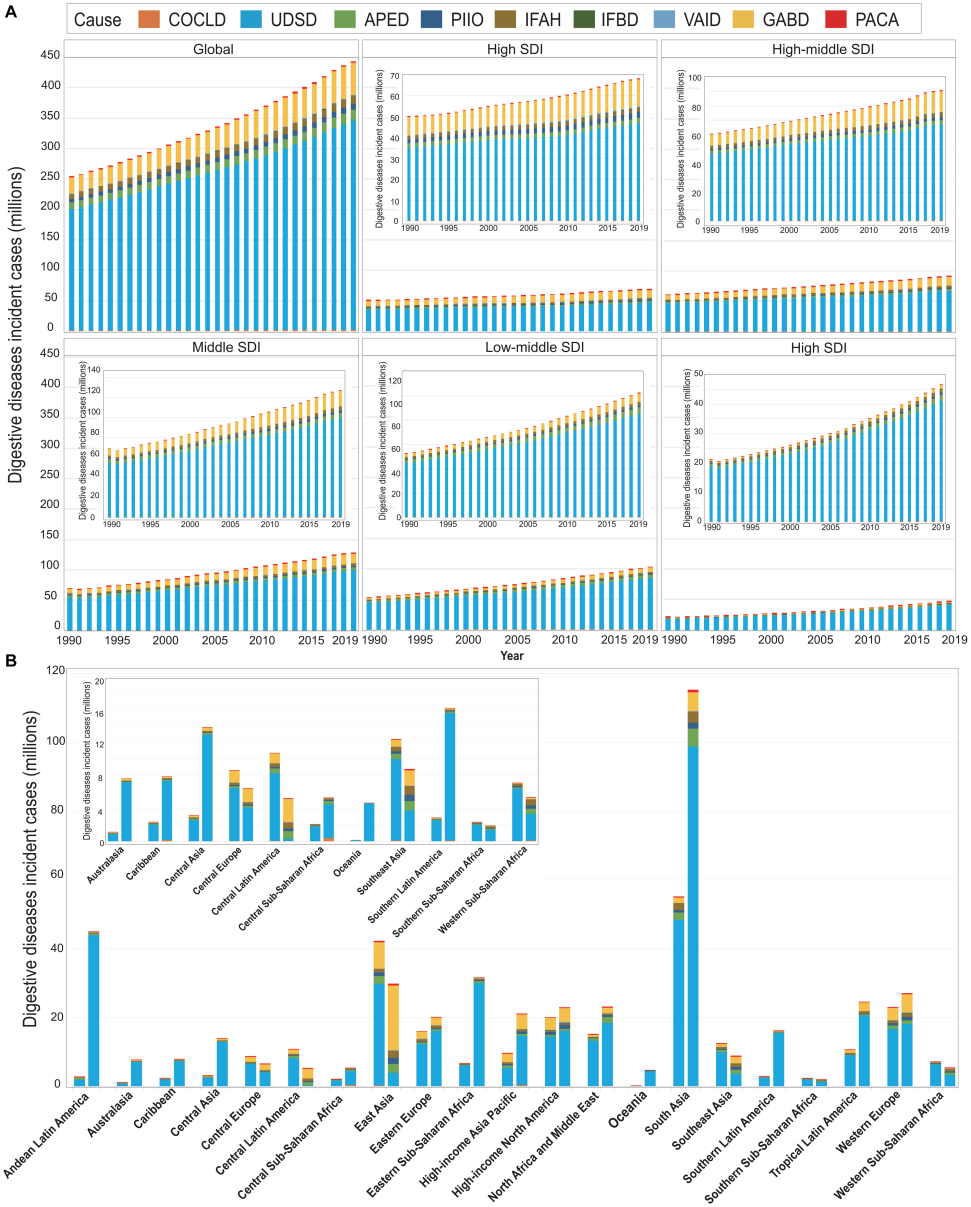
Global incident cases of digestive diseases by nine causes and regions for both sexes combined. **(A)** The incident cases of digestive diseases by nine causes and five SDI regions, from 1990 to 2019. **(B)** Incident cases of digestive diseases by nine causes and by 21 GBD regions in 1990 and 2019. For each group, the left column shows case data in 1990, and the right column shows data in 2019. Certain regions are magnified to the top-right of the panel. COCLD, cirrhosis and other chronic liver diseases; UDSD, Upper digestive system diseases; APED, Appendicitis; PIIO, Paralytic ileus and intestinal obstruction; IFAH, Inguinal, femoral, and abdominal hernia; IFBD, Inflammatory bowel disease; VAID, Vascular intestinal disorders; GABD, Gallbladder and biliary diseases; PACA, Pancreatitis.

Compared with female groups (236.26 million for incidence, 1.03 million for deaths, and 34.49 million for DALYs), males had relatively lower incident cases (207.26 million), and higher deaths (1.52 million) and DALYs (54.50 million) in 2019 (Figures 1D–F; Table 1). In general, we observed an increasing trend in the incidences, deaths, and DALYs of digestive diseases across all regions from 1990 to 2019, especially for locations with lower SDI (Table 1; Figure 2A; Supplementary Figures S1A, S2A). Among 21 GBD regions, the highest increase in the incidences, deaths, and DALYs was in Central Sub-Saharan Africa (CSS, 162.59%), Central Asia (CA, 102.92%), and CA (88.37%), respectively (Table 1; Figure 2B; Supplementary Figures S1B, S2B). While a prominent number of incidences, deaths, and DALYs were observed in South Asia (SA) both in 1990 and 2019.

### The ASIR, ASDR, and ASDALYs of digestive diseases

**Figure 3:**
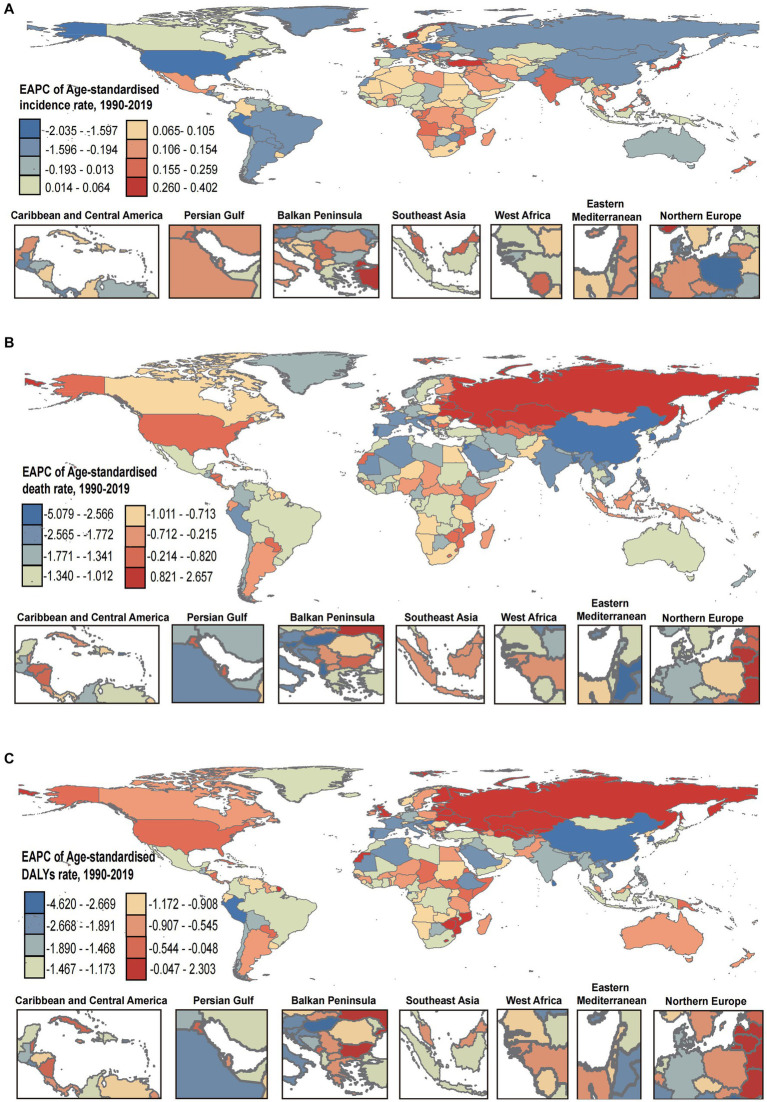
The EAPC in ASIR **(A)**, ASDR **(B)**, and ASDALYs **(C)** of digestive diseases from 1990 to 2019. DALYs, disability-adjusted life-years; ASIR, age-standardized incidence rate; ASDR, age-standardized deaths rate; ASDALYs, age-standardized DALYs rate. EAPC, estimated annual percentage change.

Globally, the ASIR, ASDR, and ASDALYs of digestive diseases were 5454.63, 32.07, and 1096.99 per 100,000 in 2019, with the variation of 2.82-fold, 17.2-fold, and 8.28-fold across countries. The highest rates in 2019 were in Mexico (8468.46 per 100,000) for ASIR, Egypt (138.60 per 100,000) for ASDR, and Cambodia (2937.01 per 100,000) for ASDALYs. While the lowest rates were in Papua New Guinea (3001.97 per 100,000) for ASIR, Singapore (8.06 per 100,000) for ASDR, and Iceland (354.88 per 100,000) for ASDALYs (Table 1; Supplementary Table S10). Although the ASIR increased by an average of 0.09% (95% CI, 0.05–0.12%) between 1990–2019, the ASDR and ASDALYs decreased instead, with the average of 1.38% (95% CI, −1.44% to −1.31%) and 1.32% (95% CI, −1.36% to −1.27%), respectively (Table 1). The countries-specific distribution of EAPC for ASIR, ASDR, and ASDALYs were presented in Figures 3A–C. Notable, The EAPC (95% CI) of ASIR, ASDR, and ASDALYs were 0.12 (0.09 to 0.15), −1.38 (−1.44 to −1.31), and − 1.31 (−1.35 to −1.27) for females, and 0.05(0.02 to 0.09), −1.40(−1.45 to −1.34), and − 1.33(−1.38 to −1.28) for males (Table 1).

**Figure 4:**
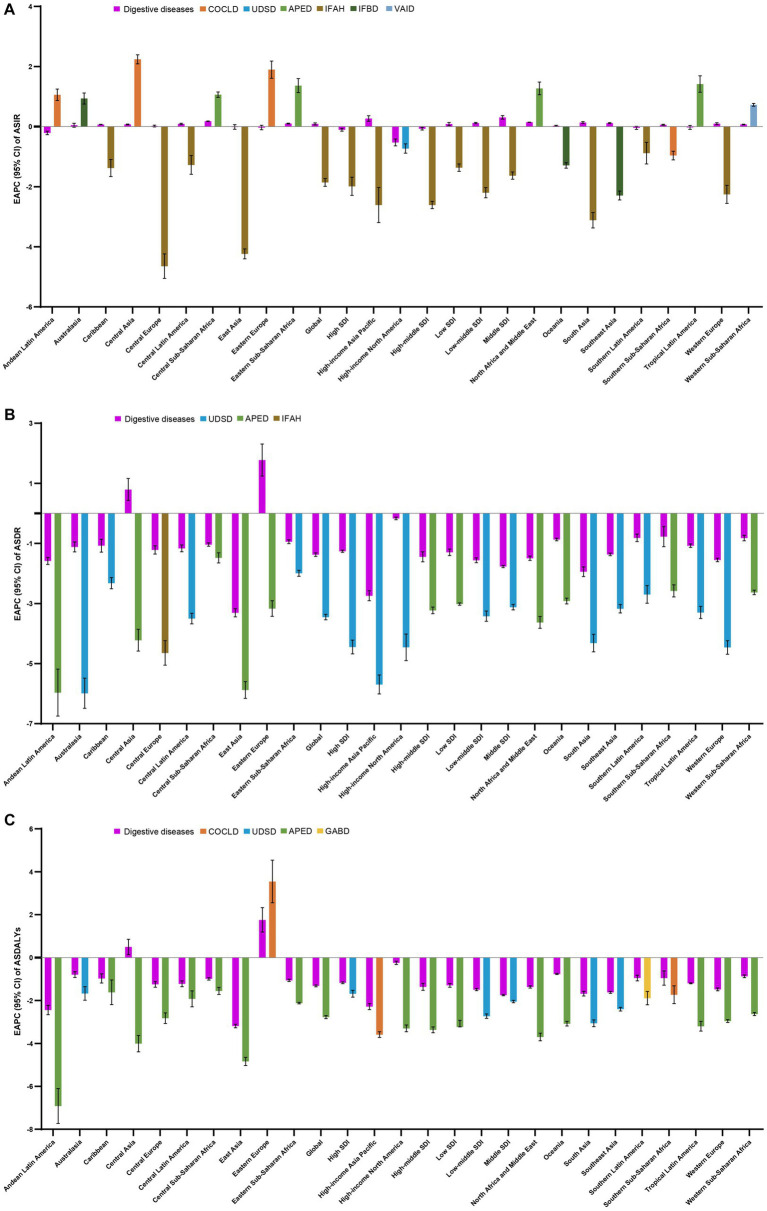
The EAPC in ASIR **(A)**, ASDR **(B)**, and ASDALYs **(C)** of digestive diseases from 1990 to 2019, by causes and by region, for both sexes combined. Those EAPCs in each of the regions are presented as the overall and the absolute maximum caused by a specific cause. DALYs, disability-adjusted life-years; ASIR, age-standardized incidence rate; ASDR, age-standardized deaths rate; ASDALYs, age-standardized DALYs rate. EAPC, estimated annual percentage change; COCLD, Cirrhosis and other chronic liver diseases; UDSD, Upper digestive system diseases; APED, Appendicitis; IFAH, Inguinal, femoral, and abdominal hernia; IFBD, Inflammatory bowel disease; VAID, Vascular intestinal disorders; GABD, Gallbladder and biliary diseases.

As for the five SDI regions, the ASIR of digestive diseases was on the rise in the regions with low to moderate SDI, while it was on the decline in the regions with high-moderate and high SDI (Figure 4A; Table 1). Meanwhile, we observed highly decreasing trends of ASDR and ASDALYs in all SDI regions (Figures 4B,C; Table 1). Moreover, stable and slight changes in ASIR were observed among 21 GBD regions over time, with the highest decreasing trend occurring in High-income North America (−0.53, −0.64 to −0.41) and the highest increasing trend appeared in High-income Asia Pacific (0.27, 0.18 to 0.36) (Figure 4A; Table 1). Of interest, the overall trends of ASDR and ASIR in various GBD regions were also decreasing, except in Central Asia (ASDR: 0.79, 0.43 to 1.16; ASDALYs: 0.50, 0.14 to 0.85) and Eastern Europe (ASDR: 1.77, 1.24 to 2.31; ASDALYs: 1.76, 1.19 to 2.33), which presented with increasing trends (Figures 4B,C; Table 1). In addition, the 204 countries and territories were also zoned as five classes by the hierarchical cluster analysis according to the EAPC and its 95%CI, and the detailed information was shown in Supplementary Figure S3.

### Incidence, deaths, and DALYs of digestive diseases by cause

**Figure 5:**
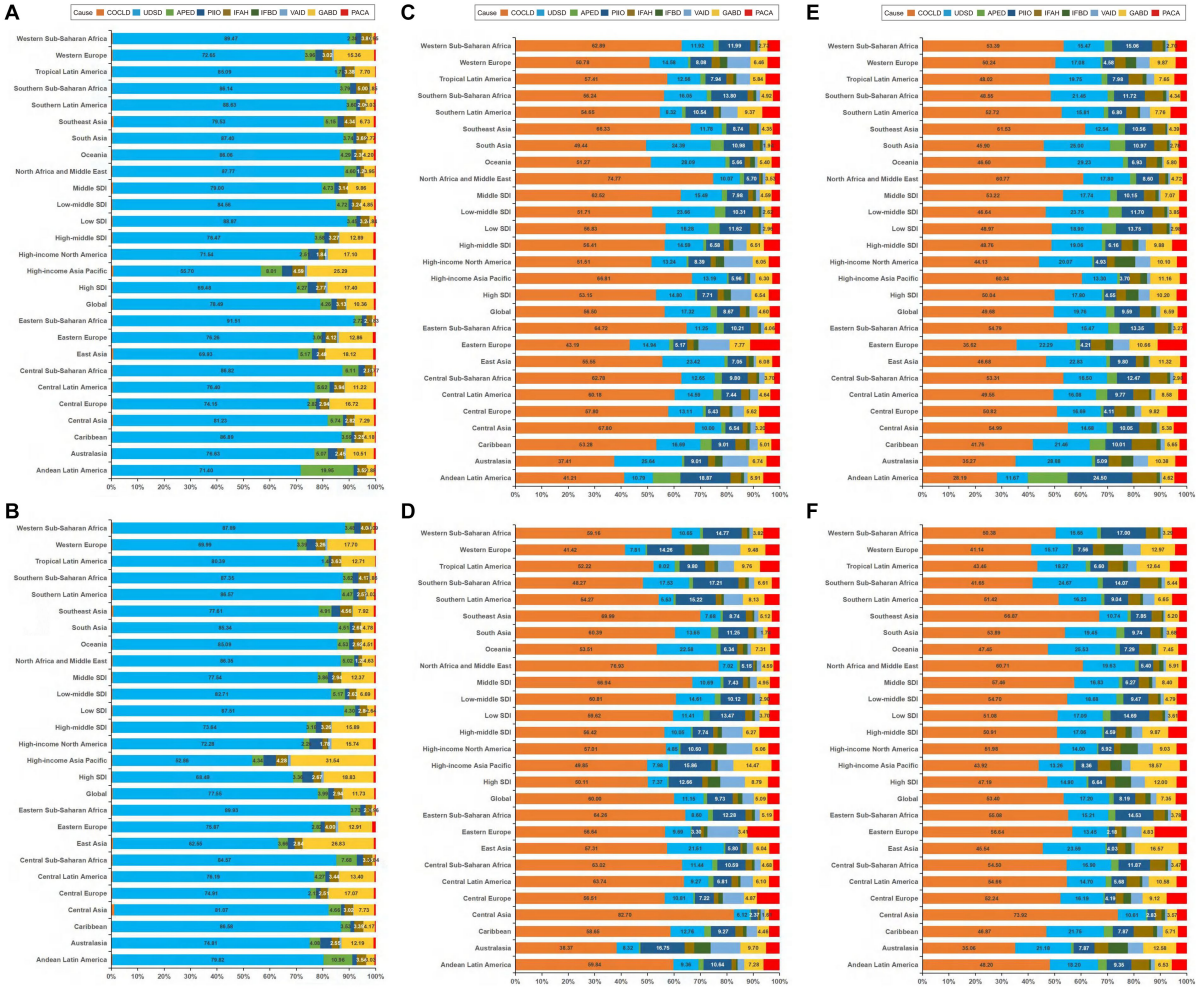
Contribution of specific causes to the cases of incidence, deaths, and DALYs of digestive diseases by regions for both sexes combined in 1990 and 2019. **(A)** The incidence in1990. **(B)** The incidence in 2019. **(C)** The deaths in1990. **(D)** The deaths in 2019. **(E)** The DALYs in1990. **(F)** The DALYs in 2019 DALYs. disability-adjusted life-years; COCLD, Cirrhosis and other chronic liver diseases; UDSD, Upper digestive system diseases; APED, Appendicitis; PIIO, Paralytic ileus and intestinal obstruction; IFAH, Inguinal, femoral, and abdominal hernia; IFBD, Inflammatory bowel disease; VAID, Vascular intestinal disorders; GABD, Gallbladder and biliary diseases; PACA, Pancreatitis.

Globally, the incident ranking of the nine causes of digestive diseases in 2019 was the same as in 1990, upper digestive system diseases was the leading incident cause of digestive diseases, followed by gallbladder and biliary diseases, appendicitis, inguinal femoral and abdominal hernia, paralytic ileus and intestinal obstruction, pancreatitis, cirrhosis and other chronic liver diseases, vascular intestinal disorders, and inflammatory bowel disease, accounting for 77.55, 11.73, 3.99, 2.94, 2.28, 0.63, 0.46, 0.33, and 0.09%, respectively (Figures 5A,B). As for deaths and DALYs, the top five leading causes were cirrhosis and other chronic liver diseases (accounts for 60.00 and 53.40%, respectively), upper digestive system diseases (accounts for 11.15 and 17.20%, respectively), paralytic ileus and intestinal obstruction (accounts for 9.73, and 8.19%, respectively), gallbladder and biliary diseases (accounts for 5.09, and 7.35%, respectively), and pancreatitis (accounts for 4.69, and 4.21%, respectively) in 2019, and appendicitis ranks the last cause of deaths (1.36%) and DALYs (1.74%) in 2019 (Figures 5C–F). The incidences for nine causes increased between 1990–2019, ranging from 37.80% for inflammatory bowel disease to 97.35% for gallbladder and biliary diseases. While both increasing and decreasing trends for deaths and DALYs were observed for different causes, with the prominent increasing and decreasing trend occurring in vascular intestinal disorders (95.25%), gallbladder and biliary diseases (−84.85%) for deaths, and pancreatitis (49.34%) and appendicitis (−80.99%) for DALYs. The trends of ASRs were also different across regions for nine causes. The details are shown in Supplementary Tables S1–S9. In the global and regional levers, the highest EAPC in ASIR among nine causes of digestive diseases varied among locations, cirrhosis and other chronic liver diseases and appendicitis in four GBD regions; upper digestive system diseases and vascular intestinal disorders in one GBD region; inguinal femoral and abdominal hernia in five SDI regions, and eight GBD regions; and inflammatory bowel disease in three GBD regions (Figure 4A; Supplementary Figure S13A). The highest EAPC in ASDR was noticed for upper digestive system diseases at the global level, except for low and high-middle SDI regions and 11 GBD regions; for appendicitis in high-middle and low SDI regions and nine GBD regions; and for inguinal femoral and abdominal hernia in one GBD region (Figure 4B; Supplementary Figure S13B). As for the distribution of the prominent EAPC in ASDALYs, we found highest EAPC of cirrhosis and other chronic liver diseases occurred in three GBD regions; upper digestive system diseases in high, middle, and low-middle SDI regions, and three GBD regions; appendicitis in high-middle and low SDI regions, and 14 GBD regions; and gallbladder and biliary diseases in one GBD region (Figure 4C; Supplementary Figure S13C). The detailed description of the absolute number of incidences, deaths, DALYs, ASRs, and EAPC for nine causes of digestive diseases and the distribution of gender, age, and locations were shown in the online Supplementary files.

### Factors correlated with the incidence, deaths, and DALYs of digestive diseases

Significant negative correlations between EAPC and baseline ASIR (*R* = -0.15, *p* = 0.033), as well as between EAPC and ASDR (*R* = −0.16, *p* = 0.027) in 1990, were observed (Supplementary Figures S14A,D). EAPC was positively correspond with the baseline ASDALYs (*R* = 0.23, *p* < 0.001, Supplementary Figure S14G). The ASDR (*R* = −0.68, *p* < 0.001 in 1990; *R* = −0.71, *p* < 0.001 in 2019, Supplementary Figures S14E,F) and ASDALYs (*R* = −0.69, *p* < 0.001 in 1990; *R* = −0.73, *p* < 0.001 in 2019, Supplementary Figures S14H,I) were strongly negatively correlated with the HDI in the corresponding year, while no significant relationship was observed between ASIR and HDI both in 1990 and 2019 (*p* > 0.05 for all, Supplementary Figures S14B,C). The associations among EAPC, ASRs, and HDI of each cause are shown in Supplementary Figures S15–S23.

## Discussion

We roundly analyzed the national, regional, and global burdens of the overall digestive diseases and by causes from 1990 to 2019, based on the results of the GBD 2019 study. In the last 30 years, the cases of the incidence, deaths, and DALYs in 2019 increased by 74.44, 37.85, and 23.46%, respectively; however, the ASDR and ASDALYs of digestives decreased by an annual average of 1.38 and 1.32%, while the ASIR also slightly increased during this period. Upper digestive system diseases, gallbladder and biliary diseases, appendicitis, and inguinal femoral and abdominal hernia rank the top four cases of incidence, while cirrhosis and other chronic liver diseases, upper digestive system diseases, paralytic ileus and intestinal obstruction, and gallbladder and biliary diseases rank the top four cases of both deaths and DALYs. The magnitude changes in the numbers were probably due to the aging trends and the growth of the world population. According to the reports from World Population Prospects 2019, the estimated number of populations had increased by 46.15%, from 5.33 billion to 7.79 billion from 1990 to 2019, and the scale of people over 60 years also increased significantly from 1990–2019 (9).

According to a previous study, there were nearly 135.9 billion dollars in the USA because of gastrointestinal diseases annually, including pancreatic, liver, and luminal, and the economic burdens are likely to keep an increasing trend (10). Moreover, most diseases of the digestive system can lead to sepsis, it was considered a major public health problem and was estimated to cost the U.S. health care system more than $20 billion a year in 2011 (11–13). In the current study, although the ASDR and ASDALYs decreased during 1990–2019, the ASIR increased, and a high increase of ASIR, ASDR, and ASDALYs was observed in various regions, which suggests the burden of digestive diseases was still a global health problem in 2019, calling for the design of flexible and country-appropriate methods to reduce the disease burdens. Besides, the current study is the first study that explored the pertinence between the EAPC and ASRs in 1990 and between ASRs and HDI both in 1990 and 2019. We found the EAPCs were significantly associated with the baseline ASRs for the overall digestive diseases and most causes, and negative correlations between ASDR, ASDALYs, and HDI in the corresponding year were also noticed. In comparison, positive correlations were observed between baseline ASIR and HDI 1990 for paralytic ileus and intestinal obstruction, inflammatory bowel disease, vascular intestinal disorders, gallbladder and biliary diseases, and pancreatitis.

Since the studies comprehensively explored the temporal trend of the incidence, deaths, and DALYs of digestive diseases, it is difficult for the current results to be directly compared with previous studies. Nevertheless, several reports for GBD 2017 studies suggested that the deaths and DALYs of cirrhosis and other chronic liver diseases and inflammatory bowel disease had increased greatly from 1990–2017, even though the ASDR and ASDALYs decreased in most regions, which is consistent with the current study (4, 5). Besides, we also observed a substantial increase in incident cases as well as a decreasing trend in ASIR for cirrhosis and other chronic liver diseases and inflammatory bowel disease from 1990 to 2019. Similarly, we found rising all-age counts of incidence, death, and DALYs as well as decreasing trends of ASIR, ASDR, and ASDALYs of pancreatitis from 1990–2019, which contrasts with the increasing trends of the age-standardized years lived with disability and prevalence rates from 1990–2017 (6).

The temporal trends in ASRs were different across various causes of digestive diseases. The gallbladder and biliary diseases, appendicitis, and paralytic ileus and intestinal obstruction dominated the incident trend of digestive diseases, with the ASIR increasing by 59, 58, and 22% annually, while a decreasing trend of ASIR was noticed in cirrhosis and other chronic liver diseases, inguinal femoral and abdominal hernia, inflammatory bowel disease, vascular intestinal disorders, and pancreatitis, and a stable trend of ASIR was observed in upper digestive system diseases. Consistent with previous GBD 2017 studies, besides cirrhosis and other chronic liver diseases and inflammatory bowel disease, the trends of deaths and DALYs of the overall digestive diseases and other seven causes were also decreasing between 1990–2019 (4, 5). Cirrhosis and other chronic liver diseases and upper digestive system diseases were two causes that presented with the highest decreasing trend of ASDR and ASDALYs, which is promising since they dominated the deaths and DALYs burden of digestive diseases in 2019. However, a clear understanding of the incidence, deaths, and DALYs rates of each digestive disease’s cause is essential for the policymakers of the healthcare system to apply systematic and target interventions that could prevent morbidity and premature deaths of digestive diseases.

Temporal trends in ASIR also varied at the regional and national levels. For overall digestive diseases and cirrhosis and other chronic liver diseases, upper digestive system diseases, inflammatory bowel disease, and vascular intestinal disorders, a slightly increasing and stable trend of ASIR was noticed in the middle, low-middle, and low SDI regions, while decreasing trends existed in other SDI regions. From 1990–2017, the increasing trends of ASYR and ASPR of pancreatitis suggested an increasing trend of burden over time, especially in regions with lower SDI (6), which is identical with the decreasing trend of ASIR for pancreatitis observed in the middle, high-middle, and high SDI regions in the current study. Although the decreasing trend of ASIR was noticed in higher SDI regions for most causes, the absolute cases and ASIR were also consistently higher in countries from higher SDI regions such as Australia, Canada, the USA, and the UK. The relatively lower incident cases and ASIR of various causes such as inflammatory bowel disease, pancreatitis, and vascular intestinal disorders in lower SDI regions might be due to the relatively lower shortages of tobacco and alcohol, and thus lower consumption of alcohol and smoking (6). The diverse dietary habits among different SDI regions may also contribute to such observing findings (14, 15). Alternatively, lower physical activity, more hygienic environments, urbanization, high BMI, and aging might contribute to higher incidence and ASIR in higher SDI regions (14, 15). Nevertheless, the general increasing trends of ASIR in lower SDI regions suggest that basic sanitation in many low-income countries is still a public issue. A promising finding is that the ASDR and ASDALYs of the nine causes declined in all SDI regions. The development of early diagnosis technology, the conduct of effective interventions at the optimal time, and improved supportive care may benefit in decreasing the global digestive burden (4, 16, 17) Additionally, the ASDR and ASDALYs also increased from 1990–2019 for the overall digestive diseases, cirrhosis and other chronic liver diseases, and vascular intestinal disorders in Eastern Europe and Central Asia, which suggests that digestive diseases are a global health problem, and not constrained to regions and countries with high or low SDI. Moreover, general negative correlations between EAPC and ASIR, as well as between ASIR and HDI in the corresponding year, were noticed, while positive correlations between ASIR and HDI both in 1990 and 2019 were observed for paralytic ileus and intestinal obstruction, inflammatory bowel disease, vascular intestinal disorders, gallbladder and biliary diseases, and pancreatitis. With the development of HDI global, our results may be partly explained by the fact that risk factors for digestive diseases, such as occupational and social factors, have changed considerably over the past 29 years, particularly in high HDI regions, while they have changed less in low HDI countries the (8). Future analysis focusing on the certain risk factors that contribute to the overall digestive diseases and by cause is required to better approach the prevention programs of digestive diseases.

In our findings, we observed gender and age differences in the global cases of incidence, deaths, and DALYs as well as the ASRs for digestive diseases by cause, with females having higher incident cases and ASIR for the overall digestive diseases, upper digestive system diseases, appendicitis, and gallbladder and biliary diseases at all ages. Particularly for gallbladder and biliary diseases, the frequency is more than two times higher in females than in males. Alternatively, the burden of deaths and DALYs were higher in males for the overall digestive diseases and most causes, except vascular intestinal disorders and gallbladder and biliary diseases. Traditionally, pancreatitis was considered a disease in men because of the higher cigarette and alcohol intake rate (6, 18). However, several studies have suggested that idiopathic causes, autoimmune diseases, and gallstones were more common in females, and factors such as hormonal and biological influences, post-partum, and pregnancy would contribute to the sex disparity of our findings (19, 20).

However, males had relatively lower incident cases but more frequent deaths compared with females. One possible explanation for this finding is that males are more likely to engage in riskier behaviors such as heavy alcohol consumption and smoking, which are known risk factors for many digestive diseases. These behaviors may contribute to a higher risk of developing severe forms of digestive diseases, which may result in more frequent deaths. In contrast, females may be more likely to seek medical attention earlier for digestive diseases, leading to earlier diagnosis and treatment, which may contribute to a lower risk of developing severe forms of the disease. Further research is needed to fully understand the underlying factors that contribute to this gender difference.

In GBD 2019, we observed that the highest burden of digestive diseases had shifted from 25–29 years in 1990 to 30–34 years in 2019 for incidence, from 60–64 years in 1990 to 65–69 years in 2019 for deaths, and remained stable in 55–59 years for DALYs, which implies that focusing on age-specific policies toward the early screening and target interventions would greatly reduce the global burden of digestive diseases. Of notice, higher deaths and DALYs burden were noticed in children aged 1–4 than 5–20, which might be due to the infantile hepatitis syndrome and biliary atresia in children. If these diseases had not been treated appropriately and timely, liver failure and worse life quality would have occurred. Therefore, additional health attention is required to minimize the digestive disease burden in children.

For all we know, the current study is the first that systematically and comprehensively explored the trends of digestive diseases at the global levels between 1990–2019, which would provide guidelines for follow-up studies and policies design. Nevertheless, several limitations require to be recognized. Firstly, since the current research is secondary data from the GBD 2019 study, the precision of the findings largely hinges on the quantity and quality of data used in the modeling. Secondly, data about the incidence, deaths, and DALYs of other digestive diseases is exclusive of our research owing to the unavailability of the data. Thirdly, even within countries and territories with the same HDI, the substantial differentiation in health gains also varies, and the effect of the health systems and policies in various locations should also be evaluated. Moreover, the risk factors that contribute to each cause of digestive diseases should be comparatively investigated.

## Conclusion

Globally, digestive diseases remained a major public health burden, with larger variation across countries, sexes, and age groups. The global ASIR of overall digestive diseases, gallbladder and biliary diseases, appendicitis, and paralytic ileus and intestinal obstruction had increased over the past three decades, whereas a decreasing and stable trend of ASIR was noticed in cirrhosis and other chronic liver diseases, upper digestive system diseases, inguinal femoral and abdominal hernia, inflammatory bowel disease, vascular intestinal disorders, and pancreatitis. Decreasing trends of ASDR and ASDALYs exist for the overall digestive diseases and by causes. Moreover, the generally negative associations between HDI and the burden of digestive diseases suggests that more aggressive and earlier medical interventions are needed in HDI-low regions. The current study’s findings would guide the development of better prevention and management strategies as well as relevant health policies to reduce the future burden of digestive diseases.

## Data availability statement

The original contributions presented in the study are included in the article/Supplementary material, further inquiries can be directed to the corresponding author.

## Author contributions

FW: conceptualization, methodology, and writing review and editing. DH: conceptualization, data curation, formal analysis, methodology, software, visualization, and writing an original draft. HS: methodology, software, and visualization. ZY, YW, LW, and TZ: conceptualization, data curation, validation, software, and visualization. NM, CZ, QZ, WH, and GY: data curation and supervision. YZ: software and writing review and editing. All authors contributed to the article and approved the submitted version.

## Funding

This study was supported by the National Natural Science Foundation of China (81602115), the Research Fund of Anhui Institute of translational medicine (2022zhyx-C35), the Natural Science Foundation for the Higher Education Institutions of Anhui Province of China (2022AH051145), the Foundation of Supporting Program for the Excellent Young Faculties in Universities of Anhui Province in China (gxyq2019012), and the Outstanding Youth from the First Affiliated Hospital of Anhui Medical University.

## Conflict of interest

The authors declare that the research was conducted in the absence of any commercial or financial relationships that could be construed as a potential conflict of interest.

## Publisher’s note

All claims expressed in this article are solely those of the authors and do not necessarily represent those of their affiliated organizations, or those of the publisher, the editors and the reviewers. Any product that may be evaluated in this article, or claim that may be made by its manufacturer, is not guaranteed or endorsed by the publisher.

## Footnotes

^1^ http://hdr.undp.org/en/composite/trends
