# Supplementary material for: Global, regional, and national burden of digestive diseases: findings from the global burden of disease study 2019
Source: Front Public Health. 2023 Aug 24;11:1202980. doi: 10.3389/fpubh.2023.1202980 (PMC10483149; doi:10.3389/fpubh.2023.1202980)
Supplement: Supplementary file 1 [file Table_1.doc]

| Table S1. The Incidence, Death, and DALYs of COCLD in 1990 and 2019 | | | | | | | | | | | | | | | |
| --- | --- | --- | --- | --- | --- | --- | --- | --- | --- | --- | --- | --- | --- | --- | --- |
| Characteristics | 1990 | | 2019 | | 1990-2019 | 1990 | | 2019 | | 1990-2019 | 1990 | | 2019 | | 1990-2019 |
|  | Incidence cases  No×10^3^ (95%UI) | ASR per 100 000  No (95% UI) | Incidence cases  No×10^3^ (95%UI) | ASR per 100 000  No (95% UI) | EAPC  No (95% CI) | Death cases  No×10^3^ (95%UI) | ASR per 100 000  No (95% UI) | Death cases  No×10^3^ (95%UI) | ASR per 100 000  No (95% UI) | EAPC  No (95% CI) | DALYs  No×10^5^ (95%UI) | ASR per 100 000  No×10^2^ (95% UI) | DALYs  No×10^5^ (95%UI) | ASR per 100 000  No×10^2^ (95% UI) | EAPC  No (95% CI) |
| Global | 1274.02(1027.19-1548.53) | 25.66(20.25-31.63) | 2051.55(1661.43-2478.13) | 25.35(20.78-30.44) | -0.03(-0.05 - -0.01) | 1012.98(948.94-1073.88) | 24.43(22.93-25.73) | 1472.01(1374.61-1578.73) | 18(16.8-19.31) | -1.1(-1.2 - -1) | 347.28(323.83-371.33) | 7.66(7.18-8.13) | 461.89(430.27-495.51) | 5.6(5.22-6.02) | -1.08(-1.19 - -0.97) |
| Sex |  |  |  |  |  |  |  |  |  |  |  |  |  |  |  |
| Female | 510.59(414.22-619.45) | 21.04(16.88-25.68) | 845.43(687.51-1016.95) | 20.91(17.22-25.15) | -0.06(-0.09 - -0.02) | 351.19(324.34-383.64) | 16.17(14.94-17.54) | 502.94(459.2-550.91) | 11.7(10.68-12.81) | -1.2(-1.28 - -1.12) | 113.56(102.55-127.72) | 4.87(4.44-5.42) | 144.08(131.6-157.59) | 3.44(3.14-3.77) | -1.29(-1.38 - -1.19) |
| Male | 763.43(600.54-940.38) | 30.1(23.39-37.32) | 1206.12(964.22-1464.59) | 29.67(23.86-35.98) | -0.05(-0.07 - -0.02) | 661.78(607.81-705.66) | 33.55(30.76-35.64) | 969.07(899.21-1045.34) | 24.81(23.07-26.75) | -1.12(-1.22 - -1.03) | 233.72(213.53-249.98) | 10.54(9.63-11.25) | 317.81(293.66-344.38) | 7.83(7.24-8.49) | -1.15(-1.25 - -1.04) |
| SDI |  |  |  |  |  |  |  |  |  |  |  |  |  |  |  |
| Low SDI | 95.75(75.24-118.16) | 21.19(15.47-27.42) | 223.34(178.08-272.66) | 23.12(17.74-29.01) | 0.49(0.40 - 0.57) | 119(103.34-138.26) | 45.54(39.29-52.2) | 187.88(163.82-215.15) | 32.78(28.9-37.11) | -1.19(-1.32 - -1.05) | 43.7(36.67-52.28) | 13.35(11.59-15.51) | 67.73(58.67-78.46) | 9.5(8.27-10.89) | -1.21(-1.35 - -1.07) |
| Low-middle SDI | 216.43(170.80-269.32) | 21.25(15.83-27.12) | 434.43(338.93-537.80) | 25.01(19.45-31.04) | 0.69(0.63 - 0.75) | 229.6(208.53-256.46) | 34.02(30.58-37.77) | 376.21(342.15-416.61) | 26.21(23.86-28.99) | -0.92(-1.02 - -0.82) | 88.01(79.52-99.97) | 10.77(9.83-12.04) | 129.74(117.42-143.7) | 8.18(7.4-9.08) | -0.95(-1.05 - -0.85) |
| Middle SDI | 399.43(303.2-502.11) | 26.33(19.56-33.53) | 658.88(521.51-805.13) | 24.67(19.68-30.06) | 0.04(-0.05 - 0.13) | 315.04(292.67-335.85) | 29.61(27.23-31.87) | 469.61(427.79-516.79) | 19.23(17.45-21.15) | -1.54(-1.6 - -1.48) | 108.72(101.2-115.72) | 8.5(7.93-9.04) | 140.74(128.98-154.06) | 5.39(4.94-5.9) | -1.66(-1.72 - -1.61) |
| High-middle SDI | 311.25(249.41-377.47) | 27(21.64-32.89) | 419.42(339.20-505.03) | 24.8(20.45-29.77) | -0.26(-0.29 - -0.22) | 198.12(188.48-207.3) | 18.42(17.5-19.25) | 251.94(236.06-269.28) | 12.81(12-13.68) | -1.35(-1.6 - -1.1) | 62.7(59.46-66.02) | 5.58(5.29-5.87) | 76.39(71.69-81.67) | 4.02(3.78-4.3) | -1.22(-1.52 - -0.92) |
| High SDI | 250.61(213.21-289.26) | 28.05(23.9-32.31) | 268.35(231.08-305.72) | 23.91(20.82-27.14) | -0.67(-0.72 - -0.61) | 150.66(145.31-153.95) | 15.18(14.65-15.5) | 185.5(173.74-196.1) | 10.77(10.23-11.31) | -1.3(-1.34 - -1.25) | 43.96(42.69-44.77) | 4.59(4.46-4.68) | 47.02(45.09-48.82) | 3.08(2.96-3.19) | -1.5(-1.55 - -1.45) |
| Region |  |  |  |  |  |  |  |  |  |  |  |  |  |  |  |
| Andean Latin America | 7.21(6.32-8.21) | 23.23(19.92-26.85) | 19.97(17.61-22.67) | 32.25(28.4-36.69) | 1.06(0.87 - 1.25) | 7.03(6.07-8.22) | 31.39(27.21-36.49) | 14.05(11.23-17.33) | 25.08(20.08-30.92) | -0.8(-0.87 - -0.73) | 2.46(2.11-2.94) | 9.28(8.03-10.88) | 3.73(2.94-4.65) | 6.39(5.06-7.96) | -1.4(-1.48 - -1.32) |
| Australasia | 2.39(2.05-2.68) | 11.12(9.53-12.49) | 3.31(2.80-3.82) | 10.26(8.75-11.72) | -0.31(-0.37 - -0.24) | 1.59(1.5-1.65) | 7.04(6.66-7.33) | 2.51(2.3-2.73) | 5.48(5.05-5.93) | -0.72(-0.89 - -0.56) | 0.45(0.44-0.47) | 2.04(1.95-2.12) | 0.63(0.59-0.68) | 1.53(1.43-1.64) | -0.77(-0.94 - -0.59) |
| Caribbean | 6.32(5.51-7.15) | 20.23(17.44-23.03) | 10.84(9.20-12.55) | 21.85(18.56-25.08) | 0.05(-0.04 - 0.13) | 6.21(5.47-6.8) | 23.12(20.65-25.14) | 9.55(7.78-11.41) | 18.52(15.04-22.19) | -1.01(-1.27 - -0.74) | 2.03(1.73-2.32) | 7.01(6.06-7.85) | 2.78(2.2-3.41) | 5.46(4.29-6.73) | -1.09(-1.39 - -0.8) |
| Central Asia | 19.49(17.32-21.89) | 32.78(28.84-36.91) | 58.16(51.50-64.78) | 59.06(52.3-66.01) | 2.24(2.09 - 2.39) | 13.65(13.22-14.24) | 27.66(26.72-28.82) | 33.91(30.49-37.74) | 42.86(38.53-47.51) | 1.37(0.93 - 1.81) | 4.62(4.47-4.83) | 8.48(8.22-8.86) | 11.73(10.54-13.09) | 13.18(11.87-14.67) | 1.31(0.81-1.8) |
| Central Europe | 42.87(38.64-47.11) | 32.86(29.48-36.13) | 37.56(33.21-41.70) | 29.11(25.85-32.45) | -0.44(-0.61 - -0.27) | 32.71(31.99-33.44) | 22.45(21.95-22.94) | 33.6(29.32-37.9) | 17.71(15.42-20.04) | -1.27(-1.5 - -1.04) | 10.17(9.96-10.4) | 7.08(6.94-7.23) | 9.65(8.4-10.92) | 5.55(4.83-6.28) | -1.36(-1.62 - -1.1) |
| Central Latin America | 55.70(43.84-68.60) | 42.69(33.05-53.15) | 106.17(85.02-127.87) | 40.76(32.74-48.98) | -0.06(-0.11 - -0.01) | 33.7(32.89-34.37) | 36.17(35.09-36.98) | 68.06(58.61-78.33) | 28.32(24.45-32.59) | -1.15(-1.27 - -1.02) | 11.96(11.7-12.23) | 11.09(10.85-11.32) | 20.32(17.41-23.44) | 8.17(7.01-9.4) | -1.4(-1.56 - -1.24) |
| Central Sub-Saharan Africa | 10.86(8.87-13.03) | 22.72(17.23-28.6) | 30.83(25.65-36.78) | 26.97(21.49-33.11) | 0.52(0.44 - 0.61) | 13.19(10.83-15.84) | 51.94(43.25-61.81) | 22.76(17.09-29.11) | 36.98(27.96-47.37) | -1.2(-1.25 - -1.14) | 4.84(3.94-5.83) | 15.16(12.43-18.19) | 8.43(6.29-11.07) | 10.9(8.21-13.93) | -1.15(-1.2 - -1.09) |
| East Asia | 324.50(238.79-415.38) | 27.29(19.59-35.34) | 424.36(321.44-529.85) | 22.51(17.71-27.56) | -0.57(-0.66 - -0.48) | 176.94(153.89-201.34) | 19.28(16.96-21.79) | 164.72(140.15-191.75) | 8.18(7.01-9.46) | -3.08(-3.26 - -2.89) | 59.97(51.78-68.48) | 5.79(5.01-6.59) | 46.99(39.82-55.13) | 2.28(1.93-2.66) | -3.38(-3.54 - -3.22) |
| Eastern Europe | 46.01(35.15-58.47) | 19.87(15.35-24.85) | 66.87(47.65-90.00) | 31.27(23.38-41.1) | 1.90(1.61 - 2.18) | 29.21(28.21-30.57) | 10.62(10.24-11.13) | 72.72(64.99-81.01) | 24.29(21.67-27.04) | 2.97(2.12- 3.83) | 9.15(8.84-9.58) | 3.39(3.27-3.55) | 25.75(22.99-28.75) | 9.2(8.21-10.27) | 3.54(2.55-4.54) |
| Eastern Sub-Saharan Africa | 35.77(27.95-44.40) | 25.39(17.98-33.88) | 86.02(67.76-105.52) | 27.15(19.8-35.41) | 0.18(0.12 - 0.25) | 47.88(38.21-57.39) | 59.24(48.67-70.2) | 76.98(66.31-91.42) | 44.15(38.47-51.91) | -1.11(-1.21 - -1.01) | 16.49(12.77-20.09) | 16.43(13.08-19.74) | 26.08(21.91-31.51) | 11.78(10.16-14.02) | -1.25(-1.35 - -1.15) |
| High-income Asia Pacific | 77.47(63.53-92.06) | 39.1(32.45-45.93) | 50.83(41.60-60.07) | 25.15(21.29-29.18) | -1.63(-1.7 - -1.56) | 41.42(39.69-42.46) | 20.84(19.87-21.41) | 36.89(32.21-41.08) | 8.69(7.87-9.38) | -3.24(-3.09 - -3.39) | 12.27(11.59-12.59) | 6(5.66-6.15) | 7.77(7.17-8.24) | 2.3(2.16-2.43) | -3.59(-3.72- -3.46) |
| High-income North America | 72.04(56.09-89.23) | 23.46(18.26-29.25) | 98.64(81.10-116.83) | 25.62(21.47-30.08) | 0.16(0.06 - 0.25) | 40.54(38.95-41.45) | 12.22(11.76-12.47) | 72.74(69.31-75.46) | 12.67(12.16-13.11) | 0.31(0.23-0.38) | 11.79(11.46-11.99) | 3.72(3.62-3.78) | 19.55(18.93-20.14) | 3.71(3.6-3.82) | 0.21(0.13-0.29) |
| North Africa and Middle East | 60.84(51.04-72.59) | 23.79(19.02-29.18) | 160.14(133.54-190.70) | 28.68(23.58-34.88) | 0.55(0.53 - 0.58) | 68.42(62.59-73.4) | 43.94(38.78-48.69) | 109.71(81.39-135.2) | 27.73(21.06-33.88) | -1.56(-1.63 - -1.48) | 19.69(17.42-21.68) | 9.79(8.98-10.41) | 28.78(21.26-35.65) | 6.17(4.58.-7.62) | -1.56(-1.62 - -1.51) |
| Oceania | 0.58(0.47-0.69) | 9.78(7.86-11.7) | 1.12(0.94-1.32) | 8.5(7.05-10) | -0.68(-0.75 - -0.61) | 0.61(0.51-0.74) | 16.57(13.81-20.07) | 1.15(0.91-1.45) | 13.19(10.56-16.35) | -0.79(-0.85 - -0.73) | 0.25(0.21-0.3) | 5.57(4.6-6.74) | 0.46(0.36-0.58) | 4.41(3.48-5.5) | -0.81(-0.86 - -0.77) |
| South Asia | 185.67(140.26-240.70) | 17.27(12.06-23.13) | 413.98(299.63-539.34) | 22.98(16.68-29.92) | 1.19(1.11 - 1.28) | 202.42(181.65-236.94) | 30.95(27.32-35.62) | 348.39(306.92-404.85) | 23.49(20.74-27.13) | -0.98(-1.16 - -0.8) | 81.12(72.92-95.83) | 9.94(8.93-11.59) | 124.36(109.54-144.14) | 7.51(6.62-8.68) | -0.97(-1.14 - -0.79) |
| Southeast Asia | 105.67(80.37-133.40) | 26.34(19.13-34.21) | 181.54(142.58-219.74) | 24.76(19.5-30) | -0.43(-0.52 - -0.34) | 119.26(107.51-130.47) | 41.61(36.9-46.43) | 186.15(165.36-207.7) | 30.21(26.88-33.49) | -1.18(-1.21 - -1.15) | 44.32(39.25-48.59) | 12.97(11.74-14.09) | 59.15(52.07-66.43) | 8.67(7.65-9.71) | -1.5(-1.55 - -1.45) |
| Southern Latin America | 12.19(10.70-13.67) | 25.94(22.69-29.19) | 22.2(19.63-24.76) | 30.5(27.07-34.08) | 0.38(0.3 - 0.47) | 10.41(10.04-10.74) | 22.51(21.67-23.23) | 14.15(13.22-15.12) | 17.34(16.22-18.49) | -0.77(-0.89 - -0.64) | 3.15(3.05-3.24) | 6.71(6.5-6.9) | 3.72(3.52-3.95) | 4.72(4.46-4.99) | -1.05(-1.21 - -0.89) |
| Southern Sub-Saharan Africa | 9.08(7.03-11.34) | 19.3(13.89-24.97) | 12.18(9.24-15.40) | 15.57(11.7-19.87) | -0.96(-1.11 - -0.82) | 7.01(5.82-8.57) | 22.65(18.51-28.31) | 9.23(8.23-10.32) | 15.43(13.82-17.16) | -1.54(-1.9 - -1.17) | 2.58(2.21-3.07) | 7.16(6.02-8.64) | 3.11(2.73-3.54) | 4.59(4.05-5.17) | -1.73(-2.15 - -1.32) |
| Tropical Latin America | 35.90(26.18-46.46) | 26.07(18.49-33.97) | 50.19(36.24-65.54) | 19.79(14.41-25.57) | -1.26(-1.39 - -1.14) | 24.77(23.81-25.53) | 24.17(23.07-24.96) | 38.78(36.55-41.26) | 15.72(14.81-16.75) | -1.46(-1.51- -1.42) | 9.18(8.86-9.45) | 7.82(7.53-8.05) | 11.93(11.34-12.61) | 4.74(4.5-5.01) | -1.75(-1.8 - -1.71) |
| Western Europe | 124.64(110.53-138.37) | 30.31(26.95-33.51) | 116.18(102.87-129.25) | 24.45(21.91-26.93) | -0.86(-0.98 - -0.74) | 85.8(82.48-87.7) | 16.02(15.44-16.35) | 77.23(72.04-82.46) | 9.41(8.93-9.99) | -2.1(-2.19- -2.01) | 23.29(22.64-23.71) | 4.68(4.56-4.76) | 18.33(17.52-19.3) | 2.61(2.51-2.74) | -2.31(-2.42 - -2.21) |
| Western Sub-Saharan Africa | 38.82(30.82-47.73) | 23.93(17.03-31.63) | 100.45(80.81-121.91) | 26.22(19.92-33.23) | 0.24(0.17 - 0.32) | 50.19(39.53-62.86) | 53.17(41.71-67.04) | 78.74(61.7-99.77) | 37.5(30.28-46.47) | -1.13(-1.26 - -1.01) | 17.5(13.92-21.53) | 14.94(11.71-18.8) | 28.62(21.96-36.89) | 10.52(8.23-13.38) | -1.14(-1.25 - -1.02) |
| COCLD: Cirrhosis and other chronic liver diseases; ASR, age- standardised incidence rate; EAPC, estimated annual percentage change; UI, uncertainty interval. | | | | | | | | | | | | | | | |
