# Supplementary material for: Global, regional, and national burden of digestive diseases: findings from the global burden of disease study 2019
Source: Front Public Health. 2023 Aug 24;11:1202980. doi: 10.3389/fpubh.2023.1202980 (PMC10483149; doi:10.3389/fpubh.2023.1202980)
Supplement: Supplementary file 2 [file Table_2.docx]

| Table S2. The Incidence, Death, and DALYs of UDSD in 1990 and 2019 | | | | | | | | | | | | | | | |
| --- | --- | --- | --- | --- | --- | --- | --- | --- | --- | --- | --- | --- | --- | --- | --- |
| Characteristics | 1990 | | 2019 | | 1990-2019 | 1990 | | 2019 | | 1990-2019 | 1990 | | 2019 | | 1990-2019 |
|  | Incidence cases  No×10^6^ (95%UI) | ASR per 100 000  No×10^3^ (95% UI) | Incidence cases  No×10^6^ (95%UI) | ASR per 100 000  No×10^3^ (95% UI) | EAPC  No (95% CI) | Death cases  No×10^4^ (95%UI) | ASR per 100 000  No (95% UI) | Death cases  No×10^4^ (95%UI) | ASR per 100 000  No (95% UI) | EAPC  No (95% CI) | DALYs  No×10^5^ (95%UI) | ASR per 100 000  No×10^2^ (95% UI) | DALYs  No×10^5^ (95%UI) | ASR per 100 000  No×10^2^ (95% UI) | EAPC  No (95% CI) |
| Global | 199.57(176.36-224.13) | 4.17(3.71-4.65) | 343.97(305.2-383.74) | 4.22(3.73-4.71) | 0.00(-0.04 - 0.03) | 31.05(29-33.42) | 8.22(7.66-8.83) | 27.36(25.24-29.88) | 3.48(3.19-3.8) | -3.45(-3.54 - -3.36) | 138.14(116.63-169.86) | 3.09(2.62-3.75) | 148.73(113.37-201.75) | 1.83(1.39-2.48) | -2.37(-2.45 - -2.29) |
| Sex |  |  |  |  |  |  |  |  |  |  |  |  |  |  |  |
| Female | 104.62(92.64-116.89) | 4.33(3.86-4.83) | 181.24(161.83-201.76) | 4.39(3.91-4.90) | 0.01(-0.03 - 0.05) | 12.64(11.24-14.33) | 6.17(5.5-6.92) | 12.51(11.07-13.85) | 2.88(2.55-3.19) | -2.84(-2.96 - -2.73) | 59.79(47.54-77.26) | 2.57(2.06-3.3) | 71.88(53.07-100.41) | 1.72(1.27-2.41) | -1.52(-1.59 - -1.45) |
| Male | 94.96(83.72-106.89) | 4.01(3.55-4.47) | 162.73(143.92-182.86) | 4.04(3.57-4.53) | 0.00(-0.04 - 0.04) | 18.4(16.56-20) | 10.67(9.64-11.55) | 14.85(13.51-16.6) | 4.16(3.8-4.63) | -3.18(-3.28 - -3.08) | 78.35(66.68-94.3) | 3.66(3.15-4.34) | 76.85(60.76-102.08) | 1.94(1.54-2.57) | -1.98(-2.05 - -1.9) |
| SDI |  |  |  |  |  |  |  |  |  |  |  |  |  |  |  |
| Low SDI | 18.86(16.63-21.30) | 5.24(4.67-5.86) | 40.92(36.10-46.10) | 5.01(4.47-5.60) | 0.02(-0.03 - 0.07) | 3.41(2.96-3.94) | 14.24(12.18-16.44) | 3.6(3.18-4.05) | 7.06(6.24-8.01) | -2.64(-2.81 - -2.47) | 16.87(14.15-20.53) | 5.08(4.33-6.06) | 22.67(17.96-29.32) | 3.04(2.46-3.88) | -1.95(-2.04 - -1.85) |
| Low-middle SDI | 44.45(39.27-49.84) | 5.14(4.58-5.72) | 84.77(75.12-94.73) | 5.09(4.54-5.66) | -0.02(-0.03 - 0.00) | 10.51(9.42-11.63) | 18.14(16.13-20.03) | 9.04(8.1-10.33) | 7.27(6.51-8.33) | -3.42(-3.59 - -3.525) | 44.82(38.63-52.45) | 5.93(5.2-6.84) | 44.31(35.16-58.16) | 2.88(2.32-3.72) | -2.73(-2.84 - -2.62) |
| Middle SDI | 54.87(48.47-61.60) | 3.76(3.36-4.18) | 99.06(87.89-110.57) | 3.80(3.37-4.25) | 0.16(0.10 - 0.22) | 7.8(7.11-8.66) | 8.68(7.89-9.52) | 7.5(6.78-8.21) | 3.53(3.16-3.86) | -3.12(-3.21 - -3.03) | 36.23(29.87-45.33) | 2.92(2.46-3.57) | 41.22(31.23-56.92) | 1.63(1.25-2.22) | -2.05(-2.11 - -1.99) |
| High-middle SDI | 46.39(41.10-51.92) | 4.00(3.56-4.48) | 66.88(59.66-74.74) | 3.81(3.38-4.25) | -0.24(-0.3 - -0.19) | 5.12(4.79-5.55) | 5.22(4.88-5.64) | 4.49(4.1-4.83) | 2.28(2.08-2.45) | -3.04(-3.17 - -2.92) | 24.51(19.47-32.08) | 2.2(1.76-2.84) | 25.6(18.91-36.16) | 1.39(1.01-2) | -1.78(-1.87 - -1.69) |
| High SDI | 34.88(30.73-39.21) | 3.74(3.3-4.21) | 46.77(41.64-52.74) | 3.61(3.18-4.09) | -0.21(-0.28 - -0.14) | 4.19(3.88-4.36) | 4.04(3.73-4.21) | 2.73(2.36-3.03) | 1.27(1.12-1.4) | -4.45(-4.48 - -4.22) | 15.64(12.14-21.13) | 1.6(1.23-2.19) | 14.84(10.25-22.1) | 1.04(0.69-1.6) | -1.68(-1.84 - -1.52) |
| Region |  |  |  |  |  |  |  |  |  |  |  |  |  |  |  |
| Andean Latin America | 1.95(1.73-2.18) | 6.47(5.80-7.16) | 4.07(3.63-4.51) | 6.49(5.82-7.17) | 0.01(0.00 - 0.02) | 0.18(0.16-0.21) | 9.06(7.99-10.13) | 0.22(0.18-0.27) | 4.07(3.26-5.04) | -2.59(-2.75 - -2.43) | 1.02(0.8-1.32) | 3.7(2.98-4.74) | 1.41(0.98-2.08) | 2.33(1.64-3.41) | -1.53(-1.65 - -1.42) |
| Australasia | 0.86(0.76-0.98) | 3.86(3.41-4.38) | 1.36(1.21-1.54) | 3.84(3.39-4.37) | -0.02(-0.12 - 0.07) | 0.11(0.1-0.12) | 4.92(4.39-5.32) | 0.05(0.04-0.06) | 0.96(0.8-1.13) | -5.99(-6.49 - -5.49) | 0.37(0.29-0.51) | 1.65(1.27-2.25) | 0.38(0.25-0.6) | 1.01(0.63-1.6) | -1.67(-1.99 - -1.36) |
| Caribbean | 2.04(1.81-2.27) | 6.43(5.78-7.12) | 3.24(2.92-3.58) | 6.49(5.81-7.17) | 0.02(0.02 - 0.03) | 0.19(0.17-0.22) | 7.52(6.71-8.47) | 0.21(0.17-0.25) | 4.04(3.4-4.78) | -2.33(-2.51 - -2.14) | 1.04(0.81-1.4) | 3.49(2.74-4.63) | 1.29(0.93-1.79) | 2.57(1.85-3.55) | -1.12(-1.24 - -1) |
| Central Asia | 2.56(2.25-2.89) | 4.41(3.90-5.00) | 4.06(3.55-4.62) | 4.42(3.91-5.02) | 0.01(0.01 - 0.01) | 0.2(0.19-0.21) | 4.17(3.93-4.39) | 0.25(0.22-0.28) | 3.54(3.17-4.01) | -0.94(-1.3 - -0.58) | 1.23(0.98-1.63) | 2.23(1.78-2.92) | 1.68(1.27-2.32) | 1.92(1.46-2.62) | -0.94(-1.19 - -0.68) |
| Central Europe | 6.41(5.69-7.21) | 4.73(4.19-5.30) | 7.20(6.45-8.04) | 4.79(4.26-5.36) | 0.06(0.06 - 0.07) | 0.74(0.71-0.77) | 5.42(5.15-5.62) | 0.64(0.57-0.72) | 3.01(2.65-3.41) | -2.04(-2.1 - -1.91) | 3.34(2.68-4.33) | 2.4(1.91-3.13) | 2.99(2.25-4.14) | 1.78(1.3-2.55) | -1.02(-1.08 - -0.96) |
| Central Latin America | 8.20(7.24-9.15) | 6.35(5.69-6.99) | 16.05(14.31-17.76) | 6.27(5.60-6.91) | -0.05(-0.05 - -0.04) | 0.82(0.77-0.85) | 10.71(9.98-11.18) | 0.99(0.86-1.14) | 4.39(3.82-5.05) | -3.5(-3.68 - -3.33) | 3.88(3.09-5.15) | 3.61(2.96-4.62) | 5.47(3.82-8.06) | 2.2(1.56-3.22) | -1.89(-2.02 - -1.76) |
| Central Sub-Saharan Africa | 1.79(1.58-2.05) | 4.94(4.40-5.54) | 4.59(4.03-5.21) | 5.08(4.51-5.68) | 0.09(0.09 - 0.10) | 0.27(0.21-0.33) | 11.47(8.27-14.75) | 0.41(0.3-0.55) | 8.07(5.65-10.93) | -1.08(-1.21 - -0.95) | 1.5(1.19-1.88) | 4.35(3.43-5.37) | 2.61(1.96-3.46) | 3.29(2.48-4.28) | -0.89(-0.97 - -0.82) |
| East Asia | 29.58(26.27-33.15) | 2.61(2.34-2.91) | 43.88(38.86-49.50) | 2.35(2.09-2.64) | -0.5(-0.62 - -0.38) | 7.46(6.44-9.11) | 10.01(8.74-11.97) | 6.18(5.32-7.08) | 3.48(3-3.96) | -3.42(-3.65 - -3.2) | 29.32(24.61-35.98) | 3.02(2.57-3.64) | 24.34(19.29-31.61) | 1.26(1-1.63) | -2.98(-3.11 - -2.86) |
| Eastern Europe | 12.29(10.88-13.82) | 4.82(4.27-5.43) | 12.91(11.5-14.54) | 4.85(4.28-5.45) | -0.13(-0.21 - -0.04) | 1.01(0.96-1.05) | 3.78(3.58-3.95) | 1.24(1.1-1.39) | 3.78(3.35-4.23) | -0.38(-0.7 - -0.05) | 5.72(4.51-7.68) | 2.17(1.7-2.93) | 6.11(4.77-8.12) | 2.13(1.64-2.9) | -0.51(-0.75 - -0.28) |
| Eastern Sub-Saharan Africa | 6.05(5.33-6.86) | 4.98(4.42-5.60) | 14.28(12.53-16.22) | 5.04(4.47-5.65) | 0.03(0.03 - 0.04) | 0.83(0.69-1.05) | 9.99(8.23-12.53) | 1.03(0.83-1.25) | 5.83(4.58-7.2) | -2(-2.1 - -1.89) | 4.66(3.81-5.87) | 4.09(3.35-5.15) | 7.2(5.56-9.43) | 2.82(2.19-3.64) | -1.4(-1.46 - -1.34) |
| High-income Asia Pacific | 5.33(4.68-6.12) | 2.72(2.39-3.12) | 7.14(6.33-8.19) | 2.73(2.4-3.13) | 0.14(0.05 - 0.24) | 0.82(0.75-0.88) | 4.81(4.35-5.16) | 0.59(0.47-0.7) | 1.01(0.83-1.16) | -5.7(-6.01 - -5.38) | 2.71(2.17-3.5) | 1.41(1.14-1.82) | 2.35(1.65-3.47) | 0.77(0.51-1.19) | -2.05(-2.3 - -1.79) |
| High-income North America | 14.41(12.67-16.20) | 4.57(4.03-5.14) | 18.27(16.29-20.59) | 4.05(3.56-4.59) | -0.73(-0.88 - -0.57) | 1.04(0.95-1.09) | 2.86(2.63-3) | 0.62(0.55-0.67) | 0.93(0.84-1) | -4.46(-4.9 - -4.02) | 5.36(3.9-7.59) | 1.64(1.18-2.35) | 5.27(3.46-8.03) | 1.1(0.7-1.71) | -1.74(-1.96 - -1.51) |
| North Africa and Middle East | 13.24(11.56-14.98) | 5.06(4.49-5.70) | 30.03(26.45-33.88) | 5.09(4.54-5.70) | 0.03(0.01 - 0.04) | 0.92(0.77-1.15) | 5.94(4.78-7.77) | 1(0.83-1.21) | 2.69(2.23-3.23) | -2.64(-2.69 - -2.59) | 5.77(4.39-7.9) | 2.48(1.93-3.3) | 9.31(6.19-14.23) | 1.7(1.17-2.53) | -1.26(-1.32 - -1.21) |
| Oceania | 0.13(0.11-0.15) | 2.61(2.31-2.95) | 0.29(0.25-0.33) | 2.60(2.30-2.95) | -0.02(-0.03 - -0.01) | 0.03(0.03-0.04) | 11.79(9.12-14.44) | 0.05(0.04-0.06) | 7.4(5.95-9.13) | -1.66(-1.69 - -1.62) | 0.16(0.12-0.19) | 3.68(2.9-4.5) | 0.25(0.2-0.31) | 2.56(2.07-3.13) | -1.29(-1.31 - -1.26) |
| South Asia | 48.28(42.52-54.33) | 5.72(5.09-6.36) | 98.71(87.46-110.64) | 5.72(5.10-6.38) | 0.01(0.00 - 0.02) | 9.99(8.67-11.27) | 18.67(16.09-21.15) | 7.88(6.71-9.42) | 6.22(5.29-7.46) | -4.32(-4.61 - -4.03) | 44.18(37.49-52.18) | 6.09(5.2-7.09) | 44.89(33.97-61.23) | 2.8(2.14-3.74) | -3.06(-3.22 - -2.9) |
| Southeast Asia | 9.88(8.64-11.22) | 2.60(2.30-2.95) | 18.11(15.96-20.61) | 2.59(2.29-2.94) | 0.00(-0.01 - 0.01) | 2.12(1.88-2.35) | 9.05(7.96-10.11) | 2.04(1.82-2.33) | 3.94(3.49-4.47) | -3.17(-3.31 - -3.03) | 9.03(7.68-10.84) | 2.78(2.41-3.23) | 9.5(7.52-12.39) | 1.46(1.18-1.87) | -2.4(-2.49 - -2.32) |
| Southern Latin America | 2.53(2.24-2.86) | 5.29(4.69-5.98) | 3.91(3.47-4.41) | 5.28(4.67-5.96) | -0.12(-0.16 - -0.08) | 0.16(0.15-0.17) | 3.67(3.37-3.93) | 0.14(0.13-0.16) | 1.7(1.48-1.93) | -2.7(-2.99 - -2.41) | 0.94(0.68-1.36) | 2.01(1.45-2.88) | 1.17(0.76-1.83) | 1.55(0.99-2.43) | -1(-1.11 - -0.89) |
| Southern Sub-Saharan Africa | 2.01(1.78-2.27) | 5.03(4.47-5.65) | 3.74(3.29-4.22) | 5.10(4.52-5.72) | 0.05(0.04 - 0.06) | 0.2(0.15-0.25) | 7.18(5.21-9.15) | 0.34(0.3-0.37) | 6.51(5.87-7.2) | 0.03(-0.45 - 0.52) | 1.14(0.88-1.49) | 3.07(2.37-3.96) | 1.84(1.45-2.45) | 2.72(2.18-3.54) | -0.16(-0.53-0.21) |
| Tropical Latin America | 9.01(8.01-9.97) | 6.89(6.19-7.54) | 16.06(14.36-17.65) | 6.51(5.83-7.16) | -0.28(-0.31 - -0.24) | 0.54(0.51-0.57) | 6.45(5.97-6.8) | 0.6(0.54-0.65) | 2.55(2.29-2.8) | -3.3(-3.5 - -3.1) | 3.78(2.83-5.22) | 3.24(2.5-4.38) | 5.02(3.38-7.6) | 2.04(1.37-3.07) | -1.73(-1.82 - -1.64) |
| Western Europe | 16.66(14.81-18.84) | 3.62(3.2-4.08) | 20.61(18.31-23.31) | 3.61(3.18-4.09) | 0.01(-0.01 - 0.02) | 2.46(2.26-2.58) | 4.23(3.87-4.43) | 1.46(1.27-1.63) | 1.36(1.2-1.5) | -4.46(-4.69 - -4.24) | 7.92(6.31-10.59) | 1.55(1.2-2.12) | 6.76(4.72-9.98) | 1.04(0.69-1.59) | -1.54(-1.66 - -1.43) |
| Western Sub-Saharan Africa | 6.34(5.58-7.21) | 4.82(4.27-5.43) | 15.45(13.54-17.59) | 4.84(4.28-5.45) | 0.01(0.00 - 0.01) | 0.95(0.72-1.32) | 10.35(7.79-14.11) | 1.42(1.03-1.9) | 7.26(5.44-9.4) | -0.95(-1.07 - -0.84) | 5.07(3.91-6.71) | 3.84(2.95-5.04) | 8.89(6.49-11.99) | 2.94(2.19-3.93) | -0.82(-0.88 - -0.75) |
| UDSD: Upper digestive system diseases; ASR, age- standardised incidence rate; EAPC, estimated annual percentage change; UI, uncertainty interval. | | | | | | | | | | | | | | | |
