# Supplementary material for: Global, regional, and national burden of digestive diseases: findings from the global burden of disease study 2019
Source: Front Public Health. 2023 Aug 24;11:1202980. doi: 10.3389/fpubh.2023.1202980 (PMC10483149; doi:10.3389/fpubh.2023.1202980)
Supplement: Supplementary file 3 [file Table_3.docx]

| Table S3. The Incidence, Death, and DALYs of APED in 1990 and 2019 | | | | | | | | | | | | | | | |
| --- | --- | --- | --- | --- | --- | --- | --- | --- | --- | --- | --- | --- | --- | --- | --- |
| Characteristics | 1990 | | 2019 | | 1990-2019 | 1990 | | 2019 | | 1990-2019 | 1990 | | 2019 | | 1990-2019 |
|  | Incidence cases  No×10^5^ (95%UI) | ASR per 100 000  No×10^2^ (95% UI) | Incidence cases  No×10^5^ (95%UI) | ASR per 100 000  No×10^2^ (95% UI) | EAPC  No (95% CI) | Death cases  No×10^3^ (95%UI) | ASR per 100 000  No (95% UI) | Death cases  No×10^3^ (95%UI) | ASR per 100 000  No (95% UI) | EAPC  No (95% CI) | DALYs  No×10^4^ (95%UI) | ASR per 100 000  No×10 (95% UI) | DALYs  No×10^4^ (95%UI) | ASR per 100 000  No×10 (95% UI) | EAPC  No (95% CI) |
| Global | 108.22(84.2-138.39) | 1.91(1.50-2.41) | 176.99(141.01-223.25) | 2.3(1.81-2.91) | 0.58(0.44 - 0.71) | 42.77(31.84-53.17) | 0.93(0.71-1.12) | 33.42(27.26-38.06) | 0.43(0.35-0.49) | -2.81(-2.88 - -2.75) | 220.59(156-283.74) | 41.27(29.68-52.38) | 150.15(123.62-170.58) | 19.35(15.92-21.98) | -2.77(-2.84 - -2.70) |
| Sex |  |  |  |  |  |  |  |  |  |  |  |  |  |  |  |
| Female | 56.72(4.43-71.94) | 2.02(1.59-2.54) | 93.77(7.47-117.63) | 2.47(1.96-3.11) | 0.66(0.54 - 0.78) | 20.89(14.24-28.27) | 0.79(0.54-1.06) | 17.08(13.19-19.74) | 0.44(0.34-0.51) | -2.63(-2.70 - -2.56) | 111.68(74.78-137.12) | 42.53(28.85-51.29) | 72.68(55.22-85.49) | 18.81(14.32-22.09) | -2.96(-3.02 - -2.89) |
| Male | 51.49(3.98-66.03) | 1.80(1.41-2.28) | 83.22(6.62-105.32) | 2.13(1.68-2.71) | 0.49(0.33 - 0.64) | 21.88(14.87-26.25) | 0.81(0.55-0.97) | 16.34(12.16-19.41) | 0.42(0.31-0.50) | -3.00(-3.07 - -2.94) | 108.91(69.5-153.05) | 40.31(26.35-55.70) | 77.47(60.4-89.75) | 20.01(15.70-23.17) | -2.63(-2.70 - -2.56) |
| SDI |  |  |  |  |  |  |  |  |  |  |  |  |  |  |  |
| Low SDI | 7.32(5.76-9.19) | 1.33(1.07-1.64) | 20.12(15.37-25.94) | 2.45(1.85-3.23) | 0.64(0.46 - 0.81) | 8.67(4.69-13.26) | 2.43(1.48-3.52) | 7.66(5.45-9.96) | 0.68(0.48-0.88) | -2.91(-3.08 - -2.75) | 47.98(23.9-75.84) | 96.50(53.76-145.82) | 39.26(28.48-50.50) | 39.82(28.82-51.16) | -3.30(-3.46 - -3.14) |
| Low-middle SDI | 24.83(19.76-31.19) | 2.01(1.64-2.48) | 53(41.97-67.12) | 2.72(2.06-3.51) | 1.04(0.95 - 1.13) | 18.04(12.89-22.90) | 2.23(1.63-2.74) | 13.6(10.69-15.74) | 0.77(0.61-0.89) | -3.23(-3.34 - -3.12) | 94.23(64.51-122.6) | 89.52(63.65-113.68) | 61.50(49.56-71) | 35.94(28.96-41.41) | -3.37(-3.5 - -3.23) |
| Middle SDI | 32.86(25.21-41.96) | 1.72(1.33-2.17) | 49.34(38.53-62.71) | 1.09(0.85-1.4) | 0.76(0.56 - 0.95) | 9.81(8.09-11.92) | 0.78(0.66-0.91) | 7.33(5.97-8.46) | 0.31(0.25-0.35) | -3.02(-3.06 - -2.98) | 53.62(43.32-67.56) | 32.03(26.25-39.74) | 31.79(26.51-36.93) | 13.31(11.07-15.50) | -3.23(-3.15 - -2.92) |
| High-middle SDI | 21.74(16.48-28.26) | 1.83(1.39-2.37) | 28.91(22.77-36.07) | 2.20(1.75-2.71) | 0.67(0.50 - 0.84) | 4.07(3.53-4.58) | 0.39(0.34-0.45) | 2.76(2.40-3.57) | 0.19(0.17-0.25) | -3.42(-3.51 - -3.34) | 16.86(14.89-19.03) | 14.96(13.22-16.88) | 10.93(9.27-13.25) | 7.22(6.01-8.70) | -2.67(-2.83 - -2.51) |
| High SDI | 21.41(16.53-27.62) | 2.70(2.06-3.50) | 22.92(19.01-27.61) | 3.58(2.71-4.6) | -0.15(-0.2 - -0.10) | 2.16(1.74-2.48) | 0.22(0.17-0.25) | 2.05(1.69-2.75) | 0.20(0.17-0.27) | -2.21(-2.39 - -2.03) | 7.75(6.48-9.10) | 8.93(7.45-10.65) | 6.57(5.34-8.06) | 5.69(4.45-7.14) | -1.39(-1.54 - -1.25) |
| Region |  |  |  |  |  |  |  |  |  |  |  |  |  |  |  |
| Andean Latin America | 5.45(4.59-6.48) | 12.27(10.50-14.40) | 5.59(4.57-6.97) | 8.52(6.98-10.59) | -1.87(-2.27 - -1.47) | 1.77(1.34-2.28) | 4.48(3.52-5.56) | 0.46(0.35-0.59) | 0.72(0.55-0.93) | -5.97(-6.75 - -5.19) | 13.10(9.58-17.18) | 281.99(212.30-361.84) | 2.49(1.97-3.19) | 39.49(31.30-50.62) | -6.92(-7.73 - -6.10) |
| Australasia | 0.57(0.43-0.76) | 2.77(2.1-3.7) | 0.74(0.58-0.95) | 2.73(2.03-3.55) | 0.12(0.10 - 0.13) | 0.03(0.03-0.04) | 0.15(0.11-0.18) | 0.04(0.03-0.06) | 0.15(0.11-0.19) | -2.03(-2.15 - -1.91) | 0.15(0.11-0.18) | 6.81(5.20-8.65) | 0.16(0.13-0.21) | 5.15(3.76-7.01) | -0.95(-1.04 - -0.87) |
| Caribbean | 0.84(0.65-1.09) | 2.20(1.73-2.81) | 1.32(1.03-1.70) | 3.06(2.36-3.89) | 0.68(0.60 - 0.77) | 0.49(0.32-0.71) | 1.50(1.05-2.07) | 0.42(0.32-0.55) | 0.88(0.68-1.16) | -1.44(-1.99 - -0.89) | 2.90(1.75-4.65) | 78.63(49.31-122.60) | 1.98(1.42-2.78) | 43.3(30.29-61.73) | -1.62(-2.19 - -1.04) |
| Central Asia | 1.81(1.34-2.37) | 2.42(1.83-3.12) | 2.33(1.75-3.07) | 1.36(1.1-1.68) | 0.02(-0.06 - 0.10) | 0.32(0.23-0.36) | 0.52(0.38-0.57) | 0.15(0.12-0.21) | 0.16(0.13-0.23) | -4.22(-4.58 - -3.86) | 1.96(1.36-2.25) | 27.38(19.57-31.02) | 0.97(0.79-1.25) | 10.43(8.46-13.43) | -4.01(-4.39 - -3.63) |
| Central Europe | 2.43(1.85-3.15) | 2.05(1.54-2.67) | 2.04(1.67-2.45) | 2.83(2.19-3.62) | 0.13(0.05 - 0.22) | 0.6(0.47-0.68) | 0.45(0.35-0.50) | 0.33(0.27-0.50) | 0.29(0.23-0.44) | -3.41(-3.66 - -3.15) | 1.98(1.55-2.23) | 15.32(12.05-17.34) | 0.93(0.76-1.29) | 6.56(5.28-8.66) | -2.83(-3.07 - -2.58) |
| Central Latin America | 6.03(4.51-7.72) | 3.10(2.39-3.9) | 9(6.87-11.60) | 1.66(1.29-2.16) | 0.34(0.24 - 0.44) | 1.73(1.46-2.03) | 1.37(1.13-1.59) | 2.03(1.52-2.46) | 0.81(0.61-0.98) | -1.31(-1.6 - -1.02) | 10.45(9.04-12.16) | 61.58(52.55-71.69) | 7.96(6.37-9.76) | 32.18(25.82-39.36) | -1.92(-2.29 - -1.55) |
| Central Sub-Saharan Africa | 1.26(0.97-1.60) | 2.09(1.66-2.63) | 4.17(3.15-5.38) | 2.67(2.13-3.35) | 1.06(0.97 - 1.15) | 0.56(0.2-0.91) | 1.49(0.7-2.58) | 0.73(0.40-1.20) | 0.55(0.3-0.92) | -1.48(-1.65 - -1.31) | 3.44(1.09-5.57) | 60.03(24.72-98.32) | 4.32(2.44-6.95) | 36.13(21.15-57.69) | -1.55(-1.72 - -1.39) |
| East Asia | 21.87(16.39-28.43) | 1.64(1.26-2.11) | 25.65(20.34-31.49) | 4.48(3.4-5.72) | 0.42(0.26 - 0.59) | 4.08(3.38-5.04) | 0.50(0.42-0.63) | 1.68(1.36-2.00) | 0.11(0.09-0.14) | -5.88(-6.16 - -5.6) | 17.91(14.93-21.6) | 16.34(13.66-19.54) | 6.87(5.52-8.47) | 4.37(3.50-5.41) | -4.84(-5.03 - -4.65) |
| Eastern Europe | 4.84(3.64-6.29) | 2.28(1.72-2.99) | 4.8(3.71-6.12) | 1.27(0.97-1.67) | 0.92(0.59 - 1.26) | 1.09(0.74-1.23) | 0.42(0.29-0.48) | 0.56(0.46-0.87) | 0.27(0.22-0.41) | -3.17(-3.43 - -2.91) | 4.24(2.95-4.86) | 17.83(12.55-20.56) | 2.12(1.73-3.01) | 9.11(7.31-12.41) | -2.58(-2.88 - -2.27) |
| Eastern Sub-Saharan Africa | 1.8(1.34-2.42) | 0.87(0.67-1.13) | 5.92(4.32-7.97) | 2.79(2.2-3.53) | 1.37(1.13 - 1.60) | 2.11(0.77-3.91) | 1.61(0.83-2.85) | 2.29(1.44-3.87) | 0.56(0.35-0.94) | -1.76(-1.82 - -1.70) | 12.86(3.56-24.01) | 63.87(25.98-117.37) | 12.48(7.48-19.52) | 34.19(22.03-56.27) | -2.12(-2.16 - -2.09) |
| High-income Asia Pacific | 7.67(5.71-10.02) | 4.60(3.40-6.03) | 5.86(4.63-7.30) | 1.63(1.45-1.82) | -0.12(-0.2 - -0.04) | 0.34(0.31-0.40) | 0.19(0.17-0.22) | 0.34(0.27-0.41) | 0.18(0.14-0.22) | -3.04(-3.53 - -2.55) | 2.04(1.66-2.53) | 11.98(9.68-14.84) | 1.22(0.95-1.55) | 6.94(4.94-9.53) | -1.53(-1.87 - -1.19) |
| High-income North America | 5.07(4.05-6.36) | 1.81(1.43-2.30) | 5.72(5.15-6.37) | 1.76(1.38-2.2) | -0.40(-0.51 - -0.30) | 0.51(0.41-0.67) | 0.15(0.12-0.2) | 0.72(0.56-0.93) | 0.2(0.15-0.25) | -0.69(-0.93 - -0.44) | 1.93(1.59-2.36) | 6.50(5.31-7.92) | 2.27(1.84-2.74) | 5.23(4.26-6.29) | -0.73(-0.85 - -0.60) |
| North Africa and Middle East | 6.94(5.15-9.13) | 1.84(1.4-2.37) | 17.46(13.29-22.72) | 2.84(2.2-3.69) | 1.29(1.22 - 1.36) | 1.43(0.81-1.94) | 0.69(0.45-0.97) | 1.35(0.92-1.73) | 0.22(0.15-0.28) | -2.73(-2.77 - -2.70) | 7.51(3.9-11.29) | 24.05(14.24-32.12) | 7.09(5.16-8.79) | 12.43(9.18-15.35) | -2.32(-2.36 - -2.28) |
| Oceania | 0.06(0.05-0.08) | 0.94(0.73-1.2) | 0.15(0.12-0.20) | 2.84(2.25-3.62) | 0.38(0.27 - 0.49) | 0.02(0.01-0.03) | 0.51(0.26-0.75) | 0.03(0.02-0.05) | 0.25(0.13-0.36) | -0.76(-0.86 - -0.65) | 0.10(0.04-0.15) | 18.40(8.96-27.63) | 0.17(0.09-0.24) | 14.33(8.15-20.09) | -0.66(-0.76 - -0.56) |
| South Asia | 20.67(16.6-26.06) | 1.76(1.44-2.16) | 52.14(41.14-66.21) | 1.83(1.39-2.41) | 1.27(1.06 - 1.48) | 20.45(13.97-27.00) | 2.76(1.95-3.49) | 15.83(12.26-18.79) | 0.88(0.68-1.04) | -3.63(-3.83 - -3.43) | 102.43(68.97-138.82) | 105.12(72.29-138.63) | 70.38(55.74-83.69) | 39.77(31.54-47.15) | -3.7(-3.88 - -3.52) |
| Southeast Asia | 6.4(4.86-8.40) | 1.24(0.96-1.60) | 11.47(8.92-14.77) | 1.20(0.9-1.59) | 0.94(0.88 - 1.00) | 3.05(1.98-4.36) | 0.83(0.60-1.08) | 2.15(1.63-2.53) | 0.32(0.24-0.38) | -2.92(-3.01 - -2.82) | 17.91(10.28-27.76) | 37.4(23.53-54.82) | 10.29(7.56-12.18) | 15.69(11.73-18.65) | -3.08(-3.19 - -2.97) |
| Southern Latin America | 1.03(0.78-1.34) | 2.04(1.57-2.64) | 2.02(1.58-2.55) | 2.60(2.11-3.21) | 1.45(1.32 - 1.57) | 0.27(0.19-0.31) | 0.60(0.43-0.68) | 0.22(0.18-0.31) | 0.32(0.27-0.47) | -2.51(-2.98 - -2.04) | 1.01(0.75-1.15) | 20.89(15.48-23.82) | 0.81(0.67-1.07) | 11.32(9.22-14.76) | -1.85(-2.24 - -1.46) |
| Southern Sub-Saharan Africa | 0.89(0.65-1.19) | 1.45(1.10-1.92) | 1.55(1.17-2.04) | 2.82(2.26-3.52) | 0.64(0.07 - 1.21) | 0.21(0.16-0.29) | 0.50(0.39-0.69) | 0.26(0.22-0.32) | 0.33(0.28-0.41) | -0.73(-1.26 - -0.18) | 1.29(0.96-1.7) | 24.52(18.74-32.12) | 1.41(1.17-1.73) | 18.08(15.12-22.14) | -0.89(-1.40 - -0.38) |
| Tropical Latin America | 1.8(1.38-2.29) | 1.06(0.83-1.33) | 2.98(2.43-3.67) | 1.63(1.29-2.06) | 0.85(0.80 - 0.91) | 0.8(0.66-1.09) | 0.70(0.57-0.96) | 1.34(0.99-1.61) | 0.6(0.44-0.72) | -0.19(-0.35 - -0.03) | 4.07(3.37-5.31) | 28.15(23.35-37.52) | 4.95(3.81-5.82) | 21.48(16.54-25.26) | -0.43(-0.61 - -0.26) |
| Western Europe | 9.09(7.02-11.87) | 2.49(1.91-3.27) | 9.97(8.07-12.30) | 2.08(1.59-2.65) | 0.36(0.29 - 0.43) | 1.31(0.98-1.47) | 0.23(0.17-0.26) | 1.05(0.86-1.57) | 0.24(0.20-0.36) | -2.58(-2.78 - -2.38) | 3.71(2.85-4.38) | 8.32(6.51-10.02) | 2.85(2.31-3.72) | 5.50(4.17-7.14) | -1.33(-1.44 - -1.22) |
| Western Sub-Saharan Africa | 1.69(1.26-2.21) | 0.81(0.63-1.06) | 6.11(4.45-8.24) | 2.18(1.68-2.77) | 1.42(1.14 - 1.69) | 1.60(0.87-2.51) | 1.08(0.67-1.70) | 1.45(0.98-1.97) | 0.32(0.21-0.43) | -2.62(-2.82 - -2.42) | 9.62(4.29-15.4) | 44.70(25.04-68.81) | 8.42(5.36-12.06) | 19.32(13.53-25.96) | -3.20(-3.43 - -2.97) |
| APED: Appendicitis; ASR, age- standardised incidence rate; EAPC, estimated annual percentage change; UI, uncertainty interval. | | | | | | | | | | | | | | | |
