# Supplementary material for: Global, regional, and national burden of digestive diseases: findings from the global burden of disease study 2019
Source: Front Public Health. 2023 Aug 24;11:1202980. doi: 10.3389/fpubh.2023.1202980 (PMC10483149; doi:10.3389/fpubh.2023.1202980)
Supplement: Supplementary file 4 [file Table_4.docx]

| Table S4. The Incidence, Death, and DALYs of PIIO in 1990 and 2019 | | | | | | | | | | | | | | | |
| --- | --- | --- | --- | --- | --- | --- | --- | --- | --- | --- | --- | --- | --- | --- | --- |
| Characteristics | 1990 | | 2019 | | 1990-2019 | 1990 | | 2019 | | 1990-2019 | 1990 | | 2019 | | 1990-2019 |
|  | Incidence cases  No×10^5^ (95%UI) | ASR per 100 000  No (95% UI) | Incidence cases  No×10^5^ (95%UI) | ASR per 100 000  No (95% UI) | EAPC  No (95% CI) | Death cases  No×10^3^ (95%UI) | ASR per 100 000  No (95% UI) | Death cases  No×10^3^ (95%UI) | ASR per 100 000  No (95% UI) | EAPC  No (95% CI) | DALYs  No×10^5^ (95%UI) | ASR per 100 000  No (95% UI) | DALYs  No×10^5^ (95%UI) | ASR per 100 000  No (95% UI) | EAPC  No (95% CI) |
| Global | 54.12(52.18-56.21) | 119.48(115.15-123.99) | 101.03(97.5-104.44) | 127.24(122.92-131.33) | 0.22(0.19 - 0.25) | 155.39(135.91-170.15) | 3.85(3.42-4.23) | 238.74(199.01-265.8) | 3.14(2.62-3.51) | -0.72(-0.76 - -0.68) | 67.03(58.17-76.14) | 128.77(111.91-143.92) | 70.84(56.48-82.54) | 93.46(74.42-109.76) | -1.14(-1.17 - -1.11) |
| Sex |  |  |  |  |  |  |  |  |  |  |  |  |  |  |  |
| Female | 27.24(26.27-28.30) | 116.37(112.12-120.73) | 49.56(47.78-51.2) | 120.28(116.15-124.18) | 0.10(0.06 - 0.15) | 71.81(62.61-81.89) | 3.30(2.86-3.78) | 114.09(90.82-129.53) | 2.71(2.16-3.08) | -0.68(-0.74 - -0.61) | 29.10(25.15-33.6) | 109.78(95.49-126.17) | 30.55(23.53-35.7) | 78.78(60.78-92.89) | -1.16(-1.18 - -1.13) |
| Male | 26.88(25.88-27.92) | 123.40(118.92-127.95) | 51.47(49.71-53.25) | 135.26(130.83-139.59) | 0.34(0.31 - 0.37) | 83.58(66.98-94.27) | 4.49(3.74-5.16) | 124.65(99.17-142.42) | 3.65(2.93-4.15) | -0.75(-0.78 - -0.72) | 37.93(30.17-44.58) | 148.74(119.71-170.49) | 40.29(31.32-48.13) | 108.76(85.43-130.57) | -1.13(-1.18 - -1.09) |
| SDI |  |  |  |  |  |  |  |  |  |  |  |  |  |  |  |
| Low SDI | 3.22(3.1-3.36) | 84.37(81.26-87.47) | 7.64(7.36-7.92) | 94.47(91.08-97.69) | 0.65(0.58 - 0.72) | 24.34(17.77-29.14) | 8.08(5.65-9.82) | 42.45(31.06-51.49) | 6.93(5.08-8.19) | -0.61(-0.67 - -0.55) | 12.27(9.49-14.79) | 253.37(180.15-304.58) | 19.48(14.17-24.95) | 208.00(151.82-252.74) | -0.74(-0.78 - -0.70) |
| Low-middle SDI | 7.88(7.58-8.18) | 90.62(87.29-93.91) | 16.42(15.84-17.01) | 105.54(101.78-109.18) | 0.57(0.53 - 0.61) | 45.77(35.39-52) | 6.52(5.02-7.39) | 62.63(48.07-73.15) | 4.69(3.66-5.45) | -1.25(-1.33 - -1.18) | 22.07(17.98-25.75) | 212.44(164.79-242.25) | 22.46(16.74-26.74) | 142.34(107.09-168.83) | -1.48(-1.53 - -1.43) |
| Middle SDI | 12.86(12.38-13.4) | 92.03(88.47-95.69) | 25.21(24.34-26.06) | 104.56(101.22-107.97) | 0.66(0.59 - 0.73) | 40.23(35.75-48.39) | 3.71(3.28-4.72) | 52.09(45.64-61.59) | 2.52(2.2-2.98) | -1.30(-1.33 - -1.26) | 20.73(17.72-24.1) | 127.81(112.02-148.88) | 15.35(12.9-17.9) | 69.66(58.63-81.39) | -2.06(-2.14 - -1.99) |
| High-middle SDI | 12.06(11.59-12.53) | 110.72(106.62-115.07) | 21.14(20.37-21.87) | 123.14(119.20-127.31) | 0.41(0.33 - 0.49) | 23.11(21.26-26.75) | 2.53(2.30-2.93) | 34.55(28.76-37.91) | 1.87(1.56-2.05) | -1.03(-1.11 - -0.96) | 7.93(7.2-8.88) | 76.77(69.79-86.45) | 6.89(5.93-7.53) | 42.76(37.07-47.40) | -2.16(-2.22 - -2.09) |
| High SDI | 18.09(17.4-18.8) | 194.23(187.15-201.46) | 28.81(27.75-29.93) | 196.90(190.16-203.54) | 0.03(0.01 - 0.04) | 21.86(18.96-26.22) | 2.17(1.87-2.58) | 46.88(35.7-52.77) | 2.14(1.65-2.38) | 0.02(-0.10 - 0.13) | 4(3.62-4.72) | 43.09(39.3-50.17) | 6.62(5.36-7.25) | 40.31(33.94-43.75) | -0.11(-0.26 - 0.03) |
| Region |  |  |  |  |  |  |  |  |  |  |  |  |  |  |  |
| Andean Latin America | 0.40(0.38-0.41) | 125.41(121.06-130.68) | 0.83(0.80-0.86) | 138.95(134.33-144.14) | 0.43(0.38 - 0.48) | 3.22(2.52-3.93) | 9.47(7.72-11.05) | 2.50(1.85-3.21) | 4.52(3.33-5.78) | -2.91(-3.09 - -2.73) | 2.14(1.57-2.72) | 454.16(346.4-564.94) | 0.72(0.54-0.95) | 121.78(90.52-159.11) | -5.07(-5.49 - -4.66) |
| Australasia | 0.39(0.37-0.41) | 177.80(170.84-184.75) | 0.79(0.76-0.83) | 194.12(186.65-202.01) | 0.31(0.25 - 0.37) | 0.38(0.32-0.47) | 1.81(1.53-2.23) | 1.10(0.78-1.29) | 1.92(1.37-2.25) | 0.42(0.34 - 0.50) | 0.07(0.06-0.08) | 30.63(27.02-36.45) | 0.14(0.11-0.16) | 29.82(23.12-33.58) | 0.14(0.06 - 0.23) |
| Caribbean | 0.30(0.29-0.32) | 96.57(93.4-100.31) | 0.55(0.53-0.57) | 112.35(108.61-116.37) | 0.56(0.49 - 0.62) | 1.05(0.87-1.34) | 3.65(3.10-4.38) | 1.51(1.25-1.83) | 3.05(2.49-3.77) | -0.59(-0.66 - -0.51) | 0.49(0.34-0.74) | 134.34(100.31-193.47) | 0.47(0.34-0.66) | 103.08(73.43-150.36) | -0.83(-1.00 - -0.66) |
| Central Asia | 0.43(0.41-0.45) | 70.47(67.74-73.34) | 0.69(0.66-0.72) | 79.55(76.69-82.55) | 0.42(0.34 - 0.50) | 1.32(1.17-1.45) | 2.04(1.82-2.24) | 0.97(0.78-1.14) | 1.41(1.09-1.63) | -1.11(-1.17 - -1.06) | 0.84(0.75-0.94) | 103.77(92.36-114.88) | 0.45(0.37-0.54) | 52.17(42.64-62.60) | -2.10(-2.20 - -2.00) |
| Central Europe | 1.38(1.33-1.43) | 103.31(99.56-107.08) | 1.69(1.63-1.76) | 102.24(98.60-105.82) | -0.09(-0.2 - 0.03) | 3.07(2.77-3.62) | 2.45(2.20-2.84) | 4.29(3.5-5.00) | 2.01(1.66-2.35) | -0.55(-0.63 - -0.48) | 0.82(0.74-0.92) | 69.46(62.33-77.21) | 0.77(0.64-0.9) | 44.39(36.99-51.85) | -1.52(-1.56 - -1.48) |
| Central Latin America | 1.79(1.72-1.86) | 125.53(121.26-130.04) | 3.25(3.15-3.35) | 136.54(132.46-140.74) | 0.32(0.27 - 0.37) | 4.17(3.7-4.65) | 3.71(3.31-4.41) | 7.28(5.67-8.62) | 3.23(2.52-3.83) | -0.18(-0.34 - -0.03) | 2.36(1.99-2.67) | 134.28(117.97-148.16) | 2.11(1.64-2.58) | 91.75(71.40-113.00) | -1.05(-1.23 - -0.87) |
| Central Sub-Saharan Africa | 0.50(0.48-0.53) | 120.48(115.54-125.54) | 1.35(1.29-1.41) | 140.74(134.86-146.15) | 0.53(0.45 - 0.61) | 2.06(1.56-2.73) | 6.81(5.19-8.96) | 3.82(2.46-5.87) | 5.71(3.76-8.49) | -0.62(-0.71 - -0.53) | 1.13(0.82-1.61) | 213.76(164.66-278.90) | 1.84(1.11-3.04) | 178.39(114.84-269.8) | -0.56(-0.68 - -0.44) |
| East Asia | 10.21(9.79-10.69) | 97.68(93.58-102.01) | 17.6(16.95-18.27) | 100.68(97.46-104.00) | 0.15(0.03 - 0.28) | 22.44(19.67-26.63) | 2.71(2.39-3.39) | 16.67(13.79-19.86) | 1.10(0.90-1.27) | -3.12(-3.21 - -3.04) | 12.59(10.60-14.79) | 113.52(96.68-132.66) | 4.16(3.56-4.83) | 32.16(27.31-37.02) | -4.44(-4.59 - -4.29) |
| Eastern Europe | 2.50(2.40-2.61) | 101.82(97.75-105.69) | 2.84(2.74-2.95) | 108.19(104.46-112.15) | 0.32(0.27 - 0.36) | 3.50(3.13-4.25) | 1.44(1.29-1.75) | 4.23(3.43-5.07) | 1.32(1.07-1.57) | -0.65(-0.79 - -0.50) | 1.08(0.92-1.23) | 47.01(40.30-52.78) | 0.99(0.83-1.2) | 36.81(31.16-44.47) | -1.36(-1.56 - -1.17) |
| Eastern Sub-Saharan Africa | 1.14(1.09-1.19) | 85.7(82.64-88.82) | 3.11(3.00-3.23) | 109.53(105.67-113.47) | 0.93(0.87 - 0.98) | 7.55(5.6-9.57) | 7.29(5.22-9.36) | 14.71(11.62-18.43) | 7.19(5.63-8.89) | 0(-0.05 - 0.05) | 4.02(2.94-5.27) | 228.9(170.49-291.92) | 6.88(5.16-8.85) | 216.19(170.22-271.15) | -0.09(-0.15 - -0.04) |
| High-income Asia Pacific | 3.77(3.6-3.95) | 202.78(194.54-211.93) | 6.37(6.12-6.63) | 216.40(208.75-224.53) | 0.24(0.21 - 0.27) | 3.69(2.99-4.06) | 2.18(1.76-2.38) | 11.73(8.03-14.04) | 2.00(1.43-2.33) | -0.31(-0.37 - -0.25) | 0.75(0.65-0.84) | 43.83(38.51-48.96) | 1.48(1.11-1.68) | 35.75(28.71-39.43) | -0.72(-0.76 - -0.69) |
| High-income North America | 8.12(7.77-8.45) | 255.12(244.6-265.15) | 12.41(11.94-12.90) | 246.10(237.85-254.54) | -0.17(-0.21 - -0.13) | 6.60(5.68-8.35) | 1.85(1.60-2.33) | 13.53(10.45-15.10) | 2.03(1.6-2.25) | 0.45(0.29 - 0.61) | 1.32(1.19-1.60) | 41.99(38.19-50.32) | 2.23(1.85-2.45) | 45.38(39.12-49.44) | 0.52(0.28 - 0.77) |
| North Africa and Middle East | 2.18(2.09-2.29) | 76.31(73.44-79.42) | 5.70(5.49-5.92) | 106.89(103.28-110.66) | 1.30(1.25 - 1.35) | 5.22(3.95-7.93) | 2.60(1.93-4.36) | 7.34(5.95-9.45) | 1.96(1.57-2.61) | -0.74(-0.87 - -0.60) | 2.79(1.97-3.87) | 77.48(58.72-114.44) | 2.56(2.06-3.28) | 51.89(42.18-65.65) | -1.16(-1.26 - -1.06) |
| Oceania | 0.03(0.03-0.03) | 52.10(49.86-54.3) | 0.06(0.06-0.06) | 53.98(51.92-56.16) | -0.02(-0.08 - 0.04) | 0.07(0.04-0.09) | 2.18(1.37-2.82) | 0.14(0.08-0.19) | 1.95(1.23-2.55) | -0.28(-0.36 - -0.20) | 0.04(0.02-0.06) | 59.85(37.71-81.15) | 0.07(0.04-0.11) | 55.74(34.08-79.60) | -0.10(-0.17 - -0.03) |
| South Asia | 7.23(6.95-7.51) | 88.97(85.57-92.28) | 17.35(16.72-17.97) | 108.99(105.06-112.79) | 0.72(0.70 - 0.74) | 44.96(31.71-53.28) | 7.38(5.09-8.66) | 64.90(46.18-78.46) | 4.78(3.46-5.74) | -1.75(-1.92 - -1.59) | 19.38(14.34-23.49) | 219.24(155.1-259.54) | 22.48(15.73-26.89) | 139.46(98.45-166.53) | -1.05(-1.23 - -0.87) |
| Southeast Asia | 3.07(2.96-3.19) | 86.53(83.64-89.59) | 7.57(7.29-7.87) | 120.18(116.18-124.4) | 1.26(1.21 - 1.31) | 15.71(12.39-20.56) | 5.68(4.68-7.58) | 23.25(20.14-28.61) | 4.59(3.93-5.79) | -0.79(-0.83 - -0.76) | 7.61(5.23-10.75) | 178.17(135-234.45) | 6.95(5.72-8.1) | 119.74(101.8-139.59) | -1.40(-1.43 - -1.37) |
| Southern Latin America | 0.40(0.38-0.41) | 85.26(82.07-88.39) | 0.88(0.85-0.91) | 115.69(111.86-119.69) | 0.95(0.86 - 1.04) | 2.01(1.75-2.39) | 4.95(4.31-5.9) | 3.97(3.13-4.5) | 4.66(3.67-5.27) | 0.03(-0.07 - 0.13) | 0.41(0.36-0.49) | 89.87(79.4-107.16) | 0.65(0.53-0.73) | 82.11(67.07-91.37) | -0.07(-0.17 - 0.03) |
| Southern Sub-Saharan Africa | 0.53(0.51-0.55) | 132.70(127.77-137.77) | 0.93(0.9-0.97) | 136.42(131.52-141.27) | 0.02(-0.17 - 0.20) | 1.72(1.29-2.35) | 6.00(4.49-8.24) | 3.29(2.81-3.86) | 6.16(5.3-7.15) | 0.06(-0.08 - 0.21) | 0.62(0.48-0.82) | 160.44(121.29-218.82) | 1.05(0.88-1.25) | 158.83(133.68-189.31) | -0.04(-0.17 - 0.09) |
| Tropical Latin America | 1.39(1.34-1.46) | 111.16(107.03-115.6) | 2.18(2.11-2.27) | 96.85(93.62-100.30) | -0.76(-0.93 - -0.58) | 3.43(2.92-3.88) | 3.67(3.15-4.27) | 7.28(5.69-8.07) | 3.26(2.55-3.61) | -0.15(-0.23 - -0.07) | 1.53(1.28-1.79) | 115.38(97.82-132.24) | 1.81(1.51-2.02) | 84.95(71.85-95.15) | -0.81(-0.90 - -0.72) |
| Western Europe | 6.87(6.59-7.19) | 140.26(135-145.71) | 10.80(10.36-11.27) | 153.50(147.76-159.05) | 0.32(0.29 - 0.34) | 13.65(11.77-16.57) | 2.39(2.05-2.88) | 26.58(21-30.18) | 2.36(1.88-2.69) | 0.05(-0.07 - 0.16) | 2.12(1.89-2.55) | 40.85(36.46-48.41) | 3.37(2.76-3.82) | 37.74(31.5-42.44) | -0.18(-0.28 - -0.07) |
| Western Sub-Saharan Africa | 1.51(1.45-1.57) | 110.62(106.49-114.69) | 4.07(3.92-4.22) | 133.97(128.91-138.77) | 0.71(0.66 - 0.76) | 9.57(6.97-12.69) | 8.31(5.68-11.46) | 19.66(12.76-28.41) | 8.39(5.55-11.9) | 0.14(0.08 - 0.20) | 4.94(3.72-6.39) | 254.76(181.32-344.51) | 9.66(6.28-14.40) | 246.62(160.34-355.33) | -0.04(-0.09 - 0.02) |
| PIIO: Paralytic ileus and intestinal obstruction; ASR, age- standardised incidence rate; EAPC, estimated annual percentage change; UI, uncertainty interval. | | | | | | | | | | | | | | | |
