# Supplementary material for: Global, regional, and national burden of digestive diseases: findings from the global burden of disease study 2019
Source: Front Public Health. 2023 Aug 24;11:1202980. doi: 10.3389/fpubh.2023.1202980 (PMC10483149; doi:10.3389/fpubh.2023.1202980)
Supplement: Supplementary file 5 [file Table_5.docx]

| Table S5. The Incidence, Death, and DALYs of IFAH in 1990 and 2019 | | | | | | | | | | | | | | | |
| --- | --- | --- | --- | --- | --- | --- | --- | --- | --- | --- | --- | --- | --- | --- | --- |
| Characteristics | 1990 | | 2019 | | 1990-2019 | 1990 | | 2019 | | 1990-2019 | 1990 | | 2019 | | 1990-2019 |
|  | Incidence cases  No×10^4^ (95%UI) | ASR per 100 000  No (95% UI) | Incidence cases  No×10^4^ (95%UI) | ASR per 100 000  No (95% UI) | EAPC  No (95% CI) | Death cases  No×10^2^ (95%UI) | ASR per 100 000  No (95% UI) | Death cases  No×10^2^ (95%UI) | ASR per 100 000  No (95% UI) | EAPC  No (95% CI) | DALYs  No×10^4^ (95%UI) | ASR per 100 000  No (95% UI) | DALYs  No×10^4^ (95%UI) | ASR per 100 000  No (95% UI) | EAPC  No (95% CI) |
| Global | 795.56(661.43-940.99) | 163.03(134.63-192.62) | 1302.07(1068.21-1302.07) | 162.96(134.12-192.86) | -0.08(-0.12 - -0.04) | 401.41(328.04-454.18) | 1.06(0.87-1.21) | 480.77(416.54-540.21) | 0.63(0.55-0.71) | -1.86(-1.99 - -1.73) | 306.99(235.59-389.79) | 62.06(48.51-77.45) | 335.40(261.06-424.71) | 42.75(33.36-53.99) | -1.41(-1.50 - -1.33) |
| Sex |  |  |  |  |  |  |  |  |  |  |  |  |  |  |  |
| Female | 104.69(86.69-124.17) | 41.51(34.23-49.10) | 178.51(146.29-178.51) | 45.69(37.67-54.48) | 0.52(0.45 - 0.59) | 146.96(102.02-172.74) | 0.72(0.52-0.83) | 191.26(146.40-226.72) | 0.44(0.34-0.52) | -1.75(-1.88 - -1.62) | 78.15(57.22-101.24) | 31.20(23.52-39.33) | 85.26(65.80-106.79) | 21.46(16.52-27.16) | -1.37(-1.48 - -1.25) |
| Male | 690.86(573.35-816.89) | 288.85(238.86-340.29) | 1123.56(922.11-1123.56) | 283.34(232.65-335.09) | -0.18(-0.22 - -0.13) | 254.45(196.94-308.91) | 1.51(1.25-1.80) | 289.51(253.98-330.02) | 0.87(0.77-0.99) | -1.99(-2.12 - -1.86) | 228.85(169.57-297.25) | 94.54(72.44-120.46) | 250.15(192.11-320.33) | 64.79(50.33-82.25) | -1.45(-1.52 - -1.37) |
| SDI |  |  |  |  |  |  |  |  |  |  |  |  |  |  |  |
| Low SDI | 68.81(57.43-81.12) | 161.53(135.09-188.55) | 130.83(108.79-130.83) | 145.34(120.87-171.45) | -0.26(-0.34 - -0.19) | 62.55(41.67-77.2) | 2.37(1.51-3.52) | 84.02(60.51-118.32) | 1.71(1.20-2.50) | -1.37(-1.49 - -1.24) | 48.02(33.49-61.58) | 106.90(81.49-130.41) | 65.59(49.95-83.24) | 77.39(58.96-99.70) | -1.29(-1.36 - -1.21) |
| Low-middle SDI | 170.49(141.00-202.60) | 180.97(149.92-214.61) | 269.77(222.50-269.77) | 163.29(134.35-195.68) | -0.58(-0.68 - -0.49) | 100.23(75.77-119.35) | 1.70(1.21-2.20) | 116.15(94.15-136.91) | 0.99(0.80-1.17) | -2.20(-2.37 - -2.03) | 83.36(63.16-106.00) | 88.47(68.88-108.73) | 87.51(67.97-112.20) | 55.59(43.89-70.30) | -1.87(-2.00 - -1.75) |
| Middle SDI | 217.89(182.24-257.70) | 145.77(120.57-172.14) | 376.17(308.48-376.17) | 150.56(124.43-177.66) | 0.23(0.16 - 0.30) | 95.96(73.28-117.95) | 0.99(0.78-1.19) | 126.98(107.3-146.46) | 0.61(0.52-0.71) | -1.63(-1.75 - -1.51) | 88.87(64.91-118.90) | 59.24(45.42-74.75) | 94.54(72.91-120.75) | 39.24(30.39-50.10) | -1.52(-1.63 - -1.42) |
| High-middle SDI | 198.67(162.94-237.62) | 176.79(144.44-211.19) | 296.1(237.61-296.1) | 181.06(147.84-216.2) | 0.13(0.07 - 0.18) | 75.10(67.98-82.86) | 0.83(0.75-0.91) | 76.74(67.86-97.03) | 0.41(0.36-0.51) | -2.61(-2.73 - -2.49) | 54.09(41.92-68.60) | 50.03(39.22-62.85) | 53.31(39.70-69.12) | 32.63(24.16-42.57) | -1.63(-1.70 - -1.55) |
| High SDI | 139.27(113.34-165.01) | 157.90(128.61-187.29) | 182.24(145.76-182.24) | 147.66(121.77-174.46) | -0.08(-0.16 - 0.01) | 67.25(56.39-71.28) | 0.66(0.55-0.70) | 76.53(63.91-88.54) | 0.35(0.30-0.40) | -1.99(-2.29 - -1.69) | 32.40(25.22-40.96) | 35.98(27.63-46.11) | 34.18(26.11-43.28) | 25.92(19.32-33.64) | -1.03(-1.12 - -0.93) |
| Region |  |  |  |  |  |  |  |  |  |  |  |  |  |  |  |
| Andean Latin America | 9.61(8.48-10.85) | 252.85(223.50-283.81) | 18.04(15.35-18.04) | 289.76(247.36-333.1) | 0.32(0.22 - 0.41) | 7.00(4.14-10.59) | 1.93(1.25-2.73) | 3.93(2.96-5.20) | 0.70(0.53-0.93) | -3.52(-3.95 - -3.09) | 8.00(5.45-11.17) | 182.21(130.05-243.45) | 5.22(3.88-7.01) | 84.55(63.15-113.28) | -2.80(-3.16 - -2.44) |
| Australasia | 2.74(2.25-3.23) | 125.56(103.15-147.54) | 4.63(3.83-4.63) | 122.51(101.55-144.69) | -0.10(-0.13 - -0.07) | 1.14(1.02-1.29) | 0.53(0.46-0.60) | 2.37(1.93-2.76) | 0.42(0.35-0.48) | -0.40(-0.58 - -0.22) | 0.63(0.49-0.81) | 29.05(22.42-37.13) | 0.96(0.73-1.23) | 23.57(17.57-30.70) | -0.61(-0.70 - -0.52) |
| Caribbean | 7.63(6.48-8.88) | 226.92(193.76-262.17) | 12.72(10.72-12.72) | 265.44(224.40-308.21) | 0.56(0.52 - 0.60) | 4.68(2.66-7.22) | 1.50(0.95-2.10) | 4.73(3.28-6.38) | 0.97(0.65-1.35) | -1.38(-1.66 - -1.09) | 4.52(2.79-6.80) | 124.80(80.76-181.05) | 4.43(3.15-6.02) | 94.88(66.51-130.08) | -0.84(-1.01 - -0.67) |
| Central Asia | 8.90(7.52-10.42) | 134.50(115.19-156.43) | 15.12(12.57-15.12) | 160.86(134.58-190.08) | 0.91(0.66 - 1.16) | 3.75(3.32-4.26) | 0.69(0.61-0.78) | 2.51(2.22-2.97) | 0.41(0.36-0.48) | -2.14(-2.32 - -1.97) | 3.92(3.14-4.85) | 57.46(46.02-71.85) | 3.95(2.86-5.36) | 43.93(32.23-59.04) | -0.98(-1.01 - -0.95) |
| Central Europe | 25.43(21.75-29.29) | 190.20(163.30-218.36) | 24.12(20.34-24.12) | 184.6(157.70-212.85) | -0.13(-0.23 - -0.02) | 20.34(15.41-21.54) | 1.60(1.21-1.72) | 10.28(8.70-14.28) | 0.47(0.40-0.65) | -4.65(-5.06 - -4.24) | 9.08(7.48-10.82) | 70.57(57.94-84.31) | 5.26(4.10-6.72) | 35.97(26.94-46.71) | -0.95(-2.64 - -2.24) |
| Central Latin America | 42.27(36.02-49.48) | 325.30(274.59-378.10) | 72.45(60.79-72.45) | 293.46(246.81-342.28) | -0.26(-0.39 - -0.14) | 16.11(14.37-18.17) | 1.90(1.64-2.03) | 27.83(23.49-32.42) | 1.24(1.04-1.44) | -1.28(-1.59 - -0.96) | 14.55(11.30-18.41) | 111.87(89.09-138.65) | 18.59(14.41-23.72) | 76.65(59.65-97.46) | -1.19(-1.44 - -0.94) |
| Central Sub-Saharan Africa | 5.80(4.75-7.01) | 117.77(101.13-135.03) | 18.17(14.74-18.17) | 171.59(143.19-201.92) | 1.26(1.23 - 1.30) | 6.88(3.06-10.17) | 2.16(1.13-3.90) | 9.02(5.30-16.65) | 1.65(0.96-3.12) | -0.84(-0.92 - -0.77) | 5.96(3.42-8.77) | 100.54(65.26-131.46) | 8.93(6.25-13.24) | 87.27(62.83-127.05) | -0.46(-0.53 - -0.39) |
| East Asia | 104.76(83.84-126.68) | 95.37(75.67-115.51) | 199.50(154.43-199.50) | 124.00(99.83-148.28) | 1.00(0.94 - 1.05) | 29.87(15.20-45.76) | 0.37(0.22-0.50) | 17.86(14.30-22.64) | 0.12(0.09-0.15) | -4.24(-4.4 - -4.07) | 38.28(24.82-54.71) | 34.51(22.91-48.36) | 30.05(20.75-40.73) | 20.09(13.97-27.99) | -2.02(-2.09 - -1.94) |
| Eastern Europe | 66.34(53.33-81.22) | 277.08(223.87-335.03) | 68.10(53.71-68.10) | 291.69(234.26-353.46) | 0.27(0.20 - 0.34) | 16.30(14.95-20.30) | 0.67(0.61-0.83) | 16.50(14.24-22.6) | 0.49(0.42-0.66) | -1.58(-1.78 - -1.37) | 14.04(10.38-18.25) | 59.48(44.18-77.13) | 12.13(8.94-15.99) | 49.28(35.41-66.30) | -0.82(-0.89 - -0.75) |
| Eastern Sub-Saharan Africa | 14.25(11.64-17.19) | 92.72(77.30-107.86) | 37.27(31.09-37.27) | 125.88(105.06-146.75) | 1.08(1.00 - 1.15) | 20.93(10.27-27.71) | 2.18(1.35-3.95) | 29.93(19.70-56.23) | 1.91(1.22-3.82) | -0.40(-0.44 - -0.36) | 15.42(8.59-21.26) | 85.28(56.56-111.25) | 20.49(15.07-29.00) | 71.99(52.21-111.60) | -0.52(-0.57 - -0.47) |
| High-income Asia Pacific | 43.90(34.96-52.87) | 258.24(210.12-307.73) | 57.76(45.43-57.76) | 276.01(226.91-323.91) | 0.20(0.16 - 0.24) | 7.01(5.68-7.54) | 0.42(0.33-0.45) | 10.78(7.86-13.76) | 0.17(0.13-0.21) | -2.61(-3.19 - -2.03) | 8.16(5.78-11.15) | 48.80(34.67-67.50) | 8.37(5.87-11.26) | 41.55(28.35-58.44) | -0.41(-0.53 - -0.29) |
| High-income North America | 36.99(28.94-44.88) | 120.55(94.77-146.04) | 45.02(35.84-45.02) | 100.76(82.56-119.66) | -0.73(-0.93 - -0.53) | 15.60(13.91-16.54) | 0.44(0.39-0.46) | 24.19(20.58-26.74) | 0.37(0.32-0.40) | -0.48(-0.58 - -0.38) | 7.63(5.99-9.61) | 24.42(18.94-30.93) | 9.22(7.40-11.35) | 19.50(15.42-24.22) | -0.79(-0.87 - -0.71) |
| North Africa and Middle East | 18.80(15.10-23.10) | 56.19(45.92-67.17) | 44.00(35.77-44.00) | 76.66(62.83-91.26) | 1.14(1.11 - 1.17) | 6.33(3.11-10.76) | 0.35(0.19-0.55) | 8.53(6.64-12.1) | 0.25(0.19-0.36) | -0.75(-0.96 - -0.53) | 7.80(4.99-11.30) | 23.48(16.11-32.12) | 11.15(8.01-15.05) | 20.44(14.93-27.10) | -0.35(-0.43 - -0.27) |
| Oceania | 0.35(0.28-0.43) | 64.88(54.48-75.09) | 0.98(0.81-0.98) | 83.49(70.51-97.24) | 0.74(0.65 - 0.82) | 0.26(0.14-0.40) | 0.82(0.45-1.32) | 0.47(0.29-0.78) | 0.69(0.44-1.12) | -0.36(-0.55 - -0.17) | 0.22(0.15-0.32) | 40.15(28.03-54.44) | 0.45(0.31-0.66) | 39.32(28.65-53.20) | 0.06(-0.04 - 0.16) |
| South Asia | 203.84(165.43-244.00) | 228.09(185.89-273.58) | 309.58(248.49-309.58) | 181.50(146.25-220.94) | -1.19(-1.34 - -1.04) | 109.12(73.38-129.23) | 2.33(1.48-3.02) | 127.47(98.50-163.31) | 1.11(0.86-1.42) | -3.12(-3.37 - -2.86) | 85.06(66.02-105.29) | 104.50(81.90-127.52) | 91.96(70.09-119.11) | 57.35(44.37-73.25) | -2.53(-2.71 - -2.34) |
| Southeast Asia | 53.87(44.73-63.24) | 149.30(123.37-175.76) | 106.39(88.17-106.39) | 157.03(130.70-184.06) | -0.11(-0.23 - 0.01) | 49.71(32.07-68.49) | 1.87(1.30-2.42) | 68.46(51.62-81.09) | 1.34(1.03-1.58) | -1.05(-1.13 - -0.98) | 34.52(21.76-57.07) | 87.35(62.22-120.75) | 34.81(27.99-42.16) | 56.13(45.68-67.29) | -1.60(-1.67 - -1.53) |
| Southern Latin America | 5.89(5.02-6.78) | 121.59(103.76-139.93) | 11.60(9.77-11.60) | 162.33(137.28-188.53) | 0.87(0.74 - 1.00) | 3.92(3.50-4.23) | 0.91(0.81-0.98) | 5.57(4.83-6.27) | 0.66(0.57-0.74) | -0.89(-1.24 - -0.53) | 2.61(2.11-3.24) | 54.27(43.79-67.17) | 3.29(2.52-4.23) | 44.74(33.92-57.96) | -0.54(-0.68 - -0.41) |
| Southern Sub-Saharan Africa | 11.68(9.51-14.23) | 231.33(191.21-276.75) | 17.84(14.61-17.84) | 231.56(190.35-279.31) | -0.09(-0.21 - 0.03) | 2.66(2.12-3.25) | 0.94(0.74-1.17) | 5.03(3.74-6.02) | 0.97(0.73-1.17) | 0.14(-0.03 - 0.31) | 3.67(2.66-4.93) | 78.06(58.56-101.64) | 5.12(3.78-6.87) | 70.42(52.8-93.05) | -0.35(-0.39 - -0.32) |
| Tropical Latin America | 35.77(29.18-42.95) | 294.06(236.32-359.11) | 72.60(58.74-72.6) | 303.14(248.14-364.78) | 0.12(-0.12 - 0.36) | 11.44(10.23-13.09) | 1.32(1.20-1.45) | 25.61(22.6-28.33) | 1.12(1.00-1.25) | -0.04(-0.32 - 0.23) | 10.29(7.97-13.00) | 85.22(67.33-106.03) | 15.91(12.34-20.32) | 67.51(52.74-85.77) | -0.61(-0.72 - -0.51) |
| Western Europe | 69.32(57.29-81.68) | 150.87(124.72-177.62) | 95.88(76.56-95.88) | 166.38(136.12-196.99) | 0.78(0.46 - 1.10) | 53.10(45.15-56.36) | 0.92(0.78-0.98) | 52.44(44.34-64.99) | 0.46(0.40-0.57) | -2.26(-2.55 - -1.95) | 19.34(15.49-24.02) | 39.65(31.10-49.73) | 19.36(14.83-24.71) | 29.29(21.65-38.53) | -0.88(-0.95 - -0.81) |
| Western Sub-Saharan Africa | 27.38(22.77-32.74) | 162.96(136.58-192.00) | 70.30(58.82-70.30) | 189.38(159.99-221.15) | 0.43(0.41 - 0.46) | 15.25(8.20-22.80) | 1.46(0.92-2.50) | 27.27(18.69-43.16) | 1.36(0.97-2.25) | -0.10(-0.20 - 0.00) | 13.30(8.70-17.24) | 78.78(56.79-103.42) | 25.75(19.00-33.28) | 74.3(55.7-97.6) | -0.21(-0.24 - -0.17) |
| IFAH: Inguinal, femoral, and abdominal hernia; ASR, age- standardised incidence rate; EAPC, estimated annual percentage change; UI, uncertainty interval. | | | | | | | | | | | | | | | |
