# Supplementary material for: Global, regional, and national burden of digestive diseases: findings from the global burden of disease study 2019
Source: Front Public Health. 2023 Aug 24;11:1202980. doi: 10.3389/fpubh.2023.1202980 (PMC10483149; doi:10.3389/fpubh.2023.1202980)
Supplement: Supplementary file 6 [file Table_6.docx]

| Table S6. The Incidence, Death, and DALYs of IFBD in 1990 and 2019 | | | | | | | | | | | | | | | |
| --- | --- | --- | --- | --- | --- | --- | --- | --- | --- | --- | --- | --- | --- | --- | --- |
| Characteristics | 1990 | | 2019 | | 1990-2019 | 1990 | | 2019 | | 1990-2019 | 1990 | | 2019 | | 1990-2019 |
|  | Incidence cases  No×10^2^ (95%UI) | ASR per 100 000  No (95% UI) | Incidence cases  No×10^2^ (95%UI) | ASR per 100 000  No (95% UI) | EAPC  No (95% CI) | Death cases  No×10^2^ (95%UI) | ASR per 100 000  No (95% UI) | Death cases  No×10^2^ (95%UI) | ASR per 100 000  No (95% CI) | EAPC  No (95% CI) | DALYs  No×10^3^ (95%UI) | ASR per 100 000  No (95% UI) | DALYs  No×10^3^ (95%UI) | ASR per 100 000  No (95% UI) | EAPC  No (95% CI) |
| Global | 2935.72(2574.25-3366.51) | 6.10(5.35-6.96) | 4045.52(3605.21-4564.78) | 4.97(4.43-5.59) | -0.60(-0.73 - -0.48) | 242.95(202.58-446.61) | 0.67(0.57-0.78) | 409.98(349.33-446.61) | 0.54(0.46-0.59) | -0.69(-0.81 - -0.57) | 1248.27(963.01-1503.17) | 27.20(21.70-32.39) | 1622.50(1356.30-1915.04) | 20.15(16.86-23.71) | -1.04(-1.06 - -1.01) |
| Sex |  |  |  |  |  |  |  |  |  |  |  |  |  |  |  |
| Female | 1404.98(1233.61-1611.73) | 5.80(5.12-6.61) | 1907.38(1711.54-2137.78) | 4.64(4.16-5.20) | -0.65(-0.78 - -0.53) | 129.47(99.53-248.38) | 0.63(0.50-0.74) | 223.65(187.59-248.38) | 0.52(0.43-0.58) | -0.64(-0.80 - -0.47) | 647.31(465.17-785.22) | 27.09(20.30-32.42) | 819.75(676.70-971.58) | 19.70(16.16-23.49) | -1.09(-1.12 - -1.06) |
| Male | 1530.74(1333.44-1746.4) | 6.39(5.60-7.29) | 2138.14(1897.59-2413.04) | 5.30(4.72-5.99) | -0.55(-0.68 - -0.43) | 113.47(90.02-209.38) | 0.69(0.56-0.88) | 186.33(150.6-209.38) | 0.56(0.45-0.62) | -0.78(-0.83 - -0.72) | 600.96(466.03-736.98) | 27.40(21.78-33.22) | 802.75(663.56-949.34) | 20.64(17.16-24.31) | -0.99(-1.01 - -0.97) |
| SDI |  |  |  |  |  |  |  |  |  |  |  |  |  |  |  |
| Low SDI | 52.79(43.95-63.79) | 1.46(1.23-1.77) | 122.15(101.64-147.93) | 1.52(1.29-1.83) | 0.37(0.31 - 0.44) | 18.93(11.44-40.16) | 0.67(0.48-0.99) | 31.25(24.42-40.16) | 0.55(0.43-0.70) | -0.80(-0.86 - -0.74) | 92.72(45.63-147.57) | 22.19(14.49-29.80) | 137.87(100.26-180.82) | 17.19(13.57-21.70) | -0.94(-0.99 - -0.89) |
| Low-middle SDI | 174.34(145.32-209.35) | 2.00(1.70-2.40) | 366.81(308.62-440.31) | 2.21(1.88-2.65) | 0.46(0.35 - 0.57) | 45.33(30.18-73.92) | 0.74(0.47-1.06) | 61.80(49.94-73.92) | 0.48(0.38-0.58) | -1.75(-1.90 - -1.60) | 203.02(126.47-268.42) | 23.29(16.18-30.53) | 236.58(189.25-283.80) | 15.37(12.47-18.32) | -1.58(-1.65 - -1.51) |
| Middle SDI | 274.59(232.59-323.10) | 1.83(1.58-2.15) | 598.17(509.73-706.48) | 2.27(1.94-2.68) | 1.07(0.95 - 1.18) | 54.25(41.46-73.41) | 0.57(0.40-0.74) | 65.98(56.02-73.41) | 0.31(0.26-0.34) | -2.44(-2.59 - -2.29) | 248.14(184.99-297.50) | 18.11(14.29-21.72) | 291.09(240.41-345.83) | 11.61(9.70-13.71) | -1.63(-1.68 - -1.58) |
| High-middle SDI | 714.74(625.45-815.10) | 6.12(5.36-6.99) | 945.53(841.01-1071.56) | 5.58(4.97-6.28) | -0.35(-0.45 - -0.24) | 50.45(44.76-81.10) | 0.52(0.46-0.60) | 72.72(65.19-81.10) | 0.38(0.34-0.43) | -1.17(-1.32 - -1.02) | 288.34(236.22-344.58) | 25.92(21.34-30.93) | 352.79(285.25-432.62) | 19.34(15.59-23.73) | -1.12(-1.17 - -1.07) |
| High SDI | 1718.41(1518.78-1957.46) | 18.96(16.73-21.58) | 1964.51(1795.56-2175.10) | 17.61(16.03-19.41) | -0.16(-0.30 - -0.01) | 73.86(66.56-195.52) | 0.72(0.65-0.85) | 178.01(142.15-195.52) | 0.84(0.69-0.92) | 0.82(0.64 - 1.01) | 415.48(323.78-520.63) | 43.95(34.13-55.06) | 603.33(489.51-729.63) | 42.93(33.66-53.08) | 0.00(-0.06 - 0.05) |
| Region |  |  |  |  |  |  |  |  |  |  |  |  |  |  |  |
| Andean Latin America | 4.72(4.02-5.50) | 1.63(1.41-1.91) | 11.28(9.75-13.14) | 1.82(1.57-2.12) | -0.05(-0.29 - 0.20) | 1.68(0.99-1.82) | 0.54(0.36-0.71) | 1.44(1.11-1.82) | 0.26(0.20-0.32) | -2.74(-3.16 - -2.32) | 10.77(5.53-17.35) | 26.36(15.78-38.96) | 5.94(4.67-7.39) | 9.83(7.75-12.19) | -3.68(-4.17 - -3.18) |
| Australasia | 29.79(25.56-34.81) | 13.56(11.64-15.80) | 64.46(57.55-72.12) | 20.03(17.79-22.57) | 0.94(0.72 - 1.16) | 1.17(1.03-4.65) | 0.54(0.47-0.64) | 4.00(2.91-4.65) | 0.72(0.53-0.83) | 1.77(1.41 - 2.13) | 5.80(4.53-7.30) | 26.19(20.48-32.91) | 13.33(10.49-16.56) | 34.98(26.87-44.64) | 0.93(0.75 - 1.12) |
| Caribbean | 6.77(5.80-7.96) | 2.11(1.83-2.49) | 13.12(11.21-15.71) | 2.63(2.24-3.14) | 0.49(0.34 - 0.64) | 1.57(1.18-2.64) | 0.56(0.44-0.69) | 2.10(1.56-2.64) | 0.42(0.31-0.53) | -1.24(-1.41 - -1.08) | 7.45(5.01-11.79) | 23.38(16.68-34.05) | 8.90(6.52-11.71) | 18.21(13.08-24.76) | -0.91(-1.06 - -0.76) |
| Central Asia | 35.14(29.95-41.46) | 5.99(5.13-7.07) | 64.61(54.87-76.37) | 6.90(5.87-8.13) | 0.50(0.46 - 0.53) | 2.15(1.67-2.88) | 0.39(0.30-0.46) | 2.49(2.18-2.88) | 0.32(0.28-0.37) | -1.00(-1.12 - -0.89) | 15.95(12.80-19.23) | 26.11(21.03-31.71) | 20.24(16.36-24.81) | 22.26(18.04-27.31) | -0.70(-0.76 - -0.65) |
| Central Europe | 209.97(180.82-241.70) | 15.78(13.61-18.19) | 148.09(134.43-163.91) | 11.59(10.51-12.81) | -1.38(-1.76 - -0.99) | 6.04(5.63-11.04) | 0.46(0.43-0.52) | 9.58(7.99-11.04) | 0.47(0.40-0.55) | 0.30(0.17 - 0.44) | 61.55(47.42-77.41) | 46.28(36.16-57.91) | 54.69(42.73-67.57) | 35.84(27.66-44.67) | -1.08(-1.37 - -0.78) |
| Central Latin America | 39.68(34.02-46.18) | 3.16(2.74-3.66) | 67.54(58.34-78.26) | 2.64(2.29-3.06) | -0.70(-0.87 - -0.54) | 3.01(2.70-9.97) | 0.32(0.28-0.35) | 8.54(7.02-9.97) | 0.37(0.30-0.43) | 0.98(0.68 - 1.28) | 18.10(15.46-20.85) | 14.84(12.69-17.31) | 36.18(30.22-43.01) | 14.68(12.25-17.41) | 0.32(0.08 - 0.56) |
| Central Sub-Saharan Africa | 3.81(3.18-4.69) | 1.04(0.88-1.26) | 11.50(9.55-14.00) | 1.26(1.06-1.52) | 0.64(0.59 - 0.70) | 1.93(1.18-4.97) | 0.72(0.51-1.03) | 3.55(2.54-4.97) | 0.60(0.45-0.83) | -0.69(-0.77 - -0.60) | 9.49(4.86-17.73) | 22.26(14.75-32.74) | 15.57(10.73-22.38) | 17.77(12.98-24.27) | -0.79(-0.88 - -0.7) |
| East Asia | 175.67(146.27-208.02) | 1.45(1.22-1.71) | 523.19(446.80-614.55) | 2.95(2.55-3.43) | 2.54(2.39 - 2.69) | 57.34(41.69-57.69) | 0.86(0.59-1.14) | 49.83(40.74-57.69) | 0.31(0.25-0.35) | -3.98(-4.33 - -3.63) | 240.23(167.51-299.89) | 24.23(17.74-29.83) | 239.96(187.12-299.81) | 13.08(10.31-16.19) | -2.31(-2.48 - -2.15) |
| Eastern Europe | 181.50(156.00-212.09) | 7.10(6.12-8.29) | 195.00(168.38-226.71) | 7.34(6.34-8.55) | 0.07(-0.02 - 0.17) | 13.05(9.92-18.65) | 0.49(0.38-0.56) | 16.33(13.70-18.65) | 0.51(0.43-0.58) | -0.25(-0.53 - 0.04) | 72.93(57.53-88.87) | 27.68(21.86-33.71) | 77.12(63.29-94.01) | 26.67(21.96-32.46) | -0.30(-0.45 - -0.14) |
| Eastern Sub-Saharan Africa | 10.92(9.08-13.15) | 0.89(0.76-1.07) | 29.38(24.49-35.38) | 1.04(0.88-1.25) | 0.55(0.48 - 0.61) | 6.97(4.62-16.78) | 0.72(0.52-1.01) | 12.53(9.23-16.78) | 0.69(0.50-0.93) | -0.12(-0.20 - -0.05) | 35.48(16.92-68.48) | 22.58(16.13-33.35) | 52.93(39.43-74.04) | 19.44(14.69-25.63) | -0.46(-0.54 - -0.39) |
| High-income Asia Pacific | 181.05(153.91-214.22) | 9.25(7.88-10.91) | 270.84(238.70-306.21) | 14.93(13.14-16.93) | 1.10(0.84 - 1.37) | 6.85(4.39-9.12) | 0.40(0.24-0.47) | 6.94(5.56-9.12) | 0.13(0.11-0.18) | -4.29(-4.64 - -3.93) | 51.70(38.59-66.56) | 26.43(19.87-33.86) | 78.60(55.26-105.05) | 34.55(23.75-46.76) | 0.35(0.11 - 0.60) |
| High-income North America | 1076.16(941.47-1238.84) | 34.88(30.59-40.02) | 996.11(919.45-1090.00) | 24.51(22.65-26.77) | -0.82(-1.20 - -0.45) | 24.42(21.89-68.86) | 0.68(0.61-0.83) | 63.14(50.76-68.86) | 0.97(0.79-1.05) | 1.43(1.15 - 1.72) | 209.07(155.33-270.98) | 65.62(48.61-84.95) | 245.23(200.56-294.02) | 51.27(40.90-62.35) | -0.46(-0.72 - -0.20) |
| North Africa and Middle East | 81.07(70.40-92.04) | 2.95(2.59-3.38) | 227.22(192.77-269.52) | 3.74(3.19-4.45) | 0.62(0.53 - 0.72) | 5.92(4.07-12.26) | 0.34(0.24-0.49) | 10.33(8.66-12.26) | 0.26(0.22-0.30) | -0.86(-0.93 - -0.80) | 35.44(25.57-46.32) | 14.36(10.79-18.73) | 72.75(56.93-91.11) | 13.26(10.60-16.47) | -0.30(-0.34 - -0.25) |
| Oceania | 0.22(0.18-0.27) | 0.44(0.36-0.52) | 0.63(0.52-0.77) | 0.56(0.47-0.67) | 0.87(0.76 - 0.98) | 0.22(0.14-0.51) | 0.63(0.42-0.86) | 0.36(0.26-0.51) | 0.44(0.33-0.60) | -1.41(-1.50 - -1.32) | 1.04(0.60-1.63) | 20.60(13.34-28.78) | 1.70(1.11-2.58) | 14.93(10.45-21.01) | -1.29(-1.38 - -1.20) |
| South Asia | 184.39(151.69-225.82) | 2.15(1.79-2.64) | 397.05(329.43-484.54) | 2.32(1.93-2.84) | 0.47(0.31 - 0.63) | 38.64(25.33-74.10) | 0.69(0.47-1.06) | 57.02(42.86-74.10) | 0.44(0.33-0.57) | -1.91(-2.07 - -1.74) | 171.45(105.18-227.33) | 21.48(14.77-29.47) | 221.98(166.88-278.49) | 14.00(10.70-17.52) | -1.68(-1.78 - -1.58) |
| Southeast Asia | 18.82(15.27-22.7) | 0.46(0.38-0.56) | 49.8(41.82-59.72) | 0.70(0.59-0.84) | 1.45(1.27 - 1.63) | 12.30(7.41-17.71) | 0.50(0.27-0.66) | 14.98(11.39-17.71) | 0.28(0.21-0.33) | -2.36(-2.52 - -2.19) | 45.32(30.76-56.84) | 13.16(8.47-15.90) | 46.69(38.85-55.19) | 7.45(6.06-8.76) | -2.29(-2.44 - -2.15) |
| Southern Latin America | 8.79(7.42-10.30) | 1.82(1.54-2.13) | 13.82(11.77-16.20) | 1.92(1.63-2.26) | 0.24(0.15 - 0.34) | 1.39(1.23-2.80) | 0.31(0.27-0.34) | 2.36(2.10-2.80) | 0.28(0.25-0.34) | -0.46(-0.62 - -0.30) | 5.25(4.58-5.99) | 11.10(9.69-12.66) | 7.74(6.67-9.15) | 10.06(8.61-11.94) | -0.40(-0.50 - -0.31) |
| Southern Sub-Saharan Africa | 5.04(4.23-6.08) | 1.30(1.10-1.56) | 9.89(8.37-11.88) | 1.37(1.17-1.65) | 0.13(0.06 - 0.20) | 1.38(0.99-2.78) | 0.42(0.33-0.53) | 2.40(1.98-2.78) | 0.44(0.36-0.51) | 0.25(-0.16 - 0.65) | 6.61(4.28-9.52) | 15.11(10.88-18.87) | 9.23(7.54-10.93) | 13.66(11.37-15.90) | -0.12(-0.44 - 0.20) |
| Tropical Latin America | 78.26(68.59-89.76) | 6.09(5.40-6.93) | 129.28(113.34-148.74) | 5.25(4.60-6.03) | -0.55(-0.59 - -0.51) | 4.55(4.00-12.17) | 0.47(0.41-0.51) | 10.47(9.50-12.17) | 0.44(0.40-0.51) | 0.03(-0.14 - 0.21) | 27.72(23.59-32.36) | 23.37(19.94-27.30) | 47.00(40.11-54.97) | 19.26(16.53-22.44) | -0.44(-0.61 - -0.26) |
| Western Europe | 590.81(537.33-655.29) | 13.98(12.67-15.48) | 786.45(716.11-867.09) | 16.94(15.33-18.73) | 0.60(0.55 - 0.65) | 46.00(41.32-133.15) | 0.82(0.73-1.04) | 119.81(92.44-133.15) | 1.11(0.87-1.23) | 1.51(1.27 - 1.75) | 185.73(149.51-227.83) | 39.24(31.18-48.16) | 310.69(255.91-371.11) | 46.79(36.98-57.57) | -0.44(-0.61 - -0.26) |
| Western Sub-Saharan Africa | 13.14(11.02-15.99) | 1.01(0.86-1.23) | 36.28(30.28-44.01) | 1.17(1.00-1.41) | 0.36(0.22 - 0.50) | 6.34(3.65-16.10) | 0.56(0.33-0.91) | 11.78(8.05-16.10) | 0.50(0.35-0.67) | -0.37(-0.46 - -0.28) | 31.20(14.38-47.87) | 19.69(11.64-30.47) | 56.03(35.33-78.05) | 16.77(11.61-22.73) | -0.57(-0.66 - -0.48) |
| IFBD: Inflamma-ry bowel disease; ASR, age- standardised incidence rate; EAPC, estimated annual percentage change; UI, uncertainty interval. | | | | | | | | | | | | | | | |
