# Supplementary material for: Global, regional, and national burden of digestive diseases: findings from the global burden of disease study 2019
Source: Front Public Health. 2023 Aug 24;11:1202980. doi: 10.3389/fpubh.2023.1202980 (PMC10483149; doi:10.3389/fpubh.2023.1202980)
Supplement: Supplementary file 7 [file Table_7.docx]

| Table S7. The Incidence, Death, and DALYs of VAID in 1990 and 2019 | | | | | | | | | | | | | | | |
| --- | --- | --- | --- | --- | --- | --- | --- | --- | --- | --- | --- | --- | --- | --- | --- |
| Characteristics | 1990 | | 2019 | | 1990-2019 | 1990 | | 2019 | | 1990-2019 | 1990 | | 2019 | | 1990-2019 |
|  | Incidence cases  No×10^4^ (95%UI) | ASR per 100 000  No (95% UI) | Incidence cases  No×10^4^ (95%UI) | ASR per 100 000  No (95% UI) | EAPC  No (95% CI) | Death cases  No×10^2^ (95%UI) | ASR per 100 000  No (95% UI) | Death cases  No×10^2^ (95%UI) | ASR per 100 000  No (95% UI) | EAPC  No (95% CI) | DALYs  No×10^4^ (95%UI) | ASR per 100 000  No (95% UI) | DALYs  No×10^4^ (95%UI) | ASR per 100 000  No (95% UI) | EAPC  No (95% CI) |
| Global | 84.03(72.42-96.71) | 20.89(17.96-23.74) | 145.64(126.49-165.31) | 18.23(15.84-20.67) | -0.38(-0.43 - -0.34) | 545.83(512.03-573.03) | 1.66(1.53-1.75) | 1065.76(951.13-1164.28) | 1.40(1.24-1.53) | -0.68(-0.77 - -0.58) | 109.15(102.01-117.03) | 28.58(26.81-30.28) | 187.23(172.2-203.44) | 23.49(21.58-25.59) | -0.78(-0.85 - -0.70) |
| Sex |  |  |  |  |  |  |  |  |  |  |  |  |  |  |  |
| Female | 45.78(39.54-52.29) | 21.12(18.15-23.94) | 77.18(67.14-87.39) | 18.09(15.71-20.50) | -0.45(-0.49 - -0.41) | 311.04(284.32-335.35) | 1.63(1.48-1.75) | 615.49(534.82-690.40) | 1.40(1.22-1.57) | -0.60(-0.71 - -0.49) | 54.63(49.94-60.34) | 26.33(24.11-28.84) | 96.34(85.79-108.59) | 22.12(19.69-24.95) | -0.68(-0.76 - -0.60) |
| Male | 38.25(32.76-44.4) | 20.28(17.48-23.22) | 68.46(58.90-78.23) | 18.23(15.85-20.75) | -0.27(-0.33 - -0.22) | 234.79(218.08-249.77) | 1.63(1.50-1.74) | 450.27(412.56-484.16) | 1.35(1.23-1.46) | -0.74(-0.81 - -0.67) | 54.52(50.13-58.97) | 30.42(28.28-32.53) | 90.89(83.93-98.02) | 24.57(22.62-26.58) | -0.85(-0.92 - -0.79) |
| SDI |  |  |  |  |  |  |  |  |  |  |  |  |  |  |  |
| Low SDI | 2.63(2.10-3.26) | 7.31(6.19-8.60) | 6.40(5.19-7.86) | 8.33(7.08-9.78) | 0.74(0.57 - 0.90) | 25.82(19.46-33.24) | 1.32(1.06-1.62) | 57.56(47.17-68.96) | 1.37(1.13-1.61) | 0.02(-0.02 - 0.07) | 7.80(5.04-11.28) | 26.71(20.33-34.40) | 14.67(11.64-18.53) | 25.73(21.09-31.05) | -0.22(-0.27 - -0.16) |
| Low-middle SDI | 6.35(5.17-7.78) | 7.84(6.56-9.34) | 15.05(12.57-18.02) | 9.90(8.37-11.65) | 0.99(0.84 - 0.84) | 50.45(41.61-59.91) | 1.10(0.9-1.32) | 136.62(111.25-170.70) | 1.19(0.98-1.47) | 0.23(0.17 - 0.28) | 12.53(10.17-15.53) | 20.58(16.97-24.48) | 28.76(23.27-36.10) | 21.62(17.58-27.02) | 0.13(0.09 - 0.17) |
| Middle SDI | 10.18(8.46-12.25) | 8.50(7.14-9.94) | 21.41(17.78-25.35) | 8.94(7.51-10.43) | 0.29(0.17 - 0.40) | 70.30(64.59-75.97) | 0.92(0.84-1.01) | 177.14(159.26-194.27) | 0.87(0.78-0.96) | -0.20(-0.24 - -0.16) | 16.07(14.74-17.7) | 16.18(14.89-17.53) | 33.45(30.71-36.35) | 14.49(13.24-15.78) | -0.41(-0.43 - -0.38) |
| High-middle SDI | 22.06(18.84-25.38) | 20.90(17.99-23.92) | 35.47(30.45-40.21) | 18.64(16.21-21.14) | -0.39(-0.45 - -0.32) | 178.61(167.64-187.78) | 1.93(1.78-2.03) | 350.58(315.32-377.45) | 1.78(1.60-1.92) | -0.26(-0.41 - -0.12) | 35.65(33.76-37.84) | 34.57(32.66-36.63) | 58.33(53.78-62.17) | 29.17(26.87-31.07) | -0.66(-0.81 - -0.52) |
| High SDI | 42.78(36.86-48.47) | 42.21(36.42-48.14) | 65.93(57.51-73.54) | 38.85(33.95-43.73) | -0.18(-0.22 - -0.14) | 220.36(202.37-231.40) | 2.09(1.91-2.19) | 343.33(298.63-375.70) | 1.59(1.41-1.73) | -1.14(-1.27 - -1.01) | 37.05(35.03-38.58) | 35.44(33.53-36.88) | 51.93(47.47-55.19) | 27.42(25.36-28.96) | -1.07(-1.17 - -0.97) |
| Region |  |  |  |  |  |  |  |  |  |  |  |  |  |  |  |
| Andean Latin America | 0.15(0.13-0.18) | 6.00(5.09-6.95) | 0.42(0.36-0.49) | 7.35(6.22-8.55) | 0.67(0.58 - 0.75) | 2.42(1.96-2.93) | 1.23(1.02-1.43) | 5.87(4.79-7.15) | 1.10(0.89-1.34) | -0.23(-0.39 - -0.06) | 0.70(0.50-0.98) | 25.82(20.35-32.79) | 1.05(0.86-1.27) | 18.79(15.49-22.84) | -1.05(-1.29 - -0.81) |
| Australasia | 0.67(0.56-0.80) | 28.61(23.95-33.75) | 1.27(1.01-1.53) | 26.69(21.56-32.06) | -0.15(-0.20 - -0.1) | 4.19(3.75-4.51) | 1.85(1.65-1.99) | 7.40(6.15-8.42) | 1.32(1.11-1.50) | -1.25(-1.32 - -1.19) | 0.71(0.65-0.76) | 30.46(28-32.45) | 1.03(0.90-1.15) | 20.28(17.83-22.53) | -1.54(-1.62 - -1.47) |
| Caribbean | 0.32(0.27-0.37) | 11.19(9.50-13.16) | 0.63(0.53-0.73) | 12.42(10.58-14.52) | 0.45(0.38 - 0.51) | 3.70(3.29-4.16) | 1.51(1.34-1.70) | 6.60(5.44-8.00) | 1.28(1.05-1.55) | -0.63(-0.82 - -0.44) | 0.79(0.69-0.91) | 29.58(26.14-33.7) | 1.25(1.04-1.52) | 24.36(20.24-29.59) | -0.71(-0.91 - -0.52) |
| Central Asia | 0.81(0.66-1.01) | 13.43(11.33-15.94) | 1.40(1.16-1.70) | 17.29(14.69-20.44) | 1.14(0.97 - 1.31) | 4.16(3.76-4.71) | 0.99(0.88-1.13) | 6.50(5.84-7.19) | 1.17(1.04-1.31) | 0.65(0.50 - 0.80) | 0.93(0.87-1.03) | 19.72(18.29-21.97) | 1.42(1.28-1.58) | 20.99(18.90-23.17) | 0.16(0.01 - 0.31) |
| Central Europe | 2.24(1.92-2.64) | 16.80(14.32-19.89) | 3.13(2.73-3.57) | 18.72(16.26-21.65) | 0.05(-0.05 - 0.15) | 24.98(23.57-26.13) | 1.81(1.70-1.90) | 47.91(42.05-53.66) | 2.13(1.87-2.39) | 0.59(0.22 - 0.97) | 5.13(4.88-5.35) | 35.60(33.93-37.15) | 8.00(7.10-8.99) | 37.67(33.46-42.28) | 0.14(-0.16 - 0.45) |
| Central Latin America | 1.76(1.49-2.08) | 17.84(15.40-20.39) | 3.47(2.98-4.03) | 14.86(12.78-17.21) | -0.79(-0.90 - -0.68) | 16.97(15.93-17.71) | 2.33(2.16-2.45) | 50.34(42.84-58.26) | 2.26(1.92-2.62) | -0.21(-0.27 - -0.14) | 3.86(3.70-4.02) | 43.29(41.19-44.99) | 9.13(7.82-10.50) | 39.36(33.67-45.24) | -0.40(-0.45 - -0.35) |
| Central Sub-Saharan Africa | 0.31(0.26-0.38) | 9.89(8.54-11.37) | 0.84(0.69-1.01) | 11.19(9.59-12.90) | 0.40(0.23 - 0.56) | 4.36(2.02-7.48) | 2.49(1.26-4.06) | 8.14(5.01-12.43) | 1.99(1.24-2.93) | -0.91(-1.08 - -0.74) | 1.29(0.53-2.30) | 49.25(23.58-84.67) | 2.14(1.31-3.35) | 37.93(23.36-58.06) | -1.02(-1.20 - -0.84) |
| East Asia | 5.87(4.67-7.06) | 6.39(5.15-7.58) | 12.26(9.97-14.55) | 6.29(5.20-7.35) | -0.22(-0.39 - -0.04) | 30.37(26.10-37.95) | 0.46(0.40-0.56) | 71.13(58.6-81.26) | 0.40(0.33-0.46) | -0.34(-0.44 - -0.24) | 6.86(5.75-8.83) | 8.33(7.15-10.43) | 13.01(10.67-14.93) | 6.64(5.48-7.57) | -0.67(-0.75 - -0.58) |
| Eastern Europe | 10.83(9.19-12.39) | 40.70(34.85-46.31) | 15.49(13.5-17.47) | 47.43(41.54-53.33) | 0.72(0.61 - 0.84) | 78.86(71.73-88.02) | 3.03(2.73-3.37) | 151.92(135.84-168.17) | 4.32(3.87-4.78) | 1.43(1.23 - 1.63) | 15.98(14.38-18.42) | 58.41(52.59-67.17) | 26.35(23.62-29.16) | 76.94(68.97-85.29) | 0.95(0.74 - 1.16) |
| Eastern Sub-Saharan Africa | 0.81(0.65-1.02) | 6.88(5.90-7.95) | 2.25(1.84-2.78) | 8.97(7.70-10.39) | 0.98(0.87 - 1.09) | 9.32(7.54-11.29) | 1.56(1.20-1.94) | 22.78(16.39-28.26) | 1.80(1.29-2.25) | 0.46(0.37 - 0.54) | 2.54(1.84-3.48) | 29.66(24.01-35.83) | 5.44(4.02-6.65) | 32.29(23.33-40.05) | 0.28(0.18 - 0.37) |
| High-income Asia Pacific | 7.84(6.33-9.44) | 39.82(32.24-47.81) | 14.90(12.5-17.22) | 44.69(36.66-53.24) | 0.48(0.38 - 0.57) | 12.82(11.90-13.63) | 0.71(0.65-0.75) | 44.02(34.64-50.67) | 0.75(0.61-0.84) | 0.46(0.29 - 0.63) | 2.56(2.40-2.75) | 13.23(12.36-14.19) | 5.84(5.01-6.37) | 12.59(11.20-13.70) | 0.00(-0.13 - 0.14) |
| High-income North America | 21.95(19.16-24.48) | 63.05(54.99-70.67) | 30.76(27.57-33.81) | 52.88(47.44-58.00) | -0.59(-0.63 - -0.54) | 85.06(77.17-89.40) | 2.31(2.10-2.43) | 124.98(111.13-134.32) | 1.86(1.68-1.99) | -1.06(-1.28 - -0.83) | 15.04(14.12-15.65) | 42.78(40.29-44.48) | 21.19(19.64-22.32) | 34.78(32.41-36.53) | -1.01(-1.18 - -0.83) |
| North Africa and Middle East | 1.77(1.46-2.13) | 7.88(6.52-9.32) | 5.71(4.62-6.87) | 11.89(9.83-14.07) | 1.21(1.12 - 1.30) | 10.52(8.41-12.24) | 0.77(0.62-0.91) | 23.65(20.39-27.08) | 0.68(0.59-0.78) | -0.31(-0.39 - -0.23) | 2.36(1.87-2.77) | 14.17(11.33-16.36) | 4.89(4.21-5.64) | 11.95(10.33-13.67) | -0.53(-0.57 - -0.48) |
| Oceania | 0.02(0.02-0.02) | 4.36(3.52-5.27) | 0.05(0.04-0.06) | 5.18(4.20-6.23) | 0.50(0.44 - 0.57) | 0.11(0.09-0.13) | 0.46(0.37-0.56) | 0.28(0.22-0.36) | 0.49(0.40-0.60) | 0.26(0.22 - 0.30) | 0.03(0.03-0.04) | 9.67(7.89-11.52) | 0.08(0.06-0.11) | 10.59(8.36-13.32) | 0.39(0.33 - 0.45) |
| South Asia | 6.87(5.53-8.55) | 8.66(7.20-10.38) | 17.98(14.87-21.88) | 11.36(9.50-13.54) | 1.08(0.96 - 1.20) | 49.67(38.77-62.74) | 1.25(0.96-1.56) | 145.8(107.44-195.39) | 1.28(0.96-1.68) | -0.10(-0.25 - 0.05) | 11.99(9.24-15.65) | 22.38(17.54-28.22) | 29.89(21.74-40.67) | 22.33(16.43-30.1) | -0.15(-0.25 - -0.04) |
| Southeast Asia | 1.98(1.64-2.39) | 6.46(5.38-7.57) | 4.92(3.99-5.88) | 8.24(6.83-9.68) | 0.84(0.79 - 0.88) | 16.01(13.52-18.62) | 0.86(0.72-1.04) | 41.34(35.71-47.06) | 0.90(0.77-1.02) | -0.01(-0.07 - 0.05) | 3.34(2.85-3.82) | 14.11(11.98-16.17) | 7.34(6.44-8.33) | 13.76(12.06-15.67) | -0.24(-0.31 - -0.18) |
| Southern Latin America | 0.79(0.66-0.92) | 17.31(14.41-20.05) | 1.61(1.35-1.92) | 19.66(16.49-23.32) | 0.17(0.06 - 0.28) | 11.89(10.94-12.89) | 2.79(2.56-3.03) | 18.89(16.78-20.97) | 2.20(1.96-2.45) | -0.87(-1.01 - -0.73) | 2.25(2.10-2.43) | 49.52(46.07-53.44) | 3.15(2.87-3.47) | 37.85(34.51-41.62) | -0.95(-1.05 - -0.84) |
| Southern Sub-Saharan Africa | 0.39(0.31-0.49) | 9.29(7.82-11.09) | 0.70(0.57-0.86) | 10.16(8.51-12.14) | 0.35(0.25 - 0.44) | 1.78(1.44-2.26) | 0.70(0.57-0.91) | 4.09(3.59-4.55) | 0.83(0.74-0.93) | 0.51(0.34 - 0.69) | 0.49(0.39-0.59) | 15.29(12.34-18.89) | 1.00(0.88-1.12) | 16.89(14.88-18.74) | 0.30(0.10 - 0.49) |
| Tropical Latin America | 1.98(1.65-2.37) | 18.68(15.88-21.88) | 2.39(2.08-2.76) | 10.11(8.82-11.64) | -1.17(-1.77 - -0.56) | 22.81(21.43-23.96) | 2.90(2.68-3.07) | 44.97(40.69-49.76) | 1.95(1.75-2.16) | -1.5(-1.56 - -1.43) | 5.58(5.33-5.82) | 58.50(55.39-61.19) | 9.11(8.43-9.83) | 37.94(35.04-41.00) | -1.60(-1.68 - -1.52) |
| Western Europe | 15.50(13.03-18.08) | 27.63(23.48-32.07) | 22.29(18.86-26.11) | 26.67(22.66-30.95) | 0.04(-0.04 - 0.12) | 147.66(136.61-155.96) | 2.46(2.26-2.59) | 221.41(192.69-245.4) | 2.01(1.78-2.21) | -0.91(-1.08 - -0.74) | 23.27(21.95-24.36) | 39.64(37.47-41.49) | 29.98(27.15-32.39) | 31.42(28.79-33.67) | -0.99(-1.13 - -0.86) |
| Western Sub-Saharan Africa | 1.16(0.91-1.49) | 7.50(6.22-9.03) | 3.15(2.46-4.01) | 9.17(7.68-10.95) | 0.72(0.68 - 0.77) | 8.19(6.52-10.36) | 0.88(0.67-1.15) | 17.74(13.53-22.98) | 1.03(0.80-1.37) | 0.74(0.63 - 0.85) | 3.45(2.21-5.10) | 21.77(17.32-27.51) | 5.92(4.45-7.91) | 21.72(16.57-28.23) | 0.06(0.00 - 0.13) |
| VAID: Vascular intestinal disorders; ASR, age- standardised incidence rate; EAPC, estimated annual percentage change; UI, uncertainty interval. | | | | | | | | | | | | | | | |
