# Supplementary material for: Global, regional, and national burden of digestive diseases: findings from the global burden of disease study 2019
Source: Front Public Health. 2023 Aug 24;11:1202980. doi: 10.3389/fpubh.2023.1202980 (PMC10483149; doi:10.3389/fpubh.2023.1202980)
Supplement: Supplementary file 8 [file Table_8.docx]

| Table S8. The Incidence, Death, and DALYs of GABD in 1990 and 2019 | | | | | | | | | | | | | | | |
| --- | --- | --- | --- | --- | --- | --- | --- | --- | --- | --- | --- | --- | --- | --- | --- |
| Characteristics | 1990 | | 2019 | | 1990-2019 | 1990 | | 2019 | | 1990-2019 | 1990 | | 2019 | | 1990-2019 |
|  | Incidence cases  No×10^5^ (95%UI) | ASR per 100 000  No (95% UI) | Incidence cases  No×10^5^ (95%UI) | ASR per 100 000  No (95% UI) | EAPC  No (95% CI) | Death cases  No×10^3^ (95%UI) | ASR per 100 000  No (95% UI) | Death cases  No×10^4^ (95%UI) | ASR per 100 000  No (95% UI) | EAPC  No (95% CI) | DALYs  No×10^5^ (95%UI) | ASR per 100 000  No (95% UI) | DALYs  No×10^5^ (95%UI) | ASR per 100 000  No (95% UI) | EAPC  No (95% CI) |
| Global | 263.51(227.9-305.98) | 585.35(506.05-679.86) | 520.04(442.02-612.12) | 634.32(540.21-742.93) | 0.59(0.48 - 0.69) | 82.43(66.63-90.66) | 2.40(1.99-2.64) | 12.49(10.71-13.86) | 1.65(1.41-1.84) | -1.22(-1.32 - -1.13) | 46.05(35.93-58.48) | 106.76(84.2-134.91) | 63.53(48.74-82.5) | 78.25(60.29-101.40) | -1.03(-1.13 - -0.93) |
| Sex |  |  |  |  |  |  |  |  |  |  |  |  |  |  |  |
| Female | 181.84(157.09-211.02) | 781.47(672.79-907.30) | 358.24(305.17-420.69) | 854.88(729.82-1000.98) | 0.61(0.50 - 0.71) | 50.02(40.63-55.04) | 2.54(2.07-2.78) | 7.22(6.02-8.29) | 1.66(1.38-1.90) | -1.41(-1.50 - -1.32) | 29.82(22.92-38.36) | 131.25(101.59-168.53) | 41.11(30.51-54.49) | 97.58(72.37-129.24) | -0.98(-1.07 - -0.88) |
| Male | 81.67(70.64-95.36) | 385.12(332.68-448.10) | 161.80(137.65-190.97) | 410.04(350.30-479.26) | 0.54(0.42 - 0.67) | 32.41(24.87-37.21) | 2.22(1.75-2.53) | 5.27(4.43-6.05) | 1.65(1.37-1.89) | -0.96(-1.06 - -0.86) | 16.23(12.62-20.32) | 81.49(64.81-100.4) | 22.42(17.84-28.11) | 58.69(47.13-73.01) | -1.09(-1.21 - -0.98) |
| SDI |  |  |  |  |  |  |  |  |  |  |  |  |  |  |  |
| Low SDI | 3.9(3.33-4.55) | 103.70(90.24-120.36) | 12.34(10.46-14.34) | 157.09(134.02-181.81) | 1.92(1.68 - 2.15) | 6.19(4.74-7.82) | 2.99(2.33-3.87) | 1.17(0.9-1.56) | 2.63(2.06-3.56) | -0.37(-0.40 - -0.33) | 2.66(1.97-3.3) | 83.64(67.08-103.85) | 4.79(3.76-5.95) | 72.15(57.82-91.69) | -0.41(-0.46 - -0.36) |
| Low-middle SDI | 25.49(21.92-29.74) | 300.92(262-349.18) | 68.59(58.77-80.33) | 427.78(368.26-497.83) | 1.53(1.36 - 1.71) | 11.64(8.58-13.94) | 2.27(1.70-2.75) | 1.79(1.57-2.08) | 1.53(1.34-1.78) | -1.34(-1.41 - -1.27) | 7.27(5.62-9.17) | 96.27(75.48-119.13) | 11.36(8.77-14.78) | 75.04(58.53-96.11) | -0.76(-0.88 - -0.63) |
| Middle SDI | 68.50(58.76-80.04) | 503.59(433.29-584.82) | 158.05(133.26-186.95) | 605.38(515.72-707.31) | 1.12(0.96 - 1.28) | 23.15(17.92-25.85) | 2.8(2.26-3.16) | 3.47(2.96-3.99) | 1.70(1.44-1.97) | -1.75(-1.81 - -1.68) | 14.44(11.12-18.5) | 118.90(93.32-148.94) | 20.58(15.58-27.16) | 81.21(62.44-106.09) | -1.26(-1.39 - -1.13) |
| High-middle SDI | 78.19(67.30-91.01) | 693.76(596.75-805.64) | 144.34(120.83-171.81) | 778.63(655.82-918.95) | 0.58(0.49 - 0.66) | 22.88(18.2-24.82) | 2.50(2.01-2.72) | 2.80(2.4-3.12) | 1.45(1.24-1.62) | -1.92(-1.99 - -1.85) | 12.70(9.67-16.47) | 116.61(89.95-150.22) | 14.8(10.96-19.79) | 78.17(57.66-104.70) | -1.47(-1.57 - -1.36) |
| High SDI | 87.36(74.88-102.24) | 922.35(791.33-1078.94) | 128.61(110.42-150) | 903.10(775.89-1051.05) | 0.35(0.22 - 0.47) | 18.53(16.09-20.69) | 1.78(1.54-1.99) | 3.26(2.53-3.68) | 1.42(1.14-1.60) | -0.50(-0.69 - -0.32) | 8.96(6.75-11.79) | 91.81(68.63-122.22) | 11.96(9.13-15.67) | 76.52(56.02-102.50) | -0.56(-0.63 - -0.48) |
| Region |  |  |  |  |  |  |  |  |  |  |  |  |  |  |  |
| Andean Latin America | 0.79(0.69-0.92) | 265.97(234.99-308.42) | 1.54(1.34-1.82) | 250.51(218.69-293.54) | -0.49(-0.67 - -0.30) | 1.01(0.82-1.19) | 5.00(4.14-5.86) | 0.17(0.14-0.22) | 3.16(2.52-4.00) | -1.32(-1.61 - -1.02) | 0.40(0.33-0.49) | 158.46(131.76-189.39) | 0.51(0.41-0.62) | 86.77(70.02-106.01) | -2.04(-2.32 - -1.76) |
| Australasia | 1.18(1.00-1.39) | 530.61(451.41-628.96) | 2.22(1.89-2.62) | 598.97(502.34-709.38) | 0.43(0.36 - 0.51) | 0.29(0.25-0.36) | 1.30(1.13-1.66) | 0.06(0.05-0.08) | 1.12(0.88-1.34) | -0.35(-0.42 - -0.27) | 0.13(0.10-0.18) | 59.57(44.8-79.17) | 0.23(0.17-0.30) | 55.12(39.82-74.93) | -0.25(-0.27 - -0.22) |
| Caribbean | 0.98(0.85-1.14) | 317.09(278.23-370.72) | 1.56(1.37-1.82) | 313.06(274.27-364.60) | 0.03(-0.01 - 0.08) | 0.58(0.44-0.67) | 2.29(1.72-2.61) | 0.07(0.06-0.09) | 1.41(1.12-1.72) | -1.59(-1.96 - -1.23) | 0.27(0.22-0.34) | 94.52(76.16-115.77) | 0.34(0.27-0.43) | 67.07(52.53-84.49) | -1.09(-1.33 - -0.86) |
| Central Asia | 2.30(2.01-2.69) | 405.77(356.56-475.24) | 3.87(3.35-4.58) | 425.29(371.01-499.57) | 0.20(0.16 - 0.24) | 0.65(0.51-0.70) | 1.44(1.15-1.56) | 0.07(0.06-0.08) | 1.04(0.90-1.26) | -1.27(-1.39 - -1.14) | 0.45(0.35-0.59) | 85.62(66.43-111) | 0.57(0.41-0.76) | 66.39(49.84-87.61) | -1.04(-1.12 - -0.96) |
| Central Europe | 14.45(12.66-16.66) | 1043.67(914.1-1201.67) | 16.40(14.22-19.08) | 1009.87(881.18-1171.35) | -0.12(-0.29 - 0.04) | 3.18(2.39-3.41) | 2.44(1.82-2.63) | 0.29(0.24-0.34) | 1.29(1.09-1.53) | -2.04(-2.43 - -1.66) | 1.97(1.48-2.63) | 140.04(105.37-186.97) | 1.68(1.22-2.29) | 95.66(68.06-131.2) | -1.27(-1.34 - -1.21) |
| Central Latin America | 12.04(10.31-14.17) | 944.90(822.58-1101.83) | 28.24(24.24-33) | 1112.70(958.42-1295.35) | 0.89(0.68 - 1.11) | 2.60(2.26-2.86) | 3.30(2.87-3.71) | 0.65(0.53-0.76) | 2.86(2.33-3.34) | -0.23(-0.49 - 0.03) | 2.07(1.57-2.76) | 186(144.25-242.42) | 3.93(2.97-5.17) | 158.41(120.62-206.94) | -0.26(-0.40 - -0.11) |
| Central Sub-Saharan Africa | 0.16(0.13-0.18) | 39.22(34.72-45.26) | 0.46(0.39-0.54) | 47.61(42.28-55.59) | 0.76(0.72 - 0.80) | 0.78(0.56-1.02) | 4.36(3.23-5.68) | 0.17(0.11-0.22) | 4.03(2.76-5.41) | -0.21(-0.26 - -0.15) | 0.27(0.19-0.34) | 98.24(73.97-126.33) | 0.54(0.36-0.71) | 86.35(60.62-111.87) | -0.37(-0.42 - -0.31) |
| East Asia | 76.66(65.75-89.66) | 719.06(612.13-836.76) | 188.20(156.84-227.43) | 958.77(807.46-1136.76) | 1.22(1.02 - 1.42) | 19.37(12.06-22.65) | 2.98(1.94-3.46) | 1.74(1.45-2.26) | 1.08(0.90-1.37) | -3.83(-3.96 - -3.71) | 14.54(10.52-19.33) | 149.08(109.68-193.87) | 17.1(11.87-24.08) | 86.89(60.59-120.86) | -1.98(-2.12 - -1.84) |
| Eastern Europe | 20.72(17.51-24.26) | 802.22(681.79-932.62) | 21.98(18.35-25.93) | 786.11(662.11-925.36) | -0.04(-0.09 - 0.02) | 5.25(3.78-5.71) | 2.02(1.47-2.20) | 0.44(0.38-0.57) | 1.26(1.08-1.64) | -1.92(-2.14 - -1.71) | 2.74(2.09-3.55) | 103.10(78.72-134.36) | 2.20(1.65-2.94) | 73.53(54.46-98.91) | -1.41(-1.51 - -1.31) |
| Eastern Sub-Saharan Africa | 0.55(0.47-0.66) | 40.30(35.67-46.54) | 1.53(1.29-1.82) | 51.74(45.46-59.84) | 0.94(0.90 - 0.97) | 3.00(2.24-4.34) | 4.76(3.6-7.24) | 0.62(0.44-1.01) | 4.75(3.34-7.86) | 0.03(-0.01 - 0.08) | 0.98(0.74-1.27) | 106.02(81.48-150.25) | 1.79(1.29-2.7) | 95.96(69.09-151.07) | -0.32(-0.37 - -0.28) |
| High-income Asia Pacific | 24.21(20.68-28.39) | 1238.95(1061.77-1449.95) | 42.61(36.21-50.25) | 1426.23(1211.62-1670.16) | 0.72(0.58 - 0.86) | 3.91(3.50-4.51) | 2.33(2.05-2.68) | 1.07(0.77-1.28) | 1.65(1.22-1.94) | -1.00(-1.21 - -0.79) | 2.27(1.66-3.04) | 117.3(86.57-156.11) | 3.29(2.47-4.37) | 94.2(66.65-130.41) | -0.80(-0.91 - -0.69) |
| High-income North America | 34.45(28.94-40.66) | 1085.41(913.37-1285.16) | 39.76(34.03-46.30) | 833.93(713.75-978.73) | 0.45(-0.03 - 0.94) | 4.76(4.15-5.43) | 1.29(1.13-1.48) | 0.77(0.64-0.88) | 1.12(0.95-1.28) | -0.33(-0.41 - -0.24) | 2.70(1.96-3.69) | 82.61(59.04-112.85) | 3.40(2.60-4.40) | 66.75(49.67-88.57) | 0.19(-0.12 - 0.49) |
| North Africa and Middle East | 5.96(5.15-6.96) | 249.39(217.64-290.44) | 16.10(13.83-18.97) | 291.01(251.96-340.51) | 0.75(0.55 - 0.94) | 3.23(2.57-4.07) | 2.31(1.84-3.1) | 0.65(0.52-0.8) | 1.88(1.52-2.28) | -0.46(-0.60 - -0.32) | 1.53(1.23-1.88) | 76.13(62.18-93.43) | 2.80(2.15-3.56) | 58.57(46.20-73.01) | -0.74(-0.8 - -0.67) |
| Oceania | 0.06(0.05-0.07) | 132.46(116.35-154.92) | 0.15(0.13-0.18) | 142.32(124.13-166.24) | 0.26(0.24 - 0.29) | 0.06(0.04-0.10) | 2.40(1.58-3.72) | 0.02(0.01-0.02) | 2.47(1.81-3.85) | 0.26(0.19 - 0.33) | 0.03(0.02-0.04) | 78.91(54.15-114.34) | 0.07(0.05-0.10) | 79.82(59.24-114.71) | 0.17(0.11 - 0.23) |
| South Asia | 15.29(12.84-17.97) | 176.66(152.09-207.13) | 55.32(46.74-65.40) | 330.26(280.99-388.41) | 2.82(2.38 - 3.27) | 7.93(5.52-10.29) | 1.66(1.17-2.16) | 1.00(0.77-1.39) | 0.84(0.67-1.16) | -2.45(-2.60 - -2.29) | 4.92(3.67-6.29) | 65.85(49.54-83.38) | 8.50(6.16-11.46) | 53.49(39.35-71.73) | -0.43(-0.74 - -0.11) |
| Southeast Asia | 8.36(7.20-9.80) | 226.23(197.01-264.30) | 18.47(15.77-21.79) | 264.47(227.71-309.85) | 0.58(0.47 - 0.68) | 7.82(5.74-9.66) | 3.69(2.78-4.57) | 1.36(1.03-1.57) | 2.82(2.17-3.27) | -1.03(-1.08 - -0.98) | 3.16(2.41-3.81) | 104.65(81.05-124.92) | 4.60(3.59-5.61) | 74.35(58.97-88.94) | -1.29(-1.35 - -1.24) |
| Southern Latin America | 0.87(0.75-1.01) | 183.55(159.69-213.66) | 1.37(1.19-1.63) | 180.13(156.69-214.88) | -1.06(-1.43 - -0.68) | 1.78(1.45-1.93) | 4.13(3.41-4.49) | 0.21(0.18-0.27) | 2.48(2.07-3.22) | -1.58(-1.93 - -1.22) | 0.46(0.37-0.52) | 100.38(81.12-113.12) | 0.48(0.41-0.59) | 59.58(49.91-72.5) | -1.89(-2.20 - -1.58) |
| Southern Sub-Saharan Africa | 0.43(0.36-0.51) | 102.42(87.51-121.32) | 0.80(0.68-0.96) | 109.99(94.01-129.39) | 0.37(0.32 - 0.41) | 0.61(0.49-0.88) | 2.34(1.87-3.29) | 0.13(0.1-0.14) | 2.52(2.04-2.87) | 0.42(0.14 - 0.71) | 0.23(0.18-0.32) | 67.21(54.30-92.23) | 0.41(0.34-0.47) | 64.87(54.57-74.29) | 0.10(-0.20 - 0.40) |
| Tropical Latin America | 8.15(6.92-9.61) | 671.30(574.69-783.03) | 25.40(21.31-30.09) | 1041.01(872.81-1229.28) | 1.77(1.42 - 2.13) | 2.52(2.29-3.18) | 3.23(2.87-4.01) | 0.72(0.58-0.8) | 3.14(2.52-3.48) | 0.14(0.06 - 0.23) | 1.46(1.14-1.88) | 134.58(108.07-168.77) | 3.47(2.72-4.46) | 141.50(111.21-180.71) | 0.18(0.07 - 0.28) |
| Western Europe | 35.22(30.42-41.32) | 724.65(624.68-851.88) | 52.14(44.56-61.23) | 825.76(703.33-973.69) | 0.27(0.12 - 0.42) | 10.92(9.61-13.06) | 1.86(1.63-2.22) | 1.77(1.41-2.00) | 1.53(1.23-1.72) | -0.26(-0.47 - -0.05) | 4.58(3.49-5.95) | 88.30(66.06-116.44) | 5.78(4.44-7.54) | 79.98(58.33-107.88) | -0.71(-0.85 - -0.56) |
| Western Sub-Saharan Africa | 0.68(0.56-0.81) | 42.78(36.79-49.74) | 1.91(1.59-2.28) | 52.30(45.09-60.88) | 0.69(0.67 - 0.71) | 2.18(1.50-2.68) | 2.62(1.94-3.32) | 0.51(0.32-0.73) | 3.04(1.97-4.60) | 0.83(0.66 - 1.00) | 0.88(0.57-1.12) | 63.79(46.49-77.96) | 1.87(1.23-2.51) | 66.85(44.83-92.63) | 0.39(0.27 - 0.51) |
| GABD: Gallbladder and biliary diseases; ASR, age- standardised incidence rate; EAPC, estimated annual percentage change; UI, uncertainty interval. | | | | | | | | | | | | | | | |
