# Supplementary material for: Global, regional, and national burden of digestive diseases: findings from the global burden of disease study 2019
Source: Front Public Health. 2023 Aug 24;11:1202980. doi: 10.3389/fpubh.2023.1202980 (PMC10483149; doi:10.3389/fpubh.2023.1202980)
Supplement: Supplementary file 9 [file Table_9.docx]

| Table S9. The Incidence, Death, and DALYs of PACA in 1990 and 2019 | | | | | | | | | | | | | | | |
| --- | --- | --- | --- | --- | --- | --- | --- | --- | --- | --- | --- | --- | --- | --- | --- |
| Characteristics | 1990 | | 2019 | | 1990-2019 | 1990 | | 2019 | | 1990-2019 | 1990 | | 2019 | | 1990-2019 |
|  | Incidence cases  No×10^5^ (95%UI) | ASR per 100 000  No (95% UI) | Incidence cases  No×10^5^ (95%UI) | ASR per 100 000  No (95% UI) | EAPC  No (95% CI) | Death cases  No×10^3^ (95%UI) | ASR per 100 000  No (95% UI) | Death cases  No×10^3^ (95%UI) | ASR per 100 000  No (95% UI) | EAPC  No (95% CI) | DALYs  No×10^5^ (95%UI) | ASR per 100 000  No×10^2^ (95% UI) | DALYs  No×10^5^ (95%UI) | ASR per 100 000  No×10^2^ (95% UI) | EAPC  No (95% CI) |
| Global | 17.28(14.52-20.60) | 37.95(31.96-44.55) | 28.15(24.14-32.94) | 34.76(29.8-40.74) | -0.34(-0.41 - -0.27) | 69.82(62.05-82.53) | 1.72(1.53-2.02) | 115.05(104.3-128.17) | 1.43(1.30-1.59) | -0.69(-0.78 - -0.60) | 24.38(21.8-28.85) | 0.54(0.48-0.63) | 36.41(32.83-40.27) | 0.44(0.40-0.49) | -0.74(-0.83 - -0.64) |
| Sex |  |  |  |  |  |  |  |  |  |  |  |  |  |  |  |
| Female | 7.99(6.78-9.44) | 34.47(29.15-40.29) | 12.74(10.98-14.79) | 30.61(26.36-35.62) | -0.45(-0.52 - -0.39) | 26.69(23.53-32.95) | 1.27(1.12-1.55) | 43.07(36.59-50.77) | 1.00(0.84-1.17) | -0.89(-0.98 - -0.80) | 8.05(6.92-10.39) | 0.35(0.31-0.45) | 11.39(9.7-13.34) | 0.27(0.23-0.32) | -1.00(-1.08 - -0.91) |
| Male | 9.29(7.75-11.16) | 41.03(34.28-48.51) | 15.41(13.07-18.14) | 38.81(33.13-45.45) | -0.22(-0.29 - -0.16) | 43.13(37.62-51.56) | 2.19(1.91-2.61) | 71.98(63.88-81.42) | 1.88(1.67-2.12) | -0.57(-0.66 - -0.47) | 16.33(14.27-19.43) | 0.72(0.63-0.86) | 25.02(22.25-28.42) | 0.62(0.55-0.70) | -0.60(-0.70 - -0.50) |
| SDI |  |  |  |  |  |  |  |  |  |  |  |  |  |  |  |
| Low SDI | 1.02(0.83-1.23) | 28.41(23.80-33.69) | 2.24(1.84-2.71) | 27.72(23.38-32.94) | 0.14(0.08 - 0.19) | 6.39(4.65-8.61) | 2.41(1.70-3.29) | 12.23(9.72-15.56) | 2.09(1.67-2.68) | -0.47(-0.52 - -0.42) | 2.44(1.85-3.31) | 0.75(0.55-1.01) | 4.59(3.67-5.84) | 0.64(0.51-0.81) | -0.53(-0.57 - -0.49) |
| Low-middle SDI | 2.97(2.45-3.62) | 34.92(29.04-41.83) | 5.99(4.97-7.21) | 36.53(30.71-43.73) | 0.23(0.20 - 0.27) | 14.31(11.86-18.51) | 2.17(1.76-2.78) | 26.44(21.69-30.71) | 1.87(1.54-2.17) | -0.43(-0.49 - -0.36) | 5.50(4.62-7.27) | 0.68(0.57-0.88) | 9.19(7.51-10.66) | 0.58(0.47-0.67) | -0.51(-0.56 - -0.46) |
| Middle SDI | 4.55(3.73-5.54) | 33.67(27.83-40.08) | 7.35(6.16-8.67) | 28.84(24.38-34.15) | -0.35(-0.51 - -0.19) | 15.56(13.21-20.29) | 1.44(1.21-1.87) | 25.78(22.84-30.11) | 1.09(0.95-1.26) | -0.92(-0.96 - -0.87) | 5.81(4.98-7.39) | 0.44(0.37-0.56) | 8.13(7.27-9.61) | 0.32(0.28-0.37) | -1.11(-1.15 - -1.07) |
| High-middle SDI | 4.83(4.07-5.69) | 43.21(36.58-50.57) | 6.67(5.70-7.69) | 36.74(31.54-42.61) | -0.63(-0.70 - -0.56) | 21.37(19.82-25.09) | 2.02(1.87-2.37) | 34.39(31.19-37.31) | 1.79(1.62-1.94) | -0.57(-0.82 - -0.31) | 7.21(6.68-8.39) | 0.64(0.59-0.74) | 10.58(9.62-11.57) | 0.57(0.52-0.62) | -0.58(-0.87 - -0.30) |
| High SDI | 3.90(3.36-4.52) | 41.32(35.36-47.99) | 5.34(4.78-5.97) | 38.07(33.88-42.72) | -0.38(-0.44 - -0.31) | 12.16(11.44-13.39) | 1.20(1.13-1.33) | 16.16(14.6-18.48) | 0.88(0.81-0.99) | -1.24(-1.33 - -1.15) | 3.41(3.21-3.70) | 0.35(0.33-0.38) | 3.90(3.60-4.29) | 0.26(0.24-0.28) | -1.23(-1.30 - -1.15) |
| Region |  |  |  |  |  |  |  |  |  |  |  |  |  |  |  |
| Andean Latin America | 0.13(0.11-0.15) | 45.8(39.61-52.94) | 0.26(0.23-0.30) | 43.53(38.36-49.34) | -0.29(-0.33 - -0.24) | 1.08(0.81-1.30) | 4.55(3.43-5.57) | 1.44(1.12-1.97) | 2.54(1.97-3.48) | -2.23(-2.36 - -2.10) | 0.42(0.31-0.49) | 1.45(1.08-1.73) | 0.44(0.34-0.57) | 0.73(0.57-0.96) | -2.67(-2.82 - -2.52) |
| Australasia | 0.09(0.07-0.10) | 38.65(32.50-45.26) | 0.14(0.12-0.17) | 37.58(31.74-44.01) | -0.13(-0.16 - -0.11) | 0.22(0.20-0.23) | 0.95(0.87-1.02) | 0.34(0.29-0.40) | 0.67(0.58-0.77) | -1.27(-1.42 - -1.11) | 0.06(0.05-0.06) | 0.24(0.22-0.27) | 0.07(0.06-0.08) | 0.17(0.15-0.19) | -1.35(-1.53 - -1.17) |
| Caribbean | 0.09(0.07-0.11) | 28.84(23.93-34.30) | 0.14(0.12-0.17) | 28.36(23.55-33.61) | -0.03(-0.05 - -0.02) | 0.38(0.34-0.44) | 1.42(1.25-1.62) | 0.66(0.56-0.80) | 1.29(1.09-1.56) | -0.33(-0.41 - -0.25) | 0.13(0.12-0.16) | 0.45(0.39-0.52) | 0.20(0.17-0.25) | 0.40(0.33-0.49) | -0.35(-0.44 - -0.25) |
| Central Asia | 0.18(0.15-0.22) | 33.73(28.41-39.35) | 0.29(0.24-0.34) | 32.93(27.71-38.49) | -0.07(-0.09 - -0.05) | 1.18(1.02-1.32) | 2.42(2.06-2.75) | 1.65(1.38-1.88) | 2.12(1.75-2.41) | -0.89(-1.27 - -0.52) | 0.41(0.37-0.45) | 0.77(0.68-0.85) | 0.60(0.51-0.69) | 0.67(0.57-0.77) | -0.95(-1.35 - -0.56) |
| Central Europe | 0.67(0.57-0.78) | 49.4(42.00-57.28) | 0.73(0.65-0.82) | 45.17(40.08-50.95) | -0.41(-0.45 - -0.36) | 4.48(4.27-5.03) | 3.20(3.05-3.62) | 5.14(4.51-5.83) | 2.70(2.37-3.05) | -0.72(-0.89 - -0.55) | 1.48(1.41-1.61) | 1.06(1.01-1.16) | 1.41(1.23-1.59) | 0.84(0.74-0.96) | -1.00(-1.15 - -0.85) |
| Central Latin America | 0.47(0.39-0.56) | 37.56(32.12-44.03) | 0.97(0.83-1.13) | 38.55(33.34-44.74) | -0.05(-0.08 - -0.02) | 2.02(1.91-2.13) | 2.06(1.95-2.21) | 4.33(3.73-5.07) | 1.81(1.55-2.11) | -0.51(-0.63 - -0.39) | 0.8(0.74-0.84) | 0.67(0.63-0.71) | 1.42(1.22-1.65) | 0.57(0.49-0.66) | -0.67(-0.82 - -0.52) |
| Central Sub-Saharan Africa | 0.07(0.06-0.09) | 21.06(17.63-25.02) | 0.18(0.15-0.22) | 20.84(17.45-24.71) | -0.03(-0.04 - -0.02) | 0.45(0.30-0.76) | 1.78(1.15-3.13) | 0.91(0.53-1.65) | 1.43(0.83-2.68) | -0.76(-0.88 - -0.64) | 0.17(0.12-0.29) | 0.54(0.37-0.91) | 0.36(0.21-0.63) | 0.45(0.27-0.80) | -0.67(-0.80 - -0.54) |
| East Asia | 3.97(3.23-4.82) | 37.96(31.07-45.39) | 5.26(4.45-6.15) | 27.57(23.49-32.16) | -1.25(-1.56 - -0.95) | 9.32(7.50-12.42) | 1.08(0.87-1.45) | 11.29(8.80-13.45) | 0.60(0.47-0.71) | -1.96(-2.02 - -1.91) | 3.33(2.66-4.31) | 0.32(0.26-0.42) | 3.20(2.56-3.81) | 0.17(0.13-0.20) | -2.26(-2.30 - -2.23) |
| Eastern Europe | 1.83(1.55-2.12) | 71.24(60.79-82.93) | 2.22(1.88-2.58) | 79.59(68.16-92.54) | 0.38(0.31 - 0.45) | 7.66(6.92-10.79) | 2.9(2.62-4.07) | 15.58(13.37-17.73) | 5.31(4.54-6.04) | 1.91(1.39 - 2.43) | 2.83(2.55-3.83) | 1.09(0.98-1.47) | 5.58(4.81-6.40) | 2.07(1.78-2.38) | 1.92(1.33 - 2.50) |
| Eastern Sub-Saharan Africa | 0.25(0.21-0.31) | 21.13(17.66-25.22) | 0.58(0.47-0.71) | 21.14(17.67-25.22) | -0.03(-0.05 - -0.01) | 1.40(0.90-2.10) | 1.69(1.05-2.60) | 2.77(1.71-4.74) | 1.49(0.91-2.65) | -0.50(-0.59 - -0.42) | 0.52(0.35-0.78) | 0.50(0.32-0.75) | 1.03(0.65-1.71) | 0.43(0.27-0.74) | -0.52(-0.60 - -0.44) |
| High-income Asia Pacific | 0.63(0.52-0.74) | 32.86(27.56-39.15) | 0.79(0.69-0.90) | 31.46(27.29-36.52) | -0.28(-0.35 - -0.21) | 1.79(1.60-2.06) | 0.95(0.85-1.08) | 2.25(1.89-2.80) | 0.48(0.42-0.57) | -2.69(-2.83 - -2.56) | 0.54(0.48-0.66) | 0.28(0.24-0.33) | 0.48(0.42-0.57) | 0.15(0.13-0.17) | -2.49(-2.63 - -2.35) |
| High-income North America | 2.01(1.73-2.32) | 62.36(53.74-72.00) | 2.58(2.36-2.84) | 52.01(47.5-56.92) | -0.65(-0.82 - -0.48) | 3.35(3.12-3.58) | 0.97(0.91-1.04) | 5.44(4.99-5.96) | 0.92(0.85-1.00) | -0.34(-0.55 - -0.13) | 1.00(0.93-1.10) | 0.31(0.29-0.34) | 1.45(1.34-1.61) | 0.29(0.26-0.32) | -0.29(-0.45 - -0.13) |
| North Africa and Middle East | 0.62(0.51-0.75) | 26.69(22.29-31.53) | 1.41(1.17-1.68) | 26.64(22.51-31.16) | 0.02(0.00 - 0.04) | 1.72(1.39-2.35) | 1.13(0.87-1.53) | 3.39(2.65-4.07) | 0.89(0.68-1.06) | -0.69(-0.78 - -0.59) | 0.50(0.43-0.69) | 0.26(0.21-0.35) | 0.92(0.74-1.11) | 0.19(0.16-0.23) | -0.93(-1.01 - -0.85) |
| Oceania | 0.01(0.01-0.01) | 24.86(20.66-29.60) | 0.02(0.02-0.03) | 24.13(19.96-28.67) | -0.11(-0.11 - -0.10) | 0.04(0.03-0.05) | 1.05(0.73-1.52) | 0.08(0.05-0.11) | 0.93(0.66-1.31) | -0.45(-0.47 - -0.43) | 0.02(0.01-0.02) | 0.34(0.24-0.48) | 0.03(0.02-0.04) | 0.30(0.21-0.42) | -0.42(-0.44 - -0.39) |
| South Asia | 3.29(2.67-4.01) | 38.09(31.60-45.89) | 7.44(6.11-9.02) | 42.98(35.89-51.68) | 0.51(0.44 - 0.59) | 14.05(11.34-19.22) | 2.25(1.77-3.08) | 25.94(20.09-31.35) | 1.80(1.39-2.16) | -0.70(-0.85 - -0.55) | 5.48(4.48-7.60) | 0.70(0.56-0.95) | 9.10(7.08-10.94) | 0.55(0.43-0.66) | -0.74(-0.86 - -0.61) |
| Southeast Asia | 0.94(0.76-1.14) | 26.02(21.63-30.91) | 1.74(1.44-2.09) | 25.33(21.2-30.08) | -0.10(-0.11 - -0.09) | 4.99(3.84-7.46) | 1.79(1.38-2.56) | 7.91(6.54-11.17) | 1.34(1.12-1.83) | -1.11(-1.16 - -1.06) | 1.88(1.45-2.87) | 0.55(0.42-0.81) | 2.55(2.05-3.77) | 0.38(0.31-0.55) | -1.41(-1.47 - -1.34) |
| Southern Latin America | 0.16(0.14-0.18) | 33.56(28.94-38.56) | 0.24(0.21-0.28) | 31.60(27.36-36.57) | -0.32(-0.36 - -0.29) | 1.27(1.14-1.37) | 2.77(2.47-3.00) | 1.50(1.35-1.73) | 1.83(1.65-2.12) | -1.74(-1.96 - -1.52) | 0.37(0.34-0.40) | 0.79(0.72-0.85) | 0.40(0.37-0.46) | 0.52(0.47-0.59) | -1.72(-1.94 - -1.50) |
| Southern Sub-Saharan Africa | 0.09(0.07-0.11) | 22.13(18.46-26.45) | 0.15(0.13-0.19) | 21.71(18.07-25.94) | -0.10(-0.14 - -0.06) | 0.33(0.26-0.41) | 1.01(0.81-1.30) | 0.57(0.45-0.68) | 0.93(0.75-1.08) | -0.61(-0.97 - -0.24) | 0.13(0.10-0.16) | 0.35(0.28-0.44) | 0.21(0.17-0.25) | 0.30(0.24-0.36) | -0.87(-1.25 - -0.49) |
| Tropical Latin America | 0.24(0.21-0.27) | 20.06(17.68-22.74) | 0.48(0.42-0.54) | 19.38(17.11-21.89) | -0.12(-0.17 - -0.06) | 2.33(2.21-2.49) | 2.27(2.13-2.43) | 5.56(4.79-5.99) | 2.29(1.98-2.48) | 0.35(0.22 - 0.47) | 0.90(0.85-0.95) | 0.75(0.71-0.80) | 1.76(1.56-1.89) | 0.71(0.62-0.76) | -0.01(-0.11 - 0.10) |
| Western Europe | 1.23(1.08-1.40) | 25.44(22.12-29.23) | 1.70(1.49-1.95) | 26.32(22.81-30.07) | -0.05(-0.14 - 0.05) | 7.99(7.45-8.87) | 1.45(1.35-1.59) | 9.98(8.93-11.46) | 1.07(0.98-1.23) | -1.19(-1.26 - -1.11) | 1.96(1.85-2.13) | 0.39(0.37-0.42) | 2.00(1.84-2.24) | 0.27(0.25-0.30) | -1.41(-1.47 - -1.35) |
| Western Sub-Saharan Africa | 0.33(0.28-0.40) | 25.98(22.04-30.59) | 0.82(0.68-0.99) | 26.75(22.71-31.37) | 0.10(0.09 - 0.11) | 3.78(2.50-5.77) | 3.77(2.51-5.82) | 8.31(5.93-11.88) | 3.62(2.66-5.10) | -0.07(-0.13 - -0.01) | 1.44(0.95-2.14) | 1.23(0.81-1.89) | 3.20(2.27-4.59) | 1.16(0.82-1.66) | -0.15(-0.22 - -0.08) |
| PACA:Pancreatitis; ASR, age- standardised incidence rate; EAPC, estimated annual percentage change; UI, uncertainty interval. | | | | | | | | | | | | | | | |
