# Supplementary material for: Global, regional, and national burden of digestive diseases: findings from the global burden of disease study 2019
Source: Front Public Health. 2023 Aug 24;11:1202980. doi: 10.3389/fpubh.2023.1202980 (PMC10483149; doi:10.3389/fpubh.2023.1202980)
Supplement: Supplementary file 10 [file Table_10.docx]

| Table S10. The change of digestive diseases between 1990 and 2019 at national level, both sexes | | | | | | | | | | | | | |
| --- | --- | --- | --- | --- | --- | --- | --- | --- | --- | --- | --- | --- | --- |
| Region | Cause | Incidence | | | | Death | | | | DALYs | | | |
|  |  | Cases in 1990 | Cases in 2019 | Change in absolute number | EAPC No (95% CI) | Cases in 1990 | Cases in 2019 | Change in absolute number | EAPC No (95% CI) | Cases in 1990 | Cases in 2019 | Change in absolute number | EAPC No (95% CI) |
| Afghanistan | Digestive diseases | 483721.53 | 1552355.24 | 220.92% | 0.06(0.05-0.07) | 5004.65 | 6928.92 | 38.45% | -1.18(-1.35 - -1) | 189886.17 | 319384.9 | 68.20% | -1.26(-1.44 - -1.07) |
| Afghanistan | APED | 26137.5 | 104470.67 | 299.70% | 0.46(0.32-0.6) | 228.41 | 276.69 | 21.14% | -2.25(-2.57 - -1.94) | 10785.69 | 14640.08 | 35.74% | -2.55(-2.88 - -2.22) |
| Afghanistan | COCLD | 1883.56 | 6354.88 | 237.39% | 0.41(0.32-0.5) | 2673.46 | 3797.37 | 42.04% | -1.17(-1.33 - -1.02) | 91450.46 | 145957.45 | 59.60% | -1.33(-1.51 - -1.16) |
| Afghanistan | GABD | 13731.84 | 48432.29 | 252.70% | 0.86(0.8-0.92) | 165.79 | 291.07 | 75.57% | 0.27(0.15-0.39) | 7501.49 | 15882.6 | 111.73% | -0.17(-0.3 - -0.03) |
| Afghanistan | IFAH | 4898.06 | 19282.24 | 293.67% | 1.07(0.87-1.27) | 45.27 | 62.46 | 37.97% | -1.16(-1.34 - -0.99) | 3596.58 | 9063.64 | 152.01% | -0.29(-0.41 - -0.17) |
| Afghanistan | IFBD | 191.77 | 684.11 | 256.73% | 0.28(0.16-0.4) | 30.66 | 57.75 | 88.36% | 0.01(-0.06-0.08) | 1237.06 | 3017.26 | 143.91% | 0.03(-0.06-0.12) |
| Afghanistan | PACA | 2256.9 | 6202.59 | 174.83% | 0.05(0.04-0.06) | 114.12 | 184.95 | 62.07% | -0.5(-0.6 - -0.39) | 3265.56 | 6275.38 | 92.17% | -0.67(-0.78 - -0.57) |
| Afghanistan | PIIO | 3514.85 | 10354.02 | 194.58% | 0.42(0.22-0.62) | 376.13 | 648.45 | 72.40% | -0.26(-0.39 - -0.12) | 20905.79 | 38243.08 | 82.93% | -0.7(-0.89 - -0.52) |
| Afghanistan | UDSD | 430634.99 | 1355079.14 | 214.67% | 0.002(-0.003-0.006) | 1237.38 | 1348.89 | 9.01% | -1.88(-2.15 - -1.6) | 47066.81 | 76656.56 | 62.87% | -1.59(-1.83 - -1.35) |
| Afghanistan | VAID | 472.07 | 1495.3 | 216.75% | 0.93(0.66-1.19) | 56.27 | 80.43 | 42.94% | -0.35(-0.41 - -0.3) | 1257.54 | 1974.72 | 57.03% | -0.42(-0.47 - -0.37) |
| Albania | Digestive diseases | 130144.98 | 160189.3 | 23.09% | 0.16(0.14-0.18) | 647.95 | 527.36 | -18.61% | -2.77(-3.17 - -2.36) | 32407.05 | 19444.51 | -40.00% | -2.46(-2.88 - -2.05) |
| Albania | APED | 7461.03 | 5263.42 | -29.45% | 0.17(0.13-0.21) | 6.68 | 3.69 | -44.76% | -3.43(-4.03 - -2.83) | 374.93 | 152.49 | -59.33% | -2.27(-2.8 - -1.74) |
| Albania | COCLD | 710.76 | 640.68 | -9.86% | -0.52(-0.68 - -0.37) | 326.44 | 332.7 | 1.92% | -2.09(-2.44 - -1.73) | 10094.09 | 8199.28 | -18.77% | -1.87(-2.26 - -1.48) |
| Albania | GABD | 22122.92 | 32860.86 | 48.54% | 0.63(0.6-0.66) | 14.66 | 21.49 | 46.59% | -1.4(-1.62 - -1.19) | 2684.89 | 3107.82 | 15.75% | -0.61(-0.69 - -0.53) |
| Albania | IFAH | 4108.5 | 5120.28 | 24.63% | 0.72(0.59-0.85) | 47.95 | 14.65 | -69.45% | -5.13(-5.75 - -4.51) | 4188.85 | 1249.96 | -70.16% | -3.84(-4.42 - -3.26) |
| Albania | IFBD | 245.93 | 290.6 | 18.16% | 0.68(0.61-0.76) | 49.74 | 12.58 | -74.71% | -5.17(-6.14 - -4.19) | 3904.01 | 990.88 | -74.62% | -4.07(-5.01 - -3.12) |
| Albania | PACA | 1084.58 | 1354.32 | 24.87% | -0.23(-0.28 - -0.19) | 21.93 | 28.47 | 29.82% | -1.61(-1.84 - -1.39) | 745.14 | 755.68 | 1.41% | -1.53(-1.76 - -1.3) |
| Albania | PIIO | 2109.72 | 3699.44 | 75.35% | 0.82(0.73-0.92) | 77.88 | 31.62 | -59.41% | -4.14(-4.56 - -3.71) | 5519.01 | 787.35 | -85.73% | -6.41(-7.06 - -5.76) |
| Albania | UDSD | 92029.12 | 110540.56 | 20.11% | -0.008(-0.025-0.01) | 62.45 | 38.51 | -38.33% | -4.6(-5.01 - -4.18) | 3642.37 | 3272.59 | -10.15% | -1.51(-1.74 - -1.27) |
| Albania | VAID | 272.4 | 419.14 | 53.87% | 1.26(1.15-1.36) | 11.38 | 24.53 | 115.55% | -0.17(-0.3 - -0.04) | 260.56 | 413.1 | 58.54% | -0.35(-0.54 - -0.15) |
| Algeria | Digestive diseases | 1073496.11 | 2420784.87 | 125.50% | 0.08(0.08-0.09) | 3980.58 | 6398.22 | 60.74% | -1.86(-1.93 - -1.8) | 165865.84 | 233281.28 | 40.64% | -1.69(-1.75 - -1.64) |
| Algeria | APED | 58397.14 | 120880.15 | 107.00% | 0.91(0.84-0.99) | 109.78 | 87.96 | -19.88% | -3.56(-3.64 - -3.47) | 5844.73 | 4158.74 | -28.85% | -3.16(-3.28 - -3.05) |
| Algeria | COCLD | 3379.83 | 7823.41 | 131.47% | -0.18(-0.31 - -0.04) | 2401.04 | 4044.9 | 68.46% | -1.73(-1.8 - -1.66) | 81293.57 | 110539.79 | 35.98% | -1.95(-2.02 - -1.88) |
| Algeria | GABD | 36609.86 | 97660.28 | 166.76% | 0.31(0.3-0.32) | 138.44 | 325.86 | 135.38% | -0.72(-0.82 - -0.62) | 7738.28 | 14952.6 | 93.23% | -0.93(-0.98 - -0.87) |
| Algeria | IFAH | 12744.14 | 29378.16 | 130.52% | 1.01(0.97-1.05) | 26.24 | 44.94 | 71.27% | -1.74(-1.85 - -1.63) | 4345.5 | 7124.88 | 63.96% | -0.3(-0.36 - -0.25) |
| Algeria | IFBD | 576.85 | 1657.35 | 187.31% | 0.95(0.86-1.04) | 27.63 | 52.53 | 90.12% | -1.14(-1.23 - -1.04) | 1826.27 | 4021.08 | 120.18% | -0.28(-0.38 - -0.17) |
| Algeria | PACA | 4471.81 | 10190.77 | 127.89% | -0.12(-0.13 - -0.11) | 100.06 | 207.81 | 107.69% | -1.03(-1.09 - -0.97) | 3087.71 | 5359.02 | 73.56% | -1.31(-1.37 - -1.25) |
| Algeria | PIIO | 16927.72 | 43221.26 | 155.33% | 1.01(0.98-1.04) | 309.92 | 454.03 | 46.50% | -1.36(-1.44 - -1.29) | 16797.07 | 14542.47 | -13.42% | -1.81(-1.88 - -1.73) |
| Algeria | UDSD | 938966.51 | 2105403.67 | 124.23% | 0.005(0.003-0.007) | 725.5 | 792.38 | 9.22% | -3.32(-3.44 - -3.21) | 40621.07 | 63186.87 | 55.55% | -1.68(-1.77 - -1.59) |
| Algeria | VAID | 1422.23 | 4569.83 | 221.31% | 1.13(1.05-1.2) | 78.1 | 190.27 | 143.62% | -0.71(-0.79 - -0.62) | 1686.31 | 3670.23 | 117.65% | -0.8(-0.89 - -0.72) |
| American Samoa | Digestive diseases | 1165.64 | 1646.91 | 41.29% | 0.02(0.01-0.03) | 8.99 | 13.6 | 51.27% | -0.93(-1.09 - -0.78) | 356.38 | 464.6 | 30.37% | -0.81(-0.96 - -0.67) |
| American Samoa | APED | 51.26 | 67.21 | 31.12% | 0.42(0.35-0.48) | 0.11 | 0.12 | 9.09% | -1.36(-1.78 - -0.93) | 5.02 | 4.77 | -4.98% | -1.09(-1.5 - -0.69) |
| American Samoa | COCLD | 6.32 | 7.33 | 15.98% | -0.36(-0.43 - -0.29) | 4.81 | 6.95 | 44.49% | -0.92(-1.09 - -0.76) | 182.3 | 232.6 | 27.59% | -0.92(-1.1 - -0.75) |
| American Samoa | GABD | 59.43 | 98.42 | 65.61% | 0.49(0.43-0.55) | 0.55 | 1.27 | 130.91% | 0.78(0.38-1.18) | 21.87 | 39.63 | 81.21% | 0.64(0.32-0.95) |
| American Samoa | IFAH | 37.04 | 66.45 | 79.40% | 0.9(0.61-1.18) | 0.22 | 0.4 | 81.82% | -0.82(-1.71-0.08) | 13.88 | 22.48 | 61.96% | -0.04(-0.59-0.51) |
| American Samoa | IFBD | 0.19 | 0.34 | 78.95% | 1.1(0.97-1.22) | 0.38 | 0.24 | -36.84% | -4.4(-4.8 - -4.01) | 14.77 | 7.77 | -47.39% | -4.23(-4.6 - -3.86) |
| American Samoa | PACA | 9.38 | 14.16 | 50.96% | 0.04(0.02-0.05) | 0.38 | 0.76 | 100.00% | 0.42(0.16-0.69) | 14.56 | 26.46 | 81.73% | 0.77(0.47-1.07) |
| American Samoa | PIIO | 31.98 | 43.26 | 35.24% | 0.21(0.13-0.29) | 0.41 | 0.89 | 118.67% | 0.46(0.28-0.64) | 14.82 | 21.55 | 45.39% | 0.24(0.09-0.38) |
| American Samoa | UDSD | 967.74 | 1345.82 | 39.07% | -0.074(-0.087 - -0.061) | 1.79 | 1.98 | 10.61% | -2.7(-2.9 - -2.5) | 77.78 | 82.29 | 5.80% | -1.92(-2.08 - -1.76) |
| American Samoa | VAID | 2.29 | 3.91 | 70.74% | 0.41(0.37-0.44) | 0.12 | 0.27 | 125.00% | 0.06(-0.03-0.15) | 2.88 | 5.9 | 104.86% | 0.18(0.05-0.31) |
| Andorra | Digestive diseases | 2715.19 | 4998.5 | 84.09% | 0.06(0.05-0.07) | 10.41 | 26.58 | 155.36% | -0.71(-0.82 - -0.61) | 365.24 | 705.48 | 93.16% | -0.62(-0.68 - -0.57) |
| Andorra | APED | 143.13 | 186.77 | 30.49% | 0.16(0.13-0.19) | 0.11 | 0.2 | 81.82% | -2.12(-2.32 - -1.93) | 4.36 | 5.59 | 28.21% | -1.16(-1.28 - -1.03) |
| Andorra | COCLD | 14.68 | 22.29 | 51.84% | -0.58(-0.65 - -0.51) | 6.18 | 12.17 | 96.93% | -1.01(-1.09 - -0.93) | 185.94 | 325.18 | 74.88% | -0.99(-1.04 - -0.94) |
| Andorra | GABD | 231.71 | 501.45 | 116.41% | 0.19(0.17-0.21) | 0.51 | 1.99 | 290.20% | -0.27(-0.42 - -0.12) | 26.1 | 58.23 | 123.10% | -0.27(-0.34 - -0.2) |
| Andorra | IFAH | 65.86 | 134 | 103.46% | 0.13(0.05-0.21) | 0.27 | 0.83 | 207.41% | -1.1(-1.35 - -0.84) | 16.45 | 31.65 | 92.40% | -0.55(-0.63 - -0.47) |
| Andorra | IFBD | 5.44 | 9.47 | 74.08% | 0.2(0.09-0.32) | 0.29 | 1.06 | 265.52% | 0.03(-0.06-0.13) | 17.21 | 36.38 | 111.39% | 0.02(-0.03-0.07) |
| Andorra | PACA | 13.2 | 24.72 | 87.27% | -0.23(-0.26 - -0.21) | 0.56 | 1.35 | 141.07% | -0.63(-0.69 - -0.58) | 16.68 | 31.93 | 91.43% | -0.59(-0.63 - -0.56) |
| Andorra | PIIO | 69.05 | 166.52 | 141.17% | 0.26(0.13-0.4) | 0.7 | 2.85 | 307.25% | -0.34(-0.49 - -0.2) | 14.18 | 37.53 | 164.72% | -0.43(-0.51 - -0.34) |
| Andorra | UDSD | 2159.7 | 3919.2 | 81.47% | 0.029(0.026-0.032) | 0.61 | 1.06 | 73.77% | -2.61(-2.84 - -2.38) | 59.91 | 104.58 | 74.56% | -0.43(-0.49 - -0.38) |
| Andorra | VAID | 12.42 | 34.07 | 174.32% | 0.46(0.34-0.57) | 0.76 | 2.97 | 290.79% | 0.3(0.23-0.37) | 15.12 | 44.41 | 193.72% | 0.2(0.15-0.25) |
| Angola | Digestive diseases | 381919.59 | 1176705.25 | 208.10% | 0.2(0.19-0.21) | 4480.92 | 8971.25 | 100.21% | -1(-1.07 - -0.93) | 194855.47 | 380612.93 | 95.33% | -1.14(-1.22 - -1.06) |
| Angola | APED | 22149.82 | 91754.79 | 314.25% | 1.31(1.21-1.42) | 115.59 | 142.48 | 23.26% | -2.36(-2.48 - -2.23) | 7336.82 | 8990.43 | 22.54% | -2.55(-2.72 - -2.38) |
| Angola | COCLD | 1629.08 | 6052.1 | 271.50% | 0.87(0.67-1.06) | 2782.22 | 5665.58 | 103.64% | -1.13(-1.2 - -1.06) | 104193.92 | 209949.92 | 101.50% | -1.22(-1.31 - -1.14) |
| Angola | GABD | 2593.15 | 9966.57 | 284.34% | 1.25(1.21-1.3) | 129 | 359.71 | 178.84% | 0.35(0.27-0.44) | 4779.48 | 11642.78 | 143.60% | -0.11(-0.19 - -0.03) |
| Angola | IFAH | 10237.83 | 33430.28 | 226.54% | 0.92(0.79-1.05) | 114.1 | 181.8 | 59.33% | -0.75(-0.82 - -0.68) | 10326.74 | 17836.35 | 72.72% | -0.88(-0.91 - -0.85) |
| Angola | IFBD | 74.67 | 263.45 | 252.82% | 0.71(0.67-0.76) | 36.94 | 78.41 | 112.26% | -0.6(-0.66 - -0.54) | 1912.63 | 3661.84 | 91.46% | -0.89(-0.95 - -0.83) |
| Angola | PACA | 1359.89 | 3959.29 | 191.15% | -0.02(-0.03 - -0.01) | 81.16 | 196.51 | 142.13% | -0.46(-0.62 - -0.29) | 3219.12 | 7712.96 | 139.60% | -0.55(-0.74 - -0.37) |
| Angola | PIIO | 8206.61 | 31624.93 | 285.36% | 1.15(1.05-1.26) | 399.66 | 899.37 | 125.03% | -0.55(-0.68 - -0.43) | 21214.07 | 44592.52 | 110.20% | -0.64(-0.78 - -0.5) |
| Angola | UDSD | 335183.96 | 997692.48 | 197.66% | 0.096(0.092-0.099) | 559.28 | 855.72 | 53.00% | -1.51(-1.59 - -1.44) | 30619.43 | 54976.86 | 79.55% | -1.38(-1.45 - -1.31) |
| Angola | VAID | 484.58 | 1961.38 | 304.76% | 1.46(1.27-1.65) | 43.83 | 136.12 | 210.56% | 0.67(0.47-0.86) | 1332.25 | 3631.61 | 172.59% | 0.34(0.14-0.54) |
| Antigua and Barbuda | Digestive diseases | 4176.95 | 7610.08 | 82.19% | 0.1(0.09-0.1) | 16.67 | 23.55 | 41.26% | -0.94(-1.19 - -0.69) | 562.76 | 845.09 | 50.17% | -0.91(-1.08 - -0.74) |
| Antigua and Barbuda | APED | 148.79 | 268.72 | 80.60% | 1.04(1-1.09) | 0.35 | 0.38 | 8.57% | -0.99(-1.54 - -0.43) | 14.04 | 15.44 | 9.97% | -0.86(-1.26 - -0.46) |
| Antigua and Barbuda | COCLD | 9.56 | 19.24 | 101.26% | -0.19(-0.26 - -0.12) | 9.06 | 12.89 | 42.27% | -1.28(-1.57 - -1) | 244.93 | 349.16 | 42.56% | -1.55(-1.83 - -1.28) |
| Antigua and Barbuda | GABD | 182.86 | 338.33 | 85.02% | 0.08(0.03-0.13) | 1.07 | 1.2 | 12.15% | -1.24(-1.66 - -0.82) | 42.56 | 58.13 | 36.58% | -1.05(-1.26 - -0.83) |
| Antigua and Barbuda | IFAH | 113.47 | 225.54 | 98.77% | 0.79(0.72-0.86) | 0.27 | 0.4 | 48.15% | -0.1(-0.44-0.25) | 33.04 | 55.07 | 66.68% | 0.18(0.09-0.26) |
| Antigua and Barbuda | IFBD | 1.16 | 2.68 | 131.03% | 0.56(0.42-0.71) | 0.53 | 0.63 | 18.87% | -1.77(-2.08 - -1.46) | 17.55 | 21.95 | 25.07% | -1.66(-1.9 - -1.42) |
| Antigua and Barbuda | PACA | 15.66 | 26.98 | 72.29% | -0.08(-0.09 - -0.07) | 0.53 | 0.9 | 69.81% | -0.27(-0.47 - -0.07) | 16.6 | 28.05 | 68.98% | -0.46(-0.59 - -0.33) |
| Antigua and Barbuda | PIIO | 61.39 | 114.88 | 87.13% | 0.74(0.7-0.79) | 1.51 | 2.6 | 72.32% | 0.64(0.48-0.79) | 36.55 | 59.29 | 62.21% | 0.4(0.27-0.53) |
| Antigua and Barbuda | UDSD | 3639.3 | 6603.84 | 81.46% | 0.024(0.02-0.028) | 2.28 | 2.1 | -7.89% | -2.32(-2.59 - -2.04) | 133.25 | 203.31 | 52.58% | -0.8(-0.9 - -0.71) |
| Antigua and Barbuda | VAID | 4.75 | 9.89 | 108.21% | 0.7(0.65-0.76) | 0.32 | 0.46 | 43.75% | -0.39(-0.69 - -0.1) | 5.88 | 8.96 | 52.38% | -0.71(-0.99 - -0.43) |
| Argentina | Digestive diseases | 1911609.57 | 2971173.97 | 55.43% | -0.08(-0.15 - -0.02) | 12139.5 | 17791.96 | 46.56% | -0.47(-0.64 - -0.31) | 373939.29 | 486287.44 | 30.04% | -0.63(-0.79 - -0.46) |
| Argentina | APED | 66689.25 | 131161.44 | 96.68% | 1.32(1.27-1.38) | 177.66 | 147.52 | -16.96% | -2.03(-2.78 - -1.27) | 6458.38 | 5525.06 | -14.45% | -1.43(-2.05 - -0.82) |
| Argentina | COCLD | 6741.42 | 13006.39 | 92.93% | 0.77(0.73-0.8) | 5942.63 | 8524.54 | 43.45% | -0.34(-0.52 - -0.16) | 175176.05 | 222884.52 | 27.23% | -0.6(-0.82 - -0.38) |
| Argentina | GABD | 57463.99 | 88714.55 | 54.38% | -1.39(-1.91 - -0.86) | 1183.76 | 1396.19 | 17.95% | -1.23(-1.63 - -0.83) | 31017.28 | 32715.78 | 5.48% | -1.65(-1.98 - -1.32) |
| Argentina | IFAH | 37230.37 | 72173.95 | 93.86% | 0.99(0.93-1.06) | 210.39 | 314.73 | 49.59% | -0.38(-0.78-0.02) | 16403.56 | 20917.91 | 27.52% | -0.31(-0.52 - -0.1) |
| Argentina | IFBD | 524.4 | 830.95 | 58.46% | 0.13(0.1-0.15) | 83.59 | 143.35 | 71.49% | -0.1(-0.25-0.04) | 2980.58 | 4555.57 | 52.84% | -0.13(-0.22 - -0.05) |
| Argentina | PACA | 11039.26 | 15524.73 | 40.63% | -0.43(-0.46 - -0.4) | 962.48 | 1041.54 | 8.21% | -1.94(-2.23 - -1.65) | 27402.61 | 28615.55 | 4.43% | -1.8(-2.07 - -1.54) |
| Argentina | PIIO | 26941.5 | 53745.01 | 99.49% | 0.7(0.61-0.79) | 1551.72 | 2917.15 | 87.99% | 0.12(0-0.23) | 30614.66 | 48681.53 | 59.01% | 0.09(-0.02-0.2) |
| Argentina | UDSD | 1699878.72 | 2586748.46 | 52.17% | -0.141(-0.194 - -0.089) | 1031.64 | 1038.86 | 0.70% | -1.97(-2.33 - -1.61) | 62730.41 | 79512.75 | 26.75% | -0.8(-0.92 - -0.68) |
| Argentina | VAID | 5100.67 | 9268.48 | 81.71% | 0.23(0.18-0.28) | 733.18 | 1050.45 | 43.27% | -0.9(-1.09 - -0.71) | 14194.59 | 18328.82 | 29.13% | -0.91(-1.06 - -0.77) |
| Armenia | Digestive diseases | 174174.97 | 200280.56 | 14.99% | 0.18(0.16-0.2) | 801.51 | 1720.34 | 114.64% | 1.87(1.37-2.36) | 33092.01 | 45280.35 | 36.83% | 0.65(0.29-1.02) |
| Armenia | APED | 8042.08 | 6796.7 | -15.49% | 0.35(0.29-0.41) | 12.58 | 6.68 | -46.90% | -2.46(-3.2 - -1.72) | 649.37 | 299.7 | -53.85% | -2.06(-2.71 - -1.39) |
| Armenia | COCLD | 660.31 | 1595.56 | 141.64% | 3.28(3.09-3.48) | 405.68 | 1114.18 | 174.65% | 2.78(2.18-3.38) | 12191.85 | 25308.46 | 107.59% | 1.92(1.4-2.45) |
| Armenia | GABD | 14295.1 | 19186.85 | 34.22% | 0.61(0.55-0.67) | 44.88 | 81.41 | 81.39% | 1.27(0.76-1.79) | 2886.26 | 3410.61 | 18.17% | -0.05(-0.29-0.19) |
| Armenia | IFAH | 4914.63 | 7450.01 | 51.59% | 1.88(1.55-2.2) | 22.87 | 39.8 | 74.03% | 0.26(0.04-0.48) | 1661.67 | 2049.66 | 23.35% | 0.37(0.19-0.55) |
| Armenia | IFBD | 178.48 | 236.7 | 32.62% | 0.71(0.66-0.77) | 7.15 | 10.82 | 51.33% | 0.72(0.36-1.09) | 637.47 | 764.07 | 19.86% | 0.2(0.05-0.36) |
| Armenia | PACA | 913.42 | 1111.6 | 21.70% | 0.05(0.03-0.07) | 17.04 | 31.2 | 83.10% | 1.06(0.75-1.37) | 601.98 | 862.79 | 43.33% | 0.66(0.33-0.99) |
| Armenia | PIIO | 2528.28 | 3589.96 | 41.99% | 0.92(0.84-1) | 78.21 | 46.54 | -40.49% | -1.69(-1.9 - -1.47) | 5406.32 | 1497.68 | -72.30% | 0.2(-3.3 - -2.8) |
| Armenia | UDSD | 142054.42 | 159273.28 | 12.12% | 0.015(0.011-0.019) | 133.4 | 222.79 | 67.01% | 0.9(0.33-1.47) | 7081.23 | 7980.77 | 12.70% | -0.32(-0.62 - -0.03) |
| Armenia | VAID | 588.25 | 1039.9 | 76.78% | 1.38(1.24-1.52) | 70.29 | 145.27 | 106.67% | 0.88(0.59-1.17) | 1528.31 | 2440.37 | 59.68% | 0.26(-0.02-0.53) |
| Australia | Digestive diseases | 907349.66 | 1492051.79 | 64.44% | 0.02(-0.07-0.11) | 3944.14 | 6632.29 | 68.16% | -1.06(-1.22 - -0.89) | 114575.28 | 166488.49 | 45.31% | -0.78(-0.92 - -0.63) |
| Australia | APED | 46185.16 | 62290.79 | 34.87% | 0.14(0.13-0.15) | 27.74 | 34.93 | 25.92% | -2.14(-2.26 - -2.02) | 1188.04 | 1359.1 | 14.40% | -0.96(-1.04 - -0.87) |
| Australia | COCLD | 2072.96 | 2876.7 | 38.77% | -0.34(-0.41 - -0.26) | 1394.49 | 2214.76 | 58.82% | -0.68(-0.86 - -0.49) | 40413.82 | 56515.5 | 39.84% | -0.72(-0.91 - -0.53) |
| Australia | GABD | 91930.75 | 164233.97 | 78.65% | 0.12(0.09-0.15) | 246.3 | 543.54 | 120.68% | -0.45(-0.54 - -0.37) | 10883.72 | 17756.62 | 63.15% | -0.5(-0.57 - -0.44) |
| Australia | IFAH | 20427.49 | 35965.45 | 76.06% | 0.03(0-0.05) | 93.26 | 195.92 | 110.08% | -0.33(-0.53 - -0.13) | 5009.8 | 7811.6 | 55.93% | -0.51(-0.59 - -0.42) |
| Australia | IFBD | 2212.9 | 5354.02 | 141.95% | 1.13(0.85-1.41) | 97.19 | 368.54 | 279.20% | 2.23(1.81-2.66) | 4535.93 | 11569.09 | 155.05% | 1.2(0.98-1.41) |
| Australia | PACA | 7381.32 | 12151.18 | 64.62% | -0.21(-0.24 - -0.18) | 179.36 | 279.92 | 56.07% | -1.35(-1.5 - -1.2) | 4652.69 | 5903.33 | 26.88% | -1.42(-1.6 - -1.25) |
| Australia | PIIO | 30803.61 | 65502.47 | 112.65% | 0.43(0.35-0.5) | 308.63 | 932.31 | 202.08% | 0.52(0.45-0.6) | 5272.33 | 12012.25 | 127.84% | 0.26(0.17-0.35) |
| Australia | UDSD | 701165 | 1132772.33 | 61.56% | -0.023(-0.141-0.094) | 875.05 | 434.81 | -50.31% | -6.09(-6.61 - -5.57) | 30156.36 | 31452.71 | 4.30% | -1.67(-2 - -1.34) |
| Australia | VAID | 5170.45 | 10904.87 | 110.91% | 0.01(-0.08-0.1) | 343.35 | 627.39 | 82.73% | -1.21(-1.28 - -1.13) | 5823.53 | 8737.53 | 50.04% | -1.48(-1.55 - -1.41) |
| Austria | Digestive diseases | 542260.92 | 672804.16 | 24.07% | -0.05(-0.11-0) | 3978.94 | 3236.69 | -18.65% | -2.39(-2.48 - -2.3) | 119263.96 | 94484.97 | -20.78% | -2.06(-2.12 - -2) |
| Austria | APED | 26138.58 | 29877.62 | 14.30% | 0.37(0.18-0.56) | 22 | 13.07 | -40.59% | -3.27(-3.58 - -2.95) | 751.1 | 572.17 | -23.82% | -0.95(-1.01 - -0.9) |
| Austria | COCLD | 3449.11 | 2913.68 | -15.52% | -1.31(-1.46 - -1.16) | 2253.14 | 1809.45 | -19.69% | -2.33(-2.42 - -2.25) | 65401.16 | 45733.86 | -30.07% | -2.73(-2.82 - -2.65) |
| Austria | GABD | 95764.35 | 105653.04 | 10.33% | -0.8(-1.04 - -0.56) | 207.35 | 188.07 | -9.30% | -1.69(-2.22 - -1.16) | 10515.46 | 9533.71 | -9.34% | -1.42(-1.54 - -1.31) |
| Austria | IFAH | 9390.52 | 17674.95 | 88.22% | 1.66(1.48-1.84) | 89.89 | 58.55 | -34.86% | -3.13(-3.42 - -2.84) | 3338.67 | 3729.16 | 11.70% | -0.12(-0.34-0.1) |
| Austria | IFBD | 1617.56 | 1939.54 | 19.91% | 0.45(0.31-0.59) | 98.63 | 101.12 | 2.52% | -1.59(-2.16 - -1.03) | 5698.76 | 6463.16 | 13.41% | -0.39(-0.65 - -0.12) |
| Austria | PACA | 3695.2 | 4985.09 | 34.91% | -0.07(-0.15-0.01) | 195.07 | 125.77 | -35.53% | -3.32(-3.58 - -3.06) | 5243.21 | 3051.23 | -41.81% | -3.31(-3.58 - -3.04) |
| Austria | PIIO | 11276.58 | 18598.03 | 64.93% | 0.6(0.49-0.72) | 156.17 | 256.8 | 64.43% | 0.03(-0.3-0.37) | 2961.03 | 3608.96 | 21.88% | -0.85(-1.1 - -0.59) |
| Austria | UDSD | 388895.54 | 487171.68 | 25.27% | 0.002(-0.001-0.005) | 521.28 | 201.17 | -61.41% | -5.31(-5.62 - -5) | 18200.18 | 14645.73 | -19.53% | -1.51(-1.69 - -1.33) |
| Austria | VAID | 2033.48 | 3990.53 | 96.24% | 1.68(1.23-2.13) | 282.44 | 243 | -13.96% | -2.71(-3.19 - -2.24) | 4269.7 | 3354.88 | -21.43% | -2.66(-3.04 - -2.29) |
| Azerbaijan | Digestive diseases | 342046.61 | 600325.13 | 75.51% | 0.07(0.05-0.09) | 2311.4 | 3945.23 | 70.69% | -0.23(-0.64-0.19) | 88599.46 | 136725.14 | 54.32% | -0.86(-1.16 - -0.56) |
| Azerbaijan | APED | 18579.59 | 24200.15 | 30.25% | 0.04(-0.09-0.18) | 32.98 | 11.82 | -64.16% | -4.94(-5.42 - -4.46) | 1940.38 | 807.29 | -58.40% | -4.43(-4.95 - -3.9) |
| Azerbaijan | COCLD | 2210.33 | 6436.92 | 191.22% | 1.53(1.36-1.7) | 1560.25 | 3117.97 | 99.84% | 0.17(-0.33-0.67) | 47356.09 | 88789.07 | 87.49% | -0.51(-0.93 - -0.08) |
| Azerbaijan | GABD | 23922.82 | 44885.74 | 87.63% | 0.17(0.1-0.23) | 64.92 | 62.21 | -4.17% | -1.66(-1.91 - -1.42) | 4895.81 | 6246.84 | 27.60% | -1.48(-1.59 - -1.37) |
| Azerbaijan | IFAH | 8611.71 | 16747.64 | 94.48% | 1.35(0.94-1.76) | 15.59 | 16.42 | 5.32% | -1.19(-1.46 - -0.91) | 2745.48 | 4118.81 | 50.02% | 0.02(-0.05-0.08) |
| Azerbaijan | IFBD | 431.54 | 804.68 | 86.47% | 0.18(0.13-0.23) | 11.61 | 13.04 | 12.32% | -1.09(-1.19 - -1) | 1079.5 | 1829.39 | 69.47% | -0.36(-0.45 - -0.27) |
| Azerbaijan | PACA | 1699.35 | 3111.38 | 83.09% | -0.04(-0.05 - -0.03) | 40.06 | 64.91 | 62.03% | -0.4(-0.55 - -0.25) | 1563.24 | 2435.16 | 55.78% | -0.89(-1.05 - -0.73) |
| Azerbaijan | PIIO | 4032.54 | 7736.05 | 91.84% | 0.75(0.61-0.89) | 108.16 | 85.45 | -21.00% | -0.95(-1.2 - -0.7) | 7168.31 | 4360.51 | -39.17% | -1.36(-1.6 - -1.11) |
| Azerbaijan | UDSD | 281784.64 | 494971.33 | 75.66% | -0.005(-0.009 - -0.001) | 244.27 | 219.55 | -10.12% | -2.81(-3.29 - -2.32) | 14424.27 | 17613.68 | 22.11% | -2.07(-2.35 - -1.79) |
| Azerbaijan | VAID | 774.09 | 1431.25 | 84.89% | 1.24(0.96-1.52) | 25.29 | 46.16 | 82.52% | 0.69(0.58-0.8) | 604.75 | 1134.19 | 87.55% | 0.23(0.13-0.33) |
| Bahamas | Digestive diseases | 17082.13 | 31205.24 | 82.68% | 0.06(0.05-0.06) | 71.2 | 119.42 | 67.72% | -1.25(-1.41 - -1.09) | 2963.69 | 4610.02 | 55.55% | -1.11(-1.26 - -0.96) |
| Bahamas | APED | 639.35 | 1127.5 | 76.35% | 1.01(0.96-1.07) | 1.28 | 1.66 | 29.69% | -0.89(-1.32 - -0.46) | 65.61 | 74.9 | 14.16% | -0.76(-1.14 - -0.38) |
| Bahamas | COCLD | 50.78 | 82.06 | 61.60% | -0.92(-1.03 - -0.8) | 39.71 | 61.5 | 54.87% | -1.73(-1.94 - -1.52) | 1338.32 | 1953.87 | 45.99% | -1.77(-1.99 - -1.54) |
| Bahamas | GABD | 773.68 | 1428.14 | 84.59% | -0.09(-0.12 - -0.05) | 4.11 | 6.38 | 55.23% | -1.34(-1.63 - -1.05) | 202.42 | 309.39 | 52.85% | -1.04(-1.25 - -0.82) |
| Bahamas | IFAH | 556.02 | 945.31 | 70.01% | 0.12(0.05-0.2) | 1.42 | 2.35 | 65.49% | -0.85(-1.04 - -0.66) | 186.98 | 273.55 | 46.30% | -0.5(-0.57 - -0.43) |
| Bahamas | IFBD | 5.22 | 13.83 | 164.94% | 1.14(1.01-1.27) | 1.02 | 1.86 | 82.35% | -0.66(-0.78 - -0.53) | 48.64 | 77.96 | 60.28% | -0.72(-0.81 - -0.63) |
| Bahamas | PACA | 67.38 | 123.38 | 83.11% | -0.07(-0.1 - -0.05) | 4.06 | 8.11 | 99.75% | -0.23(-0.34 - -0.12) | 155.25 | 286.6 | 84.61% | -0.24(-0.35 - -0.12) |
| Bahamas | PIIO | 210.77 | 396.4 | 88.07% | 0.33(0.22-0.44) | 6.08 | 11.82 | 94.38% | -0.31(-0.38 - -0.23) | 231.84 | 318.74 | 37.48% | -0.78(-0.86 - -0.7) |
| Bahamas | UDSD | 14753.13 | 27034.23 | 83.24% | 0.024(0.021-0.027) | 8.69 | 11.63 | 33.83% | -2.36(-2.6 - -2.13) | 586.25 | 948.52 | 61.79% | -0.95(-1.06 - -0.83) |
| Bahamas | VAID | 25.81 | 54.4 | 110.77% | 0.07(0.05-0.09) | 2.45 | 4.92 | 100.82% | -0.65(-0.82 - -0.48) | 61.77 | 115.5 | 86.98% | -0.72(-0.93 - -0.52) |
| Bahrain | Digestive diseases | 25175.87 | 96900.09 | 284.89% | 0.06(0.05-0.07) | 89.24 | 211.59 | 137.09% | -2.71(-3.02 - -2.4) | 3415.22 | 8660.3 | 153.58% | -2.69(-2.93 - -2.45) |
| Bahrain | APED | 1104.97 | 4187.59 | 278.98% | 1.32(1.29-1.35) | 1.4 | 1.57 | 12.14% | -3.68(-4.02 - -3.34) | 69.26 | 92.99 | 34.26% | -3.44(-3.7 - -3.17) |
| Bahrain | COCLD | 89.37 | 480.21 | 437.33% | 0.28(0.11-0.45) | 50.06 | 138.33 | 176.33% | -2.32(-2.57 - -2.07) | 1609.91 | 4283.28 | 166.06% | -2.71(-2.95 - -2.47) |
| Bahrain | GABD | 849.04 | 3781.14 | 345.34% | -0.34(-0.45 - -0.23) | 4.71 | 8.85 | 87.90% | -3.03(-3.48 - -2.57) | 208.89 | 503.06 | 140.83% | -3.1(-3.45 - -2.75) |
| Bahrain | IFAH | 293.18 | 1363.09 | 364.93% | 1.04(1.01-1.07) | 0.73 | 1.13 | 54.79% | -2.76(-3.11 - -2.42) | 95.52 | 275.34 | 188.25% | -1.05(-1.18 - -0.92) |
| Bahrain | IFBD | 13.44 | 58.31 | 333.85% | 0.45(0.28-0.62) | 0.43 | 1.19 | 176.74% | -1.34(-1.69 - -0.99) | 43.54 | 166.78 | 283.05% | -0.53(-0.63 - -0.43) |
| Bahrain | PACA | 112.52 | 443.63 | 294.27% | -0.22(-0.25 - -0.2) | 2.82 | 8.43 | 198.94% | -1.43(-1.79 - -1.07) | 91.97 | 273.24 | 197.10% | -1.65(-1.92 - -1.39) |
| Bahrain | PIIO | 353.85 | 1879.52 | 431.16% | 1.82(1.68-1.95) | 5.18 | 15.55 | 200.44% | 0.47(-0.05-1) | 226.96 | 387.48 | 70.73% | -0.56(-0.93 - -0.19) |
| Bahrain | UDSD | 22312.65 | 84468.89 | 278.57% | -0.033(-0.043 - -0.023) | 14.5 | 20.54 | 41.66% | -4.28(-4.84 - -3.73) | 823.37 | 2289.78 | 178.10% | -3.03(-3.37 - -2.69) |
| Bahrain | VAID | 46.85 | 237.71 | 407.39% | -0.25(-0.51-0.01) | 4.49 | 8.69 | 93.54% | -3.94(-4.73 - -3.14) | 91.21 | 182.15 | 99.70% | -4.06(-4.83 - -3.29) |
| Bangladesh | Digestive diseases | 5741150.53 | 11842821.29 | 106.28% | 0.03(-0.05-0.11) | 47555.02 | 41679.2 | -12.36% | -3.61(-3.83 - -3.4) | 2116496.54 | 1674977.74 | -20.86% | -3.42(-3.55 - -3.29) |
| Bangladesh | APED | 1086794.92 | 2295560.88 | 111.22% | 1.14(1.01-1.28) | 2532.08 | 1545.73 | -38.95% | -3.98(-4.21 - -3.76) | 142473.27 | 89973.87 | -36.85% | -3.3(-3.41 - -3.2) |
| Bangladesh | COCLD | 18413.63 | 28892.64 | 56.91% | 0.36(0.2-0.52) | 26628.43 | 26164.6 | -1.74% | -3.03(-3.33 - -2.73) | 1109872.48 | 842270.28 | -24.11% | -3.45(-3.67 - -3.23) |
| Bangladesh | GABD | 79377.96 | 208749.39 | 162.98% | 1.21(1.16-1.26) | 921.15 | 1043.12 | 13.24% | -2.6(-2.86 - -2.34) | 45711.45 | 51643.15 | 12.98% | -2.05(-2.18 - -1.93) |
| Bangladesh | IFAH | 105377.2 | 266905.29 | 153.29% | 1.26(1.09-1.44) | 312.61 | 397.74 | 27.23% | -1.76(-2.11 - -1.41) | 45258.5 | 71162.22 | 57.24% | -0.18(-0.33 - -0.04) |
| Bangladesh | IFBD | 1355.12 | 3404.18 | 151.21% | 0.7(0.62-0.78) | 544.42 | 712.65 | 30.90% | -2.14(-2.39 - -1.89) | 23780.62 | 26353.91 | 10.82% | -2.03(-2.15 - -1.92) |
| Bangladesh | PACA | 23619.61 | 49297.19 | 108.71% | 0.18(0.14-0.23) | 1747.35 | 2459.36 | 40.75% | -1.89(-2.14 - -1.64) | 66991.66 | 82793.65 | 23.59% | -1.99(-2.14 - -1.83) |
| Bangladesh | PIIO | 184686.35 | 423416.44 | 129.26% | 0.55(0.49-0.61) | 5437.74 | 5783.47 | 6.36% | -2.39(-2.61 - -2.16) | 284007.16 | 208534.65 | -26.57% | -2.67(-2.81 - -2.52) |
| Bangladesh | UDSD | 4237215.19 | 8550691.87 | 101.80% | -0.282(-0.376 - -0.189) | 8583.78 | 1582.93 | -81.56% | -10.48(-11.13 - -9.82) | 373084.58 | 254665.82 | -31.74% | -5.39(-5.93 - -4.85) |
| Bangladesh | VAID | 4310.53 | 15903.41 | 268.94% | 2.41(2.31-2.5) | 585.43 | 1569.65 | 168.12% | 0(-0.29-0.3) | 13970.76 | 32388.8 | 131.83% | -0.16(-0.37-0.05) |
| Barbados | Digestive diseases | 19100.3 | 26870.22 | 40.68% | 0.09(0.08-0.09) | 82.18 | 113.02 | 37.53% | -0.94(-1.05 - -0.82) | 2642.7 | 3424.59 | 29.59% | -0.92(-1.02 - -0.81) |
| Barbados | APED | 589.21 | 798.56 | 35.53% | 1.02(0.99-1.05) | 1.67 | 1.48 | -11.38% | -1.78(-2.16 - -1.4) | 62.56 | 48.58 | -22.35% | -1.65(-2.01 - -1.3) |
| Barbados | COCLD | 36.63 | 46.98 | 28.26% | -0.52(-0.59 - -0.45) | 35.65 | 46.73 | 31.08% | -1.42(-1.62 - -1.22) | 972.34 | 1204.98 | 23.93% | -1.63(-1.85 - -1.41) |
| Barbados | GABD | 824.46 | 1163.49 | 41.12% | 0.06(0.01-0.11) | 3.97 | 4.13 | 4.03% | -1.54(-1.81 - -1.27) | 172.41 | 193.62 | 12.30% | -1.04(-1.2 - -0.87) |
| Barbados | IFAH | 497.57 | 785.63 | 57.89% | 0.55(0.49-0.61) | 2.08 | 2.85 | 37.02% | -0.71(-0.83 - -0.59) | 161.74 | 216.87 | 34.09% | -0.18(-0.22 - -0.14) |
| Barbados | IFBD | 11.09 | 18.04 | 62.67% | 0.33(0.17-0.5) | 1.19 | 1.76 | 47.90% | -0.41(-0.57 - -0.25) | 58.01 | 80.72 | 39.15% | -0.42(-0.48 - -0.36) |
| Barbados | PACA | 77.21 | 106.52 | 37.96% | -0.09(-0.1 - -0.07) | 3.3 | 5.25 | 59.09% | -0.28(-0.38 - -0.17) | 100.57 | 144.81 | 43.99% | -0.5(-0.61 - -0.38) |
| Barbados | PIIO | 288.36 | 486.27 | 68.63% | 0.63(0.55-0.71) | 13.95 | 20.79 | 49.03% | -0.22(-0.31 - -0.13) | 305.53 | 402.04 | 31.59% | -0.52(-0.58 - -0.47) |
| Barbados | UDSD | 16750.04 | 23421.61 | 39.83% | 0.025(0.023-0.027) | 14.91 | 14.49 | -2.82% | -2.28(-2.45 - -2.1) | 689.62 | 834.01 | 20.94% | -0.92(-1 - -0.84) |
| Barbados | VAID | 25.74 | 43.13 | 67.56% | 0.35(0.31-0.39) | 2.33 | 3.26 | 39.91% | -0.65(-0.8 - -0.49) | 43.87 | 59.89 | 36.52% | -0.88(-1.07 - -0.68) |
| Belarus | Digestive diseases | 744564.52 | 784265.42 | 5.33% | 0.09(0.06-0.11) | 2467.47 | 4188.4 | 69.74% | 1.66(0.99-2.33) | 99515.32 | 152557.03 | 53.30% | 1.48(0.83-2.13) |
| Belarus | APED | 30976.32 | 25809.24 | -16.68% | 0.1(0.02-0.17) | 47.16 | 20.95 | -55.58% | -2.96(-3.47 - -2.44) | 1891.93 | 859.77 | -54.56% | -2.28(-2.6 - -1.96) |
| Belarus | COCLD | 1372.89 | 2576.53 | 87.67% | 3.22(2.71-3.74) | 836.71 | 2234.59 | 167.07% | 3.72(2.53-4.92) | 25418.86 | 73482.88 | 189.09% | 4.08(2.75-5.42) |
| Belarus | GABD | 110988.37 | 122284.55 | 10.18% | 0.1(0.04-0.16) | 226.05 | 168.49 | -25.46% | -1.91(-2.23 - -1.59) | 15929.23 | 13269.79 | -16.70% | -1.07(-1.14 - -1) |
| Belarus | IFAH | 21625.85 | 26362.09 | 21.90% | 0.92(0.66-1.17) | 94.72 | 77.42 | -18.26% | -1.87(-1.98 - -1.76) | 6719.43 | 6086.7 | -9.42% | -0.59(-0.68 - -0.51) |
| Belarus | IFBD | 511.46 | 649.44 | 26.98% | 0.71(0.65-0.77) | 44.74 | 58.12 | 29.91% | 0.24(0.09-0.39) | 2689.57 | 3062.64 | 13.87% | 0.17(0.09-0.25) |
| Belarus | PACA | 7646.36 | 8869.72 | 16.00% | 0.27(0.22-0.32) | 368.46 | 585.81 | 58.99% | 1.05(0.58-1.53) | 12891.71 | 20179.87 | 56.53% | 1.13(0.59-1.67) |
| Belarus | PIIO | 15019.01 | 17072.34 | 13.67% | 0.1(0.02-0.18) | 143.41 | 164.14 | 14.45% | -0.18(-0.34 - -0.02) | 4272.04 | 3860.62 | -9.63% | -0.7(-0.88 - -0.53) |
| Belarus | UDSD | 553390.53 | 574508.09 | 3.82% | 0.017(0.011-0.024) | 380.41 | 256.03 | -32.70% | -2.43(-2.71 - -2.14) | 22508.45 | 19899.52 | -11.59% | -0.88(-1.02 - -0.74) |
| Belarus | VAID | 3033.73 | 6133.41 | 102.17% | 2.05(1.85-2.24) | 266.6 | 517.37 | 94.06% | 1.54(1.37-1.71) | 4845.13 | 8518.13 | 75.81% | 1.27(1.07-1.46) |
| Belgium | Digestive diseases | 564285.71 | 703540.18 | 24.68% | 0.19(0.1-0.28) | 4302.58 | 5662.48 | 31.61% | -1.03(-1.21 - -0.85) | 104987.87 | 120784.83 | 15.05% | -0.78(-0.93 - -0.63) |
| Belgium | APED | 26216.74 | 32130.67 | 22.56% | 0.6(0.4-0.8) | 28.87 | 22.66 | -21.51% | -2.76(-2.92 - -2.59) | 831.98 | 710.88 | -14.56% | -0.82(-0.88 - -0.77) |
| Belgium | COCLD | 2602.18 | 2920.33 | 12.23% | -0.18(-0.28 - -0.08) | 1765.56 | 2105.25 | 19.24% | -0.76(-0.87 - -0.66) | 46073 | 49978.41 | 8.48% | -1(-1.15 - -0.85) |
| Belgium | GABD | 50421.96 | 65576.91 | 30.06% | 0.71(0.11-1.31) | 392.31 | 492.17 | 25.45% | -1.39(-1.49 - -1.29) | 9215.96 | 9895.32 | 7.37% | -0.56(-0.87 - -0.26) |
| Belgium | IFAH | 15336.68 | 19411.14 | 26.57% | 1.06(0.53-1.6) | 132.24 | 110.52 | -16.42% | -3.03(-3.34 - -2.73) | 4779.49 | 4535.42 | -5.11% | -0.31(-0.6 - -0.02) |
| Belgium | IFBD | 829.28 | 1651.25 | 99.12% | 1.96(1.56-2.37) | 112.94 | 272.62 | 141.38% | 0.24(-0.9-1.4) | 3306.81 | 6369.84 | 92.63% | 0.82(0.35-1.28) |
| Belgium | PACA | 2766.99 | 3324.9 | 20.16% | -0.27(-0.31 - -0.23) | 222.44 | 202.54 | -8.95% | -2.13(-2.31 - -1.96) | 5101.67 | 4308.59 | -15.55% | -1.85(-2.02 - -1.68) |
| Belgium | PIIO | 14839.56 | 24096.65 | 62.38% | 0.57(0.49-0.66) | 379 | 725.6 | 91.45% | -0.07(-0.32-0.18) | 5626.9 | 9115.08 | 61.99% | -0.08(-0.29-0.12) |
| Belgium | UDSD | 446143.43 | 546246.39 | 22.44% | 0.038(0.028-0.048) | 610.55 | 363.2 | -40.51% | -4.44(-4.63 - -4.25) | 19733.46 | 17417.11 | -11.74% | -1.34(-1.41 - -1.26) |
| Belgium | VAID | 5128.9 | 8181.95 | 59.53% | 0.18(0.1-0.26) | 496.36 | 820.5 | 65.30% | -0.78(-1.19 - -0.37) | 7497.77 | 11029.69 | 47.11% | -0.65(-0.99 - -0.3) |
| Belize | Digestive diseases | 10045.64 | 28750.65 | 186.20% | 0.09(0.09-0.1) | 37.14 | 102.86 | 176.93% | -0.22(-0.56-0.13) | 1693.46 | 4274.04 | 152.39% | -0.23(-0.46-0.01) |
| Belize | APED | 432.81 | 1283.31 | 196.51% | 1.06(0.96-1.16) | 1.04 | 1.61 | 54.81% | -0.62(-0.9 - -0.35) | 65.94 | 83.92 | 27.27% | -1.03(-1.38 - -0.68) |
| Belize | COCLD | 26.68 | 97.23 | 264.43% | 0.45(0.33-0.56) | 20.09 | 65.46 | 225.83% | -0.03(-0.42-0.36) | 647.86 | 2149.04 | 231.71% | 0.06(-0.3-0.42) |
| Belize | GABD | 411.81 | 1198.7 | 191.08% | 0.24(0.2-0.28) | 2.22 | 4.72 | 112.61% | -0.9(-1.06 - -0.73) | 106.65 | 257.77 | 141.70% | -0.59(-0.68 - -0.51) |
| Belize | IFAH | 331.05 | 858.63 | 159.37% | 0.55(0.5-0.61) | 1.3 | 1.96 | 50.77% | -0.7(-0.93 - -0.47) | 165.51 | 267.22 | 61.45% | -0.55(-0.67 - -0.44) |
| Belize | IFBD | 2.84 | 10.33 | 263.73% | 0.56(0.43-0.68) | 0.83 | 1.81 | 118.07% | -1.14(-1.52 - -0.76) | 40.42 | 77.41 | 91.51% | -1.22(-1.49 - -0.95) |
| Belize | PACA | 35.52 | 99.45 | 179.98% | 0.07(0.05-0.08) | 0.9 | 3.23 | 258.89% | 0.45(0.12-0.79) | 34.3 | 124.32 | 262.45% | 0.49(0.2-0.77) |
| Belize | PIIO | 132.52 | 319.6 | 141.18% | 0.43(0.35-0.51) | 5.31 | 9.42 | 77.44% | -0.52(-0.61 - -0.43) | 265.63 | 306.79 | 15.50% | -1.26(-1.34 - -1.18) |
| Belize | UDSD | 8663.34 | 24855.62 | 186.91% | 0.032(0.027-0.037) | 4 | 7.66 | 91.50% | -1.78(-2.27 - -1.29) | 306.68 | 799.92 | 160.83% | -0.55(-0.72 - -0.38) |
| Belize | VAID | 9.08 | 27.77 | 205.84% | 0.77(0.72-0.81) | 0.44 | 1.36 | 209.09% | 0.25(-0.05-0.55) | 10.35 | 32.15 | 210.63% | 0.22(-0.06-0.49) |
| Benin | Digestive diseases | 161253.07 | 457542.88 | 183.74% | 0.06(0.05-0.08) | 1910.12 | 3464.64 | 81.38% | -1.07(-1.18 - -0.95) | 80067.15 | 150261.87 | 87.67% | -1.02(-1.12 - -0.91) |
| Benin | APED | 5482.84 | 19803.91 | 261.20% | 0.93(0.8-1.07) | 34.89 | 34.3 | -1.69% | -2.47(-2.74 - -2.19) | 2177.96 | 1976.96 | -9.23% | -3.14(-3.44 - -2.83) |
| Benin | COCLD | 951.04 | 2722.14 | 186.23% | 0.09(-0.11-0.3) | 1144.99 | 1943.06 | 69.70% | -1.5(-1.67 - -1.33) | 40005.91 | 72098.96 | 80.22% | -1.39(-1.55 - -1.23) |
| Benin | GABD | 1372 | 4151.47 | 202.59% | 0.55(0.53-0.57) | 50.5 | 123.23 | 144.02% | 0.58(0.46-0.7) | 2093.94 | 4706.11 | 124.75% | 0.19(0.08-0.3) |
| Benin | IFAH | 4899.06 | 15112.59 | 208.48% | 0.9(0.81-0.98) | 36.15 | 65.48 | 81.13% | -0.59(-0.79 - -0.39) | 3181.38 | 6637.72 | 108.64% | -0.19(-0.3 - -0.07) |
| Benin | IFBD | 31.68 | 96.88 | 205.81% | 0.33(0.23-0.44) | 13.67 | 29.55 | 116.17% | -0.42(-0.64 - -0.2) | 693.5 | 1437.3 | 107.25% | -0.5(-0.7 - -0.3) |
| Benin | PACA | 721.99 | 2005.85 | 177.82% | 0.05(0.03-0.08) | 69.34 | 202.56 | 192.13% | 0.16(0-0.33) | 2608.5 | 7909.65 | 203.23% | 0.25(0.08-0.42) |
| Benin | PIIO | 3519.06 | 10768.19 | 206.00% | 0.65(0.61-0.68) | 234.86 | 479.36 | 104.10% | -0.27(-0.43 - -0.11) | 13020.4 | 24061.01 | 84.79% | -0.5(-0.65 - -0.35) |
| Benin | UDSD | 144047.22 | 402179.99 | 179.20% | 0(-0.005-0.005) | 222.58 | 395.98 | 77.90% | -0.67(-0.77 - -0.57) | 11528.8 | 23514.08 | 103.96% | -0.63(-0.7 - -0.56) |
| Benin | VAID | 228.18 | 701.85 | 207.59% | 0.92(0.85-0.99) | 17.59 | 41.37 | 135.19% | 0.65(0.52-0.78) | 773.54 | 1443.15 | 86.56% | 0.06(-0.09-0.2) |
| Bermuda | Digestive diseases | 4908.91 | 6328.66 | 28.92% | 0.14(0.13-0.15) | 19.46 | 19.25 | -1.04% | -2.84(-3.09 - -2.58) | 656.4 | 595.11 | -9.34% | -1.97(-2.19 - -1.74) |
| Bermuda | APED | 133.27 | 166.04 | 24.59% | 1.18(1.12-1.25) | 0.36 | 0.24 | -33.33% | -3.24(-3.94 - -2.53) | 13.29 | 7.36 | -44.62% | -2.36(-2.96 - -1.76) |
| Bermuda | COCLD | 11.44 | 10.56 | -7.69% | -0.99(-1.13 - -0.86) | 9.89 | 7.9 | -20.12% | -3.32(-3.71 - -2.93) | 273.08 | 189.97 | -30.43% | -3.28(-3.68 - -2.87) |
| Bermuda | GABD | 216.4 | 287.36 | 32.79% | 0.16(0.1-0.21) | 0.9 | 0.65 | -27.78% | -3.93(-4.45 - -3.41) | 40.7 | 34.47 | -15.31% | -1.9(-2.19 - -1.61) |
| Bermuda | IFAH | 150.73 | 229.1 | 51.99% | 0.73(0.7-0.76) | 0.56 | 0.64 | 14.29% | -2.55(-2.91 - -2.2) | 45.54 | 51.07 | 12.14% | -0.67(-0.81 - -0.52) |
| Bermuda | IFBD | 1.58 | 2.47 | 56.33% | 0.54(0.4-0.69) | 0.53 | 0.45 | -15.09% | -3.73(-4.07 - -3.4) | 17.89 | 13.9 | -22.30% | -2.73(-3 - -2.46) |
| Bermuda | PACA | 20.53 | 26.37 | 28.45% | -0.25(-0.27 - -0.24) | 1.09 | 1.12 | 2.75% | -2.47(-2.67 - -2.26) | 31.26 | 26.66 | -14.72% | -2.27(-2.48 - -2.07) |
| Bermuda | PIIO | 75.92 | 173.38 | 128.37% | 1.5(1.36-1.63) | 0.49 | 1.84 | 277.78% | 1.52(0.86-2.19) | 13.33 | 33.59 | 152.07% | 1.2(0.68-1.71) |
| Bermuda | UDSD | 4290.84 | 5416.44 | 26.23% | 0.036(0.033-0.039) | 3.65 | 2.37 | -35.07% | -4.67(-4.96 - -4.39) | 180.04 | 173.07 | -3.87% | -1.44(-1.59 - -1.29) |
| Bermuda | VAID | 8.2 | 16.95 | 106.71% | 0.49(0.46-0.52) | 0.97 | 1.17 | 20.62% | -2.86(-3.08 - -2.65) | 17.33 | 17.93 | 3.46% | -2.83(-3.05 - -2.62) |
| Bhutan | Digestive diseases | 32447.41 | 54730.8 | 68.68% | 0.17(0.13-0.21) | 213.39 | 285.33 | 33.71% | -1.39(-1.44 - -1.34) | 9727.06 | 10298.38 | 5.87% | -1.7(-1.76 - -1.65) |
| Bhutan | APED | 6080.53 | 9738.31 | 60.16% | 1.03(0.69-1.37) | 8.44 | 5.21 | -38.27% | -3.43(-3.54 - -3.32) | 501.97 | 304.74 | -39.29% | -3.15(-3.25 - -3.04) |
| Bhutan | COCLD | 125.91 | 200.91 | 59.57% | 0.61(0.56-0.66) | 111.15 | 176.26 | 58.58% | -0.63(-0.74 - -0.53) | 4716.23 | 5871.83 | 24.50% | -1.12(-1.23 - -1.01) |
| Bhutan | GABD | 435.89 | 924.68 | 112.14% | 0.92(0.9-0.94) | 3.17 | 4.85 | 53.00% | -1.15(-1.22 - -1.07) | 195.82 | 214.98 | 9.78% | -1.72(-1.83 - -1.62) |
| Bhutan | IFAH | 678.3 | 1339.38 | 97.46% | 0.68(0.6-0.76) | 4.86 | 4.52 | -7.00% | -2.57(-2.69 - -2.44) | 414.07 | 379.79 | -8.28% | -1.88(-2 - -1.76) |
| Bhutan | IFBD | 7.5 | 14.13 | 88.40% | 0.09(-0.1-0.29) | 1.84 | 2.88 | 56.52% | -0.84(-0.97 - -0.72) | 85.59 | 101.77 | 18.90% | -1.18(-1.27 - -1.1) |
| Bhutan | PACA | 143.35 | 247.92 | 72.95% | 0.13(0.09-0.16) | 7.04 | 11.25 | 59.80% | -0.67(-0.75 - -0.58) | 292.47 | 368.77 | 26.09% | -1.19(-1.27 - -1.12) |
| Bhutan | PIIO | 961.79 | 1980.62 | 105.93% | 1.1(0.99-1.2) | 21.44 | 31.33 | 46.14% | -0.54(-0.67 - -0.42) | 1077.34 | 1001.91 | -7.00% | -1.23(-1.36 - -1.11) |
| Bhutan | UDSD | 23985.27 | 40197.08 | 67.59% | -0.082(-0.096 - -0.067) | 52.39 | 38.93 | -25.69% | -3.84(-3.99 - -3.69) | 2339.5 | 1839.27 | -21.38% | -3.31(-3.49 - -3.13) |
| Bhutan | VAID | 28.88 | 87.76 | 203.88% | 2.34(2.26-2.42) | 2.11 | 8.11 | 284.36% | 1.47(1.32-1.63) | 53.79 | 150.61 | 180.00% | 0.98(0.85-1.11) |
| Bolivia (Plurinational State of) | Digestive diseases | 411021.65 | 887771.58 | 115.99% | -0.03(-0.05 - -0.01) | 3414.5 | 5832.5 | 70.82% | -1.02(-1.08 - -0.95) | 162793.03 | 190292.76 | 16.89% | -1.73(-1.83 - -1.64) |
| Bolivia (Plurinational State of) | APED | 65145.13 | 98270.25 | 50.85% | -0.66(-0.79 - -0.53) | 280.21 | 139.89 | -50.08% | -4.04(-4.27 - -3.8) | 19459.23 | 7024.79 | -63.90% | -5.01(-5.26 - -4.76) |
| Bolivia (Plurinational State of) | COCLD | 1220.92 | 3422.04 | 180.28% | 0.78(0.66-0.9) | 1487.32 | 3318.77 | 123.14% | -0.52(-0.58 - -0.47) | 51241.29 | 89407.22 | 74.48% | -1.08(-1.14 - -1.01) |
| Bolivia (Plurinational State of) | GABD | 9664.63 | 25405.21 | 162.87% | 0.62(0.58-0.66) | 192.13 | 387.28 | 101.57% | -0.9(-1.01 - -0.79) | 7331.21 | 11577.49 | 57.92% | -1.48(-1.58 - -1.37) |
| Bolivia (Plurinational State of) | IFAH | 15939.75 | 33734.08 | 111.63% | 0.42(0.36-0.49) | 136.96 | 105.75 | -22.79% | -2.34(-2.5 - -2.18) | 15281.04 | 12243.89 | -19.88% | -2.33(-2.45 - -2.21) |
| Bolivia (Plurinational State of) | IFBD | 78.54 | 196.61 | 150.33% | -0.17(-0.38-0.04) | 24.67 | 34.43 | 39.56% | -0.83(-0.91 - -0.75) | 1551.61 | 1348.47 | -13.09% | -2.04(-2.15 - -1.94) |
| Bolivia (Plurinational State of) | PACA | 2158.13 | 4626.81 | 114.39% | -0.34(-0.36 - -0.32) | 211.73 | 355.86 | 68.07% | -1.47(-1.58 - -1.37) | 7913.76 | 10473.25 | 32.34% | -2(-2.11 - -1.88) |
| Bolivia (Plurinational State of) | PIIO | 4699.79 | 10679.45 | 127.23% | 0.43(0.37-0.49) | 439.32 | 584.37 | 33.02% | -0.61(-0.68 - -0.54) | 28510.74 | 19507.7 | -31.58% | -2.38(-2.45 - -2.31) |
| Bolivia (Plurinational State of) | UDSD | 311896.62 | 710785.4 | 127.89% | -0.001(-0.005-0.003) | 480.81 | 587.29 | 22.15% | -2.5(-2.64 - -2.36) | 21988.47 | 29360.25 | 33.53% | -1.95(-2.07 - -1.82) |
| Bolivia (Plurinational State of) | VAID | 218.14 | 651.73 | 198.77% | 0.68(0.62-0.74) | 44.62 | 119.34 | 167.46% | 0.06(-0.04-0.16) | 1327.15 | 2306.54 | 73.80% | -0.7(-0.84 - -0.56) |
| Bosnia and Herzegovina | Digestive diseases | 268952.2 | 255338.83 | -5.06% | 0.09(0.08-0.1) | 1315.12 | 1235.46 | -6.06% | -1.88(-2.12 - -1.63) | 50718.12 | 38768.12 | -23.56% | -1.76(-1.99 - -1.53) |
| Bosnia and Herzegovina | APED | 9547.76 | 5752.56 | -39.75% | 0.05(-0.02-0.12) | 23.27 | 10.27 | -55.87% | -5.23(-6.15 - -4.29) | 909.4 | 300.95 | -66.91% | -4.56(-5.33 - -3.78) |
| Bosnia and Herzegovina | COCLD | 1322.48 | 802.13 | -39.35% | -1.03(-1.1 - -0.96) | 758.67 | 593.31 | -21.80% | -2.33(-2.57 - -2.09) | 23854.06 | 15755.99 | -33.95% | -2.51(-2.78 - -2.25) |
| Bosnia and Herzegovina | GABD | 43142.29 | 47635.76 | 10.42% | 0.37(0.34-0.41) | 54.99 | 73.32 | 33.33% | -0.97(-1.18 - -0.75) | 6164.43 | 5383.94 | -12.66% | -0.96(-1.04 - -0.89) |
| Bosnia and Herzegovina | IFAH | 7546.85 | 6655.07 | -11.82% | 0.18(-0.02-0.37) | 48.21 | 30.69 | -36.34% | -4.47(-5.09 - -3.85) | 2813.59 | 1727.05 | -38.62% | -2.28(-2.64 - -1.91) |
| Bosnia and Herzegovina | IFBD | 344.93 | 365.17 | 5.87% | 1.12(0.97-1.26) | 18.24 | 25.58 | 40.24% | -0.2(-0.52-0.12) | 1284.61 | 1371.94 | 6.80% | 0.14(-0.01-0.28) |
| Bosnia and Herzegovina | PACA | 2052.94 | 2059 | 0.30% | -0.07(-0.11 - -0.04) | 90.01 | 120.75 | 34.15% | -0.51(-0.72 - -0.3) | 2745.58 | 2837.4 | 3.34% | -0.78(-0.99 - -0.56) |
| Bosnia and Herzegovina | PIIO | 3339.39 | 4380.7 | 31.18% | 0.98(0.76-1.2) | 68.04 | 139.13 | 104.47% | 0.9(0.56-1.24) | 1877.44 | 2547.22 | 35.68% | 0.27(0.07-0.48) |
| Bosnia and Herzegovina | UDSD | 201246.11 | 187046.58 | -7.06% | 0.003(-0.006-0.012) | 206.04 | 122.5 | -40.55% | -4.03(-4.47 - -3.59) | 9773.56 | 6554.25 | -32.94% | -2.05(-2.33 - -1.77) |
| Bosnia and Herzegovina | VAID | 409.45 | 641.86 | 56.76% | 1.66(1.5-1.83) | 36.16 | 77.48 | 114.27% | 0.38(0.15-0.61) | 709.35 | 1248.42 | 75.99% | 0.16(0.01-0.31) |
| Botswana | Digestive diseases | 54247.47 | 127124.33 | 134.34% | 0.01(-0.02-0.04) | 443.03 | 785.37 | 77.27% | -1.59(-2.03 - -1.16) | 17709.67 | 31651.43 | 78.72% | -1.54(-1.95 - -1.12) |
| Botswana | APED | 4131.66 | 8008.31 | 93.83% | 0.13(-0.04-0.29) | 8.12 | 8.05 | -0.86% | -3.05(-3.51 - -2.59) | 489.6 | 499.29 | 1.98% | -2.75(-3.15 - -2.35) |
| Botswana | COCLD | 269.88 | 589.52 | 118.44% | 0.15(-0.02-0.32) | 231.44 | 417.72 | 80.49% | -1.66(-2.09 - -1.23) | 8250 | 15138.4 | 83.50% | -1.66(-2.1 - -1.22) |
| Botswana | GABD | 973.01 | 2417.48 | 148.45% | 1.35(0.4-2.3) | 21.37 | 48.46 | 126.77% | -0.43(-0.87-0) | 766.91 | 1668.39 | 117.55% | -0.47(-1-0.06) |
| Botswana | IFAH | 2503.58 | 5058.31 | 102.04% | -0.13(-0.27-0.01) | 7.06 | 12.29 | 74.08% | -1.44(-1.87 - -1) | 990.23 | 1584.5 | 60.01% | -1(-1.23 - -0.78) |
| Botswana | IFBD | 11.61 | 31.44 | 170.80% | 0.62(0.52-0.72) | 4.38 | 8.97 | 104.79% | -0.77(-1.15 - -0.39) | 187.7 | 387.92 | 106.67% | -0.41(-0.71 - -0.11) |
| Botswana | PACA | 181.72 | 403.29 | 121.93% | -0.16(-0.19 - -0.13) | 9.97 | 21.7 | 117.65% | -1.29(-1.83 - -0.74) | 375.67 | 842.06 | 124.15% | -1.33(-1.91 - -0.75) |
| Botswana | PIIO | 1509.71 | 4309.7 | 185.46% | 1.02(0.88-1.15) | 57.84 | 112.52 | 94.53% | -1.22(-1.62 - -0.82) | 2101.54 | 3957.54 | 88.32% | -1.15(-1.51 - -0.79) |
| Botswana | UDSD | 44590.45 | 106098.53 | 137.94% | -0.065(-0.078 - -0.051) | 71.09 | 100.66 | 41.60% | -2.1(-2.6 - -1.6) | 3400.92 | 5694.12 | 67.43% | -1.75(-2.17 - -1.33) |
| Botswana | VAID | 75.84 | 207.76 | 173.95% | 1.28(1.18-1.38) | 4.88 | 13.19 | 170.29% | -0.09(-0.52-0.33) | 144.59 | 385.61 | 166.69% | -0.19(-0.65-0.27) |
| Brazil | Digestive diseases | 10337548.31 | 19448348.05 | 88.13% | -0.02(-0.09-0.04) | 43933.24 | 78483.68 | 78.64% | -1.1(-1.16 - -1.05) | 1925884.72 | 2815955.38 | 46.22% | -1.22(-1.24 - -1.19) |
| Brazil | APED | 166813.87 | 281107.09 | 68.52% | 1.01(0.94-1.08) | 766.33 | 1301.43 | 69.83% | -0.16(-0.31-0) | 38555.02 | 47947.47 | 24.36% | -0.38(-0.55 - -0.2) |
| Brazil | COCLD | 35430.22 | 49123.8 | 38.65% | -1.28(-1.41 - -1.16) | 24473.98 | 38028.63 | 55.38% | -1.49(-1.53 - -1.44) | 907970.56 | 1170623.43 | 28.93% | -1.78(-1.82 - -1.73) |
| Brazil | GABD | 797740.24 | 2497016.35 | 213.01% | 1.8(1.45-2.16) | 2464.91 | 7117.36 | 188.75% | 0.14(0.06-0.23) | 142684.1 | 339869.17 | 138.20% | 0.18(0.08-0.29) |
| Brazil | IFAH | 349407.21 | 708514.89 | 102.78% | 0.12(-0.13-0.37) | 1097.05 | 2488.47 | 126.83% | -0.02(-0.3-0.25) | 98432.73 | 153769.2 | 56.22% | -0.6(-0.71 - -0.49) |
| Brazil | IFBD | 7717.14 | 12705.46 | 64.64% | -0.54(-0.59 - -0.5) | 451.06 | 1034.94 | 129.45% | 0.01(-0.16-0.19) | 27396.46 | 46349.6 | 69.18% | -0.44(-0.62 - -0.27) |
| Brazil | PACA | 23196.81 | 46320.52 | 99.68% | -0.12(-0.18 - -0.07) | 2289.64 | 5445.18 | 137.82% | 0.34(0.21-0.46) | 88956.42 | 172622.09 | 94.05% | -0.02(-0.12-0.09) |
| Brazil | PIIO | 133012.97 | 209335.28 | 57.38% | -0.76(-0.94 - -0.57) | 3344.03 | 7067.62 | 111.35% | -0.2(-0.28 - -0.12) | 147797.77 | 175523.93 | 18.76% | -0.84(-0.92 - -0.75) |
| Brazil | UDSD | 8804652.93 | 15620863.83 | 77.42% | -0.28(-0.32 - -0.241) | 5329.19 | 5831.62 | 9.43% | -3.32(-3.53 - -3.12) | 370029.92 | 488715.05 | 32.07% | -1.75(-1.84 - -1.66) |
| Brazil | VAID | 19576.92 | 23360.84 | 19.33% | -1.21(-1.82 - -0.6) | 2248.71 | 4399.4 | 95.64% | -1.54(-1.61 - -1.47) | 55152.65 | 89266.66 | 61.85% | -1.64(-1.72 - -1.56) |
| Brunei Darussalam | Digestive diseases | 9767.14 | 20906.38 | 114.05% | 0(-0.01-0) | 37.36 | 60.68 | 62.43% | -1.85(-2.09 - -1.62) | 1718.7 | 2761 | 60.64% | -1.73(-1.96 - -1.5) |
| Brunei Darussalam | APED | 1201.06 | 1925.74 | 60.34% | 0.27(0.22-0.32) | 0.41 | 0.68 | 65.85% | 0(-0.33-0.32) | 32.93 | 46.06 | 39.87% | -0.41(-0.68 - -0.14) |
| Brunei Darussalam | COCLD | 52.17 | 77.46 | 48.48% | -1.43(-1.54 - -1.33) | 18.02 | 31.17 | 72.97% | -1.98(-2.21 - -1.74) | 674.72 | 1088.65 | 61.35% | -2.14(-2.42 - -1.86) |
| Brunei Darussalam | GABD | 1803.51 | 4455.47 | 147.04% | 0.06(0.02-0.1) | 3.17 | 7.03 | 121.77% | -0.27(-0.38 - -0.16) | 255 | 532.44 | 108.80% | -0.62(-0.68 - -0.57) |
| Brunei Darussalam | IFAH | 436.73 | 709.2 | 62.39% | 0.18(0.14-0.22) | 0.24 | 0.56 | 133.33% | -0.2(-0.35 - -0.04) | 112.18 | 161.47 | 43.94% | -0.37(-0.45 - -0.29) |
| Brunei Darussalam | IFBD | 12.69 | 25.73 | 102.76% | 0.08(0.04-0.12) | 1.13 | 2.57 | 127.43% | 0.09(-0.02-0.2) | 57.09 | 113.86 | 99.44% | -0.25(-0.33 - -0.18) |
| Brunei Darussalam | PACA | 47.25 | 96.9 | 105.08% | -0.19(-0.22 - -0.15) | 1.52 | 3.1 | 103.95% | -0.71(-0.81 - -0.61) | 61.47 | 114.56 | 86.37% | -0.96(-1.12 - -0.78) |
| Brunei Darussalam | PIIO | 152.43 | 341.27 | 123.89% | 0.41(0.33-0.49) | 1.74 | 5.38 | 209.12% | 1.59(1.45-1.73) | 67.23 | 149.71 | 122.69% | 0.9(0.76-1.05) |
| Brunei Darussalam | UDSD | 5999.44 | 13109.7 | 118.52% | -0.087(-0.095 - -0.078) | 8.73 | 5.35 | -38.72% | -5.4(-5.87 - -4.94) | 374.74 | 414.14 | 10.51% | -3.74(-4.12 - -3.35) |
| Brunei Darussalam | VAID | 61.86 | 164.91 | 166.59% | 0.68(0.61-0.74) | 1.06 | 2.9 | 173.58% | 0.32(0.23-0.4) | 30.39 | 73.11 | 140.57% | -0.02(-0.12-0.09) |
| Bulgaria | Digestive diseases | 606891.74 | 556304.64 | -8.34% | 0(-0.02-0.02) | 3786.01 | 4506.74 | 19.04% | 0.08(-0.1-0.26) | 135000 | 138907.87 | 2.89% | -0.01(-0.17-0.15) |
| Bulgaria | APED | 17336 | 12531.04 | -27.72% | 0.16(0.11-0.21) | 42.49 | 42.52 | 0.07% | 0.2(-0.45-0.85) | 1410.73 | 1064.18 | -24.57% | -0.29(-0.77-0.2) |
| Bulgaria | COCLD | 2651.28 | 2425.46 | -8.52% | 0.4(0.37-0.43) | 2246.2 | 2707.09 | 20.52% | 0.31(0.1-0.52) | 67942.64 | 79237.73 | 16.62% | 0.43(0.2-0.65) |
| Bulgaria | GABD | 100144.87 | 97776.89 | -2.36% | 0.11(0.02-0.19) | 140.71 | 124.73 | -11.36% | -0.96(-1.51 - -0.4) | 13157.05 | 10762.83 | -18.20% | -0.56(-0.71 - -0.4) |
| Bulgaria | IFAH | 27628.36 | 14114.68 | -48.91% | -1.85(-2.09 - -1.62) | 180.1 | 77.57 | -56.93% | -5.91(-6.71 - -5.1) | 9439.68 | 3977.76 | -57.86% | -3.66(-4.13 - -3.18) |
| Bulgaria | IFBD | 741.6 | 850.88 | 14.74% | 0.76(0.53-0.99) | 24.6 | 40.72 | 65.53% | 1.13(0.73-1.54) | 2779.16 | 2569.29 | -7.55% | 0(-0.21-0.21) |
| Bulgaria | PACA | 4489.32 | 4496 | 0.15% | 0.08(0.04-0.11) | 185.64 | 274.71 | 47.98% | 1.19(0.85-1.54) | 5866.48 | 7197 | 22.68% | 0.91(0.63-1.19) |
| Bulgaria | PIIO | 10961.94 | 10549.85 | -3.76% | -0.27(-0.41 - -0.13) | 212.88 | 403.67 | 89.62% | 1.39(1.11-1.67) | 5652.77 | 7644.13 | 35.23% | 0.59(0.37-0.82) |
| Bulgaria | UDSD | 441655.5 | 411701.71 | -6.78% | 0.061(0.053-0.068) | 553.86 | 439.17 | -20.71% | -1.75(-2.05 - -1.46) | 23599.42 | 18492.55 | -21.64% | -0.88(-1.03 - -0.72) |
| Bulgaria | VAID | 1282.86 | 1858.13 | 44.84% | 1.37(1.23-1.52) | 146.98 | 281.47 | 91.50% | 2.38(1.84-2.93) | 3124.08 | 4967.67 | 59.01% | 2.12(1.61-2.64) |
| Burkina Faso | Digestive diseases | 326104.02 | 835721 | 156.27% | 0.12(0.11-0.13) | 3776.2 | 6368.61 | 68.65% | -1.15(-1.27 - -1.03) | 157946.28 | 291259.45 | 84.40% | -0.88(-1.02 - -0.75) |
| Burkina Faso | APED | 10876.79 | 34961.25 | 221.43% | 0.93(0.82-1.04) | 100.02 | 109.23 | 9.21% | -1.69(-2.05 - -1.33) | 6189.41 | 6372.89 | 2.96% | -2.33(-2.68 - -1.97) |
| Burkina Faso | COCLD | 1778.92 | 4254.76 | 139.18% | -0.07(-0.22-0.08) | 2275.59 | 2993.05 | 31.53% | -2.33(-2.64 - -2.02) | 75755.69 | 109924.73 | 45.10% | -2.1(-2.42 - -1.77) |
| Burkina Faso | GABD | 2710.31 | 7821.75 | 188.59% | 0.87(0.83-0.91) | 128.22 | 334.21 | 160.65% | 1.19(0.97-1.4) | 5440.41 | 13165.58 | 142.00% | 0.96(0.79-1.13) |
| Burkina Faso | IFAH | 10476.34 | 31791.34 | 203.46% | 1.72(1.56-1.88) | 90.77 | 185.87 | 104.77% | 0.51(0.19-0.84) | 7278.29 | 15682.48 | 115.47% | 0.71(0.5-0.91) |
| Burkina Faso | IFBD | 59.67 | 159.42 | 167.17% | 0.21(0.06-0.36) | 35.22 | 81.14 | 130.38% | 0.56(0.28-0.85) | 1781.43 | 3941.9 | 121.28% | 0.38(0.15-0.62) |
| Burkina Faso | PACA | 1453.49 | 3752.35 | 158.16% | 0.21(0.18-0.24) | 169.1 | 509.11 | 201.07% | 1.15(0.88-1.42) | 6241.82 | 19702.18 | 215.65% | 1.36(1.11-1.62) |
| Burkina Faso | PIIO | 6728.82 | 18798.98 | 179.38% | 0.82(0.7-0.93) | 475.37 | 1189.36 | 150.20% | 0.86(0.57-1.15) | 27648.09 | 66157.53 | 139.28% | 0.83(0.6-1.06) |
| Burkina Faso | UDSD | 291646.22 | 733088.45 | 151.36% | 0.033(0.028-0.039) | 252.38 | 497.26 | 97.03% | -0.19(-0.47-0.09) | 16016.6 | 36279.96 | 126.51% | 0.03(-0.12-0.17) |
| Burkina Faso | VAID | 373.45 | 1092.7 | 192.60% | 1.19(1.11-1.27) | 38.68 | 97.07 | 150.96% | 1.3(1.06-1.54) | 1705.3 | 3473.01 | 103.66% | 0.83(0.58-1.07) |
| Burundi | Digestive diseases | 195450.14 | 449438.2 | 129.95% | 0.19(0.17-0.2) | 2402.58 | 3542.83 | 47.46% | -1.22(-1.36 - -1.08) | 95038.32 | 145406.93 | 53.00% | -1.23(-1.36 - -1.11) |
| Burundi | APED | 6654.47 | 21172.61 | 218.17% | 1.47(1.34-1.6) | 67.43 | 94.02 | 39.43% | -0.71(-0.91 - -0.51) | 4093.32 | 5174.69 | 26.42% | -1.1(-1.27 - -0.93) |
| Burundi | COCLD | 882.1 | 2004.63 | 127.26% | 0.38(0.29-0.46) | 1458.65 | 1905.75 | 30.65% | -1.9(-2.1 - -1.69) | 49638.16 | 66995.74 | 34.97% | -1.96(-2.16 - -1.77) |
| Burundi | GABD | 1428 | 3888.74 | 172.32% | 1.17(1.12-1.22) | 104.93 | 197.46 | 88.18% | 0.18(0.01-0.34) | 3310.54 | 6137.05 | 85.38% | -0.1(-0.28-0.09) |
| Burundi | IFAH | 3306.24 | 11000.32 | 232.71% | 2.59(2.37-2.82) | 61.04 | 99.7 | 63.34% | 0.38(0.18-0.59) | 4191.31 | 7073.6 | 68.77% | 0.56(0.37-0.75) |
| Burundi | IFBD | 34.15 | 78.56 | 130.04% | 0.27(0.22-0.32) | 21.31 | 37.59 | 76.40% | 0.15(-0.07-0.37) | 1063.86 | 1607.8 | 51.13% | -0.2(-0.43-0.03) |
| Burundi | PACA | 721.79 | 1592.53 | 120.64% | 0.08(0.03-0.13) | 47.76 | 96.71 | 102.49% | -0.17(-0.46-0.12) | 1747.76 | 3690.35 | 111.15% | -0.17(-0.46-0.13) |
| Burundi | PIIO | 3592.88 | 9455.46 | 163.17% | 1.16(1.04-1.29) | 178.29 | 399.98 | 124.34% | 0.83(0.61-1.05) | 9420.23 | 18882.66 | 100.45% | 0.71(0.49-0.93) |
| Burundi | UDSD | 178621.51 | 399718.2 | 123.78% | 0.075(0.063-0.087) | 334.82 | 491.92 | 46.92% | -1.2(-1.44 - -0.95) | 16912.51 | 28504.54 | 68.54% | -0.89(-1.09 - -0.69) |
| Burundi | VAID | 209.01 | 527.15 | 152.21% | 1.33(1.19-1.48) | 27.72 | 54.69 | 97.29% | 0.25(0.02-0.47) | 719.42 | 1412.2 | 96.30% | 0.2(-0.05-0.44) |
| Cabo Verde | Digestive diseases | 13192.78 | 29052.69 | 120.22% | 0.14(0.12-0.16) | 107.35 | 175.48 | 63.46% | -1.13(-1.38 - -0.89) | 3854.65 | 5861.85 | 52.07% | -1.24(-1.38 - -1.09) |
| Cabo Verde | APED | 426.34 | 970.17 | 127.56% | 1.39(1.23-1.56) | 0.53 | 0.49 | -7.55% | -2.46(-2.78 - -2.14) | 22.57 | 23.74 | 5.18% | -1.94(-2.16 - -1.72) |
| Cabo Verde | COCLD | 64.67 | 134.97 | 108.71% | 0.28(0.25-0.32) | 58.83 | 114.33 | 94.34% | -0.84(-1.15 - -0.53) | 1923.2 | 3468.25 | 80.34% | -1.07(-1.31 - -0.83) |
| Cabo Verde | GABD | 115.34 | 246.82 | 113.99% | 0.49(0.47-0.51) | 3.22 | 6.21 | 92.86% | -0.22(-0.54-0.11) | 88.7 | 139.88 | 57.70% | -0.78(-1.01 - -0.55) |
| Cabo Verde | IFAH | 367.46 | 952.92 | 159.33% | 1.49(1.43-1.54) | 1.94 | 2.89 | 48.97% | -1.17(-1.67 - -0.66) | 176.89 | 268.4 | 51.73% | -0.38(-0.49 - -0.26) |
| Cabo Verde | IFBD | 2.37 | 6.4 | 170.04% | 0.68(0.6-0.77) | 0.81 | 1.11 | 37.04% | -2.05(-2.56 - -1.54) | 33.75 | 43.28 | 28.24% | -2.12(-2.5 - -1.74) |
| Cabo Verde | PACA | 59.84 | 128.31 | 114.42% | 0.09(0.06-0.13) | 4.6 | 12.01 | 161.09% | 0.17(-0.03-0.37) | 158.52 | 414.7 | 161.61% | 0.15(-0.05-0.34) |
| Cabo Verde | PIIO | 422.8 | 1187.79 | 180.94% | 1.84(1.67-2.01) | 10.88 | 17.4 | 59.86% | -0.66(-0.93 - -0.38) | 441.23 | 469.45 | 6.39% | -1.29(-1.49 - -1.09) |
| Cabo Verde | UDSD | 11713.54 | 25375.62 | 116.63% | -0.01(-0.021-0) | 22.39 | 12.74 | -43.10% | -4.02(-4.69 - -3.34) | 890.91 | 834.72 | -6.31% | -2.54(-2.98 - -2.1) |
| Cabo Verde | VAID | 20.44 | 49.72 | 143.25% | 1.53(1.47-1.59) | 1.35 | 3.31 | 145.19% | 0.51(0.03-0.99) | 33.27 | 67.68 | 103.43% | -0.03(-0.45-0.39) |
| Cambodia | Digestive diseases | 245908.76 | 521471.6 | 112.06% | 0.06(0.04-0.07) | 7864.83 | 12187.8 | 54.97% | -1.44(-1.53 - -1.36) | 332220.37 | 407786.25 | 22.75% | -1.78(-1.88 - -1.68) |
| Cambodia | APED | 25561.78 | 46189.41 | 80.70% | 0.46(0.22-0.71) | 119.48 | 73.74 | -38.28% | -3.14(-3.34 - -2.94) | 8107.65 | 4054.2 | -50.00% | -3.58(-3.82 - -3.34) |
| Cambodia | COCLD | 3738.87 | 8387.84 | 124.34% | 0.21(0.17-0.25) | 4703.57 | 8387.77 | 78.33% | -0.98(-1.04 - -0.92) | 188682.72 | 282662.93 | 49.81% | -1.37(-1.43 - -1.3) |
| Cambodia | GABD | 13431.37 | 34109.84 | 153.96% | 0.66(0.63-0.69) | 149.78 | 284.41 | 89.89% | -0.75(-0.83 - -0.66) | 7762.45 | 11362.84 | 46.38% | -1.37(-1.45 - -1.29) |
| Cambodia | IFAH | 5378.97 | 15401.96 | 186.34% | 1.05(0.93-1.16) | 137.49 | 92.4 | -32.80% | -2.13(-2.25 - -2.01) | 11319.22 | 6644.64 | -41.30% | -2.52(-2.7 - -2.34) |
| Cambodia | IFBD | 30.09 | 79.3 | 163.54% | 1.06(1.01-1.12) | 25.79 | 37.76 | 46.41% | -1.44(-1.54 - -1.34) | 1175.08 | 1221.73 | 3.97% | -1.86(-1.96 - -1.77) |
| Cambodia | PACA | 1641.98 | 3590.08 | 118.64% | -0.05(-0.06 - -0.04) | 99.04 | 177.61 | 79.33% | -0.91(-0.95 - -0.87) | 4003.95 | 6153.2 | 53.68% | -1.25(-1.29 - -1.21) |
| Cambodia | PIIO | 5069.45 | 13670.21 | 169.66% | 1.51(1.41-1.6) | 535.44 | 635.88 | 18.76% | -0.38(-0.54 - -0.21) | 37195.77 | 26608 | -28.46% | -1.38(-1.6 - -1.15) |
| Cambodia | UDSD | 190787.57 | 399207.43 | 109.24% | -0.108(-0.12 - -0.097) | 1970.94 | 2254.26 | 14.37% | -2.75(-2.93 - -2.56) | 68869.39 | 62744.68 | -8.89% | -3.1(-3.28 - -2.93) |
| Cambodia | VAID | 268.69 | 835.53 | 210.96% | 1.47(1.36-1.59) | 22.22 | 69.61 | 213.28% | 0.55(0.47-0.62) | 529.96 | 1335.3 | 151.96% | 0.19(0.12-0.25) |
| Cameroon | Digestive diseases | 367660.23 | 1135493.9 | 208.84% | 0.07(0.05-0.08) | 3757.62 | 7136.6 | 89.92% | -1.5(-1.72 - -1.29) | 150540.91 | 306139.8 | 103.36% | -1.3(-1.49 - -1.1) |
| Cameroon | APED | 12137.23 | 48314.19 | 298.07% | 1.05(0.87-1.23) | 55.79 | 54.89 | -1.61% | -3.44(-3.78 - -3.11) | 3082.05 | 3184.72 | 3.33% | -3.62(-3.94 - -3.3) |
| Cameroon | COCLD | 2237.79 | 6303.86 | 181.70% | -0.08(-0.16-0) | 2283.19 | 4130.79 | 80.92% | -1.75(-1.98 - -1.52) | 79548.37 | 153174.56 | 92.56% | -1.57(-1.79 - -1.34) |
| Cameroon | GABD | 3014 | 10185.1 | 237.93% | 0.6(0.57-0.63) | 100.79 | 256.25 | 154.24% | -0.27(-0.52 - -0.03) | 3876.63 | 9644.62 | 148.79% | -0.39(-0.62 - -0.17) |
| Cameroon | IFAH | 11321.7 | 37130.49 | 227.96% | 0.9(0.8-1.01) | 61.28 | 123.34 | 101.27% | -1.07(-1.32 - -0.83) | 5724.3 | 13920.2 | 143.18% | -0.29(-0.4 - -0.19) |
| Cameroon | IFBD | 79.99 | 265.02 | 231.32% | 0.21(0.05-0.37) | 29.51 | 57.56 | 95.05% | -1.69(-1.94 - -1.43) | 1366.75 | 2830 | 107.06% | -1.43(-1.66 - -1.2) |
| Cameroon | PACA | 1726.15 | 5125.06 | 196.91% | -0.03(-0.05 - -0.01) | 188.76 | 524.85 | 178.05% | -0.44(-0.66 - -0.21) | 7054.67 | 20701.06 | 193.44% | -0.25(-0.47 - -0.03) |
| Cameroon | PIIO | 7950.57 | 27708.43 | 248.51% | 0.92(0.79-1.06) | 366.32 | 799.91 | 118.36% | -0.92(-1.14 - -0.7) | 17524.57 | 36521.83 | 108.40% | -0.82(-1.01 - -0.63) |
| Cameroon | UDSD | 328699.06 | 998797.71 | 203.86% | -0.006(-0.012-0) | 448.6 | 758.27 | 69.03% | -1.6(-1.84 - -1.37) | 23253.46 | 49623.67 | 113.40% | -1.15(-1.33 - -0.98) |
| Cameroon | VAID | 493.75 | 1664.04 | 237.02% | 1.09(0.98-1.21) | 34.24 | 103.27 | 201.61% | 0.7(0.53-0.88) | 1284.1 | 3318.39 | 158.42% | 0.45(0.29-0.61) |
| Canada | Digestive diseases | 1431031.58 | 2240002.25 | 56.53% | 0.06(0.05-0.08) | 7296.42 | 12871.95 | 76.41% | -0.97(-1.02 - -0.91) | 219068.15 | 330573.43 | 50.90% | -0.62(-0.68 - -0.55) |
| Canada | APED | 49025.62 | 62888.82 | 28.28% | 0.07(0.05-0.08) | 48.15 | 72.39 | 50.34% | -1.18(-1.39 - -0.98) | 1688.54 | 2045.74 | 21.15% | -0.75(-0.86 - -0.64) |
| Canada | COCLD | 6170.25 | 7771.62 | 25.95% | -0.21(-0.26 - -0.16) | 3144.26 | 5442.01 | 73.08% | -0.64(-0.72 - -0.56) | 82563.68 | 129216.46 | 56.51% | -0.54(-0.65 - -0.43) |
| Canada | GABD | 195298.76 | 329455.42 | 68.69% | 0.14(0.12-0.16) | 470.12 | 894.31 | 90.23% | -0.79(-0.94 - -0.64) | 21823.65 | 33734.19 | 54.58% | -0.46(-0.53 - -0.38) |
| Canada | IFAH | 28694.65 | 46193.24 | 60.98% | -0.17(-0.19 - -0.16) | 191.67 | 389.01 | 102.96% | -0.58(-0.68 - -0.47) | 7902.26 | 11907.43 | 50.68% | -0.67(-0.73 - -0.6) |
| Canada | IFBD | 11089.42 | 14206.11 | 28.11% | 0.28(0.12-0.44) | 270.37 | 403.27 | 49.15% | -2.43(-3.11 - -1.75) | 22693.55 | 29915.34 | 31.82% | -0.31(-0.41 - -0.21) |
| Canada | PACA | 17772.05 | 29038.28 | 63.39% | -0.09(-0.09 - -0.08) | 298.02 | 545.8 | 83.14% | -0.93(-1.06 - -0.79) | 7886.69 | 12203.58 | 54.74% | -0.72(-0.78 - -0.65) |
| Canada | PIIO | 75717.24 | 157947.45 | 108.60% | 0.71(0.65-0.77) | 563.88 | 1481.97 | 162.82% | 0.17(0.04-0.3) | 10401.61 | 21027.71 | 102.16% | 0.01(-0.08-0.11) |
| Canada | UDSD | 1029283.24 | 1555145 | 51.09% | -0.001(-0.013-0.01) | 1054.64 | 786.41 | -25.43% | -4.58(-5.02 - -4.13) | 42096.31 | 47506.92 | 12.85% | -1.37(-1.55 - -1.18) |
| Canada | VAID | 17980.34 | 37356.31 | 107.76% | 0.4(0.31-0.48) | 699.08 | 1410.13 | 101.71% | -0.63(-0.72 - -0.54) | 12059.33 | 21773.39 | 80.55% | -0.57(-0.65 - -0.5) |
| Central African Republic | Digestive diseases | 108224.54 | 219464.26 | 102.79% | 0.08(0.07-0.09) | 1406.64 | 2202.33 | 56.57% | -0.54(-0.61 - -0.47) | 58796.83 | 92647.4 | 57.57% | -0.63(-0.68 - -0.57) |
| Central African Republic | APED | 6744.16 | 14416.03 | 113.76% | 0.29(0.21-0.36) | 39.83 | 66.98 | 68.16% | -0.22(-0.28 - -0.16) | 2207.85 | 3643.91 | 65.04% | -0.29(-0.35 - -0.23) |
| Central African Republic | COCLD | 667.49 | 1359.62 | 103.69% | 0.03(-0.01-0.07) | 740.65 | 1105.26 | 49.23% | -0.9(-0.99 - -0.81) | 26984.37 | 41112.95 | 52.36% | -0.92(-1 - -0.84) |
| Central African Republic | GABD | 792.52 | 1667.49 | 110.40% | 0.37(0.33-0.42) | 56.78 | 111.09 | 95.65% | 0.49(0.37-0.6) | 1952.67 | 3768.83 | 93.01% | 0.31(0.21-0.42) |
| Central African Republic | IFAH | 3357.94 | 6436.13 | 91.67% | 0.26(0.16-0.36) | 40.75 | 62.97 | 54.53% | 0.06(0.04-0.08) | 3318.63 | 5178.61 | 56.05% | -0.08(-0.12 - -0.04) |
| Central African Republic | IFBD | 20.68 | 42.36 | 104.84% | 0.12(0.1-0.14) | 11.51 | 20.75 | 80.28% | 0.02(-0.06-0.1) | 509.88 | 889.63 | 74.48% | -0.07(-0.13-0) |
| Central African Republic | PACA | 397.77 | 765.92 | 92.55% | -0.1(-0.11 - -0.1) | 35.04 | 60.19 | 71.78% | -0.45(-0.5 - -0.41) | 1330.26 | 2346.8 | 76.42% | -0.47(-0.53 - -0.43) |
| Central African Republic | PIIO | 2086.47 | 3452.77 | 65.48% | -0.22(-0.27 - -0.17) | 133 | 211.24 | 58.83% | -0.31(-0.35 - -0.28) | 6575.14 | 9688.67 | 47.35% | -0.48(-0.54 - -0.41) |
| Central African Republic | UDSD | 94028.61 | 191077.7 | 103.21% | 0.07(0.067-0.074) | 251.67 | 404.21 | 60.61% | -0.29(-0.36 - -0.22) | 12266.86 | 20267.99 | 65.23% | -0.46(-0.5 - -0.42) |
| Central African Republic | VAID | 128.88 | 246.23 | 91.05% | 0.04(0-0.09) | 18.93 | 32.51 | 71.74% | -0.05(-0.16-0.05) | 548.1 | 933.31 | 70.28% | -0.17(-0.27 - -0.08) |
| Chad | Digestive diseases | 210043.53 | 537009.88 | 155.67% | 0.03(0.02-0.03) | 2631.51 | 5147.36 | 95.60% | -0.27(-0.42 - -0.12) | 106121.11 | 225540.37 | 112.53% | -0.25(-0.4 - -0.11) |
| Chad | APED | 7220.34 | 24295.51 | 236.49% | 0.52(0.29-0.75) | 57.52 | 62.3 | 8.31% | -2.38(-2.67 - -2.09) | 3276.58 | 3665.17 | 11.86% | -2.91(-3.19 - -2.63) |
| Chad | COCLD | 1360.67 | 3690.29 | 171.21% | -0.09(-0.15 - -0.03) | 1429 | 2885.6 | 101.93% | -0.2(-0.31 - -0.09) | 48476.01 | 108481.5 | 123.78% | -0.08(-0.2-0.03) |
| Chad | GABD | 1703.83 | 4871.47 | 185.91% | 0.32(0.3-0.34) | 76.43 | 168.13 | 119.98% | 0.45(0.25-0.64) | 2951.79 | 7051.53 | 138.89% | 0.2(0-0.4) |
| Chad | IFAH | 7242.02 | 20505.95 | 183.15% | 0.61(0.53-0.69) | 59.09 | 110.4 | 86.83% | -0.16(-0.41-0.09) | 4794.77 | 10605.05 | 121.18% | -0.06(-0.21-0.1) |
| Chad | IFBD | 35.35 | 96.69 | 173.52% | 0.18(0.03-0.33) | 20.87 | 43.6 | 108.91% | -0.24(-0.45 - -0.04) | 959.74 | 2083.54 | 117.09% | -0.36(-0.55 - -0.16) |
| Chad | PACA | 983.08 | 2435.33 | 147.72% | -0.03(-0.05-0) | 107.94 | 261.85 | 142.59% | 0.1(-0.15-0.36) | 3952.67 | 10255.58 | 159.46% | 0.18(-0.09-0.44) |
| Chad | PIIO | 4119.57 | 10944.27 | 165.67% | 0.36(0.24-0.48) | 342.84 | 703.31 | 105.15% | -0.09(-0.27-0.09) | 17091.76 | 35579.88 | 108.17% | -0.32(-0.47 - -0.17) |
| Chad | UDSD | 187085.26 | 469279.63 | 150.84% | -0.015(-0.019 - -0.012) | 386.93 | 671.52 | 73.55% | -0.5(-0.76 - -0.24) | 18302.52 | 37419.67 | 104.45% | -0.47(-0.7 - -0.24) |
| Chad | VAID | 293.39 | 890.72 | 203.60% | 0.73(0.67-0.79) | 30.06 | 62.55 | 108.08% | 0.57(0.42-0.71) | 1219.14 | 2325.6 | 90.76% | 0.11(-0.02-0.23) |
| Chile | Digestive diseases | 748513.9 | 1304429.78 | 74.27% | 0.03(0-0.06) | 5984.44 | 8426.25 | 40.80% | -1.71(-1.82 - -1.6) | 198418.57 | 235910.06 | 18.90% | -1.8(-1.93 - -1.66) |
| Chile | APED | 29680.23 | 61126.19 | 105.95% | 1.77(1.46-2.09) | 72.26 | 53.35 | -26.17% | -3.87(-4.09 - -3.64) | 3037.43 | 2134.47 | -29.73% | -2.86(-3.04 - -2.68) |
| Chile | COCLD | 4865.21 | 8553.36 | 75.81% | -0.27(-0.45 - -0.1) | 3929.52 | 5157.32 | 31.25% | -1.71(-1.84 - -1.59) | 125571.83 | 138437.89 | 10.25% | -2.02(-2.18 - -1.86) |
| Chile | GABD | 23431.97 | 40283.04 | 71.91% | -0.43(-0.51 - -0.36) | 487.07 | 570.61 | 17.15% | -2.95(-3.25 - -2.65) | 12649.4 | 12430.41 | -1.73% | -2.97(-3.27 - -2.67) |
| Chile | IFAH | 17135.76 | 37574.54 | 119.28% | 0.55(0.26-0.85) | 137.97 | 200.44 | 45.28% | -1.87(-2.15 - -1.59) | 7596.23 | 10117.05 | 33.19% | -1.09(-1.13 - -1.06) |
| Chile | IFBD | 313.74 | 485.43 | 54.72% | 0.43(0.2-0.66) | 45.62 | 70.07 | 53.59% | -1.75(-2.09 - -1.4) | 1928 | 2611.52 | 35.45% | -1.09(-1.28 - -0.91) |
| Chile | PACA | 3720.52 | 7360.5 | 97.84% | -0.11(-0.17 - -0.06) | 243.44 | 388.24 | 59.48% | -1.02(-1.14 - -0.9) | 7875.61 | 9956.23 | 26.42% | -1.43(-1.6 - -1.26) |
| Chile | PIIO | 9507.06 | 29105.8 | 206.15% | 1.56(1.43-1.69) | 288.77 | 770.84 | 166.94% | 0.16(0.04-0.27) | 6873.64 | 12618.92 | 83.58% | -0.29(-0.4 - -0.19) |
| Chile | UDSD | 658070.29 | 1114660.16 | 69.38% | -0.09(-0.112 - -0.069) | 407.77 | 311.83 | -23.53% | -4.45(-4.66 - -4.24) | 24833.6 | 31606.89 | 27.27% | -1.53(-1.64 - -1.41) |
| Chile | VAID | 1789.14 | 5280.76 | 195.16% | 0.09(-0.32-0.49) | 259.26 | 537.55 | 107.34% | -1.03(-1.15 - -0.91) | 4982.8 | 8830.96 | 77.23% | -1.16(-1.24 - -1.08) |
| China | Digestive diseases | 40981204.96 | 68153740.92 | 66.30% | -0.01(-0.08-0.07) | 322745.7 | 277141.98 | -14.13% | -3.38(-3.53 - -3.24) | 13131185.51 | 10017880.42 | -23.71% | -3.25(-3.34 - -3.17) |
| China | APED | 2127289.42 | 2486787.15 | 16.90% | 0.44(0.27-0.61) | 4002.95 | 1565.58 | -60.89% | -6.06(-6.35 - -5.76) | 175669.21 | 65126.64 | -62.93% | -4.96(-5.16 - -4.76) |
| China | COCLD | 310191.32 | 409693.64 | 32.08% | -0.52(-0.62 - -0.42) | 167670.71 | 152261.88 | -9.19% | -3.14(-3.33 - -2.95) | 5698267.54 | 4343006.24 | -23.78% | -3.47(-3.64 - -3.3) |
| China | GABD | 7551312.79 | 18642298.7 | 146.87% | 1.23(1.03-1.44) | 18652.93 | 16017.3 | -14.13% | -3.99(-4.12 - -3.85) | 1426017.22 | 1671671.35 | 17.23% | -2(-2.14 - -1.85) |
| China | IFAH | 1017192.27 | 1946932.31 | 91.40% | 1.02(0.96-1.08) | 2832.7 | 1625.61 | -42.61% | -4.31(-4.45 - -4.16) | 370235.23 | 288731.6 | -22.01% | -2.04(-2.12 - -1.97) |
| China | IFBD | 17221.41 | 51461.96 | 198.83% | 2.54(2.39-2.69) | 5559.54 | 4675.97 | -15.89% | -4.11(-4.5 - -3.71) | 234245.17 | 232463.85 | -0.76% | -2.36(-2.54 - -2.18) |
| China | PACA | 380018.33 | 493765.44 | 29.93% | -1.35(-1.67 - -1.02) | 8975.82 | 10663.58 | 18.80% | -2.01(-2.07 - -1.94) | 320704.63 | 301309.93 | -6.05% | -2.32(-2.37 - -2.28) |
| China | PIIO | 932592.19 | 1683762.5 | 80.55% | 0.35(0.21-0.49) | 21751.98 | 15476.85 | -28.85% | -3.26(-3.35 - -3.17) | 1225816.29 | 391903.84 | -68.03% | -4.55(-4.71 - -4.39) |
| China | UDSD | 28590235.03 | 42328194.66 | 48.05% | -0.518(-0.645 - -0.391) | 71725.02 | 58691.69 | -18.17% | -3.4(-3.64 - -3.16) | 2834505.11 | 2324765.48 | -17.98% | -3.01(-3.14 - -2.87) |
| China | VAID | 55152.2 | 110844.58 | 100.98% | -0.47(-0.65 - -0.28) | 2925.38 | 6412.81 | 119.21% | -0.68(-0.74 - -0.62) | 66074.5 | 118664.86 | 79.59% | -0.93(-0.98 - -0.87) |
| Colombia | Digestive diseases | 2178741.74 | 4153196.95 | 90.62% | 0.09(0.09-0.1) | 5889.23 | 11497.24 | 95.22% | -1.68(-1.83 - -1.53) | 272517.48 | 404012.47 | 48.25% | -1.41(-1.51 - -1.32) |
| Colombia | APED | 133572.61 | 199206.72 | 49.14% | 0.57(0.53-0.62) | 180.96 | 319.09 | 76.33% | -0.89(-1.18 - -0.6) | 9383.1 | 11703.16 | 24.73% | -0.78(-0.98 - -0.58) |
| Colombia | COCLD | 4961.92 | 7858.12 | 58.37% | -0.77(-0.83 - -0.71) | 2098.29 | 3984.48 | 89.89% | -1.96(-2.16 - -1.76) | 67748.83 | 96342.91 | 42.21% | -2.22(-2.39 - -2.04) |
| Colombia | GABD | 219169.57 | 502908.09 | 129.46% | 0.6(0.56-0.64) | 439.03 | 1295.82 | 195.16% | 0.01(-0.24-0.27) | 37507.37 | 70910.99 | 89.06% | -0.46(-0.55 - -0.37) |
| Colombia | IFAH | 67158.37 | 130340.77 | 94.08% | -0.02(-0.14-0.1) | 231.47 | 410.41 | 77.31% | -1.94(-2.24 - -1.65) | 22816.67 | 32293.46 | 41.53% | -1.2(-1.35 - -1.06) |
| Colombia | IFBD | 598.47 | 1128.91 | 88.63% | -0.16(-0.34-0.01) | 55.89 | 154.12 | 175.76% | 0.75(0.38-1.12) | 3215.04 | 6419.29 | 99.66% | 0.36(0.12-0.6) |
| Colombia | PACA | 9861.31 | 18313.63 | 85.71% | -0.23(-0.25 - -0.21) | 274.98 | 530.97 | 93.09% | -1.44(-1.56 - -1.32) | 10016.58 | 14689.87 | 46.66% | -1.66(-1.77 - -1.54) |
| Colombia | PIIO | 40801.03 | 78187.61 | 91.63% | 0.44(0.37-0.51) | 654.33 | 1475.12 | 125.44% | -0.11(-0.34-0.12) | 33044.58 | 36951.47 | 11.82% | -0.79(-0.96 - -0.63) |
| Colombia | UDSD | 1699699.71 | 3205731 | 88.61% | -0.013(-0.017 - -0.01) | 1389.72 | 1273.61 | -8.35% | -4.95(-5.18 - -4.72) | 72209.03 | 93468.56 | 29.44% | -2.08(-2.26 - -1.9) |
| Colombia | VAID | 2918.75 | 9522.1 | 226.24% | 0.76(0.7-0.83) | 330.11 | 1079.69 | 227.07% | -0.3(-0.57 - -0.02) | 7797.03 | 19056.35 | 144.41% | -0.56(-0.76 - -0.36) |
| Comoros | Digestive diseases | 17103.53 | 34930.48 | 104.23% | 0.11(0.1-0.11) | 171.89 | 298.6 | 73.72% | -0.81(-0.97 - -0.66) | 6268.32 | 9946.92 | 58.69% | -0.91(-1.11 - -0.7) |
| Comoros | APED | 691.71 | 1386.55 | 100.45% | 1(0.93-1.07) | 4.39 | 4.83 | 10.02% | -1.87(-2.09 - -1.66) | 222.87 | 205.69 | -7.71% | -2(-2.32 - -1.69) |
| Comoros | COCLD | 84.19 | 165.02 | 96.01% | 0.26(0.19-0.33) | 89.03 | 161.14 | 81.00% | -0.79(-0.96 - -0.62) | 2795.97 | 4865.34 | 74.01% | -0.9(-1.13 - -0.67) |
| Comoros | GABD | 139.23 | 320.45 | 130.16% | 0.93(0.87-1) | 10.39 | 22.01 | 111.84% | -0.14(-0.25 - -0.03) | 284.35 | 525.36 | 84.76% | -0.43(-0.57 - -0.29) |
| Comoros | IFAH | 328.04 | 718.98 | 119.17% | 0.94(0.89-1) | 5.39 | 8.03 | 48.98% | -0.69(-0.76 - -0.62) | 325.51 | 398.02 | 22.28% | -0.64(-0.69 - -0.58) |
| Comoros | IFBD | 3.06 | 7.11 | 132.35% | 0.36(0.28-0.44) | 2 | 3.62 | 81.00% | -0.51(-0.67 - -0.36) | 76.89 | 118.86 | 54.58% | -0.63(-0.84 - -0.41) |
| Comoros | PACA | 65.34 | 124.94 | 91.22% | -0.13(-0.14 - -0.12) | 4.32 | 7.53 | 74.31% | -1.03(-1.22 - -0.84) | 143.34 | 249.35 | 73.96% | -1.06(-1.33 - -0.78) |
| Comoros | PIIO | 351.2 | 853.24 | 142.95% | 1.31(1.23-1.38) | 18.96 | 34.05 | 79.59% | -0.27(-0.41 - -0.13) | 850.78 | 1218.15 | 43.18% | -0.39(-0.6 - -0.17) |
| Comoros | UDSD | 15417.04 | 31301.23 | 103.03% | 0.023(0.02-0.026) | 24.32 | 31.69 | 30.30% | -1.87(-2.07 - -1.67) | 1186.54 | 1731.51 | 45.93% | -1.39(-1.58 - -1.21) |
| Comoros | VAID | 23.72 | 52.94 | 123.19% | 0.96(0.89-1.03) | 3.33 | 7.38 | 121.62% | -0.14(-0.24 - -0.03) | 75.35 | 152.37 | 102.22% | -0.27(-0.41 - -0.14) |
| Congo | Digestive diseases | 91839.3 | 240448.06 | 161.81% | 0.15(0.13-0.17) | 1056.28 | 1722.37 | 63.06% | -1.24(-1.38 - -1.1) | 41810.71 | 66545.49 | 59.16% | -1.44(-1.57 - -1.31) |
| Congo | APED | 5154.52 | 15089.99 | 192.75% | 1.38(1.18-1.58) | 19.7 | 20.94 | 6.29% | -2.31(-2.55 - -2.08) | 1091.28 | 1116.79 | 2.34% | -2.49(-2.72 - -2.25) |
| Congo | COCLD | 426.42 | 1022.14 | 139.70% | 0.12(0.01-0.22) | 651.98 | 1070.65 | 64.22% | -1.4(-1.53 - -1.27) | 23094.29 | 37357.59 | 61.76% | -1.6(-1.74 - -1.46) |
| Congo | GABD | 708.32 | 1980.57 | 179.62% | 0.82(0.75-0.88) | 40.56 | 86.2 | 112.52% | 0.07(-0.12-0.27) | 1289.4 | 2486.74 | 92.86% | -0.37(-0.55 - -0.18) |
| Congo | IFAH | 2281.24 | 6046.65 | 165.06% | 0.99(0.87-1.12) | 20.55 | 29.44 | 43.26% | -0.87(-1.02 - -0.71) | 1676.77 | 2645.39 | 57.77% | -0.7(-0.77 - -0.63) |
| Congo | IFBD | 18.27 | 60.12 | 229.06% | 0.81(0.7-0.93) | 9.1 | 17.08 | 87.69% | -0.58(-0.75 - -0.41) | 395.55 | 681.18 | 72.21% | -0.82(-0.99 - -0.66) |
| Congo | PACA | 331.69 | 811.98 | 144.80% | -0.1(-0.1 - -0.09) | 23.62 | 40.94 | 73.33% | -1.33(-1.42 - -1.23) | 892.37 | 1530.65 | 71.53% | -1.56(-1.68 - -1.45) |
| Congo | PIIO | 2245.52 | 6342.63 | 182.46% | 1.04(0.9-1.19) | 85.36 | 152.55 | 78.70% | -0.71(-0.82 - -0.6) | 4040.3 | 6241.08 | 54.47% | -0.96(-1.08 - -0.84) |
| Congo | UDSD | 80533.61 | 208699.44 | 159.15% | 0.046(0.039-0.054) | 129.61 | 167.93 | 29.57% | -1.72(-1.95 - -1.49) | 6701.7 | 10385.56 | 54.97% | -1.54(-1.66 - -1.42) |
| Congo | VAID | 139.72 | 394.55 | 182.39% | 1.01(0.88-1.15) | 17.86 | 37.42 | 109.52% | -0.27(-0.4 - -0.14) | 473.77 | 902.93 | 90.58% | -0.6(-0.71 - -0.49) |
| Cook Islands | Digestive diseases | 492.33 | 630.91 | 28.15% | 0.09(0.08-0.1) | 4.02 | 4.53 | 12.54% | -1.85(-2 - -1.7) | 140 | 133.64 | -4.54% | -1.6(-1.76 - -1.43) |
| Cook Islands | APED | 19.37 | 19.75 | 1.96% | 0.5(0.46-0.55) | 0.03 | 0.02 | -33.33% | -2.91(-3.17 - -2.66) | 1.16 | 0.78 | -32.76% | -1.93(-2.18 - -1.68) |
| Cook Islands | COCLD | 1.02 | 1.07 | 4.90% | 0.04(-0.01-0.09) | 1.88 | 1.99 | 5.85% | -1.88(-2.05 - -1.71) | 61.23 | 56.59 | -7.58% | -1.81(-2.01 - -1.6) |
| Cook Islands | GABD | 26.81 | 39.05 | 45.65% | 0.28(0.25-0.31) | 0.56 | 0.74 | 32.14% | -1.48(-1.59 - -1.38) | 17.57 | 18.71 | 6.49% | -1.39(-1.52 - -1.27) |
| Cook Islands | IFAH | 13.54 | 21.27 | 57.09% | 0.6(0.51-0.7) | 0.15 | 0.16 | 6.67% | -2.46(-2.65 - -2.27) | 6.58 | 6.82 | 3.65% | -1.19(-1.36 - -1.02) |
| Cook Islands | IFBD | 0.09 | 0.15 | 66.67% | 1.18(1.06-1.31) | 0.21 | 0.18 | -14.29% | -2.7(-2.9 - -2.5) | 7.66 | 5.08 | -33.68% | -2.7(-2.88 - -2.51) |
| Cook Islands | PACA | 4.05 | 5.51 | 36.05% | -0.09(-0.09 - -0.09) | 0.36 | 0.39 | 8.33% | -1.4(-1.53 - -1.26) | 13.58 | 11.91 | -12.30% | -1.29(-1.45 - -1.12) |
| Cook Islands | PIIO | 16.88 | 25.51 | 51.07% | 0.64(0.61-0.66) | 0.2 | 0.32 | 58.26% | -0.95(-1.09 - -0.8) | 5.82 | 6 | 3.20% | -1.24(-1.37 - -1.1) |
| Cook Islands | UDSD | 409.57 | 516.48 | 26.10% | 0.013(0.005-0.021) | 0.41 | 0.3 | -26.83% | -4.04(-4.23 - -3.85) | 20.4 | 18.67 | -8.48% | -1.72(-1.85 - -1.58) |
| Cook Islands | VAID | 1.01 | 2.12 | 109.90% | 1.13(1.08-1.17) | 0.13 | 0.25 | 92.31% | -0.37(-0.49 - -0.26) | 3.12 | 5.07 | 62.50% | -0.38(-0.48 - -0.29) |
| Costa Rica | Digestive diseases | 206569.86 | 413592.15 | 100.22% | 0.04(0.02-0.05) | 610.72 | 1775.57 | 190.74% | -0.56(-0.83 - -0.29) | 25057.2 | 56576.82 | 125.79% | -0.58(-0.77 - -0.39) |
| Costa Rica | APED | 13708.47 | 19117.89 | 39.46% | 0.16(0.1-0.23) | 15.64 | 24.1 | 54.09% | -1.72(-2.24 - -1.2) | 818.97 | 940.04 | 14.78% | -1.42(-1.87 - -0.98) |
| Costa Rica | COCLD | 762.15 | 1575.2 | 106.68% | 0(-0.04-0.04) | 317.21 | 959.61 | 202.52% | -0.34(-0.61 - -0.06) | 10159.11 | 26139.74 | 157.30% | -0.58(-0.85 - -0.31) |
| Costa Rica | GABD | 21764.58 | 48762.78 | 124.05% | 0.27(0.22-0.32) | 41.68 | 111.07 | 166.48% | -0.81(-1.19 - -0.44) | 3010.82 | 6194.09 | 105.73% | -0.54(-0.72 - -0.36) |
| Costa Rica | IFAH | 6080.52 | 12489.43 | 105.40% | 0.41(0.32-0.5) | 11.59 | 37.91 | 227.09% | 0.43(0.18-0.69) | 1678.23 | 3023.57 | 80.16% | -0.09(-0.2-0.03) |
| Costa Rica | IFBD | 63 | 119.95 | 90.40% | -0.46(-0.59 - -0.33) | 2.69 | 9.97 | 270.63% | 1.04(0.82-1.26) | 189.9 | 524.42 | 176.16% | 0.58(0.48-0.69) |
| Costa Rica | PACA | 1045.82 | 2067.19 | 97.66% | -0.2(-0.21 - -0.19) | 44.22 | 104.53 | 136.39% | -1.17(-1.47 - -0.86) | 1419.4 | 2869.14 | 102.14% | -1.2(-1.48 - -0.91) |
| Costa Rica | PIIO | 5359.43 | 8267.05 | 54.25% | -0.16(-0.3 - -0.01) | 37.5 | 98.4 | 162.42% | -0.32(-0.48 - -0.16) | 1393.67 | 2147.74 | 54.11% | -0.63(-0.76 - -0.5) |
| Costa Rica | UDSD | 157368.94 | 319966.44 | 103.32% | -0.015(-0.017 - -0.013) | 72.3 | 111.13 | 53.71% | -3.64(-4.14 - -3.14) | 4949.73 | 9160.88 | 85.08% | -1.14(-1.3 - -0.97) |
| Costa Rica | VAID | 416.96 | 1226.21 | 194.08% | 0.44(0.36-0.53) | 56.13 | 204.98 | 265.19% | 0.02(-0.25-0.29) | 982.23 | 3081.27 | 213.70% | -0.14(-0.36-0.08) |
| Côte d'Ivoire | Digestive diseases | 419635.42 | 1041098.8 | 148.10% | 0.06(0.05-0.08) | 3668.78 | 6623.49 | 80.54% | -1.27(-1.48 - -1.06) | 169017.96 | 290052.89 | 71.61% | -1.21(-1.4 - -1.02) |
| Côte d'Ivoire | APED | 14341.81 | 40062.36 | 179.34% | 0.92(0.73-1.11) | 55.93 | 52.4 | -6.31% | -2.62(-2.95 - -2.3) | 3507.28 | 3019.97 | -13.89% | -3.06(-3.38 - -2.73) |
| Côte d'Ivoire | COCLD | 2503.97 | 6084.01 | 142.97% | 0.07(-0.12-0.27) | 2280.36 | 3947.55 | 73.11% | -1.57(-1.77 - -1.37) | 87688.02 | 148383.52 | 69.22% | -1.48(-1.67 - -1.28) |
| Côte d'Ivoire | GABD | 3392.42 | 8733.2 | 157.43% | 0.51(0.49-0.53) | 76.92 | 208.85 | 171.52% | 0.16(-0.14-0.46) | 3759.89 | 8353.26 | 122.17% | -0.06(-0.34-0.22) |
| Côte d'Ivoire | IFAH | 13467.13 | 36113.9 | 168.16% | 0.75(0.63-0.86) | 65.34 | 117.42 | 79.71% | -0.85(-1.13 - -0.57) | 7249.71 | 13668.28 | 88.54% | -0.33(-0.44 - -0.21) |
| Côte d'Ivoire | IFBD | 84.52 | 241.64 | 185.90% | 0.31(0.19-0.43) | 25.49 | 54.03 | 111.97% | -0.71(-0.97 - -0.45) | 1323.78 | 2582.84 | 95.11% | -0.63(-0.86 - -0.4) |
| Côte d'Ivoire | PACA | 1815.31 | 4524.16 | 149.22% | 0.03(0.01-0.05) | 169.1 | 442.5 | 161.68% | -0.42(-0.73 - -0.1) | 6894.06 | 17731.16 | 157.19% | -0.39(-0.72 - -0.07) |
| Côte d'Ivoire | PIIO | 8874.9 | 22686.65 | 155.63% | 0.5(0.4-0.6) | 405.34 | 725.35 | 78.95% | -0.56(-0.76 - -0.36) | 24013.59 | 33727.13 | 40.45% | -0.94(-1.16 - -0.71) |
| Côte d'Ivoire | UDSD | 374516.04 | 921030.06 | 145.93% | 0.006(0.002-0.01) | 409.8 | 749.17 | 82.81% | -1.09(-1.41 - -0.78) | 25721.23 | 49398.93 | 92.06% | -0.91(-1.17 - -0.65) |
| Côte d'Ivoire | VAID | 639.32 | 1622.82 | 153.84% | 0.67(0.57-0.76) | 34.94 | 81.23 | 132.48% | 0.11(-0.15-0.36) | 1692.79 | 2799.42 | 65.37% | -0.35(-0.59 - -0.11) |
| Croatia | Digestive diseases | 341890.85 | 350202.24 | 2.43% | 0.08(0.05-0.11) | 2871.62 | 2233.57 | -22.22% | -2.01(-2.18 - -1.83) | 95092.41 | 62548.54 | -34.22% | -2.03(-2.17 - -1.88) |
| Croatia | APED | 12620.09 | 9297.11 | -26.33% | -0.04(-0.09-0.01) | 17.3 | 11.02 | -36.30% | -2.56(-2.94 - -2.18) | 535.77 | 303.62 | -43.33% | -1.65(-1.83 - -1.47) |
| Croatia | COCLD | 2404.64 | 1532.5 | -36.27% | -1.19(-1.29 - -1.09) | 1882.7 | 1150.73 | -38.88% | -2.6(-2.84 - -2.37) | 58361.4 | 29869.21 | -48.82% | -2.96(-3.2 - -2.73) |
| Croatia | GABD | 58666.39 | 69072.3 | 17.74% | 0.47(0.35-0.59) | 98.98 | 133.05 | 34.42% | -0.08(-0.43-0.27) | 6849.34 | 6902.73 | 0.78% | -0.28(-0.36 - -0.21) |
| Croatia | IFAH | 11937.89 | 13450.24 | 12.67% | 0.84(0.7-0.98) | 78.51 | 53.01 | -32.48% | -2.56(-3.03 - -2.08) | 3625.94 | 2913.15 | -19.66% | -0.64(-0.75 - -0.52) |
| Croatia | IFBD | 527.39 | 591.19 | 12.10% | 0.93(0.89-0.96) | 16.03 | 33.1 | 106.49% | 1.79(1.42-2.17) | 1866.17 | 2288.04 | 22.61% | 0.79(0.65-0.93) |
| Croatia | PACA | 2860.95 | 3012.19 | 5.29% | -0.26(-0.34 - -0.17) | 157.83 | 143.5 | -9.08% | -1.64(-1.9 - -1.38) | 4864.36 | 3229.9 | -33.60% | -2.32(-2.55 - -2.09) |
| Croatia | PIIO | 7791.31 | 8860.73 | 13.73% | -0.1(-0.19 - -0.02) | 123.07 | 166.58 | 35.36% | -0.42(-0.56 - -0.27) | 2683.39 | 2678.27 | -0.19% | -1.15(-1.28 - -1.03) |
| Croatia | UDSD | 243922.02 | 243034.07 | -0.36% | -0.036(-0.051 - -0.021) | 278.28 | 234.3 | -15.80% | -2.08(-2.28 - -1.89) | 11722.36 | 9293.48 | -20.72% | -1.29(-1.38 - -1.2) |
| Croatia | VAID | 1160.17 | 1351.92 | 16.53% | -0.49(-0.77 - -0.21) | 181.72 | 227.38 | 25.13% | -1.14(-1.44 - -0.83) | 3388.66 | 3360.64 | -0.83% | -1.72(-2.02 - -1.42) |
| Cuba | Digestive diseases | 835891.28 | 1075551.48 | 28.67% | 0.08(0.07-0.09) | 2682.55 | 4364.57 | 62.70% | -0.62(-0.88 - -0.37) | 95421.68 | 137119.05 | 43.70% | -0.38(-0.56 - -0.21) |
| Cuba | APED | 28507.41 | 32134.74 | 12.72% | 0.84(0.79-0.89) | 92.09 | 97.96 | 6.37% | -1.04(-1.63 - -0.45) | 3461.84 | 2782.96 | -19.61% | -1.22(-1.74 - -0.71) |
| Cuba | COCLD | 1508.2 | 2228.32 | 47.75% | 0.57(0.5-0.64) | 1127.13 | 2035.78 | 80.62% | -0.1(-0.44-0.23) | 30839.51 | 56053.78 | 81.76% | 0.18(-0.12-0.48) |
| Cuba | GABD | 38963.49 | 50446.64 | 29.47% | -0.1(-0.17 - -0.04) | 231.04 | 237.9 | 2.97% | -2.07(-2.54 - -1.59) | 9101.14 | 8853.5 | -2.72% | -1.5(-1.78 - -1.21) |
| Cuba | IFAH | 26298.02 | 39363.38 | 49.68% | 0.72(0.6-0.84) | 99.01 | 168.4 | 70.08% | -0.64(-0.84 - -0.43) | 7572.87 | 10061.26 | 32.86% | -0.17(-0.25 - -0.1) |
| Cuba | IFBD | 235.25 | 358.72 | 52.48% | 0.35(0.19-0.52) | 36.22 | 42.13 | 16.32% | -2.04(-2.47 - -1.6) | 1518.34 | 1681.68 | 10.76% | -1.38(-1.64 - -1.12) |
| Cuba | PACA | 3079.24 | 4303.22 | 39.75% | 0.13(0.11-0.16) | 98.02 | 210.35 | 114.60% | 1.14(0.96-1.32) | 3375 | 5914.46 | 75.24% | 0.81(0.66-0.95) |
| Cuba | PIIO | 13068.73 | 22245.4 | 70.22% | 0.8(0.74-0.86) | 275.58 | 471.5 | 71.09% | -0.5(-0.61 - -0.39) | 6771.53 | 8954.98 | 32.24% | -0.75(-0.87 - -0.63) |
| Cuba | UDSD | 722689.32 | 921528.41 | 27.51% | 0.012(0.008-0.016) | 478.1 | 537.93 | 12.51% | -2.64(-2.96 - -2.31) | 27319.66 | 32104.19 | 17.51% | -0.99(-1.12 - -0.86) |
| Cuba | VAID | 1541.62 | 2942.65 | 90.88% | 0.6(0.49-0.71) | 214.72 | 390.92 | 82.06% | -0.29(-0.48 - -0.11) | 4270 | 6961.68 | 63.04% | -0.37(-0.55 - -0.2) |
| Cyprus | Digestive diseases | 33878.49 | 69692.24 | 105.71% | 0.14(0.08-0.2) | 231.55 | 364.2 | 57.29% | -2.32(-2.45 - -2.18) | 5776.49 | 8439.98 | 46.11% | -1.89(-1.97 - -1.81) |
| Cyprus | APED | 1483.16 | 2314.23 | 56.03% | 0.22(0.06-0.38) | 1.49 | 1.22 | -18.12% | -4.72(-5.03 - -4.41) | 47.8 | 50.95 | 6.59% | -1.89(-2.03 - -1.74) |
| Cyprus | COCLD | 161.4 | 287.74 | 78.28% | -0.62(-0.71 - -0.53) | 102.32 | 140.37 | 37.19% | -2.53(-2.68 - -2.39) | 2449.14 | 3106.43 | 26.84% | -2.45(-2.59 - -2.32) |
| Cyprus | GABD | 2642.68 | 5946.63 | 125.02% | 0.71(0.13-1.3) | 23 | 44.52 | 93.57% | -1.97(-2.13 - -1.8) | 565.78 | 939.46 | 66.05% | -1.5(-1.71 - -1.3) |
| Cyprus | IFAH | 762.91 | 1899.33 | 148.96% | 1.7(1.11-2.29) | 4.13 | 7.95 | 92.49% | -1.99(-2.23 - -1.74) | 235.07 | 423.96 | 80.35% | -0.05(-0.36-0.25) |
| Cyprus | IFBD | 32.33 | 91.5 | 183.02% | 1.22(0.91-1.53) | 11.64 | 15.8 | 35.74% | -3.13(-3.45 - -2.81) | 257.41 | 370.45 | 43.91% | -2.26(-2.48 - -2.03) |
| Cyprus | PACA | 175.05 | 378.64 | 116.30% | -0.24(-0.33 - -0.15) | 11.73 | 20.06 | 71.01% | -1.9(-2.02 - -1.78) | 252.78 | 381.21 | 50.81% | -1.91(-2.01 - -1.82) |
| Cyprus | PIIO | 707.08 | 2240.16 | 216.82% | 1.6(1.44-1.76) | 24.06 | 56.51 | 134.82% | -0.99(-1.14 - -0.85) | 453.91 | 794.82 | 75.10% | -1.45(-1.59 - -1.32) |
| Cyprus | UDSD | 27849.55 | 56328.8 | 102.26% | -0.025(-0.049 - -0.002) | 28.78 | 24.06 | -16.40% | -5.14(-5.37 - -4.91) | 1076.86 | 1546.34 | 43.60% | -1.96(-2.13 - -1.79) |
| Cyprus | VAID | 64.33 | 205.21 | 219.00% | 0.5(0.38-0.61) | 11.81 | 23.84 | 101.86% | -1.54(-1.66 - -1.41) | 197.57 | 349.47 | 76.88% | -1.67(-1.78 - -1.56) |
| Czechia | Digestive diseases | 715174.98 | 872182.28 | 21.95% | 0.12(0.1-0.14) | 4889.86 | 4845.71 | -0.90% | -1.08(-1.23 - -0.92) | 159031.84 | 147765.63 | -7.08% | -0.95(-1.06 - -0.83) |
| Czechia | APED | 21383.25 | 22933.89 | 7.25% | 0.98(0.71-1.25) | 62.6 | 28.66 | -54.22% | -3.92(-4.23 - -3.61) | 1756.77 | 816.17 | -53.54% | -2.6(-2.84 - -2.35) |
| Czechia | COCLD | 2715.16 | 2670.56 | -1.64% | -0.19(-0.27 - -0.12) | 2287.41 | 2319.71 | 1.41% | -0.68(-0.86 - -0.49) | 71316.09 | 66474.14 | -6.79% | -0.84(-1.02 - -0.65) |
| Czechia | GABD | 149863.21 | 185740.9 | 23.94% | 0.04(-0.01-0.08) | 494.61 | 416.44 | -15.80% | -1.52(-2.29 - -0.75) | 21963.54 | 19431.97 | -11.53% | -1.06(-1.34 - -0.78) |
| Czechia | IFAH | 18064.93 | 23285.48 | 28.90% | 1.13(0.9-1.37) | 182.89 | 73.54 | -59.79% | -4.2(-5.12 - -3.28) | 6748.21 | 4915.14 | -27.16% | -0.64(-1.05 - -0.23) |
| Czechia | IFBD | 542.96 | 1041.22 | 91.77% | 1.84(1.64-2.04) | 71.25 | 113.6 | 59.44% | 0.8(0.45-1.15) | 3246.02 | 5258.98 | 62.01% | 0.98(0.9-1.06) |
| Czechia | PACA | 6353.21 | 7695.39 | 21.13% | -0.27(-0.35 - -0.2) | 421.42 | 393.9 | -6.53% | -1.63(-1.78 - -1.48) | 12654.57 | 10047.17 | -20.60% | -1.94(-2.06 - -1.81) |
| Czechia | PIIO | 15293.49 | 21833.84 | 42.77% | -0.05(-0.17-0.07) | 245.82 | 363.55 | 47.89% | -0.28(-0.41 - -0.14) | 5494.3 | 6242.83 | 13.62% | -0.97(-1.09 - -0.85) |
| Czechia | UDSD | 498990.67 | 604057.05 | 21.06% | 0.059(0.047-0.07) | 701.19 | 524.97 | -25.13% | -2.63(-2.94 - -2.31) | 26922.64 | 23560.63 | -12.49% | -1.2(-1.29 - -1.1) |
| Czechia | VAID | 1968.1 | 2923.95 | 48.57% | -0.31(-0.57 - -0.06) | 266.01 | 277.39 | 4.28% | -1.46(-1.63 - -1.29) | 4838.43 | 4413.58 | -8.78% | -1.71(-1.84 - -1.57) |
| Democratic People's Republic of Korea | Digestive diseases | 645896.65 | 951871.19 | 47.37% | -0.21(-0.24 - -0.18) | 6398.62 | 9204.68 | 43.85% | -1.01(-1.11 - -0.92) | 246410.82 | 289277.68 | 17.40% | -1.09(-1.13 - -1.05) |
| Democratic People's Republic of Korea | APED | 35025.04 | 43908.81 | 25.36% | -0.39(-0.52 - -0.26) | 48.02 | 65.54 | 36.48% | -1.04(-1.31 - -0.78) | 2340.25 | 2326.54 | -0.59% | -1.27(-1.41 - -1.13) |
| Democratic People's Republic of Korea | COCLD | 5236.56 | 6457.55 | 23.32% | -0.83(-0.98 - -0.68) | 3621.92 | 5170.78 | 42.76% | -0.96(-1.01 - -0.91) | 122841.55 | 155742.34 | 26.78% | -1.06(-1.11 - -1) |
| Democratic People's Republic of Korea | GABD | 63121.13 | 97382.64 | 54.28% | -0.31(-0.36 - -0.26) | 285.09 | 414.56 | 45.41% | -1.49(-1.6 - -1.38) | 14686.07 | 19113.8 | 30.15% | -1.17(-1.21 - -1.12) |
| Democratic People's Republic of Korea | IFAH | 12570 | 20567.24 | 63.62% | 1.46(1.32-1.6) | 76.97 | 59.76 | -22.36% | -1.75(-1.83 - -1.68) | 7477.15 | 6023.19 | -19.45% | -0.48(-0.63 - -0.34) |
| Democratic People's Republic of Korea | IFBD | 196.52 | 387.24 | 97.05% | 1.23(1.07-1.39) | 73.86 | 107.66 | 45.76% | -1.1(-1.2 - -1) | 3362.72 | 3161.37 | -5.99% | -1.31(-1.41 - -1.21) |
| Democratic People's Republic of Korea | PACA | 7222.75 | 12273.67 | 69.93% | 0(-0.01-0) | 171.51 | 283.21 | 65.13% | -0.46(-0.54 - -0.38) | 6117.68 | 8546.41 | 39.70% | -0.56(-0.61 - -0.52) |
| Democratic People's Republic of Korea | PIIO | 31158.91 | 19958.25 | -35.95% | -3.19(-3.54 - -2.85) | 408.05 | 475.52 | 16.53% | -0.6(-0.74 - -0.47) | 25061.06 | 12513.5 | -50.07% | -1.74(-1.85 - -1.62) |
| Democratic People's Republic of Korea | UDSD | 490208.94 | 748639.45 | 52.72% | -0.104(-0.111 - -0.098) | 1477.57 | 2257.08 | 52.76% | -1.16(-1.42 - -0.89) | 55595.26 | 72742.1 | 30.84% | -1.06(-1.23 - -0.88) |
| Democratic People's Republic of Korea | VAID | 1156.8 | 2296.33 | 98.51% | 0.43(0.21-0.65) | 54 | 123.95 | 129.54% | -0.08(-0.16-0.01) | 1252.9 | 2442.63 | 94.96% | -0.08(-0.16 - -0.01) |
| Democratic Republic of the Congo | Digestive diseases | 1428709.85 | 3647335.78 | 155.29% | 0.19(0.18-0.2) | 14646.03 | 24530.63 | 67.49% | -1.08(-1.15 - -1.01) | 640229.6 | 1054484.1 | 64.70% | -0.93(-0.99 - -0.87) |
| Democratic Republic of the Congo | APED | 89392.22 | 285812.94 | 219.73% | 1.02(0.94-1.11) | 369.42 | 489.62 | 32.54% | -1.26(-1.45 - -1.06) | 23118.72 | 28963.68 | 25.28% | -1.28(-1.46 - -1.09) |
| Democratic Republic of the Congo | COCLD | 7896.19 | 21836.69 | 176.55% | 0.56(0.49-0.64) | 8622.54 | 14392.67 | 66.92% | -1.25(-1.32 - -1.19) | 316335.57 | 537020.19 | 69.76% | -1.12(-1.18 - -1.05) |
| Democratic Republic of the Congo | GABD | 11313.29 | 30718.72 | 171.53% | 0.67(0.63-0.7) | 525.1 | 1086.46 | 106.91% | -0.42(-0.47 - -0.35) | 18249.66 | 34627.8 | 89.74% | -0.46(-0.52 - -0.4) |
| Democratic Republic of the Congo | IFAH | 40628.2 | 131871.18 | 224.58% | 1.47(1.42-1.53) | 499.14 | 614.12 | 23.04% | -0.92(-1.01 - -0.84) | 43149.8 | 62147.91 | 44.03% | -0.32(-0.4 - -0.24) |
| Democratic Republic of the Congo | IFBD | 256.25 | 745.09 | 190.77% | 0.6(0.52-0.68) | 130.2 | 230.74 | 77.22% | -0.78(-0.91 - -0.64) | 6455.84 | 10017.94 | 55.18% | -0.82(-0.97 - -0.66) |
| Democratic Republic of the Congo | PACA | 5128.57 | 12234.98 | 138.57% | -0.02(-0.03 - -0.01) | 298.1 | 592.7 | 98.83% | -0.78(-0.92 - -0.65) | 11451.29 | 23453.68 | 104.81% | -0.59(-0.73 - -0.45) |
| Democratic Republic of the Congo | PIIO | 36386.62 | 88777.04 | 143.98% | 0.31(0.23-0.39) | 1390.65 | 2490.03 | 79.05% | -0.67(-0.77 - -0.56) | 79015.45 | 120196.81 | 52.12% | -0.5(-0.63 - -0.37) |
| Democratic Republic of the Congo | UDSD | 1235429.02 | 3069863.66 | 148.49% | 0.1(0.096-0.104) | 1637.01 | 2631.5 | 60.75% | -0.89(-1.06 - -0.72) | 96274 | 170756.38 | 77.36% | -0.68(-0.78 - -0.57) |
| Democratic Republic of the Congo | VAID | 2279.51 | 5475.49 | 140.20% | 0.1(-0.08-0.29) | 342.76 | 583.27 | 70.17% | -1.3(-1.53 - -1.08) | 10195.27 | 15366.82 | 50.72% | -1.31(-1.54 - -1.08) |
| Denmark | Digestive diseases | 325059.31 | 380965 | 17.20% | -0.07(-0.1 - -0.05) | 2155.43 | 2683.93 | 24.52% | -1.27(-1.76 - -0.79) | 61747.01 | 63681.62 | 3.13% | -1.49(-1.86 - -1.13) |
| Denmark | APED | 12911.05 | 14097.25 | 9.19% | 0.28(0.14-0.42) | 26.29 | 27.03 | 2.81% | -1.97(-2.47 - -1.47) | 685.18 | 594.09 | -13.29% | -1.73(-2.07 - -1.39) |
| Denmark | COCLD | 1015.31 | 1242.01 | 22.33% | 0.62(0.21-1.02) | 770.1 | 954.67 | 23.97% | -1.17(-1.65 - -0.69) | 25189.27 | 25022.17 | -0.66% | -1.84(-2.31 - -1.37) |
| Denmark | GABD | 45039.9 | 45576.53 | 1.19% | -0.92(-1.04 - -0.81) | 121.51 | 196.7 | 61.88% | -0.14(-0.48-0.21) | 5451.87 | 5521.67 | 1.28% | -1.3(-1.46 - -1.14) |
| Denmark | IFAH | 5056.97 | 6396.76 | 26.49% | 0(-0.34-0.33) | 54.83 | 69.28 | 26.35% | -1.09(-1.38 - -0.79) | 1786.75 | 1915.33 | 7.20% | -1.02(-1.26 - -0.77) |
| Denmark | IFBD | 1373.33 | 1333.57 | -2.90% | -0.01(-0.12-0.11) | 42.63 | 70.49 | 65.35% | 0.01(-0.61-0.63) | 2892.96 | 3390.74 | 17.21% | 0.14(-0.02-0.29) |
| Denmark | PACA | 1413.16 | 1738.32 | 23.01% | -0.04(-0.08 - -0.01) | 117.42 | 157.98 | 34.54% | -0.67(-1.04 - -0.31) | 3223.6 | 3577.55 | 10.98% | -1.18(-1.53 - -0.83) |
| Denmark | PIIO | 7533.09 | 11750.24 | 55.98% | 0.69(0.6-0.77) | 186.34 | 313.71 | 68.35% | 0.03(-0.39-0.45) | 3041.71 | 4294.32 | 41.18% | -0.53(-0.9 - -0.15) |
| Denmark | UDSD | 249150.58 | 296937.94 | 19.18% | 0.009(0.002-0.017) | 489.38 | 435.12 | -11.09% | -2.88(-3.52 - -2.24) | 13504.6 | 12623.61 | -6.52% | -1.56(-1.86 - -1.27) |
| Denmark | VAID | 1565.91 | 1892.37 | 20.85% | -0.62(-0.9 - -0.33) | 165.59 | 162.14 | -2.08% | -2.46(-3.01 - -1.91) | 2863.15 | 2503.13 | -12.57% | -2.82(-3.35 - -2.29) |
| Djibouti | Digestive diseases | 16976.34 | 55914.64 | 229.37% | 0.09(0.07-0.11) | 115.51 | 357.25 | 209.28% | -0.73(-0.81 - -0.64) | 5279.96 | 14262.38 | 170.12% | -0.81(-0.92 - -0.7) |
| Djibouti | APED | 736.35 | 2244.69 | 204.84% | 1.12(0.93-1.32) | 2.6 | 4.65 | 78.85% | -1.73(-1.99 - -1.47) | 166.09 | 255.53 | 53.85% | -1.81(-2.11 - -1.5) |
| Djibouti | COCLD | 92.16 | 263.58 | 186.00% | -0.27(-0.35 - -0.18) | 68.1 | 226.01 | 231.88% | -0.66(-0.73 - -0.59) | 2560.88 | 7748.84 | 202.59% | -0.76(-0.86 - -0.66) |
| Djibouti | GABD | 128.76 | 447.77 | 247.76% | 0.63(0.57-0.69) | 3.91 | 15.31 | 291.56% | -0.05(-0.11-0) | 149.7 | 472.21 | 215.44% | -0.35(-0.43 - -0.27) |
| Djibouti | IFAH | 357.39 | 1222.01 | 241.93% | 0.84(0.71-0.97) | 2.64 | 6.63 | 151.14% | -0.53(-0.61 - -0.46) | 252.39 | 537.51 | 112.97% | -0.49(-0.55 - -0.42) |
| Djibouti | IFBD | 3.01 | 10.42 | 246.18% | -0.02(-0.16-0.11) | 1.13 | 3.28 | 190.27% | -0.56(-0.67 - -0.45) | 60.38 | 144.28 | 138.95% | -0.67(-0.82 - -0.53) |
| Djibouti | PACA | 59.03 | 185.45 | 214.16% | -0.16(-0.18 - -0.14) | 2.41 | 7.63 | 216.60% | -0.83(-0.93 - -0.73) | 98.76 | 288.33 | 191.95% | -0.9(-1.03 - -0.77) |
| Djibouti | PIIO | 381.63 | 1510.85 | 295.89% | 1.31(1.13-1.49) | 14.35 | 40.43 | 181.74% | -0.32(-0.39 - -0.25) | 796.95 | 1777.41 | 123.03% | -0.51(-0.61 - -0.4) |
| Djibouti | UDSD | 15196.35 | 49954.46 | 228.73% | 0.003(-0.005-0.012) | 14.33 | 33.6 | 134.47% | -1.71(-1.94 - -1.48) | 949.77 | 2407.89 | 153.52% | -1.21(-1.36 - -1.05) |
| Djibouti | VAID | 21.67 | 75.4 | 247.95% | 0.75(0.61-0.9) | 1.42 | 6.22 | 338.03% | 0.08(0.04-0.13) | 42.99 | 159.73 | 271.55% | -0.06(-0.12 - -0.01) |
| Dominica | Digestive diseases | 4888.41 | 5634.96 | 15.27% | 0.02(0.02-0.03) | 26.12 | 25.12 | -3.86% | -0.92(-1.01 - -0.83) | 820.56 | 796.36 | -2.95% | -0.8(-0.87 - -0.73) |
| Dominica | APED | 171.3 | 181.35 | 5.87% | 0.72(0.67-0.67) | 0.55 | 0.39 | -29.09% | -1.12(-1.63 - -0.6) | 21.75 | 14.66 | -32.60% | -0.88(-1.31 - -0.46) |
| Dominica | COCLD | 10.16 | 10.9 | 7.28% | -0.74(-0.84 - -0.64) | 11.83 | 10.88 | -8.03% | -1.29(-1.41 - -1.18) | 313.07 | 291.48 | -6.90% | -1.37(-1.48 - -1.26) |
| Dominica | GABD | 200.58 | 214.08 | 6.73% | -0.23(-0.28 - -0.18) | 1.15 | 0.79 | -31.30% | -1.76(-2.08 - -1.44) | 48.35 | 40.51 | -16.22% | -1.14(-1.33 - -0.94) |
| Dominica | IFAH | 135.03 | 154.03 | 14.07% | 0.55(0.51-0.59) | 0.59 | 0.58 | -1.69% | -0.41(-0.61 - -0.21) | 47.83 | 49.36 | 3.20% | 0.16(0.09-0.23) |
| Dominica | IFBD | 1.34 | 2.33 | 73.88% | 1.13(0.92-1.33) | 0.71 | 0.65 | -8.45% | -1.2(-1.28 - -1.12) | 23.12 | 20.54 | -11.16% | -1.17(-1.27 - -1.07) |
| Dominica | PACA | 20.15 | 22.76 | 12.95% | -0.08(-0.1 - -0.05) | 1.24 | 1.28 | 3.23% | -0.63(-0.68 - -0.58) | 36.19 | 36.98 | 2.18% | -0.71(-0.76 - -0.66) |
| Dominica | PIIO | 70.82 | 69.03 | -2.52% | -0.14(-0.25 - -0.02) | 3.69 | 3.74 | 1.50% | -0.35(-0.52 - -0.19) | 93.26 | 83.62 | -10.34% | -0.26(-0.41 - -0.11) |
| Dominica | UDSD | 4270.92 | 4970.73 | 16.39% | -0.004(-0.007 - -0.002) | 4.11 | 2.9 | -29.44% | -2.2(-2.39 - -2) | 189.29 | 180.75 | -4.51% | -0.95(-1.04 - -0.86) |
| Dominica | VAID | 8.11 | 9.75 | 20.22% | 0.12(0.05-0.2) | 1.44 | 1.49 | 3.47% | -0.45(-0.52 - -0.37) | 26.17 | 26.87 | 2.67% | -0.54(-0.61 - -0.47) |
| Dominican Republic | Digestive diseases | 421652.22 | 800364.42 | 89.82% | 0.1(0.08-0.11) | 2354.91 | 3915.31 | 66.26% | -0.97(-1.38 - -0.56) | 114589.27 | 141907.07 | 23.84% | -1.24(-1.66 - -0.82) |
| Dominican Republic | APED | 18739.04 | 34350.66 | 83.31% | 0.63(0.45-0.82) | 119.84 | 82.3 | -31.33% | -2.02(-3.03 - -1) | 8753.79 | 4619.61 | -47.23% | -2.62(-3.67 - -1.55) |
| Dominican Republic | COCLD | 1417.61 | 3263.11 | 130.18% | 0.39(0.26-0.52) | 1530 | 2945.24 | 92.50% | -0.94(-1.39 - -0.49) | 53226.49 | 83024.44 | 55.98% | -1.25(-1.75 - -0.75) |
| Dominican Republic | GABD | 14747.25 | 29445.62 | 99.67% | 0.33(0.29-0.38) | 43.93 | 89.82 | 104.46% | -0.24(-0.81-0.33) | 3408.9 | 5443.76 | 59.69% | -0.6(-0.93 - -0.28) |
| Dominican Republic | IFAH | 13196.27 | 24867.36 | 88.44% | 0.71(0.65-0.77) | 95.51 | 40.89 | -57.19% | -3.25(-3.98 - -2.52) | 11037.98 | 7296.81 | -33.89% | -1.85(-2.31 - -1.38) |
| Dominican Republic | IFBD | 116.69 | 288.73 | 147.43% | 0.77(0.64-0.89) | 11.25 | 22.44 | 99.47% | 0.09(-0.13-0.32) | 732.25 | 1108.89 | 51.44% | -0.27(-0.5 - -0.04) |
| Dominican Republic | PACA | 1444.81 | 2760.87 | 91.09% | 0.01(-0.01-0.04) | 38.62 | 79.88 | 106.84% | 0.06(-0.22-0.33) | 1588.04 | 2678.59 | 68.67% | -0.29(-0.59-0.01) |
| Dominican Republic | PIIO | 4268.42 | 9675.96 | 126.69% | 0.83(0.69-0.98) | 189.39 | 144.81 | -23.54% | -1.46(-1.66 - -1.25) | 14512.91 | 7149.21 | -50.74% | -2.44(-2.98 - -1.9) |
| Dominican Republic | UDSD | 367361.13 | 694753.03 | 89.12% | 0.033(0.03-0.036) | 238.21 | 343.53 | 44.21% | -1.31(-1.55 - -1.07) | 16591.58 | 25376.99 | 52.95% | -0.72(-0.86 - -0.59) |
| Dominican Republic | VAID | 361.01 | 959.08 | 165.67% | 1.32(1.27-1.36) | 24.86 | 71.39 | 187.17% | 0.91(0.55-1.27) | 662.29 | 1494.62 | 125.67% | 0.53(0.15-0.91) |
| Ecuador | Digestive diseases | 645611.01 | 1383781.79 | 114.34% | 0.07(0.05-0.08) | 3487.86 | 6908.31 | 98.07% | -0.4(-0.64 - -0.16) | 154003.87 | 217093.94 | 40.97% | -1.13(-1.32 - -0.94) |
| Ecuador | APED | 77754.23 | 156015.22 | 100.65% | 0.55(0.39-0.71) | 227.51 | 130.75 | -42.53% | -3.13(-3.88 - -2.36) | 14480.64 | 6690.23 | -53.80% | -3.61(-4.34 - -2.88) |
| Ecuador | COCLD | 1689.27 | 6405.89 | 279.21% | 1.87(1.6-2.13) | 1472.87 | 3904.69 | 165.11% | 0.35(0.12-0.58) | 50167.61 | 100815.45 | 100.96% | -0.43(-0.65 - -0.21) |
| Ecuador | GABD | 16822.8 | 44457.63 | 164.27% | 0.77(0.73-0.82) | 226.52 | 560.14 | 147.28% | 0.81(0.1-1.51) | 8256.32 | 15516.96 | 87.94% | -0.14(-0.66-0.38) |
| Ecuador | IFAH | 20357.73 | 48632.66 | 138.89% | 1.01(0.93-1.09) | 95.68 | 127.17 | 32.91% | -0.77(-1.24 - -0.3) | 11766.6 | 14414.59 | 22.50% | -0.82(-1.09 - -0.55) |
| Ecuador | IFBD | 119.9 | 323.26 | 169.61% | -0.07(-0.42-0.28) | 16.16 | 33.6 | 107.92% | 0.55(-0.03-1.13) | 982.02 | 1475.5 | 50.25% | -0.33(-0.74-0.08) |
| Ecuador | PACA | 3129.66 | 7753.54 | 147.74% | -0.02(-0.15-0.12) | 339.82 | 449.16 | 32.18% | -2.69(-2.91 - -2.48) | 12200.58 | 13308.72 | 9.08% | -3.08(-3.32 - -2.84) |
| Ecuador | PIIO | 10169.61 | 21210.51 | 108.57% | 0.08(-0.01-0.16) | 430.13 | 585.08 | 36.02% | -1.06(-1.22 - -0.89) | 23588.72 | 15861.15 | -32.76% | -2.6(-2.81 - -2.39) |
| Ecuador | UDSD | 515148.68 | 1097789.53 | 113.10% | -0.06(-0.075 - -0.045) | 523.96 | 545.52 | 4.11% | -2.87(-3.31 - -2.43) | 25430.75 | 35534.83 | 39.73% | -1.72(-1.93 - -1.51) |
| Ecuador | VAID | 419.14 | 1193.57 | 184.77% | 0.56(0.33-0.79) | 68.83 | 253.06 | 267.66% | 1.7(1.27-2.14) | 1652.72 | 4438.5 | 168.56% | 1(0.59-1.41) |
| Egypt | Digestive diseases | 2511997.56 | 5248731.38 | 108.95% | 0.1(0.09-0.11) | 44121.15 | 69215.68 | 56.88% | -0.78(-0.91 - -0.65) | 1245164.08 | 1873908.63 | 50.49% | -0.86(-0.97 - -0.74) |
| Egypt | APED | 108283.3 | 281599.5 | 160.06% | 1.27(1.19-1.36) | 150.5 | 149.67 | -0.55% | -1.99(-2.23 - -1.75) | 7142.02 | 8464.8 | 18.52% | -1.43(-1.68 - -1.18) |
| Egypt | COCLD | 24559.52 | 65387.67 | 166.24% | 0.74(0.68-0.8) | 39203.26 | 62635.37 | 59.77% | -0.77(-0.9 - -0.64) | 983919.08 | 1519838.08 | 54.47% | -0.85(-0.97 - -0.73) |
| Egypt | GABD | 110624.8 | 252063.91 | 127.85% | 0.29(0.25-0.33) | 1137.72 | 1907.92 | 67.70% | -0.47(-0.6 - -0.33) | 46154.9 | 73664.97 | 59.60% | -0.73(-0.85 - -0.61) |
| Egypt | IFAH | 25760.66 | 61950.72 | 140.49% | 1.29(1.26-1.32) | 72.53 | 96.81 | 33.48% | -0.75(-0.98 - -0.51) | 9592.5 | 15915.6 | 65.92% | -0.03(-0.13-0.07) |
| Egypt | IFBD | 1624.29 | 3832.86 | 135.97% | 0.32(0.17-0.47) | 59.23 | 118.96 | 100.84% | 0.32(0.07-0.56) | 4520.52 | 9452.6 | 109.10% | 0.19(0.1-0.27) |
| Egypt | PACA | 10581.59 | 21875 | 106.73% | 0.09(0.04-0.13) | 298.66 | 571.46 | 91.34% | 0.05(-0.1-0.2) | 8576.48 | 16098.67 | 87.71% | -0.04(-0.18-0.11) |
| Egypt | PIIO | 31699.62 | 78841.71 | 148.71% | 1(0.93-1.08) | 1239.62 | 1302.94 | 5.11% | -1.22(-1.38 - -1.05) | 69016.22 | 55274.01 | -19.91% | -1.72(-1.88 - -1.56) |
| Egypt | UDSD | 2196203.8 | 4474849.96 | 103.75% | -0.003(-0.006-0) | 1472.8 | 1480.65 | 0.53% | -1.93(-2.14 - -1.72) | 98706.76 | 143819.16 | 45.70% | -1(-1.12 - -0.87) |
| Egypt | VAID | 2659.97 | 8330.04 | 213.16% | 1.64(1.54-1.74) | 140.13 | 299.36 | 113.63% | 0.27(0.15-0.4) | 2985.63 | 6650.72 | 122.76% | 0.33(0.21-0.46) |
| El Salvador | Digestive diseases | 340534.58 | 495173.19 | 45.41% | -0.09(-0.13 - -0.05) | 2123.67 | 2431.96 | 14.52% | -1.83(-2.13 - -1.52) | 95144.54 | 81699.18 | -14.13% | -1.96(-2.28 - -1.64) |
| El Salvador | APED | 31782.6 | 25045.83 | -21.20% | -1.72(-2.24 - -1.2) | 145.14 | 30.21 | -79.19% | -5.65(-6.84 - -4.44) | 9840.4 | 1307.93 | -86.71% | -6.48(-7.69 - -5.26) |
| El Salvador | COCLD | 1372.76 | 2188.46 | 59.42% | 0.22(0.12-0.32) | 970.6 | 1500.02 | 54.55% | -0.78(-0.98 - -0.57) | 35642.59 | 43530.42 | 22.13% | -1.15(-1.41 - -0.9) |
| El Salvador | GABD | 33734.94 | 56714.75 | 68.12% | 0.3(0.25-0.35) | 116.11 | 132.15 | 13.81% | -1.84(-2.56 - -1.11) | 7611.51 | 8237 | 8.22% | -1.47(-1.83 - -1.1) |
| El Salvador | IFAH | 10874.98 | 13584.95 | 24.92% | -0.39(-0.52 - -0.25) | 68.26 | 65.71 | -3.74% | -2.16(-2.52 - -1.81) | 6001.31 | 4099.03 | -31.70% | -2.09(-2.39 - -1.78) |
| El Salvador | IFBD | 100.77 | 148.13 | 47.00% | -0.22(-0.36 - -0.09) | 12.66 | 9.03 | -28.67% | -3.87(-4.22 - -3.52) | 660.53 | 437.06 | -33.83% | -2.87(-3.18 - -2.56) |
| El Salvador | PACA | 1598.1 | 2262.68 | 41.59% | -0.3(-0.31 - -0.28) | 84.87 | 93.37 | 10.02% | -2.21(-2.46 - -1.95) | 3117.99 | 2733.22 | -12.34% | -2.45(-2.73 - -2.16) |
| El Salvador | PIIO | 5450.89 | 7862.47 | 44.24% | 0.61(0.54-0.68) | 145.56 | 112.35 | -22.82% | -2.25(-2.59 - -1.9) | 8764.8 | 3158.73 | -63.96% | -3.6(-4.11 - -3.09) |
| El Salvador | UDSD | 255321.59 | 386656.82 | 51.44% | -0.032(-0.037 - -0.028) | 450.76 | 298.67 | -33.74% | -4.9(-5.43 - -4.37) | 16845.41 | 13819.93 | -17.96% | -2.99(-3.34 - -2.63) |
| El Salvador | VAID | 297.94 | 709.11 | 138.00% | 1.57(1.5-1.63) | 23.33 | 64.38 | 175.95% | 0.94(0.76-1.12) | 499.54 | 1081.6 | 116.52% | 0.62(0.43-0.82) |
| Equa-rial Guinea | Digestive diseases | 15930.66 | 58267.53 | 265.76% | 0.18(0.15-0.22) | 183.04 | 233.93 | 27.80% | -2.46(-2.66 - -2.25) | 7479.91 | 10078.18 | 34.74% | -2.77(-3 - -2.55) |
| Equa-rial Guinea | APED | 831.6 | 4631.06 | 456.89% | 1.39(1.09-1.69) | 5.68 | 2.8 | -50.70% | -6.13(-6.56 - -5.7) | 306.99 | 188.3 | -38.66% | -6.23(-6.65 - -5.81) |
| Equa-rial Guinea | COCLD | 92.24 | 282.06 | 205.79% | -0.19(-0.3 - -0.07) | 91.71 | 126.16 | 37.56% | -2.37(-2.56 - -2.18) | 3267.74 | 4598.54 | 40.73% | -2.74(-2.95 - -2.52) |
| Equa-rial Guinea | GABD | 111.75 | 521.3 | 366.49% | 1.41(1.23-1.58) | 8.05 | 14.51 | 80.25% | -0.88(-0.98 - -0.78) | 261.57 | 413.48 | 58.08% | -1.72(-1.84 - -1.61) |
| Equa-rial Guinea | IFAH | 435.87 | 1689.4 | 287.59% | 1.9(1.41-2.4) | 5.52 | 4.69 | -15.04% | -3.09(-3.39 - -2.79) | 430.86 | 625.34 | 45.14% | -2.11(-2.28 - -1.93) |
| Equa-rial Guinea | IFBD | 2.83 | 15.44 | 445.58% | 1.92(1.78-2.07) | 1.68 | 2.8 | 66.67% | -1.45(-1.6 - -1.3) | 72.61 | 118.56 | 63.28% | -1.89(-2.04 - -1.73) |
| Equa-rial Guinea | PACA | 61.24 | 197.98 | 223.29% | -0.11(-0.12 - -0.1) | 4.78 | 7.34 | 53.56% | -2.21(-2.46 - -1.96) | 175.29 | 288.97 | 64.85% | -2.53(-2.82 - -2.24) |
| Equa-rial Guinea | PIIO | 320.99 | 1863.98 | 480.69% | 2.96(2.74-3.18) | 18.64 | 27.63 | 48.21% | -2.04(-2.25 - -1.83) | 889.5 | 1229.16 | 38.19% | -2.43(-2.63 - -2.23) |
| Equa-rial Guinea | UDSD | 14053.5 | 48932.65 | 248.19% | -0.003(-0.013-0.007) | 32.22 | 22.51 | -30.14% | -4.43(-4.71 - -4.14) | 1546.34 | 1849.82 | 19.63% | -3.52(-3.81 - -3.23) |
| Equa-rial Guinea | VAID | 20.64 | 133.65 | 547.53% | 3.36(3.05-3.66) | 2.99 | 8.68 | 190.30% | 1.05(0.88-1.22) | 81.34 | 203.49 | 150.17% | 0.37(0.26-0.48) |
| Eritrea | Digestive diseases | 102577.42 | 275891.79 | 168.96% | 0.15(0.14-0.16) | 975.62 | 2491.36 | 155.36% | 0.04(-0.14-0.21) | 42779.12 | 101331.5 | 136.87% | -0.15(-0.3-0.01) |
| Eritrea | APED | 3725.37 | 12810.27 | 243.87% | 1.29(1.18-1.4) | 28.95 | 46.88 | 61.93% | -0.63(-0.73 - -0.54) | 1821.52 | 2596.26 | 42.53% | -0.91(-1.04 - -0.79) |
| Eritrea | COCLD | 539.9 | 1631.33 | 202.15% | 0.67(0.57-0.77) | 610.4 | 1572.29 | 157.58% | -0.12(-0.32-0.08) | 22770.05 | 56606.51 | 148.60% | -0.25(-0.44 - -0.05) |
| Eritrea | GABD | 687.97 | 2302.31 | 234.65% | 1.37(1.31-1.43) | 33.85 | 117.25 | 246.38% | 1.23(1.07-1.4) | 1324.26 | 3744.97 | 182.80% | 0.77(0.64-0.9) |
| Eritrea | IFAH | 1705.53 | 5584.77 | 227.45% | 1.55(1.45-1.64) | 20.73 | 47.12 | 127.30% | 0.64(0.56-0.71) | 1753.64 | 3425.32 | 95.33% | 0.3(0.25-0.35) |
| Eritrea | IFBD | 17.7 | 54.05 | 205.37% | 0.62(0.57-0.67) | 8.55 | 22.69 | 165.38% | 0.71(0.52-0.89) | 474.08 | 1017.42 | 114.61% | 0.34(0.2-0.49) |
| Eritrea | PACA | 371.4 | 949.29 | 155.60% | -0.05(-0.08 - -0.02) | 17.45 | 51.23 | 193.58% | 0.18(0.04-0.33) | 703.48 | 2041.23 | 190.16% | 0.14(0-0.28) |
| Eritrea | PIIO | 1588.07 | 5366.43 | 237.92% | 1.25(1.2-1.31) | 76.89 | 224.61 | 192.14% | 0.68(0.53-0.83) | 4203.72 | 10795.57 | 156.81% | 0.61(0.47-0.75) |
| Eritrea | UDSD | 93839.11 | 246859.35 | 163.07% | 0.058(0.053-0.063) | 133.48 | 274.21 | 105.43% | -0.51(-0.64 - -0.39) | 7849.57 | 16643.44 | 112.03% | -0.54(-0.63 - -0.45) |
| Eritrea | VAID | 102.37 | 333.98 | 226.25% | 1.22(1.14-1.3) | 8.81 | 33.84 | 284.11% | 1.22(0.97-1.46) | 279.37 | 902.06 | 222.89% | 0.93(0.7-1.16) |
| Es-nia | Digestive diseases | 114052.81 | 110501.07 | -3.11% | 0.06(0.05-0.07) | 457.22 | 583.95 | 27.72% | -0.11(-0.66-0.44) | 17204.53 | 18811.52 | 9.34% | -0.11(-0.58-0.37) |
| Es-nia | APED | 4581.89 | 3452.57 | -24.65% | 0.1(0.06-0.15) | 5.79 | 1.54 | -73.40% | -5.85(-6.15 - -5.55) | 226.36 | 71.87 | -68.25% | -3.3(-3.55 - -3.05) |
| Es-nia | COCLD | 209.3 | 277.57 | 32.62% | 1.8(1.38-2.22) | 143.23 | 277.21 | 93.54% | 1.82(0.89-2.75) | 4742.51 | 8892.07 | 87.50% | 1.82(0.84-2.81) |
| Es-nia | GABD | 17202.9 | 16717.05 | -2.82% | -0.15(-0.19 - -0.11) | 37.46 | 22.73 | -39.32% | -3.6(-3.97 - -3.23) | 2430.56 | 1652.9 | -32.00% | -1.77(-1.91 - -1.62) |
| Es-nia | IFAH | 3212.8 | 3559.6 | 10.79% | 1.07(0.87-1.26) | 7.43 | 6.52 | -12.25% | -2.4(-2.79 - -2.01) | 831.23 | 716.84 | -13.76% | -0.32(-0.47 - -0.17) |
| Es-nia | IFBD | 61.95 | 104.94 | 69.39% | 1.82(1.65-1.99) | 10.39 | 8.53 | -17.90% | -2.35(-2.68 - -2.02) | 510.68 | 447.78 | -12.32% | -1.07(-1.32 - -0.81) |
| Es-nia | PACA | 1158.67 | 1177.85 | 1.66% | -0.09(-0.15 - -0.02) | 53.18 | 44.17 | -16.94% | -1.72(-2.13 - -1.3) | 1801.54 | 1295.04 | -28.11% | -1.82(-2.23 - -1.41) |
| Es-nia | PIIO | 2552.44 | 2923.2 | 14.53% | 0.53(0.35-0.71) | 18.51 | 23.7 | 28.04% | -0.73(-0.88 - -0.59) | 593.14 | 458.26 | -22.74% | -1.93(-2.12 - -1.75) |
| Es-nia | UDSD | 84248.53 | 80875.79 | -4.00% | 0.021(0.017-0.026) | 78.98 | 57.66 | -26.99% | -2.75(-3.19 - -2.3) | 4027.8 | 3041.46 | -24.49% | -1.46(-1.67 - -1.24) |
| Es-nia | VAID | 824.32 | 1412.5 | 71.35% | 1.12(0.94-1.3) | 90.92 | 119.82 | 31.79% | -0.9(-1.35 - -0.45) | 1635.03 | 1703.5 | 4.19% | -1.45(-1.91 - -1) |
| Eswatini | Digestive diseases | 29987.95 | 53627.66 | 78.83% | 0.03(0-0.06) | 203.14 | 346.71 | 70.68% | -0.13(-0.56-0.31) | 8589.94 | 14127.24 | 64.46% | -0.11(-0.51-0.28) |
| Eswatini | APED | 2307.47 | 3555.12 | 54.07% | 0.04(-0.19-0.27) | 4.22 | 4.63 | 9.72% | -0.72(-1.25 - -0.19) | 276.01 | 289.35 | 4.83% | -0.71(-1.12 - -0.3) |
| Eswatini | COCLD | 129.86 | 204.18 | 57.23% | -0.27(-0.45 - -0.08) | 115.28 | 189.54 | 64.42% | -0.46(-0.84 - -0.08) | 4184.3 | 6875.57 | 64.32% | -0.36(-0.75-0.04) |
| Eswatini | GABD | 495.09 | 902.1 | 82.21% | 0.34(0.29-0.39) | 8.99 | 19.83 | 120.58% | 0.96(0.47-1.45) | 347.77 | 698.49 | 100.85% | 0.72(0.25-1.19) |
| Eswatini | IFAH | 1189.29 | 1762.68 | 48.21% | 0.26(0.08-0.43) | 3.12 | 5.22 | 67.31% | 0.4(0-0.8) | 512.85 | 699.23 | 36.34% | 0.05(-0.09-0.18) |
| Eswatini | IFBD | 6.44 | 13.48 | 109.32% | 0.48(0.44-0.52) | 2.08 | 3.58 | 72.12% | 0.53(0.14-0.91) | 105.78 | 152.55 | 44.21% | 0.38(0.14-0.63) |
| Eswatini | PACA | 101.01 | 175.22 | 73.47% | -0.05(-0.08 - -0.01) | 4.1 | 9.26 | 125.85% | 0.52(-0.01-1.06) | 152.58 | 360.75 | 136.43% | 0.74(0.18-1.3) |
| Eswatini | PIIO | 776.81 | 1306.68 | 68.21% | 0.1(-0.14-0.33) | 24.53 | 44.16 | 80.05% | 0.21(-0.14-0.56) | 988.16 | 1654.4 | 67.42% | 0.2(-0.09-0.48) |
| Eswatini | UDSD | 24943.04 | 45633.33 | 82.95% | 0.019(0.01-0.029) | 26.51 | 45.28 | 70.80% | 0.23(-0.38-0.84) | 1473.29 | 2518.9 | 70.97% | 0.16(-0.29-0.62) |
| Eswatini | VAID | 38.94 | 74.87 | 92.27% | 0.84(0.61-1.08) | 1.71 | 4.83 | 182.46% | 1.64(1.39-1.89) | 51.04 | 141.33 | 176.90% | 1.68(1.43-1.94) |
| Ethiopia | Digestive diseases | 1776822.75 | 4054217.16 | 128.17% | 0.04(0.03-0.05) | 27140 | 35185.11 | 29.64% | -1.58(-1.7 - -1.46) | 1106638.81 | 1298790.39 | 17.36% | -1.94(-2.05 - -1.82) |
| Ethiopia | APED | 10376.45 | 69515.56 | 569.94% | 3.74(2.61-4.87) | 823.15 | 570.86 | -30.65% | -3.26(-3.4 - -3.13) | 49276.09 | 29153.01 | -40.84% | -3.95(-4.08 - -3.82) |
| Ethiopia | COCLD | 10269.53 | 22299.51 | 117.14% | 0.18(0.11-0.26) | 16794 | 22772.46 | 35.60% | -1.51(-1.62 - -1.4) | 584042.76 | 737046.57 | 26.20% | -1.86(-1.98 - -1.75) |
| Ethiopia | GABD | 14607.33 | 43122.85 | 195.21% | 1.4(1.35-1.46) | 972.88 | 1670.78 | 71.74% | -0.7(-0.83 - -0.57) | 33607.93 | 44752.1 | 33.16% | -1.38(-1.52 - -1.25) |
| Ethiopia | IFAH | 42336.9 | 99099.84 | 134.07% | 0.78(0.75-0.81) | 679.85 | 828.15 | 21.81% | -0.86(-0.93 - -0.78) | 49209.56 | 50914.87 | 3.47% | -1.33(-1.37 - -1.29) |
| Ethiopia | IFBD | 269.81 | 710.07 | 163.17% | 0.64(0.55-0.73) | 219.08 | 324.77 | 48.24% | -0.67(-0.82 - -0.52) | 11296.24 | 12866.19 | 13.90% | -1.33(-1.48 - -1.18) |
| Ethiopia | PACA | 7235.55 | 15756.13 | 117.76% | -0.1(-0.11 - -0.08) | 454.28 | 625.05 | 37.59% | -1.64(-1.78 - -1.5) | 16842.06 | 22234.95 | 32.02% | -1.91(-2.04 - -1.77) |
| Ethiopia | PIIO | 15703.04 | 45110.08 | 187.27% | 1.14(1.1-1.18) | 2493.87 | 3821.89 | 53.25% | -0.62(-0.69 - -0.54) | 134686.28 | 167589.19 | 24.43% | -1.09(-1.17 - -1.02) |
| Ethiopia | UDSD | 1674046.83 | 3753215.59 | 124.20% | -0.017(-0.022 - -0.013) | 3324.48 | 2257.15 | -32.11% | -3.94(-4.1 - -3.78) | 173047.1 | 166976.8 | -3.51% | -3.03(-3.15 - -2.91) |
| Ethiopia | VAID | 1977.31 | 5387.54 | 172.47% | 0.86(0.7-1.01) | 287.97 | 608.43 | 111.28% | -0.19(-0.43-0.05) | 8502.62 | 13717.42 | 61.33% | -0.67(-0.91 - -0.42) |
| Fiji | Digestive diseases | 18516.89 | 26918.5 | 45.37% | 0.02(0-0.04) | 125.63 | 180.86 | 43.96% | -0.79(-0.89 - -0.69) | 5264.29 | 6724.89 | 27.75% | -0.76(-0.86 - -0.67) |
| Fiji | APED | 832.12 | 1134.84 | 36.38% | 0.53(0.45-0.61) | 1.77 | 2.99 | 68.93% | 1.05(0.54-1.56) | 88.16 | 129.31 | 46.68% | 1.06(0.59-1.53) |
| Fiji | COCLD | 79.73 | 96.16 | 20.61% | -0.43(-0.5 - -0.36) | 62.24 | 86.53 | 39.03% | -0.88(-0.98 - -0.78) | 2461.08 | 3014 | 22.47% | -0.98(-1.07 - -0.88) |
| Fiji | GABD | 836.13 | 1371.62 | 64.04% | 0.36(0.29-0.42) | 5.08 | 12.68 | 149.61% | 1.42(1.11-1.74) | 267.21 | 505.37 | 89.13% | 0.92(0.67-1.17) |
| Fiji | IFAH | 413.96 | 870.1 | 110.19% | 1.35(1.22-1.47) | 1.88 | 4.52 | 140.43% | 1.24(0.76-1.74) | 159.02 | 302.92 | 90.49% | 1.16(0.87-1.46) |
| Fiji | IFBD | 3.41 | 6.5 | 90.62% | 1.01(0.92-1.09) | 1.39 | 1.78 | 28.06% | -1.16(-1.28 - -1.03) | 59.47 | 68.2 | 14.68% | -1.08(-1.21 - -0.95) |
| Fiji | PACA | 135.14 | 200.75 | 48.55% | -0.17(-0.18 - -0.15) | 3.67 | 4.33 | 17.98% | -1.47(-1.67 - -1.28) | 153.44 | 155.52 | 1.36% | -1.72(-1.95 - -1.49) |
| Fiji | PIIO | 390.88 | 566.67 | 44.97% | 0.09(0.04-0.15) | 9.18 | 15.47 | 68.50% | -0.23(-0.38 - -0.09) | 326.09 | 460.24 | 41.14% | -0.08(-0.23-0.07) |
| Fiji | UDSD | 15795.4 | 22615.77 | 43.18% | -0.065(-0.078 - -0.052) | 33.29 | 36.38 | 9.28% | -2.05(-2.18 - -1.93) | 1492.63 | 1592.71 | 6.70% | -1.66(-1.76 - -1.56) |
| Fiji | VAID | 30.11 | 56.09 | 86.28% | 0.49(0.44-0.54) | 1.9 | 4.1 | 115.79% | 0.26(0.19-0.33) | 56.36 | 107.33 | 90.44% | 0.15(0.07-0.24) |
| Finland | Digestive diseases | 313474.6 | 380179.86 | 21.28% | -0.07(-0.14-0) | 1781.97 | 2589.11 | 45.29% | -0.32(-0.57 - -0.07) | 52993.8 | 68862.54 | 29.94% | 0.06(-0.22-0.34) |
| Finland | APED | 13580.97 | 12949.35 | -4.65% | -0.23(-0.26 - -0.2) | 21.03 | 12.36 | -41.23% | -4.38(-4.83 - -3.93) | 573.32 | 347.46 | -39.40% | -2.31(-2.56 - -2.05) |
| Finland | COCLD | 837.2 | 1124.65 | 34.33% | 1.69(1.33-2.05) | 619.48 | 1152.24 | 86.00% | 1.57(1.08-2.06) | 20205.64 | 32607.1 | 61.38% | 1.38(0.87-1.89) |
| Finland | GABD | 29139.39 | 37900.51 | 30.07% | -0.3(-0.99-0.4) | 161.13 | 205.77 | 27.70% | -2.23(-2.38 - -2.09) | 4531.7 | 4847.32 | 6.96% | -1.49(-1.91 - -1.06) |
| Finland | IFAH | 6819.97 | 8680.69 | 27.28% | -0.17(-0.4-0.07) | 64.76 | 56.84 | -12.23% | -2.89(-3.22 - -2.55) | 2234.28 | 2141.27 | -4.16% | -1.4(-1.46 - -1.34) |
| Finland | IFBD | 789.86 | 1499.7 | 89.87% | 1.58(1.37-1.79) | 33.08 | 39.31 | 18.83% | -2.22(-2.47 - -1.96) | 2012.01 | 3465.46 | 72.24% | 1.06(0.98-1.14) |
| Finland | PACA | 1983.72 | 2725.04 | 37.37% | 0.1(0.02-0.19) | 136.56 | 179.02 | 31.09% | -0.31(-0.53 - -0.08) | 4454.54 | 4711.96 | 5.78% | -0.52(-0.76 - -0.29) |
| Finland | PIIO | 7021.84 | 12808.07 | 82.40% | 0.68(0.57-0.78) | 152.74 | 271.73 | 77.91% | -0.78(-1.07 - -0.49) | 2629.05 | 3740.71 | 42.28% | -0.81(-1.08 - -0.54) |
| Finland | UDSD | 251939.9 | 300209.6 | 19.16% | -0.067(-0.091 - -0.044) | 371.41 | 210.39 | -43.35% | -4.86(-5.09 - -4.62) | 12768.19 | 10597.76 | -17.00% | -1.8(-1.91 - -1.69) |
| Finland | VAID | 1361.75 | 2282.25 | 67.60% | -0.27(-0.47 - -0.08) | 176.49 | 244.42 | 38.49% | -1.65(-1.83 - -1.46) | 2646.1 | 3250.22 | 22.83% | -1.63(-1.79 - -1.47) |
| France | Digestive diseases | 2828186.22 | 3518218.36 | 24.40% | -0.02(-0.17-0.13) | 29688.6 | 29763.19 | 0.25% | -2.23(-2.3 - -2.15) | 717242.85 | 616284.22 | -14.08% | -1.98(-2.04 - -1.92) |
| France | APED | 137645.32 | 147348.1 | 7.05% | 0.23(0.19-0.26) | 200.56 | 147.48 | -26.47% | -3.31(-3.44 - -3.19) | 5300.88 | 3937.18 | -25.73% | -1.61(-1.71 - -1.5) |
| France | COCLD | 18974.93 | 14331.19 | -24.47% | -1.55(-1.64 - -1.47) | 13685.88 | 10722.42 | -21.65% | -2.59(-2.7 - -2.47) | 377183.41 | 259455.84 | -31.21% | -2.76(-2.9 - -2.61) |
| France | GABD | 332743.92 | 359968.92 | 8.18% | -1(-1.2 - -0.79) | 1948.82 | 2394.08 | 22.85% | -1.69(-1.83 - -1.55) | 51191.47 | 49582.23 | -3.14% | -1.67(-1.82 - -1.52) |
| France | IFAH | 80461.83 | 98276.24 | 22.14% | -0.28(-0.34 - -0.23) | 972.48 | 859.96 | -11.57% | -3.33(-3.54 - -3.13) | 28388.15 | 25597.82 | -9.83% | -1.66(-1.76 - -1.55) |
| France | IFBD | 6784.22 | 8431.03 | 24.27% | 0.72(0.65-0.8) | 943.11 | 2180.91 | 131.25% | 0.74(0.46-1.02) | 23072.87 | 40856.12 | 77.07% | 1.07(0.94-1.2) |
| France | PACA | 14830.02 | 18571.14 | 25.23% | -0.29(-0.31 - -0.27) | 1116.24 | 1186 | 6.25% | -1.69(-1.81 - -1.57) | 26099.08 | 23824.54 | -8.72% | -1.51(-1.66 - -1.37) |
| France | PIIO | 82181.08 | 148312.46 | 80.47% | 0.72(0.63-0.81) | 3573.56 | 5364.99 | 50.13% | -1.18(-1.22 - -1.13) | 49338.99 | 63129.15 | 27.95% | -1.05(-1.1 - -0.99) |
| France | UDSD | 2136707.99 | 2693193.32 | 26.04% | 0.077(-0.097-0.25) | 3517.33 | 1412.9 | -59.83% | -6.07(-6.7 - -5.42) | 101358.85 | 77919.35 | -23.13% | -1.75(-2.13 - -1.36) |
| France | VAID | 17856.9 | 29785.95 | 66.80% | 0.38(0.32-0.43) | 2632.04 | 3230.97 | 22.76% | -1.81(-1.9 - -1.71) | 37409.68 | 42309.05 | 13.10% | -1.44(-1.5 - -1.38) |
| Gabon | Digestive diseases | 40323.46 | 85290.96 | 111.52% | 0.11(0.1-0.13) | 458.87 | 603.02 | 31.41% | -1.17(-1.31 - -1.02) | 16849.19 | 21752.24 | 29.10% | -1.36(-1.48 - -1.23) |
| Gabon | APED | 1973.44 | 5189.98 | 162.99% | 1.38(1.21-1.55) | 6.13 | 5.31 | -13.38% | -2.02(-2.24 - -1.79) | 325.23 | 279.49 | -14.06% | -2.09(-2.27 - -1.92) |
| Gabon | COCLD | 144.22 | 280.9 | 94.77% | 0(-0.11-0.1) | 301.57 | 397.24 | 31.72% | -1.3(-1.42 - -1.18) | 9975.01 | 13010.29 | 30.43% | -1.5(-1.62 - -1.38) |
| Gabon | GABD | 329.67 | 730.53 | 121.59% | 0.49(0.48-0.51) | 18.86 | 30.93 | 64.00% | -0.17(-0.4-0.05) | 516.13 | 782.4 | 51.59% | -0.55(-0.77 - -0.32) |
| Gabon | IFAH | 1090.26 | 2189.96 | 100.87% | 0.48(0.37-0.59) | 8.44 | 8.87 | 5.09% | -0.9(-1.05 - -0.75) | 715.28 | 820.77 | 14.75% | -0.9(-0.96 - -0.83) |
| Gabon | IFBD | 8.74 | 23.3 | 166.59% | 0.85(0.82-0.89) | 3.55 | 5.42 | 52.68% | -0.49(-0.7 - -0.28) | 144.09 | 204.64 | 42.02% | -0.71(-0.88 - -0.54) |
| Gabon | PACA | 148.04 | 286.14 | 93.29% | -0.12(-0.12 - -0.11) | 9.67 | 13.76 | 42.30% | -1.14(-1.26 - -1.02) | 344.67 | 490.4 | 42.28% | -1.36(-1.47 - -1.25) |
| Gabon | PIIO | 1174.62 | 2694.31 | 129.38% | 0.84(0.72-0.95) | 30.92 | 42.82 | 38.50% | -0.63(-0.78 - -0.48) | 1457.7 | 1669.33 | 14.52% | -1.08(-1.2 - -0.95) |
| Gabon | UDSD | 35377.47 | 73720.94 | 108.38% | 0.022(0.018-0.026) | 47.55 | 48.32 | 1.62% | -1.79(-2.06 - -1.53) | 2378.23 | 3131.91 | 31.69% | -1.37(-1.5 - -1.23) |
| Gabon | VAID | 77.01 | 174.91 | 127.13% | 0.85(0.76-0.94) | 9.29 | 16.28 | 75.24% | -0.06(-0.24-0.11) | 224.11 | 362.05 | 61.55% | -0.35(-0.51 - -0.18) |
| Gambia | Digestive diseases | 34230.22 | 87088.65 | 154.42% | 0(-0.01-0.01) | 325.2 | 625.05 | 92.21% | -1.34(-1.54 - -1.14) | 14031.01 | 24363.86 | 73.64% | -1.34(-1.55 - -1.12) |
| Gambia | APED | 1261.73 | 3820.44 | 202.79% | 0.71(0.55-0.87) | 6.12 | 4.92 | -19.61% | -3.51(-3.84 - -3.19) | 357.99 | 238.24 | -33.45% | -4.22(-4.58 - -3.86) |
| Gambia | COCLD | 208.76 | 492.71 | 136.02% | -0.07(-0.12 - -0.02) | 173.52 | 357.43 | 105.99% | -1.19(-1.38 - -1) | 6278.1 | 12242.06 | 95.00% | -1.14(-1.36 - -0.92) |
| Gambia | GABD | 292.48 | 758.83 | 159.45% | 0.37(0.35-0.38) | 8.53 | 21.86 | 156.27% | -0.33(-0.48 - -0.18) | 364.95 | 706.47 | 93.58% | -0.63(-0.83 - -0.42) |
| Gambia | IFAH | 1221.87 | 2852.77 | 133.48% | -0.11(-0.17 - -0.04) | 6.44 | 11.38 | 76.71% | -1.53(-1.78 - -1.27) | 615.48 | 1113.23 | 80.87% | -0.99(-1.14 - -0.85) |
| Gambia | IFBD | 5.78 | 19.24 | 232.87% | 1.15(1.07-1.23) | 2.74 | 5.08 | 85.40% | -1.52(-1.81 - -1.24) | 132.04 | 214.82 | 62.69% | -1.56(-1.85 - -1.28) |
| Gambia | PACA | 155.9 | 386.9 | 148.17% | -0.09(-0.11 - -0.07) | 16.26 | 36.61 | 125.15% | -1.03(-1.32 - -0.74) | 637.22 | 1378.53 | 116.34% | -1.02(-1.32 - -0.72) |
| Gambia | PIIO | 777.07 | 2034.02 | 161.75% | 0.3(0.26-0.35) | 45.14 | 81.5 | 80.54% | -1.31(-1.58 - -1.03) | 2295.71 | 3288.58 | 43.25% | -1.55(-1.84 - -1.26) |
| Gambia | UDSD | 30255.36 | 76592.09 | 153.15% | -0.024(-0.028 - -0.019) | 46.5 | 73.12 | 57.25% | -1.86(-2.05 - -1.68) | 2476.66 | 4062.38 | 64.03% | -1.58(-1.75 - -1.4) |
| Gambia | VAID | 51.27 | 131.65 | 156.78% | 0.31(0.25-0.36) | 3.67 | 8.36 | 127.79% | -0.53(-0.78 - -0.27) | 153.69 | 235.32 | 53.11% | -1.04(-1.31 - -0.76) |
| Georgia | Digestive diseases | 311520.55 | 244050.3 | -21.66% | 0.16(0.14-0.19) | 2210.92 | 2138.78 | -3.26% | 0.56(0.31-0.82) | 77789.56 | 68095.62 | -12.46% | 0.6(0.38-0.81) |
| Georgia | APED | 11556.78 | 7515.6 | -34.97% | 0.38(0.3-0.46) | 9.21 | 8.8 | -4.45% | 1.44(0.43-2.47) | 476.75 | 327.35 | -31.34% | 0.72(0.1-1.34) |
| Georgia | COCLD | 1833.26 | 1247.73 | -31.94% | -0.17(-0.29 - -0.04) | 1719.44 | 1562.03 | -9.15% | 0.38(0.2-0.57) | 51931.18 | 46391.1 | -10.67% | 0.71(0.5-0.92) |
| Georgia | GABD | 22845.63 | 21471.02 | -6.02% | 0.87(0.69-1.05) | 28.86 | 29.27 | 1.42% | -0.34(-0.68-0) | 3023.86 | 2702.16 | -10.64% | 0.56(0.35-0.78) |
| Georgia | IFAH | 7631.91 | 8824.65 | 15.63% | 2.32(1.74-2.9) | 24.79 | 32.68 | 31.83% | 0.85(-0.08-1.78) | 2585.34 | 2443.09 | -5.50% | 0.75(0.31-1.2) |
| Georgia | IFBD | 381.42 | 275.43 | -27.79% | -0.16(-0.21 - -0.11) | 16.47 | 17.03 | 3.40% | 0.18(-1.12-1.5) | 1222.29 | 990.53 | -18.96% | 0.02(-0.38-0.41) |
| Georgia | PACA | 1604.45 | 1287.3 | -19.77% | 0.01(-0.14-0.15) | 22.6 | 43.08 | 90.62% | 3.35(2.53-4.17) | 666.43 | 1130.32 | 69.61% | 3.7(2.91-4.49) |
| Georgia | PIIO | 5494.54 | 4292.16 | -21.88% | -0.43(-0.58 - -0.27) | 77.53 | 131.41 | 69.49% | 2.14(1.52-2.77) | 3224.78 | 2960.56 | -8.19% | 0.26(-0.18-0.71) |
| Georgia | UDSD | 259494.7 | 198425.45 | -23.53% | 0.026(0.022-0.03) | 270.91 | 200.11 | -26.13% | -0.32(-0.91-0.26) | 13568.12 | 8887.28 | -34.50% | -0.46(-0.73 - -0.19) |
| Georgia | VAID | 677.86 | 710.96 | 4.88% | 1.11(0.85-1.36) | 26.19 | 61.82 | 136.04% | 3.32(2.41-4.23) | 508.18 | 1045.47 | 105.73% | 3.51(2.6-4.42) |
| Germany | Digestive diseases | 4699832.43 | 5548558.49 | 18.06% | 0.13(0.01-0.24) | 42168.91 | 48419.68 | 14.82% | -1.39(-1.48 - -1.29) | 1175803.89 | 1105172.14 | -6.01% | -1.59(-1.71 - -1.48) |
| Germany | APED | 218704.13 | 259081.69 | 18.46% | 0.99(0.74-1.23) | 481.73 | 244.82 | -49.18% | -3.67(-4.31 - -3.03) | 11803.46 | 6935.33 | -41.24% | -1.55(-1.86 - -1.24) |
| Germany | COCLD | 29343.89 | 25398.93 | -13.44% | -0.62(-0.79 - -0.45) | 22262.84 | 20491.64 | -7.96% | -2.12(-2.32 - -1.92) | 647780.94 | 498183.6 | -23.09% | -2.57(-2.79 - -2.34) |
| Germany | GABD | 747251.68 | 830297.57 | 11.11% | -0.31(-0.88-0.26) | 2584.79 | 3027.97 | 17.15% | -0.64(-1.32-0.04) | 101042.78 | 95500.66 | -5.48% | -0.96(-1.2 - -0.71) |
| Germany | IFAH | 96690.33 | 113808.49 | 17.70% | 1.21(0.83-1.58) | 1200.97 | 704.25 | -41.36% | -3.14(-4.45 - -1.82) | 36704.28 | 27830.3 | -24.18% | -0.47(-0.96-0.03) |
| Germany | IFBD | 9760.35 | 16745.6 | 71.57% | 1.14(0.86-1.41) | 1201.42 | 4285.69 | 256.72% | 3.73(3.26-4.2) | 37493.44 | 92002.21 | 145.38% | 1.8(1.57-2.02) |
| Germany | PACA | 24979.61 | 37430.85 | 49.85% | 0.1(-0.12-0.32) | 1994.49 | 2173.32 | 8.97% | -1.36(-1.57 - -1.14) | 52313.89 | 47104.35 | -9.96% | -1.67(-1.88 - -1.45) |
| Germany | PIIO | 119320.71 | 197302.82 | 65.36% | 0.49(0.36-0.63) | 2651.33 | 5682.3 | 114.32% | 0.76(0.57-0.95) | 42505.59 | 75198.75 | 76.91% | 0.71(0.24-0.61) |
| Germany | UDSD | 3410547.17 | 4004930.96 | 17.43% | 0.082(0.065-0.099) | 4927.48 | 3980.34 | -19.22% | -2.83(-2.97 - -2.69) | 165444.6 | 147721.67 | -10.71% | -1.19(-1.28 - -1.1) |
| Germany | VAID | 43234.56 | 63561.58 | 47.02% | 0.61(0.33-0.89) | 4211.06 | 4757.4 | 12.97% | -1.76(-2 - -1.51) | 66337.12 | 67665.03 | 2.00% | -1.71(-1.88 - -1.54) |
| Ghana | Digestive diseases | 541910.26 | 1386023.35 | 155.77% | 0.11(0.1-0.13) | 6029.78 | 10229.14 | 69.64% | -1.47(-1.54 - -1.41) | 243137.55 | 408611.42 | 68.06% | -1.31(-1.37 - -1.25) |
| Ghana | APED | 19917.46 | 55052.34 | 176.40% | 0.62(0.53-0.72) | 122.93 | 157.1 | 27.80% | -1.78(-1.95 - -1.62) | 6809.19 | 8123.06 | 19.30% | -1.67(-1.83 - -1.52) |
| Ghana | COCLD | 3065.53 | 6227.05 | 103.13% | -0.62(-0.7 - -0.54) | 4065.77 | 6124.55 | 50.64% | -2.01(-2.12 - -1.91) | 145131.3 | 213288.04 | 46.96% | -1.94(-2.04 - -1.83) |
| Ghana | GABD | 4579.33 | 12492.98 | 172.81% | 0.74(0.7-0.77) | 146.21 | 450.29 | 207.97% | 1.27(0.98-1.57) | 5916.41 | 15470.24 | 161.48% | 1.11(0.82-1.4) |
| Ghana | IFAH | 15107.27 | 40915.97 | 170.84% | 0.72(0.57-0.87) | 91.28 | 189.28 | 107.36% | -0.47(-0.66 - -0.28) | 7645.21 | 15773.38 | 106.32% | -0.11(-0.23-0.01) |
| Ghana | IFBD | 103.93 | 306.95 | 195.34% | 0.43(0.28-0.58) | 55.84 | 102.87 | 84.22% | -0.97(-1.23 - -0.7) | 2632.7 | 4506.13 | 71.16% | -1.02(-1.27 - -0.77) |
| Ghana | PACA | 2417.9 | 6041.94 | 149.88% | 0.05(0.02-0.08) | 298.8 | 754.19 | 152.41% | -0.17(-0.34 - -0.01) | 11180.09 | 28361.45 | 153.68% | -0.03(-0.19-0.13) |
| Ghana | PIIO | 12580.57 | 36073.69 | 186.74% | 0.62(0.54-0.71) | 627.93 | 1233.07 | 96.37% | -0.84(-1.03 - -0.65) | 27811.87 | 49392.39 | 77.59% | -0.6(-0.77 - -0.43) |
| Ghana | UDSD | 483339.06 | 1226740.65 | 153.81% | 0.066(0.058-0.073) | 244.72 | 372.1 | 52.05% | -1.5(-1.68 - -1.31) | 20291.05 | 41893.56 | 106.46% | -0.51(-0.57 - -0.45) |
| Ghana | VAID | 799.21 | 2171.8 | 171.74% | 0.64(0.5-0.79) | 61.59 | 131.26 | 113.12% | -0.33(-0.68-0.03) | 2653.05 | 4209.52 | 58.67% | -1.06(-1.55 - -0.57) |
| Greece | Digestive diseases | 615716.16 | 711589.63 | 15.57% | 0.06(0.06-0.07) | 2969.41 | 4074.88 | 37.23% | -1.09(-1.26 - -0.92) | 79054.5 | 87725.09 | 10.97% | -0.65(-0.78 - -0.52) |
| Greece | APED | 24064.82 | 20999.48 | -12.74% | 0.24(0.18-0.29) | 19.57 | 18.03 | -7.87% | -2.6(-3.53 - -1.66) | 655.9 | 514.44 | -21.57% | -0.86(-1.26 - -0.45) |
| Greece | COCLD | 2627.17 | 1803.36 | -31.36% | -1.55(-1.65 - -1.46) | 1748.29 | 1366.43 | -21.84% | -2.45(-2.69 - -2.21) | 38304.66 | 29349.22 | -23.38% | -1.72(-1.95 - -1.49) |
| Greece | GABD | 48747.58 | 68206.77 | 39.92% | 0.38(0.33-0.44) | 156.74 | 451.63 | 188.14% | 1.52(1-2.04) | 6066.49 | 9970.84 | 64.36% | 0.54(0.28-0.8) |
| Greece | IFAH | 11380.31 | 13727.37 | 20.62% | 0.37(0.29-0.45) | 53.54 | 68.75 | 28.41% | -1.58(-1.79 - -1.36) | 2999.13 | 3264.72 | 8.86% | -0.28(-0.33 - -0.22) |
| Greece | IFBD | 908.12 | 911.34 | 0.35% | -0.09(-0.17 - -0.01) | 35.47 | 66.25 | 86.78% | -0.36(-0.92-0.2) | 2407.41 | 2423.96 | 0.69% | -0.78(-0.85 - -0.71) |
| Greece | PACA | 2754.28 | 3482.92 | 26.45% | -0.13(-0.18 - -0.07) | 203.36 | 349.39 | 71.81% | -0.75(-1.15 - -0.36) | 4121.09 | 5577.31 | 35.34% | -0.58(-0.84 - -0.32) |
| Greece | PIIO | 15913.99 | 22743.7 | 42.92% | 0.1(0-0.2) | 233.07 | 634.45 | 172.21% | 0.79(0.67-0.92) | 3705.62 | 7797.09 | 110.41% | 0.75(0.64-0.85) |
| Greece | UDSD | 507221.53 | 575994.93 | 13.56% | 0.02(0.014-0.026) | 319.76 | 670.87 | 109.80% | 0.1(0.01-0.19) | 17207.68 | 22382.48 | 30.07% | 0.06(0.03-0.09) |
| Greece | VAID | 2098.37 | 3719.77 | 77.27% | 0.7(0.68-0.72) | 152.26 | 345.59 | 126.97% | 0.07(-0.26-0.4) | 2474.42 | 4599.73 | 85.89% | 0.15(-0.18-0.49) |
| Greenland | Digestive diseases | 2589.45 | 3135.7 | 21.10% | -0.03(-0.04 - -0.02) | 18.58 | 24.42 | 31.39% | -1.48(-1.64 - -1.33) | 717.21 | 781.04 | 8.90% | -1.42(-1.51 - -1.32) |
| Greenland | APED | 98.07 | 101.81 | 3.81% | 0.35(0.31-0.39) | 0.08 | 0.16 | 100.00% | 0.88(0.37-1.4) | 4.49 | 5.93 | 32.07% | 0.02(-0.42-0.48) |
| Greenland | COCLD | 9.91 | 9.14 | -7.77% | 0.25(0.2-0.29) | 5.97 | 8.57 | 43.55% | -0.37(-0.46 - -0.29) | 228.82 | 285.87 | 24.93% | -0.48(-0.58 - -0.38) |
| Greenland | GABD | 309.59 | 413.19 | 33.46% | 0.06(0.02-0.09) | 0.75 | 1.11 | 48.00% | -1.59(-1.83 - -1.35) | 53.37 | 63.46 | 18.91% | -1.09(-1.2 - -0.97) |
| Greenland | IFAH | 41.12 | 65.81 | 60.04% | 0.92(0.86-0.99) | 0.18 | 0.4 | 122.22% | 1.12(0.87-1.36) | 15.59 | 22.22 | 42.53% | 0.33(0.22-0.44) |
| Greenland | IFBD | 11.28 | 15.43 | 36.79% | 0.72(0.59-0.84) | 0.17 | 0.42 | 147.06% | 1.07(0.8-1.34) | 16.92 | 25.23 | 49.11% | 0.36(0.25-0.47) |
| Greenland | PACA | 27.87 | 36.25 | 30.07% | -0.14(-0.16 - -0.13) | 0.9 | 0.91 | 1.11% | -2.09(-2.36 - -1.82) | 35.21 | 29.72 | -15.59% | -2.26(-2.5 - -2.02) |
| Greenland | PIIO | 40.55 | 73.5 | 81.26% | 0.68(0.62-0.75) | 1.37 | 2.71 | 97.72% | -0.02(-0.2-0.16) | 51.35 | 60.9 | 18.61% | -0.77(-0.9 - -0.64) |
| Greenland | UDSD | 2032.93 | 2382.53 | 17.20% | -0.118(-0.133 - -0.102) | 6.78 | 5.42 | -20.06% | -3.58(-3.97 - -3.19) | 249.16 | 184.24 | -26.06% | -3.13(-3.44 - -2.82) |
| Greenland | VAID | 18.14 | 38.05 | 109.76% | 0.72(0.64-0.81) | 1.06 | 2.28 | 115.09% | -0.47(-0.71 - -0.24) | 25.4 | 49.55 | 95.08% | -0.46(-0.67 - -0.25) |
| Grenada | Digestive diseases | 5307.09 | 8480.37 | 59.79% | 0.07(0.06-0.08) | 32.68 | 36.86 | 12.78% | -0.98(-1.15 - -0.81) | 1063.66 | 1296.1 | 21.85% | -0.96(-1.12 - -0.8) |
| Grenada | APED | 190.63 | 299.76 | 57.25% | 1.08(1.01-1.16) | 0.48 | 0.38 | -20.83% | -1.04(-1.67 - -0.41) | 21.2 | 16.15 | -23.82% | -1.1(-1.66 - -0.54) |
| Grenada | COCLD | 13.21 | 24.3 | 83.95% | -0.16(-0.25 - -0.08) | 15.61 | 18.33 | 17.42% | -1.34(-1.54 - -1.14) | 444.71 | 537.36 | 20.83% | -1.48(-1.7 - -1.26) |
| Grenada | GABD | 227.14 | 348.63 | 53.49% | -0.07(-0.13 - -0.01) | 2.01 | 1.59 | -20.90% | -1.74(-2.07 - -1.41) | 72.88 | 74.87 | 2.73% | -1.41(-1.67 - -1.15) |
| Grenada | IFAH | 157.41 | 287.15 | 82.42% | 1.07(1.05-1.1) | 0.69 | 0.75 | 8.70% | -0.24(-0.47-0) | 61.58 | 79.48 | 29.07% | 0.1(-0.01-0.22) |
| Grenada | IFBD | 1.58 | 3.39 | 114.56% | 0.73(0.57-0.88) | 1.02 | 1.32 | 29.41% | -0.55(-0.68 - -0.41) | 32.67 | 42.76 | 30.88% | -0.64(-0.73 - -0.56) |
| Grenada | PACA | 20.58 | 28.95 | 40.67% | -0.2(-0.22 - -0.17) | 0.71 | 0.99 | 39.44% | -0.4(-0.5 - -0.3) | 22.33 | 31.83 | 42.54% | -0.56(-0.64 - -0.47) |
| Grenada | PIIO | 70.23 | 107.81 | 53.51% | 0.58(0.47-0.69) | 4.42 | 4.67 | 5.67% | -0.35(-0.47 - -0.23) | 118.43 | 121.01 | 2.18% | -0.56(-0.67 - -0.45) |
| Grenada | UDSD | 4620.73 | 7369.55 | 59.49% | -0.002(-0.005-0.001) | 6.03 | 4.51 | -25.21% | -2.34(-2.58 - -2.1) | 246.44 | 286.7 | 16.34% | -1.2(-1.34 - -1.05) |
| Grenada | VAID | 5.59 | 10.83 | 93.74% | 1.13(1.08-1.18) | 0.51 | 0.71 | 39.22% | 0.43(0.2-0.65) | 9.31 | 14.94 | 60.47% | 0.32(0.06-0.57) |
| Guam | Digestive diseases | 3736.54 | 5467.58 | 46.33% | -0.03(-0.05 - -0.01) | 26.6 | 44.59 | 67.63% | -1.68(-2 - -1.36) | 1045.91 | 1468.71 | 40.42% | -0.98(-1.18 - -0.78) |
| Guam | APED | 145.52 | 193.16 | 32.74% | 0.41(0.34-0.48) | 0.17 | 0.16 | -5.88% | -3.28(-3.73 - -2.83) | 8.2 | 7.21 | -12.07% | -2(-2.28 - -1.71) |
| Guam | COCLD | 30.7 | 31.78 | 3.52% | -0.88(-1 - -0.76) | 20.53 | 33.02 | 60.84% | -1.47(-1.72 - -1.22) | 744.89 | 1023.89 | 37.46% | -0.99(-1.16 - -0.83) |
| Guam | GABD | 184.54 | 286.71 | 55.36% | -0.06(-0.12-0) | 0.59 | 1.43 | 142.37% | -1.29(-1.59 - -0.99) | 31.63 | 56.23 | 77.77% | -0.34(-0.49 - -0.19) |
| Guam | IFAH | 113.77 | 187.35 | 64.67% | -0.34(-0.6 - -0.08) | 0.41 | 0.67 | 63.41% | -3.03(-4.02 - -2.02) | 32.53 | 50.09 | 53.98% | -1.01(-1.49 - -0.52) |
| Guam | IFBD | 0.7 | 1.34 | 91.43% | 1.19(1.08-1.31) | 0.23 | 0.28 | 21.74% | -3(-3.55 - -2.45) | 9.13 | 9.4 | 2.96% | -2.12(-2.65 - -1.59) |
| Guam | PACA | 28.04 | 44.49 | 58.67% | -0.07(-0.1 - -0.04) | 0.51 | 1.05 | 105.88% | -0.87(-1.24 - -0.51) | 17.93 | 34.32 | 91.41% | 0.39(0.11-0.66) |
| Guam | PIIO | 128.7 | 198.64 | 54.35% | -0.37(-0.59 - -0.16) | 0.81 | 2.48 | 204.60% | -1.02(-1.44 - -0.61) | 30.77 | 50.5 | 64.14% | -0.46(-0.69 - -0.22) |
| Guam | UDSD | 3096.2 | 4506.63 | 45.55% | -0.009(-0.016 - -0.001) | 2.46 | 2.8 | 13.82% | -4.46(-5.05 - -3.87) | 144.29 | 173.18 | 20.02% | -1.69(-1.98 - -1.41) |
| Guam | VAID | 8.38 | 17.49 | 108.71% | 0.24(0.19-0.29) | 0.38 | 1.08 | 184.21% | -1(-1.42 - -0.57) | 8.58 | 21.75 | 153.50% | -0.08(-0.47-0.31) |
| Guatemala | Digestive diseases | 488876.76 | 1274198.37 | 160.64% | -0.1(-0.15 - -0.04) | 4714.26 | 8569.99 | 81.79% | -1.44(-1.61 - -1.27) | 243386.65 | 338831.31 | 39.22% | -1.73(-1.92 - -1.55) |
| Guatemala | APED | 60111.61 | 85531.17 | 42.29% | -2.09(-2.09 - -1.46) | 416.02 | 159.79 | -61.59% | -5.61(-6.22 - -5.01) | 30207.24 | 8951.79 | -70.37% | -6.32(-7.03 - -5.61) |
| Guatemala | COCLD | 2898.88 | 8092.29 | 179.15% | 0.47(0.44-0.5) | 2345.73 | 5520.71 | 135.35% | -0.69(-0.89 - -0.49) | 96351.92 | 191533.28 | 98.79% | -1.05(-1.26 - -0.84) |
| Guatemala | GABD | 42056.66 | 134238.79 | 219.19% | 0.63(0.59-0.67) | 156.49 | 256.05 | 63.62% | -1.58(-2.3 - -0.86) | 12686.84 | 22194.8 | 74.94% | -1.4(-1.8 - -0.99) |
| Guatemala | IFAH | 20215.43 | 37956.84 | 87.76% | -0.52(-0.62 - -0.43) | 159.53 | 184.02 | 15.35% | -2.41(-2.76 - -2.05) | 15267.22 | 14632.98 | -4.15% | -2.36(-2.69 - -2.04) |
| Guatemala | IFBD | 130.08 | 358.26 | 175.42% | -0.18(-0.31 - -0.05) | 12.63 | 35.4 | 180.29% | 1.32(0.72-1.92) | 865.92 | 1760.83 | 103.35% | 0.71(0.18-1.24) |
| Guatemala | PACA | 2398.45 | 5915.49 | 146.64% | -0.3(-0.32 - -0.28) | 230.46 | 395.93 | 71.80% | -2(-2.17 - -1.83) | 10745.33 | 16522.18 | 53.76% | -2.03(-2.22 - -1.84) |
| Guatemala | PIIO | 6054.09 | 14089.85 | 132.73% | 0.64(0.64-0.76) | 495.15 | 478.44 | -3.38% | -1.94(-2.27 - -1.62) | 35573.72 | 19721.75 | -44.56% | -3.22(-3.81 - -2.63) |
| Guatemala | UDSD | 354639.55 | 986843.86 | 178.27% | -0.003(-0.013-0.008) | 753.85 | 1156.76 | 53.45% | -3.14(-3.62 - -2.66) | 32605.95 | 50682.48 | 55.44% | -2.5(-2.8 - -2.19) |
| Guatemala | VAID | 372.01 | 1171.81 | 214.99% | 1.04(0.96-1.11) | 28.2 | 74.67 | 164.79% | -0.73(-0.85 - -0.61) | 954.11 | 1615.89 | 69.36% | -1.13(-1.34 - -0.92) |
| Guinea | Digestive diseases | 228804.19 | 464283.03 | 102.92% | 0.05(0.04-0.06) | 2788.79 | 3915.42 | 40.40% | -0.56(-0.68 - -0.43) | 114896.77 | 163199.13 | 42.04% | -0.55(-0.64 - -0.45) |
| Guinea | APED | 6894.14 | 19753.08 | 186.52% | 0.89(0.73-1.05) | 55.69 | 39.99 | -28.19% | -2.56(-2.76 - -2.36) | 3488.51 | 2321.29 | -33.46% | -3.23(-3.44 - -3.01) |
| Guinea | COCLD | 1262.34 | 2419.03 | 91.63% | -0.41(-0.56 - -0.25) | 1686.11 | 2381.88 | 41.26% | -0.63(-0.77 - -0.48) | 57561.22 | 85732.38 | 48.94% | -0.51(-0.64 - -0.38) |
| Guinea | GABD | 1755.15 | 3915.49 | 123.09% | 0.43(0.42-0.45) | 72.98 | 128.96 | 76.71% | 0.39(0.23-0.55) | 3052.06 | 4950.4 | 62.20% | 0.02(-0.09-0.13) |
| Guinea | IFAH | 6879.98 | 15372.81 | 123.44% | 0.65(0.6-0.71) | 56.56 | 74.34 | 31.44% | -0.25(-0.37 - -0.12) | 4930.31 | 7236.29 | 46.77% | -0.2(-0.23 - -0.16) |
| Guinea | IFBD | 42.2 | 90.64 | 114.79% | 0.16(0.07-0.26) | 19.23 | 28.78 | 49.66% | -0.36(-0.5 - -0.22) | 1007.59 | 1414.5 | 40.38% | -0.58(-0.7 - -0.47) |
| Guinea | PACA | 1018.51 | 1974.14 | 93.83% | -0.05(-0.06 - -0.03) | 99.88 | 182.58 | 82.80% | 0.02(-0.15-0.18) | 3681.57 | 6950.88 | 88.80% | 0.14(-0.01-0.29) |
| Guinea | PIIO | 4542.78 | 9687.65 | 113.25% | 0.49(0.44-0.54) | 296.1 | 419.51 | 41.68% | -0.35(-0.49 - -0.21) | 16152.39 | 20280.06 | 25.55% | -0.7(-0.81 - -0.59) |
| Guinea | UDSD | 206090.04 | 410373.07 | 99.12% | -0.003(-0.007-0.002) | 356.8 | 480.07 | 34.55% | -0.5(-0.61 - -0.39) | 18157.76 | 26959.36 | 48.47% | -0.46(-0.53 - -0.38) |
| Guinea | VAID | 319.03 | 697.11 | 118.51% | 0.48(0.4-0.56) | 28.86 | 46.27 | 60.33% | 0.34(0.2-0.47) | 1295.82 | 1587.81 | 22.53% | -0.37(-0.46 - -0.27) |
| Guinea-Bissau | Digestive diseases | 35102.79 | 72967.14 | 107.87% | 0.02(0.01-0.03) | 520.61 | 738.84 | 41.92% | -0.62(-0.68 - -0.56) | 22069.1 | 30843.31 | 39.76% | -0.69(-0.76 - -0.61) |
| Guinea-Bissau | APED | 1257.75 | 3062.75 | 143.51% | 0.64(0.46-0.83) | 11.56 | 6.91 | -40.22% | -2.87(-3.02 - -2.71) | 677.79 | 348.08 | -48.64% | -3.73(-3.91 - -3.55) |
| Guinea-Bissau | COCLD | 238.89 | 429.94 | 79.97% | -0.54(-0.59 - -0.49) | 298.52 | 449.4 | 50.54% | -0.58(-0.64 - -0.51) | 10980.17 | 16889.67 | 53.82% | -0.54(-0.63 - -0.45) |
| Guinea-Bissau | GABD | 271.99 | 602.05 | 121.35% | 0.59(0.57-0.62) | 12.63 | 20.95 | 65.87% | 0.37(0.25-0.5) | 561.89 | 828.58 | 47.46% | -0.09(-0.23-0.06) |
| Guinea-Bissau | IFAH | 1097.75 | 2355.66 | 114.59% | 0.36(0.3-0.42) | 10.36 | 12.39 | 19.59% | -0.88(-0.96 - -0.79) | 825.91 | 1145.65 | 38.71% | -0.62(-0.69 - -0.55) |
| Guinea-Bissau | IFBD | 6.54 | 15.05 | 130.12% | 0.31(0.21-0.41) | 4.12 | 5.77 | 40.05% | -0.81(-0.93 - -0.68) | 200.69 | 264.61 | 31.85% | -0.94(-1.07 - -0.8) |
| Guinea-Bissau | PACA | 165.4 | 321.15 | 94.17% | -0.15(-0.17 - -0.13) | 24.73 | 44.5 | 79.94% | -0.23(-0.37 - -0.09) | 961.13 | 1756.72 | 82.78% | -0.22(-0.36 - -0.07) |
| Guinea-Bissau | PIIO | 584.59 | 1275.83 | 118.24% | 0.62(0.54-0.7) | 50.55 | 72.65 | 43.72% | -0.21(-0.34 - -0.09) | 2643.85 | 3306.29 | 25.06% | -0.48(-0.61 - -0.34) |
| Guinea-Bissau | UDSD | 31432.62 | 64806.85 | 106.18% | -0.018(-0.022 - -0.015) | 78.6 | 93.8 | 19.34% | -0.87(-0.99 - -0.75) | 3871.01 | 5046.39 | 30.36% | -0.96(-1.06 - -0.85) |
| Guinea-Bissau | VAID | 47.26 | 97.86 | 107.07% | 0.37(0.34-0.41) | 4.8 | 7.5 | 56.25% | 0.41(0.28-0.55) | 214.14 | 252.21 | 17.78% | -0.28(-0.4 - -0.17) |
| Guyana | Digestive diseases | 46572.2 | 57505.83 | 23.48% | 0.05(0.04-0.06) | 356.43 | 393.09 | 10.28% | -0.84(-1.06 - -0.61) | 14588.37 | 14983.95 | 2.71% | -0.73(-0.9 - -0.56) |
| Guyana | APED | 1959.07 | 2443.02 | 24.70% | 0.64(0.48-0.81) | 12.28 | 9.02 | -26.55% | -0.92(-1.41 - -0.44) | 633.29 | 407.85 | -35.60% | -0.8(-1.22 - -0.39) |
| Guyana | COCLD | 214.74 | 262.79 | 22.38% | -0.02(-0.09-0.06) | 218.45 | 250.85 | 14.83% | -0.81(-1.04 - -0.58) | 7791.04 | 8418.77 | 8.06% | -0.83(-1.03 - -0.63) |
| Guyana | GABD | 1917.87 | 2350.79 | 22.57% | 0.02(-0.05-0.08) | 13.76 | 12.26 | -10.90% | -1.49(-1.95 - -1.03) | 668.2 | 639.14 | -4.35% | -1.06(-1.34 - -0.79) |
| Guyana | IFAH | 1640.64 | 1990.26 | 21.31% | 0.18(0.11-0.25) | 8.93 | 7.46 | -16.46% | -1.02(-1.27 - -0.77) | 904.71 | 720.21 | -20.39% | -0.76(-0.88 - -0.64) |
| Guyana | IFBD | 14.37 | 21.89 | 52.33% | 0.47(0.3-0.65) | 5.37 | 6.45 | 20.11% | -0.53(-0.7 - -0.35) | 239.49 | 256.78 | 7.22% | -0.46(-0.59 - -0.32) |
| Guyana | PACA | 175.01 | 215.2 | 22.96% | -0.01(-0.04-0.02) | 10.71 | 15.81 | 47.62% | 0.35(0.14-0.56) | 419.13 | 584.24 | 39.39% | 0.42(0.26-0.59) |
| Guyana | PIIO | 407.44 | 511.83 | 25.62% | 0.27(0.18-0.36) | 20.64 | 22.67 | 9.85% | -0.23(-0.43 - -0.03) | 931.78 | 774.66 | -16.86% | -0.24(-0.44 - -0.04) |
| Guyana | UDSD | 40203.36 | 49648.27 | 23.49% | 0.024(0.022-0.026) | 56.26 | 45.18 | -19.69% | -2.09(-2.36 - -1.81) | 2639.05 | 2477.4 | -6.13% | -1.2(-1.37 - -1.03) |
| Guyana | VAID | 39.69 | 61.78 | 55.66% | 0.63(0.53-0.73) | 3.84 | 5.81 | 51.30% | -0.01(-0.27-0.26) | 99.55 | 149.96 | 50.64% | 0.19(-0.05-0.43) |
| Haiti | Digestive diseases | 356288.32 | 808355.42 | 126.88% | 0.09(0.09-0.1) | 3306.38 | 4384.03 | 32.59% | -1.26(-1.37 - -1.14) | 155201.13 | 191268.05 | 23.24% | -1.3(-1.44 - -1.15) |
| Haiti | APED | 11128.47 | 30099.1 | 170.47% | 0.92(0.76-1.08) | 202.75 | 176.88 | -12.76% | -1.92(-2.21 - -1.63) | 13017.77 | 9813.29 | -24.62% | -2.28(-2.64 - -1.92) |
| Haiti | COCLD | 1084.26 | 2778.57 | 156.26% | 0.55(0.51-0.6) | 1474.47 | 2151.4 | 45.91% | -1.15(-1.28 - -1.02) | 53902.73 | 73339.95 | 36.06% | -1.28(-1.43 - -1.12) |
| Haiti | GABD | 12004.57 | 30022.21 | 150.09% | 0.72(0.65-0.79) | 136.16 | 188.93 | 38.76% | -1.24(-1.4 - -1.08) | 7321.84 | 10744.85 | 46.75% | -1.15(-1.27 - -1.03) |
| Haiti | IFAH | 14440.31 | 30213.97 | 109.23% | 0.51(0.47-0.56) | 188.85 | 162.18 | -14.12% | -1.61(-1.79 - -1.43) | 17824.04 | 17448.71 | -2.11% | -1.36(-1.51 - -1.21) |
| Haiti | IFBD | 96.07 | 263.34 | 174.11% | 0.49(0.33-0.65) | 58.33 | 85.03 | 45.77% | -0.77(-0.89 - -0.65) | 3029.56 | 3782.37 | 24.85% | -0.94(-1.12 - -0.77) |
| Haiti | PACA | 1352.96 | 2925.01 | 116.19% | -0.03(-0.04 - -0.02) | 94.7 | 156.69 | 65.46% | -0.8(-0.93 - -0.68) | 3633.5 | 5881.51 | 61.87% | -0.83(-0.98 - -0.68) |
| Haiti | PIIO | 2100.82 | 4766.08 | 126.87% | 0.7(0.67-0.74) | 300.09 | 446.23 | 48.70% | -0.58(-0.66 - -0.5) | 17407.89 | 20354.11 | 16.92% | -0.68(-0.73 - -0.63) |
| Haiti | UDSD | 313821.99 | 706684.02 | 125.19% | 0.022(0.019-0.026) | 711.44 | 717.12 | 0.80% | -2.19(-2.31 - -2.07) | 32722.79 | 38769.79 | 18.48% | -1.82(-1.96 - -1.68) |
| Haiti | VAID | 258.87 | 603.11 | 132.98% | 0.64(0.57-0.7) | 38.97 | 62.39 | 60.10% | -1.11(-1.28 - -0.95) | 1029.13 | 1578.31 | 53.36% | -0.97(-1.16 - -0.77) |
| Honduras | Digestive diseases | 275123 | 714340.54 | 159.64% | 0.02(0.01-0.04) | 2791.89 | 5661.73 | 102.79% | 0.14(-0.02-0.29) | 154783.55 | 198610.25 | 28.31% | -1.01(-1.08 - -0.93) |
| Honduras | APED | 25517 | 49979.88 | 95.87% | -0.28(-0.4 - -0.16) | 152.31 | 162.58 | 6.74% | -1.12(-1.21 - -1.04) | 10286.67 | 6956.8 | -32.37% | -2.75(-2.82 - -2.68) |
| Honduras | COCLD | 1361.84 | 3080.38 | 126.19% | -0.43(-0.53 - -0.32) | 1124.6 | 2952.23 | 162.51% | 0.39(0.24-0.54) | 45816.95 | 84990.77 | 85.50% | -0.45(-0.55 - -0.36) |
| Honduras | GABD | 28881.33 | 87404.4 | 202.63% | 0.54(0.49-0.59) | 135.06 | 444.71 | 229.27% | 1.04(0.81-1.28) | 8901.87 | 20892.22 | 134.69% | -0.17(-0.26 - -0.08) |
| Honduras | IFAH | 9861.83 | 22481.61 | 127.97% | -0.07(-0.15-0.02) | 108.4 | 189.5 | 74.82% | 0.2(0.02-0.39) | 8451.8 | 9852.26 | 16.57% | -1.06(-1.19 - -0.93) |
| Honduras | IFBD | 72.89 | 182.18 | 149.94% | -0.35(-0.5 - -0.21) | 21.65 | 49.57 | 128.96% | 0.87(0.73-1.02) | 1240.48 | 1815.7 | 46.37% | -0.38(-0.43 - -0.32) |
| Honduras | PACA | 1316.48 | 3274.12 | 148.70% | -0.05(-0.07 - -0.03) | 96.99 | 253.35 | 161.21% | 0.49(0.34-0.64) | 4180.27 | 7963.63 | 90.51% | -0.53(-0.64 - -0.43) |
| Honduras | PIIO | 4114.87 | 7939.77 | 92.95% | 0.1(-0.02-0.21) | 418.4 | 277.35 | -33.71% | -1.96(-2.04 - -1.88) | 35025.65 | 18480.35 | -47.24% | -3.19(-3.25 - -3.13) |
| Honduras | UDSD | 203678.42 | 539079.89 | 164.67% | -0.027(-0.033 - -0.02) | 362.73 | 755.18 | 108.19% | -0.46(-0.7 - -0.22) | 17248.6 | 29105.12 | 68.74% | -0.99(-1.1 - -0.89) |
| Honduras | VAID | 318.35 | 918.32 | 188.46% | 0.49(0.43-0.54) | 63.31 | 237.02 | 274.38% | 1.55(1.31-1.8) | 1706.62 | 4510.38 | 164.29% | 0.91(0.75-1.08) |
| Hungary | Digestive diseases | 758481.41 | 816240.7 | 7.62% | 0.01(0-0.02) | 9161.76 | 6413.39 | -30.00% | -3.08(-3.4 - -2.77) | 315344.84 | 194782.5 | -38.23% | -3.26(-3.6 - -2.92) |
| Hungary | APED | 21349.72 | 17933.25 | -16.00% | 0.07(0.03-0.12) | 67.01 | 39.66 | -40.81% | -2.59(-2.87 - -2.32) | 1934.35 | 1026.31 | -46.94% | -2.29(-2.51 - -2.07) |
| Hungary | COCLD | 6257.71 | 3700.96 | -40.86% | -2.38(-2.63 - -2.13) | 5950.8 | 3510.54 | -41.01% | -3.81(-4.28 - -3.34) | 203835.19 | 101513 | -50.20% | -4.45(-4.98 - -3.93) |
| Hungary | GABD | 151224.62 | 167451.36 | 10.73% | -0.09(-0.13 - -0.05) | 496.99 | 407.35 | -18.04% | -1.84(-2.26 - -1.41) | 23598.86 | 19619.03 | -16.86% | -1.17(-1.35 - -0.98) |
| Hungary | IFAH | 17466.39 | 21689.62 | 24.18% | 0.73(0.7-0.77) | 152.99 | 114.07 | -25.44% | -2.25(-2.58 - -1.91) | 6358.71 | 5446.87 | -14.34% | -0.75(-0.92 - -0.58) |
| Hungary | IFBD | 1315.89 | 2268.31 | 72.38% | 2.22(1.94-2.51) | 84.22 | 100.44 | 19.26% | -0.62(-1.04 - -0.2) | 5849.49 | 8339.03 | 42.56% | 1.15(0.85-1.46) |
| Hungary | PACA | 6350.92 | 6337.42 | -0.21% | -0.43(-0.47 - -0.4) | 568.05 | 378.52 | -33.37% | -2.6(-2.8 - -2.39) | 18772.94 | 10049.35 | -46.47% | -3.16(-3.4 - -2.92) |
| Hungary | PIIO | 13092.71 | 16244.92 | 24.08% | 0.05(-0.04-0.14) | 216.55 | 262.46 | 21.20% | -0.84(-0.92 - -0.77) | 5173.12 | 4774.49 | -7.71% | -1.53(-1.58 - -1.47) |
| Hungary | UDSD | 538728.22 | 577076.8 | 7.12% | 0.02(0.016-0.024) | 966.17 | 708.82 | -26.64% | -2.58(-2.75 - -2.42) | 35044 | 27570.44 | -21.33% | -1.54(-1.64 - -1.44) |
| Hungary | VAID | 2695.24 | 3538.08 | 31.27% | 0.13(0.04-0.23) | 471.65 | 575.42 | 22.00% | -0.93(-1.13 - -0.73) | 9655.86 | 9707.86 | 0.54% | -1.34(-1.54 - -1.15) |
| Iceland | Digestive diseases | 9893.45 | 15838.06 | 60.09% | 0.19(0.14-0.23) | 46.88 | 62.34 | 32.98% | -1.74(-1.83 - -1.64) | 1296.07 | 1681.5 | 29.74% | -1.18(-1.24 - -1.13) |
| Iceland | APED | 701.02 | 962.08 | 37.24% | 0.5(0.43-0.57) | 0.44 | 0.26 | -40.91% | -4.24(-4.37 - -4.11) | 18.17 | 16.1 | -11.39% | -1.28(-1.41 - -1.15) |
| Iceland | COCLD | 23.26 | 33.53 | 44.15% | 0.3(0.25-0.36) | 12.69 | 16.91 | 33.25% | -1.45(-1.53 - -1.37) | 363.94 | 453.5 | 24.61% | -1.55(-1.65 - -1.44) |
| Iceland | GABD | 690.14 | 1143.59 | 65.70% | 0.94(0.53-1.36) | 4.13 | 5.57 | 34.87% | -1.5(-1.63 - -1.36) | 113.27 | 147.38 | 30.11% | -0.49(-0.71 - -0.26) |
| Iceland | IFAH | 227.71 | 395.61 | 73.73% | 0.3(0.25-0.34) | 1.31 | 1.27 | -3.05% | -3.14(-3.58 - -2.71) | 59.68 | 76.46 | 28.12% | -0.89(-1.05 - -0.73) |
| Iceland | IFBD | 29.57 | 64.68 | 118.74% | 0.84(0.54-1.14) | 1.97 | 4.1 | 108.12% | 0.14(-0.01-0.29) | 89.77 | 186.54 | 107.80% | 0.47(0.33-0.62) |
| Iceland | PACA | 53.93 | 98.36 | 82.38% | -0.06(-0.24-0.11) | 3.19 | 3.6 | 12.85% | -2.2(-2.32 - -2.08) | 82.01 | 86.14 | 5.04% | -2.04(-2.17 - -1.92) |
| Iceland | PIIO | 370.17 | 676.18 | 82.67% | 0.24(0.11-0.37) | 4.44 | 7.99 | 79.83% | -0.76(-0.91 - -0.6) | 77.25 | 114.29 | 47.94% | -0.91(-1.03 - -0.79) |
| Iceland | UDSD | 7701.95 | 12281.2 | 59.46% | 0.066(0.055-0.076) | 8.47 | 4.7 | -44.51% | -5.14(-5.36 - -4.92) | 316.96 | 336.84 | 6.27% | -1.63(-1.77 - -1.49) |
| Iceland | VAID | 95.71 | 182.83 | 91.02% | 0.47(0.32-0.62) | 5.39 | 7.06 | 30.98% | -2.2(-2.47 - -1.92) | 87.7 | 100.56 | 14.66% | -2.34(-2.59 - -2.1) |
| India | Digestive diseases | 43379092.81 | 90709157.74 | 109.11% | 0.18(0.14-0.22) | 315247.78 | 464913.93 | 47.48% | -1.9(-2.08 - -1.73) | 13738700.57 | 18467788.93 | 34.42% | -1.6(-1.73 - -1.46) |
| India | APED | 693664.57 | 2262368.69 | 226.15% | 2.44(1.8-3.09) | 15824.52 | 11925.97 | -24.64% | -3.83(-4.06 - -3.61) | 783803.24 | 497437.9 | -36.54% | -4.04(-4.27 - -3.81) |
| India | COCLD | 138576.4 | 326903.94 | 135.90% | 1.51(1.4-1.61) | 144247.27 | 270036.76 | 87.20% | -0.65(-0.85 - -0.44) | 5851340.75 | 9635297.41 | 64.67% | -0.66(-0.88 - -0.45) |
| India | GABD | 1321987.66 | 5019382.75 | 279.68% | 3.07(2.58-3.56) | 5951.99 | 7703.27 | 29.42% | -2.52(-2.74 - -2.31) | 399252.91 | 733078.29 | 83.61% | -0.18(-0.55-0.2) |
| India | IFAH | 1709410.61 | 2449459.09 | 43.29% | -1.39(-1.55 - -1.23) | 9200.03 | 11355.2 | 23.43% | -3.3(-3.59 - -3) | 715662.22 | 751139.93 | 4.96% | -2.7(-2.9 - -2.49) |
| India | IFBD | 15360.33 | 31774.51 | 106.86% | 0.42(0.24-0.61) | 2770.18 | 4214.16 | 52.13% | -1.93(-2.11 - -1.74) | 127567.73 | 164416.35 | 28.89% | -1.73(-1.86 - -1.61) |
| India | PACA | 271026.67 | 618862.35 | 128.34% | 0.59(0.51-0.67) | 10535.71 | 20455.86 | 94.16% | -0.51(-0.67 - -0.35) | 418930 | 715968.58 | 70.90% | -0.58(-0.72 - -0.43) |
| India | PIIO | 446176.38 | 1115695.61 | 150.06% | 0.84(0.81-0.88) | 34579.5 | 52421.73 | 51.60% | -1.76(-1.94 - -1.58) | 1467483.5 | 1781453.61 | 21.40% | -1.73(-1.89 - -1.57) |
| India | UDSD | 38726597.04 | 78738926.49 | 103.32% | 0.051(0.033-0.068) | 86882.92 | 72608.2 | -16.43% | -4.31(-4.62 - -4) | 3804298.89 | 3842059.19 | 0.99% | -3.08(-3.24 - -2.91) |
| India | VAID | 56293.15 | 145784.32 | 158.97% | 1.1(0.98-1.22) | 3530.98 | 11373.37 | 222.10% | -0.04(-0.21-0.13) | 88436.63 | 232036.38 | 162.38% | -0.13(-0.26-0) |
| Indonesia | Digestive diseases | 4843081.36 | 8701982.33 | 79.68% | 0.06(0.03-0.08) | 84478.22 | 126347.5 | 49.56% | -0.54(-0.61 - -0.47) | 3542926.73 | 4036404.09 | 13.93% | -1.27(-1.34 - -1.2) |
| Indonesia | APED | 101300.03 | 248461.16 | 145.27% | 2.28(1.51-3.05) | 1663.39 | 1160.66 | -30.22% | -2.28(-2.42 - -2.15) | 97005.71 | 49347.18 | -49.13% | -3.17(-3.31 - -3.04) |
| Indonesia | COCLD | 47107.27 | 64125.63 | 36.13% | -1.27(-1.52 - -1.03) | 57982.69 | 88670.11 | 52.93% | -0.56(-0.66 - -0.46) | 2209728.32 | 2704508.62 | 22.39% | -1.24(-1.36 - -1.13) |
| Indonesia | GABD | 301822.42 | 669895.44 | 121.95% | 0.76(0.65-0.87) | 3595.95 | 5951.94 | 65.52% | -0.43(-0.52 - -0.33) | 140931.96 | 189771.13 | 34.65% | -1.07(-1.13 - -1.01) |
| Indonesia | IFAH | 343041.22 | 527664.21 | 53.82% | -0.82(-1.05 - -0.59) | 3563 | 5145.64 | 44.42% | -0.31(-0.42 - -0.2) | 239828.44 | 215391.53 | -10.19% | -1.59(-1.68 - -1.5) |
| Indonesia | IFBD | 681.12 | 1504.03 | 120.82% | 0.85(0.76-0.93) | 704.33 | 912.65 | 29.58% | -1.42(-1.56 - -1.28) | 26026.06 | 24942.78 | -4.16% | -2.04(-2.17 - -1.91) |
| Indonesia | PACA | 40776.47 | 72002.06 | 76.58% | -0.11(-0.12 - -0.1) | 2278.02 | 3439.86 | 51.00% | -0.66(-0.71 - -0.62) | 86087.79 | 106212.31 | 23.38% | -1.28(-1.33 - -1.23) |
| Indonesia | PIIO | 70544.35 | 144818.7 | 105.29% | 0.72(0.64-0.8) | 9260.21 | 13017.39 | 40.57% | -0.2(-0.26 - -0.14) | 466784.71 | 398472.63 | -14.63% | -1.19(-1.24 - -1.14) |
| Indonesia | UDSD | 3929285.31 | 6956812.02 | 77.05% | 0.024(0.006-0.043) | 2357.86 | 2105.06 | -10.72% | -2.32(-2.48 - -2.16) | 175766.29 | 218221.33 | 24.15% | -1.33(-1.38 - -1.28) |
| Indonesia | VAID | 8523.14 | 16699.06 | 95.93% | 0.04(-0.03-0.1) | 718.57 | 1878.46 | 161.42% | 0.74(0.67-0.81) | 15263.95 | 33089.51 | 116.78% | 0.27(0.19-0.34) |
| Iran (Islamic Republic of) | Digestive diseases | 2414372.5 | 5311051.92 | 119.98% | 0.12(0.09-0.15) | 6936.08 | 11482.01 | 65.54% | -1.53(-1.69 - -1.36) | 334644.4 | 438713.96 | 31.10% | -1.28(-1.39 - -1.17) |
| Iran (Islamic Republic of) | APED | 97503.21 | 212552.2 | 118.00% | 1.61(1.38-1.83) | 224.55 | 161.26 | -28.19% | -3.54(-3.67 - -3.41) | 12724.99 | 7535.53 | -40.78% | -2.95(-3.09 - -2.8) |
| Iran (Islamic Republic of) | COCLD | 6502.38 | 14345.98 | 120.63% | 0.34(0.19-0.49) | 3999.08 | 6831.73 | 70.83% | -1.39(-1.54 - -1.23) | 148681.58 | 184174.44 | 23.87% | -1.54(-1.71 - -1.38) |
| Iran (Islamic Republic of) | GABD | 151908.21 | 416857.72 | 174.41% | 0.74(0.32-1.17) | 223.79 | 785.39 | 250.95% | 0.48(0.16-0.8) | 18632.41 | 40971.61 | 119.89% | -0.08(-0.26-0.1) |
| Iran (Islamic Republic of) | IFAH | 45082.33 | 83709.93 | 85.68% | 0.57(0.52-0.62) | 63.69 | 102.41 | 60.79% | -1.02(-1.34 - -0.69) | 10949.84 | 14066.45 | 28.46% | -0.27(-0.43 - -0.11) |
| Iran (Islamic Republic of) | IFBD | 1347.43 | 3166.82 | 135.03% | 0.21(0.06-0.36) | 51.09 | 131.82 | 158.02% | -0.11(-0.64-0.42) | 4022.43 | 9481.08 | 135.71% | 0.12(-0.02-0.25) |
| Iran (Islamic Republic of) | PACA | 8893.76 | 19554.57 | 119.87% | 0.07(0.03-0.11) | 170.6 | 398.28 | 133.46% | -0.67(-0.92 - -0.43) | 5400.67 | 10144.38 | 87.84% | -0.76(-0.97 - -0.55) |
| Iran (Islamic Republic of) | PIIO | 46980.93 | 93329.08 | 98.65% | 0.4(0.36-0.45) | 567.13 | 743.73 | 31.14% | -1(-1.49 - -0.51) | 35779.26 | 21503.92 | -39.90% | -1.44(-1.84 - -1.03) |
| Iran (Islamic Republic of) | UDSD | 2052590.49 | 4459212.3 | 117.25% | -0.009(-0.063-0.045) | 1372.94 | 1485.76 | 8.22% | -3.41(-3.54 - -3.28) | 89343.76 | 129963.83 | 45.46% | -1.53(-1.65 - -1.41) |
| Iran (Islamic Republic of) | VAID | 3563.76 | 8323.34 | 133.56% | -0.19(-0.33 - -0.05) | 109.28 | 322.78 | 195.37% | -0.36(-0.7 - -0.01) | 2525.56 | 6273.55 | 148.40% | -0.34(-0.68 - -0.01) |
| Iraq | Digestive diseases | 695367.31 | 2147645.29 | 208.85% | 0.12(0.11-0.14) | 2278.12 | 3656 | 60.48% | -1.62(-1.75 - -1.5) | 110878.62 | 177244.95 | 59.85% | -1.57(-1.66 - -1.48) |
| Iraq | APED | 34868.71 | 131681.54 | 277.65% | 1.47(1.38-1.57) | 18.59 | 19.31 | 3.87% | -2.68(-2.86 - -2.49) | 1413.31 | 2378.99 | 68.33% | -1.48(-1.58 - -1.38) |
| Iraq | COCLD | 2161.65 | 6593.13 | 205.00% | 0.47(0.34-0.6) | 1490.05 | 2720.08 | 82.55% | -1.42(-1.54 - -1.29) | 54583.31 | 87671.27 | 60.62% | -1.73(-1.85 - -1.6) |
| Iraq | GABD | 22523.69 | 75270.94 | 234.19% | 0.42(0.36-0.47) | 73.7 | 118.94 | 61.38% | -1.79(-1.93 - -1.65) | 4863.67 | 10259.81 | 110.95% | -1.35(-1.41 - -1.29) |
| Iraq | IFAH | 9180.25 | 25811.12 | 181.16% | 1.25(1.09-1.41) | 41.69 | 28.8 | -30.92% | -2.35(-2.49 - -2.21) | 5291.03 | 6845.28 | 29.38% | -1.04(-1.08 - -1) |
| Iraq | IFBD | 239.84 | 983.98 | 310.27% | 1.33(1.19-1.46) | 33.67 | 51.3 | 52.36% | -1.58(-1.76 - -1.39) | 2135.49 | 3718.57 | 74.13% | -0.96(-1.14 - -0.78) |
| Iraq | PACA | 2804.65 | 7590.92 | 170.65% | -0.25(-0.26 - -0.25) | 41.52 | 98.7 | 137.72% | -0.47(-0.65 - -0.3) | 1467.28 | 3519.41 | 139.86% | -0.57(-0.72 - -0.43) |
| Iraq | PIIO | 10800.9 | 32972.42 | 205.27% | 1.27(1.04-1.5) | 259.69 | 244.16 | -5.98% | -1.7(-1.83 - -1.57) | 17342.14 | 10506.88 | -39.41% | -2.82(-3.01 - -2.63) |
| Iraq | UDSD | 611904.61 | 1863458.45 | 204.53% | 0.018(0.013-0.023) | 242.86 | 223.37 | -8.03% | -3.62(-3.77 - -3.47) | 20695.02 | 47191.07 | 128.03% | -1.14(-1.2 - -1.07) |
| Iraq | VAID | 883 | 3282.79 | 271.78% | 1.52(1.29-1.75) | 31.47 | 75.42 | 139.66% | -0.27(-0.44 - -0.1) | 803.33 | 2000.5 | 149.03% | -0.19(-0.35 - -0.03) |
| Ireland | Digestive diseases | 162014.73 | 263588.22 | 62.69% | 0.08(0.08-0.09) | 838.14 | 1169.66 | 39.55% | -1.27(-1.52 - -1.03) | 22015.91 | 31003.46 | 40.82% | -0.76(-0.99 - -0.52) |
| Ireland | APED | 9015.97 | 11387.93 | 26.31% | 0.26(0.22-0.31) | 5.14 | 4.47 | -13.04% | -3.4(-3.9 - -2.89) | 226.92 | 214.59 | -5.43% | -1.49(-1.7 - -1.28) |
| Ireland | COCLD | 391.54 | 826.01 | 110.96% | 1.12(0.89-1.35) | 194.94 | 388.48 | 99.28% | 0.5(0.03-0.98) | 5287 | 10587.31 | 100.25% | 0.72(0.18-1.27) |
| Ireland | GABD | 14667.69 | 26628.31 | 81.54% | 0.19(0.16-0.22) | 52.86 | 88.05 | 66.57% | -0.56(-0.65 - -0.47) | 2034.68 | 2957.75 | 45.37% | -0.71(-0.75 - -0.66) |
| Ireland | IFAH | 3755.08 | 7047.98 | 87.69% | 0.29(0.09-0.49) | 24.39 | 34.89 | 43.05% | -1.8(-2.28 - -1.33) | 1127.68 | 1533.69 | 36.00% | -0.97(-1.2 - -0.74) |
| Ireland | IFBD | 350.97 | 436.82 | 24.46% | -0.76(-0.97 - -0.55) | 33.05 | 50.76 | 53.59% | -1.61(-2.56 - -0.66) | 1106.08 | 1623.27 | 46.76% | -0.8(-1.3 - -0.3) |
| Ireland | PACA | 719.17 | 1160.2 | 61.32% | -0.11(-0.14 - -0.07) | 30.32 | 48.23 | 59.07% | -0.82(-1.17 - -0.46) | 773.58 | 1148.2 | 48.43% | -0.84(-1.22 - -0.46) |
| Ireland | PIIO | 4074.45 | 8932.23 | 119.23% | 1.08(0.99-1.16) | 67.83 | 124.37 | 83.36% | -0.65(-0.77 - -0.52) | 1195.17 | 1847.54 | 54.58% | -0.77(-0.9 - -0.63) |
| Ireland | UDSD | 128297.87 | 205401.01 | 60.10% | 0.008(0.005-0.011) | 252.03 | 110.71 | -56.07% | -6.12(-6.63 - -5.61) | 7128.81 | 6280.01 | -11.91% | -2.51(-2.7 - -2.32) |
| Ireland | VAID | 741.98 | 1767.73 | 138.24% | 1.22(1.16-1.28) | 74.06 | 136.56 | 84.39% | 0.33(0.1-0.56) | 1325.02 | 2143.63 | 61.78% | -0.01(-0.21-0.19) |
| Israel | Digestive diseases | 213844.16 | 447661.18 | 109.34% | 0.05(0.03-0.07) | 911.68 | 2067.22 | 126.75% | -1.14(-1.39 - -0.88) | 25913.2 | 48177.67 | 85.92% | -1.07(-1.24 - -0.9) |
| Israel | APED | 12077.42 | 22331.73 | 84.90% | 0.26(0.2-0.32) | 9.33 | 10.12 | 8.47% | -2.95(-3.54 - -2.35) | 347.7 | 425.52 | 22.38% | -1.4(-1.73 - -1.06) |
| Israel | COCLD | 826.59 | 1588.78 | 92.21% | -0.56(-0.74 - -0.38) | 399.13 | 755.95 | 89.40% | -1.54(-1.89 - -1.18) | 9718.68 | 15750.31 | 62.06% | -1.74(-2.06 - -1.42) |
| Israel | GABD | 18432.97 | 43699.11 | 137.07% | 0.27(0.22-0.32) | 72.63 | 287.54 | 295.90% | 0.64(0.23-1.05) | 2629.7 | 6389.99 | 142.99% | -0.08(-0.25-0.09) |
| Israel | IFAH | 4409.77 | 10149.3 | 130.15% | 0.25(0.2-0.3) | 36.13 | 52.19 | 44.45% | -3.51(-3.77 - -3.25) | 1544.54 | 2380.61 | 54.13% | -1.52(-1.66 - -1.39) |
| Israel | IFBD | 297.83 | 651.75 | 118.83% | 0.33(0.14-0.51) | 21.77 | 45.94 | 111.02% | -1.01(-1.36 - -0.66) | 999.71 | 1850.85 | 85.14% | -0.64(-0.76 - -0.54) |
| Israel | PACA | 958.17 | 2003.17 | 109.06% | -0.12(-0.14 - -0.09) | 45.43 | 109.38 | 140.77% | -0.46(-0.81 - -0.12) | 974.82 | 1974.22 | 102.52% | -0.63(-0.91 - -0.34) |
| Israel | PIIO | 4700.06 | 12856.74 | 173.54% | 0.83(0.75-0.91) | 78.39 | 277.44 | 253.91% | 0.66(0.51-0.82) | 1567.49 | 3956.79 | 152.43% | 0.2(0.08-0.31) |
| Israel | UDSD | 171226.12 | 351981.33 | 105.57% | -0.019(-0.048-0.01) | 118.89 | 81.87 | -31.14% | -6.43(-6.98 - -5.87) | 5805.38 | 9040.27 | 55.72% | -1.44(-1.64 - -1.24) |
| Israel | VAID | 915.23 | 2399.27 | 162.15% | 0.42(0.36-0.48) | 97.3 | 242.26 | 148.98% | -0.77(-0.92 - -0.62) | 1617.14 | 3353.27 | 107.36% | -1(-1.15 - -0.85) |
| Italy | Digestive diseases | 4497510.61 | 5563658.85 | 23.71% | 0.14(0.08-0.19) | 29518.18 | 28523.03 | -3.37% | -2.45(-2.58 - -2.33) | 877050.39 | 700554.54 | -20.12% | -2.08(-2.25 - -1.91) |
| Italy | APED | 113265.45 | 93251.36 | -17.67% | -0.12(-0.3-0.06) | 106.73 | 96.6 | -9.49% | -2.42(-2.67 - -2.16) | 3760.13 | 2624.14 | -30.21% | -1.48(-1.65 - -1.31) |
| Italy | COCLD | 30032.31 | 24482.73 | -18.48% | -1.35(-1.47 - -1.23) | 18129.39 | 12450.48 | -31.32% | -3.31(-3.45 - -3.18) | 450644.32 | 247093.49 | -45.17% | -3.61(-3.77 - -3.46) |
| Italy | GABD | 1098118.74 | 1590986.28 | 44.88% | 0.69(0.51-0.86) | 1594.56 | 2609.03 | 63.62% | -0.62(-0.87 - -0.37) | 127728.95 | 134030.32 | 4.93% | -0.92(-1.32 - -0.52) |
| Italy | IFAH | 183915.57 | 287753.18 | 56.46% | 1.04(0.6-1.49) | 669.75 | 664.07 | -0.85% | -2.85(-3.02 - -2.69) | 36954.29 | 41095.03 | 11.21% | -0.53(-0.68 - -0.38) |
| Italy | IFBD | 12109.39 | 12194.2 | 0.70% | 0.33(0.17-0.49) | 350.7 | 1305.42 | 272.23% | 2.55(2.03-3.07) | 33890.37 | 44426.69 | 31.09% | 0.17(0-0.34) |
| Italy | PACA | 20578.97 | 27615.22 | 34.19% | -0.26(-0.32 - -0.21) | 984.45 | 1304.93 | 32.55% | -1.41(-1.51 - -1.3) | 23580.59 | 22720.29 | -3.65% | -1.79(-1.87 - -1.71) |
| Italy | PIIO | 150904.06 | 214754.64 | 42.31% | -0.27(-0.35 - -0.2) | 2070.57 | 4012.71 | 93.80% | -0.2(-0.34 - -0.06) | 33588.63 | 49898.33 | 48.56% | -0.55(-0.7 - -0.4) |
| Italy | UDSD | 2863533 | 3287324.07 | 14.80% | -0.071(-0.1 - -0.041) | 3009.97 | 1204.95 | -59.97% | -6.66(-7.13 - -6.19) | 118887.18 | 91456.09 | -23.07% | -1.7(-1.82 - -1.58) |
| Italy | VAID | 25053.12 | 25297.17 | 0.97% | -2.08(-2.53 - -1.62) | 1637.15 | 2353.01 | 43.73% | -1.37(-1.47 - -1.26) | 27008.86 | 29999.47 | 11.07% | -1.78(-1.94 - -1.63) |
| Jamaica | Digestive diseases | 148071.82 | 224462.22 | 51.59% | 0.09(0.08-0.09) | 393.96 | 535.72 | 35.99% | -0.85(-1.17 - -0.53) | 17086.89 | 20472.82 | 19.82% | -0.86(-1.1 - -0.61) |
| Jamaica | APED | 5885.6 | 8539.01 | 45.08% | 0.92(0.86-0.98) | 11.68 | 10.77 | -7.79% | -1.44(-1.97 - -0.92) | 665.9 | 465.23 | -30.14% | -1.85(-2.36 - -1.33) |
| Jamaica | COCLD | 196.23 | 246.16 | 25.44% | -0.89(-0.99 - -0.78) | 155.61 | 185.13 | 18.97% | -1.49(-1.85 - -1.13) | 4874.36 | 5080.81 | 4.24% | -1.85(-2.25 - -1.45) |
| Jamaica | GABD | 5943.19 | 8841.74 | 48.77% | 0.09(0.02-0.16) | 18.07 | 23.49 | 29.99% | -0.74(-1.27 - -0.22) | 1074.59 | 1396.86 | 29.99% | -0.55(-0.82 - -0.27) |
| Jamaica | IFAH | 4345.58 | 6851.75 | 57.67% | 0.87(0.8-0.95) | 10.39 | 17.73 | 70.64% | 1.1(0.61-1.6) | 1503.03 | 1948.13 | 29.61% | 0.44(0.28-0.6) |
| Jamaica | IFBD | 38.29 | 75.62 | 97.49% | 0.72(0.54-0.9) | 5.92 | 7.21 | 21.79% | -1.45(-1.78 - -1.11) | 304.34 | 342.91 | 12.67% | -1.28(-1.5 - -1.06) |
| Jamaica | PACA | 536.13 | 781.24 | 45.72% | -0.13(-0.15 - -0.1) | 11.02 | 17.55 | 59.26% | -0.36(-0.63 - -0.09) | 359.75 | 543.59 | 51.10% | -0.43(-0.74 - -0.11) |
| Jamaica | PIIO | 2256.32 | 3100.75 | 37.42% | 0.37(0.24-0.51) | 54.68 | 70.17 | 28.33% | -0.69(-0.95 - -0.43) | 2108.85 | 1643.57 | -22.06% | -1.42(-1.78 - -1.06) |
| Jamaica | UDSD | 128704.47 | 195767.3 | 52.11% | 0.025(0.022-0.027) | 97.58 | 113.92 | 16.75% | -1.62(-2.04 - -1.2) | 5292.99 | 7094.55 | 34.04% | -0.75(-0.95 - -0.55) |
| Jamaica | VAID | 166 | 258.63 | 55.80% | 0.24(0.19-0.29) | 12.5 | 18.55 | 48.40% | -0.53(-0.92 - -0.13) | 259.61 | 352.71 | 35.86% | -0.66(-1.08 - -0.24) |
| Japan | Digestive diseases | 7372877.37 | 9749034.88 | 32.23% | 0.31(0.22-0.39) | 41722.08 | 60801.06 | 45.73% | -2(-2.16 - -1.84) | 1272592.08 | 1302463.24 | 2.35% | -1.37(-1.46 - -1.29) |
| Japan | APED | 547093.96 | 381573.57 | -30.25% | -0.22(-0.34 - -0.1) | 130.2 | 202.13 | 55.25% | -1.74(-1.84 - -1.64) | 9564.52 | 7511.63 | -21.46% | -0.57(-0.67 - -0.48) |
| Japan | COCLD | 46733.96 | 33114.85 | -29.14% | -0.32(-0.49 - -0.16) | 25310.23 | 26248.36 | 3.71% | -2.4(-2.55 - -2.26) | 663242.82 | 489987.64 | -26.12% | -2.52(-2.63 - -2.42) |
| Japan | GABD | 1993171.79 | 3393914.75 | 70.28% | 1.05(0.87-1.23) | 2813.63 | 8584.94 | 205.12% | -0.54(-0.75 - -0.33) | 164235.13 | 243455.91 | 48.24% | -0.41(-0.49 - -0.32) |
| Japan | IFAH | 361710.46 | 450865.67 | 24.65% | 0.27(0.23-0.3) | 483.07 | 929.18 | 92.35% | -1.81(-2.3 - -1.32) | 58350.81 | 61598.07 | 5.57% | -0.04(-0.11-0.03) |
| Japan | IFBD | 16382.81 | 22435.8 | 36.95% | 1.32(1.02-1.61) | 366.91 | 452.47 | 23.32% | -2.39(-2.83 - -1.95) | 41769.34 | 67423.73 | 61.42% | 1.21(0.88-1.54) |
| Japan | PACA | 51754.79 | 60692.64 | 17.27% | -0.1(-0.2-0) | 1272.4 | 1655.06 | 30.07% | -2.46(-2.58 - -2.34) | 36231.62 | 33113.99 | -8.60% | -2.04(-2.15 - -1.93) |
| Japan | PIIO | 341119.26 | 504985.27 | 48.04% | 0(-0.02-0.03) | 3284.33 | 10083.04 | 207.00% | -0.29(-0.34 - -0.24) | 62338.15 | 122741.58 | 96.90% | -0.51(-0.56 - -0.45) |
| Japan | UDSD | 3947887.41 | 4792577.59 | 21.40% | 0.02(-0.029-0.069) | 5843.12 | 4757.1 | -18.59% | -4.85(-5.11 - -4.59) | 186750.5 | 167592.31 | -10.26% | -1.61(-1.76 - -1.47) |
| Japan | VAID | 67022.93 | 108874.75 | 62.44% | 0.17(0.06-0.29) | 1133.92 | 3709.83 | 227.17% | 0.34(0.18-0.5) | 21963.32 | 46929.71 | 113.67% | -0.1(-0.22-0.02) |
| Jordan | Digestive diseases | 148645.84 | 609039.78 | 309.73% | 0.13(0.13-0.14) | 459.75 | 1102.21 | 139.74% | -2.65(-2.89 - -2.41) | 18311.9 | 47550.93 | 159.67% | -2.14(-2.34 - -1.95) |
| Jordan | APED | 8214.64 | 36686.49 | 346.60% | 1.42(1.37-1.48) | 6.03 | 9.5 | 57.55% | -3.96(-4.3 - -3.61) | 334.61 | 744.57 | 122.52% | -2.54(-2.82 - -2.25) |
| Jordan | COCLD | 424.55 | 1763.46 | 315.37% | 0.12(0.05-0.18) | 247.11 | 644.07 | 160.64% | -2.3(-2.53 - -2.08) | 7893.29 | 18722.41 | 137.19% | -2.5(-2.75 - -2.24) |
| Jordan | GABD | 5319.65 | 24203.59 | 354.98% | 0.12(0.08-0.17) | 28.9 | 82.07 | 183.98% | -1.88(-2 - -1.76) | 1243.7 | 3671.71 | 195.22% | -1.79(-1.88 - -1.7) |
| Jordan | IFAH | 1934.96 | 7548.04 | 290.09% | 1.08(1.01-1.16) | 2.92 | 6.9 | 136.30% | -2.12(-2.26 - -1.99) | 601.57 | 1705.35 | 183.48% | -0.45(-0.5 - -0.4) |
| Jordan | IFBD | 129.92 | 778.63 | 499.31% | 1.49(1.33-1.66) | 5.72 | 13.47 | 135.49% | -2.62(-2.98 - -2.26) | 451.96 | 2154.97 | 376.81% | 0.46(0.34-0.58) |
| Jordan | PACA | 877.54 | 3806.55 | 333.78% | 0.2(0.09-0.3) | 19.93 | 47.36 | 137.63% | -3.09(-3.44 - -2.73) | 601.44 | 1456.11 | 142.10% | -2.96(-3.3 - -2.61) |
| Jordan | PIIO | 2542.68 | 12214.43 | 380.38% | 1.65(1.51-1.79) | 48.98 | 132.43 | 170.34% | -1.63(-1.77 - -1.5) | 1855.2 | 3631.41 | 95.74% | -1.79(-1.95 - -1.63) |
| Jordan | UDSD | 129048.15 | 520973.33 | 303.70% | 0.024(0.021-0.027) | 63.36 | 78.85 | 24.45% | -5.02(-5.44 - -4.59) | 4251.28 | 13073 | 207.51% | -1.82(-1.94 - -1.7) |
| Jordan | VAID | 153.75 | 1065.26 | 592.85% | 1.49(1.4-1.58) | 16.51 | 45.79 | 177.35% | -2.5(-2.85 - -2.15) | 400.64 | 1072.31 | 167.65% | -2.58(-2.96 - -2.21) |
| Kazakhstan | Digestive diseases | 827777.07 | 1043784.73 | 26.09% | 0.05(0.04-0.06) | 4435.77 | 8896.38 | 100.56% | 1.54(1.04-2.04) | 176565.92 | 326980.87 | 85.19% | 1.31(0.71-1.91) |
| Kazakhstan | APED | 42727.5 | 43912.06 | 2.77% | 0.08(-0.03-0.2) | 84.92 | 23.21 | -72.67% | -6.39(-6.83 - -5.94) | 4661.82 | 1527.84 | -67.23% | -5.4(-5.81 - -4.98) |
| Kazakhstan | COCLD | 3469.89 | 12698.31 | 265.96% | 4.26(4.05-4.48) | 2184.32 | 6704.96 | 206.96% | 3.39(2.76-4.02) | 67654.82 | 217820 | 221.96% | 3.49(2.62-4.37) |
| Kazakhstan | GABD | 66512.74 | 85546.5 | 28.62% | -0.01(-0.08-0.06) | 239.62 | 161.34 | -32.67% | -2.89(-3.42 - -2.37) | 13939.85 | 12278.45 | -11.92% | -1.91(-2.28 - -1.54) |
| Kazakhstan | IFAH | 25856.17 | 32373.58 | 25.21% | 0.24(0.11-0.37) | 144.8 | 60.63 | -58.13% | -4.8(-5.56 - -4.04) | 11083.39 | 8332.58 | -24.82% | -2.22(-2.61 - -1.82) |
| Kazakhstan | IFBD | 922.22 | 1388.99 | 50.61% | 0.43(0.3-0.56) | 75.83 | 81.53 | 7.52% | -1.26(-1.68 - -0.85) | 4457.76 | 5172.4 | 16.03% | -0.74(-1.02 - -0.45) |
| Kazakhstan | PACA | 6412.54 | 8054.02 | 25.60% | -0.15(-0.17 - -0.12) | 829.96 | 881.12 | 6.16% | -1.47(-2.09 - -0.85) | 28287.76 | 31211.91 | 10.34% | -1.34(-2.01 - -0.67) |
| Kazakhstan | PIIO | 10896.08 | 16529.85 | 51.70% | 1.03(0.78-1.28) | 251.12 | 187.12 | -25.49% | -2.16(-2.42 - -2.42) | 12598.25 | 8199.56 | -34.92% | -2.44(-2.73 - -2.14) |
| Kazakhstan | UDSD | 668433.1 | 839377.34 | 25.57% | -0.007(-0.01 - -0.003) | 395.82 | 467.15 | 18.02% | -0.81(-1.32 - -0.3) | 27801.17 | 32878.76 | 18.26% | -0.71(-1.04 - -0.37) |
| Kazakhstan | VAID | 2546.82 | 3904.09 | 53.29% | 1.02(0.89-1.15) | 197.31 | 224.68 | 13.87% | -0.93(-1.32 - -0.53) | 4187.1 | 5091.65 | 21.60% | -0.84(-1.27 - -0.41) |
| Kenya | Digestive diseases | 791579.06 | 2132667.04 | 169.42% | 0.06(0.04-0.09) | 8753.51 | 20749.4 | 137.04% | -0.04(-0.18-0.1) | 319398.05 | 735858.93 | 130.39% | -0.05(-0.22-0.12) |
| Kenya | APED | 11826.27 | 39716.97 | 235.84% | 1.56(0.69-2.45) | 126.04 | 227.09 | 80.17% | -0.3(-0.45 - -0.15) | 6533.91 | 10183.75 | 55.86% | -0.3(-0.51 - -0.08) |
| Kenya | COCLD | 5233.59 | 14848.37 | 183.71% | -0.06(-0.16-0.05) | 5429.56 | 13094.85 | 141.18% | -0.1(-0.3-0.11) | 174630.06 | 424115.64 | 142.87% | -0.08(-0.32-0.16) |
| Kenya | GABD | 8721.4 | 23990.85 | 175.08% | 0.42(0.37-0.47) | 346.05 | 942.05 | 172.23% | 0.59(0.49-0.68) | 9457.89 | 24924.32 | 163.53% | 0.47(0.36-0.58) |
| Kenya | IFAH | 22459.22 | 59461.01 | 164.75% | 0.82(0.64-0.99) | 199.22 | 397.21 | 99.38% | 0.21(0.12-0.3) | 13227.62 | 22682.79 | 71.48% | 0.12(-0.01-0.24) |
| Kenya | IFBD | 123.74 | 426.85 | 244.96% | 0.67(0.53-0.82) | 73.79 | 180.07 | 144.03% | 0.31(0.22-0.4) | 3011.08 | 6452.81 | 114.30% | 0.26(0.16-0.36) |
| Kenya | PACA | 3378.2 | 8680.43 | 156.95% | -0.02(-0.04-0.01) | 154.94 | 432.37 | 179.06% | 0.23(0.17-0.29) | 5462.92 | 15564.04 | 184.90% | 0.33(0.23-0.43) |
| Kenya | PIIO | 11716.38 | 31274.09 | 166.93% | 0.48(0.44-0.51) | 1017.44 | 2432.63 | 139.09% | 0.16(0.06-0.26) | 43652.31 | 91769.74 | 110.23% | 0.24(0.12-0.36) |
| Kenya | UDSD | 726704.89 | 1950703.45 | 168.43% | 0.021(0.004-0.039) | 859.69 | 1634.86 | 90.17% | -0.9(-1.04 - -0.75) | 46951.64 | 102945.48 | 119.26% | -0.53(-0.65 - -0.42) |
| Kenya | VAID | 1415.39 | 3565.01 | 151.87% | 0.1(-0.03-0.22) | 120.57 | 380.78 | 215.82% | 1.05(0.97-1.13) | 2728.38 | 8410.04 | 208.24% | 0.99(0.92-1.06) |
| Kiribati | Digestive diseases | 1828.41 | 3169.24 | 73.33% | -0.02(-0.03 - -0.01) | 45.54 | 52.31 | 14.87% | -1.6(-1.68 - -1.51) | 1753.13 | 2030.22 | 15.81% | -1.66(-1.75 - -1.58) |
| Kiribati | APED | 80.98 | 136.49 | 68.55% | -0.04(-0.12-0.04) | 0.67 | 0.73 | 8.96% | -1.66(-1.75 - -1.57) | 30.92 | 33.89 | 9.61% | -1.66(-1.73 - -1.59) |
| Kiribati | COCLD | 13.6 | 20.01 | 47.13% | -0.62(-0.76 - -0.49) | 23.64 | 27.06 | 14.47% | -1.78(-1.92 - -1.64) | 913.76 | 1047.69 | 14.66% | -1.81(-1.95 - -1.68) |
| Kiribati | GABD | 86.89 | 164.14 | 88.91% | 0.33(0.31-0.35) | 2.27 | 3.28 | 44.49% | -0.6(-0.66 - -0.55) | 87.26 | 127.61 | 46.24% | -0.73(-0.78 - -0.69) |
| Kiribati | IFAH | 42.8 | 92.74 | 116.68% | 0.75(0.66-0.84) | 0.56 | 0.65 | 16.07% | -1.25(-1.41 - -1.08) | 31.29 | 45.45 | 45.25% | -0.67(-0.83 - -0.51) |
| Kiribati | IFBD | 0.29 | 0.6 | 106.90% | 0.56(0.48-0.64) | 0.7 | 0.73 | 4.29% | -2.13(-2.29 - -1.98) | 28.43 | 29.59 | 4.08% | -2.07(-2.2 - -1.93) |
| Kiribati | PACA | 14.51 | 25.42 | 75.19% | -0.03(-0.04 - -0.03) | 1.66 | 2.34 | 40.96% | -1(-1.07 - -0.94) | 70.32 | 99.7 | 41.78% | -0.99(-1.06 - -0.92) |
| Kiribati | PIIO | 24.25 | 40.91 | 68.70% | 0.18(0.13-0.24) | 1.65 | 2.03 | 23.00% | -0.84(-0.9 - -0.78) | 61.9 | 67.41 | 8.89% | -1.11(-1.17 - -1.05) |
| Kiribati | UDSD | 1562 | 2682.62 | 71.74% | -0.062(-0.071 - -0.053) | 12.47 | 12.6 | 1.04% | -1.91(-2.01 - -1.8) | 463.47 | 481.41 | 3.87% | -1.98(-2.08 - -1.88) |
| Kiribati | VAID | 3.09 | 6.31 | 104.21% | 0.65(0.62-0.68) | 0.76 | 1.25 | 64.47% | -0.31(-0.4 - -0.22) | 24.45 | 39.58 | 61.88% | -0.38(-0.44 - -0.33) |
| Kuwait | Digestive diseases | 87641.07 | 287739.88 | 228.32% | 0.16(0.14-0.18) | 106.62 | 337.66 | 216.68% | -0.19(-0.64-0.27) | 6214.73 | 17317.52 | 178.65% | -0.43(-0.76 - -0.09) |
| Kuwait | APED | 3982.33 | 13613.67 | 241.85% | 1.26(1.22-1.31) | 1.76 | 2.58 | 46.59% | -2.38(-2.83 - -1.94) | 116.11 | 225.52 | 94.23% | -0.94(-1.23 - -0.65) |
| Kuwait | COCLD | 174.84 | 755.74 | 332.25% | 1.27(1.11-1.43) | 60.92 | 203.88 | 234.67% | 0.07(-0.62-0.76) | 2212.23 | 6083.02 | 174.97% | -0.5(-1.2-0.2) |
| Kuwait | GABD | 2739.5 | 11735.78 | 328.39% | 0.58(0.48-0.69) | 4.95 | 17.26 | 248.69% | -0.19(-0.45-0.08) | 370.96 | 1200.58 | 223.64% | -0.36(-0.5 - -0.21) |
| Kuwait | IFAH | 1183.78 | 3762.67 | 217.85% | 0.22(0.12-0.32) | 1.68 | 3.63 | 116.07% | -1.5(-1.86 - -1.14) | 313.41 | 759.15 | 142.22% | -0.63(-0.73 - -0.53) |
| Kuwait | IFBD | 61.16 | 195.23 | 219.21% | 0.4(0.3-0.51) | 1.36 | 4.02 | 195.59% | -0.66(-1.06 - -0.25) | 187.69 | 558.35 | 197.49% | -0.33(-0.5 - -0.15) |
| Kuwait | PACA | 364.97 | 1205.69 | 230.35% | -0.02(-0.04-0.01) | 7.04 | 22.44 | 218.75% | -0.09(-0.31-0.13) | 283.31 | 794.65 | 180.49% | -0.39(-0.58 - -0.19) |
| Kuwait | PIIO | 1978.48 | 6933.63 | 250.45% | 0.81(0.67-0.95) | 7.74 | 27.14 | 250.87% | 0.84(0.63-1.06) | 416.83 | 837.43 | 100.91% | 0.22(0.03-0.4) |
| Kuwait | UDSD | 77013.75 | 248841.67 | 223.11% | 0.059(0.048-0.07) | 13.73 | 23.81 | 73.42% | -2.57(-3.02 - -2.13) | 2048.01 | 6031.95 | 194.53% | -0.62(-0.72 - -0.52) |
| Kuwait | VAID | 142.28 | 695.8 | 389.04% | 1.5(1.41-1.59) | 5.24 | 18.54 | 253.82% | -0.18(-0.5-0.13) | 144.98 | 405.89 | 179.96% | -0.38(-0.68 - -0.08) |
| Kyrgyzstan | Digestive diseases | 197329.86 | 325153.95 | 64.78% | 0.06(0.04-0.08) | 1379.73 | 2482.48 | 79.92% | 0.25(-0.32-0.82) | 55476.86 | 101282.9 | 82.57% | 0.24(-0.29-0.77) |
| Kyrgyzstan | APED | 10604.78 | 16572.78 | 56.28% | 0.34(0.21-0.47) | 16.83 | 9.08 | -46.05% | -3.7(-4.09 - -3.31) | 951.17 | 585.46 | -38.45% | -3.28(-3.69 - -2.87) |
| Kyrgyzstan | COCLD | 1311.31 | 3182.65 | 142.71% | 1.27(1-1.53) | 1024.41 | 2143.06 | 109.20% | 0.73(0.07-1.41) | 34946.07 | 77751.71 | 122.49% | 0.85(0.16-1.55) |
| Kyrgyzstan | GABD | 14712.3 | 23736.07 | 61.33% | -0.07(-0.13 - -0.02) | 45.89 | 35.27 | -23.14% | -2.28(-2.57 - -2) | 3095.31 | 3379.47 | 9.18% | -1.61(-1.73 - -1.48) |
| Kyrgyzstan | IFAH | 5070.75 | 9903.7 | 95.31% | 1.19(0.85-1.54) | 13.04 | 9.97 | -23.54% | -2.07(-2.45 - -1.69) | 1800.13 | 2498.39 | 38.79% | -0.19(-0.24 - -0.14) |
| Kyrgyzstan | IFBD | 221.64 | 390.26 | 76.08% | 0.12(0.06-0.18) | 15.26 | 9.75 | -36.11% | -3.52(-3.86 - -3.19) | 1109.15 | 1030.99 | -7.05% | -2.11(-2.28 - -1.94) |
| Kyrgyzstan | PACA | 1097.04 | 1835.25 | 67.29% | 0.11(0.08-0.15) | 40.9 | 85.76 | 109.68% | 1.12(0.75-1.49) | 1628.15 | 3473.22 | 113.32% | 1.07(0.69-1.44) |
| Kyrgyzstan | PIIO | 2554.51 | 4438.22 | 73.74% | 0.59(0.5-0.68) | 52.66 | 50.24 | -4.59% | -1.2(-1.49 - -0.92) | 2627.64 | 2039.12 | -22.40% | -2.37(-2.75 - -1.99) |
| Kyrgyzstan | UDSD | 161309.16 | 264378.56 | 63.90% | 0.001(-0.004-0.007) | 136.91 | 93.2 | -31.93% | -3.61(-3.99 - -3.22) | 8088.12 | 8979.14 | 11.02% | -2.01(-2.24 - -1.77) |
| Kyrgyzstan | VAID | 448.37 | 716.46 | 59.79% | 0.48(0.29-0.67) | 17.45 | 20.42 | 17.02% | -0.58(-0.82 - -0.34) | 442.01 | 479.33 | 8.44% | -1.39(-1.63 - -1.15) |
| Lao People's Democratic Republic | Digestive diseases | 101699.19 | 213935.27 | 110.36% | 0.08(0.08-0.09) | 2074.52 | 2464.12 | 18.78% | -2.04(-2.14 - -1.93) | 77567.62 | 86469.74 | 11.48% | -2.2(-2.3 - -2.11) |
| Lao People's Democratic Republic | APED | 11056.02 | 20906.5 | 89.10% | 0.34(0.12-0.57) | 38.42 | 26.06 | -32.17% | -3.27(-3.42 - -3.12) | 2347.51 | 1537 | -34.53% | -3.21(-3.38 - -3.04) |
| Lao People's Democratic Republic | COCLD | 739.52 | 1683.11 | 127.59% | 0.26(0.15-0.37) | 896.33 | 1272.77 | 42.00% | -1.45(-1.57 - -1.33) | 33203.06 | 43902.92 | 32.23% | -1.75(-1.86 - -1.63) |
| Lao People's Democratic Republic | GABD | 4838.66 | 13238.17 | 173.59% | 1.06(1.04-1.09) | 58.48 | 93.12 | 59.23% | -0.86(-0.95 - -0.76) | 3010.69 | 4454.47 | 47.96% | -1.23(-1.3 - -1.17) |
| Lao People's Democratic Republic | IFAH | 2031.36 | 6223.06 | 206.35% | 1.57(1.45-1.7) | 24.04 | 28.55 | 18.76% | -1.1(-1.15 - -1.06) | 1754.07 | 2288.39 | 30.46% | -0.69(-0.71 - -0.66) |
| Lao People's Democratic Republic | IFBD | 12.49 | 35 | 180.22% | 1.04(0.97-1.1) | 9.78 | 10.64 | 8.79% | -2.4(-2.6 - -2.19) | 385.01 | 386.94 | 0.50% | -2.47(-2.65 - -2.3) |
| Lao People's Democratic Republic | PACA | 694.06 | 1465.28 | 111.12% | -0.02(-0.02 - -0.02) | 44.35 | 67.82 | 52.92% | -1.14(-1.23 - -1.05) | 1735.98 | 2534.83 | 46.02% | -1.39(-1.47 - -1.31) |
| Lao People's Democratic Republic | PIIO | 1793.71 | 4516.79 | 151.81% | 1.28(1.19-1.37) | 87.37 | 157.14 | 79.87% | -0.14(-0.2 - -0.08) | 3998.44 | 5736.7 | 43.47% | -0.21(-0.3 - -0.12) |
| Lao People's Democratic Republic | UDSD | 80419.27 | 165540.96 | 105.85% | -0.091(-0.104 - -0.077) | 873.2 | 736.67 | -15.64% | -3.13(-3.26 - -3) | 29535.29 | 23562.42 | -20.22% | -3.36(-3.47 - -3.24) |
| Lao People's Democratic Republic | VAID | 114.1 | 326.39 | 186.06% | 1.63(1.5-1.76) | 9.09 | 21.58 | 137.40% | 0.35(0.23-0.46) | 224.65 | 463.21 | 106.19% | 0.02(-0.1-0.13) |
| Latvia | Digestive diseases | 202879.37 | 168734.92 | -16.83% | 0.07(0.02-0.12) | 798.41 | 944.75 | 18.33% | 0.19(-0.28-0.66) | 30335.21 | 30299.38 | -0.12% | 0.19(-0.24-0.62) |
| Latvia | APED | 8121.92 | 5286.16 | -34.91% | 0.2(-0.01-0.41) | 14.68 | 3.07 | -79.09% | -6.35(-7.08 - -5.63) | 531.61 | 130.16 | -75.52% | -3.99(-4.49 - -3.49) |
| Latvia | COCLD | 360.55 | 353.65 | -1.91% | 1.78(1.39-2.18) | 248.1 | 387.88 | 56.34% | 1.64(0.91-2.38) | 7817.57 | 12328.16 | 57.70% | 1.85(1.04-2.66) |
| Latvia | GABD | 35740.65 | 30906.39 | -13.53% | 0.17(-0.02-0.37) | 56.05 | 40.65 | -27.48% | -2.33(-2.55 - -2.1) | 4729.4 | 3268.46 | -30.89% | -0.98(-1.12 - -0.83) |
| Latvia | IFAH | 5337.05 | 4385.99 | -17.82% | 0.57(0.38-0.76) | 17.98 | 9.83 | -45.33% | -4.1(-4.55 - -3.65) | 1461.26 | 960.4 | -34.28% | -0.89(-1.04 - -0.74) |
| Latvia | IFBD | 160.97 | 154.39 | -4.09% | 0.53(0.28-0.78) | 11.56 | 13.69 | 18.43% | 0.22(-0.08-0.51) | 811.22 | 778.69 | -4.01% | 0.14(-0.02-0.29) |
| Latvia | PACA | 1982.04 | 1655.34 | -16.48% | -0.06(-0.1 - -0.02) | 100.97 | 100.23 | -0.73% | -0.03(-0.42-0.36) | 3597.28 | 3219.09 | -10.51% | -0.06(-0.46-0.34) |
| Latvia | PIIO | 4002.89 | 3527.92 | -11.87% | -0.04(-0.18-0.11) | 31.88 | 33.62 | 5.47% | -0.92(-1.1 - -0.74) | 961.83 | 667.25 | -30.63% | -1.91(-2.18 - -1.64) |
| Latvia | UDSD | 145680.7 | 120411.37 | -17.35% | 0.014(0.008-0.02) | 131.95 | 117.72 | -10.78% | -1.42(-1.95 - -0.88) | 6792.13 | 5139.02 | -24.34% | -0.96(-1.26 - -0.66) |
| Latvia | VAID | 1492.59 | 2053.7 | 37.59% | 0.14(-0.21-0.5) | 166.06 | 206.12 | 24.12% | -0.52(-0.87 - -0.17) | 2922.26 | 2973.89 | 1.77% | -0.99(-1.35 - -0.64) |
| Lebanon | Digestive diseases | 156437.05 | 318375.79 | 103.52% | 0.2(0.19-0.2) | 566.89 | 959.1 | 69.19% | -1.35(-1.44 - -1.25) | 20013.34 | 29820.46 | 49.00% | -1.06(-1.17 - -0.96) |
| Lebanon | APED | 6520.44 | 15020.52 | 130.36% | 1.44(1.4-1.48) | 9.49 | 10.67 | 12.43% | -2.58(-2.76 - -2.4) | 405.92 | 443.58 | 9.28% | -1.67(-1.89 - -1.45) |
| Lebanon | COCLD | 469.59 | 1109.65 | 136.30% | 0.98(0.89-1.08) | 357.93 | 596.34 | 66.61% | -1.33(-1.44 - -1.23) | 10057.76 | 14010.89 | 39.30% | -1.39(-1.54 - -1.25) |
| Lebanon | GABD | 5790.76 | 14570.96 | 151.62% | 0.84(0.8-0.88) | 20.22 | 56.5 | 179.43% | 0.14(-0.01-0.29) | 1042.06 | 2015.2 | 93.39% | -0.3(-0.42 - -0.18) |
| Lebanon | IFAH | 1793.66 | 3970.59 | 121.37% | 1.31(1.25-1.37) | 4.18 | 7.54 | 80.38% | -1.46(-1.59 - -1.33) | 538.77 | 864.48 | 60.45% | -0.17(-0.22 - -0.13) |
| Lebanon | IFBD | 65.15 | 193.54 | 197.07% | 1.39(1.29-1.49) | 3.05 | 7.52 | 146.56% | 0.32(0.1-0.55) | 219.9 | 545.48 | 148.06% | 0.79(0.65-0.93) |
| Lebanon | PACA | 713.26 | 1423.31 | 99.55% | -0.07(-0.1 - -0.04) | 18.12 | 36.42 | 100.99% | -0.89(-1 - -0.79) | 477.41 | 804.64 | 68.54% | -0.96(-1.13 - -0.79) |
| Lebanon | PIIO | 2995.37 | 8770.69 | 192.81% | 2.06(1.95-2.17) | 31.37 | 60.1 | 91.61% | -0.64(-0.73 - -0.56) | 1363.99 | 1533.44 | 12.42% | -0.79(-0.87 - -0.7) |
| Lebanon | UDSD | 137855.93 | 272497.73 | 97.67% | 0.035(0.029-0.041) | 99.15 | 113.58 | 14.55% | -2.86(-2.93 - -2.8) | 5313.47 | 8166.22 | 53.69% | -1.06(-1.12 - -1) |
| Lebanon | VAID | 232.89 | 818.8 | 251.58% | 2.03(1.98-2.09) | 12.85 | 33.16 | 158.05% | -0.32(-0.41 - -0.24) | 255.06 | 579.56 | 127.22% | -0.24(-0.36 - -0.13) |
| Lesotho | Digestive diseases | 78132.75 | 107279.8 | 37.30% | -0.01(-0.04-0.02) | 640.45 | 861.36 | 34.49% | 0.46(0.23-0.68) | 24086.51 | 32837.48 | 36.33% | 0.42(0.22-0.62) |
| Lesotho | APED | 5414.25 | 6266.88 | 15.75% | -0.35(-0.56 - -0.14) | 12.98 | 12.72 | -2.00% | -0.14(-0.44-0.17) | 749.19 | 694.36 | -7.32% | -0.23(-0.49-0.03) |
| Lesotho | COCLD | 343.41 | 431.83 | 25.75% | -0.64(-0.82 - -0.46) | 307.81 | 394.95 | 28.31% | 0.02(-0.14-0.18) | 10292.46 | 13717.54 | 33.28% | 0.13(-0.03-0.29) |
| Lesotho | GABD | 1205.3 | 1743.1 | 44.62% | 0.35(0.32-0.39) | 31.62 | 54.45 | 72.20% | 1.62(1.3-1.93) | 1063.66 | 1832.46 | 72.28% | 1.47(1.16-1.79) |
| Lesotho | IFAH | 3397 | 3975.47 | 17.03% | -0.36(-0.5 - -0.22) | 12.6 | 16.39 | 30.08% | 0.56(0.32-0.8) | 1568.63 | 1740.38 | 10.95% | -0.19(-0.27 - -0.1) |
| Lesotho | IFBD | 15.45 | 24.51 | 58.64% | 0.47(0.43-0.51) | 5.7 | 8.65 | 51.75% | 1.08(0.85-1.31) | 222.37 | 325.78 | 46.50% | 1.06(0.87-1.26) |
| Lesotho | PACA | 271.38 | 363.65 | 34.00% | -0.02(-0.07-0.02) | 14.05 | 23.24 | 65.41% | 0.9(0.65-1.14) | 487.12 | 863.25 | 77.22% | 1.05(0.8-1.29) |
| Lesotho | PIIO | 1789.37 | 2101.81 | 17.46% | -0.26(-0.4 - -0.11) | 88.75 | 116.37 | 31.12% | 0.46(0.28-0.65) | 2995.16 | 3830.49 | 27.89% | 0.3(0.16-0.44) |
| Lesotho | UDSD | 65618.22 | 92257.85 | 40.60% | 0.022(0.013-0.032) | 119.78 | 176.7 | 47.52% | 1.12(0.7-1.54) | 5173.31 | 7969.28 | 54.05% | 1.01(0.67-1.36) |
| Lesotho | VAID | 78.37 | 114.7 | 46.36% | 0.47(0.29-0.65) | 5.68 | 9.8 | 72.54% | 1.45(1.25-1.65) | 148.71 | 267.54 | 79.91% | 1.47(1.28-1.66) |
| Liberia | Digestive diseases | 72273.56 | 195296.33 | 170.22% | 0.11(0.1-0.12) | 1103.36 | 1523.62 | 38.09% | -1.2(-1.31 - -1.09) | 44329.35 | 63144.72 | 42.44% | -1.28(-1.4 - -1.17) |
| Liberia | APED | 2172.45 | 8235.08 | 279.07% | 1.29(1.15-1.43) | 19.5 | 11.56 | -40.72% | -3.27(-3.66 - -2.88) | 1208.48 | 631.76 | -47.72% | -4.32(-4.81 - -3.83) |
| Liberia | COCLD | 315.35 | 897.21 | 184.51% | 0.38(0.23-0.52) | 694.7 | 954.73 | 37.43% | -1.43(-1.57 - -1.29) | 23050.92 | 34739.15 | 50.71% | -1.39(-1.51 - -1.27) |
| Liberia | GABD | 546.12 | 1597.35 | 192.49% | 0.75(0.7-0.8) | 25.58 | 45.59 | 78.23% | 0.36(0.24-0.47) | 1062.07 | 1674.77 | 57.69% | -0.3(-0.4 - -0.2) |
| Liberia | IFAH | 2004.82 | 5531.76 | 175.92% | 0.9(0.72-1.08) | 20.2 | 22.65 | 12.13% | -1.13(-1.39 - -0.88) | 1561.89 | 2288.49 | 46.52% | -0.88(-1 - -0.76) |
| Liberia | IFBD | 14.66 | 42.8 | 191.95% | 0.43(0.19-0.66) | 7.25 | 10.62 | 46.48% | -0.84(-1.09 - -0.6) | 360.46 | 490.09 | 35.96% | -1.17(-1.44 - -0.89) |
| Liberia | PACA | 321.05 | 812.36 | 153.03% | 0.05(0.03-0.07) | 37.48 | 83.62 | 123.11% | -0.24(-0.45 - -0.03) | 1366.18 | 3219.41 | 135.65% | -0.27(-0.51 - -0.02) |
| Liberia | PIIO | 1483.3 | 4305.94 | 190.29% | 1.11(0.97-1.25) | 126.52 | 179.81 | 42.12% | -0.45(-0.62 - -0.29) | 7289.32 | 8556.56 | 17.38% | -1.11(-1.34 - -0.88) |
| Liberia | UDSD | 65323.63 | 173639.11 | 165.81% | 0.027(0.022-0.033) | 116.3 | 142.19 | 22.26% | -1.3(-1.46 - -1.14) | 5835.3 | 8851.31 | 51.69% | -1.17(-1.32 - -1.02) |
| Liberia | VAID | 92.17 | 234.72 | 154.66% | 1.02(0.84-1.19) | 10.19 | 15.76 | 54.66% | 0.46(0.32-0.59) | 456.83 | 494.34 | 8.21% | -0.5(-0.84 - -0.15) |
| Libya | Digestive diseases | 171085.55 | 412409.73 | 141.05% | 0.12(0.11-0.12) | 663.72 | 1121.7 | 69.00% | -1.4(-1.53 - -1.27) | 25720.79 | 40604.22 | 57.87% | -1.27(-1.38 - -1.17) |
| Libya | APED | 9093.53 | 20448.23 | 124.87% | 1.21(1.18-1.23) | 14.27 | 17.25 | 20.88% | -2.07(-2.33 - -1.8) | 734.53 | 766.28 | 4.32% | -1.84(-2.07 - -1.61) |
| Libya | COCLD | 548.03 | 1142.37 | 108.45% | -0.22(-0.33 - -0.11) | 416.74 | 723.88 | 73.70% | -1.43(-1.55 - -1.32) | 13391.23 | 20416.53 | 52.46% | -1.55(-1.66 - -1.43) |
| Libya | GABD | 5547.55 | 16122.36 | 190.62% | 0.44(0.42-0.47) | 23.25 | 52.68 | 126.58% | -0.15(-0.36-0.05) | 1114.09 | 2464.43 | 121.21% | -0.43(-0.54 - -0.32) |
| Libya | IFAH | 2346.05 | 4105.35 | 74.99% | 0.47(0.35-0.59) | 4.62 | 7.37 | 59.52% | -1.39(-1.55 - -1.23) | 732.42 | 1097.82 | 49.89% | -0.47(-0.62 - -0.33) |
| Libya | IFBD | 51.28 | 177.95 | 247.02% | 1.02(0.72-1.32) | 4.12 | 10.68 | 159.22% | 0.11(-0.02-0.24) | 228.83 | 594.99 | 160.01% | 0.21(0.12-0.31) |
| Libya | PACA | 752.4 | 1770.59 | 135.33% | -0.03(-0.06-0) | 18.72 | 38.91 | 107.85% | -0.8(-0.92 - -0.68) | 529.18 | 1059.51 | 100.22% | -0.9(-1.02 - -0.78) |
| Libya | PIIO | 3170.73 | 6285.91 | 98.25% | 0.7(0.5-0.9) | 50.42 | 74.2 | 47.17% | -0.94(-1.06 - -0.83) | 2350.12 | 1738.14 | -26.04% | -1.51(-1.64 - -1.37) |
| Libya | UDSD | 149327.61 | 361693.91 | 142.22% | 0.037(0.034-0.039) | 106.12 | 127.12 | 19.79% | -2.66(-2.9 - -2.41) | 5937.15 | 10792.53 | 81.78% | -1.2(-1.32 - -1.07) |
| Libya | VAID | 248.37 | 663.05 | 166.96% | 0.83(0.73-0.93) | 12.25 | 27.37 | 123.43% | -0.61(-0.69 - -0.54) | 240.66 | 541.27 | 124.91% | -0.58(-0.65 - -0.51) |
| Lithuania | Digestive diseases | 280203.35 | 260543.35 | -7.02% | 0.11(0.06-0.17) | 910.7 | 1881.68 | 106.62% | 2.66(1.94-3.38) | 37928.48 | 59313.73 | 56.38% | 2.15(1.48-2.82) |
| Lithuania | APED | 11598.83 | 7898.37 | -31.90% | 0.18(0.05-0.3) | 16.76 | 8.99 | -46.36% | -2.71(-3.34 - -2.07) | 613.07 | 286.32 | -53.30% | -1.97(-2.38 - -1.57) |
| Lithuania | COCLD | 529.6 | 677.79 | 27.98% | 2.84(2.25-3.44) | 353.14 | 830.46 | 135.16% | 3.7(2.58-4.83) | 11903.95 | 27626.47 | 132.08% | 3.87(2.69-5.05) |
| Lithuania | GABD | 49715.31 | 50494.87 | 1.57% | 0.24(0.01-0.47) | 71.31 | 99.5 | 39.53% | -0.1(-0.52-0.32) | 6108.66 | 5789.41 | -5.23% | -0.33(-0.46 - -0.2) |
| Lithuania | IFAH | 8657.69 | 8373.18 | -3.29% | 0.72(0.61-0.82) | 29.78 | 27.97 | -6.08% | -1.82(-2.19 - -1.44) | 2335.06 | 1949.26 | -16.52% | -0.36(-0.42 - -0.3) |
| Lithuania | IFBD | 233 | 257.64 | 10.58% | 1.22(1.15-1.29) | 14.11 | 17.82 | 26.29% | 0.13(-0.2-0.47) | 1054.22 | 1104.51 | 4.77% | 0.49(0.33-0.65) |
| Lithuania | PACA | 2477.26 | 2647.73 | 6.88% | 0.34(0.31-0.37) | 105.86 | 183.54 | 73.38% | 2.32(1.83-2.82) | 3684.47 | 5429.79 | 47.37% | 2.25(1.73-2.77) |
| Lithuania | PIIO | 5829.2 | 5013.27 | -14.00% | -0.59(-0.86 - -0.32) | 44.04 | 64.15 | 45.65% | 0.42(0.2-0.65) | 1288.2 | 1253.28 | -2.71% | -0.25(-0.43 - -0.06) |
| Lithuania | UDSD | 199788.06 | 182340.27 | -8.73% | 0.044(0.04-0.048) | 134.18 | 260.23 | 93.94% | 1.8(1.35-2.24) | 7986.96 | 9502.99 | 18.98% | 0.72(0.49-0.96) |
| Lithuania | VAID | 1374.39 | 2840.24 | 106.65% | 0.93(0.67-1.2) | 118.86 | 303.09 | 155.00% | 2.37(2-2.74) | 2090 | 4418.08 | 111.39% | 1.91(1.56-2.27) |
| Luxembourg | Digestive diseases | 21610.85 | 36918.07 | 70.83% | -0.01(-0.13-0.11) | 187.3 | 212.26 | 13.32% | -2.16(-2.27 - -2.04) | 5162.17 | 5481.02 | 6.18% | -1.98(-2.08 - -1.88) |
| Luxembourg | APED | 1133.72 | 1709.3 | 50.77% | -0.06(-0.25-0.13) | 1.6 | 1.1 | -31.25% | -3.72(-3.91 - -3.54) | 45.71 | 38.84 | -15.03% | -1.93(-2.03 - -1.84) |
| Luxembourg | COCLD | 157.9 | 196.06 | 24.17% | -1.36(-1.62 - -1.1) | 99.74 | 90.31 | -9.45% | -2.72(-2.86 - -2.57) | 2841.12 | 2383.9 | -16.09% | -2.91(-3.03 - -2.79) |
| Luxembourg | GABD | 2220.46 | 3932.96 | 77.12% | -0.05(-0.72-0.63) | 10.95 | 14.83 | 35.43% | -1.45(-1.58 - -1.32) | 340.4 | 446.82 | 31.26% | -1.04(-1.41 - -0.67) |
| Luxembourg | IFAH | 492.62 | 858.9 | 74.35% | 0.89(0.55-1.24) | 5.52 | 4.77 | -13.59% | -3.25(-3.54 - -2.96) | 182.37 | 202.96 | 11.29% | -0.92(-1.1 - -0.75) |
| Luxembourg | IFBD | 83.86 | 111.24 | 32.65% | -0.49(-0.65 - -0.33) | 4.89 | 10.97 | 124.34% | 0.55(0.3-0.8) | 202.16 | 375.64 | 85.81% | 0.27(0.15-0.38) |
| Luxembourg | PACA | 125.74 | 242.01 | 92.47% | -0.04(-0.14-0.05) | 6.79 | 7.8 | 14.87% | -1.99(-2.07 - -1.9) | 172.01 | 183.57 | 6.72% | -1.98(-2.04 - -1.92) |
| Luxembourg | PIIO | 520.08 | 1172.22 | 125.39% | 0.71(0.55-0.87) | 13.56 | 26.48 | 95.28% | -0.5(-0.64 - -0.36) | 219.64 | 352.76 | 60.61% | -0.75(-0.85 - -0.64) |
| Luxembourg | UDSD | 16711.94 | 28374.38 | 69.79% | -0.037(-0.06 - -0.014) | 18.61 | 11.46 | -38.42% | -4.78(-4.92 - -4.63) | 725.57 | 847.22 | 16.77% | -1.52(-1.6 - -1.43) |
| Luxembourg | VAID | 164.53 | 321 | 95.10% | -0.66(-0.91 - -0.41) | 18.17 | 26.13 | 43.81% | -1.65(-1.91 - -1.38) | 292.98 | 365.71 | 24.82% | -1.8(-2.05 - -1.54) |
| Madagascar | Digestive diseases | 425429.96 | 1071083.88 | 151.77% | 0.13(0.12-0.15) | 4221.54 | 7189.48 | 70.30% | -0.58(-0.65 - -0.5) | 177008.17 | 298137.42 | 68.43% | -0.69(-0.72 - -0.66) |
| Madagascar | APED | 17302.94 | 48938.35 | 182.83% | 0.65(0.5-0.79) | 103.55 | 127.58 | 23.21% | -0.53(-0.75 - -0.32) | 6797.85 | 7119.89 | 4.74% | -1.24(-1.57 - -0.9) |
| Madagascar | COCLD | 1900.85 | 5724.26 | 201.14% | 0.93(0.68-1.18) | 2798.27 | 4523.78 | 61.66% | -1.03(-1.19 - -0.87) | 99881.89 | 161143.98 | 61.33% | -1.12(-1.24 - -1.01) |
| Madagascar | GABD | 3149.55 | 8716.56 | 176.76% | 0.8(0.77-0.83) | 144.62 | 320.36 | 121.52% | 0.78(0.7-0.85) | 4905.39 | 10458.95 | 113.21% | 0.5(0.4-0.6) |
| Madagascar | IFAH | 7599.22 | 20779.03 | 173.44% | 1.13(1-1.26) | 100.94 | 150.41 | 49.01% | 0.32(0.18-0.45) | 7819.2 | 11711.77 | 49.78% | 0.03(-0.13-0.19) |
| Madagascar | IFBD | 79.3 | 207.07 | 161.12% | 0.3(0.27-0.32) | 36.08 | 69.31 | 92.10% | 0.45(0.34-0.56) | 1874.81 | 2999.93 | 60.01% | -0.03(-0.18-0.13) |
| Madagascar | PACA | 1467.48 | 3564.48 | 142.90% | 0.09(0.05-0.13) | 62.98 | 148.1 | 135.15% | 0.35(0.18-0.52) | 2446.75 | 5775.9 | 136.06% | 0.29(0.08-0.51) |
| Madagascar | PIIO | 9188.71 | 23401.97 | 154.68% | 0.51(0.41-0.61) | 362.61 | 727.41 | 100.60% | 0.44(0.31-0.57) | 19766.03 | 34666.23 | 75.38% | 0.22(0.05-0.4) |
| Madagascar | UDSD | 384257.92 | 958478.09 | 149.44% | 0.082(0.074-0.091) | 398.09 | 686.46 | 72.44% | -0.27(-0.38 - -0.16) | 25514.59 | 49945.22 | 95.75% | -0.25(-0.37 - -0.14) |
| Madagascar | VAID | 483.99 | 1274.06 | 163.24% | 1(0.89-1.12) | 45.57 | 103.99 | 128.20% | 0.8(0.71-0.9) | 1221.6 | 2691.08 | 120.29% | 0.71(0.6-0.83) |
| Malawi | Digestive diseases | 340614.53 | 716100.66 | 110.24% | 0.17(0.16-0.18) | 4386.39 | 5844.97 | 33.25% | -1.27(-1.46 - -1.08) | 185023.07 | 230796.68 | 24.74% | -1.31(-1.5 - -1.11) |
| Malawi | APED | 13063.04 | 36340.16 | 178.19% | 1.18(1.06-1.3) | 117.72 | 108.49 | -7.84% | -1.6(-1.73 - -1.47) | 7870.74 | 6035.37 | -23.32% | -2.29(-2.42 - -2.16) |
| Malawi | COCLD | 2095.8 | 3866.05 | 84.47% | -0.22(-0.3 - -0.15) | 2900.1 | 3690.73 | 27.26% | -1.63(-1.91 - -1.35) | 102911.68 | 127118.53 | 23.52% | -1.68(-1.98 - -1.38) |
| Malawi | GABD | 2732.15 | 6558.38 | 140.04% | 0.87(0.8-0.93) | 150.65 | 280.18 | 85.98% | 0.02(-0.05-0.08) | 5106.96 | 7970.4 | 56.07% | -0.26(-0.33 - -0.18) |
| Malawi | IFAH | 6413.27 | 16320.92 | 154.49% | 1.67(1.49-1.86) | 123.42 | 132.49 | 7.35% | -0.51(-0.62 - -0.4) | 9599.96 | 9438.08 | -1.69% | -0.5(-0.67 - -0.34) |
| Malawi | IFBD | 60.37 | 138.88 | 130.05% | 0.53(0.47-0.6) | 39.29 | 54.8 | 39.48% | -0.26(-0.39 - -0.12) | 2236.21 | 2323.24 | 3.89% | -0.85(-1.02 - -0.69) |
| Malawi | PACA | 1198.04 | 2494.23 | 108.19% | 0.08(0.03-0.12) | 66.17 | 130.36 | 97.01% | -0.08(-0.22-0.06) | 2526.45 | 4930.67 | 95.16% | -0.02(-0.17-0.14) |
| Malawi | PIIO | 7465.54 | 16853.09 | 125.75% | 0.9(0.83-0.97) | 410.2 | 630 | 53.58% | -0.15(-0.28 - -0.01) | 23741.87 | 28860.11 | 21.56% | -0.29(-0.41 - -0.18) |
| Malawi | UDSD | 307201.72 | 632537.35 | 105.90% | 0.086(0.08-0.092) | 348.6 | 448.16 | 28.56% | -1.58(-1.78 - -1.38) | 21333.57 | 32707.05 | 53.31% | -0.92(-1.06 - -0.78) |
| Malawi | VAID | 384.59 | 991.61 | 157.84% | 1.38(1.23-1.52) | 41.53 | 93.2 | 124.42% | 0.54(0.44-0.63) | 1191.52 | 2210.96 | 85.56% | 0.42(0.31-0.53) |
| Malaysia | Digestive diseases | 464652.93 | 1090265.35 | 134.64% | 0.21(0.19-0.23) | 3495.64 | 10072.29 | 188.14% | -0.61(-1.07 - -0.16) | 119455.44 | 287897 | 141.01% | -0.72(-0.98 - -0.45) |
| Malaysia | APED | 34749.03 | 79149.22 | 127.77% | 0.88(0.73-1.03) | 47.92 | 66.95 | 39.71% | -2.3(-2.57 - -2.03) | 2530.83 | 3199.19 | 26.41% | -1.91(-2.07 - -1.74) |
| Malaysia | COCLD | 2622.6 | 6861.08 | 161.61% | 0.32(0.15-0.48) | 1651.48 | 4782.89 | 189.61% | -0.51(-0.87 - -0.15) | 53683.39 | 135961.7 | 153.27% | -0.65(-0.89 - -0.41) |
| Malaysia | GABD | 31816.12 | 83209.71 | 161.53% | 0.42(0.39-0.46) | 243.97 | 809.03 | 231.61% | 0.14(-0.25-0.54) | 9394.77 | 23527.3 | 150.43% | -0.33(-0.49 - -0.17) |
| Malaysia | IFAH | 14812.92 | 53850.58 | 263.54% | 1.1(0.95-1.26) | 95.28 | 264.02 | 177.10% | -0.52(-0.88 - -0.16) | 5501.64 | 14258.44 | 159.17% | -0.12(-0.25-0.02) |
| Malaysia | IFBD | 72.05 | 251.19 | 248.63% | 1.6(1.47-1.73) | 16.09 | 41.44 | 157.55% | -0.75(-1.06 - -0.43) | 612.35 | 1434.47 | 134.26% | -0.54(-0.73 - -0.35) |
| Malaysia | PACA | 3640.59 | 8262.08 | 126.94% | -0.09(-0.12 - -0.05) | 237.51 | 635.18 | 167.43% | -0.54(-0.86 - -0.22) | 7806.26 | 18361.62 | 135.22% | -0.71(-0.91 - -0.5) |
| Malaysia | PIIO | 19739.61 | 57467.23 | 191.13% | 1.23(1.09-1.37) | 342.68 | 1223.49 | 257.03% | 0(-0.67-0.68) | 10864.32 | 27335.03 | 151.60% | -0.2(-0.66-0.27) |
| Malaysia | UDSD | 356254.62 | 797555.02 | 123.87% | 0.001(-0.004-0.006) | 612.98 | 1332.41 | 117.37% | -2.09(-2.78 - -1.41) | 22869.66 | 45131.22 | 97.34% | -1.72(-2.09 - -1.34) |
| Malaysia | VAID | 945.38 | 3659.23 | 287.06% | 1.83(1.67-1.99) | 69.08 | 341.61 | 394.51% | 1.56(1.11-2.02) | 1541.13 | 6505.23 | 322.11% | 1.44(1.14-1.74) |
| Maldives | Digestive diseases | 4835.91 | 17118.44 | 253.99% | 0.31(0.3-0.33) | 31 | 49.18 | 58.68% | -3.08(-3.31 - -2.84) | 1365.17 | 2017.72 | 47.80% | -2.95(-3.22 - -2.67) |
| Maldives | APED | 366.28 | 1134.45 | 209.72% | 1.18(1.16-1.2) | 0.21 | 0.12 | -42.86% | -5.67(-5.99 - -5.36) | 14.69 | 17.45 | 18.79% | -2.68(-3.05 - -2.31) |
| Maldives | COCLD | 19.9 | 76.1 | 282.41% | 0.31(0.07-0.54) | 20.05 | 32.64 | 62.79% | -2.86(-3.12 - -2.6) | 770.64 | 1066.32 | 38.37% | -3.36(-3.68 - -3.04) |
| Maldives | GABD | 248.17 | 1068.57 | 330.58% | 0.87(0.78-0.95) | 0.57 | 1.14 | 100.00% | -2.62(-2.82 - -2.42) | 51.11 | 111.99 | 119.12% | -1.85(-1.96 - -1.73) |
| Maldives | IFAH | 115.13 | 588.02 | 410.74% | 1.87(1.69-2.05) | 0.67 | 1.03 | 53.73% | -3.2(-3.39 - -3.01) | 54.27 | 116.77 | 115.16% | -1.02(-1.26 - -0.77) |
| Maldives | IFBD | 0.58 | 2.84 | 389.66% | 1.26(1.17-1.34) | 0.44 | 0.87 | 97.73% | -2.37(-2.52 - -2.22) | 18.52 | 29.28 | 58.10% | -2.5(-2.68 - -2.32) |
| Maldives | PACA | 28.34 | 103.88 | 266.55% | -0.12(-0.17 - -0.07) | 0.96 | 1.62 | 68.75% | -3.26(-3.5 - -3.03) | 36.51 | 60.33 | 65.24% | -3.44(-3.76 - -3.11) |
| Maldives | PIIO | 192.27 | 970.94 | 404.98% | 2.77(2.61-2.92) | 2.24 | 4.02 | 79.41% | -1.67(-1.74 - -1.61) | 129.43 | 125.64 | -2.93% | -1.87(-1.98 - -1.76) |
| Maldives | UDSD | 3857.98 | 13141.46 | 240.63% | -0.012(-0.019 - -0.006) | 4.29 | 3.81 | -11.19% | -5.63(-6 - -5.27) | 235.62 | 406.56 | 72.55% | -3.02(-3.3 - -2.73) |
| Maldives | VAID | 7.26 | 32.18 | 343.25% | 1.99(1.92-2.07) | 0.41 | 1.47 | 258.54% | -0.72(-0.87 - -0.57) | 10.87 | 26.08 | 139.93% | -1.34(-1.5 - -1.17) |
| Mali | Digestive diseases | 308099.25 | 777604.83 | 152.39% | 0.1(0.09-0.12) | 4152.51 | 6246.38 | 50.42% | -1.39(-1.47 - -1.3) | 178422.14 | 278398.2 | 56.03% | -1.34(-1.42 - -1.26) |
| Mali | APED | 9947 | 35065.05 | 252.52% | 0.88(0.76-0.99) | 87.79 | 74.85 | -14.74% | -2.72(-3 - -3) | 5660.37 | 4494.81 | -20.59% | -3.52(-3.84 - -3.21) |
| Mali | COCLD | 1683.6 | 5093.22 | 202.52% | 1.14(1-1.28) | 2579.25 | 3266.78 | 26.66% | -2.26(-2.48 - -2.04) | 92805.71 | 122983.58 | 32.52% | -2.16(-2.36 - -1.97) |
| Mali | GABD | 2454.25 | 7459 | 203.92% | 0.96(0.91-1.01) | 100.83 | 231.36 | 129.46% | 0.57(0.48-0.67) | 4631.43 | 9274.21 | 100.25% | 0.08(0.01-0.15) |
| Mali | IFAH | 9449.7 | 32056.2 | 239.23% | 2.17(2.02-2.31) | 73.55 | 141.31 | 92.13% | 0.34(0.11-0.57) | 6747.63 | 14460.91 | 114.31% | 0.71(0.52-0.91) |
| Mali | IFBD | 53.41 | 143.12 | 167.96% | 0.24(0.11-0.37) | 28.73 | 54.62 | 90.11% | -0.28(-0.45 - -0.1) | 1568.57 | 2748.35 | 75.21% | -0.64(-0.8 - -0.48) |
| Mali | PACA | 1341.92 | 3446.34 | 156.82% | 0.26(0.24-0.29) | 144.26 | 355.77 | 146.62% | 0.48(0.29-0.67) | 5556.73 | 13776.93 | 147.93% | 0.5(0.29-0.71) |
| Mali | PIIO | 6066.84 | 18488.81 | 204.75% | 1.02(0.87-1.16) | 438.55 | 956.62 | 118.13% | 0.4(0.28-0.52) | 24373.29 | 47788.21 | 96.07% | 0.04(-0.1-0.18) |
| Mali | UDSD | 276724.63 | 674624.03 | 143.79% | -0.015(-0.034-0.004) | 493.91 | 827.14 | 67.47% | -0.49(-0.73 - -0.25) | 26725.16 | 47834.41 | 78.99% | -0.54(-0.74 - -0.34) |
| Mali | VAID | 377.9 | 1229.07 | 225.24% | 1.57(1.47-1.66) | 34.78 | 79.84 | 129.56% | 0.93(0.84-1.03) | 1722.03 | 3033.15 | 76.14% | 0.21(0.09-0.32) |
| Malta | Digestive diseases | 17396.13 | 25490.26 | 46.53% | 0.15(0.11-0.19) | 88.45 | 123.55 | 39.68% | -1.82(-1.92 - -1.71) | 2559.24 | 3079.48 | 20.33% | -1.21(-1.29 - -1.14) |
| Malta | APED | 750.32 | 859.49 | 14.55% | 0.48(0.37-0.58) | 0.65 | 0.56 | -13.85% | -3.31(-3.47 - -3.15) | 23.37 | 20.02 | -14.33% | -1.21(-1.36 - -1.06) |
| Malta | COCLD | 66.92 | 61.12 | -8.67% | -0.6(-0.67 - -0.54) | 36.71 | 40.47 | 10.24% | -1.99(-2.09 - -1.9) | 1029.35 | 1019.06 | -1.00% | -1.79(-1.86 - -1.72) |
| Malta | GABD | 814.26 | 1348.81 | 65.65% | 0.89(0.48-1.3) | 6.18 | 10.92 | 76.70% | -1.27(-1.37 - -1.17) | 164.59 | 237.97 | 44.58% | -0.59(-0.8 - -0.37) |
| Malta | IFAH | 467.66 | 736.92 | 57.58% | 0.91(0.48-1.34) | 4.21 | 4.48 | 6.41% | -3.18(-3.43 - -2.93) | 163.87 | 180.64 | 10.23% | -0.77(-1 - -0.54) |
| Malta | IFBD | 31.61 | 43.83 | 38.66% | 0.32(0.24-0.39) | 2.12 | 5.97 | 181.60% | 0.78(0.57-0.99) | 83.88 | 148.6 | 77.16% | 0.4(0.3-0.5) |
| Malta | PACA | 70.73 | 123.13 | 74.08% | -0.08(-0.16-0.01) | 4.63 | 6.68 | 44.28% | -1.48(-1.63 - -1.34) | 115.61 | 145.49 | 25.85% | -1.15(-1.25 - -1.05) |
| Malta | PIIO | 409.87 | 851.26 | 107.69% | 0.55(0.5-0.59) | 6.8 | 15.72 | 131.35% | -0.56(-0.78 - -0.34) | 124.52 | 223.22 | 79.27% | -0.54(-0.71 - -0.37) |
| Malta | UDSD | 14736.75 | 21339.9 | 44.81% | 0.045(0.036-0.055) | 16.67 | 12.42 | -25.49% | -4.53(-4.67 - -4.39) | 663.14 | 707.35 | 6.67% | -1.41(-1.49 - -1.33) |
| Malta | VAID | 48 | 125.8 | 162.08% | 0.14(-0.05-0.32) | 6.52 | 12.88 | 97.55% | -1.29(-1.61 - -0.97) | 111.36 | 190.65 | 71.20% | -1.31(-1.61 - -1) |
| Marshall Islands | Digestive diseases | 938.59 | 1556.93 | 65.88% | -0.03(-0.05 - -0.01) | 13.76 | 17.16 | 24.74% | -1.59(-1.64 - -1.54) | 545.19 | 689 | 26.38% | -1.49(-1.53 - -1.45) |
| Marshall Islands | APED | 49.09 | 69.3 | 41.17% | 0.22(0.13-0.32) | 0.2 | 0.22 | 10.00% | -1.55(-1.64 - -1.46) | 9.71 | 10.31 | 6.18% | -1.49(-1.61 - -1.38) |
| Marshall Islands | COCLD | 6.92 | 9.79 | 41.47% | -0.57(-0.67 - -0.47) | 7.47 | 10.16 | 36.01% | -1.51(-1.56 - -1.46) | 293.21 | 392.67 | 33.92% | -1.48(-1.53 - -1.44) |
| Marshall Islands | GABD | 45.13 | 83.05 | 84.02% | 0.22(0.19-0.25) | 0.75 | 1.12 | 49.33% | -0.66(-0.75 - -0.58) | 29.03 | 46.84 | 61.35% | -0.59(-0.67 - -0.51) |
| Marshall Islands | IFAH | 25.51 | 45.45 | 78.17% | 0.42(0.32-0.51) | 0.22 | 0.25 | 13.64% | -1.26(-1.39 - -1.13) | 13.58 | 18.19 | 33.95% | -0.67(-0.74 - -0.6) |
| Marshall Islands | IFBD | 0.15 | 0.33 | 120.00% | 1.14(1.02-1.25) | 0.25 | 0.29 | 16.00% | -1.9(-2.04 - -1.76) | 10.2 | 11.78 | 15.49% | -1.8(-1.93 - -1.68) |
| Marshall Islands | PACA | 7.23 | 12.24 | 69.29% | -0.07(-0.08 - -0.07) | 0.46 | 0.67 | 45.65% | -1.04(-1.09 - -0.99) | 18.75 | 27.15 | 44.80% | -0.91(-0.96 - -0.86) |
| Marshall Islands | PIIO | 19.27 | 26.49 | 37.47% | 0.28(0.17-0.4) | 0.6 | 0.71 | 18.20% | -0.98(-1.09 - -0.86) | 22.04 | 24.2 | 9.78% | -1.04(-1.13 - -0.94) |
| Marshall Islands | UDSD | 783.87 | 1307.62 | 66.82% | -0.079(-0.093 - -0.065) | 3.33 | 2.98 | -10.51% | -2.41(-2.49 - -2.33) | 132.19 | 131.77 | -0.32% | -2.17(-2.24 - -2.09) |
| Marshall Islands | VAID | 1.41 | 2.66 | 88.65% | 0.73(0.66-0.8) | 0.13 | 0.24 | 84.62% | -0.24(-0.38 - -0.09) | 3.3 | 6.81 | 106.36% | -0.05(-0.21-0.11) |
| Mauritania | Digestive diseases | 75208.55 | 163482.5 | 117.37% | 0.08(0.07-0.09) | 978.58 | 1025.71 | 4.82% | -2.4(-2.49 - -2.31) | 35906.44 | 37790.52 | 5.25% | -2.31(-2.38 - -2.23) |
| Mauritania | APED | 2415.12 | 6976 | 188.85% | 1.17(1.04-1.31) | 14.66 | 7.35 | -49.86% | -4.49(-4.7 - -4.29) | 697.89 | 336.36 | -51.80% | -4.88(-5.13 - -4.63) |
| Mauritania | COCLD | 432.58 | 825.98 | 90.94% | -0.4(-0.54 - -0.25) | 595.5 | 602.66 | 1.20% | -2.63(-2.71 - -2.54) | 19382.23 | 19381.76 | 0.00% | -2.61(-2.69 - -2.54) |
| Mauritania | GABD | 595.87 | 1444.72 | 142.46% | 0.62(0.59-0.66) | 24.2 | 34.54 | 42.73% | -1.27(-1.37 - -1.17) | 843.73 | 1048.12 | 24.22% | -1.52(-1.62 - -1.43) |
| Mauritania | IFAH | 2184.82 | 5262.84 | 140.88% | 0.92(0.85-0.99) | 15.37 | 16.94 | 10.21% | -1.84(-1.94 - -1.73) | 1215.99 | 1779.53 | 46.34% | -0.84(-0.89 - -0.8) |
| Mauritania | IFBD | 15.71 | 33.8 | 115.15% | 0.06(-0.12-0.24) | 7.77 | 9.32 | 19.95% | -2.01(-2.18 - -1.84) | 321.89 | 376.37 | 16.93% | -1.97(-2.14 - -1.8) |
| Mauritania | PACA | 340.96 | 702.45 | 106.02% | -0.09(-0.1 - -0.07) | 44.38 | 55.87 | 25.89% | -1.8(-1.89 - -1.71) | 1573.71 | 1951.4 | 24.00% | -1.87(-1.94 - -1.79) |
| Mauritania | PIIO | 1682.83 | 4876.1 | 189.76% | 1.32(1.23-1.4) | 105.4 | 140.68 | 33.46% | -1.29(-1.46 - -1.13) | 4530.33 | 5024.81 | 10.91% | -1.55(-1.72 - -1.37) |
| Mauritania | UDSD | 67431.33 | 143086.91 | 112.20% | -0.012(-0.017 - -0.007) | 115.92 | 97.89 | -15.55% | -2.85(-2.9 - -2.79) | 5358.36 | 6085.98 | 13.58% | -2(-2.06 - -1.94) |
| Mauritania | VAID | 109.32 | 273.71 | 150.38% | 0.98(0.92-1.04) | 9.69 | 14.98 | 54.59% | -0.89(-1.04 - -0.74) | 315.84 | 374.67 | 18.63% | -1.33(-1.47 - -1.18) |
| Mauritius | Digestive diseases | 32276.27 | 50022.76 | 54.98% | 0.06(0.05-0.07) | 375.83 | 397.51 | 5.77% | -2.88(-3.15 - -2.62) | 14396.99 | 13531.92 | -6.01% | -2.78(-3.04 - -2.51) |
| Mauritius | APED | 1985.16 | 2700.24 | 36.02% | 0.87(0.84-0.89) | 0.77 | 0.85 | 10.39% | -1.93(-2.35 - -1.52) | 54.3 | 55.93 | 3.00% | -0.46(-0.71 - -0.21) |
| Mauritius | COCLD | 314.95 | 252.62 | -19.79% | -2.06(-2.32 - -1.81) | 265.35 | 263.21 | -0.81% | -3.31(-3.69 - -2.94) | 9621.44 | 8329.25 | -13.43% | -3.43(-3.82 - -3.03) |
| Mauritius | GABD | 1989.13 | 3505.71 | 76.24% | 0.4(0.34-0.47) | 4.89 | 13.24 | 170.76% | 0.62(0.25-1.01) | 336.68 | 604.72 | 79.61% | 0.15(-0.06-0.37) |
| Mauritius | IFAH | 668.13 | 1584.01 | 137.08% | 0.9(0.72-1.08) | 1.97 | 2.58 | 30.96% | -2.31(-2.53 - -2.08) | 200.82 | 335.07 | 66.85% | -0.29(-0.45 - -0.12) |
| Mauritius | IFBD | 4.96 | 8.8 | 77.42% | 0.81(0.78-0.84) | 1.26 | 3.47 | 175.40% | 1.44(1.1-1.78) | 49.81 | 116.63 | 134.15% | 1.88(1.53-2.23) |
| Mauritius | PACA | 262.61 | 377.1 | 43.60% | -0.33(-0.37 - -0.29) | 31.37 | 31.04 | -1.05% | -2.44(-2.8 - -2.08) | 1371.02 | 1203.09 | -12.25% | -2.39(-2.72 - -2.06) |
| Mauritius | PIIO | 1674.56 | 2932.77 | 75.14% | 0.08(-0.02-0.17) | 8.44 | 22.34 | 164.76% | 1.06(0.87-1.25) | 352.07 | 538.36 | 52.91% | 0.41(0.28-0.54) |
| Mauritius | UDSD | 25322.59 | 38531.41 | 52.16% | -0.036(-0.04 - -0.031) | 56.52 | 38.33 | -32.18% | -4.31(-4.51 - -4.11) | 2241.83 | 1805.48 | -19.46% | -2.79(-2.93 - -2.64) |
| Mauritius | VAID | 54.18 | 130.12 | 140.16% | 0.86(0.8-0.91) | 2.53 | 7.45 | 194.47% | 0.28(0.12-0.44) | 54.99 | 144.65 | 163.05% | 0.48(0.27-0.69) |
| Mexico | Digestive diseases | 5623738.44 | 10845926.08 | 92.86% | 0.14(0.11-0.18) | 36292.35 | 71123.87 | 95.97% | -1.14(-1.29 - -0.99) | 1454468.33 | 2364013.8 | 62.53% | -1.19(-1.33 - -1.04) |
| Mexico | APED | 222485.73 | 360798.4 | 62.17% | 1.09(0.89-1.28) | 547.24 | 1133.92 | 107.21% | 0.18(-0.01-0.37) | 26973.14 | 40134.97 | 48.80% | 0.04(-0.15-0.23) |
| Mexico | COCLD | 39192.54 | 73785.2 | 88.26% | -0.05(-0.1-0) | 24012.63 | 46327.64 | 92.93% | -1.27(-1.42 - -1.11) | 845547.81 | 1392883.09 | 64.73% | -1.55(-1.74 - -1.37) |
| Mexico | GABD | 698957.56 | 1641466.91 | 134.85% | 1.21(0.86-1.56) | 1423.62 | 3723.39 | 161.54% | 0.02(-0.28-0.33) | 110797.33 | 218206.78 | 96.94% | 0.13(-0.05-0.3) |
| Mexico | IFAH | 260385.9 | 417495.28 | 60.34% | -0.4(-0.58 - -0.22) | 865.6 | 1637.06 | 89.12% | -1.02(-1.43 - -0.6) | 71455.57 | 96052.64 | 34.42% | -1.07(-1.39 - -0.74) |
| Mexico | IFBD | 2540.3 | 3944.47 | 55.28% | -0.91(-1.09 - -0.72) | 157.46 | 519.42 | 229.87% | 1.67(1.33-2.02) | 9625.55 | 21132.46 | 119.55% | 0.68(0.41-0.96) |
| Mexico | PACA | 23224.07 | 50891.19 | 119.13% | 0.08(0.02-0.14) | 1102.93 | 2511.34 | 127.70% | -0.08(-0.27-0.11) | 43519.53 | 82889.16 | 90.46% | -0.26(-0.44 - -0.08) |
| Mexico | PIIO | 85940.97 | 154532.36 | 79.81% | 0.33(0.21-0.45) | 1881.56 | 3795.51 | 101.72% | 0.26(0.06-0.46) | 93669.28 | 103644.28 | 10.65% | -0.24(-0.42 - -0.07) |
| Mexico | UDSD | 4279815.76 | 8127844.96 | 89.91% | -0.077(-0.088 - -0.066) | 4427.58 | 5230.06 | 18.12% | -3.44(-3.69 - -3.19) | 198237.66 | 272890.9 | 37.66% | -1.92(-2.11 - -1.73) |
| Mexico | VAID | 11195.61 | 15167.32 | 35.48% | -2.14(-2.34 - -1.93) | 971.94 | 2603.5 | 167.87% | -0.45(-0.54 - -0.37) | 21464.18 | 47173.22 | 119.78% | -0.61(-0.73 - -0.49) |
| Micronesia (Federated States of) | Digestive diseases | 2301.79 | 2895.82 | 25.81% | -0.01(-0.03-0.01) | 38.75 | 33.84 | -12.67% | -1.77(-1.99 - -1.55) | 1496.01 | 1273.64 | -14.86% | -1.73(-1.93 - -1.54) |
| Micronesia (Federated States of) | APED | 109.88 | 129.87 | 18.19% | 0.41(0.35-0.47) | 0.55 | 0.35 | -36.36% | -2.31(-2.47 - -2.15) | 25.83 | 16.05 | -37.86% | -2.22(-2.36 - -2.07) |
| Micronesia (Federated States of) | COCLD | 17.35 | 18.69 | 7.72% | -0.45(-0.55 - -0.36) | 20.64 | 19.7 | -4.55% | -1.55(-1.79 - -1.32) | 800.16 | 724.13 | -9.50% | -1.63(-1.84 - -1.41) |
| Micronesia (Federated States of) | GABD | 116.86 | 165.75 | 41.84% | 0.33(0.3-0.37) | 2.21 | 2.37 | 7.24% | -0.73(-0.89 - -0.58) | 84.18 | 88.51 | 5.14% | -0.84(-0.96 - -0.71) |
| Micronesia (Federated States of) | IFAH | 62.13 | 88.31 | 42.14% | 0.51(0.37-0.65) | 0.57 | 0.46 | -19.30% | -1.71(-2.03 - -1.4) | 34.07 | 33.3 | -2.26% | -0.9(-1.15 - -0.65) |
| Micronesia (Federated States of) | IFBD | 0.38 | 0.64 | 68.42% | 1.12(1.03-1.21) | 0.68 | 0.53 | -22.06% | -2.09(-2.29 - -1.88) | 27.63 | 20.11 | -27.22% | -2.07(-2.24 - -1.89) |
| Micronesia (Federated States of) | PACA | 18.3 | 22.42 | 22.51% | -0.16(-0.18 - -0.15) | 1.25 | 1.29 | 3.20% | -1.11(-1.27 - -0.94) | 49.66 | 50.19 | 1.07% | -1.1(-1.23 - -0.96) |
| Micronesia (Federated States of) | PIIO | 43.43 | 55.92 | 28.77% | 0.85(0.77-0.92) | 1.59 | 1.42 | -10.57% | -1.26(-1.5 - -1.01) | 55.86 | 43.03 | -22.96% | -1.25(-1.47 - -1.03) |
| Micronesia (Federated States of) | UDSD | 1930.01 | 2408.84 | 24.81% | -0.085(-0.098 - -0.073) | 9.84 | 6.02 | -38.82% | -2.91(-3.12 - -2.69) | 371.03 | 244.97 | -33.98% | -2.65(-2.86 - -2.45) |
| Micronesia (Federated States of) | VAID | 3.45 | 5.38 | 55.94% | 1.02(0.93-1.1) | 0.34 | 0.54 | 58.82% | 0.29(0.2-0.38) | 8.81 | 13.97 | 58.57% | 0.35(0.24-0.47) |
| Monaco | Digestive diseases | 1881.8 | 2384.91 | 26.74% | 0.06(0.06-0.06) | 13.46 | 17.7 | 31.56% | -0.08(-0.2-0.04) | 321.45 | 390.25 | 21.40% | -0.19(-0.25 - -0.13) |
| Monaco | APED | 60.43 | 72.23 | 19.53% | 0.16(0.13-0.2) | 0.14 | 0.11 | -21.43% | -2.01(-2.2 - -1.83) | 2.96 | 2.46 | -16.89% | -1.06(-1.23 - -0.9) |
| Monaco | COCLD | 7.39 | 8.64 | 16.91% | 0.31(0.24-0.38) | 6.17 | 7.42 | 20.26% | -0.29(-0.36 - -0.22) | 150.78 | 174.32 | 15.61% | -0.32(-0.39 - -0.26) |
| Monaco | GABD | 191.12 | 261.22 | 36.68% | 0.24(0.22-0.26) | 0.98 | 1.42 | 44.90% | 0.2(0-0.39) | 26.92 | 34.22 | 27.12% | -0.06(-0.13-0) |
| Monaco | IFAH | 50.65 | 67.67 | 33.60% | 0.08(0.01-0.16) | 0.67 | 0.66 | -1.49% | -1.38(-1.52 - -1.25) | 17.71 | 18.4 | 3.90% | -0.71(-0.8 - -0.62) |
| Monaco | IFBD | 3.49 | 4.21 | 20.63% | 0.03(-0.02-0.07) | 0.29 | 0.4 | 37.93% | -0.07(-0.15-0.01) | 10.67 | 13.94 | 30.65% | 0.08(0.06-0.1) |
| Monaco | PACA | 9.58 | 11.87 | 23.90% | -0.1(-0.12 - -0.08) | 0.52 | 0.65 | 25.00% | -0.18(-0.28 - -0.09) | 9.94 | 11.8 | 18.71% | -0.19(-0.25 - -0.13) |
| Monaco | PIIO | 72.27 | 98.41 | 36.17% | 0.1(0.05-0.15) | 1.54 | 3.02 | 95.41% | 1.38(0.91-1.85) | 21.69 | 37.58 | 73.25% | 0.86(0.5-1.22) |
| Monaco | UDSD | 1472.68 | 1838.86 | 24.86% | 0.023(0.019-0.027) | 1.51 | 0.95 | -37.09% | -2.99(-3.28 - -2.7) | 55.41 | 53.95 | -2.63% | -0.64(-0.74 - -0.55) |
| Monaco | VAID | 14.19 | 21.81 | 53.70% | 0.57(0.55-0.59) | 1.21 | 1.72 | 42.15% | 0.09(0.01-0.18) | 17.96 | 23.84 | 32.74% | -0.02(-0.1-0.05) |
| Mongolia | Digestive diseases | 96570.67 | 184488.39 | 91.04% | -0.09(-0.12 - -0.07) | 1407.86 | 2184.97 | 55.20% | -0.59(-0.81 - -0.37) | 62302.6 | 79665.15 | 27.87% | -1.37(-1.51 - -1.22) |
| Mongolia | APED | 14479.64 | 11036.9 | -23.78% | -1.7(-2.12 - -1.29) | 61.37 | 24.56 | -59.98% | -5.31(-5.65 - -4.97) | 4396.96 | 1404.78 | -68.05% | -5.77(-6.11 - -5.43) |
| Mongolia | COCLD | 816.84 | 2950.78 | 261.24% | 1.88(1.69-2.06) | 832.64 | 1656.48 | 98.94% | 0.12(-0.18-0.41) | 29775.41 | 54154.65 | 81.88% | -0.51(-0.74 - -0.29) |
| Mongolia | GABD | 6628.18 | 14556.39 | 119.61% | 0.01(-0.03-0.05) | 51.24 | 41.52 | -18.97% | -3.69(-4.04 - -3.34) | 2434.17 | 2784.79 | 14.40% | -2.89(-3.13 - -2.65) |
| Mongolia | IFAH | 3918.58 | 5270.94 | 34.51% | -1.49(-1.93 - -1.06) | 40.5 | 5.28 | -86.96% | -9.44(-10.45 - -8.41) | 3746.19 | 1386.29 | -62.99% | -5.9(-6.65 - -5.15) |
| Mongolia | IFBD | 97.76 | 251.06 | 156.81% | 0.58(0.55-0.61) | 10.71 | 11.93 | 11.39% | -2.1(-2.26 - -1.94) | 690.26 | 813.99 | 17.93% | -1.88(-2 - -1.75) |
| Mongolia | PACA | 545.06 | 1194.07 | 119.07% | -0.08(-0.09 - -0.08) | 59.75 | 104.53 | 74.95% | -1.18(-1.34 - -1.02) | 1906.66 | 3676.09 | 92.80% | -1.29(-1.47 - -1.1) |
| Mongolia | PIIO | 807.2 | 1898.86 | 135.24% | 1.25(1.1-1.4) | 130.84 | 87.75 | -32.93% | -2.64(-2.8 - -2.49) | 8800.93 | 4436.58 | -49.59% | -3.61(-3.82 - -3.4) |
| Mongolia | UDSD | 69076.31 | 146893.81 | 112.65% | 0.055(0.05-0.06) | 148.84 | 192.44 | 29.29% | -1.13(-1.48 - -0.78) | 6333.82 | 8902.08 | 40.55% | -1.31(-1.51 - -1.1) |
| Mongolia | VAID | 201.1 | 435.58 | 116.60% | 1.34(1.18-1.51) | 6.53 | 10.61 | 62.48% | -0.65(-0.77 - -0.53) | 153.16 | 258.27 | 68.63% | -0.84(-0.97 - -0.71) |
| Montenegro | Digestive diseases | 36876.24 | 44781.09 | 21.44% | 0.09(0.07-0.11) | 103.54 | 152.72 | 47.51% | -0.08(-0.26-0.1) | 4505.6 | 5585.83 | 23.98% | -0.29(-0.42 - -0.15) |
| Montenegro | APED | 1327.71 | 1174.6 | -11.53% | 0.09(0.03-0.14) | 0.67 | 0.77 | 14.93% | -0.95(-1.22 - -0.69) | 38.34 | 33.79 | -11.87% | -0.82(-0.96 - -0.67) |
| Montenegro | COCLD | 67.96 | 66.61 | -1.99% | -0.04(-0.09-0) | 35.54 | 49.91 | 40.43% | -0.13(-0.3-0.04) | 1189.19 | 1486.2 | 24.98% | -0.34(-0.52 - -0.16) |
| Montenegro | GABD | 5786.46 | 7587.9 | 31.13% | 0.28(0.23-0.32) | 2.17 | 3.45 | 58.99% | 0.14(0.01-0.27) | 573.65 | 683.37 | 19.13% | -0.26(-0.29 - -0.23) |
| Montenegro | IFAH | 815.49 | 1162.74 | 42.58% | 1.18(1.03-1.34) | 1.22 | 1.74 | 42.62% | -0.47(-0.66 - -0.29) | 183.6 | 238.04 | 29.65% | 0.42(0.37-0.47) |
| Montenegro | IFBD | 58.91 | 66.72 | 13.26% | 0.34(0.24-0.43) | 1.74 | 2.6 | 49.43% | 0.27(0.08-0.46) | 191.11 | 221.2 | 15.74% | -0.08(-0.14 - -0.02) |
| Montenegro | PACA | 281.28 | 336.36 | 19.58% | -0.18(-0.2 - -0.16) | 10.35 | 13.73 | 32.66% | -0.22(-0.37 - -0.07) | 375.27 | 420.94 | 12.17% | -0.55(-0.71 - -0.38) |
| Montenegro | PIIO | 795.22 | 920.18 | 15.71% | -0.3(-0.48 - -0.12) | 13.17 | 17.51 | 32.92% | -0.69(-0.86 - -0.52) | 369.58 | 386.99 | 4.71% | -1.19(-1.36 - -1.03) |
| Montenegro | UDSD | 27580.67 | 33207.73 | 20.40% | 0.02(0.015-0.026) | 14.44 | 18.01 | 24.72% | -0.71(-1.1 - -0.31) | 1009.07 | 1173.5 | 16.30% | -0.39(-0.57 - -0.2) |
| Montenegro | VAID | 162.54 | 258.25 | 58.88% | 0.61(0.5-0.72) | 22.73 | 42.32 | 86.19% | 0.66(0.51-0.8) | 504.08 | 843.06 | 67.25% | 0.38(0.22-0.53) |
| Morocco | Digestive diseases | 1148794.91 | 2106244.39 | 83.34% | 0.08(0.07-0.09) | 4808.59 | 7422.37 | 54.36% | -1.13(-1.3 - -0.96) | 186919.11 | 244822.57 | 30.98% | -1.19(-1.26 - -1.12) |
| Morocco | APED | 55271.98 | 100696.49 | 82.18% | 0.96(0.89-1.03) | 142.98 | 120.02 | -16.06% | -2.78(-2.89 - -2.66) | 6617.7 | 4714.88 | -28.75% | -2.78(-2.87 - -2.69) |
| Morocco | COCLD | 3296.16 | 6917.49 | 109.87% | 0.26(0.12-0.4) | 2732.65 | 4551.27 | 66.55% | -0.99(-1.16 - -0.83) | 86746.77 | 120388.18 | 38.78% | -1.24(-1.33 - -1.15) |
| Morocco | GABD | 35452.01 | 83147.17 | 134.53% | 0.68(0.67-0.7) | 170.57 | 395.73 | 132.00% | 0.26(0.1-0.43) | 9050.51 | 16558.01 | 82.95% | -0.29(-0.36 - -0.23) |
| Morocco | IFAH | 11221.62 | 22662.22 | 101.95% | 1.05(0.98-1.11) | 40.66 | 56 | 37.73% | -1.02(-1.29 - -0.75) | 4913.5 | 6430 | 30.86% | -0.34(-0.41 - -0.27) |
| Morocco | IFBD | 300.45 | 598.6 | 99.23% | 0.32(0.26-0.39) | 30.77 | 59.02 | 91.81% | -0.17(-0.38-0.04) | 1438.07 | 2614.58 | 81.81% | -0.09(-0.21-0.02) |
| Morocco | PACA | 4882.94 | 9291.97 | 90.29% | -0.01(-0.02 - -0.01) | 130.42 | 251.17 | 92.59% | -0.43(-0.54 - -0.32) | 3688.88 | 6243.17 | 69.24% | -0.63(-0.69 - -0.58) |
| Morocco | PIIO | 12611.59 | 27569.37 | 118.60% | 1.07(0.99-1.16) | 400.23 | 534.34 | 33.51% | -0.62(-0.75 - -0.49) | 19939.66 | 14304.28 | -28.26% | -1.25(-1.32 - -1.17) |
| Morocco | UDSD | 1024519.51 | 1851794.82 | 80.75% | -0.013(-0.016 - -0.01) | 990.5 | 1037.18 | 4.71% | -2.49(-2.68 - -2.31) | 49809.95 | 63949.73 | 28.39% | -1.5(-1.58 - -1.43) |
| Morocco | VAID | 1238.66 | 3566.25 | 187.91% | 1.44(1.32-1.57) | 77.67 | 180.69 | 132.64% | -0.01(-0.19-0.18) | 1579.29 | 3492.03 | 121.11% | -0.04(-0.18-0.1) |
| Mozambique | Digestive diseases | 479015.72 | 1088353.02 | 127.21% | 0.17(0.16-0.19) | 3728.73 | 7033.94 | 88.64% | 0.13(0.04-0.21) | 162325.32 | 317225.59 | 95.43% | 0.26(0.14-0.38) |
| Mozambique | APED | 18683.06 | 63027.18 | 237.35% | 1.27(1.19-1.35) | 171.16 | 244.16 | 42.65% | -0.1(-0.33-0.12) | 10326.95 | 12981.72 | 25.71% | -0.49(-0.76 - -0.22) |
| Mozambique | COCLD | 1754.95 | 4206.29 | 139.68% | 0.27(0.11-0.43) | 1679.86 | 2546.56 | 51.59% | -0.7(-0.95 - -0.46) | 58485.44 | 93441.07 | 59.77% | -0.55(-0.82 - -0.29) |
| Mozambique | GABD | 3676.49 | 10512.81 | 185.95% | 1.2(1.15-1.26) | 216.62 | 522.67 | 141.28% | 1.28(1.08-1.47) | 7107.27 | 15910.41 | 123.86% | 1.17(0.94-1.4) |
| Mozambique | IFAH | 10242.99 | 27981.63 | 173.18% | 1.37(1.27-1.47) | 163.36 | 253.92 | 55.44% | 0.22(0.04-0.39) | 11937.31 | 17410.59 | 45.85% | 0.01(-0.15-0.17) |
| Mozambique | IFBD | 75.97 | 192 | 152.73% | 0.74(0.7-0.77) | 51.53 | 106.71 | 107.08% | 0.94(0.77-1.12) | 2566.77 | 4503.13 | 75.44% | 0.62(0.41-0.84) |
| Mozambique | PACA | 1798.88 | 3888.71 | 116.17% | 0.04(0-0.08) | 102.51 | 274.11 | 167.40% | 1.31(1.08-1.54) | 3698.58 | 10628.91 | 187.38% | 1.6(1.35-1.86) |
| Mozambique | PIIO | 8699.05 | 25128.13 | 188.86% | 1.08(1.01-1.14) | 593.18 | 1586.06 | 167.38% | 1.05(0.85-1.24) | 30311.32 | 85660.42 | 182.60% | 1.46(1.21-1.71) |
| Mozambique | UDSD | 433542.81 | 951693.08 | 119.52% | 0.078(0.068-0.088) | 430.4 | 822.48 | 91.10% | 0.34(0.14-0.54) | 26402.18 | 55384.56 | 109.77% | 0.48(0.32-0.64) |
| Mozambique | VAID | 541.53 | 1723.18 | 218.21% | 1.72(1.61-1.82) | 72.07 | 202.89 | 181.52% | 1.65(1.52-1.77) | 1954.19 | 5270.78 | 169.72% | 1.78(1.62-1.94) |
| Myanmar | Digestive diseases | 1086642.62 | 1781525.68 | 63.95% | 0.05(0.04-0.07) | 23138.93 | 25894.79 | 11.91% | -1.82(-1.91 - -1.73) | 1009900.67 | 1026865.77 | 1.68% | -1.87(-1.95 - -1.78) |
| Myanmar | APED | 99057.48 | 138735.61 | 40.06% | 0.36(0.22-0.5) | 278.24 | 116.11 | -58.27% | -4.2(-4.53 - -3.86) | 18676.12 | 7003.28 | -62.50% | -4.35(-4.65 - -4.04) |
| Myanmar | COCLD | 12459.92 | 19740.09 | 58.43% | -0.11(-0.16 - -0.07) | 14646.56 | 20361.32 | 39.02% | -1.07(-1.15 - -0.98) | 607942.25 | 786939.44 | 29.44% | -1.19(-1.28 - -1.11) |
| Myanmar | GABD | 54912.88 | 109370.53 | 99.17% | 0.62(0.59-0.65) | 327.77 | 429.93 | 31.17% | -1.14(-1.35 - -0.94) | 22271.71 | 26533.21 | 19.13% | -1.41(-1.55 - -1.26) |
| Myanmar | IFAH | 24020.65 | 48874.6 | 103.47% | 0.63(0.47-0.8) | 354.04 | 161.61 | -54.35% | -3.21(-3.42 - -3) | 31711.52 | 16656.06 | -47.48% | -2.91(-3.07 - -2.75) |
| Myanmar | IFBD | 141.32 | 306.41 | 116.82% | 1.22(1.15-1.3) | 59.71 | 59.3 | -0.69% | -1.95(-2.07 - -1.83) | 2819.97 | 2257.54 | -19.94% | -2.29(-2.41 - -2.17) |
| Myanmar | PACA | 7054.6 | 11496.87 | 62.97% | -0.17(-0.18 - -0.16) | 316.21 | 364.26 | 15.20% | -1.5(-1.64 - -1.37) | 13570.1 | 13962.06 | 2.89% | -1.79(-1.92 - -1.66) |
| Myanmar | PIIO | 23252.73 | 49173.14 | 111.47% | 1.2(1.07-1.33) | 1104.69 | 938.67 | -15.03% | -1.08(-1.26 - -0.9) | 76108.44 | 40217.71 | -47.16% | -2.11(-2.34 - -1.88) |
| Myanmar | UDSD | 864644.79 | 1400993.2 | 62.03% | -0.065(-0.08 - -0.05) | 5771.03 | 3076.45 | -46.69% | -4.38(-4.57 - -4.2) | 223914.46 | 121404.17 | -45.78% | -4.18(-4.32 - -4.04) |
| Myanmar | VAID | 1098.25 | 2835.23 | 158.16% | 1.64(1.5-1.77) | 60.23 | 127.09 | 111.01% | 0.34(0.19-0.48) | 1709.1 | 2910.75 | 70.31% | -0.14(-0.27-0) |
| Namibia | Digestive diseases | 58912.83 | 119279.91 | 102.47% | 0.11(0.09-0.14) | 469.9 | 729.16 | 55.17% | -0.97(-1.19 - -0.75) | 17242.92 | 26669.35 | 54.67% | -0.95(-1.16 - -0.74) |
| Namibia | APED | 3933.46 | 7459.83 | 89.65% | 0.44(0.32-0.56) | 8.94 | 7.52 | -15.88% | -2.79(-3.1 - -2.47) | 499.25 | 440.67 | -11.73% | -2.52(-2.76 - -2.27) |
| Namibia | COCLD | 236.42 | 492.64 | 108.37% | 0.03(-0.07-0.14) | 219.82 | 339.04 | 54.24% | -1.06(-1.29 - -0.84) | 7192.26 | 11242.92 | 56.32% | -1.07(-1.32 - -0.83) |
| Namibia | GABD | 960.37 | 2179.69 | 126.96% | 0.7(0.65-0.75) | 28.27 | 51.59 | 82.49% | -0.28(-0.48 - -0.08) | 914.69 | 1531.35 | 67.42% | -0.57(-0.78 - -0.36) |
| Namibia | IFAH | 2433.7 | 4928.76 | 102.52% | 0.72(0.62-0.82) | 8.93 | 14.17 | 58.68% | -0.69(-0.88 - -0.5) | 1083.23 | 1654.19 | 52.71% | -0.44(-0.51 - -0.36) |
| Namibia | IFBD | 12.71 | 27.39 | 115.50% | 0.32(0.24-0.41) | 5.53 | 8.96 | 62.03% | -0.61(-0.8 - -0.41) | 218.41 | 333.38 | 52.64% | -0.52(-0.63 - -0.41) |
| Namibia | PACA | 205.83 | 405.5 | 97.01% | 0.01(-0.02-0.05) | 11.16 | 20.55 | 84.14% | -0.51(-0.78 - -0.23) | 376.03 | 723.21 | 92.33% | -0.45(-0.77 - -0.12) |
| Namibia | PIIO | 1414.01 | 3774.7 | 166.95% | 1.46(1.33-1.59) | 69.4 | 126.85 | 82.79% | -0.37(-0.52 - -0.22) | 2272.94 | 3987.74 | 75.44% | -0.33(-0.46 - -0.2) |
| Namibia | UDSD | 49632.84 | 99807.19 | 101.09% | 0.016(0.006-0.025) | 80.1 | 100.97 | 26.05% | -1.55(-1.87 - -1.22) | 3471.61 | 5014.26 | 44.44% | -1.19(-1.44 - -0.94) |
| Namibia | VAID | 83.49 | 204.21 | 144.59% | 1.36(1.19-1.53) | 6.36 | 14.53 | 128.46% | 0.43(0.28-0.59) | 168.51 | 364.31 | 116.19% | 0.32(0.14-0.5) |
| Nauru | Digestive diseases | 232.01 | 263.16 | 13.43% | 0.05(0.02-0.07) | 3.02 | 2.38 | -21.16% | -1.11(-1.33 - -0.88) | 131.66 | 108.92 | -17.27% | -1.11(-1.34 - -0.89) |
| Nauru | APED | 11.11 | 14.16 | 27.45% | 0.43(0.32-0.55) | 0.04 | 0.03 | -25.00% | -0.99(-1.37 - -0.61) | 1.93 | 1.51 | -21.76% | -0.98(-1.41 - -0.54) |
| Nauru | COCLD | 1.55 | 1.81 | 16.77% | 0.08(0.01-0.14) | 1.84 | 1.52 | -17.39% | -1.13(-1.29 - -0.96) | 77.53 | 64.36 | -16.99% | -1.19(-1.36 - -1.02) |
| Nauru | GABD | 12.36 | 15.12 | 22.33% | 0.33(0.29-0.37) | 0.15 | 0.14 | -6.67% | -0.2(-0.38 - -0.01) | 6.62 | 6.91 | 4.38% | -0.18(-0.4-0.04) |
| Nauru | IFAH | 7.98 | 8.58 | 7.52% | 0.18(-0.02-0.37) | 0.03 | 0.02 | -33.33% | -0.29(-0.5 - -0.07) | 2.88 | 2.66 | -7.64% | -0.18(-0.4-0.03) |
| Nauru | IFBD | 0.04 | 0.06 | 50.00% | 1.18(1.03-1.33) | 0.07 | 0.04 | -42.86% | -1.85(-2.09 - -1.61) | 3.22 | 2.12 | -34.16% | -1.87(-2.08 - -1.66) |
| Nauru | PACA | 1.71 | 1.86 | 8.77% | -0.06(-0.07 - -0.05) | 0.11 | 0.1 | -9.09% | -0.62(-0.86 - -0.37) | 4.78 | 4.72 | -1.26% | -0.59(-0.81 - -0.37) |
| Nauru | PIIO | 6.36 | 6.66 | 4.78% | 0.14(-0.15-0.43) | 0.1 | 0.08 | -15.75% | -0.09(-0.39-0.21) | 4.41 | 3.87 | -12.34% | -0.16(-0.49-0.16) |
| Nauru | UDSD | 190.49 | 214.43 | 12.57% | 0.001(-0.006-0.008) | 0.58 | 0.34 | -41.38% | -1.88(-2.25 - -1.5) | 26.44 | 18.75 | -29.08% | -1.68(-2.05 - -1.31) |
| Nauru | VAID | 0.42 | 0.49 | 16.67% | 0.45(0.13-0.77) | 0.03 | 0.03 | 0.00% | 0.07(-0.13-0.26) | 0.91 | 1.05 | 15.38% | 0.14(-0.05-0.34) |
| Nepal | Digestive diseases | 1041481.03 | 1943789.29 | 86.64% | -0.13(-0.16 - -0.1) | 8275.88 | 11323.32 | 36.82% | -1.44(-1.62 - -1.26) | 356340.15 | 403133 | 13.13% | -1.75(-1.94 - -1.57) |
| Nepal | APED | 183007.74 | 259903.62 | 42.02% | -1.06(-1.27 - -0.86) | 463.04 | 269.62 | -41.77% | -3.99(-4.31 - -3.67) | 24378.25 | 12968.53 | -46.80% | -4.26(-4.59 - -3.93) |
| Nepal | COCLD | 3745.77 | 7106.79 | 89.73% | 0.53(0.48-0.58) | 5458.67 | 7945.38 | 45.56% | -1.19(-1.35 - -1.03) | 213575.36 | 253449.44 | 18.67% | -1.65(-1.82 - -1.47) |
| Nepal | GABD | 9609.53 | 22891.07 | 138.21% | 1.64(1.31-1.96) | 182.03 | 223.18 | 22.61% | -1.9(-2.22 - -1.58) | 7784.8 | 8050.37 | 3.41% | -1.96(-2.21 - -1.7) |
| Nepal | IFAH | 25934.19 | 47759.32 | 84.16% | 0.07(0.02-0.12) | 283.82 | 223.51 | -21.25% | -3.54(-3.83 - -3.25) | 17857.36 | 16201.89 | -9.27% | -2.47(-2.67 - -2.27) |
| Nepal | IFBD | 242.04 | 624.25 | 157.91% | 1.03(0.96-1.09) | 93.29 | 114.84 | 23.10% | -1.88(-2.06 - -1.69) | 3779.64 | 3819.28 | 1.05% | -2.15(-2.36 - -1.94) |
| Nepal | PACA | 3746.45 | 6949.07 | 85.48% | 0(-0.03-0.03) | 353.95 | 531.3 | 50.11% | -1.09(-1.32 - -0.86) | 13295.72 | 16402.39 | 23.37% | -1.62(-1.87 - -1.36) |
| Nepal | PIIO | 30158.78 | 58138.76 | 92.78% | 0.06(-0.07-0.19) | 1028.87 | 1335.09 | 29.76% | -1.45(-1.57 - -1.33) | 45008.72 | 41642.96 | -7.48% | -1.98(-2.1 - -1.86) |
| Nepal | UDSD | 784348.61 | 1538290.5 | 96.12% | -0.028(-0.039 - -0.016) | 236.27 | 240.63 | 1.85% | -3.06(-3.36 - -2.76) | 25596.63 | 41219.45 | 61.03% | -1(-1.13 - -0.86) |
| Nepal | VAID | 687.92 | 2125.9 | 209.03% | 1.53(1.45-1.62) | 123.16 | 343.14 | 178.61% | 0.16(0.02-0.29) | 2909.73 | 6470.38 | 122.37% | -0.31(-0.45 - -0.17) |
| Netherlands | Digestive diseases | 650836.13 | 848001.39 | 30.29% | 0.05(0.04-0.05) | 4863.05 | 6477.55 | 33.20% | -1.47(-1.76 - -1.19) | 111872.6 | 127785.25 | 14.22% | -1.42(-1.66 - -1.18) |
| Netherlands | APED | 37940.77 | 39047.59 | 2.92% | 0.14(0.12-0.16) | 44.44 | 44.15 | -0.65% | -2.56(-3 - -2.12) | 1400.67 | 1182.76 | -15.56% | -1.73(-1.96 - -1.49) |
| Netherlands | COCLD | 2751.74 | 2786.76 | 1.27% | -0.39(-0.55 - -0.23) | 1454.34 | 1810.37 | 24.48% | -1.63(-1.96 - -1.3) | 34710.74 | 36730.01 | 5.82% | -1.89(-2.24 - -1.55) |
| Netherlands | GABD | 76153.48 | 107837.73 | 41.61% | 0.03(0.01-0.05) | 400.33 | 576.5 | 44.01% | -0.78(-0.89 - -0.68) | 11241.04 | 14030.37 | 24.81% | -0.77(-0.81 - -0.73) |
| Netherlands | IFAH | 17967.82 | 26392.77 | 46.89% | 0.31(0.27-0.34) | 122.89 | 167.94 | 36.66% | -1.22(-1.33 - -1.1) | 5020.4 | 6210.57 | 23.71% | -0.59(-0.64 - -0.54) |
| Netherlands | IFBD | 2179.53 | 1460.01 | -33.01% | -1.79(-2.43 - -1.15) | 278.47 | 781.56 | 180.66% | 1.35(0.87-1.84) | 8234.04 | 12100.22 | 46.95% | -0.5(-0.93 - -0.06) |
| Netherlands | PACA | 1666.87 | 2259.01 | 35.52% | -0.19(-0.24 - -0.14) | 188 | 269.12 | 43.15% | -1.18(-1.55 - -0.8) | 4483.19 | 5412.07 | 20.72% | -1.41(-1.74 - -1.07) |
| Netherlands | PIIO | 23765.49 | 39597.8 | 66.62% | 0.43(0.38-0.49) | 550.53 | 1022.13 | 85.66% | -0.62(-1.07 - -0.17) | 8137.52 | 12761 | 56.82% | -0.84(-1.25 - -0.43) |
| Netherlands | UDSD | 483938.01 | 620739.53 | 28.27% | 0.019(0.013-0.024) | 946.37 | 388.63 | -58.93% | -6.12(-6.49 - -5.76) | 24551.49 | 18848.24 | -23.23% | -2.19(-2.35 - -2.04) |
| Netherlands | VAID | 4472.42 | 7880.18 | 76.19% | 0.35(0.28-0.42) | 431.73 | 620.18 | 43.65% | -1.12(-1.42 - -0.83) | 7065.12 | 9609.4 | 36.01% | -1.11(-1.4 - -0.82) |
| New Zealand | Digestive diseases | 211023.1 | 327482.63 | 55.19% | 0.2(0.18-0.22) | 750 | 1088.08 | 45.08% | -1.48(-1.64 - -1.31) | 22288.23 | 29726.68 | 33.37% | -0.86(-0.95 - -0.78) |
| New Zealand | APED | 10475.61 | 11856.36 | 13.18% | 0.01(-0.04-0.07) | 5.54 | 7.81 | 40.97% | -1.48(-1.67 - -1.29) | 263.46 | 282.2 | 7.11% | -0.93(-1.03 - -0.82) |
| New Zealand | COCLD | 318.87 | 438.16 | 37.41% | -0.14(-0.19 - -0.1) | 192.26 | 297.22 | 54.59% | -1.05(-1.11 - -0.98) | 5053.38 | 6737.21 | 33.32% | -1.2(-1.26 - -1.15) |
| New Zealand | GABD | 25662.61 | 57507.1 | 124.09% | 1.44(1.19-1.68) | 39.38 | 91.4 | 132.10% | 0.24(0.09-0.4) | 2501.02 | 4943.98 | 97.68% | 0.74(0.52-0.96) |
| New Zealand | IFAH | 6940.93 | 10378.21 | 49.52% | -0.43(-0.49 - -0.37) | 20.97 | 40.81 | 94.61% | -0.63(-0.81 - -0.46) | 1305.99 | 1770.98 | 35.60% | -0.97(-1.1 - -0.84) |
| New Zealand | IFBD | 766.13 | 1092.3 | 42.57% | 0.26(0.21-0.31) | 19.86 | 31.4 | 58.11% | -1.7(-2.05 - -1.34) | 1266.53 | 1758.47 | 38.84% | -0.32(-0.42 - -0.22) |
| New Zealand | PACA | 1237.07 | 2243.59 | 81.36% | 0.28(0.24-0.33) | 36.5 | 59.04 | 61.75% | -0.86(-1.13 - -0.59) | 909.91 | 1234.12 | 35.63% | -0.99(-1.24 - -0.74) |
| New Zealand | PIIO | 8206.92 | 13826.25 | 68.47% | -0.15(-0.16 - -0.14) | 73.71 | 164.44 | 123.08% | -0.06(-0.23-0.11) | 1293.92 | 2191.67 | 69.38% | -0.39(-0.56 - -0.21) |
| New Zealand | UDSD | 155877.46 | 228343.53 | 46.49% | -0.001(-0.023-0.02) | 212.6 | 109.93 | -48.29% | -5.52(-5.96 - -5.09) | 7076.84 | 6745.08 | -4.69% | -1.64(-1.9 - -1.39) |
| New Zealand | VAID | 1537.51 | 1797.14 | 16.89% | -0.91(-1.43 - -0.38) | 75.28 | 112.5 | 49.44% | -1.49(-1.8 - -1.17) | 1300.66 | 1608.62 | 23.68% | -1.83(-2.16 - -1.49) |
| Nicaragua | Digestive diseases | 215249.78 | 486365.12 | 125.95% | 0.08(0.06-0.09) | 848.77 | 2236.56 | 163.51% | 0.25(0.08-0.42) | 41376.67 | 81117.47 | 96.05% | -0.21(-0.31 - -0.1) |
| Nicaragua | APED | 17227.55 | 28985.18 | 68.25% | 0.51(0.42-0.61) | 35.27 | 35.41 | 0.40% | -2.06(-2.23 - -1.89) | 2241.55 | 1665.84 | -25.68% | -2.47(-2.73 - -2.2) |
| Nicaragua | COCLD | 838.75 | 2395.86 | 185.65% | 1.05(0.84-1.26) | 495.72 | 1623.15 | 227.43% | 0.87(0.73-1.01) | 17567.8 | 47647.06 | 171.22% | 0.51(0.36-0.67) |
| Nicaragua | GABD | 20072.05 | 51949.71 | 158.82% | 0.49(0.43-0.54) | 34.71 | 85.73 | 146.99% | -0.34(-0.62 - -0.06) | 3583.83 | 6950.92 | 93.95% | -0.88(-0.99 - -0.78) |
| Nicaragua | IFAH | 7141.52 | 14581.62 | 104.18% | 0.5(0.39-0.61) | 29.69 | 41.81 | 40.82% | -1(-1.31 - -0.69) | 3345.74 | 3952.74 | 18.14% | -1.11(-1.19 - -1.04) |
| Nicaragua | IFBD | 60.3 | 124.37 | 106.25% | -0.44(-0.52 - -0.36) | 2.69 | 7.46 | 177.32% | 0.48(0.3-0.67) | 186.79 | 443.79 | 137.59% | 0.12(-0.02-0.25) |
| Nicaragua | PACA | 992.92 | 2088.96 | 110.39% | -0.22(-0.23 - -0.2) | 28.17 | 67.6 | 139.97% | -0.33(-0.52 - -0.13) | 1064.83 | 2089.15 | 96.20% | -0.78(-0.92 - -0.64) |
| Nicaragua | PIIO | 4350.98 | 7837.72 | 80.14% | 0.79(0.57-1.01) | 84.82 | 121.37 | 43.09% | -0.01(-0.37-0.34) | 5481.22 | 4081.04 | -25.54% | -1.25(-1.32 - -1.18) |
| Nicaragua | UDSD | 164385.49 | 377905.71 | 129.89% | -0.033(-0.035 - -0.031) | 108.65 | 143.41 | 31.99% | -2.77(-3.18 - -2.36) | 6597.7 | 11318.15 | 71.55% | -1.55(-1.66 - -1.44) |
| Nicaragua | VAID | 180.22 | 495.97 | 175.20% | 1.38(1.24-1.53) | 10 | 38.37 | 283.70% | 1.19(0.76-1.62) | 228.65 | 757.29 | 231.20% | 0.76(0.49-1.03) |
| Niger | Digestive diseases | 265459.91 | 760209.98 | 186.37% | 0.07(0.04-0.09) | 3052.15 | 6498.69 | 112.92% | -0.76(-0.84 - -0.67) | 148428.25 | 299124.2 | 101.53% | -0.88(-0.98 - -0.78) |
| Niger | APED | 9858.81 | 36103.64 | 266.21% | 0.71(0.61-0.82) | 91.6 | 92.72 | 1.22% | -2.7(-3 - -2.4) | 6344.86 | 5706.23 | -10.07% | -3.7(-4.07 - -3.32) |
| Niger | COCLD | 1939.03 | 5635.98 | 190.66% | 0.12(0.06-0.18) | 1650.26 | 3324.85 | 101.47% | -1.22(-1.38 - -1.07) | 65293.59 | 128488.91 | 96.79% | -1.22(-1.37 - -1.07) |
| Niger | GABD | 2151.21 | 7455.4 | 246.57% | 0.97(0.94-1) | 80.8 | 234.17 | 189.81% | 0.77(0.7-0.85) | 4408.8 | 10282.07 | 133.22% | 0.16(0.08-0.25) |
| Niger | IFAH | 10222.16 | 37898.01 | 270.74% | 1.5(1.42-1.58) | 66.83 | 154.61 | 131.35% | 0.05(-0.05-0.15) | 6748.02 | 16863.28 | 149.90% | 0.25(0.18-0.31) |
| Niger | IFBD | 42.43 | 122.43 | 188.55% | 0.02(-0.1-0.15) | 26.9 | 55.41 | 105.99% | -0.71(-0.92 - -0.51) | 1564.4 | 2834.79 | 81.21% | -1.08(-1.3 - -0.86) |
| Niger | PACA | 1204.78 | 3441.5 | 185.65% | 0.09(0.08-0.11) | 125.5 | 361.33 | 187.91% | 0.28(0.16-0.39) | 5213.58 | 14241.99 | 173.17% | 0.2(0.07-0.33) |
| Niger | PIIO | 4875.68 | 17282.54 | 254.46% | 0.95(0.89-1.01) | 393.27 | 1001.56 | 154.68% | 0.15(0.04-0.27) | 22816.87 | 51238.81 | 124.57% | -0.24(-0.4 - -0.09) |
| Niger | UDSD | 234799 | 651127.23 | 177.31% | -0.032(-0.056 - -0.009) | 429.83 | 936.21 | 117.81% | -0.31(-0.42 - -0.2) | 25570.53 | 53718.12 | 110.08% | -0.56(-0.67 - -0.45) |
| Niger | VAID | 366.8 | 1143.24 | 211.68% | 0.76(0.7-0.83) | 33.32 | 77.81 | 133.52% | 0.55(0.47-0.62) | 1887 | 3103.59 | 64.47% | -0.42(-0.58 - -0.26) |
| Nigeria | Digestive diseases | 3502539.89 | 8358235 | 138.63% | 0.07(0.06-0.08) | 43375.11 | 70076.79 | 61.56% | -0.48(-0.65 - -0.32) | 1749358.14 | 2977406.59 | 70.20% | -0.67(-0.79 - -0.55) |
| Nigeria | APED | 46217.56 | 223056.15 | 382.62% | 2.72(2.12-3.32) | 785.7 | 661.57 | -15.80% | -2.87(-3.04 - -2.71) | 46749.77 | 39720.23 | -15.04% | -3.52(-3.75 - -3.3) |
| Nigeria | COCLD | 17729.51 | 47955.3 | 170.48% | 0.48(0.39-0.57) | 25713.12 | 40380.62 | 57.04% | -0.64(-0.83 - -0.45) | 881039.49 | 1460194.71 | 65.74% | -0.75(-0.92 - -0.59) |
| Nigeria | GABD | 38332.8 | 108056.74 | 181.89% | 0.74(0.71-0.77) | 1129.92 | 2499.53 | 121.21% | 1.08(0.82-1.35) | 43521.61 | 88202.27 | 102.66% | 0.5(0.3-0.7) |
| Nigeria | IFAH | 161334.78 | 371061.9 | 129.99% | -0.07(-0.16-0.01) | 764.17 | 1308.04 | 71.17% | 0.12(-0.07-0.3) | 64654.18 | 116992.02 | 80.95% | -0.51(-0.61 - -0.4) |
| Nigeria | IFBD | 634.4 | 1712.89 | 170.00% | 0.44(0.27-0.61) | 313.64 | 559.95 | 78.53% | -0.11(-0.21 - -0.02) | 15139.26 | 26793.54 | 76.98% | -0.43(-0.51 - -0.35) |
| Nigeria | PACA | 17216.12 | 41313.18 | 139.97% | 0.14(0.14-0.15) | 2059.99 | 3882.05 | 88.45% | -0.21(-0.31 - -0.11) | 78083.76 | 148822.18 | 90.59% | -0.45(-0.55 - -0.36) |
| Nigeria | PIIO | 75208.13 | 190504.69 | 153.30% | 0.69(0.6-0.79) | 4991.05 | 10358.3 | 107.54% | 0.48(0.36-0.6) | 251533.24 | 524678.71 | 108.59% | 0.27(0.18-0.36) |
| Nigeria | UDSD | 3139221.06 | 7357084.58 | 134.36% | 0.014(0.009-0.02) | 5197.44 | 6926.66 | 33.27% | -0.98(-1.17 - -0.79) | 265953.72 | 433199.78 | 62.89% | -0.93(-1.04 - -0.83) |
| Nigeria | VAID | 6645.53 | 17489.56 | 163.18% | 0.65(0.61-0.69) | 412.38 | 870.08 | 110.99% | 1.04(0.82-1.27) | 16335.03 | 28539.85 | 74.72% | 0.3(0.14-0.46) |
| Niue | Digestive diseases | 64.46 | 57.76 | -10.39% | 0.06(0.05-0.07) | 1.08 | 0.69 | -35.87% | -1.46(-1.52 - -1.4) | 30.87 | 19.67 | -36.28% | -1.52(-1.59 - -1.45) |
| Niue | APED | 2.29 | 1.98 | -13.54% | 0.62(0.58-0.66) | 0.01 | 0 | -100.00% | -2.01(-2.08 - -1.93) | 0.31 | 0.15 | -51.61% | -1.9(-1.99 - -1.81) |
| Niue | COCLD | 0.29 | 0.25 | -13.79% | -0.12(-0.24-0.01) | 0.55 | 0.37 | -32.73% | -1.44(-1.52 - -1.37) | 17.08 | 10.82 | -36.65% | -1.69(-1.77 - -1.6) |
| Niue | GABD | 3.88 | 3.68 | -5.15% | 0.16(0.15-0.18) | 0.08 | 0.06 | -25.00% | -0.47(-0.53 - -0.41) | 1.99 | 1.61 | -19.10% | -0.53(-0.58 - -0.48) |
| Niue | IFAH | 1.71 | 1.82 | 6.43% | 0.77(0.71-0.84) | 0.02 | 0.01 | -50.00% | -1.13(-1.16 - -1.09) | 0.74 | 0.58 | -21.62% | -0.34(-0.39 - -0.3) |
| Niue | IFBD | 0.01 | 0.01 | 0.00% | 1.36(1.24-1.48) | 0.02 | 0.01 | -50.00% | -1.75(-1.86 - -1.63) | 0.65 | 0.35 | -46.15% | -1.92(-2.07 - -1.78) |
| Niue | PACA | 0.57 | 0.5 | -12.28% | -0.03(-0.04 - -0.02) | 0.03 | 0.03 | 0.00% | -0.93(-1.01 - -0.85) | 1.03 | 0.74 | -28.16% | -1.03(-1.13 - -0.93) |
| Niue | PIIO | 2.11 | 2.2 | 4.39% | 0.97(0.91-1.04) | 0.06 | 0.05 | -27.53% | -0.46(-0.5 - -0.43) | 1.35 | 0.93 | -30.93% | -0.51(-0.56 - -0.46) |
| Niue | UDSD | 53.47 | 47.14 | -11.84% | -0.037(-0.047 - -0.027) | 0.25 | 0.11 | -56.00% | -2.74(-2.85 - -2.62) | 6.54 | 3.41 | -47.86% | -2.18(-2.28 - -2.07) |
| Niue | VAID | 0.14 | 0.17 | 21.43% | 1.25(1.19-1.32) | 0.02 | 0.02 | 0.00% | 0.32(0.21-0.43) | 0.35 | 0.36 | 2.86% | 0.44(0.31-0.58) |
| North Macedonia | Digestive diseases | 114532.84 | 155560.69 | 35.82% | 0.09(0.08-0.1) | 419.5 | 593.46 | 41.47% | -0.59(-0.7 - -0.47) | 17341.24 | 21135.01 | 21.88% | -0.8(-0.87 - -0.74) |
| North Macedonia | APED | 4169.85 | 3966.3 | -4.88% | 0.17(0.12-0.22) | 3.11 | 1.99 | -36.01% | -3.33(-3.59 - -3.07) | 144.04 | 93.51 | -35.08% | -2.08(-2.31 - -1.85) |
| North Macedonia | COCLD | 353.39 | 500.16 | 41.53% | 0.57(0.48-0.66) | 197.86 | 316.64 | 60.03% | -0.15(-0.28 - -0.02) | 6321.36 | 8846.12 | 39.94% | -0.49(-0.61 - -0.36) |
| North Macedonia | GABD | 17399.9 | 26146.66 | 50.27% | 0.27(0.23-0.31) | 13.34 | 16.46 | 23.39% | -1.05(-1.22 - -0.87) | 2139.92 | 2523.11 | 17.91% | -0.81(-0.87 - -0.75) |
| North Macedonia | IFAH | 2716.54 | 4133.14 | 52.15% | 0.76(0.68-0.84) | 14.99 | 11.68 | -22.08% | -2.86(-3.1 - -2.62) | 1042.77 | 968.78 | -7.10% | -1.24(-1.44 - -1.04) |
| North Macedonia | IFBD | 193.53 | 253.27 | 30.87% | 0.32(0.26-0.38) | 3.55 | 4.87 | 37.18% | -0.21(-0.36 - -0.07) | 490.13 | 634.95 | 29.55% | 0(-0.04-0.05) |
| North Macedonia | PACA | 858.29 | 1162.02 | 35.39% | -0.1(-0.12 - -0.08) | 30 | 49.38 | 64.60% | 0.2(-0.02-0.42) | 993.62 | 1345.75 | 35.44% | -0.41(-0.6 - -0.22) |
| North Macedonia | PIIO | 1651.23 | 2688.18 | 62.80% | 0.5(0.43-0.57) | 43.53 | 82.63 | 89.83% | 1.09(0.86-1.32) | 1378.9 | 1679.99 | 21.84% | -0.19(-0.38 - -0.01) |
| North Macedonia | UDSD | 86921.28 | 116316.09 | 33.82% | 0.018(0.012-0.024) | 72.83 | 57.8 | -20.64% | -2.93(-3.18 - -2.68) | 3718.12 | 3865.18 | 3.96% | -1.41(-1.51 - -1.3) |
| North Macedonia | VAID | 268.84 | 394.86 | 46.88% | 0.23(0.22-0.25) | 30.45 | 40.25 | 32.18% | -1.27(-1.54 - -1.01) | 697.55 | 804.51 | 15.33% | -1.82(-2.12 - -1.53) |
| Northern Mariana Islands | Digestive diseases | 1271.13 | 1605.28 | 26.29% | 0.07(0.05-0.09) | 13.32 | 19.09 | 43.31% | -1.62(-1.74 - -1.5) | 574.7 | 626.34 | 8.99% | -1.6(-1.73 - -1.48) |
| Northern Mariana Islands | APED | 51.28 | 49.36 | -3.74% | 0.48(0.42-0.54) | 0.05 | 0.03 | -40.00% | -3.56(-4.02 - -3.09) | 2.93 | 1.59 | -45.73% | -2.6(-3 - -2.19) |
| Northern Mariana Islands | COCLD | 17.41 | 14.2 | -18.44% | -0.78(-0.94 - -0.61) | 10.22 | 12.8 | 25.24% | -2.03(-2.2 - -1.86) | 423.92 | 409.74 | -3.34% | -2.12(-2.28 - -1.95) |
| Northern Mariana Islands | GABD | 76.91 | 123.2 | 60.19% | 0.29(0.21-0.38) | 0.65 | 1.4 | 115.38% | -0.71(-0.89 - -0.53) | 28 | 44.15 | 57.68% | -0.59(-0.75 - -0.42) |
| Northern Mariana Islands | IFAH | 30.29 | 51.23 | 69.13% | 0.47(0.32-0.62) | 0.03 | 0.07 | 133.33% | -1.77(-2.1 - -1.44) | 6.99 | 10.63 | 52.07% | -0.1(-0.28-0.07) |
| Northern Mariana Islands | IFBD | 0.26 | 0.38 | 46.15% | 0.79(0.69-0.9) | 0.28 | 0.15 | -46.43% | -4.76(-5.32 - -4.19) | 12.07 | 5.13 | -57.50% | -4.28(-4.78 - -3.78) |
| Northern Mariana Islands | PACA | 9.46 | 12.8 | 35.31% | 0.02(0.01-0.04) | 0.21 | 0.6 | 185.71% | 1.3(0.97-1.62) | 8.87 | 19.7 | 122.10% | 1.85(1.43-2.28) |
| Northern Mariana Islands | PIIO | 40.69 | 60.91 | 49.67% | 0.01(-0.18-0.21) | 0.39 | 0.96 | 143.77% | -0.38(-0.62 - -0.14) | 15.31 | 22.81 | 48.98% | -0.34(-0.63 - -0.04) |
| Northern Mariana Islands | UDSD | 1041.92 | 1286.75 | 23.50% | 0.031(0.025-0.037) | 0.99 | 1.37 | 38.38% | -2.64(-2.72 - -2.56) | 58.64 | 67.06 | 14.36% | -1.62(-1.67 - -1.56) |
| Northern Mariana Islands | VAID | 2.91 | 6.46 | 121.99% | 0.16(0.03-0.28) | 0.15 | 0.39 | 160.00% | -0.84(-0.99 - -0.69) | 4.63 | 9.69 | 109.29% | -0.66(-0.84 - -0.47) |
| Norway | Digestive diseases | 231565.9 | 315709.01 | 36.34% | 0.4(0.29-0.52) | 1391.24 | 1408.64 | 1.25% | -1.47(-1.56 - -1.37) | 38285.8 | 40340.06 | 5.37% | -1.01(-1.06 - -0.96) |
| Norway | APED | 11474.59 | 14786.29 | 28.86% | 0.33(0.28-0.38) | 12.76 | 10.62 | -16.77% | -1.95(-2.13 - -1.77) | 377.27 | 332.87 | -11.77% | -1.07(-1.17 - -0.98) |
| Norway | COCLD | 701.02 | 835.03 | 19.12% | 0.65(0.35-0.95) | 367.4 | 360.26 | -1.94% | -1.52(-1.63 - -1.4) | 9408.05 | 8302.03 | -11.76% | -1.81(-1.95 - -1.67) |
| Norway | GABD | 63270.35 | 88870.64 | 40.46% | 1.3(0.88-1.72) | 179.23 | 161.69 | -9.79% | -1.72(-1.96 - -1.49) | 7282.14 | 8129.76 | 11.64% | -0.55(-0.64 - -0.47) |
| Norway | IFAH | 10450.98 | 13645.92 | 30.57% | -0.29(-0.4 - -0.18) | 60.25 | 49.11 | -18.49% | -1.75(-2.2 - -1.31) | 2299.03 | 2301.64 | 0.11% | -1.24(-1.47 - -1) |
| Norway | IFBD | 1361.58 | 2113.09 | 55.19% | 0.79(0.67-0.91) | 29.2 | 38.15 | 30.65% | -0.86(-1.19 - -0.53) | 3551.39 | 5413.71 | 52.44% | 0.17(0.01-0.33) |
| Norway | PACA | 1599.31 | 2857.18 | 78.65% | 0.25(-0.05-0.55) | 66.23 | 69.71 | 5.25% | -1.38(-1.54 - -1.22) | 1478.25 | 1550.84 | 4.91% | -1.31(-1.47 - -1.16) |
| Norway | PIIO | 11592.06 | 16062.65 | 38.57% | 0.37(0.23-0.5) | 153.01 | 188.81 | 23.40% | -0.72(-0.77 - -0.67) | 2265.85 | 2490.27 | 9.90% | -0.89(-0.95 - -0.84) |
| Norway | UDSD | 129142.07 | 174322.32 | 34.98% | 0.005(-0.005-0.014) | 306.97 | 215.67 | -29.74% | -2.96(-3.21 - -2.7) | 8074.35 | 7297.77 | -9.62% | -1.42(-1.49 - -1.35) |
| Norway | VAID | 1973.93 | 2215.89 | 12.26% | -1.01(-1.19 - -0.82) | 117.58 | 121.35 | 3.21% | -1.59(-1.78 - -1.4) | 1823.24 | 1723.44 | -5.47% | -1.78(-1.97 - -1.6) |
| Oman | Digestive diseases | 81837.89 | 267975.79 | 227.45% | 0.07(0.05-0.09) | 223.53 | 349.67 | 56.43% | -0.73(-0.98 - -0.49) | 9737.39 | 18293.38 | 87.87% | -0.97(-1.17 - -0.77) |
| Oman | APED | 4006.78 | 14752.73 | 268.19% | 1.31(1.24-1.37) | 2.14 | 2.52 | 17.76% | -0.47(-1.12-0.18) | 125.61 | 254.77 | 102.83% | -0.39(-0.76 - -0.01) |
| Oman | COCLD | 317.79 | 1036.46 | 226.15% | 0.54(0.44-0.63) | 164.63 | 278.68 | 69.28% | -0.57(-0.81 - -0.34) | 5562.05 | 9193.86 | 65.30% | -1.02(-1.29 - -0.74) |
| Oman | GABD | 2352.03 | 8091.97 | 244.04% | 0.24(0.1-0.37) | 4.17 | 6.64 | 59.23% | -0.36(-0.59 - -0.14) | 344.27 | 777.92 | 125.96% | -0.84(-0.92 - -0.76) |
| Oman | IFAH | 1263.86 | 3493.85 | 176.44% | 0.7(0.63-0.77) | 1.35 | 1.71 | 26.67% | -0.76(-1.05 - -0.47) | 342.25 | 694.67 | 102.97% | -0.36(-0.45 - -0.28) |
| Oman | IFBD | 38.42 | 142.81 | 271.71% | 0.06(-0.11-0.22) | 1.58 | 3.73 | 136.08% | 0.58(0.34-0.82) | 122.48 | 375.34 | 206.45% | 0.17(0-0.35) |
| Oman | PACA | 314.33 | 930.79 | 196.12% | -0.09(-0.11 - -0.07) | 3.56 | 5.9 | 65.73% | -0.75(-0.93 - -0.56) | 120.89 | 228.9 | 89.35% | -1.05(-1.21 - -0.9) |
| Oman | PIIO | 1474.75 | 5180.39 | 251.27% | 1.28(1.11-1.46) | 9.54 | 15.33 | 60.72% | 0.09(-0.41-0.59) | 418.46 | 578.42 | 38.22% | -0.17(-0.63-0.29) |
| Oman | UDSD | 71953.18 | 233888.09 | 225.06% | -0.04(-0.056 - -0.023) | 30.64 | 22.64 | -26.11% | -2.52(-2.94 - -2.09) | 2502.99 | 5782.98 | 131.04% | -1.31(-1.46 - -1.17) |
| Oman | VAID | 116.74 | 458.71 | 292.93% | 1.53(1.44-1.63) | 3.19 | 6.77 | 112.23% | 0.33(0.12-0.54) | 75.28 | 164.7 | 118.78% | 0.03(-0.1-0.17) |
| Pakistan | Digestive diseases | 5040334.69 | 11117192.6 | 120.56% | 0(-0.03-0.03) | 40424.85 | 62350.96 | 54.24% | -0.9(-1.01 - -0.79) | 1558922.16 | 2668882.99 | 71.20% | -0.86(-0.96 - -0.75) |
| Pakistan | APED | 97754.68 | 386437.87 | 295.31% | 2.36(1.68-3.04) | 1621.85 | 2083.58 | 28.47% | -1.76(-1.96 - -1.56) | 73111.95 | 103133.75 | 41.06% | -1.72(-1.91 - -1.52) |
| Pakistan | COCLD | 24805.74 | 50880.21 | 105.11% | -0.01(-0.04-0.03) | 25978.94 | 44070.18 | 69.64% | -0.64(-0.76 - -0.52) | 932501.27 | 1699392.32 | 82.24% | -0.66(-0.8 - -0.53) |
| Pakistan | GABD | 117751.39 | 279911.8 | 137.71% | 0.5(0.47-0.53) | 875.44 | 1040.46 | 18.85% | -1.21(-1.24 - -1.17) | 38740.21 | 56906.2 | 46.89% | -1.19(-1.22 - -1.15) |
| Pakistan | IFAH | 196988.18 | 330361.1 | 67.71% | -1.19(-1.36 - -1.02) | 1110.58 | 765.67 | -31.06% | -3.4(-3.52 - -3.27) | 71428.11 | 80702.94 | 12.98% | -2.6(-2.76 - -2.43) |
| Pakistan | IFBD | 1474.39 | 3887.63 | 163.68% | 0.69(0.65-0.74) | 453.8 | 657.29 | 44.84% | -0.87(-0.93 - -0.8) | 16234.03 | 27286.26 | 68.08% | -0.71(-0.77 - -0.66) |
| Pakistan | PACA | 30082.71 | 68167.49 | 126.60% | 0.24(0.21-0.27) | 1406.26 | 2479.01 | 76.28% | -0.42(-0.57 - -0.27) | 48914.97 | 94460.12 | 93.11% | -0.48(-0.64 - -0.33) |
| Pakistan | PIIO | 60854.84 | 135551.73 | 122.75% | 0.48(0.45-0.51) | 3892.58 | 5324.64 | 36.79% | -1.1(-1.13 - -1.07) | 140650.45 | 215447.05 | 53.18% | -1.02(-1.05 - -0.99) |
| Pakistan | UDSD | 4503270.54 | 9846093.53 | 118.64% | -0.023(-0.035 - -0.012) | 4099.28 | 4298.98 | 4.87% | -1.88(-2.08 - -1.69) | 212945.02 | 349273.6 | 64.02% | -1.13(-1.23 - -1.04) |
| Pakistan | VAID | 7352.23 | 15901.24 | 116.28% | 0.4(0.29-0.51) | 725.33 | 1285.34 | 77.21% | 0.41(0.32-0.51) | 14546.2 | 27854.01 | 91.49% | 0.26(0.17-0.35) |
| Palau | Digestive diseases | 426.09 | 674.28 | 58.25% | 0.04(0.04-0.05) | 4.04 | 6.1 | 50.84% | -0.91(-0.98 - -0.84) | 150 | 217.77 | 45.18% | -0.76(-0.83 - -0.69) |
| Palau | APED | 17.19 | 22.61 | 31.53% | 0.56(0.49-0.62) | 0.04 | 0.04 | 0.00% | -1.16(-1.24 - -1.08) | 1.69 | 1.84 | 8.88% | -0.7(-0.81 - -0.59) |
| Palau | COCLD | 2.39 | 3.6 | 50.63% | 0.11(-0.01-0.23) | 2.45 | 3.81 | 55.51% | -0.95(-1.03 - -0.86) | 89.26 | 131.07 | 46.84% | -0.88(-0.96 - -0.8) |
| Palau | GABD | 23.61 | 42.54 | 80.18% | 0.13(0.11-0.16) | 0.26 | 0.44 | 69.23% | -0.27(-0.32 - -0.22) | 9.23 | 15.4 | 66.85% | -0.21(-0.28 - -0.15) |
| Palau | IFAH | 10.14 | 21.17 | 108.78% | 0.71(0.63-0.79) | 0.06 | 0.08 | 33.33% | -1.11(-1.24 - -0.97) | 3.76 | 5.85 | 55.59% | -0.22(-0.29 - -0.15) |
| Palau | IFBD | 0.08 | 0.17 | 112.50% | 1.11(1.02-1.2) | 0.09 | 0.11 | 22.22% | -1.36(-1.47 - -1.24) | 3.35 | 3.85 | 14.93% | -1.21(-1.31 - -1.1) |
| Palau | PACA | 3.31 | 5.54 | 67.37% | 0.01(-0.01-0.02) | 0.15 | 0.25 | 66.67% | -0.27(-0.31 - -0.22) | 5.56 | 9.06 | 62.95% | -0.03(-0.08-0.02) |
| Palau | PIIO | 12.42 | 21.42 | 72.44% | 0.4(0.33-0.48) | 0.14 | 0.2 | 40.76% | -0.67(-0.75 - -0.59) | 4.35 | 5.12 | 17.62% | -0.6(-0.67 - -0.53) |
| Palau | UDSD | 356.16 | 555.44 | 55.95% | -0.027(-0.034 - -0.02) | 0.68 | 0.81 | 19.12% | -1.54(-1.65 - -1.43) | 27.67 | 35.52 | 28.37% | -1.07(-1.16 - -0.97) |
| Palau | VAID | 0.79 | 1.8 | 127.85% | 0.96(0.86-1.06) | 0.05 | 0.13 | 160.00% | 0.57(0.45-0.68) | 1.34 | 3.22 | 140.30% | 0.71(0.57-0.84) |
| Palestine | Digestive diseases | 79813.88 | 237328.35 | 197.35% | 0.07(0.05-0.09) | 318.95 | 522.36 | 63.78% | -1.17(-1.31 - -1.03) | 12097.66 | 20951.08 | 73.18% | -1.12(-1.25 - -0.99) |
| Palestine | APED | 4606.71 | 15708.21 | 240.99% | 0.95(0.83-1.08) | 8.41 | 8.52 | 1.31% | -2.76(-2.88 - -2.65) | 372 | 461.57 | 24.08% | -2.43(-2.57 - -2.29) |
| Palestine | COCLD | 272.44 | 821.19 | 201.42% | 0.42(0.12-0.72) | 206.43 | 358.66 | 73.74% | -1.06(-1.22 - -0.91) | 6165.87 | 10036.88 | 62.78% | -1.26(-1.43 - -1.1) |
| Palestine | GABD | 2767.57 | 8631.4 | 211.88% | 0.15(0.05-0.24) | 14.34 | 27.62 | 92.61% | -0.51(-0.64 - -0.39) | 592.11 | 1332.62 | 125.06% | -0.71(-0.81 - -0.62) |
| Palestine | IFAH | 1196.67 | 3289.37 | 174.88% | 0.97(0.88-1.05) | 2.66 | 3.08 | 15.79% | -1.5(-1.62 - -1.38) | 430.55 | 831.68 | 93.17% | -0.1(-0.22-0.01) |
| Palestine | IFBD | 28.9 | 122.09 | 322.46% | 1.11(0.92-1.3) | 2.74 | 4.11 | 50.00% | -2.83(-3.48 - -2.18) | 132.33 | 320.48 | 142.18% | -1.16(-1.42 - -0.9) |
| Palestine | PACA | 358.49 | 983.34 | 174.30% | -0.11(-0.13 - -0.09) | 12.55 | 22.91 | 82.55% | -0.97(-1.08 - -0.87) | 311.69 | 604.26 | 93.87% | -1.21(-1.32 - -1.1) |
| Palestine | PIIO | 1508.95 | 3951.41 | 161.87% | 0.4(0.24-0.55) | 33.09 | 39.04 | 17.96% | -1.46(-1.56 - -1.35) | 1582.58 | 1395.99 | -11.79% | -1.76(-1.87 - -1.65) |
| Palestine | UDSD | 68976.38 | 203458.99 | 194.97% | 0.004(-0.002-0.01) | 25.52 | 29.17 | 14.30% | -2.49(-2.79 - -2.2) | 2126.18 | 5166.49 | 142.99% | -0.74(-0.84 - -0.65) |
| Palestine | VAID | 97.79 | 362.34 | 270.53% | 1.22(1.05-1.39) | 6.65 | 16.05 | 141.35% | 0.12(-0.04-0.28) | 135.43 | 329.02 | 142.94% | -0.08(-0.21-0.06) |
| Panama | Digestive diseases | 165365.89 | 333760.98 | 101.83% | -0.03(-0.05 - -0.01) | 455.45 | 934.84 | 105.26% | -0.75(-0.9 - -0.6) | 21104.48 | 35161.27 | 66.61% | -0.71(-0.89 - -0.54) |
| Panama | APED | 11466.27 | 17580.87 | 53.33% | -0.29(-0.43 - -0.15) | 20.17 | 14.65 | -27.37% | -3.17(-3.74 - -2.59) | 1308.45 | 795.65 | -39.19% | -3.11(-3.11 - -2.53) |
| Panama | COCLD | 425.66 | 949.95 | 123.17% | 0.17(0.03-0.3) | 212.5 | 502.87 | 136.64% | -0.23(-0.39 - -0.07) | 6467.88 | 12929.29 | 99.90% | -0.34(-0.54 - -0.15) |
| Panama | GABD | 16476.16 | 35392.98 | 114.81% | 0.05(0-0.09) | 34.02 | 49.61 | 45.83% | -2.36(-2.61 - -2.1) | 2609.28 | 4313.75 | 65.32% | -1.07(-1.22 - -0.92) |
| Panama | IFAH | 4978.01 | 10380.45 | 108.53% | -0.05(-0.18-0.09) | 15.38 | 17.85 | 16.06% | -2.68(-2.89 - -2.47) | 1898.34 | 2458.97 | 29.53% | -1.36(-1.51 - -1.21) |
| Panama | IFBD | 50.19 | 92.49 | 84.28% | -0.63(-0.82 - -0.43) | 7.3 | 24.81 | 239.86% | 2.2(1.67-2.73) | 435.73 | 1002.06 | 129.97% | 1.57(1.1-2.05) |
| Panama | PACA | 719.65 | 1469.66 | 104.22% | -0.1(-0.13 - -0.07) | 14.9 | 35.3 | 136.91% | -0.22(-0.48-0.05) | 515.97 | 1067.14 | 106.82% | -0.09(-0.4-0.22) |
| Panama | PIIO | 3546.38 | 6586.25 | 85.72% | -0.26(-0.39 - -0.14) | 53.68 | 110.42 | 105.70% | -0.2(-0.38 - -0.03) | 2484.08 | 3081.25 | 24.04% | -0.63(-0.98 - -0.28) |
| Panama | UDSD | 127485.49 | 260649.86 | 104.45% | -0.02(-0.022 - -0.017) | 59.51 | 57.17 | -3.93% | -3.98(-4.13 - -3.84) | 4202.08 | 6839.74 | 62.77% | -1.13(-1.21 - -1.04) |
| Panama | VAID | 218.08 | 658.47 | 201.94% | 0.63(0.54-0.72) | 20.92 | 57.84 | 176.48% | -0.44(-0.61 - -0.26) | 446.53 | 1022.31 | 128.95% | -0.57(-0.76 - -0.38) |
| Papua New Guinea | Digestive diseases | 92630.55 | 242926.76 | 162.25% | 0.04(0.02-0.06) | 629.07 | 1368.39 | 117.53% | -0.46(-0.5 - -0.42) | 30518.82 | 66276.07 | 117.16% | -0.48(-0.52 - -0.45) |
| Papua New Guinea | APED | 3858.78 | 11088.65 | 187.36% | 0.47(0.34-0.6) | 11.51 | 22.23 | 93.14% | -0.7(-0.8 - -0.6) | 615.52 | 1196.12 | 94.33% | -0.7(-0.81 - -0.6) |
| Papua New Guinea | COCLD | 236.53 | 617.35 | 161.00% | -0.06(-0.11 - -0.01) | 279.34 | 660.45 | 136.43% | -0.28(-0.31 - -0.24) | 11841.71 | 27531.38 | 132.49% | -0.39(-0.42 - -0.35) |
| Papua New Guinea | GABD | 3563.33 | 10259.64 | 187.92% | 0.37(0.34-0.39) | 35.87 | 101.93 | 184.17% | 0.58(0.47-0.7) | 1860.68 | 5018.37 | 169.71% | 0.31(0.22-0.4) |
| Papua New Guinea | IFAH | 2043.24 | 6787.19 | 232.18% | 0.84(0.75-0.94) | 16.55 | 32.28 | 95.05% | -0.16(-0.27 - -0.04) | 1547.98 | 3395.49 | 119.35% | 0.03(-0.04-0.1) |
| Papua New Guinea | IFBD | 13.07 | 43.44 | 232.36% | 0.93(0.81-1.05) | 12.92 | 25.31 | 95.90% | -1.01(-1.1 - -0.93) | 660.79 | 1258.15 | 90.40% | -1.09(-1.18 - -1) |
| Papua New Guinea | PACA | 657.39 | 1680.22 | 155.59% | -0.08(-0.09 - -0.07) | 19.64 | 49.55 | 152.29% | -0.03(-0.08-0.02) | 850.6 | 2133.82 | 150.86% | -0.11(-0.15 - -0.06) |
| Papua New Guinea | PIIO | 1505.08 | 3881.94 | 157.92% | 0.08(0.02-0.14) | 39.27 | 89.64 | 128.26% | 0.13(0.06-0.2) | 2580.05 | 5502.63 | 113.28% | 0.03(-0.07-0.12) |
| Papua New Guinea | UDSD | 80643.78 | 208244.97 | 158.23% | -0.01(-0.022-0.002) | 193.83 | 330.96 | 70.75% | -1.19(-1.24 - -1.13) | 9697.07 | 17948.13 | 85.09% | -1.04(-1.08 - -0.99) |
| Papua New Guinea | VAID | 109.35 | 323.35 | 195.70% | 0.57(0.46-0.68) | 4.46 | 14.56 | 226.46% | 0.84(0.73-0.94) | 144.38 | 478.86 | 231.67% | 0.94(0.82-1.06) |
| Paraguay | Digestive diseases | 256239.32 | 530559.1 | 107.06% | -0.1(-0.12 - -0.08) | 724.45 | 1668.04 | 130.25% | 0.29(0.21-0.38) | 36464 | 64496.94 | 76.88% | -0.17(-0.28 - -0.06) |
| Paraguay | APED | 13416.26 | 16904.12 | 26.00% | -1.54(-1.75 - -1.33) | 35.02 | 37.88 | 8.17% | -0.73(-1.42 - -0.04) | 2148.48 | 1594.81 | -25.77% | -1.79(-2.41 - -1.16) |
| Paraguay | COCLD | 471.04 | 1062.89 | 125.65% | 0.12(0.04-0.21) | 300.91 | 749.69 | 149.14% | 0.13(0-0.26) | 10207.03 | 22716.2 | 122.55% | 0.06(-0.09-0.21) |
| Paraguay | GABD | 17662.26 | 43233.77 | 144.78% | 0.47(0.43-0.51) | 56.25 | 132.32 | 135.24% | 0.15(-0.16-0.46) | 3617.51 | 7296.15 | 101.69% | -0.24(-0.41 - -0.07) |
| Paraguay | IFAH | 8321.81 | 17448.17 | 109.67% | 0.12(-0.01-0.26) | 47.1 | 72.62 | 54.18% | -0.27(-0.5 - -0.03) | 4460.34 | 5377.93 | 20.57% | -0.92(-1.12 - -0.71) |
| Paraguay | IFBD | 108.6 | 222.25 | 104.65% | -0.6(-1.02 - -0.17) | 4.29 | 11.63 | 171.10% | 1.36(1.08-1.63) | 327.24 | 647.15 | 97.76% | 0.12(-0.06-0.29) |
| Paraguay | PACA | 507.32 | 1188.59 | 134.29% | 0.24(0.2-0.28) | 39.97 | 112.19 | 180.69% | 1.04(0.78-1.3) | 1288.14 | 3238.13 | 151.38% | 0.77(0.51-1.03) |
| Paraguay | PIIO | 6262.3 | 9126.69 | 45.74% | -0.86(-0.99 - -0.72) | 84.23 | 208.63 | 147.70% | 2.11(1.71-2.51) | 4830.3 | 5626.35 | 16.48% | 0.59(0.3-0.89) |
| Paraguay | UDSD | 209281.17 | 440796.38 | 110.62% | -0.083(-0.098 - -0.069) | 92.72 | 126.73 | 36.68% | -2.18(-2.39 - -1.97) | 7554.39 | 13077.2 | 73.11% | -0.94(-0.98 - -0.89) |
| Paraguay | VAID | 208.57 | 576.24 | 176.28% | 1(0.96-1.05) | 32.65 | 97.22 | 197.76% | 0.85(0.71-0.99) | 663.52 | 1850.06 | 178.83% | 0.83(0.67-0.98) |
| Peru | Digestive diseases | 1674676.51 | 2824548.22 | 68.66% | -0.38(-0.47 - -0.29) | 10789.94 | 11648.94 | 7.96% | -2.37(-2.57 - -2.17) | 602440.2 | 394879.29 | -34.45% | -3.31(-3.65 - -2.96) |
| Peru | APED | 401891.58 | 304376.07 | -24.26% | -2.81(-3.37 - -2.24) | 1264.47 | 184.44 | -85.41% | -7.96(-9.03 - -6.87) | 97078.58 | 11187.58 | -88.48% | -8.7(-9.76 - -7.62) |
| Peru | COCLD | 4297.47 | 10145.25 | 136.07% | 0.69(0.48-0.89) | 4065.78 | 6828.28 | 67.95% | -1.44(-1.61 - -1.28) | 144448.91 | 182835.4 | 26.57% | -1.98(-2.16 - -1.8) |
| Peru | GABD | 52264.7 | 84579.63 | 61.83% | -1.23(-1.52 - -0.93) | 588.3 | 761.98 | 29.52% | -2.52(-2.78 - -2.25) | 24737.31 | 23448.88 | -5.21% | -3.13(-3.45 - -2.82) |
| Peru | IFAH | 59850.54 | 98070.29 | 63.86% | -0.01(-0.15-0.13) | 467.23 | 160.53 | -65.64% | -5.37(-5.95 - -4.79) | 52929.29 | 25538.36 | -51.75% | -3.79(-4.28 - -3.29) |
| Peru | IFBD | 273.69 | 608.29 | 122.26% | 0(-0.22-0.22) | 127.64 | 76.23 | -40.28% | -4.2(-4.68 - -3.72) | 8234.1 | 3118.03 | -62.13% | -5.08(-5.69 - -4.47) |
| Peru | PACA | 7463.83 | 14065.77 | 88.45% | -0.41(-0.42 - -0.39) | 525.27 | 639.9 | 21.82% | -2.23(-2.52 - -1.93) | 21566.86 | 19872.95 | -7.85% | -2.74(-3.07 - -2.4) |
| Peru | PIIO | 24665.86 | 51330.51 | 108.10% | 0.62(0.55-0.7) | 2347.98 | 1327.98 | -43.44% | -4.22(-4.53 - -3.91) | 161527.32 | 36990.25 | -77.10% | -6.68(-7.26 - -6.1) |
| Peru | UDSD | 1123101.32 | 2259005.88 | 101.14% | 0.052(0.042-0.062) | 834.96 | 1064.94 | 27.54% | -2.29(-2.5 - -2.09) | 54333.07 | 75968.24 | 39.82% | -1.26(-1.37 - -1.15) |
| Peru | VAID | 867.52 | 2366.53 | 172.79% | 0.75(0.69-0.81) | 128.09 | 214.87 | 67.75% | -1.85(-2.13 - -1.58) | 4032.16 | 3732.23 | -7.44% | -2.84(-3.21 - -2.47) |
| Philippines | Digestive diseases | 1608146.2 | 3489350.99 | 116.98% | 0.12(0.08-0.16) | 18284.21 | 27944.71 | 52.84% | -2.08(-2.24 - -1.91) | 677706.84 | 1013153.28 | 49.50% | -1.7(-1.85 - -1.56) |
| Philippines | APED | 42881.19 | 127151.08 | 196.52% | 2.07(1.48-2.65) | 303.09 | 367.71 | 21.32% | -1.42(-1.6 - -1.24) | 17660.65 | 19948.19 | 12.95% | -1.21(-1.43 - -0.99) |
| Philippines | COCLD | 8344.52 | 21019.08 | 151.89% | 0.84(0.72-0.96) | 7843.58 | 14286.91 | 82.15% | -1.14(-1.32 - -0.96) | 286216.75 | 479215.9 | 67.43% | -1.32(-1.47 - -1.17) |
| Philippines | GABD | 138126.55 | 364361.56 | 163.79% | 0.94(0.79-1.1) | 589.41 | 1174.3 | 99.23% | -0.85(-1.08 - -0.61) | 32636.3 | 65551.05 | 100.85% | -0.41(-0.6 - -0.23) |
| Philippines | IFAH | 62318.66 | 151411.15 | 142.96% | 0.19(0.12-0.27) | 226.98 | 397.23 | 75.01% | -1.24(-1.44 - -1.04) | 17601.01 | 32969.95 | 87.32% | -0.6(-0.74 - -0.47) |
| Philippines | IFBD | 242.31 | 656.6 | 170.98% | 0.79(0.68-0.89) | 98.16 | 117.38 | 19.58% | -3.25(-3.54 - -2.95) | 3743.12 | 4555.8 | 21.71% | -2.61(-2.86 - -2.35) |
| Philippines | PACA | 9350.01 | 19310.12 | 106.53% | -0.28(-0.36 - -0.21) | 396.77 | 765.97 | 93.05% | -0.87(-1.01 - -0.73) | 16045.04 | 28716.79 | 78.98% | -0.99(-1.11 - -0.86) |
| Philippines | PIIO | 27712.69 | 63175.43 | 127.97% | 0.46(0.44-0.47) | 1011.88 | 2086.05 | 106.16% | -0.56(-0.82 - -0.3) | 38811.72 | 75902.17 | 95.57% | -0.09(-0.34-0.17) |
| Philippines | UDSD | 1316534.3 | 2736314.13 | 107.84% | -0.045(-0.061 - -0.03) | 7412.75 | 7870.11 | 6.17% | -3.64(-3.82 - -3.47) | 250823.18 | 279693.44 | 11.51% | -2.9(-3.03 - -2.77) |
| Philippines | VAID | 2635.97 | 5951.86 | 125.79% | 0.28(0.19-0.37) | 94.02 | 239.29 | 154.51% | -0.2(-0.5-0.11) | 2178.77 | 5067.5 | 132.59% | -0.31(-0.56 - -0.05) |
| Poland | Digestive diseases | 3066741.51 | 3584449.02 | 16.88% | -0.19(-0.26 - -0.12) | 14466.76 | 18145.45 | 25.43% | -0.82(-0.97 - -0.67) | 513143.22 | 579379.54 | 12.91% | -0.63(-0.77 - -0.48) |
| Poland | APED | 66976.61 | 57580.05 | -14.03% | -0.33(-0.52 - -0.14) | 208.64 | 98.27 | -52.90% | -4.47(-4.77 - -4.17) | 5997.49 | 2603.71 | -56.59% | -3.44(-3.74 - -3.15) |
| Poland | COCLD | 9550.29 | 8644.37 | -9.49% | -0.43(-0.76 - -0.09) | 6450.07 | 8564.83 | 32.79% | -0.25(-0.49 - -0.01) | 184375.12 | 264518.37 | 43.47% | 0.32(0-0.64) |
| Poland | GABD | 401232.93 | 442219.51 | 10.22% | -0.88(-1.4 - -0.36) | 1153.66 | 935.47 | -18.91% | -3.25(-3.61 - -2.9) | 51166.71 | 40530.93 | -20.79% | -2.26(-2.48 - -2.03) |
| Poland | IFAH | 103505.68 | 81839.32 | -20.93% | -1.36(-1.68 - -1.04) | 866.64 | 352.59 | -59.32% | -5.96(-6.49 - -5.43) | 31573.43 | 13781.27 | -56.35% | -4.01(-4.25 - -3.76) |
| Poland | IFBD | 14031.43 | 5805.16 | -58.63% | -3.85(-4.56 - -3.13) | 180.49 | 360.95 | 99.98% | 0.82(0.68-0.96) | 30297.02 | 20081.31 | -33.72% | -2.7(-3.28 - -2.11) |
| Poland | PACA | 21054.31 | 21951.23 | 4.26% | -1(-1.19 - -0.82) | 1415.99 | 2002.7 | 41.43% | -0.39(-0.73 - -0.04) | 45988.36 | 56016.22 | 21.81% | -0.42(-0.72 - -0.11) |
| Poland | PIIO | 42333.5 | 43516.96 | 2.80% | -1.01(-1.37 - -0.65) | 983.42 | 1169.8 | 18.95% | -1.74(-1.94 - -1.55) | 23814.82 | 19958.66 | -16.19% | -2.31(-2.4 - -2.23) |
| Poland | UDSD | 2401580.1 | 2915913.55 | 21.42% | 0.002(-0.013-0.018) | 2501.75 | 2480.27 | -0.86% | -1.72(-1.92 - -1.51) | 122016.55 | 123127.7 | 0.91% | -0.78(-0.91 - -0.65) |
| Poland | VAID | 6476.65 | 6978.88 | 7.75% | -1.21(-1.63 - -0.78) | 421.43 | 1509.62 | 258.21% | 2.03(0.89-3.18) | 8334.11 | 23918.27 | 186.99% | 1.6(0.56-2.65) |
| Portugal | Digestive diseases | 487819.75 | 633458.05 | 29.85% | 0.16(0.14-0.17) | 5560.4 | 5433.29 | -2.29% | -2.57(-2.69 - -2.45) | 155874.71 | 117540.25 | -24.59% | -2.67(-2.78 - -2.56) |
| Portugal | APED | 14903.04 | 14762.7 | -0.94% | 0.77(0.68-0.86) | 25.91 | 27.61 | 6.56% | -2(-2.35 - -1.66) | 886.34 | 645.79 | -27.14% | -1.86(-2.13 - -1.59) |
| Portugal | COCLD | 3889.63 | 2238.94 | -42.44% | -2.79(-2.96 - -2.62) | 3658.59 | 2091.65 | -42.83% | -3.84(-3.98 - -3.7) | 105573.42 | 52938.49 | -49.86% | -3.97(-4.1 - -3.84) |
| Portugal | GABD | 18054.47 | 35581.93 | 97.08% | 1.6(1.37-1.82) | 204.95 | 505.03 | 146.42% | 0.34(0.17-0.51) | 4867.61 | 8593.21 | 76.54% | 0.34(0.16-0.53) |
| Portugal | IFAH | 8389.95 | 15580.27 | 85.70% | 1.62(1.35-1.88) | 117.1 | 154.47 | 31.91% | -1.75(-2.41 - -1.07) | 3898.85 | 4568.32 | 17.17% | -0.62(-1.1 - -0.13) |
| Portugal | IFBD | 607.14 | 1112.32 | 83.21% | 1.51(1.22-1.8) | 81.02 | 305.56 | 277.14% | 2.38(1.99-2.77) | 2424.94 | 5331.73 | 119.87% | 1.24(1.07-1.41) |
| Portugal | PACA | 1805.22 | 2719.82 | 50.66% | 0.12(-0.04-0.29) | 243.98 | 353.74 | 44.99% | -1.67(-1.94 - -1.4) | 6086.93 | 6287.03 | 3.29% | -2.1(-2.33 - -1.86) |
| Portugal | PIIO | 9814.19 | 21392.2 | 117.97% | 1.27(1.13-1.41) | 237.11 | 481.47 | 103.06% | -0.33(-0.58 - -0.08) | 4579.37 | 6432.64 | 40.47% | -1.15(-1.37 - -0.93) |
| Portugal | UDSD | 428094.42 | 534139.91 | 24.77% | 0.007(0.004-0.011) | 612.19 | 329.07 | -46.25% | -5.95(-6.48 - -5.41) | 20662.4 | 16673.06 | -19.31% | -2.02(-2.25 - -1.8) |
| Portugal | VAID | 2261.69 | 5929.96 | 162.19% | 1.44(1.15-1.72) | 270.53 | 855.54 | 216.25% | 1.16(0.84-1.48) | 4602.18 | 11165.42 | 142.61% | 0.8(0.51-1.08) |
| Puer- Rico | Digestive diseases | 267753.15 | 332460.42 | 24.17% | 0.1(0.1-0.11) | 1561.96 | 1653.6 | 5.87% | -2.78(-3.06 - -2.5) | 52462.67 | 45646.54 | -12.99% | -2.42(-2.67 - -2.17) |
| Puer- Rico | APED | 8468.77 | 9352.96 | 10.44% | 1.1(1.07-1.14) | 12.56 | 8.05 | -35.91% | -3.19(-3.73 - -2.65) | 540.6 | 299.67 | -44.57% | -1.9(-2.33 - -1.47) |
| Puer- Rico | COCLD | 1216.42 | 948.13 | -22.06% | -1.56(-1.71 - -1.4) | 1066.48 | 1018.83 | -4.47% | -2.99(-3.33 - -2.65) | 32008.04 | 24290.61 | -24.11% | -3.33(-3.69 - -2.97) |
| Puer- Rico | GABD | 13088.45 | 16285.8 | 24.43% | 0(-0.04-0.04) | 75.02 | 88.37 | 17.80% | -2.49(-2.72 - -2.25) | 2827.53 | 2812.11 | -0.55% | -1.6(-1.76 - -1.44) |
| Puer- Rico | IFAH | 7752.49 | 9535.05 | 22.99% | 0.52(0.49-0.56) | 22.33 | 24.71 | 10.66% | -2.94(-3.4 - -2.47) | 2291.94 | 2195.74 | -4.20% | -0.71(-0.86 - -0.57) |
| Puer- Rico | IFBD | 86.55 | 122.07 | 41.04% | 0.48(0.33-0.63) | 19.81 | 17.91 | -9.59% | -3.7(-4.28 - -3.1) | 755.25 | 591.88 | -21.63% | -2.69(-3.09 - -2.29) |
| Puer- Rico | PACA | 1192.34 | 1489.2 | 24.90% | -0.31(-0.33 - -0.28) | 88.39 | 102.39 | 15.84% | -2.46(-2.78 - -2.14) | 2357.03 | 2200.09 | -6.66% | -2.33(-2.6 - -2.05) |
| Puer- Rico | PIIO | 4800.39 | 8389.9 | 74.78% | 1.16(1.07-1.25) | 79.13 | 146.2 | 84.76% | -0.8(-0.89 - -0.71) | 2119.41 | 2471.6 | 16.62% | -1.01(-1.16 - -0.87) |
| Puer- Rico | UDSD | 230683 | 285596.2 | 23.80% | 0.033(0.03-0.036) | 127.95 | 55.08 | -56.95% | -6.52(-7.11 - -5.93) | 8003.3 | 7737.39 | -3.32% | -1.15(-1.33 - -0.97) |
| Puer- Rico | VAID | 464.74 | 741.11 | 59.47% | 0.03(-0.03-0.09) | 39.61 | 51.94 | 31.13% | -2.67(-2.93 - -2.41) | 785.87 | 828.47 | 5.42% | -2.79(-3.07 - -2.5) |
| Qatar | Digestive diseases | 22992.3 | 183871.88 | 699.71% | 0.15(0.14-0.16) | 47.62 | 220.83 | 363.78% | -0.03(-0.27-0.22) | 2375.77 | 12480.72 | 425.33% | -0.85(-1.01 - -0.68) |
| Qatar | APED | 929.88 | 9337.97 | 904.21% | 1.45(1.41-1.49) | 0.32 | 0.94 | 193.75% | -1.87(-2.18 - -1.55) | 26.47 | 155.95 | 489.16% | -0.97(-1.13 - -0.81) |
| Qatar | COCLD | 102.35 | 934.24 | 812.79% | 1.02(0.82-1.23) | 36.79 | 176.67 | 380.21% | -0.16(-0.39-0.07) | 1327.83 | 6072.41 | 357.32% | -1.14(-1.36 - -0.92) |
| Qatar | GABD | 578.95 | 5064.14 | 774.71% | 0.25(0.17-0.32) | 0.75 | 3.42 | 356.00% | 1.08(0.69-1.46) | 82.41 | 476.63 | 478.36% | -0.48(-0.63 - -0.32) |
| Qatar | IFAH | 270.57 | 2560.21 | 846.23% | 1.87(1.73-2.01) | 0.33 | 1.22 | 269.70% | 1.78(1.24-2.32) | 78.62 | 479.75 | 510.21% | 0.33(0.22-0.45) |
| Qatar | IFBD | 13 | 125.15 | 862.69% | 0.9(0.83-0.96) | 0.41 | 2.09 | 409.76% | 0.52(0.07-0.98) | 40.66 | 296.03 | 628.06% | 0.13(-0.11-0.37) |
| Qatar | PACA | 99.62 | 733.76 | 636.56% | -0.15(-0.16 - -0.14) | 1.84 | 9.66 | 425.00% | -0.65(-0.94 - -0.37) | 73.63 | 417.19 | 466.60% | -0.99(-1.19 - -0.78) |
| Qatar | PIIO | 340.39 | 3819.22 | 1022.01% | 2.38(2.16-2.6) | 3.01 | 10.83 | 259.99% | -0.08(-0.08-0.44) | 146.5 | 442.76 | 202.22% | -0.64(-1 - -0.29) |
| Qatar | UDSD | 20580.89 | 160509.97 | 679.90% | -0.025(-0.034 - -0.015) | 2.16 | 5.31 | 145.83% | -1.21(-1.64 - -0.77) | 518.38 | 3723.92 | 618.38% | -0.57(-0.68 - -0.46) |
| Qatar | VAID | 76.65 | 787.22 | 927.03% | 1.88(1.67-2.09) | 0.57 | 3.1 | 443.86% | 0.26(0.01-0.5) | 16.91 | 109.56 | 547.90% | -0.02(-0.23-0.19) |
| Republic of Korea | Digestive diseases | 2039125.94 | 3388732.18 | 66.19% | 0.15(0.02-0.29) | 21168 | 17385.28 | -17.87% | -5.08(-5.44 - -4.72) | 778657.4 | 512621.51 | -34.17% | -4.62(-4.94 - -4.3) |
| Republic of Korea | APED | 204644.13 | 183446.56 | -10.36% | 0.09(0.03-0.15) | 206.57 | 137.55 | -33.41% | -4.43(-5.1 - -3.75) | 10488.49 | 4373.71 | -58.30% | -3.26(-3.92 - -2.6) |
| Republic of Korea | COCLD | 30126.55 | 16886.53 | -43.95% | -3.9(-4.09 - -3.72) | 15888.22 | 10355.65 | -34.82% | -5.57(-5.93 - -5.22) | 556869.47 | 279336.99 | -49.84% | -5.84(-6.16 - -5.52) |
| Republic of Korea | GABD | 397572.07 | 788133.21 | 98.24% | -0.17(-0.23 - -0.11) | 1057.68 | 2034.1 | 92.32% | -2.8(-2.94 - -2.66) | 59363.81 | 78129.04 | 31.61% | -2.28(-2.46 - -2.1) |
| Republic of Korea | IFAH | 72082.52 | 113934.54 | 58.06% | 0.1(-0.01-0.21) | 211.62 | 138.43 | -34.59% | -6.24(-7.03 - -5.45) | 22042.25 | 19829.5 | -10.04% | -1.71(-2 - -1.41) |
| Republic of Korea | IFBD | 1586.68 | 4407.24 | 177.76% | 1.94(1.57-2.3) | 313.12 | 233.74 | -25.35% | -7.96(-8.67 - -7.25) | 9497.96 | 10451.4 | 10.04% | -3.63(-4.02 - -3.23) |
| Republic of Korea | PACA | 10532.95 | 16904.26 | 60.49% | -0.41(-0.44 - -0.37) | 502.8 | 562.14 | 11.80% | -3.54(-3.71 - -3.38) | 17628.97 | 14067.87 | -20.20% | -3.71(-3.92 - -3.5) |
| Republic of Korea | PIIO | 32416.47 | 120474.94 | 271.65% | 2.31(2.1-2.53) | 391.33 | 1593.79 | 307.27% | 0.4(0.11-0.69) | 12286.12 | 23925.29 | 94.73% | -0.75(-0.82 - -0.67) |
| Republic of Korea | UDSD | 1279863.64 | 2108397.41 | 64.74% | 0.225(0.003-0.448) | 2235.9 | 1081.43 | -51.63% | -8.63(-9.38 - -7.87) | 79349.56 | 60460.97 | -23.80% | -4.1(-4.68 - -3.52) |
| Republic of Korea | VAID | 10300.92 | 36147.48 | 250.92% | 1.95(1.89-2.02) | 134.88 | 631.58 | 368.25% | 1.53(1.23-1.83) | 3316.11 | 10298.28 | 210.55% | 0.84(0.56-1.11) |
| Republic of Moldova | Digestive diseases | 291257.17 | 296461.66 | 1.79% | -0.03(-0.04 - -0.01) | 4015.71 | 3513.16 | -12.51% | -1.2(-1.58 - -0.82) | 139451.78 | 114770.77 | -17.70% | -1.17(-1.53 - -0.81) |
| Republic of Moldova | APED | 13836.58 | 10069.57 | -27.23% | -0.13(-0.22 - -0.04) | 21.15 | 7.58 | -64.16% | -4.22(-4.66 - -3.77) | 980.47 | 341.21 | -65.20% | -3.38(-3.76 - -3) |
| Republic of Moldova | COCLD | 4338.57 | 3504.24 | -19.23% | -0.6(-0.88 - -0.32) | 3143.96 | 2744.78 | -12.70% | -1.13(-1.55 - -0.7) | 96716.59 | 81367.58 | -15.87% | -1.05(-1.48 - -0.6) |
| Republic of Moldova | GABD | 39054.11 | 40437.44 | 3.54% | -0.22(-0.28 - -0.16) | 63.79 | 41.27 | -35.30% | -3(-3.46 - -2.53) | 6039.01 | 4767.41 | -21.06% | -1.46(-1.59 - -1.32) |
| Republic of Moldova | IFAH | 9098.3 | 8282.17 | -8.97% | 0.12(-0.08-0.33) | 42.86 | 20.95 | -51.12% | -4(-4.55 - -3.44) | 3225.72 | 2136.07 | -33.78% | -1.4(-1.58 - -1.22) |
| Republic of Moldova | IFBD | 201.32 | 193.78 | -3.75% | 0.38(0.18-0.58) | 16.8 | 17.6 | 4.76% | -0.6(-0.96 - -0.25) | 1045.82 | 999.31 | -4.45% | -0.2(-0.52-0.13) |
| Republic of Moldova | PACA | 3396.62 | 3465.28 | 2.02% | -0.26(-0.32 - -0.21) | 357.96 | 230.24 | -35.68% | -2.7(-3.27 - -2.12) | 14360.94 | 8519.27 | -40.68% | -2.84(-3.42 - -2.26) |
| Republic of Moldova | PIIO | 4605.64 | 4545.38 | -1.31% | -0.35(-0.53 - -0.16) | 52.05 | 59.5 | 14.32% | -0.73(-0.87 - -0.59) | 2122.61 | 1384.61 | -34.77% | -1.97(-2.19 - -1.75) |
| Republic of Moldova | UDSD | 215885.72 | 224201.15 | 3.85% | 0.016(0.01-0.022) | 227.17 | 173.46 | -23.64% | -1.84(-2.19 - -1.48) | 12410.38 | 10375.3 | -16.40% | -1.09(-1.3 - -0.87) |
| Republic of Moldova | VAID | 840.31 | 1762.64 | 109.76% | 2.31(2.04-2.57) | 75.71 | 183.93 | 142.94% | 2.67(2.22-3.13) | 1812.78 | 3773.24 | 108.15% | 2.39(1.97-2.81) |
| Romania | Digestive diseases | 1524641.51 | 1538366.01 | 0.90% | 0.12(0.1-0.14) | 12596.31 | 14625.97 | 16.11% | -0.74(-1.03 - -0.46) | 456340.02 | 443335.08 | -2.85% | -0.93(-1.21 - -0.66) |
| Romania | APED | 48093.15 | 33758.48 | -29.81% | 0.06(0.02-0.1) | 111.86 | 48.04 | -57.05% | -3.88(-4.26 - -3.49) | 4858.68 | 1611.57 | -66.83% | -3.58(-3.96 - -3.2) |
| Romania | COCLD | 11829.77 | 12134.67 | 2.58% | 0.06(-0.15-0.27) | 8646.67 | 10386.46 | 20.12% | -0.66(-1.07 - -0.25) | 264143.18 | 284065.63 | 7.54% | -0.87(-1.32 - -0.43) |
| Romania | GABD | 292409 | 309796.13 | 5.95% | 0.33(0.23-0.42) | 355.17 | 277.98 | -21.73% | -2.45(-2.89 - -2.02) | 41437.86 | 31771.86 | -23.33% | -1(-1.1 - -0.89) |
| Romania | IFAH | 36009.2 | 34809.01 | -3.33% | 0.76(0.65-0.88) | 305.27 | 116.87 | -61.72% | -5.1(-5.65 - -4.53) | 16780.41 | 8625.49 | -48.60% | -2.05(-2.31 - -1.79) |
| Romania | IFBD | 1287.4 | 1186.56 | -7.83% | 0.59(0.34-0.83) | 63.91 | 74.04 | 15.85% | -0.65(-1.01 - -0.29) | 5268.78 | 4363.51 | -17.18% | -0.63(-0.89 - -0.37) |
| Romania | PACA | 12244.69 | 14037.18 | 14.64% | 0.01(-0.07-0.1) | 968.01 | 1042.88 | 7.73% | -0.54(-0.69 - -0.39) | 35385.54 | 31145.42 | -11.98% | -0.98(-1.15 - -0.81) |
| Romania | PIIO | 21762.66 | 29189.61 | 34.13% | 0.66(0.61-0.71) | 601.19 | 769.25 | 27.95% | -0.45(-0.52 - -0.39) | 18840.05 | 15491.92 | -17.77% | -1.2(-1.35 - -1.05) |
| Romania | UDSD | 1096147.45 | 1095308.25 | -0.08% | 0.029(0.023-0.034) | 999.85 | 666.93 | -33.30% | -2.77(-2.96 - -2.58) | 55008.72 | 40882.08 | -25.68% | -1.38(-1.51 - -1.25) |
| Romania | VAID | 4858.2 | 8146.12 | 67.68% | 1.2(1.06-1.33) | 478.11 | 1005.98 | 110.41% | 1.54(1.31-1.76) | 11084.4 | 18527.39 | 67.15% | 1.03(0.85-1.22) |
| Russian Federation | Digestive diseases | 10373000.57 | 11545990.72 | 11.31% | -0.06(-0.16-0.05) | 43349 | 90886.94 | 109.66% | 1.85(1.28-2.43) | 1646786.43 | 3108298.06 | 88.75% | 1.75(1.12-2.38) |
| Russian Federation | APED | 290798.72 | 327059.84 | 12.47% | 1.32(0.88-1.76) | 731.08 | 413.14 | -43.49% | -3.13(-3.35 - -2.9) | 27733.92 | 15013.3 | -45.87% | -2.66(-2.9 - -2.43) |
| Russian Federation | COCLD | 28493.78 | 45082.18 | 58.22% | 2.02(1.74-2.29) | 16501.87 | 46101.77 | 179.37% | 3.15(2.21-4.09) | 515451.46 | 1611988.32 | 212.73% | 3.71(2.62-4.81) |
| Russian Federation | GABD | 1160224.87 | 1332175.73 | 14.82% | 0.09(0.01-0.16) | 3811.7 | 3479.95 | -8.70% | -1.84(-2.06 - -1.63) | 168454.01 | 141769.72 | -15.84% | -1.5(-1.63 - -1.38) |
| Russian Federation | IFAH | 474255.07 | 500395.09 | 5.51% | 0.14(0.09-0.19) | 1271.5 | 1332.83 | 4.82% | -1.69(-1.92 - -1.46) | 101473.93 | 88819.08 | -12.47% | -1.06(-1.15 - -0.98) |
| Russian Federation | IFBD | 13521.69 | 14514.09 | 7.34% | -0.06(-0.16-0.05) | 977.35 | 1257.34 | 28.65% | -0.4(-0.74 - -0.06) | 53401.08 | 56532.96 | 5.86% | -0.52(-0.7 - -0.35) |
| Russian Federation | PACA | 122545.1 | 157598.65 | 28.60% | 0.42(0.34-0.51) | 4786.45 | 11615.29 | 142.67% | 2.34(1.67-3.02) | 177165.27 | 409216.71 | 130.98% | 2.28(1.51-3.05) |
| Russian Federation | PIIO | 151725.55 | 188914.97 | 24.51% | 0.53(0.44-0.61) | 2461.23 | 3117.01 | 26.64% | -0.68(-0.86 - -0.5) | 76965.96 | 73583 | -4.40% | -1.47(-1.69 - -1.24) |
| Russian Federation | UDSD | 8060455.89 | 8866402.87 | 10.00% | -0.193(-0.319 - -0.068) | 7036.86 | 9396.98 | 33.54% | -0.29(-0.69-0.1) | 390476.43 | 432700.92 | 10.81% | -0.64(-0.93 - -0.35) |
| Russian Federation | VAID | 70979.91 | 113847.3 | 60.39% | 1.03(0.88-1.17) | 5073.86 | 11231.6 | 121.36% | 1.87(1.61-2.14) | 107400.99 | 193247.76 | 79.93% | 1.16(0.89-1.42) |
| Rwanda | Digestive diseases | 255724.69 | 552152.44 | 115.92% | 0.16(0.15-0.18) | 3921.37 | 4296.59 | 9.57% | -2.78(-3.11 - -2.45) | 155209.48 | 166514.15 | 7.28% | -2.8(-3.11 - -2.49) |
| Rwanda | APED | 10443.9 | 25795.86 | 146.99% | 1.37(1.18-1.55) | 109.62 | 70.4 | -35.78% | -3.97(-4.33 - -3.61) | 6759.56 | 3965.26 | -41.34% | -4.11(-4.42 - -3.8) |
| Rwanda | COCLD | 1688.58 | 3382.74 | 100.33% | 0.3(0.22-0.38) | 2416.7 | 2599.14 | 7.55% | -3.03(-3.35 - -2.69) | 83901.74 | 88101.66 | 5.01% | -3.18(-3.52 - -2.84) |
| Rwanda | GABD | 2068.27 | 5345.03 | 158.43% | 1.33(1.28-1.39) | 165.38 | 237.74 | 43.75% | -1.44(-1.69 - -1.18) | 5336.29 | 6776.98 | 27.00% | -1.91(-2.19 - -1.63) |
| Rwanda | IFAH | 4940.51 | 13094.57 | 165.04% | 1.99(1.78-2.21) | 104.12 | 104.78 | 0.63% | -1.97(-2.21 - -1.72) | 7225.46 | 7312.38 | 1.20% | -1.27(-1.4 - -1.15) |
| Rwanda | IFBD | 44.98 | 101.8 | 126.32% | 0.4(0.3-0.5) | 34.47 | 42.04 | 21.96% | -1.79(-2.06 - -1.51) | 1761.18 | 1837.6 | 4.34% | -1.92(-2.15 - -1.69) |
| Rwanda | PACA | 977.01 | 1965.59 | 101.18% | -0.08(-0.1 - -0.05) | 76.87 | 102.54 | 33.39% | -2.33(-2.71 - -1.95) | 2870.53 | 3843.97 | 33.91% | -2.36(-2.73 - -1.99) |
| Rwanda | PIIO | 4564.2 | 13592.92 | 197.82% | 2.17(1.95-2.39) | 243.34 | 379.67 | 56.02% | -0.89(-1.11 - -0.68) | 13056.07 | 17506.34 | 34.09% | -0.77(-0.9 - -0.64) |
| Rwanda | UDSD | 230724.97 | 488126.06 | 111.56% | 0.034(0.03-0.038) | 557.75 | 477.2 | -14.44% | -3.8(-4.22 - -3.38) | 26372.84 | 28350.97 | 7.50% | -3.01(-3.36 - -2.66) |
| Rwanda | VAID | 272.27 | 747.89 | 174.69% | 1.79(1.6-1.99) | 40.69 | 71.85 | 76.58% | -0.91(-1.22 - -0.61) | 1067.16 | 1701.61 | 59.45% | -1.13(-1.46 - -0.8) |
| Saint Kitts and Nevis | Digestive diseases | 2751.29 | 5143.08 | 86.93% | 0.03(0.03-0.04) | 22.01 | 23.89 | 8.56% | -1.88(-2.16 - -1.6) | 683.1 | 826.32 | 20.97% | -1.93(-2.21 - -1.66) |
| Saint Kitts and Nevis | APED | 108.36 | 188.48 | 73.94% | 0.81(0.73-0.89) | 0.53 | 0.35 | -33.96% | -2.41(-3.14 - -1.68) | 20.91 | 13.46 | -35.63% | -2.36(-3.07 - -1.65) |
| Saint Kitts and Nevis | COCLD | 8.65 | 18.32 | 111.79% | -0.86(-1.01 - -0.71) | 11.52 | 12.48 | 8.33% | -2.55(-2.95 - -2.14) | 315.92 | 365.96 | 15.84% | -2.87(-3.3 - -2.43) |
| Saint Kitts and Nevis | GABD | 144.95 | 246.43 | 70.01% | -0.47(-0.56 - -0.38) | 1.83 | 1.2 | -34.43% | -2.76(-3.25 - -2.26) | 56.63 | 51.34 | -9.34% | -2.43(-2.82 - -2.04) |
| Saint Kitts and Nevis | IFAH | 75.64 | 123.17 | 62.84% | 0.32(0.22-0.43) | 1.43 | 0.41 | -71.33% | -5.36(-6.61 - -4.1) | 58.12 | 35.6 | -38.75% | -2.86(-3.59 - -2.14) |
| Saint Kitts and Nevis | IFBD | 0.78 | 2.04 | 161.54% | 0.69(0.47-0.9) | 0.8 | 0.88 | 10.00% | -2.01(-2.26 - -1.76) | 24.81 | 28.43 | 14.59% | -2.07(-2.32 - -1.82) |
| Saint Kitts and Nevis | PACA | 11.17 | 19.91 | 78.25% | -0.06(-0.08 - -0.04) | 1.22 | 1.8 | 47.54% | -1.33(-1.51 - -1.15) | 37.93 | 57.98 | 52.86% | -1.56(-1.75 - -1.38) |
| Saint Kitts and Nevis | PIIO | 41.39 | 80.41 | 94.27% | 0.89(0.78-1) | 0.81 | 2.66 | 228.38% | 3.19(2.27-4.12) | 24.63 | 66.19 | 168.74% | 2.32(1.56-3.08) |
| Saint Kitts and Nevis | UDSD | 2354.98 | 4454.57 | 89.16% | 0.009(0.006-0.011) | 2.31 | 1.42 | -38.53% | -3.71(-4.02 - -3.39) | 107.61 | 143.38 | 33.24% | -1.43(-1.59 - -1.28) |
| Saint Kitts and Nevis | VAID | 5.38 | 9.75 | 81.23% | 0.26(0.2-0.33) | 0.86 | 0.92 | 6.98% | -1.06(-1.25 - -0.88) | 16.88 | 20.24 | 19.91% | -1.36(-1.55 - -1.17) |
| Saint Lucia | Digestive diseases | 8131.98 | 14988.84 | 84.32% | 0.09(0.08-0.09) | 37.95 | 57.66 | 51.93% | -1.98(-2.34 - -1.62) | 1426.81 | 2001.67 | 40.29% | -1.55(-1.83 - -1.28) |
| Saint Lucia | APED | 327.53 | 507 | 54.79% | 1.14(1.1-1.18) | 1.05 | 1.02 | -2.86% | -2.11(-2.78 - -1.45) | 49.39 | 38.33 | -22.39% | -1.77(-2.33 - -1.21) |
| Saint Lucia | COCLD | 23.2 | 39.77 | 71.42% | -0.77(-0.91 - -0.62) | 23.26 | 32.86 | 41.27% | -2.34(-2.76 - -1.92) | 704.32 | 939.89 | 33.45% | -2.27(-2.67 - -1.87) |
| Saint Lucia | GABD | 320.25 | 598.64 | 86.93% | 0.06(0-0.12) | 1.58 | 2.02 | 27.85% | -2.47(-3.03 - -1.9) | 77.94 | 106.72 | 36.93% | -1.46(-1.78 - -1.14) |
| Saint Lucia | IFAH | 245.68 | 431.2 | 75.51% | 0.69(0.65-0.73) | 0.69 | 0.99 | 43.48% | -1.55(-1.89 - -1.21) | 90.31 | 116.11 | 28.57% | -0.35(-0.46 - -0.25) |
| Saint Lucia | IFBD | 2.48 | 5.4 | 117.74% | 0.28(0.11-0.45) | 0.89 | 1.32 | 48.31% | -2.24(-2.7 - -1.78) | 35.52 | 44.94 | 26.52% | -1.84(-2.19 - -1.49) |
| Saint Lucia | PACA | 27.79 | 49.95 | 79.74% | -0.14(-0.16 - -0.12) | 0.54 | 1.04 | 92.59% | -1.04(-1.23 - -0.85) | 18.8 | 34.74 | 84.79% | -0.66(-0.79 - -0.52) |
| Saint Lucia | PIIO | 105.16 | 196.33 | 86.70% | 0.84(0.76-0.92) | 2.58 | 5.18 | 100.66% | -0.31(-0.45 - -0.16) | 97.75 | 120.42 | 23.20% | -0.48(-0.59 - -0.36) |
| Saint Lucia | UDSD | 7069.91 | 13138.2 | 85.83% | 0.02(0.018-0.022) | 5 | 5.74 | 14.80% | -3.29(-3.71 - -2.86) | 286.25 | 439.46 | 53.52% | -1.18(-1.34 - -1.02) |
| Saint Lucia | VAID | 9.97 | 22.35 | 124.17% | 0.44(0.39-0.49) | 1.12 | 2.27 | 102.68% | -1.28(-1.66 - -0.89) | 24.42 | 45.01 | 84.32% | -1.2(-1.56 - -0.83) |
| Saint Vincent and the Grenadines | Digestive diseases | 6651.61 | 9414.71 | 41.54% | 0.05(0.05-0.06) | 28.14 | 41.6 | 47.83% | -0.82(-1.12 - -0.52) | 1099.66 | 1460.76 | 32.84% | -0.79(-0.99 - -0.58) |
| Saint Vincent and the Grenadines | APED | 266.06 | 308.09 | 15.80% | 0.98(0.9-1.05) | 0.55 | 0.53 | -3.64% | -0.78(-1.37 - -0.18) | 29.11 | 21.4 | -26.49% | -0.94(-1.41 - -0.48) |
| Saint Vincent and the Grenadines | COCLD | 13.27 | 18.62 | 40.32% | -0.42(-0.52 - -0.32) | 12.13 | 18.92 | 55.98% | -0.89(-1.26 - -0.52) | 377.22 | 563.74 | 49.45% | -0.95(-1.29 - -0.6) |
| Saint Vincent and the Grenadines | GABD | 268.9 | 368.42 | 37.01% | -0.14(-0.19 - -0.09) | 1.53 | 1.76 | 15.03% | -1.27(-1.82 - -0.72) | 70.04 | 82.25 | 17.43% | -1.04(-1.32 - -0.75) |
| Saint Vincent and the Grenadines | IFAH | 210 | 307.96 | 46.65% | 0.66(0.61-0.71) | 0.88 | 1.2 | 36.36% | -0.36(-0.75-0.04) | 89.34 | 98.72 | 10.50% | -0.2(-0.32 - -0.09) |
| Saint Vincent and the Grenadines | IFBD | 1.87 | 3.53 | 88.77% | 0.49(0.27-0.7) | 1 | 1.55 | 55.00% | -0.56(-0.7 - -0.41) | 38.5 | 49.08 | 27.48% | -0.8(-0.94 - -0.67) |
| Saint Vincent and the Grenadines | PACA | 25.38 | 35.71 | 40.70% | -0.07(-0.09 - -0.05) | 1.11 | 1.87 | 68.47% | -0.18(-0.39-0.03) | 39.36 | 60.04 | 52.54% | -0.31(-0.47 - -0.16) |
| Saint Vincent and the Grenadines | PIIO | 86.13 | 109.18 | 26.75% | 0.21(0.15-0.27) | 2.6 | 3.41 | 31.15% | -0.6(-0.88 - -0.32) | 93.24 | 84.9 | -8.94% | -0.98(-1.17 - -0.78) |
| Saint Vincent and the Grenadines | UDSD | 5774.07 | 8253.2 | 42.94% | 0.003(-0.004-0.01) | 6.75 | 7.44 | 10.22% | -2.01(-2.32 - -1.71) | 312.65 | 382.98 | 22.49% | -1.15(-1.29 - -1.02) |
| Saint Vincent and the Grenadines | VAID | 5.93 | 9.99 | 68.47% | 0.63(0.56-0.71) | 0.36 | 0.52 | 44.44% | -0.57(-1.02 - -0.12) | 7.8 | 10.6 | 35.90% | -0.73(-1.13 - -0.33) |
| Samoa | Digestive diseases | 3777.77 | 5605.43 | 48.38% | 0.02(0.01-0.03) | 47.44 | 55.27 | 16.50% | -1.19(-1.33 - -1.05) | 1656 | 1872.32 | 13.06% | -1.12(-1.23 - -1.01) |
| Samoa | APED | 169.42 | 256.23 | 51.24% | 0.43(0.36-0.5) | 0.46 | 0.47 | 2.17% | -1.3(-1.43 - -1.16) | 19.77 | 19.46 | -1.57% | -1.21(-1.31 - -1.11) |
| Samoa | COCLD | 23.06 | 33.67 | 46.01% | -0.23(-0.32 - -0.14) | 25.02 | 30.09 | 20.26% | -1.06(-1.2 - -0.91) | 891.07 | 1028.73 | 15.45% | -1.09(-1.2 - -0.97) |
| Samoa | GABD | 199.15 | 320.26 | 60.81% | 0.19(0.15-0.23) | 2.82 | 4.19 | 48.58% | -0.34(-0.45 - -0.23) | 96.87 | 135.97 | 40.36% | -0.34(-0.41 - -0.26) |
| Samoa | IFAH | 95.01 | 173.13 | 82.22% | 0.71(0.68-0.74) | 0.73 | 0.89 | 21.92% | -1(-1.29 - -0.7) | 42.15 | 58.5 | 38.79% | -0.27(-0.43 - -0.11) |
| Samoa | IFBD | 0.6 | 1.13 | 88.33% | 1.1(0.77-1.44) | 0.86 | 0.92 | 6.98% | -1.64(-1.8 - -1.47) | 30.66 | 30.33 | -1.08% | -1.62(-1.75 - -1.49) |
| Samoa | PACA | 30.03 | 45.08 | 50.12% | -0.06(-0.07 - -0.05) | 1.45 | 2 | 37.93% | -0.64(-0.72 - -0.55) | 51.71 | 68.98 | 33.40% | -0.57(-0.61 - -0.54) |
| Samoa | PIIO | 92.03 | 141.03 | 53.24% | 0.26(0.14-0.38) | 2.04 | 2.88 | 41.40% | -0.45(-0.66 - -0.23) | 62.76 | 71.28 | 13.58% | -0.62(-0.79 - -0.45) |
| Samoa | UDSD | 3162.42 | 4623.66 | 46.21% | -0.039(-0.047 - -0.031) | 12.04 | 10.88 | -9.63% | -2.06(-2.17 - -1.95) | 401.3 | 378.76 | -5.62% | -1.76(-1.86 - -1.66) |
| Samoa | VAID | 6.04 | 11.24 | 86.09% | 0.79(0.69-0.89) | 0.52 | 0.94 | 80.77% | 0.18(0.09-0.27) | 11.74 | 20.72 | 76.49% | 0.32(0.25-0.39) |
| San Marino | Digestive diseases | 1194.6 | 1882.92 | 57.62% | 0.07(0.07-0.07) | 7.19 | 14 | 94.75% | -0.37(-0.48 - -0.26) | 186.03 | 295.21 | 58.69% | -0.38(-0.47 - -0.29) |
| San Marino | APED | 57.99 | 75.33 | 29.90% | 0.2(0.15-0.24) | 0.07 | 0.12 | 71.43% | -1.1(-1.26 - -0.93) | 1.79 | 2.38 | 32.96% | -0.58(-0.7 - -0.47) |
| San Marino | COCLD | 8.97 | 12.08 | 34.67% | -0.43(-0.48 - -0.38) | 4.77 | 8.21 | 72.12% | -0.52(-0.62 - -0.43) | 110.97 | 164.86 | 48.56% | -0.61(-0.72 - -0.5) |
| San Marino | GABD | 99.72 | 174.47 | 74.96% | 0.23(0.21-0.26) | 0.2 | 0.47 | 135.00% | -0.23(-0.38 - -0.08) | 10.39 | 17.21 | 65.64% | -0.22(-0.28 - -0.15) |
| San Marino | IFAH | 24.42 | 41.86 | 71.42% | 0.44(0.36-0.52) | 0.09 | 0.2 | 122.22% | -0.41(-0.58 - -0.23) | 5.7 | 9.42 | 65.26% | -0.08(-0.16-0) |
| San Marino | IFBD | 2.21 | 3.45 | 56.11% | 0.2(0.15-0.25) | 0.11 | 0.3 | 172.73% | 0.22(0.06-0.38) | 5.97 | 10.44 | 74.87% | 0.15(0.11-0.19) |
| San Marino | PACA | 6.01 | 9.77 | 62.56% | -0.14(-0.16 - -0.11) | 0.41 | 0.86 | 109.76% | -0.18(-0.34 - -0.02) | 7.15 | 12.66 | 77.06% | -0.09(-0.24-0.06) |
| San Marino | PIIO | 37.75 | 70.26 | 86.12% | 0.19(0.14-0.24) | 0.69 | 2.2 | 219.72% | 0.69(0.53-0.86) | 9.37 | 23.44 | 150.08% | 0.44(0.29-0.6) |
| San Marino | UDSD | 953.1 | 1486.76 | 55.99% | 0.034(0.031-0.036) | 0.54 | 0.7 | 29.63% | -2.43(-2.61 - -2.24) | 29.31 | 41.92 | 43.02% | -0.41(-0.47 - -0.36) |
| San Marino | VAID | 4.43 | 8.95 | 102.03% | 0.57(0.51-0.63) | 0.17 | 0.44 | 158.82% | 0.37(0.23-0.5) | 2.72 | 5.74 | 111.03% | 0.32(0.2-0.43) |
| Sao Tome and Principe | Digestive diseases | 4304.84 | 8854.41 | 105.68% | 0.06(0.05-0.07) | 58.87 | 65.56 | 11.38% | -1.51(-1.84 - -1.18) | 2259.44 | 2481.95 | 9.85% | -1.62(-1.94 - -1.3) |
| Sao Tome and Principe | APED | 146.41 | 346.69 | 136.79% | 1.05(0.9-1.19) | 0.66 | 0.24 | -63.64% | -4.3(-4.67 - -3.92) | 37.51 | 13.12 | -65.02% | -5.02(-5.34 - -4.71) |
| Sao Tome and Principe | COCLD | 37.99 | 62.94 | 65.68% | -0.49(-0.54 - -0.44) | 41.55 | 49.5 | 19.13% | -1.39(-1.72 - -1.07) | 1400.26 | 1661.41 | 18.65% | -1.56(-1.91 - -1.21) |
| Sao Tome and Principe | GABD | 37.73 | 74.73 | 98.07% | 0.22(0.19-0.25) | 1.21 | 1.27 | 4.96% | -1.25(-1.63 - -0.87) | 42.86 | 42.22 | -1.49% | -1.6(-1.97 - -1.22) |
| Sao Tome and Principe | IFAH | 129.64 | 260.89 | 101.24% | 0.56(0.49-0.62) | 0.81 | 0.78 | -3.70% | -1.29(-1.7 - -0.88) | 69.74 | 86.47 | 23.99% | -0.84(-1.01 - -0.67) |
| Sao Tome and Principe | IFBD | 0.74 | 1.9 | 156.76% | 0.72(0.5-0.94) | 0.34 | 0.35 | 2.94% | -1.58(-1.93 - -1.24) | 14.86 | 15.56 | 4.71% | -1.71(-2.03 - -1.38) |
| Sao Tome and Principe | PACA | 19.97 | 37.66 | 88.58% | -0.13(-0.15 - -0.11) | 1.81 | 2.93 | 61.88% | -0.77(-1.22 - -0.31) | 62.75 | 109.06 | 73.80% | -0.82(-1.31 - -0.33) |
| Sao Tome and Principe | PIIO | 109.8 | 259 | 135.87% | 1.19(1.07-1.31) | 5.75 | 4.93 | -14.23% | -1.69(-1.94 - -1.44) | 302.39 | 211.14 | -30.18% | -2.16(-2.39 - -1.93) |
| Sao Tome and Principe | UDSD | 3815.69 | 7797.38 | 104.35% | -0.008(-0.012 - -0.004) | 3.99 | 3.17 | -20.55% | -2.49(-3.01 - -1.97) | 214.96 | 265.14 | 23.34% | -1.54(-1.84 - -1.23) |
| Sao Tome and Principe | VAID | 6.87 | 13.22 | 92.43% | 0.64(0.59-0.69) | 0.53 | 0.66 | 24.53% | -0.15(-0.49-0.2) | 21.11 | 17.7 | -16.15% | -1.04(-1.32 - -0.77) |
| Saudi Arabia | Digestive diseases | 677358.79 | 2213277.26 | 226.75% | 0.12(0.12-0.13) | 3473.16 | 4819.46 | 38.76% | -2.28(-2.38 - -2.19) | 114023.03 | 198773.3 | 74.33% | -2.25(-2.35 - -2.15) |
| Saudi Arabia | APED | 33255.85 | 113938.76 | 242.61% | 1.45(1.4-1.51) | 26.06 | 26.71 | 2.49% | -2.8(-2.94 - -2.66) | 1357.45 | 2322.2 | 71.07% | -1.62(-1.79 - -1.45) |
| Saudi Arabia | COCLD | 2998.53 | 10224.45 | 240.98% | 0.28(0.23-0.34) | 2614.19 | 3763.12 | 43.95% | -2.22(-2.33 - -2.11) | 72469.4 | 109527.28 | 51.14% | -2.58(-2.72 - -2.45) |
| Saudi Arabia | GABD | 23318 | 86825.41 | 272.35% | 0.23(0.18-0.29) | 176.03 | 231.11 | 31.29% | -2.37(-2.52 - -2.22) | 6632.75 | 12757.53 | 92.34% | -2.09(-2.19 - -1.99) |
| Saudi Arabia | IFAH | 8841.21 | 27153.37 | 207.12% | 1.05(0.95-1.15) | 14.36 | 15.06 | 4.87% | -2.78(-2.94 - -2.61) | 2671.26 | 5485.57 | 105.36% | -0.61(-0.71 - -0.52) |
| Saudi Arabia | IFBD | 341.24 | 1122.14 | 228.84% | -0.14(-0.29-0.01) | 11.79 | 27.26 | 131.21% | -0.6(-0.71 - -0.48) | 1011.64 | 3180.72 | 214.41% | -0.18(-0.25 - -0.1) |
| Saudi Arabia | PACA | 2890.75 | 8606.46 | 197.72% | -0.1(-0.13 - -0.06) | 100.67 | 165.65 | 64.55% | -2.06(-2.15 - -1.98) | 2936.62 | 5881.26 | 100.27% | -1.95(-2.03 - -1.88) |
| Saudi Arabia | PIIO | 11053.68 | 40876.76 | 269.80% | 1.66(1.54-1.78) | 199.15 | 243.99 | 22.52% | -2.3(-2.51 - -2.08) | 5616.34 | 7354.32 | 30.94% | -2.32(-2.53 - -2.11) |
| Saudi Arabia | UDSD | 593728.25 | 1920713.69 | 223.50% | 0.004(0.001-0.008) | 262.53 | 214.44 | -18.32% | -3.53(-3.64 - -3.42) | 19355.56 | 48008.99 | 148.04% | -1.32(-1.4 - -1.24) |
| Saudi Arabia | VAID | 931.27 | 3816.22 | 309.79% | 1.49(1.38-1.59) | 44.65 | 79.83 | 78.79% | -1.44(-1.59 - -1.29) | 968.23 | 2127.99 | 119.78% | -1.36(-1.48 - -1.23) |
| Senegal | Digestive diseases | 263270.69 | 609361.48 | 131.46% | 0.06(0.05-0.07) | 2669.04 | 4373.76 | 63.87% | -1.03(-1.15 - -0.91) | 111886.81 | 170621.61 | 52.49% | -1(-1.14 - -0.85) |
| Senegal | APED | 9423.82 | 24640.85 | 161.47% | 0.74(0.59-0.89) | 50.32 | 35.81 | -28.84% | -3(-3.26 - -2.74) | 2993.07 | 1708.63 | -42.91% | -3.79(-4.1 - -3.48) |
| Senegal | COCLD | 1672.43 | 3594.2 | 114.91% | 0.04(-0.04-0.11) | 1489.47 | 2364.72 | 58.76% | -1.31(-1.43 - -1.19) | 52708.4 | 80650.7 | 53.01% | -1.22(-1.38 - -1.06) |
| Senegal | GABD | 2167.9 | 5259.88 | 142.63% | 0.58(0.56-0.61) | 66.9 | 147.12 | 119.91% | 0.26(0.16-0.37) | 2875.33 | 4929.89 | 71.45% | -0.13(-0.27-0) |
| Senegal | IFAH | 8887.97 | 22687.58 | 155.26% | 0.95(0.91-1) | 53.2 | 91.36 | 71.73% | -0.63(-0.76 - -0.5) | 4773.39 | 8680.01 | 81.84% | -0.11(-0.17 - -0.04) |
| Senegal | IFBD | 51.17 | 132.03 | 158.02% | 0.34(0.21-0.47) | 22.77 | 36.88 | 61.97% | -1(-1.21 - -0.78) | 1083.5 | 1526.64 | 40.90% | -1.12(-1.32 - -0.91) |
| Senegal | PACA | 1205.04 | 2756.74 | 128.77% | 0.04(0.02-0.05) | 118.13 | 276.51 | 134.07% | -0.07(-0.22-0.09) | 4462.81 | 10293.59 | 130.65% | 0.02(-0.16-0.2) |
| Senegal | PIIO | 5877.51 | 14850.85 | 152.67% | 0.7(0.66-0.73) | 338.23 | 643.15 | 90.15% | -0.19(-0.32 - -0.05) | 16436.94 | 25528.33 | 55.31% | -0.29(-0.46 - -0.12) |
| Senegal | UDSD | 233565.51 | 534422.6 | 128.81% | -0.013(-0.019 - -0.007) | 360.96 | 527.23 | 46.06% | -1.17(-1.32 - -1.02) | 19042.42 | 29064.69 | 52.63% | -1.05(-1.18 - -0.93) |
| Senegal | VAID | 419.35 | 1016.76 | 142.46% | 0.77(0.71-0.83) | 35.74 | 71.58 | 100.28% | 0.21(0.09-0.33) | 1540.15 | 1973.55 | 28.14% | -0.49(-0.67 - -0.3) |
| Serbia | Digestive diseases | 586673.19 | 654860.26 | 11.62% | 0.26(0.23-0.29) | 3435.8 | 4245.29 | 23.56% | -0.29(-0.5 - -0.09) | 117406.85 | 116174.99 | -1.05% | -0.72(-0.86 - -0.57) |
| Serbia | APED | 17301.14 | 17466.42 | 0.96% | 0.82(0.66-0.98) | 35.04 | 28.01 | -20.06% | -2.22(-2.49 - -1.95) | 1156.41 | 763.08 | -34.01% | -1.99(-2.16 - -1.83) |
| Serbia | COCLD | 1975.07 | 1640.76 | -16.93% | -0.26(-0.36 - -0.16) | 1516.38 | 1401.08 | -7.60% | -1.31(-1.53 - -1.1) | 46667.08 | 37484.41 | -19.68% | -1.53(-1.75 - -1.31) |
| Serbia | GABD | 93447.53 | 115286.12 | 23.37% | 0.59(0.5-0.67) | 135.8 | 236.81 | 74.38% | 0.67(0.49-0.85) | 12544.75 | 13336.55 | 6.31% | -0.29(-0.35 - -0.22) |
| Serbia | IFAH | 13294.22 | 18503.48 | 39.18% | 1.75(1.5-1.99) | 79.43 | 107.18 | 34.94% | -0.48(-0.75 - -0.22) | 4253 | 4891.9 | 15.02% | 0.33(0.15-0.51) |
| Serbia | IFBD | 909.46 | 1243.71 | 36.75% | 0.78(0.55-1.01) | 49.48 | 113.26 | 128.90% | 2.49(2.05-2.94) | 3314.47 | 4741.09 | 43.04% | 0.83(0.67-0.98) |
| Serbia | PACA | 4281.9 | 4387.87 | 2.47% | -0.14(-0.26 - -0.02) | 310.61 | 398.89 | 28.42% | -0.08(-0.26-0.11) | 9783.13 | 9545.06 | -2.43% | -0.81(-0.95 - -0.66) |
| Serbia | PIIO | 9475.95 | 13560.63 | 43.11% | 0.65(0.57-0.73) | 332.07 | 565.48 | 70.29% | 0.8(0.56-1.04) | 7801.66 | 9689.68 | 24.20% | -0.3(-0.46 - -0.14) |
| Serbia | UDSD | 444203.64 | 479957.4 | 8.05% | 0.101(0.086-0.115) | 613.29 | 744.61 | 21.41% | -0.23(-0.57-0.1) | 23511.46 | 23874.29 | 1.54% | -0.44(-0.61 - -0.26) |
| Serbia | VAID | 1784.27 | 2813.88 | 57.70% | 0.23(0.06-0.39) | 300.98 | 463.98 | 54.16% | -0.02(-0.35-0.3) | 6341.21 | 7880.03 | 24.27% | -0.73(-1.03 - -0.43) |
| Seychelles | Digestive diseases | 2137.34 | 3841.99 | 79.76% | 0.08(0.06-0.1) | 32.32 | 51.37 | 58.95% | -1.08(-1.29 - -0.87) | 1021.72 | 1632.73 | 59.80% | -1.27(-1.47 - -1.08) |
| Seychelles | APED | 148.28 | 259.6 | 75.07% | 0.96(0.75-1.17) | 0.3 | 0.52 | 73.33% | -0.73(-1.4 - -0.06) | 11.98 | 19.07 | 59.18% | -0.78(-1.37 - -0.19) |
| Seychelles | COCLD | 17.31 | 34.69 | 100.40% | -0.15(-0.19 - -0.1) | 17.76 | 33.41 | 88.12% | -0.83(-1.11 - -0.55) | 592.1 | 1081.35 | 82.63% | -1.12(-1.38 - -0.86) |
| Seychelles | GABD | 118.14 | 245.25 | 107.59% | 0.32(0.28-0.37) | 0.61 | 1.04 | 70.49% | 0.03(-0.18-0.24) | 26.02 | 42.88 | 64.80% | -0.5(-0.56 - -0.44) |
| Seychelles | IFAH | 45.22 | 118.19 | 161.37% | 0.97(0.88-1.05) | 0.28 | 0.4 | 42.86% | -0.78(-0.9 - -0.65) | 16.62 | 30.17 | 81.53% | -0.11(-0.17 - -0.06) |
| Seychelles | IFBD | 0.33 | 0.7 | 112.12% | 0.67(0.62-0.73) | 0.17 | 0.22 | 29.41% | -1.44(-1.65 - -1.23) | 5.22 | 6.87 | 31.61% | -1.35(-1.51 - -1.19) |
| Seychelles | PACA | 15.3 | 27.41 | 79.15% | -0.08(-0.09 - -0.08) | 0.81 | 1.44 | 77.78% | -0.72(-0.89 - -0.54) | 29.14 | 50.29 | 72.58% | -0.93(-1.11 - -0.74) |
| Seychelles | PIIO | 94.25 | 187.29 | 98.71% | 0.64(0.52-0.76) | 2.84 | 4.86 | 71.22% | -0.28(-0.57-0.01) | 71.93 | 111.69 | 55.28% | -0.52(-0.73 - -0.32) |
| Seychelles | UDSD | 1693.97 | 2958.72 | 74.66% | -0.083(-0.091 - -0.075) | 8.76 | 7.68 | -12.33% | -2.62(-2.76 - -2.48) | 251.67 | 250.33 | -0.53% | -2.51(-2.66 - -2.36) |
| Seychelles | VAID | 4.53 | 10.16 | 124.28% | 0.97(0.89-1.04) | 0.35 | 0.72 | 105.71% | 0.34(0.05-0.64) | 6.44 | 13.43 | 108.54% | 0.11(-0.16-0.39) |
| Sierra Leone | Digestive diseases | 137442.08 | 330597.55 | 140.54% | 0.16(0.14-0.18) | 1671.54 | 2323.94 | 39.03% | -1.15(-1.22 - -1.08) | 68031.64 | 102547.4 | 50.73% | -0.98(-1.02 - -0.94) |
| Sierra Leone | APED | 4208.95 | 14172.82 | 236.73% | 1.12(0.91-1.32) | 28.14 | 24.47 | -13.04% | -1.86(-2.11 - -1.61) | 1770.99 | 1395.06 | -21.23% | -2.78(-3.08 - -2.48) |
| Sierra Leone | COCLD | 594.96 | 1747.09 | 193.65% | 1.34(1.13-1.55) | 1037.09 | 1192.27 | 14.96% | -2.22(-2.47 - -1.97) | 34572.03 | 44240.43 | 27.97% | -1.95(-2.17 - -1.74) |
| Sierra Leone | GABD | 1026.3 | 2851.63 | 177.86% | 1(0.93-1.07) | 40.83 | 88.15 | 115.90% | 1.21(1.04-1.37) | 1671.84 | 3365.14 | 101.28% | 0.79(0.64-0.93) |
| Sierra Leone | IFAH | 3683.47 | 12403.56 | 236.74% | 2.31(2.01-2.61) | 33.39 | 53.95 | 61.58% | 0.49(0.25-0.73) | 2818.96 | 5602.33 | 98.74% | 0.87(0.69-1.04) |
| Sierra Leone | IFBD | 27.05 | 67.04 | 147.84% | 0.15(-0.01-0.32) | 10.81 | 20.22 | 87.05% | 0.2(-0.07-0.48) | 554.25 | 980.54 | 76.91% | 0(-0.27-0.26) |
| Sierra Leone | PACA | 593.96 | 1458.81 | 145.61% | 0.35(0.31-0.38) | 55.37 | 150.06 | 171.01% | 1.23(0.96-1.51) | 2053.26 | 5860.31 | 185.41% | 1.4(1.1-1.71) |
| Sierra Leone | PIIO | 2741.78 | 7071.67 | 157.92% | 0.84(0.72-0.95) | 190.61 | 347.93 | 82.54% | 0.51(0.28-0.75) | 10867.82 | 17482.09 | 60.86% | 0.04(-0.17-0.25) |
| Sierra Leone | UDSD | 124393.68 | 290370.88 | 133.43% | 0.041(0.036-0.047) | 191.88 | 320.3 | 66.93% | 0.21(-0.04-0.45) | 9873.5 | 18464.83 | 87.01% | 0.1(-0.07-0.28) |
| Sierra Leone | VAID | 171.93 | 454.05 | 164.09% | 1.21(1.02-1.41) | 15.29 | 29.7 | 94.24% | 1.05(0.84-1.26) | 674.43 | 1016.41 | 50.71% | 0.27(-0.04-0.57) |
| Singapore | Digestive diseases | 150191.5 | 351577.81 | 134.09% | 0.09(0.08-0.1) | 399.73 | 592.18 | 48.14% | -3.12(-3.28 - -2.96) | 17133.82 | 25796.83 | 50.56% | -2.21(-2.36 - -2.07) |
| Singapore | APED | 13689.03 | 19343.95 | 41.31% | 0.12(0.08-0.15) | 3.3 | 3.16 | -4.24% | -4.46(-4.73 - -4.19) | 288.64 | 304.96 | 5.65% | -1.72(-1.86 - -1.58) |
| Singapore | COCLD | 560.65 | 747.57 | 33.34% | -1.6(-1.77 - -1.42) | 205.77 | 253 | 22.95% | -3.48(-3.75 - -3.21) | 6332.49 | 6482.99 | 2.38% | -3.82(-4.07 - -3.56) |
| Singapore | GABD | 28094.74 | 74758.51 | 166.09% | 0.04(0.01-0.06) | 33.61 | 83.43 | 148.23% | -1.88(-2.03 - -1.73) | 3030.35 | 6409.11 | 111.50% | -1.16(-1.21 - -1.11) |
| Singapore | IFAH | 4801.41 | 12086.23 | 151.72% | 0.41(0.38-0.44) | 6.57 | 9.71 | 47.79% | -3.33(-3.58 - -3.08) | 1122.28 | 2078.73 | 85.22% | -0.6(-0.64 - -0.56) |
| Singapore | IFBD | 122.86 | 214.98 | 74.98% | -0.35(-0.57 - -0.13) | 3.83 | 5.36 | 39.95% | -3.18(-3.41 - -2.95) | 375.05 | 612.43 | 63.29% | -1.52(-1.73 - -1.31) |
| Singapore | PACA | 317.34 | 1303.89 | 310.88% | 1.13(1.06-1.21) | 15.66 | 27.75 | 77.20% | -2.21(-2.37 - -2.05) | 510.92 | 767.89 | 50.30% | -2.26(-2.4 - -2.11) |
| Singapore | PIIO | 3314.5 | 11504.06 | 247.08% | 1.24(1.18-1.29) | 16.1 | 51.95 | 222.61% | -0.12(-0.3-0.07) | 543.53 | 1000.75 | 84.12% | -1.02(-1.28 - -0.77) |
| Singapore | UDSD | 98287.9 | 227786.31 | 131.75% | 0.012(0.002-0.021) | 87.05 | 58.68 | -32.59% | -6.74(-7.03 - -6.44) | 4088.66 | 6035.14 | 47.61% | -2.56(-2.72 - -2.39) |
| Singapore | VAID | 1003.06 | 3832.33 | 282.06% | 1.52(1.43-1.62) | 11.69 | 58.09 | 396.92% | 1.71(1.21-2.2) | 303.9 | 1100.8 | 262.22% | 1.05(0.59-1.51) |
| Slovakia | Digestive diseases | 359254.07 | 450845.28 | 25.49% | 0(-0.01-0.02) | 2674.5 | 2988.95 | 11.76% | -0.6(-0.84 - -0.36) | 97403.62 | 97938.23 | 0.55% | -0.82(-1.02 - -0.62) |
| Slovakia | APED | 11471.57 | 11513.36 | 0.36% | 0.65(0.52-0.78) | 13.66 | 11.84 | -13.32% | -1.79(-2.08 - -1.51) | 468.14 | 372.34 | -20.46% | -1.06(-1.27 - -0.86) |
| Slovakia | COCLD | 2042.8 | 2056.9 | 0.69% | -0.29(-0.4 - -0.17) | 1634.22 | 1693.71 | 3.64% | -0.84(-1.1 - -0.58) | 53999.18 | 51927.32 | -3.84% | -0.98(-1.25 - -0.72) |
| Slovakia | GABD | 85687.02 | 105458.33 | 23.07% | -0.28(-0.36 - -0.2) | 160.72 | 161.06 | 0.21% | -1.21(-1.62 - -0.79) | 11198.36 | 10855.15 | -3.06% | -1.18(-1.32 - -1.04) |
| Slovakia | IFAH | 7869.42 | 11410.56 | 45.00% | 0.92(0.83-1.01) | 50.78 | 48.82 | -3.86% | -1.58(-1.73 - -1.42) | 2685 | 2773.26 | 3.29% | -0.45(-0.55 - -0.35) |
| Slovakia | IFBD | 566.32 | 569.25 | 0.52% | -0.17(-0.27 - -0.08) | 30.98 | 58.31 | 88.22% | 1.56(1.21-1.92) | 2273.84 | 2855.9 | 25.60% | 0.34(0.19-0.49) |
| Slovakia | PACA | 4414.99 | 4950.06 | 12.12% | -0.25(-0.33 - -0.17) | 230.42 | 237.36 | 3.01% | -1.03(-1.27 - -0.78) | 7647.36 | 6778.31 | -11.36% | -1.34(-1.58 - -1.1) |
| Slovakia | PIIO | 6306.07 | 8911.48 | 41.32% | 0.11(0.05-0.17) | 122.48 | 233.71 | 90.82% | 1.29(0.98-1.6) | 3017.53 | 4281.71 | 41.89% | 0.43(0.16-0.69) |
| Slovakia | UDSD | 240366.2 | 304953.62 | 26.87% | 0.032(0.029-0.034) | 319.01 | 295.35 | -7.42% | -1.29(-1.55 - -1.02) | 13131.43 | 13064.21 | -0.51% | -0.82(-0.97 - -0.66) |
| Slovakia | VAID | 529.69 | 1021.73 | 92.89% | 0.61(0.16-1.06) | 45.09 | 126.49 | 180.53% | 2.9(2.55-3.25) | 871.06 | 2093.88 | 140.38% | 2.3(1.96-2.64) |
| Slovenia | Digestive diseases | 134520.34 | 171328.25 | 27.36% | 0.13(0.08-0.18) | 1215.17 | 1123.73 | -7.52% | -2.62(-2.9 - -2.33) | 39106.71 | 29922.17 | -23.49% | -2.53(-2.77 - -2.28) |
| Slovenia | APED | 4356.15 | 4372.87 | 0.38% | 0.37(0.11-0.63) | 5.92 | 3.53 | -40.37% | -4.36(-4.67 - -4.06) | 170.85 | 110.66 | -35.23% | -1.88(-2.01 - -1.75) |
| Slovenia | COCLD | 992.62 | 739.96 | -25.45% | -1.45(-1.58 - -1.33) | 781.72 | 569.32 | -27.17% | -2.99(-3.3 - -2.68) | 24751.39 | 15135.01 | -38.85% | -3.39(-3.72 - -3.05) |
| Slovenia | GABD | 24338.11 | 33412.95 | 37.29% | 0.34(0.21-0.48) | 62.11 | 86.49 | 39.25% | -1.1(-1.4 - -0.8) | 3062.86 | 3382.32 | 10.43% | -0.76(-0.87 - -0.66) |
| Slovenia | IFAH | 3368.01 | 4977.8 | 47.80% | 1.31(1.09-1.53) | 25.28 | 25.29 | 0.04% | -2.85(-3.02 - -2.68) | 1068.66 | 1107.78 | 3.66% | -0.47(-0.59 - -0.34) |
| Slovenia | IFBD | 231.04 | 276.82 | 19.81% | 0.45(0.37-0.54) | 9.88 | 18.37 | 85.93% | -1.39(-2.13 - -0.65) | 781.34 | 977.46 | 25.10% | -0.42(-0.67 - -0.17) |
| Slovenia | PACA | 1075.25 | 1236.8 | 15.02% | -0.72(-0.76 - -0.68) | 66.42 | 55.58 | -16.32% | -3.25(-3.51 - -2.99) | 1917.12 | 1209.8 | -36.89% | -3.61(-3.87 - -3.35) |
| Slovenia | PIIO | 3042.11 | 4894.66 | 60.90% | 0.37(0.28-0.46) | 32.43 | 88.17 | 171.89% | 0.9(0.5-1.3) | 679.45 | 1230.4 | 81.09% | -0.1(-0.38-0.18) |
| Slovenia | UDSD | 96563.92 | 120435.02 | 24.72% | 0.025(0.002-0.049) | 132.62 | 93.66 | -29.38% | -4.73(-5.25 - -4.21) | 4848.75 | 4139.36 | -14.63% | -1.99(-2.18 - -1.8) |
| Slovenia | VAID | 553.13 | 981.38 | 77.42% | -0.18(-0.44-0.08) | 85.81 | 138.79 | 61.74% | -1.1(-1.37 - -0.83) | 1445.59 | 1836.88 | 27.07% | -1.65(-1.92 - -1.39) |
| Solomon Islands | Digestive diseases | 7270.85 | 16078.35 | 121.13% | 0.01(-0.02-0.03) | 109.09 | 191.63 | 75.66% | -0.91(-0.95 - -0.87) | 4751.88 | 8473.66 | 78.32% | -0.87(-0.91 - -0.83) |
| Solomon Islands | APED | 359.98 | 815.52 | 126.55% | 0.16(0.01-0.01) | 1.3 | 1.99 | 53.08% | -1.27(-1.4 - -1.14) | 65.2 | 101.38 | 55.49% | -1.2(-1.32 - -1.08) |
| Solomon Islands | COCLD | 63.87 | 132.78 | 107.89% | -0.4(-0.48 - -0.32) | 70.62 | 133.08 | 88.45% | -0.75(-0.8 - -0.71) | 2950.68 | 5623.14 | 90.57% | -0.74(-0.79 - -0.7) |
| Solomon Islands | GABD | 357.63 | 874.16 | 144.43% | 0.35(0.32-0.38) | 4.01 | 9.09 | 126.68% | 0.16(0.07-0.25) | 196.84 | 431.32 | 119.12% | -0.04(-0.12-0.04) |
| Solomon Islands | IFAH | 214.16 | 544.44 | 154.22% | 0.43(0.24-0.62) | 1.19 | 1.78 | 49.58% | -0.94(-1.05 - -0.83) | 108.93 | 196.04 | 79.97% | -0.41(-0.51 - -0.31) |
| Solomon Islands | IFBD | 1.05 | 2.96 | 181.90% | 0.88(0.79-0.97) | 1.18 | 1.94 | 64.41% | -1.42(-1.55 - -1.29) | 52.56 | 85.81 | 63.26% | -1.38(-1.51 - -1.25) |
| Solomon Islands | PACA | 49.72 | 109.52 | 120.27% | -0.08(-0.09 - -0.07) | 2.32 | 4.84 | 108.62% | -0.4(-0.47 - -0.33) | 98.8 | 209.82 | 112.37% | -0.39(-0.46 - -0.31) |
| Solomon Islands | PIIO | 141.93 | 312.27 | 120.01% | 0.33(0.2-0.47) | 3.18 | 5.35 | 68.29% | -0.48(-0.54 - -0.43) | 161.71 | 236.67 | 46.35% | -0.61(-0.69-0.54) |
| Solomon Islands | UDSD | 6073.8 | 13265 | 118.40% | -0.042(-0.052 - -0.032) | 23.07 | 28.73 | 24.53% | -1.8(-1.83 - -1.77) | 1028.26 | 1404.05 | 36.55% | -1.63(-1.67 - -1.6) |
| Solomon Islands | VAID | 8.71 | 21.68 | 148.91% | 0.69(0.59-0.78) | 0.51 | 1.32 | 158.82% | 0.49(0.33-0.65) | 15.7 | 40.87 | 160.32% | 0.5(0.34-0.67) |
| Somalia | Digestive diseases | 246216.86 | 716905.61 | 191.17% | 0.09(0.08-0.1) | 2607.27 | 6018.24 | 130.83% | -0.41(-0.52 - -0.3) | 110939.93 | 261591.61 | 135.80% | -0.43(-0.52 - -0.34) |
| Somalia | APED | 9876.29 | 36193.9 | 266.47% | 0.93(0.77-1.08) | 75.88 | 164.38 | 116.63% | -0.59(-0.68 - -0.49) | 4652.02 | 9954.85 | 113.99% | -0.63(-0.73 - -0.52) |
| Somalia | COCLD | 1548.32 | 4398.14 | 184.06% | 0.11(0.03-0.19) | 1656.09 | 3790.38 | 128.88% | -0.46(-0.57 - -0.36) | 59901.45 | 139559.97 | 132.98% | -0.49(-0.59 - -0.4) |
| Somalia | GABD | 1648.39 | 5334.55 | 223.62% | 0.8(0.73-0.86) | 82.49 | 210.28 | 154.92% | 0.28(0.17-0.4) | 3075.97 | 7534.68 | 144.95% | 0.04(-0.06-0.15) |
| Somalia | IFAH | 5184.05 | 15610.77 | 201.13% | 0.65(0.59-0.72) | 64.58 | 146.45 | 126.77% | -0.06(-0.16-0.05) | 5343.14 | 12528.56 | 134.48% | -0.01(-0.08-0.06) |
| Somalia | IFBD | 36.56 | 100.22 | 174.12% | 0.02(-0.01-0.05) | 18.99 | 39.8 | 109.58% | -0.45(-0.53 - -0.37) | 1020.96 | 1986.46 | 94.57% | -0.63(-0.69 - -0.56) |
| Somalia | PACA | 862.94 | 2412.59 | 179.58% | -0.02(-0.03 - -0.01) | 43.1 | 97.65 | 126.57% | -0.62(-0.68 - -0.56) | 1676.41 | 3924.62 | 134.11% | -0.59(-0.65 - -0.53) |
| Somalia | PIIO | 4149.35 | 11906 | 186.94% | 0.36(0.28-0.44) | 217.53 | 578.68 | 166.02% | -0.05(-0.13-0.03) | 11818.76 | 31196.69 | 163.96% | 0.04(-0.03-0.1) |
| Somalia | UDSD | 222700.99 | 640346.25 | 187.54% | 0.048(0.044-0.052) | 329.25 | 741.1 | 125.09% | -0.57(-0.72 - -0.43) | 18522.29 | 45419.01 | 145.21% | -0.46(-0.57 - -0.36) |
| Somalia | VAID | 209.97 | 603.18 | 187.27% | 0.41(0.33-0.5) | 21.46 | 52.63 | 145.25% | 0.03(-0.04-0.1) | 637.08 | 1479.31 | 132.20% | -0.16(-0.22 - -0.11) |
| South Africa | Digestive diseases | 1722516.07 | 3210873.15 | 86.41% | 0.07(0.06-0.09) | 9048.18 | 13356.22 | 47.61% | -0.96(-1.37 - -0.54) | 394534.49 | 501159.28 | 27.03% | -1.32(-1.76 - -0.89) |
| South Africa | APED | 45225.17 | 87538.3 | 93.56% | 1.28(0.42-2.13) | 141.61 | 163.58 | 15.51% | -1.01(-1.75 - -0.27) | 8456.79 | 8298.71 | -1.87% | -1.4(-2.16 - -0.63) |
| South Africa | COCLD | 6346.69 | 7807.48 | 23.02% | -1.28(-1.38 - -1.18) | 4683.94 | 5824.45 | 24.35% | -1.86(-2.32 - -1.39) | 180253.57 | 192238.02 | 6.65% | -2.27(-2.8 - -1.73) |
| South Africa | GABD | 33321.81 | 62361.38 | 87.15% | 0.34(0.29-0.38) | 429.24 | 882.82 | 105.67% | 0.32(-0.02-0.66) | 16463.99 | 27266.91 | 65.62% | -0.17(-0.53-0.2) |
| South Africa | IFAH | 86659.07 | 132063.04 | 52.39% | -0.04(-0.12-0.03) | 162.35 | 317.81 | 95.76% | 0.21(-0.02-0.43) | 24006.51 | 31837.76 | 32.62% | -0.52(-0.61 - -0.44) |
| South Africa | IFBD | 376.05 | 746.31 | 98.46% | 0.13(0.06-0.2) | 98.01 | 166.49 | 69.87% | 0.13(-0.38-0.64) | 4862.11 | 6142.82 | 26.34% | -0.56(-0.99 - -0.14) |
| South Africa | PACA | 6593.6 | 11714.56 | 77.67% | -0.15(-0.19 - -0.11) | 239.74 | 388.55 | 62.07% | -0.97(-1.39 - -0.55) | 9944.07 | 14257.91 | 43.38% | -1.41(-1.85 - -0.96) |
| South Africa | PIIO | 35457.06 | 66824.86 | 88.47% | 0.21(0.06-0.37) | 1019.53 | 2027.15 | 98.83% | 0.17(-0.02-0.35) | 36108.16 | 58762.05 | 62.74% | -0.16(-0.39-0.07) |
| South Africa | UDSD | 1505428.33 | 2836182.77 | 88.40% | 0.049(0.033-0.066) | 1350.52 | 2217.15 | 64.17% | 0.02(-0.57-0.62) | 81413.73 | 125065.06 | 53.62% | -0.32(-0.78-0.14) |
| South Africa | VAID | 3108.29 | 5634.44 | 81.27% | 0.33(0.24-0.41) | 140.71 | 324.33 | 130.50% | 0.44(0.25-0.63) | 3999.69 | 7887.17 | 97.19% | 0.13(-0.08-0.35) |
| South Sudan | Digestive diseases | 209162.75 | 345211.82 | 65.04% | 0.1(0.08-0.11) | 1968.35 | 2712.9 | 37.83% | -0.28(-0.34 - -0.23) | 78907.25 | 107631.11 | 36.40% | -0.43(-0.49 - -0.37) |
| South Sudan | APED | 7506.58 | 15376.62 | 104.84% | 0.93(0.76-1.1) | 47.61 | 52.33 | 9.91% | -0.84(-0.93 - -0.74) | 3001.81 | 3166.14 | 5.47% | -1.08(-1.27 - -0.89) |
| South Sudan | COCLD | 953.93 | 1705.48 | 78.78% | 0.27(0.19-0.35) | 1209.46 | 1706.91 | 41.13% | -0.32(-0.38 - -0.25) | 39803.49 | 57050.91 | 43.33% | -0.45(-0.52 - -0.38) |
| South Sudan | GABD | 1473.64 | 2678.8 | 81.78% | 0.51(0.47-0.55) | 78.62 | 118.69 | 50.97% | 0.24(0.17-0.31) | 2467.93 | 3587.34 | 45.36% | 0.03(-0.04-0.1) |
| South Sudan | IFAH | 3864.37 | 6603.44 | 70.88% | 0.37(0.29-0.45) | 56.69 | 66.28 | 16.92% | 0.01(-0.07-0.09) | 4514.45 | 5220.5 | 15.64% | -0.33(-0.42 - -0.23) |
| South Sudan | IFBD | 37 | 63.47 | 71.54% | 0.25(0.22-0.28) | 22.11 | 28.67 | 29.67% | -0.05(-0.09-0) | 1158.03 | 1315.28 | 13.58% | -0.45(-0.58 - -0.32) |
| South Sudan | PACA | 753.51 | 1211.26 | 60.75% | -0.06(-0.08 - -0.05) | 35.18 | 47.49 | 34.99% | -0.58(-0.61 - -0.54) | 1253.7 | 1696.84 | 35.35% | -0.74(-0.8 - -0.67) |
| South Sudan | PIIO | 4247.79 | 6961.1 | 63.88% | 0.2(0.13-0.28) | 199.3 | 286.07 | 43.54% | 0.08(0.03-0.13) | 10636.42 | 14169.52 | 33.22% | -0.21(-0.29 - -0.14) |
| South Sudan | UDSD | 190014.08 | 310068.3 | 63.18% | 0.064(0.057-0.071) | 201.6 | 240.39 | 19.24% | -0.8(-0.85 - -0.75) | 11830 | 16082.75 | 35.95% | -0.56(-0.6 - -0.51) |
| South Sudan | VAID | 311.86 | 543.35 | 74.23% | 0.43(0.35-0.5) | 30.9 | 47.36 | 53.27% | 0.23(0.2-0.27) | 827.15 | 1177.05 | 42.30% | -0.01(-0.05-0.03) |
| Spain | Digestive diseases | 1852171.87 | 2561916.21 | 38.32% | 0.05(-0.06-0.15) | 20286.91 | 23211.95 | 14.42% | -2.26(-2.35 - -2.16) | 512755.11 | 453572.42 | -11.54% | -2.33(-2.43 - -2.23) |
| Spain | APED | 95528.76 | 99780.44 | 4.45% | 0.3(0.27-0.33) | 104.19 | 127.35 | 22.23% | -1.64(-1.9 - -1.39) | 3316.88 | 3128.94 | -5.67% | -0.98(-1.11 - -0.85) |
| Spain | COCLD | 15287.28 | 13480.1 | -11.82% | -2.03(-2.13 - -1.94) | 11524.62 | 8217.99 | -28.69% | -3.34(-3.46 - -3.22) | 300446.57 | 180815.07 | -39.82% | -3.57(-3.7 - -3.45) |
| Spain | GABD | 154063.31 | 210412.35 | 36.58% | -0.47(-0.66 - -0.27) | 1196.53 | 2915.75 | 143.68% | 0.05(-0.07-0.17) | 29249.09 | 45110.01 | 54.23% | -0.7(-0.75 - -0.64) |
| Spain | IFAH | 54739.62 | 78200 | 42.86% | -0.06(-0.1 - -0.02) | 547.18 | 752.59 | 37.54% | -2(-2.16 - -1.84) | 18609.43 | 20784.06 | 11.69% | -1.25(-1.37 - -1.12) |
| Spain | IFBD | 4177.16 | 6797.82 | 62.74% | 0.86(0.76-0.97) | 277.73 | 377.14 | 35.79% | -2.16(-2.54 - -1.78) | 11346.66 | 17465.75 | 53.93% | -0.26(-0.46 - -0.05) |
| Spain | PACA | 15591.92 | 17924.98 | 14.96% | -0.59(-0.84 - -0.34) | 1161.25 | 1591.99 | 37.09% | -1.61(-1.75 - -1.46) | 26829.61 | 27236.62 | 1.52% | -2.04(-2.17 - -1.92) |
| Spain | PIIO | 54126.73 | 109540.37 | 102.38% | 0.8(0.68-0.92) | 1067.61 | 2940.01 | 175.38% | 0.55(0.27-0.83) | 16988.63 | 35108.27 | 106.66% | 0.16(-0.08-0.41) |
| Spain | UDSD | 1446078.37 | 1999623.23 | 38.28% | 0.071(-0.048-0.191) | 2163.89 | 879.05 | -59.38% | -6.58(-7.09 - -6.07) | 68835.91 | 56835.73 | -17.43% | -1.89(-2.21 - -1.57) |
| Spain | VAID | 12578.72 | 26156.92 | 107.95% | 0.51(0.29-0.73) | 1687.9 | 3649.77 | 116.23% | -0.61(-1.03 - -0.18) | 26768.46 | 43744.45 | 63.42% | -0.98(-1.36 - -0.6) |
| Sri Lanka | Digestive diseases | 493495.43 | 810063.92 | 64.15% | 0.16(0.13-0.19) | 4683.02 | 5349.97 | 14.24% | -2.53(-3.02 - -2.04) | 183137.51 | 179188.91 | -2.16% | -2.79(-3.3 - -2.27) |
| Sri Lanka | APED | 30981.55 | 47868.96 | 54.51% | 0.95(0.91-0.99) | 31.51 | 17.45 | -44.62% | -3.57(-4.02 - -3.11) | 1844 | 1188.65 | -35.54% | -2.22(-2.57 - -1.86) |
| Sri Lanka | COCLD | 4156.86 | 5600.81 | 34.74% | -0.67(-0.89 - -0.45) | 3849.83 | 4368.85 | 13.48% | -2.68(-3.25 - -2.1) | 140131.57 | 129944.34 | -7.27% | -3.24(-3.9 - -2.57) |
| Sri Lanka | GABD | 28269.08 | 54428.91 | 92.54% | 0.7(0.61-0.78) | 60.7 | 80.33 | 32.34% | -1.51(-1.73 - -1.29) | 4740.2 | 6574.24 | 38.69% | -0.85(-0.94 - -0.76) |
| Sri Lanka | IFAH | 12935.99 | 29572.42 | 128.61% | 0.56(0.26-0.87) | 129.93 | 104.71 | -19.41% | -2.4(-3.02 - -1.78) | 5977.86 | 6830.9 | 14.27% | -1.32(-1.68 - -0.96) |
| Sri Lanka | IFBD | 106.87 | 267.83 | 150.61% | 1.86(1.62-2.11) | 18.86 | 21.5 | 14.00% | -2.15(-2.31 - -1.99) | 812.99 | 895.19 | 10.11% | -1.45(-1.57 - -1.33) |
| Sri Lanka | PACA | 3002.51 | 4694.13 | 56.34% | -0.22(-0.25 - -0.2) | 93.15 | 97.06 | 4.20% | -2.12(-2.21 - -2.03) | 3667.1 | 3163.25 | -13.74% | -2.46(-2.62 - -2.3) |
| Sri Lanka | PIIO | 21365.14 | 50879.68 | 138.14% | 1.28(1.16-1.4) | 148.82 | 272.59 | 83.17% | -1.91(-2.55 - -1.27) | 5408.77 | 6663.08 | 23.19% | -1.99(-2.47 - -1.51) |
| Sri Lanka | UDSD | 391981.38 | 614761.35 | 56.83% | -0.029(-0.041 - -0.018) | 259.27 | 187.72 | -27.60% | -3.09(-3.35 - -2.82) | 17649.58 | 19509.38 | 10.54% | -1.3(-1.42 - -1.18) |
| Sri Lanka | VAID | 696.05 | 1989.83 | 185.87% | 1.6(1.53-1.67) | 37.98 | 93.78 | 146.92% | 0.25(0-0.51) | 887.97 | 1805.01 | 103.27% | -0.09(-0.29-0.12) |
| Sudan | Digestive diseases | 815115.05 | 1881971.99 | 130.88% | 0.1(0.09-0.11) | 4665.85 | 6314.17 | 35.33% | -1.04(-1.09 - -0.98) | 207371.18 | 262401.81 | 26.54% | -1.15(-1.2 - -1.09) |
| Sudan | APED | 40729.91 | 120298.43 | 195.36% | 1.08(0.96-1.2) | 157.69 | 137.57 | -12.76% | -2.03(-2.08 - -1.98) | 9314.2 | 7453.02 | -19.98% | -2.33(-2.42 - -2.24) |
| Sudan | COCLD | 1954.93 | 4800.61 | 145.56% | 0.57(0.25-0.89) | 3055.6 | 4099.69 | 34.17% | -1.24(-1.34 - -1.13) | 114382.41 | 134732.52 | 17.79% | -1.48(-1.58 - -1.38) |
| Sudan | GABD | 23656.82 | 61371.45 | 159.42% | 0.57(0.53-0.62) | 117.56 | 263 | 123.72% | 0.75(0.64-0.86) | 6869.83 | 13067.24 | 90.21% | -0.05(-0.13-0.03) |
| Sudan | IFAH | 9581.17 | 22586.28 | 135.74% | 1(0.95-1.05) | 36.71 | 45.58 | 24.16% | -0.6(-0.73 - -0.47) | 4871.09 | 7534.59 | 54.68% | -0.17(-0.23 - -0.12) |
| Sudan | IFBD | 334.03 | 933.96 | 179.60% | 0.67(0.63-0.72) | 21.32 | 47.74 | 123.92% | 0.95(0.78-1.11) | 1309.54 | 3002.85 | 129.31% | 0.71(0.58-0.84) |
| Sudan | PACA | 3327.82 | 7298.48 | 119.32% | 0.05(0.02-0.07) | 80.28 | 166.15 | 106.96% | 0.25(0.19-0.32) | 2440.98 | 4933.12 | 102.10% | 0.06(-0.01-0.14) |
| Sudan | PIIO | 8509.91 | 21006.15 | 146.84% | 0.83(0.73-0.92) | 359.98 | 503.92 | 39.98% | 0.32(0.21-0.43) | 23044 | 24243.17 | 5.20% | -0.19(-0.33 - -0.06) |
| Sudan | UDSD | 726309.53 | 1641489.56 | 126.00% | 0.021(0.018-0.023) | 734.9 | 763.9 | 3.95% | -1.91(-1.94 - -1.87) | 41327.15 | 58265.08 | 40.98% | -1.2(-1.23 - -1.17) |
| Sudan | VAID | 710.94 | 2187.05 | 207.63% | 1.42(1.27-1.57) | 43.27 | 93.55 | 116.20% | 0.4(0.36-0.45) | 959.6 | 1983.07 | 106.66% | 0.31(0.26-0.37) |
| Suriname | Digestive diseases | 24806.42 | 44965.79 | 81.27% | 0.07(0.06-0.08) | 145.88 | 250.7 | 71.86% | -0.98(-1.18 - -0.79) | 5731.49 | 8498.3 | 48.27% | -1.12(-1.26 - -0.98) |
| Suriname | APED | 872.03 | 1592.17 | 82.58% | 1.06(0.9-1.22) | 3.11 | 2.96 | -4.82% | -1.81(-2.32 - -1.3) | 161.41 | 126.48 | -21.64% | -1.97(-2.5 - -1.44) |
| Suriname | COCLD | 74.3 | 151.18 | 103.47% | -0.03(-0.12-0.05) | 73.31 | 126.73 | 72.87% | -1.19(-1.37 - -1.01) | 2347.97 | 3659.95 | 55.88% | -1.42(-1.6 - -1.24) |
| Suriname | GABD | 1023.56 | 1888.67 | 84.52% | 0.04(-0.02-0.09) | 7.98 | 11.12 | 39.35% | -1.62(-1.98 - -1.25) | 353.19 | 489.74 | 38.66% | -1.34(-1.58 - -1.11) |
| Suriname | IFAH | 825.49 | 1491.97 | 80.74% | 0.31(0.26-0.36) | 5.38 | 7.12 | 32.34% | -1.39(-1.53 - -1.24) | 463.41 | 555.5 | 19.87% | -1.14(-1.21 - -1.08) |
| Suriname | IFBD | 7.54 | 16.92 | 124.40% | 0.51(0.37-0.65) | 2.85 | 3.78 | 32.63% | -1.99(-2.24 - -1.73) | 117.65 | 137.61 | 16.97% | -1.99(-2.24 - -1.75) |
| Suriname | PACA | 94.95 | 177.13 | 86.55% | 0.03(0.01-0.04) | 4.85 | 9.87 | 103.51% | -0.29(-0.51 - -0.08) | 176.47 | 326.8 | 85.19% | -0.41(-0.61 - -0.21) |
| Suriname | PIIO | 247.63 | 491.98 | 98.67% | 0.6(0.53-0.67) | 19.52 | 36.22 | 85.53% | 0.03(-0.23-0.29) | 802.97 | 989.49 | 23.23% | -0.63(-0.85 - -0.41) |
| Suriname | UDSD | 21637.7 | 39103.02 | 80.72% | 0.019(0.017-0.021) | 23.62 | 30.29 | 28.24% | -2.31(-2.63 - -1.99) | 1135.35 | 1653.34 | 45.62% | -1.35(-1.5 - -1.19) |
| Suriname | VAID | 23.22 | 52.77 | 127.26% | 0.88(0.77-0.98) | 1.49 | 3.28 | 120.13% | -0.19(-0.38-0) | 32.35 | 66.37 | 105.16% | -0.34(-0.51 - -0.16) |
| Sweden | Digestive diseases | 481614.16 | 617233.33 | 28.16% | 0.1(0.01-0.19) | 2893.97 | 3226.28 | 11.48% | -1.01(-1.08 - -0.94) | 73431.33 | 76634.32 | 4.36% | -0.79(-0.85 - -0.73) |
| Sweden | APED | 25140.91 | 30880.52 | 22.83% | 0.26(0.15-0.38) | 25.05 | 22.95 | -8.38% | -1.59(-1.77 - -1.41) | 776.5 | 714.8 | -7.95% | -0.68(-0.74 - -0.61) |
| Sweden | COCLD | 1238.32 | 1605.43 | 29.65% | 0.52(0.35-0.69) | 841.81 | 1068.87 | 26.97% | -0.29(-0.42 - -0.15) | 21706.7 | 23696.52 | 9.17% | -0.67(-0.82 - -0.52) |
| Sweden | GABD | 73114.74 | 107776.21 | 47.41% | 0.62(0.3-0.95) | 259.82 | 319.57 | 23.00% | -0.65(-0.87 - -0.43) | 7614.51 | 9097.05 | 19.47% | -0.19(-0.34 - -0.04) |
| Sweden | IFAH | 14994.88 | 16232.39 | 8.25% | -0.49(-0.57 - -0.41) | 87 | 86.74 | -0.30% | -1.34(-1.6 - -1.08) | 3306.77 | 3000.03 | -9.28% | -1.09(-1.2 - -0.99) |
| Sweden | IFBD | 2295.59 | 3173.4 | 38.24% | 0.67(0.62-0.71) | 106.69 | 94.55 | -11.38% | -3.16(-4.26 - -2.06) | 6157.65 | 7116.9 | 15.58% | -0.35(-0.54 - -0.16) |
| Sweden | PACA | 4146.73 | 6186.63 | 49.19% | 0.43(0.31-0.54) | 130.39 | 184.02 | 41.13% | -0.09(-0.25-0.07) | 3390.31 | 3804.36 | 12.21% | -0.61(-0.78 - -0.45) |
| Sweden | PIIO | 27197.98 | 33233.16 | 22.19% | -0.23(-0.26 - -0.21) | 255.25 | 411.91 | 61.38% | 0.09(0.01-0.18) | 4169.51 | 5546.89 | 33.03% | -0.29(-0.34 - -0.24) |
| Sweden | UDSD | 328908.73 | 414691.76 | 26.08% | 0.01(-0.046-0.066) | 647.6 | 394.08 | -39.15% | -3.45(-3.55 - -3.34) | 17829.42 | 14620.79 | -18.00% | -1.49(-1.56 - -1.43) |
| Sweden | VAID | 4576.29 | 3453.84 | -24.53% | -1.95(-2.32 - -1.58) | 331.74 | 267.69 | -19.31% | -2.3(-2.41 - -2.19) | 4878.87 | 3588.73 | -26.44% | -2.4(-2.49 - -2.31) |
| Switzerland | Digestive diseases | 283094.09 | 401157.96 | 41.70% | 0.15(0.01-0.28) | 1845.65 | 2972.13 | 61.03% | -0.73(-1.02 - -0.45) | 50370.3 | 62977.41 | 25.03% | -1.05(-1.27 - -0.83) |
| Switzerland | APED | 20395.7 | 24236.27 | 18.83% | 0.08(0.02-0.13) | 14.56 | 19.66 | 35.03% | -1.42(-1.85 - -0.98) | 534.89 | 568.13 | 6.21% | -0.91(-1.05 - -0.76) |
| Switzerland | COCLD | 1468.48 | 1747.12 | 18.97% | -0.33(-0.47 - -0.19) | 884.12 | 1065.05 | 20.46% | -1.44(-1.64 - -1.25) | 24325.78 | 24272.5 | -0.22% | -1.92(-2.09 - -1.75) |
| Switzerland | GABD | 34910.15 | 54290.36 | 55.51% | 0.09(-0.71-0.89) | 94.79 | 218.54 | 130.55% | 0.54(0.3-0.77) | 3837.98 | 5943 | 54.85% | -0.23(-0.85-0.4) |
| Switzerland | IFAH | 10440.22 | 16571.48 | 58.73% | 0.53(-0.07-1.13) | 39.59 | 86.35 | 118.11% | 0.28(-0.06-0.63) | 2408.81 | 3591.2 | 49.09% | 0(-0.49-0.5) |
| Switzerland | IFBD | 852.99 | 1108.47 | 29.95% | -0.01(-0.14-0.12) | 72.91 | 123.22 | 69.00% | -1.03(-1.8 - -0.26) | 2816.56 | 3806.9 | 35.16% | -0.56(-0.82 - -0.31) |
| Switzerland | PACA | 2138.71 | 3200.58 | 49.65% | -0.11(-0.21 - -0.01) | 70.28 | 105.83 | 50.58% | -0.84(-1.13 - -0.54) | 1735.45 | 2145.7 | 23.64% | -1.18(-1.38 - -0.98) |
| Switzerland | PIIO | 12008.32 | 20057.63 | 67.03% | 0.26(0.07-0.45) | 128.62 | 413.9 | 221.81% | 1.78(1.35-2.21) | 2028.58 | 4880.69 | 140.60% | 0.97(0.65-1.29) |
| Switzerland | UDSD | 199036.52 | 276646.96 | 38.99% | 0.142(0.066-0.218) | 237.98 | 236.25 | -0.73% | -2.77(-3.11 - -2.44) | 8051.77 | 8948.28 | 11.13% | -1.05(-1.12 - -0.97) |
| Switzerland | VAID | 1842.99 | 3299.1 | 79.01% | 1.24(1.03-1.46) | 120.44 | 251.28 | 108.64% | 0.2(-0.22-0.61) | 1840.78 | 3327.21 | 80.75% | -0.01(-0.34-0.32) |
| Syrian Arab Republic | Digestive diseases | 498213.97 | 822854.5 | 65.16% | 0.13(0.13-0.14) | 1911.14 | 2595.63 | 35.82% | -1.24(-1.47 - -1.01) | 81169.12 | 92841.92 | 14.38% | -1.27(-1.48 - -1.05) |
| Syrian Arab Republic | APED | 30463.51 | 45532.65 | 49.47% | 1.01(0.89-1.13) | 55.95 | 28.21 | -49.58% | -3.68(-4.03 - -3.34) | 3599.4 | 1722.67 | -52.14% | -3.23(-3.56 - -2.9) |
| Syrian Arab Republic | COCLD | 1902.83 | 3557.58 | 86.96% | 0.67(0.64-0.7) | 1354.29 | 2023.57 | 49.42% | -0.94(-1.18 - -0.69) | 46469.5 | 55831.88 | 20.15% | -1.25(-1.5 - -1) |
| Syrian Arab Republic | GABD | 16015.71 | 34130.71 | 113.11% | 0.5(0.47-0.53) | 54.34 | 76.7 | 41.15% | -1.55(-1.78 - -1.32) | 3091.54 | 4619.72 | 49.43% | -1.23(-1.39 - -1.06) |
| Syrian Arab Republic | IFAH | 6469.55 | 10121.1 | 56.44% | 1.23(1.15-1.3) | 11.83 | 12.1 | 2.28% | -1.62(-1.83 - -1.41) | 2225.76 | 2604.86 | 17.03% | -0.19(-0.36 - -0.03) |
| Syrian Arab Republic | IFBD | 241.3 | 448.84 | 86.01% | 0.26(0.05-0.46) | 23.35 | 36.71 | 57.22% | -1(-1.2 - -0.8) | 1090.62 | 1653.12 | 51.58% | -0.46(-0.53 - -0.39) |
| Syrian Arab Republic | PACA | 2130.32 | 3542.47 | 66.29% | -0.16(-0.18 - -0.14) | 47.24 | 69.51 | 47.14% | -1.53(-1.76 - -1.3) | 1370.64 | 1887.9 | 37.74% | -1.64(-1.86 - -1.41) |
| Syrian Arab Republic | PIIO | 8183.14 | 14681.9 | 79.42% | 1.38(1.18-1.59) | 186.8 | 170.29 | -8.84% | -2.23(-2.52 - -1.94) | 7780.08 | 4294.63 | -44.80% | -2.57(-2.87 - -2.26) |
| Syrian Arab Republic | UDSD | 432263.92 | 709479.13 | 64.13% | 0.035(0.026-0.043) | 129.95 | 92.88 | -28.53% | -3.42(-3.69 - -3.15) | 13865.45 | 17850.84 | 28.74% | -0.92(-1.02 - -0.81) |
| Syrian Arab Republic | VAID | 543.69 | 1360.12 | 150.16% | 1.54(1.44-1.63) | 21.98 | 42.09 | 91.49% | -0.53(-0.67 - -0.39) | 449.38 | 872.22 | 94.09% | -0.62(-0.76 - -0.47) |
| Taiwan (China) | Digestive diseases | 676501.4 | 1037960.91 | 53.43% | -0.17(-0.19 - -0.15) | 8444.59 | 11755.6 | 39.21% | -2.86(-3.1 - -2.62) | 263458.62 | 309747.57 | 17.57% | -2.37(-2.57 - -2.18) |
| Taiwan (China) | APED | 24708.95 | 34571.05 | 39.91% | 0.45(0.39-0.5) | 28.32 | 46.99 | 65.93% | -1.89(-2.05 - -1.73) | 1056.42 | 1259.16 | 19.19% | -1.29(-1.43 - -1.16) |
| Taiwan (China) | COCLD | 9075.23 | 8204.77 | -9.59% | -2.13(-2.4 - -1.87) | 5646.79 | 7287.14 | 29.05% | -2.74(-2.98 - -2.5) | 175794.65 | 199865.26 | 13.69% | -2.44(-2.67 - -2.21) |
| Taiwan ( China) | GABD | 51692.07 | 80781.15 | 56.27% | -0.21(-0.38 - -0.04) | 430.36 | 923.31 | 114.54% | -2(-2.15 - -1.84) | 13266.88 | 18827.09 | 41.91% | -1.92(-2.06 - -1.77) |
| Taiwan (China) | IFAH | 17879.73 | 27454.52 | 53.55% | -0.52(-0.89 - -0.16) | 77.64 | 101.04 | 30.14% | -4.73(-5.41 - -4.05) | 5044.52 | 5766.53 | 14.31% | -2.06(-2.41 - -1.71) |
| Taiwan (China) | IFBD | 149.36 | 469.55 | 214.37% | 3.87(3.46-4.29) | 100.85 | 199.14 | 97.46% | -2.08(-3.37 - -0.77) | 2623.11 | 4337.77 | 65.37% | -0.85(-1.78-0.1) |
| Taiwan (China) | PACA | 9446.59 | 20027.35 | 112.01% | 0.58(0.45-0.7) | 176.55 | 342.97 | 94.26% | -1.52(-2.06 - -0.97) | 6454.26 | 10116.7 | 56.74% | -1.31(-1.85 - -0.76) |
| Taiwan (China) | PIIO | 56776.51 | 55864.26 | -1.61% | -2.42(-2.63 - -2.63) | 284.11 | 716.34 | 152.13% | -1.34(-1.42 - -1.25) | 8178.54 | 11450.21 | 40.00% | -1.62(-1.71 - -1.54) |
| Taiwan (China) | UDSD | 504367.16 | 801127.57 | 58.84% | 0.029(-0.025-0.083) | 1382.63 | 891.35 | -35.53% | -6.91(-7.46 - -6.35) | 42138.77 | 35998.83 | -14.57% | -3.82(-4.11 - -3.54) |
| Taiwan (China) | VAID | 2405.81 | 9460.69 | 293.24% | 2.48(1.72-3.24) | 57.47 | 576.05 | 902.35% | 4.31(3.26-5.37) | 1250.79 | 8959.2 | 616.28% | 3.92(2.94-4.91) |
| Tajikistan | Digestive diseases | 208105.09 | 438055.78 | 110.50% | 0.06(0.05-0.08) | 1521.3 | 2656.87 | 74.64% | 0.47(0.23-0.71) | 80401.44 | 117060.61 | 45.60% | -0.3(-0.44 - -0.16) |
| Tajikistan | APED | 13499.19 | 24344.5 | 80.34% | 0.03(-0.09-0.14) | 23.13 | 20.37 | -11.93% | -2.49(-2.83 - -2.16) | 1493.18 | 1310.03 | -12.27% | -2.85(-3.22 - -2.47) |
| Tajikistan | COCLD | 1251.95 | 4716.53 | 276.73% | 2.4(2.31-2.48) | 927.16 | 2187.7 | 135.96% | 1.01(0.76-1.26) | 36500.08 | 81344.23 | 122.86% | 0.37(0.16-0.59) |
| Tajikistan | GABD | 13119.06 | 29375.55 | 123.92% | 0.29(0.24-0.34) | 29.42 | 37.76 | 28.35% | -0.21(-0.33 - -0.08) | 2723.84 | 4636.13 | 70.21% | -0.62(-0.68 - -0.56) |
| Tajikistan | IFAH | 5939.89 | 12745.09 | 114.57% | 0.98(0.59-1.38) | 25.51 | 6.67 | -73.85% | -1.55(-1.89 - -1.21) | 3518.87 | 3391.42 | -3.62% | -0.32(-0.64-0.01) |
| Tajikistan | IFBD | 225.24 | 518.41 | 130.16% | 0.16(0.09-0.23) | 20.6 | 18.29 | -11.21% | -0.79(-0.88 - -0.7) | 1691.06 | 1646.14 | -2.66% | -0.89(-1.1 - -0.69) |
| Tajikistan | PACA | 1031.65 | 2148.08 | 108.22% | 0.01(-0.04-0.07) | 20.45 | 35 | 71.15% | 0.66(0.46-0.86) | 633.24 | 1217.37 | 92.24% | -0.17(-0.36-0.02) |
| Tajikistan | PIIO | 2517.21 | 4326.8 | 71.89% | -0.09(-0.13 - -0.06) | 278.45 | 107.02 | -61.56% | -1.75(-2.07 - -1.42) | 22127.1 | 6437.93 | -70.90% | -2.92(-3.48 - -2.36) |
| Tajikistan | UDSD | 170017.14 | 358936.11 | 111.12% | 0.004(0.001-0.007) | 155.42 | 176.16 | 13.34% | -2.1(-2.68 - -1.52) | 9411.63 | 14311.74 | 52.06% | -1.84(-2.2 - -1.47) |
| Tajikistan | VAID | 503.75 | 944.72 | 87.54% | 0.93(0.68-1.18) | 14.65 | 29.03 | 98.16% | 2.09(1.93-2.25) | 429.38 | 758.31 | 76.61% | 1.38(1.26-1.49) |
| Thailand | Digestive diseases | 1750330.61 | 3025738.67 | 72.87% | 0.11(0.11-0.12) | 17334.16 | 32634.79 | 88.27% | -1.32(-1.42 - -1.21) | 648186.45 | 969435.9 | 49.56% | -1.33(-1.45 - -1.21) |
| Thailand | APED | 145340.13 | 163024.54 | 12.17% | 0.08(-0.2-0.35) | 212.94 | 123.24 | -42.12% | -4.52(-4.84 - -4.2) | 13478.34 | 6231.64 | -53.77% | -4.11(-4.45 - -3.77) |
| Thailand | COCLD | 12333.19 | 20264.85 | 64.31% | 0.39(0.32-0.46) | 10445.93 | 19603.4 | 87.67% | -0.95(-1.09 - -0.81) | 382413.61 | 615180.85 | 60.87% | -0.97(-1.1 - -0.83) |
| Thailand | GABD | 144329.46 | 229590.91 | 59.07% | -0.6(-0.8 - -0.39) | 1367.76 | 3070.96 | 124.52% | -1.47(-1.66 - -1.28) | 49362.65 | 70930.66 | 43.69% | -2.05(-2.24 - -1.86) |
| Thailand | IFAH | 39829.28 | 122114.31 | 206.59% | 1.31(1.26-1.37) | 213.83 | 424.61 | 98.57% | -1.23(-1.56 - -0.91) | 15620.45 | 28482.9 | 82.34% | -0.51(-0.63 - -0.38) |
| Thailand | IFBD | 183.24 | 373.23 | 103.68% | 1.11(1.03-1.18) | 71.76 | 110.55 | 54.06% | -2.46(-2.78 - -2.14) | 2898.7 | 3271.7 | 12.87% | -2.4(-2.71 - -2.09) |
| Thailand | PACA | 11374.61 | 20654.33 | 81.58% | -0.05(-0.07 - -0.04) | 685.35 | 1254.48 | 83.04% | -0.92(-1.11 - -0.72) | 27377.54 | 40150.38 | 46.65% | -1.08(-1.29 - -0.87) |
| Thailand | PIIO | 69135.6 | 192327.19 | 178.19% | 1.55(1.42-1.69) | 1263.89 | 2819.44 | 123.08% | -0.9(-1.11 - -0.7) | 46934.21 | 54910.76 | 17.00% | -1.95(-2.11 - -1.79) |
| Thailand | UDSD | 1324940.08 | 2267860.67 | 71.17% | 0.033(0.026-0.041) | 748.55 | 1888.2 | 152.25% | -0.27(-0.71-0.18) | 53473.15 | 93101.43 | 74.11% | -0.29(-0.46 - -0.13) |
| Thailand | VAID | 2865.02 | 9528.63 | 232.59% | 1.34(1.28-1.4) | 209.27 | 731.08 | 249.35% | -0.2(-0.35 - -0.05) | 4492.92 | 11682.04 | 160.01% | -0.52(-0.68 - -0.37) |
| Timor-Leste | Digestive diseases | 18568.94 | 36426.2 | 96.17% | 0.18(0.16-0.19) | 225.21 | 431.43 | 91.56% | -0.92(-1.05 - -0.79) | 9439.14 | 13825.31 | 46.47% | -1.21(-1.39 - -1.04) |
| Timor-Leste | APED | 1709.75 | 3865.06 | 126.06% | 0.94(0.71-1.17) | 4.23 | 4.74 | 12.06% | -1.79(-1.99 - -1.59) | 268.91 | 259.34 | -3.56% | -2.1(-2.32 - -1.87) |
| Timor-Leste | COCLD | 140.43 | 275.45 | 96.15% | 0.73(0.69-0.78) | 103.6 | 210.27 | 102.96% | -0.37(-0.51 - -0.23) | 4116.53 | 6606.3 | 60.48% | -0.77(-0.96 - -0.58) |
| Timor-Leste | GABD | 936.19 | 2315.54 | 147.34% | 1.16(1.12-1.2) | 6.62 | 17.68 | 167.07% | 0.25(0.14-0.37) | 417.8 | 738.42 | 76.74% | -0.49(-0.58 - -0.39) |
| Timor-Leste | IFAH | 406.97 | 1171.35 | 187.82% | 1.61(1.43-1.79) | 3 | 6.14 | 104.67% | 0.1(0.04-0.16) | 266.05 | 444.27 | 66.99% | -0.05(-0.13-0.02) |
| Timor-Leste | IFBD | 2.3 | 5.83 | 153.48% | 1.5(1.36-1.65) | 1.33 | 2.75 | 106.77% | -0.57(-0.7 - -0.44) | 58.66 | 86.35 | 47.20% | -0.95(-1.13 - -0.77) |
| Timor-Leste | PACA | 129.98 | 248.49 | 91.18% | 0.01(0-0.02) | 5.53 | 10.72 | 93.85% | -0.37(-0.55 - -0.19) | 233.96 | 358.63 | 53.29% | -0.76(-1 - -0.53) |
| Timor-Leste | PIIO | 375.42 | 963.71 | 156.71% | 1.37(1.26-1.47) | 13.17 | 37.39 | 183.83% | 0.87(0.78-0.96) | 642.64 | 1206.2 | 87.70% | 0.45(0.37-0.53) |
| Timor-Leste | UDSD | 14848.82 | 27522.11 | 85.35% | -0.067(-0.081 - -0.053) | 82.78 | 127.16 | 53.61% | -2.15(-2.33 - -1.97) | 3225.73 | 3733.56 | 15.74% | -2.43(-2.65 - -2.22) |
| Timor-Leste | VAID | 19.07 | 58.66 | 207.60% | 1.8(1.67-1.93) | 0.91 | 3.93 | 331.87% | 1.18(1.06-1.3) | 23.6 | 80.06 | 239.24% | 0.92(0.81-1.04) |
| Togo | Digestive diseases | 122125.02 | 329945.22 | 170.17% | 0.11(0.09-0.13) | 1137.34 | 2330.09 | 104.87% | -0.92(-1 - -0.85) | 50132.1 | 96306.17 | 92.10% | -0.85(-0.91 - -0.78) |
| Togo | APED | 4500.56 | 12512.25 | 178.02% | 0.92(0.71-1.13) | 18.83 | 20.02 | 6.32% | -1.79(-2.04 - -1.54) | 1168.65 | 967.81 | -17.19% | -2.69(-3 - -2.37) |
| Togo | COCLD | 736.91 | 1880.45 | 155.18% | 0.28(0.17-0.39) | 695.96 | 1272.07 | 82.78% | -1.67(-1.89 - -1.46) | 26060.83 | 46280.46 | 77.59% | -1.52(-1.73 - -1.31) |
| Togo | GABD | 1065.72 | 2905.68 | 172.65% | 0.63(0.56-0.7) | 28.39 | 81.43 | 186.83% | 0.89(0.78-0.99) | 1304.61 | 2956.16 | 126.59% | 0.51(0.41-0.62) |
| Togo | IFAH | 3824.84 | 12693.41 | 231.87% | 1.63(1.45-1.81) | 18.53 | 44.47 | 139.99% | 0.18(-0.02-0.38) | 1964.69 | 4734.72 | 140.99% | 0.66(0.52-0.79) |
| Togo | IFBD | 22.08 | 74.27 | 236.37% | 0.58(0.43-0.73) | 8.75 | 21.2 | 142.29% | -0.05(-0.31-0.21) | 457.07 | 940.34 | 105.73% | -0.23(-0.48-0.01) |
| Togo | PACA | 527.37 | 1451.23 | 175.18% | 0.15(0.13-0.17) | 45.43 | 171.03 | 276.47% | 0.89(0.71-1.08) | 1790.97 | 6649.08 | 271.26% | 1.06(0.87-1.25) |
| Togo | PIIO | 2803.81 | 7754.51 | 176.57% | 0.61(0.47-0.74) | 126.73 | 302.79 | 138.93% | 0.28(0.09-0.48) | 6789.34 | 12416.84 | 82.89% | 0(-0.22-0.23) |
| Togo | UDSD | 108464.73 | 290192.19 | 167.55% | 0.028(0.022-0.034) | 135.15 | 289.18 | 113.97% | -0.26(-0.43 - -0.09) | 7793.2 | 16739.59 | 114.80% | -0.29(-0.41 - -0.17) |
| Togo | VAID | 179 | 481.24 | 168.85% | 0.9(0.78-1.03) | 11.4 | 31.31 | 174.65% | 0.9(0.75-1.06) | 521.68 | 981.16 | 88.08% | 0.34(0.12-0.56) |
| Tokelau | Digestive diseases | 41.27 | 40.61 | -1.58% | 0.04(0.02-0.06) | 0.65 | 0.41 | -37.38% | -1.52(-1.58 - -1.45) | 19.4 | 12.52 | -35.44% | -1.48(-1.54 - -1.42) |
| Tokelau | APED | 1.51 | 1.59 | 5.30% | 0.73(0.66-0.81) | 0.01 | 0 | -100.00% | -2.4(-2.48 - -2.33) | 0.22 | 0.11 | -50.00% | -2.17(-2.23 - -2.12) |
| Tokelau | COCLD | 0.17 | 0.16 | -5.88% | -0.06(-0.11 - -0.02) | 0.31 | 0.22 | -29.03% | -1.34(-1.42 - -1.25) | 9.83 | 6.75 | -31.33% | -1.41(-1.5 - -1.33) |
| Tokelau | GABD | 2.07 | 2.25 | 8.70% | 0.31(0.27-0.36) | 0.05 | 0.04 | -20.00% | -0.58(-0.62 - -0.53) | 1.36 | 1.03 | -24.26% | -0.78(-0.81 - -0.74) |
| Tokelau | IFAH | 1.04 | 1.27 | 22.12% | 1.23(1.14-1.33) | 0.01 | 0.01 | 0.00% | -1.53(-1.74 - -1.33) | 0.53 | 0.42 | -20.75% | -0.48(-0.61 - -0.35) |
| Tokelau | IFBD | 0.01 | 0.01 | 0.00% | 0.57(0.34-0.8) | 0.01 | 0.01 | 0.00% | -1.72(-1.88 - -1.57) | 0.36 | 0.21 | -41.67% | -1.68(-1.79 - -1.56) |
| Tokelau | PACA | 0.35 | 0.34 | -2.86% | -0.01(-0.02-0) | 0.02 | 0.01 | -50.00% | -0.68(-0.71 - -0.65) | 0.56 | 0.45 | -19.64% | -0.75(-0.77 - -0.73) |
| Tokelau | PIIO | 0.98 | 1.13 | 16.00% | 1.2(1.17-1.22) | 0.04 | 0.03 | -25.13% | -0.6(-0.64 - -0.55) | 0.9 | 0.6 | -33.64% | -0.77(-0.79 - -0.74) |
| Tokelau | UDSD | 35.08 | 33.77 | -3.73% | -0.071(-0.084 - -0.059) | 0.17 | 0.07 | -58.82% | -2.95(-3.02 - -2.88) | 4.93 | 2.34 | -52.54% | -2.46(-2.53 - -2.4) |
| Tokelau | VAID | 0.07 | 0.09 | 28.57% | 1.42(1.38-1.47) | 0.01 | 0.01 | 0.00% | 0.65(0.53-0.76) | 0.18 | 0.19 | 5.56% | 0.55(0.44-0.66) |
| Tonga | Digestive diseases | 2322.78 | 2811.23 | 21.03% | -0.03(-0.05 - -0.02) | 34.08 | 36.97 | 8.46% | -1.19(-1.3 - -1.08) | 1129.15 | 1105.06 | -2.13% | -1.1(-1.18 - -1.01) |
| Tonga | APED | 99.84 | 121.58 | 21.77% | 0.43(0.37-0.48) | 0.31 | 0.3 | -3.23% | -1.49(-1.63 - -1.36) | 11.93 | 10.75 | -9.89% | -1.2(-1.35 - -1.05) |
| Tonga | COCLD | 15.9 | 18.54 | 16.60% | -0.43(-0.52 - -0.34) | 17.85 | 19 | 6.44% | -1.01(-1.09 - -0.92) | 604.8 | 592.14 | -2.09% | -1.03(-1.1 - -0.96) |
| Tonga | GABD | 127.55 | 167.38 | 31.23% | 0.09(0.06-0.12) | 2.07 | 2.92 | 41.06% | -0.4(-0.52 - -0.28) | 67.13 | 81.38 | 21.23% | -0.42(-0.51 - -0.34) |
| Tonga | IFAH | 69.58 | 94.85 | 36.32% | 0.25(0.16-0.34) | 0.59 | 0.67 | 13.56% | -1.19(-1.33 - -1.04) | 31.79 | 34.56 | 8.71% | -0.57(-0.67 - -0.47) |
| Tonga | IFBD | 0.34 | 0.59 | 73.53% | 1.3(1.13-1.48) | 0.62 | 0.62 | 0.00% | -1.73(-1.92 - -1.54) | 21.23 | 18.84 | -11.26% | -1.63(-1.83 - -1.44) |
| Tonga | PACA | 19.17 | 24.37 | 27.13% | -0.02(-0.04 - -0.01) | 0.98 | 1.28 | 30.61% | -0.54(-0.68 - -0.39) | 32.09 | 39.15 | 22.00% | -0.39(-0.55 - -0.23) |
| Tonga | PIIO | 57.34 | 72.45 | 26.36% | 0.24(0.18-0.29) | 1.96 | 2.79 | 42.06% | -0.66(-0.74 - -0.59) | 61.66 | 63.38 | 2.79% | -0.7(-0.78 - -0.62) |
| Tonga | UDSD | 1928.45 | 2304.01 | 19.47% | -0.08(-0.092 - -0.067) | 8.14 | 7.03 | -13.64% | -2.27(-2.45 - -2.09) | 254.71 | 208.04 | -18.32% | -1.88(-2.01 - -1.75) |
| Tonga | VAID | 4.61 | 7.46 | 61.82% | 0.69(0.63-0.75) | 0.46 | 0.82 | 78.26% | 0.2(0.05-0.34) | 10.33 | 16.5 | 59.73% | 0.33(0.18-0.49) |
| Trinidad and bago | Digestive diseases | 80782.81 | 122404.99 | 51.52% | 0.03(0.02-0.05) | 334.49 | 464.72 | 38.93% | -1.7(-1.91 - -1.49) | 13118.14 | 16208.81 | 23.56% | -1.38(-1.53 - -1.22) |
| Trinidad and bago | APED | 2735.51 | 3677.23 | 34.43% | 1.12(1.04-1.19) | 7.97 | 5.94 | -25.47% | -3.16(-3.56 - -2.75) | 365.81 | 228.45 | -37.55% | -2.67(-3.02 - -2.33) |
| Trinidad and bago | COCLD | 168.15 | 201.77 | 19.99% | -0.6(-0.66 - -0.53) | 149.98 | 187.51 | 25.02% | -1.83(-2.03 - -1.63) | 5004.27 | 5583.09 | 11.57% | -1.84(-2.03 - -1.66) |
| Trinidad and bago | GABD | 3272.59 | 5044.53 | 54.14% | 0.03(-0.02-0.07) | 15.85 | 21.06 | 32.87% | -1.92(-2.24 - -1.6) | 799.81 | 1016.52 | 27.10% | -1.31(-1.49 - -1.14) |
| Trinidad and bago | IFAH | 2613.89 | 3955.44 | 51.32% | 0.49(0.37-0.61) | 11.45 | 15.83 | 38.25% | -1.71(-1.92 - -1.49) | 1043.69 | 1226.04 | 17.47% | -0.78(-0.88 - -0.68) |
| Trinidad and bago | IFBD | 27.19 | 52.37 | 92.61% | 0.39(0.18-0.6) | 3.06 | 5.12 | 67.32% | -0.98(-1.25 - -0.72) | 143.46 | 215.09 | 49.93% | -0.66(-0.84 - -0.48) |
| Trinidad and bago | PACA | 289.7 | 447.46 | 54.46% | -0.05(-0.07 - -0.02) | 9.46 | 16.74 | 76.96% | -0.38(-0.55 - -0.2) | 356.94 | 558.62 | 56.50% | -0.33(-0.52 - -0.13) |
| Trinidad and bago | PIIO | 948.41 | 1593.76 | 68.05% | 0.77(0.7-0.84) | 32.36 | 48.56 | 50.05% | -1.55(-1.76 - -1.34) | 1039.96 | 1124.83 | 8.16% | -1.53(-1.76 - -1.31) |
| Trinidad and bago | UDSD | 70624.53 | 107243.91 | 51.85% | -0.032(-0.052 - -0.012) | 87.15 | 99.34 | 13.99% | -3(-3.4 - -2.6) | 3900.8 | 4878.55 | 25.07% | -1.64(-1.87 - -1.42) |
| Trinidad and bago | VAID | 102.84 | 188.5 | 83.29% | 0.27(0.22-0.31) | 10.36 | 14.55 | 40.44% | -2.4(-2.74 - -2.06) | 224.25 | 284.24 | 26.75% | -2.42(-2.77 - -2.07) |
| Tunisia | Digestive diseases | 388534.22 | 730627.55 | 88.05% | 0.11(0.1-0.11) | 1212.63 | 2174.53 | 79.32% | -1.05(-1.08 - -1.01) | 48811.95 | 69777.77 | 42.95% | -1.03(-1.06 - -0.99) |
| Tunisia | APED | 18152.3 | 32353.66 | 78.23% | 1.21(1.16-1.25) | 24.96 | 24.93 | -0.12% | -2.66(-2.69 - -2.62) | 1302.36 | 1041.76 | -20.01% | -2.08(-2.16 - -2) |
| Tunisia | COCLD | 1153.68 | 2680.87 | 132.38% | 0.61(0.53-0.68) | 743.77 | 1366.85 | 83.77% | -0.93(-0.97 - -0.9) | 23551.49 | 33910.04 | 43.98% | -1.15(-1.18 - -1.11) |
| Tunisia | GABD | 8618.23 | 20947.31 | 143.06% | -0.13(-0.44-0.19) | 42.04 | 133.18 | 216.79% | 0.65(0.5-0.79) | 1879.17 | 4001.86 | 112.96% | -0.33(-0.42 - -0.24) |
| Tunisia | IFAH | 4344.66 | 8835.79 | 103.37% | 1.01(0.98-1.03) | 9.45 | 16.41 | 73.65% | -1.25(-1.34 - -1.16) | 1404.94 | 2024.92 | 44.13% | -0.23(-0.29 - -0.17) |
| Tunisia | IFBD | 167.94 | 349.13 | 107.89% | 0.57(0.53-0.61) | 7.2 | 16.17 | 124.58% | -0.05(-0.15-0.06) | 568.84 | 1214.95 | 113.58% | 0.27(0.17-0.37) |
| Tunisia | PACA | 1735.49 | 3448.93 | 98.73% | -0.04(-0.05 - -0.03) | 32.9 | 76.56 | 132.71% | -0.31(-0.36 - -0.25) | 939.04 | 1815.84 | 93.37% | -0.46(-0.5 - -0.43) |
| Tunisia | PIIO | 7129.64 | 16669.4 | 133.80% | 1.27(1.23-1.31) | 100.58 | 147.83 | 46.98% | -0.91(-1.04 - -0.79) | 5006.14 | 3559.91 | -28.89% | -1.67(-1.86 - -1.48) |
| Tunisia | UDSD | 346731.81 | 643660.64 | 85.64% | 0.019(0.017-0.021) | 206.49 | 254.53 | 23.27% | -2.72(-2.75 - -2.68) | 12857.78 | 19178.97 | 49.16% | -1.13(-1.18 - -1.08) |
| Tunisia | VAID | 500.48 | 1681.8 | 236.04% | 1.7(1.65-1.75) | 23.01 | 63.98 | 178.05% | 0.04(0-0.09) | 473.94 | 1195.33 | 152.21% | 0.06(0.03-0.09) |
| Turkey | Digestive diseases | 3009233.37 | 5867857.12 | 95.00% | 0.37(0.33-0.4) | 9049.02 | 14115.57 | 55.99% | -1.21(-1.34 - -1.09) | 381381.56 | 481138.61 | 26.16% | -1.43(-1.49 - -1.36) |
| Turkey | APED | 118815.55 | 233991.78 | 96.94% | 1.56(1.52-1.61) | 138 | 104.79 | -24.07% | -3.4(-3.69 - -3.12) | 7059.62 | 5308.03 | -24.81% | -2.65(-2.95 - -2.35) |
| Turkey | COCLD | 6651.17 | 16568.32 | 149.10% | 1.5(1.35-1.65) | 4950.22 | 7349.26 | 48.46% | -1.56(-1.63 - -1.48) | 156613.78 | 172252.48 | 9.99% | -2.3(-2.39 - -2.21) |
| Turkey | GABD | 110557.57 | 272186 | 146.19% | 1.66(1.24-2.08) | 742.67 | 1499.29 | 101.88% | -0.46(-0.71 - -0.21) | 30520.09 | 46853.68 | 53.52% | -0.84(-1 - -0.68) |
| Turkey | IFAH | 31065.18 | 72796.65 | 134.34% | 1.84(1.72-1.95) | 229.14 | 295.65 | 29.03% | -0.22(-0.72-0.28) | 21262.11 | 19008.13 | -10.60% | -1.05(-1.22 - -0.88) |
| Turkey | IFBD | 2084.17 | 5960.59 | 185.99% | 1.27(1.06-1.48) | 260.66 | 328.99 | 26.21% | -1.87(-2.02 - -1.72) | 13909.52 | 22487.78 | 61.67% | -0.61(-0.7 - -0.51) |
| Turkey | PACA | 11822.23 | 23398.92 | 97.92% | -0.03(-0.05 - -0.01) | 456.48 | 832.41 | 82.35% | -0.92(-1.08 - -0.76) | 12874 | 17970.88 | 39.59% | -1.74(-1.91 - -1.57) |
| Turkey | PIIO | 39239.89 | 119917.61 | 205.60% | 2.63(2.41-2.85) | 776.82 | 1537.38 | 97.91% | 0.3(-0.05-0.66) | 32415.25 | 31739.98 | -2.08% | -0.92(-1.13 - -0.71) |
| Turkey | UDSD | 2685978.52 | 5112417.91 | 90.34% | 0.182(0.15-0.215) | 858.9 | 943.9 | 9.90% | -2.2(-2.6 - -1.8) | 87210.79 | 138746.39 | 59.09% | -0.5(-0.63 - -0.37) |
| Turkey | VAID | 3019.1 | 10619.33 | 251.74% | 0.93(0.7-1.16) | 333.17 | 681.89 | 104.67% | -0.59(-0.82 - -0.35) | 7789.58 | 12913.46 | 65.78% | -1.23(-1.38 - -1.07) |
| Turkmenistan | Digestive diseases | 150452.76 | 265230.08 | 76.29% | 0.09(0.07-0.1) | 1111.14 | 2748.69 | 147.37% | 0.66(0.41-0.91) | 50798.11 | 116709.62 | 129.75% | 1.01(0.79-1.23) |
| Turkmenistan | APED | 9383.96 | 12603 | 34.30% | 0.22(0.1-0.34) | 15.16 | 7.63 | -49.67% | -4.76(-5.68 - -3.82) | 970.07 | 514.23 | -46.99% | -3.98(-4.84 - -3.12) |
| Turkmenistan | COCLD | 1188.45 | 2843.11 | 139.23% | 1.12(1.01-1.23) | 845.58 | 2381.05 | 181.59% | 0.94(0.67-1.21) | 31262.15 | 92638.62 | 196.33% | 1.52(1.24-1.8) |
| Turkmenistan | GABD | 10099.78 | 19282.13 | 90.92% | 0.19(0.15-0.23) | 22.02 | 30.34 | 37.78% | -1.61(-1.84 - -1.38) | 1965.61 | 2942.8 | 49.71% | -0.99(-1.07 - -0.91) |
| Turkmenistan | IFAH | 4538.34 | 8550 | 88.39% | 1.05(0.74-1.36) | 35.59 | 25.63 | -27.99% | -2.11(-2.28 - -1.93) | 3413.93 | 2787.21 | -18.36% | -1.14(-1.22 - -1.06) |
| Turkmenistan | IFBD | 187.71 | 382.78 | 103.92% | 0.54(0.44-0.64) | 9.76 | 14.18 | 45.29% | -0.5(-0.78 - -0.21) | 765.2 | 1120.13 | 46.38% | -0.2(-0.38 - -0.02) |
| Turkmenistan | PACA | 750.16 | 1475.24 | 96.66% | 0.18(0.16-0.2) | 20.76 | 62.62 | 201.64% | 1.23(0.98-1.49) | 807.15 | 2425.16 | 200.46% | 1.46(1.23-1.69) |
| Turkmenistan | PIIO | 1875.61 | 3457.25 | 84.33% | 0.64(0.55-0.73) | 68.17 | 52.7 | -22.69% | -0.44(-0.71 - -0.17) | 5239.65 | 3146.8 | -39.94% | -0.54(-0.97 - -0.12) |
| Turkmenistan | UDSD | 122043.94 | 215890.49 | 76.90% | 0.018(0.014-0.022) | 73.25 | 123.24 | 68.25% | -1.06(-1.39 - -0.74) | 5384.02 | 9370.54 | 74.04% | -0.45(-0.63 - -0.26) |
| Turkmenistan | VAID | 384.81 | 746.06 | 93.88% | 1.42(1.2-1.63) | 10.94 | 29.27 | 167.55% | 0.98(0.76-1.21) | 287.18 | 709.33 | 147.00% | 0.97(0.75-1.2) |
| Tuvalu | Digestive diseases | 251.66 | 348.39 | 38.43% | 0.01(0-0.02) | 4.35 | 4.1 | -5.63% | -1.49(-1.62 - -1.36) | 155.33 | 138.55 | -10.80% | -1.47(-1.6 - -1.34) |
| Tuvalu | APED | 9.08 | 14.47 | 59.36% | 0.58(0.52-0.64) | 0.05 | 0.04 | -20.00% | -1.61(-1.73 - -1.5) | 2.11 | 1.6 | -24.17% | -1.64(-1.77 - -1.5) |
| Tuvalu | COCLD | 1.16 | 1.63 | 40.52% | 0.16(0.11-0.21) | 2.32 | 2.25 | -3.02% | -1.32(-1.47 - -1.17) | 82.6 | 77.2 | -6.54% | -1.33(-1.48 - -1.18) |
| Tuvalu | GABD | 12.94 | 19.48 | 50.54% | 0.27(0.22-0.32) | 0.26 | 0.33 | 26.92% | -0.6(-0.64 - -0.56) | 9.35 | 10.29 | 10.05% | -0.79(-0.84 - -0.74) |
| Tuvalu | IFAH | 7.03 | 10.17 | 44.67% | 0.54(0.39-0.69) | 0.07 | 0.07 | 0.00% | -1.24(-1.44 - -1.05) | 4.02 | 3.84 | -4.48% | -0.89(-1.11 - -0.66) |
| Tuvalu | IFBD | 0.04 | 0.08 | 100.00% | 1.23(1.17-1.3) | 0.07 | 0.07 | 0.00% | -1.6(-1.73 - -1.47) | 2.72 | 2.27 | -16.54% | -1.65(-1.76 - -1.55) |
| Tuvalu | PACA | 1.97 | 2.81 | 42.64% | 0.05(0.04-0.05) | 0.13 | 0.15 | 15.38% | -0.77(-0.84 - -0.7) | 4.48 | 5.27 | 17.63% | -0.57(-0.62 - -0.51) |
| Tuvalu | PIIO | 4.96 | 7.84 | 58.10% | 0.67(0.6-0.75) | 0.19 | 0.23 | 18.90% | -0.86(-0.98 - -0.73) | 6.68 | 5.69 | -14.86% | -1.23(-1.37 - -1.09) |
| Tuvalu | UDSD | 214.15 | 291.25 | 36.00% | -0.07(-0.08 - -0.06) | 1.1 | 0.76 | -30.91% | -2.58(-2.69 - -2.47) | 38.5 | 26.65 | -30.78% | -2.39(-2.52 - -2.27) |
| Tuvalu | VAID | 0.34 | 0.65 | 91.18% | 1.09(1.01-1.16) | 0.04 | 0.07 | 75.00% | 0(-0.12-0.12) | 1.04 | 1.59 | 52.88% | 0.08(-0.02-0.17) |
| Uganda | Digestive diseases | 581184.67 | 1469181.06 | 152.79% | 0.11(0.1-0.13) | 5074.48 | 9155.1 | 80.41% | -1.04(-1.23 - -0.85) | 199545.15 | 378620.77 | 89.74% | -1(-1.21 - -0.79) |
| Uganda | APED | 23060.06 | 78075.53 | 238.57% | 1.13(0.98-1.28) | 127.81 | 187.13 | 46.41% | -1.71(-1.95 - -1.47) | 7465.49 | 11100.16 | 48.69% | -1.59(-1.85 - -1.33) |
| Uganda | COCLD | 2791.94 | 6934.03 | 148.36% | -0.05(-0.12-0.03) | 3010.18 | 5188.95 | 72.38% | -1.33(-1.55 - -1.1) | 98378.19 | 180774.07 | 83.75% | -1.36(-1.62 - -1.1) |
| Uganda | GABD | 4851.19 | 13500.37 | 178.29% | 0.67(0.63-0.71) | 225.73 | 494.14 | 118.91% | -0.05(-0.17-0.06) | 6734.53 | 14550.19 | 116.05% | -0.22(-0.37 - -0.07) |
| Uganda | IFAH | 11652.55 | 31667.53 | 171.76% | 0.91(0.81-1.02) | 162.44 | 241.95 | 48.95% | -0.77(-0.9 - -0.64) | 11765.1 | 18266.67 | 55.26% | -0.52(-0.61 - -0.43) |
| Uganda | IFBD | 96.89 | 280.59 | 189.60% | 0.65(0.58-0.72) | 47.56 | 101.85 | 114.15% | -0.03(-0.17-0.1) | 2256.89 | 4600.43 | 103.84% | -0.02(-0.16-0.13) |
| Uganda | PACA | 2087.68 | 5035.37 | 141.19% | -0.04(-0.06 - -0.02) | 111.65 | 245.41 | 119.80% | -0.56(-0.73 - -0.39) | 3946.04 | 9396.94 | 138.14% | -0.49(-0.68 - -0.3) |
| Uganda | PIIO | 13919.16 | 39915.55 | 186.77% | 0.95(0.86-1.04) | 480.29 | 1027.38 | 113.91% | -0.07(-0.18-0.04) | 25014.09 | 51088.61 | 104.24% | 0.05(-0.06-0.15) |
| Uganda | UDSD | 522049.52 | 1291656.28 | 147.42% | 0.043(0.038-0.048) | 570.1 | 971.74 | 70.45% | -1.46(-1.77 - -1.14) | 32542.88 | 66635.91 | 104.76% | -0.94(-1.18 - -0.7) |
| Uganda | VAID | 675.68 | 2115.83 | 213.14% | 1.21(1.12-1.3) | 79.43 | 202.44 | 154.87% | 0.49(0.39-0.6) | 1983.6 | 4972.47 | 150.68% | 0.43(0.29-0.56) |
| Ukraine | Digestive diseases | 4106133.39 | 3850363.22 | -6.23% | 0.04(0.01-0.06) | 16845.71 | 30239.82 | 79.51% | 2.07(1.53-2.63) | 643404.06 | 1176626.82 | 82.88% | 2.3(1.73-2.88) |
| Ukraine | APED | 124026.87 | 100837.15 | -18.70% | 0.46(0.21-0.7) | 257.62 | 101.64 | -60.55% | -3.19(-4.01 - -2.36) | 10375.57 | 4510.75 | -56.53% | -2.25(-3.04 - -1.47) |
| Ukraine | COCLD | 10702.44 | 14401.21 | 34.56% | 2.03(1.74-2.31) | 7986.61 | 20141.02 | 152.18% | 3.45(2.58-4.33) | 252470.35 | 759649.44 | 200.89% | 4.08(3.08-5.1) |
| Ukraine | GABD | 659547.12 | 604578.61 | -8.33% | -0.16(-0.21 - -0.1) | 988.2 | 528.08 | -46.56% | -2.83(-3.22 - -2.43) | 69857.8 | 48996.41 | -29.86% | -1.28(-1.41 - -1.15) |
| Ukraine | IFAH | 141172.64 | 129659.47 | -8.16% | 0.44(0.34-0.55) | 166 | 174.66 | 5.22% | -0.79(-1.01 - -0.57) | 24344.02 | 20659.74 | -15.13% | -0.1(-0.2-0) |
| Ukraine | IFBD | 3459.28 | 3625.56 | 4.81% | 0.19(0.1-0.28) | 230.48 | 259.55 | 12.61% | 0.17(-0.1-0.44) | 13419.08 | 14197.04 | 5.80% | 0.34(0.12-0.56) |
| Ukraine | PACA | 43371.56 | 46530.66 | 7.28% | 0.29(0.25-0.34) | 1889.23 | 2819.09 | 49.22% | 1.2(0.84-1.55) | 69885.12 | 110269.44 | 57.79% | 1.5(1.11-1.89) |
| Ukraine | PIIO | 66745.79 | 62196.7 | -6.82% | 0.07(0.04-0.1) | 744.44 | 772 | 3.70% | -0.72(-0.84 - -0.59) | 21857.3 | 18033.25 | -17.50% | -1.11(-1.31 - -0.91) |
| Ukraine | UDSD | 3027332.72 | 2861658.84 | -5.47% | 0.023(0.015-0.031) | 2113.28 | 2177.15 | 3.02% | -0.45(-0.84 - -0.05) | 127921.11 | 130738.91 | 2.20% | -0.04(-0.27-0.18) |
| Ukraine | VAID | 29774.96 | 26875.01 | -9.74% | -0.44(-0.51 - -0.37) | 2093.65 | 2630.2 | 25.63% | 0.02(-0.12-0.16) | 39122.48 | 48824.07 | 24.80% | 0.22(0.03-0.42) |
| United Arab Emirates | Digestive diseases | 91118.75 | 620584.54 | 581.07% | 0.06(0.04-0.07) | 113.62 | 734.48 | 546.42% | -1.56(-1.99 - -1.12) | 6576.26 | 44358.89 | 574.53% | -1.19(-1.45 - -0.93) |
| United Arab Emirates | APED | 3883.44 | 25789.59 | 564.09% | 1.19(1.1-1.27) | 3.21 | 14.73 | 358.88% | -1.89(-2.33 - -1.46) | 202.45 | 945.07 | 366.82% | -1.33(-1.48 - -1.19) |
| United Arab Emirates | COCLD | 219.46 | 2019.06 | 820.01% | 0.09(0-0.17) | 71.45 | 505.62 | 607.66% | -1.37(-1.78 - -0.96) | 2525.95 | 18427.71 | 629.54% | -1.31(-1.62 - -1) |
| United Arab Emirates | GABD | 2216.09 | 18228.78 | 722.56% | 0.14(0.08-0.2) | 3.87 | 24.73 | 539.02% | -1(-1.69 - -0.3) | 378.76 | 2570.35 | 578.62% | -0.86(-1.22 - -0.51) |
| United Arab Emirates | IFAH | 1100.55 | 7704.89 | 600.09% | 0.52(0.37-0.68) | 0.48 | 2.5 | 420.83% | -1.87(-2.3 - -1.44) | 291.31 | 1619.96 | 456.09% | -0.39(-0.47 - -0.3) |
| United Arab Emirates | IFBD | 58.23 | 520.36 | 793.63% | 0.76(0.59-0.94) | 1.23 | 12.8 | 940.65% | 0.72(0.26-1.2) | 144.24 | 1400.7 | 871.09% | 0.73(0.52-0.94) |
| United Arab Emirates | PACA | 370.6 | 2635.78 | 611.22% | -0.02(-0.04-0.01) | 5.71 | 40.17 | 603.50% | -1.28(-1.76 - -0.8) | 243.75 | 1768.29 | 625.45% | -1.07(-1.35 - -0.79) |
| United Arab Emirates | PIIO | 1162.9 | 7663.12 | 558.96% | 0.42(0.33-0.52) | 4.11 | 28.84 | 601.92% | -0.36(-0.69 - -0.03) | 205.62 | 1215.2 | 490.98% | -0.3(-0.52 - -0.08) |
| United Arab Emirates | UDSD | 81973.43 | 554888.03 | 576.91% | -0.014(-0.023 - -0.006) | 18.26 | 50.72 | 177.77% | -3.43(-3.98 - -2.88) | 2358.64 | 14189.45 | 501.59% | -1.66(-1.84 - -1.48) |
| United Arab Emirates | VAID | 134.04 | 1134.94 | 746.72% | 0.73(0.6-0.86) | 2.69 | 19.32 | 618.22% | -1.02(-1.58 - -0.47) | 85.22 | 695.47 | 716.09% | -0.91(-1.37 - -0.44) |
| United Kingdom | Digestive diseases | 4075174.53 | 5702060.5 | 39.92% | 0.25(0.13-0.37) | 21015.89 | 33215.06 | 58.05% | 0.34(0.13-0.54) | 580145.22 | 882863.65 | 52.18% | 0.26(0.05-0.46) |
| United Kingdom | APED | 105166.83 | 123137.27 | 17.09% | 0.18(0.17-0.19) | 157.78 | 191.8 | 21.56% | -0.53(-0.96 - -0.09) | 4712.85 | 4878.54 | 3.52% | -0.58(-0.85 - -0.32) |
| United Kingdom | COCLD | 8657.36 | 16129.72 | 86.31% | 1.46(1.19-1.73) | 4899.18 | 10031.46 | 104.76% | 1.5(1.05-1.96) | 137680.78 | 283251.01 | 105.73% | 1.57(1.07-2.07) |
| United Kingdom | GABD | 611933.46 | 1416872.44 | 131.54% | 1.09(0.76-1.42) | 1234.75 | 2943.78 | 138.41% | 2.01(1.83-2.2) | 71403.5 | 156249.62 | 118.83% | 0.16(-0.37-0.69) |
| United Kingdom | IFAH | 156379.69 | 204313.11 | 30.65% | 1.18(0.51-1.87) | 1015.94 | 1242.44 | 22.29% | -0.61(-0.8 - -0.42) | 37189.19 | 37959.07 | 2.07% | -1.05(-1.12 - -0.99) |
| United Kingdom | IFBD | 12548.06 | 16687.86 | 32.99% | 0.54(0.47-0.61) | 856.01 | 1793.33 | 109.50% | 1.22(0.84-1.59) | 37500.14 | 55170.62 | 47.12% | 0.41(0.25-0.57) |
| United Kingdom | PACA | 21086.28 | 31122.18 | 47.59% | 0.36(0.25-0.46) | 1046.31 | 1523.87 | 45.64% | 0.08(-0.11-0.26) | 25304.74 | 34238.31 | 35.30% | 0.07(-0.1-0.24) |
| United Kingdom | PIIO | 127706.46 | 162078.99 | 26.92% | 0.12(-0.13-0.36) | 1647.17 | 3319.88 | 101.55% | 1.05(0.85-1.26) | 26841.32 | 45213.91 | 68.45% | 0.69(0.49-0.9) |
| United Kingdom | UDSD | 3004881.86 | 3701706.9 | 23.19% | -0.047(-0.168-0.073) | 5482.69 | 3377.91 | -38.39% | -3.65(-3.91 - -3.38) | 160243.32 | 138588.44 | -13.51% | -1.44(-1.59 - -1.29) |
| United Kingdom | VAID | 26814.54 | 30012.02 | 11.92% | -0.27(-0.79-0.25) | 1824.75 | 3744.94 | 105.23% | 1.31(1.17-1.44) | 31331.36 | 55098.32 | 75.86% | 0.91(0.79-1.02) |
| United Republic of Tanzania | Digestive diseases | 901431.81 | 2203020.69 | 144.39% | 0.12(0.11-0.14) | 8351.89 | 14521.96 | 73.88% | -0.72(-0.98 - -0.45) | 353692.82 | 603319.02 | 70.58% | -0.69(-0.92 - -0.46) |
| United Republic of Tanzania | APED | 34784.14 | 105166.52 | 202.34% | 1.11(0.97-1.24) | 214.41 | 297.7 | 38.85% | -0.78(-0.98 - -0.59) | 13909.16 | 17873.06 | 28.50% | -0.88(-1.06 - -0.7) |
| United Republic of Tanzania | COCLD | 4065.49 | 10010.96 | 146.24% | 0.1(0.02-0.18) | 5355.73 | 8546.67 | 59.58% | -1.15(-1.46 - -0.83) | 184503.66 | 294664.28 | 59.71% | -1.16(-1.46 - -0.86) |
| United Republic of Tanzania | GABD | 7444.22 | 20022.87 | 168.97% | 0.69(0.66-0.72) | 343.96 | 829.62 | 141.20% | 0.47(0.25-0.69) | 11236.55 | 23949.32 | 113.14% | 0.28(0.1-0.46) |
| United Republic of Tanzania | IFAH | 16727.36 | 48951.9 | 192.65% | 1.33(1.23-1.44) | 259.18 | 404.8 | 56.18% | -0.1(-0.31-0.11) | 20123.39 | 29818.32 | 48.18% | -0.08(-0.21-0.06) |
| United Republic of Tanzania | IFBD | 155.72 | 421.21 | 170.49% | 0.56(0.41-0.71) | 85.85 | 181.36 | 111.25% | 0.38(0.19-0.57) | 4655.41 | 8564.7 | 83.97% | 0.25(0.07-0.43) |
| United Republic of Tanzania | PACA | 3245.63 | 7699.03 | 137.21% | 0.02(0-0.04) | 159.52 | 382.53 | 139.80% | 0.24(0.1-0.38) | 5903.69 | 14159.52 | 139.84% | 0.26(0.13-0.39) |
| United Republic of Tanzania | PIIO | 22376.31 | 61067.87 | 172.91% | 0.8(0.73-0.87) | 888.11 | 1880.4 | 111.73% | 0.57(0.35-0.78) | 54153.11 | 99437.98 | 83.62% | 0.51(0.27-0.74) |
| United Republic of Tanzania | UDSD | 811524.86 | 1946145.08 | 139.81% | 0.047(0.04-0.054) | 506.89 | 827.92 | 63.33% | -0.96(-1.22 - -0.69) | 38614.21 | 76578.48 | 98.32% | -0.43(-0.55 - -0.31) |
| United Republic of Tanzania | VAID | 1108.08 | 3535.25 | 219.04% | 1.44(1.34-1.54) | 110.47 | 323.13 | 192.50% | 0.9(0.73-1.07) | 3013.23 | 7880.23 | 161.52% | 0.89(0.75-1.03) |
| United States of America | Digestive diseases | 18712057.95 | 23025035.36 | 23.05% | -0.58(-0.7 - -0.45) | 78301.04 | 129343.13 | 65.19% | -0.1(-0.14 - -0.05) | 2579264.67 | 3667587.07 | 42.20% | -0.21(-0.29 - -0.14) |
| United States of America | APED | 457420.35 | 508783.47 | 11.23% | -0.46(-0.57 - -0.34) | 465.99 | 650.8 | 39.66% | -0.65(-0.9 - -0.4) | 17612.77 | 20638.74 | 17.18% | -0.73(-0.86 - -0.6) |
| United States of America | COCLD | 65860.15 | 90854.41 | 37.95% | 0.19(0.09-0.29) | 37385.8 | 67286.45 | 79.98% | 0.39(0.31-0.46) | 1096610.98 | 1825799.85 | 66.49% | 0.27(0.19-0.35) |
| United States of America | GABD | 3248935.19 | 3646530.96 | 12.24% | 0.49(-0.03-1.01) | 4287.11 | 6834.17 | 59.41% | -0.29(-0.37 - -0.21) | 247968.51 | 306034.66 | 23.42% | 0.25(-0.08-0.58) |
| United States of America | IFAH | 341142.83 | 403967.86 | 18.42% | -0.79(-1.01 - -0.57) | 1367.99 | 2029.32 | 48.34% | -0.53(-0.63 - -0.43) | 68340.71 | 80272.8 | 17.46% | -0.82(-0.91 - -0.74) |
| United States of America | IFBD | 96513.12 | 85387.95 | -11.53% | -1(-1.44 - -0.55) | 2171.48 | 5910.12 | 172.17% | 1.8(1.52-2.07) | 186357.1 | 215289.16 | 15.53% | -0.48(-0.78 - -0.18) |
| United States of America | PACA | 183031.85 | 228699.22 | 24.95% | -0.72(-0.91 - -0.53) | 3049.17 | 4896.91 | 60.60% | -0.29(-0.51 - -0.07) | 92110.21 | 133259.77 | 44.67% | -0.26(-0.43 - -0.09) |
| United States of America | PIIO | 735825.47 | 1083333.99 | 47.23% | -0.28(-0.33 - -0.23) | 6038.31 | 12043.17 | 99.45% | 0.47(0.3-0.63) | 121216.96 | 201466.31 | 66.20% | 0.56(0.31-0.81) |
| United States of America | UDSD | 13381852.82 | 16707286.04 | 24.85% | -0.79(-0.959 - -0.621) | 9361.02 | 5396.47 | -42.35% | -4.47(-4.91 - -4.02) | 494107.53 | 478871.36 | -3.08% | -1.77(-2.01 - -1.53) |
| United States of America | VAID | 201476.17 | 270191.45 | 34.11% | -0.7(-0.75 - -0.65) | 7805.71 | 11085.44 | 42.02% | -1.1(-1.34 - -0.86) | 138291.72 | 190066.94 | 37.44% | -1.05(-1.24 - -0.86) |
| United States Virgin Islands | Digestive diseases | 7589.02 | 9361.49 | 23.36% | 0.1(0.09-0.11) | 32.74 | 57.04 | 74.23% | -0.32(-0.51 - -0.12) | 1265.03 | 1673.6 | 32.30% | -0.49(-0.61 - -0.36) |
| United States Virgin Islands | APED | 279.59 | 286.25 | 2.38% | 0.82(0.7-0.95) | 0.71 | 0.84 | 18.31% | -1.03(-1.24 - -0.82) | 33.63 | 26.66 | -20.73% | -1.62(-1.77 - -1.46) |
| United States Virgin Islands | COCLD | 25.09 | 27.5 | 9.61% | 0.29(0.24-0.34) | 20.26 | 33.15 | 63.62% | -0.44(-0.69 - -0.19) | 641.46 | 854.93 | 33.28% | -0.62(-0.84 - -0.4) |
| United States Virgin Islands | GABD | 372.95 | 483.73 | 29.70% | 0.13(0.09-0.17) | 2.19 | 3.69 | 68.49% | -0.87(-1.12 - -0.61) | 96.3 | 121.09 | 25.74% | -0.84(-1 - -0.69) |
| United States Virgin Islands | IFAH | 210.88 | 261.72 | 24.11% | 0.5(0.34-0.66) | 0.5 | 0.76 | 52.00% | -0.88(-1.23 - -0.53) | 67.32 | 72.46 | 7.64% | -0.28(-0.37 - -0.19) |
| United States Virgin Islands | IFBD | 2.35 | 3.62 | 54.04% | 0.73(0.57-0.89) | 0.65 | 0.73 | 12.31% | -2.32(-2.66 - -1.99) | 25.68 | 23.29 | -9.31% | -2.03(-2.27 - -1.78) |
| United States Virgin Islands | PACA | 27.82 | 36.55 | 31.38% | -0.02(-0.04-0) | 1.03 | 1.77 | 71.84% | 0.04(-0.13-0.21) | 37.05 | 48.54 | 31.01% | -0.32(-0.38 - -0.26) |
| United States Virgin Islands | PIIO | 104.94 | 169.51 | 61.54% | 0.76(0.62-0.9) | 2.12 | 5.39 | 154.30% | 1.03(0.72-1.34) | 69.42 | 109.33 | 57.49% | 0.28(0.03-0.53) |
| United States Virgin Islands | UDSD | 6555.07 | 8071.13 | 23.13% | 0.034(0.032-0.037) | 2.68 | 3.49 | 30.22% | -1.85(-2.11 - -1.58) | 222.18 | 268.9 | 21.03% | -0.51(-0.6 - -0.43) |
| United States Virgin Islands | VAID | 10.33 | 21.49 | 108.03% | 0.75(0.68-0.83) | 0.9 | 1.89 | 110.00% | -0.35(-0.59 - -0.11) | 20.4 | 35.93 | 76.13% | -0.53(-0.76 - -0.3) |
| Uruguay | Digestive diseases | 197872.51 | 242680.32 | 22.64% | 0.08(0.07-0.08) | 1319.76 | 1546.07 | 17.15% | -1.04(-1.1 - -0.99) | 35646.05 | 35337.96 | -0.86% | -1.11(-1.17 - -1.05) |
| Uruguay | APED | 6399.43 | 9657.69 | 50.91% | 1.25(1.19-1.31) | 20 | 15.97 | -20.15% | -1.65(-1.96 - -1.34) | 608.18 | 461.43 | -24.13% | -1.25(-1.53 - -0.97) |
| Uruguay | COCLD | 580.27 | 637.33 | 9.83% | -0.58(-0.69 - -0.46) | 532.98 | 470.98 | -11.63% | -1.91(-2.04 - -1.79) | 14149.54 | 11026.46 | -22.07% | -2.11(-2.24 - -1.97) |
| Uruguay | GABD | 5803.2 | 7729.38 | 33.19% | 0.24(0.2-0.28) | 113.9 | 153.84 | 35.07% | -0.02(-0.34-0.31) | 2654.26 | 3008.55 | 13.35% | -0.27(-0.53 - -0.01) |
| Uruguay | IFAH | 4525.32 | 6290.39 | 39.00% | 0.53(0.47-0.6) | 43.47 | 42.13 | -3.08% | -1.54(-1.97 - -1.11) | 2109.98 | 1898.57 | -10.02% | -1.02(-1.25 - -0.8) |
| Uruguay | IFBD | 41.25 | 65.53 | 58.86% | 0.85(0.76-0.94) | 10.06 | 22.44 | 123.06% | 1.1(0.62-1.58) | 338.84 | 572.06 | 68.83% | 0.63(0.27-0.98) |
| Uruguay | PACA | 1088.9 | 1280.78 | 17.62% | -0.27(-0.3 - -0.24) | 61.6 | 65.8 | 6.82% | -1.19(-1.44 - -0.94) | 1775.87 | 1718.64 | -3.22% | -1.28(-1.48 - -1.07) |
| Uruguay | PIIO | 3363.88 | 5313.75 | 57.96% | 0.71(0.63-0.78) | 166.71 | 281.52 | 68.87% | 0.16(0.06-0.25) | 3102.32 | 4149.98 | 33.77% | -0.07(-0.17-0.04) |
| Uruguay | UDSD | 175020.69 | 210112.79 | 20.05% | -0.007(-0.009 - -0.005) | 144.56 | 90.92 | -37.11% | -3.58(-3.76 - -3.39) | 6868.55 | 6364.7 | -7.34% | -1.06(-1.14 - -0.97) |
| Uruguay | VAID | 1049.56 | 1592.69 | 51.75% | 0.3(0.25-0.36) | 196.08 | 300.51 | 53.26% | -0.23(-0.35 - -0.11) | 3344.5 | 4381.17 | 31.00% | -0.38(-0.47 - -0.29) |
| Uzbekistan | Digestive diseases | 848205.99 | 1703745.03 | 100.86% | 0.11(0.09-0.12) | 5416 | 15018.63 | 177.30% | 0.82(0.2-1.45) | 236301.25 | 624149.53 | 164.13% | 0.48(-0.07-1.03) |
| Uzbekistan | APED | 52357.89 | 86047.79 | 64.35% | 0.16(0.06-0.27) | 64.06 | 40.74 | -36.40% | -3.49(-3.84 - -3.14) | 4020.72 | 2907 | -27.70% | -3.26(-3.65 - -2.88) |
| Uzbekistan | COCLD | 6750.22 | 22488.64 | 233.15% | 1.58(1.26-1.91) | 4146.9 | 13042.32 | 214.51% | 1.01(0.33-1.69) | 150132.11 | 488946.67 | 225.68% | 0.84(0.18-1.5) |
| Uzbekistan | GABD | 57839.96 | 128974.5 | 122.99% | 0.4(0.36-0.43) | 118.18 | 179.16 | 51.60% | 0.83(0.5-1.16) | 10202.03 | 18220.38 | 78.60% | -0.19(-0.29 - -0.09) |
| Uzbekistan | IFAH | 22510.32 | 49314.75 | 119.08% | 1.31(1.04-1.58) | 52.6 | 53.5 | 1.71% | -0.12(-0.36-0.12) | 8625.09 | 12470.36 | 44.58% | 0.1(0-0.19) |
| Uzbekistan | IFBD | 868.11 | 2213 | 154.92% | 1.04(0.95-1.13) | 47.93 | 72.55 | 51.37% | -0.33(-0.58 - -0.09) | 4299.2 | 6868.98 | 59.77% | -0.47(-0.58 - -0.36) |
| Uzbekistan | PACA | 4311.9 | 8818.38 | 104.51% | 0.06(0.04-0.07) | 127.55 | 345.09 | 170.55% | 1.11(0.93-1.29) | 5121.24 | 13879.16 | 171.01% | 0.73(0.57-0.88) |
| Uzbekistan | PIIO | 12090.04 | 22374.35 | 85.06% | 0.32(0.26-0.37) | 272.09 | 223.06 | -18.02% | -0.31(-0.4 - -0.22) | 17193.18 | 11801.15 | -31.36% | -1.53(-1.66 - -1.4) |
| Uzbekistan | UDSD | 689462.43 | 1379437.81 | 100.07% | 0.017(0.015-0.02) | 454.56 | 814.7 | 79.23% | -0.59(-1.36-0.18) | 31161.57 | 59451.76 | 90.79% | -0.75(-1.23 - -0.26) |
| Uzbekistan | VAID | 2015.11 | 4075.81 | 102.26% | 1.18(1.01-1.35) | 47.03 | 82.51 | 75.44% | 1.78(1.62-1.94) | 1151.61 | 2320.98 | 101.54% | 1.27(1.17-1.37) |
| Vanuatu | Digestive diseases | 3506.44 | 7709.93 | 119.88% | -0.05(-0.07 - -0.03) | 48.76 | 92.01 | 88.71% | -1.35(-1.49 - -1.21) | 1894.87 | 3475.71 | 83.43% | -1.24(-1.4 - -1.09) |
| Vanuatu | APED | 176.09 | 363.53 | 106.45% | -0.15(-0.27 - -0.02) | 0.73 | 1.32 | 80.82% | -1.32(-1.41 - -1.23) | 33.19 | 57.9 | 74.45% | -1.18(-1.3 - -1.07) |
| Vanuatu | COCLD | 33.42 | 61.78 | 84.86% | -0.56(-0.63 - -0.49) | 26.46 | 49.43 | 86.81% | 1.69(-1.5 - -1.14) | 1017.08 | 1839.34 | 80.85% | -1.3(-1.48 - -1.11) |
| Vanuatu | GABD | 182.3 | 426.28 | 133.83% | 0.13(0.09-0.18) | 2.61 | 6.63 | 154.02% | -0.38(-0.49 - -0.27) | 105.8 | 250.03 | 136.32% | -0.34(-0.44 - -0.23) |
| Vanuatu | IFAH | 113.28 | 278.16 | 145.55% | 0.08(-0.04-0.21) | 0.82 | 1.63 | 98.78% | -1.16(-1.23 - -1.09) | 53.78 | 113.95 | 111.88% | -0.56(-0.63 - -0.48) |
| Vanuatu | IFBD | 0.51 | 1.46 | 186.27% | 0.99(0.88-1.1) | 0.85 | 1.52 | 78.82% | -1.74(-1.94 - -1.54) | 33.38 | 57.27 | 71.57% | -1.58(-1.8 - -1.36) |
| Vanuatu | PACA | 28.57 | 64.87 | 127.06% | -0.02(-0.03-0) | 1.47 | 3.44 | 134.01% | -0.62(-0.77 - -0.48) | 58.12 | 133.83 | 130.26% | -0.53(-0.69 - -0.36) |
| Vanuatu | PIIO | 62.02 | 122.27 | 97.16% | -0.07(-0.17-0.03) | 2.01 | 4.57 | 127.01% | -0.51(-0.59 - -0.44) | 71.45 | 141.87 | 98.56% | -0.41(-0.5 - -0.31) |
| Vanuatu | UDSD | 2905.48 | 6377.97 | 119.52% | -0.059(-0.068 - -0.049) | 12.3 | 19.39 | 57.64% | -2.09(-2.26 - -1.93) | 471.05 | 750.63 | 59.35% | -1.85(-2.01 - -1.69) |
| Vanuatu | VAID | 4.78 | 13.6 | 184.52% | 1.06(1.03-1.1) | 0.31 | 1.11 | 258.06% | 0.92(0.79-1.06) | 7.87 | 28.65 | 264.04% | 1.16(1-1.33) |
| Venezuela (Bolivarian Republic of) | Digestive diseases | 1236353.58 | 2355932.76 | 90.55% | 0.01(0-0.01) | 4058.39 | 8600.63 | 111.92% | -1.09(-1.27 - -0.91) | 189998.44 | 314017.67 | 65.27% | -1.04(-1.2 - -0.88) |
| Venezuela (Bolivarian Republic of) | APED | 87135.45 | 114066.21 | 30.91% | -0.08(-0.19-0.03) | 220.18 | 153.94 | -30.08% | -2.68(-3.4 - -1.94) | 13426.34 | 7155.54 | -46.71% | -2.56(-3.29 - -1.83) |
| Venezuela (Bolivarian Republic of) | COCLD | 3887.7 | 6248.87 | 60.73% | -0.85(-0.92 - -0.79) | 2124.12 | 4691.73 | 120.88% | -1.1(-1.32 - -0.88) | 70880.13 | 135947.76 | 91.80% | -1.16(-1.38 - -0.94) |
| Venezuela (Bolivarian Republic of) | GABD | 123313.69 | 265053.5 | 114.94% | 0.22(0.2-0.25) | 219.43 | 412.77 | 88.11% | -1.65(-2.04 - -1.25) | 20473.26 | 35400.64 | 72.91% | -1.01(-1.2 - -0.81) |
| Venezuela (Bolivarian Republic of) | IFAH | 36043.6 | 65179.41 | 80.83% | 0.21(0.12-0.3) | 120.73 | 198.71 | 64.59% | -1.28(-1.47 - -1.09) | 14605.36 | 19546.54 | 33.83% | -0.82(-0.94 - -0.7) |
| Venezuela (Bolivarian Republic of) | IFBD | 351.55 | 654.83 | 86.27% | -0.64(-0.84 - -0.44) | 27.82 | 44.02 | 58.23% | -2.32(-2.82 - -1.82) | 1676.4 | 2642.1 | 57.61% | -1.36(-1.66 - -1.05) |
| Venezuela (Bolivarian Republic of) | PACA | 5490.96 | 10494.35 | 91.12% | -0.15(-0.17 - -0.13) | 140.93 | 340.22 | 141.41% | -0.41(-0.55 - -0.27) | 5437.96 | 10909.93 | 100.63% | -0.46(-0.62 - -0.31) |
| Venezuela (Bolivarian Republic of) | PIIO | 23079.48 | 39294.78 | 70.26% | 0.24(0.16-0.31) | 397.25 | 806.33 | 102.98% | -0.09(-0.32-0.13) | 20531.43 | 19898.6 | -3.08% | -0.95(-1.31 - -0.59) |
| Venezuela (Bolivarian Republic of) | UDSD | 955398.62 | 1850062.28 | 93.64% | -0.026(-0.027 - -0.025) | 548.09 | 873.02 | 59.28% | -2.99(-3.28 - -2.7) | 35207.8 | 59235.26 | 68.24% | -1.27(-1.38 - -1.17) |
| Venezuela (Bolivarian Republic of) | VAID | 1652.53 | 4878.52 | 195.22% | 0.79(0.68-0.91) | 192.81 | 673.13 | 249.12% | 0.47(0.3-0.65) | 4556 | 13002.97 | 185.40% | 0.33(0.16-0.49) |
| Viet Nam | Digestive diseases | 1754709.72 | 3559392.27 | 102.85% | 0.26(0.25-0.27) | 23827.13 | 31306.12 | 31.39% | -2.32(-2.67 - -1.97) | 772187.27 | 1012759.17 | 31.15% | -2.11(-2.45 - -1.76) |
| Viet Nam | APED | 144397.65 | 265708.23 | 84.01% | 1.25(1.07-1.07) | 340.55 | 189.89 | -44.24% | -4.39(-4.64 - -4.14) | 16906.18 | 9878.63 | -41.57% | -3.4(-3.62 - -3.17) |
| Viet Nam | COCLD | 13537.6 | 32979.7 | 143.62% | 0.28(0.01-0.54) | 16676.24 | 23636.91 | 41.74% | -2.15(-2.55 - -1.74) | 508765.08 | 712199.22 | 39.99% | -2.17(-2.58 - -1.75) |
| Viet Nam | GABD | 113865.81 | 279417.22 | 145.39% | 0.58(0.5-0.66) | 1398.54 | 1663.4 | 18.94% | -2.44(-2.65 - -2.23) | 44512.24 | 58751.63 | 31.99% | -2.01(-2.19 - -1.83) |
| Viet Nam | IFAH | 32424.55 | 103956.95 | 220.61% | 1.87(1.74-2.01) | 213.79 | 208.28 | -2.58% | -2.96(-3.29 - -2.64) | 14921.57 | 23184.52 | 55.38% | -0.59(-0.8 - -0.37) |
| Viet Nam | IFBD | 401.81 | 1481.73 | 268.76% | 2.62(2.11-3.13) | 220.67 | 177.48 | -19.57% | -3.98(-4.35 - -3.61) | 6650.49 | 7419.38 | 11.56% | -2.48(-2.72 - -2.23) |
| Viet Nam | PACA | 15445.32 | 31786.37 | 105.80% | 0.05(-0.05-0.14) | 797.66 | 1056.1 | 32.40% | -2.08(-2.35 - -1.81) | 25769.9 | 33331.82 | 29.34% | -2.02(-2.29 - -1.74) |
| Viet Nam | PIIO | 65604.64 | 175287.04 | 167.19% | 1.6(1.55-1.64) | 1904.82 | 1995.33 | 4.75% | -2.49(-2.73 - -2.24) | 72465.62 | 55999.87 | -22.72% | -2.48(-2.73 - -2.22) |
| Viet Nam | UDSD | 1366505.69 | 2661660.23 | 94.78% | 0.003(-0.006-0.011) | 992.09 | 772.27 | -22.16% | -3.89(-4.14 - -3.65) | 53117.89 | 79364.3 | 49.41% | -1.3(-1.42 - -1.18) |
| Viet Nam | VAID | 2526.66 | 7114.81 | 181.59% | 1.29(1.25-1.34) | 374.01 | 612.99 | 63.90% | -1.45(-1.65 - -1.24) | 6407.19 | 10194.55 | 59.11% | -1.48(-1.69 - -1.26) |
| Yemen | Digestive diseases | 493907.14 | 1405149.24 | 184.50% | 0.08(0.08-0.09) | 2728.58 | 4876.84 | 78.73% | -1.37(-1.46 - -1.27) | 120959.02 | 204366.5 | 68.96% | -1.29(-1.37 - -1.21) |
| Yemen | APED | 29146.92 | 90188.73 | 209.43% | 0.9(0.87-0.94) | 99.74 | 141 | 41.37% | -2.03(-2.14 - -1.91) | 5494.18 | 7000.28 | 27.41% | -2.02(-2.12 - -1.91) |
| Yemen | COCLD | 1736.96 | 4660.28 | 168.30% | 0.35(0.18-0.52) | 1546.16 | 2785.77 | 80.17% | -1.45(-1.6 - -1.31) | 56872.86 | 93431.5 | 64.28% | -1.51(-1.65 - -1.38) |
| Yemen | GABD | 14848.02 | 48677.72 | 227.84% | 0.73(0.7-0.76) | 79.15 | 233.27 | 194.72% | 0.32(0.22-0.42) | 4414.79 | 11376.13 | 157.68% | -0.07(-0.12 - -0.01) |
| Yemen | IFAH | 7316.16 | 17459.48 | 138.64% | 1.04(0.92-1.16) | 22.13 | 40.88 | 84.73% | -0.31(-0.4 - -0.22) | 3432.78 | 6942.47 | 102.24% | 0.06(-0.02-0.15) |
| Yemen | IFBD | 192.4 | 646.77 | 236.16% | 0.73(0.69-0.77) | 13.97 | 44.35 | 217.47% | 0.85(0.73-0.97) | 772.46 | 2423.13 | 213.69% | 0.79(0.66-0.93) |
| Yemen | PACA | 2020.74 | 5560.45 | 175.17% | 0.06(0.05-0.07) | 54.31 | 136.62 | 151.56% | -0.45(-0.51 - -0.38) | 1670.94 | 4204.33 | 151.61% | -0.44(-0.49 - -0.39) |
| Yemen | PIIO | 5569.23 | 13562.64 | 143.53% | 0.8(0.68-0.91) | 246.92 | 399.54 | 61.81% | -0.09(-0.16 - -0.02) | 16319.25 | 18543.69 | 13.63% | -0.58(-0.67 - -0.49) |
| Yemen | UDSD | 432586.92 | 1222816.75 | 182.68% | -0.001(-0.004-0.001) | 601.41 | 886.43 | 47.39% | -2.37(-2.5 - -2.24) | 29689.27 | 53763.06 | 81.09% | -1.68(-1.77 - -1.59) |
| Yemen | VAID | 489.79 | 1576.41 | 221.85% | 1.2(1.13-1.27) | 27.96 | 73.91 | 164.34% | -0.18(-0.23 - -0.12) | 639.86 | 1661.7 | 159.70% | -0.15(-0.2 - -0.11) |
| Zambia | Digestive diseases | 269297.37 | 704220.89 | 161.50% | 0.1(0.09-0.12) | 3584.47 | 6528.26 | 82.13% | -1.14(-1.39 - -0.89) | 149016.85 | 267667.87 | 79.62% | -1.07(-1.31 - -0.84) |
| Zambia | APED | 11322.15 | 35588.22 | 214.32% | 1.18(1.01-1.36) | 86.16 | 84.37 | -2.08% | -2.4(-2.77 - -2.02) | 5651.17 | 4974.62 | -11.97% | -2.59(-2.88 - -2.29) |
| Zambia | COCLD | 1839.09 | 4513.03 | 145.39% | -0.02(-0.09-0.04) | 2373.12 | 4592.97 | 93.54% | -1.07(-1.32 - -0.82) | 84008.48 | 166807.09 | 98.56% | -1.01(-1.26 - -0.76) |
| Zambia | GABD | 2240.15 | 6191.75 | 176.40% | 0.6(0.56-0.64) | 120.66 | 237.55 | 96.88% | -0.58(-0.82 - -0.35) | 4111.94 | 7357.13 | 78.92% | -0.83(-1.07 - -0.6) |
| Zambia | IFAH | 5319.63 | 14308.73 | 168.98% | 0.74(0.61-0.87) | 87.91 | 102.69 | 16.81% | -1.38(-1.64 - -1.13) | 6761.39 | 8021.01 | 18.63% | -1.23(-1.36 - -1.1) |
| Zambia | IFBD | 53.17 | 143.01 | 168.97% | 0.32(0.22-0.43) | 35.13 | 55.19 | 57.10% | -0.86(-1.04 - -0.69) | 1941 | 2547.96 | 31.27% | -1.05(-1.14 - -0.96) |
| Zambia | PACA | 991.86 | 2359.28 | 137.86% | -0.23(-0.25 - -0.21) | 57.98 | 118.41 | 104.23% | -1.07(-1.34 - -0.79) | 2189.83 | 4678.89 | 113.66% | -0.92(-1.2 - -0.64) |
| Zambia | PIIO | 5723.58 | 18830.87 | 229.01% | 1.34(1.17-1.51) | 351.65 | 646.27 | 83.78% | -0.77(-0.94 - -0.6) | 19395.69 | 32664.5 | 68.41% | -0.63(-0.76 - -0.5) |
| Zambia | UDSD | 241447.77 | 621242.43 | 157.30% | 0.024(0.019-0.03) | 283.4 | 356.58 | 25.82% | -2.64(-3.05 - -2.22) | 17194.16 | 29187.67 | 69.75% | -1.63(-1.9 - -1.36) |
| Zambia | VAID | 359.97 | 1043.57 | 189.90% | 0.87(0.72-1.03) | 39.26 | 86.91 | 121.37% | -0.28(-0.44 - -0.12) | 1121.59 | 2243.17 | 100.00% | -0.35(-0.51 - -0.19) |
| Zimbabwe | Digestive diseases | 395177.39 | 663033.86 | 67.78% | -0.02(-0.08-0.05) | 2625.95 | 4437.16 | 68.97% | 0.13(-0.04-0.3) | 105116.16 | 180829.87 | 72.03% | 0.27(0.12-0.42) |
| Zimbabwe | APED | 27692.14 | 41992.16 | 51.64% | -0.18(-0.53-0.18) | 37.42 | 64.08 | 71.25% | 1.06(0.78-1.34) | 2417.96 | 3856.22 | 59.48% | 1.06(0.74-1.39) |
| Zimbabwe | COCLD | 1753.26 | 2657.44 | 51.57% | -0.37(-0.74-0) | 1449.65 | 2065.06 | 42.45% | -0.54(-0.78 - -0.3) | 47952.2 | 71581.36 | 49.28% | -0.33(-0.58 - -0.07) |
| Zimbabwe | GABD | 6259.07 | 10198.68 | 62.94% | 0(-0.18-0.18) | 92.91 | 206.84 | 122.62% | 1.32(1.1-1.53) | 3502.13 | 7603.27 | 117.10% | 1.37(1.17-1.58) |
| Zimbabwe | IFAH | 20653.62 | 30620.05 | 48.26% | -0.5(-0.89 - -0.11) | 71.85 | 136.81 | 90.41% | 0.6(0.44-0.75) | 8524.22 | 13693.04 | 60.64% | 0.29(0.12-0.46) |
| Zimbabwe | IFBD | 81.42 | 145.55 | 78.76% | -0.14(-0.27 - -0.01) | 21.93 | 43.45 | 98.13% | 0.96(0.85-1.06) | 1018.39 | 1887.98 | 85.39% | 1.22(0.99-1.44) |
| Zimbabwe | PACA | 1251.39 | 2145.05 | 71.41% | 0.11(0.09-0.13) | 46.35 | 110.56 | 138.53% | 0.87(0.72-1.01) | 1647.89 | 4173.38 | 153.26% | 1.07(0.9-1.24) |
| Zimbabwe | PIIO | 12306.96 | 14962.36 | 21.58% | -1.14(-1.45 - -0.83) | 459.31 | 864.34 | 88.18% | 0.41(0.33-0.5) | 17827.68 | 32818.52 | 84.09% | 0.58(0.5-0.66) |
| Zimbabwe | UDSD | 324666.32 | 559525.16 | 72.34% | 0.047(0.022-0.073) | 351.47 | 710.97 | 102.28% | 0.88(0.66-1.09) | 19121.77 | 37801.65 | 97.69% | 0.81(0.66-0.96) |
| Zimbabwe | VAID | 513.22 | 787.41 | 53.43% | -0.21(-0.51-0.1) | 19.08 | 42.05 | 120.39% | 0.92(0.85-0.98) | 412.73 | 927.54 | 124.73% | 1.02(0.95-1.09) |
| COCLD: Cirrhosis and other chronic liver diseases; UDSD: Upper digestive system diseases; APED: Appendicitis; PIIO: Paralytic ileus and intestinal obstruction; IFAH: Inguinal, femoral, and abdominal hernia; IFBD: Inflamma-ry bowel disease; VAID: Vascular intestinal disorders; GABD: Gallbladder and biliary diseases; PACA:Pancreatitis. | | | | | | | | | | | | | |
