# Supplementary figures and images for: Global, regional, and national burden of digestive diseases: findings from the global burden of disease study 2019
Source: Front Public Health. 2023 Aug 24;11:1202980. doi: 10.3389/fpubh.2023.1202980 (PMC10483149; doi:10.3389/fpubh.2023.1202980)

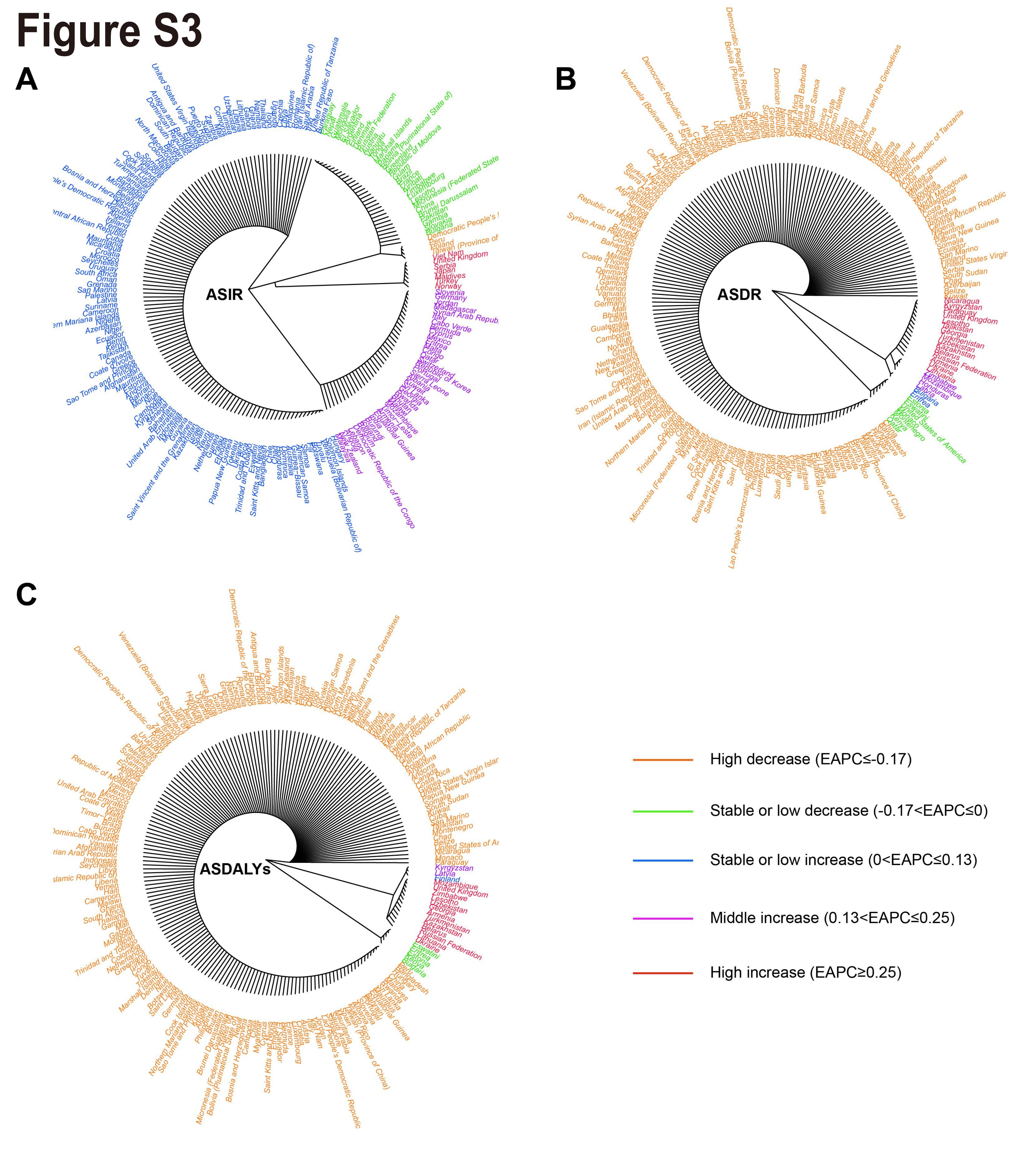

Supplement: Supplementary file 13 [file Image_3.JPEG]

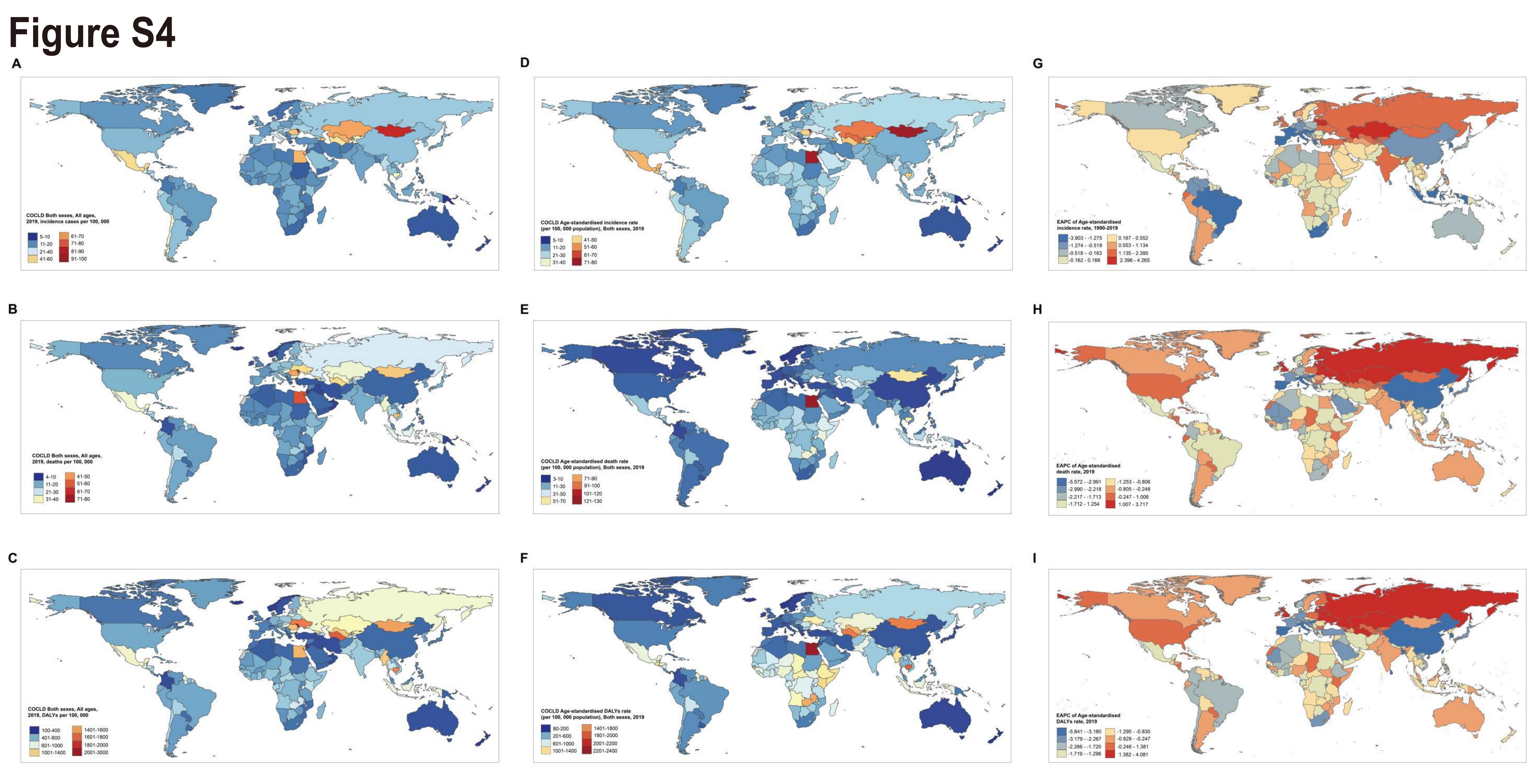

Supplement: Supplementary file 14 [file Image_4.JPEG]

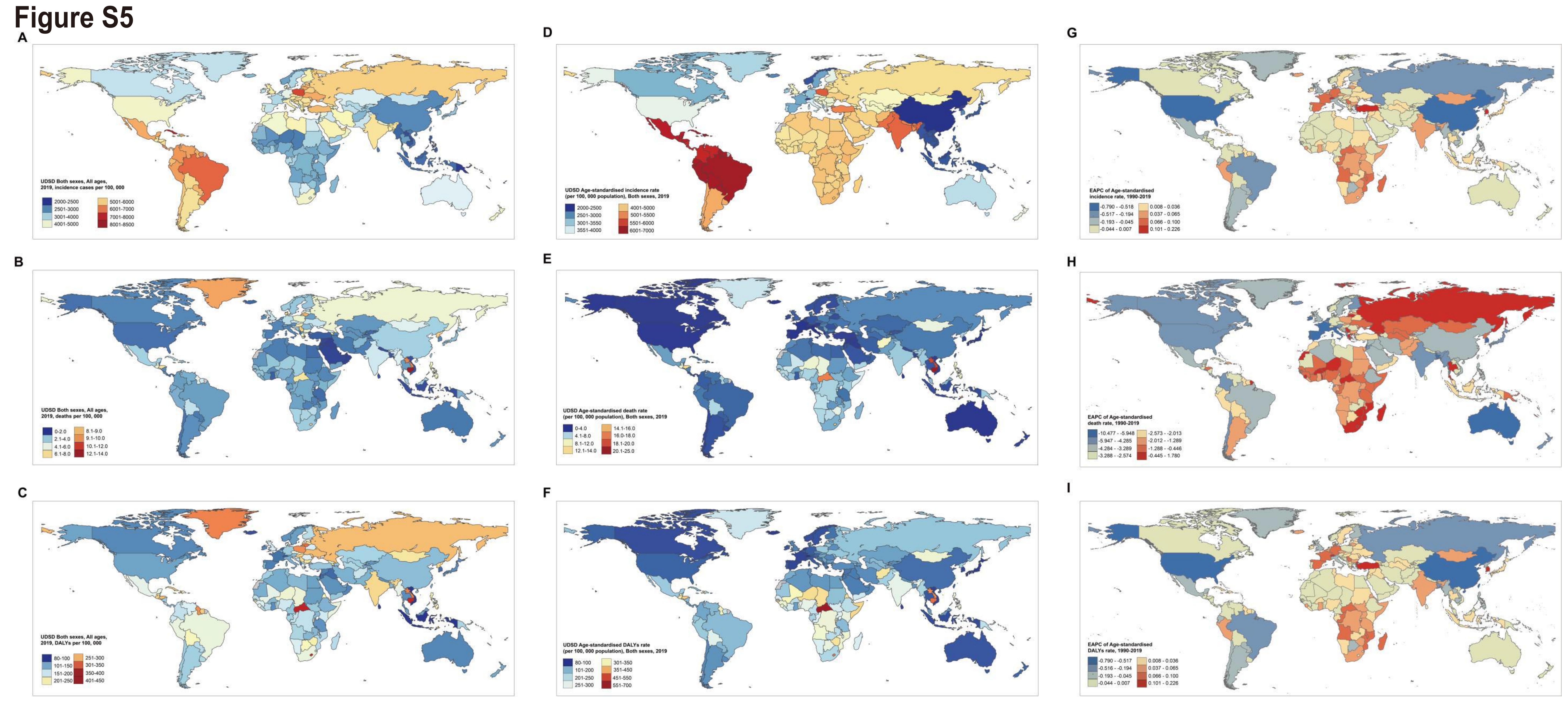

Supplement: Supplementary file 15 [file Image_5.JPEG]

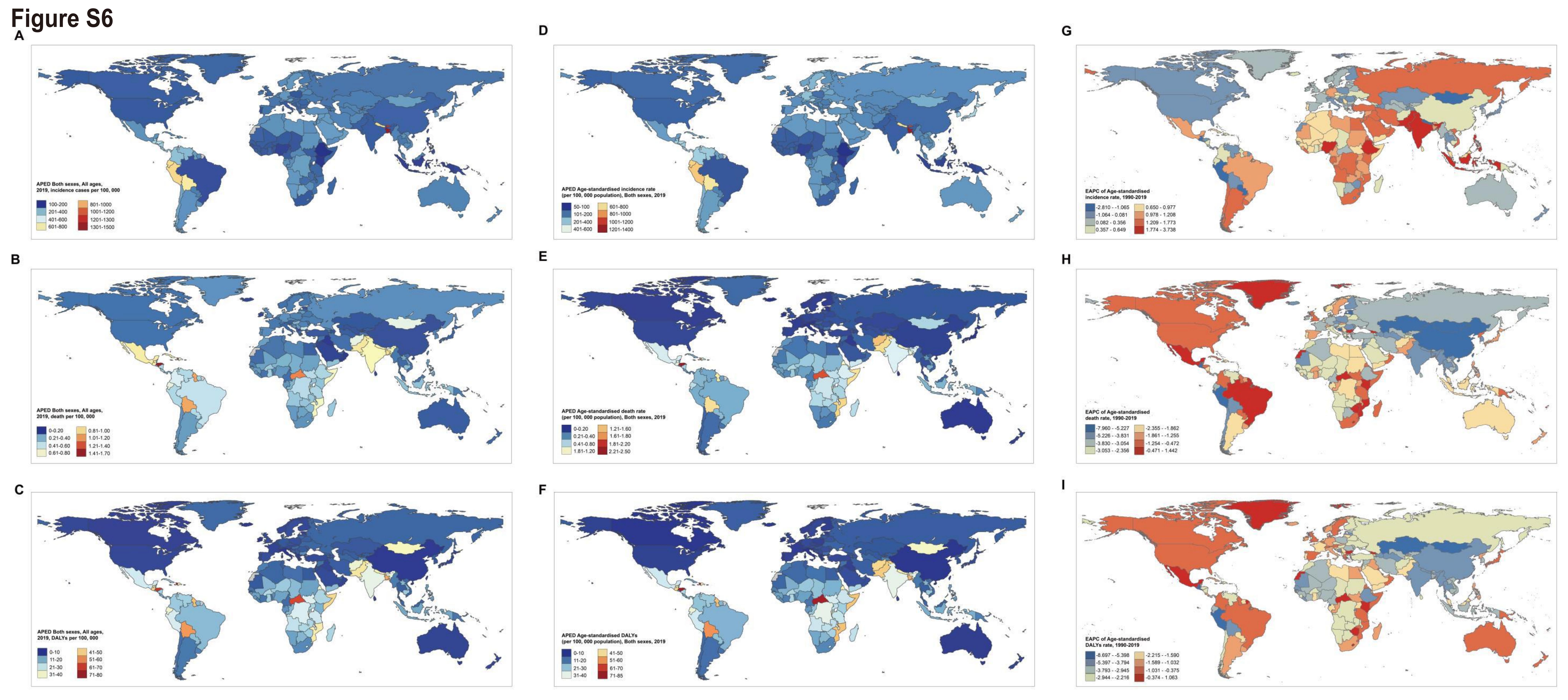

Supplement: Supplementary file 16 [file Image_6.JPEG]

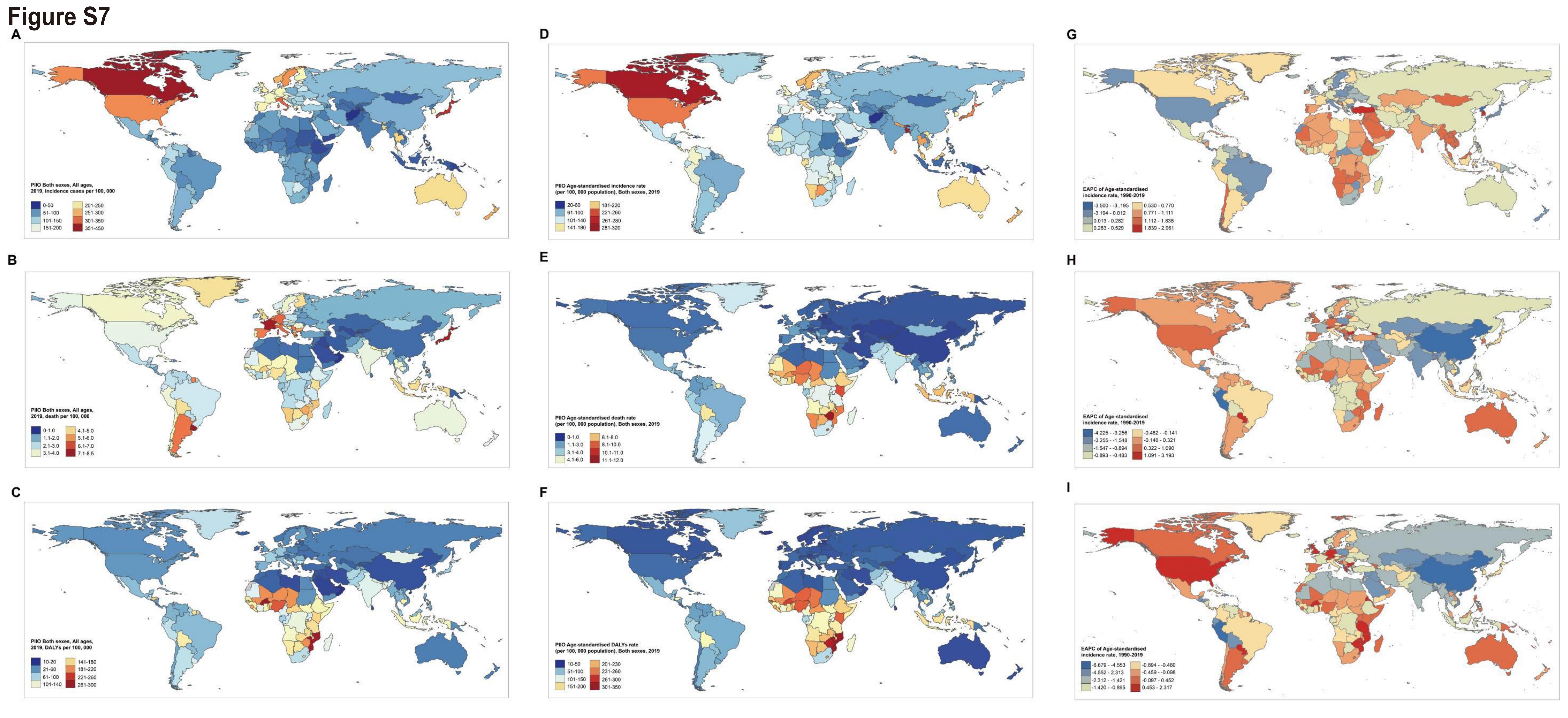

Supplement: Supplementary file 17 [file Image_7.JPEG]

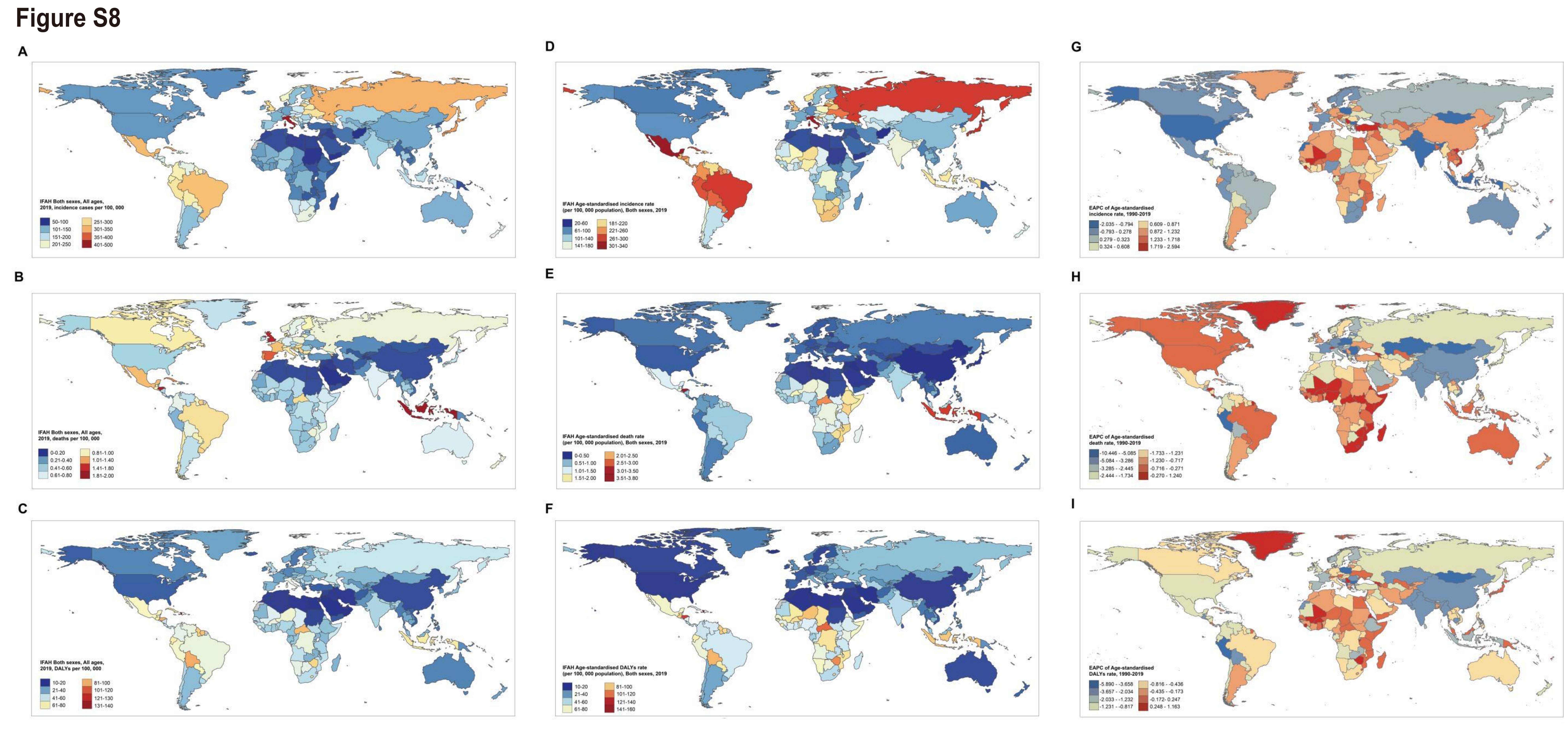

Supplement: Supplementary file 18 [file Image_8.JPEG]

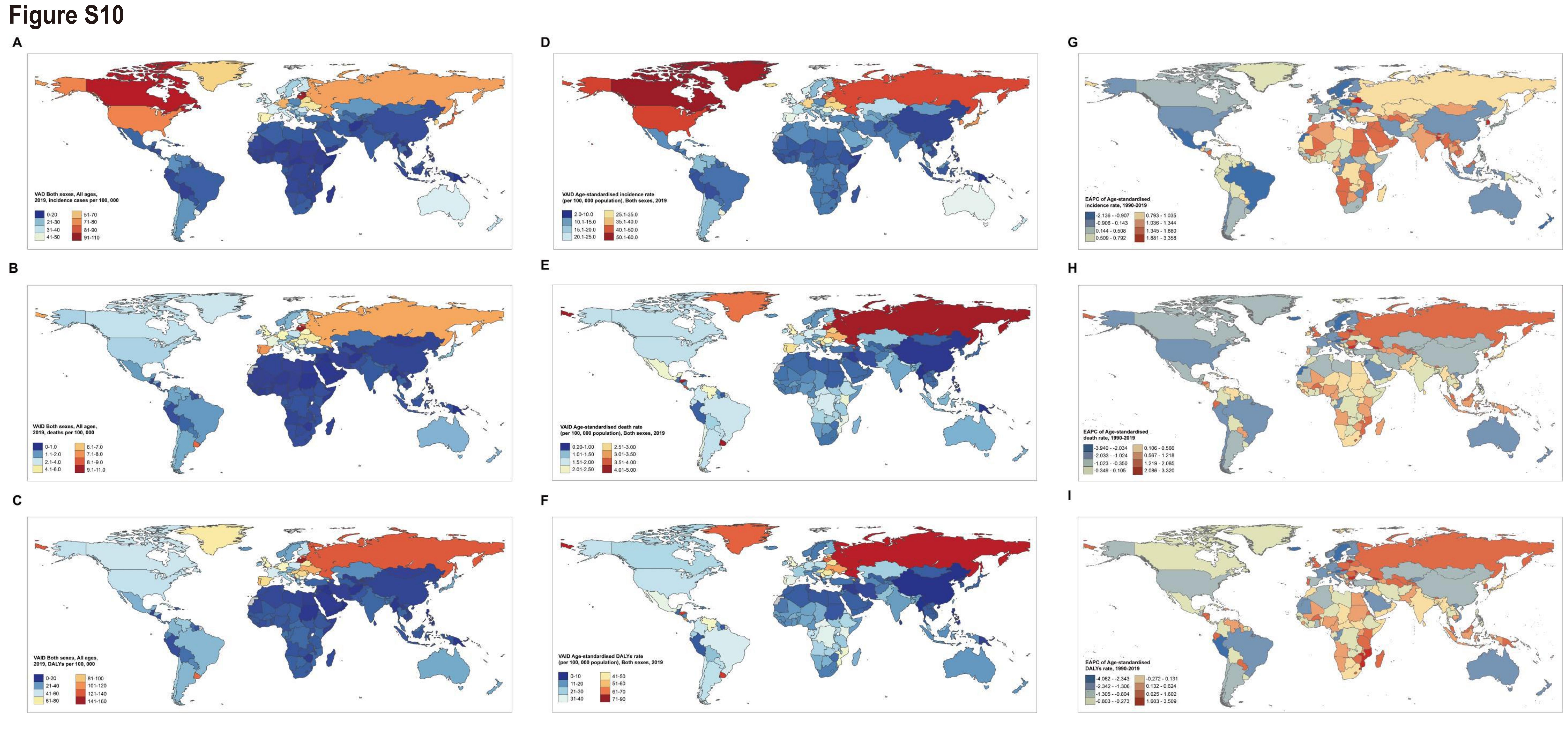

Supplement: Supplementary file 20 [file Image_10.JPEG]

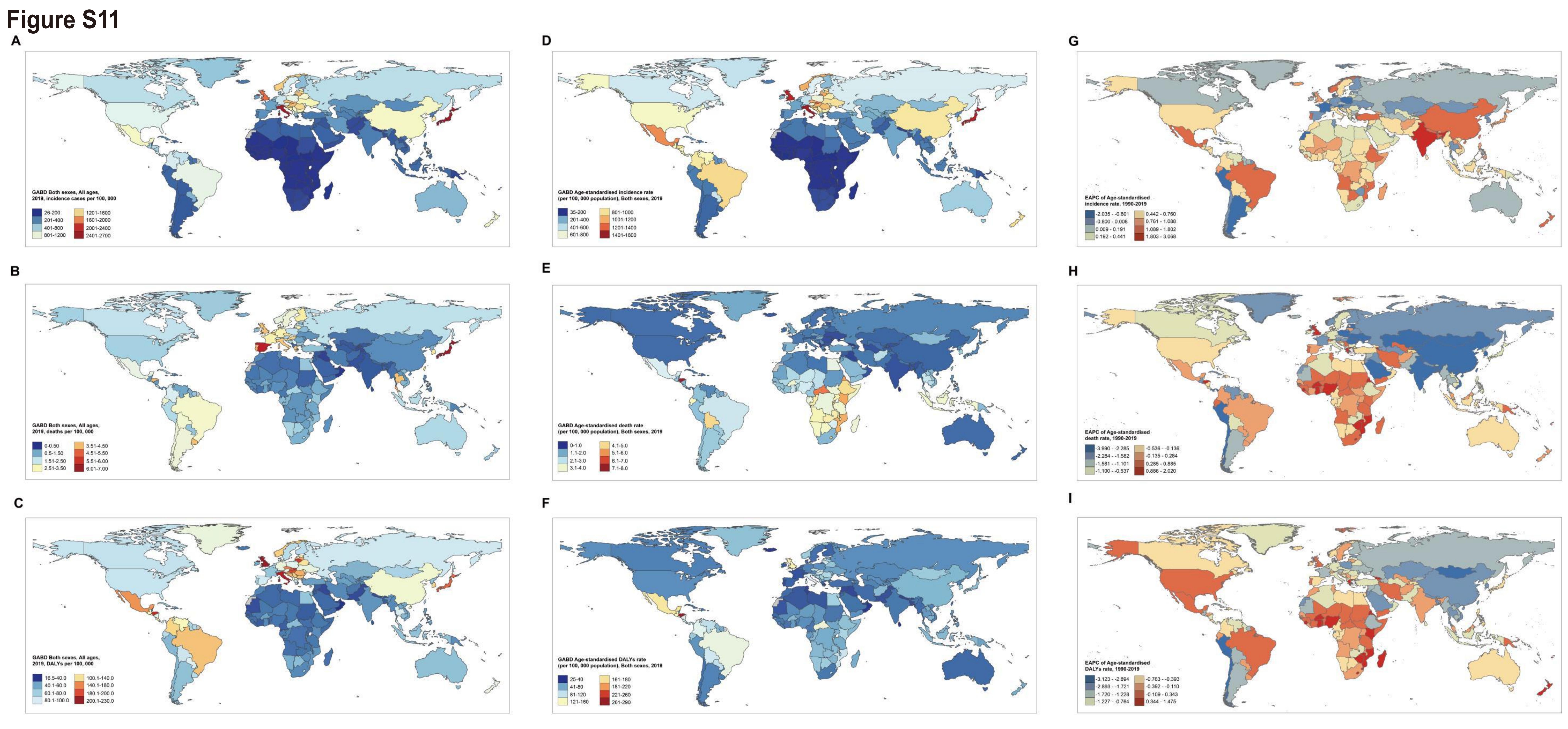

Supplement: Supplementary file 21 [file Image_11.JPEG]

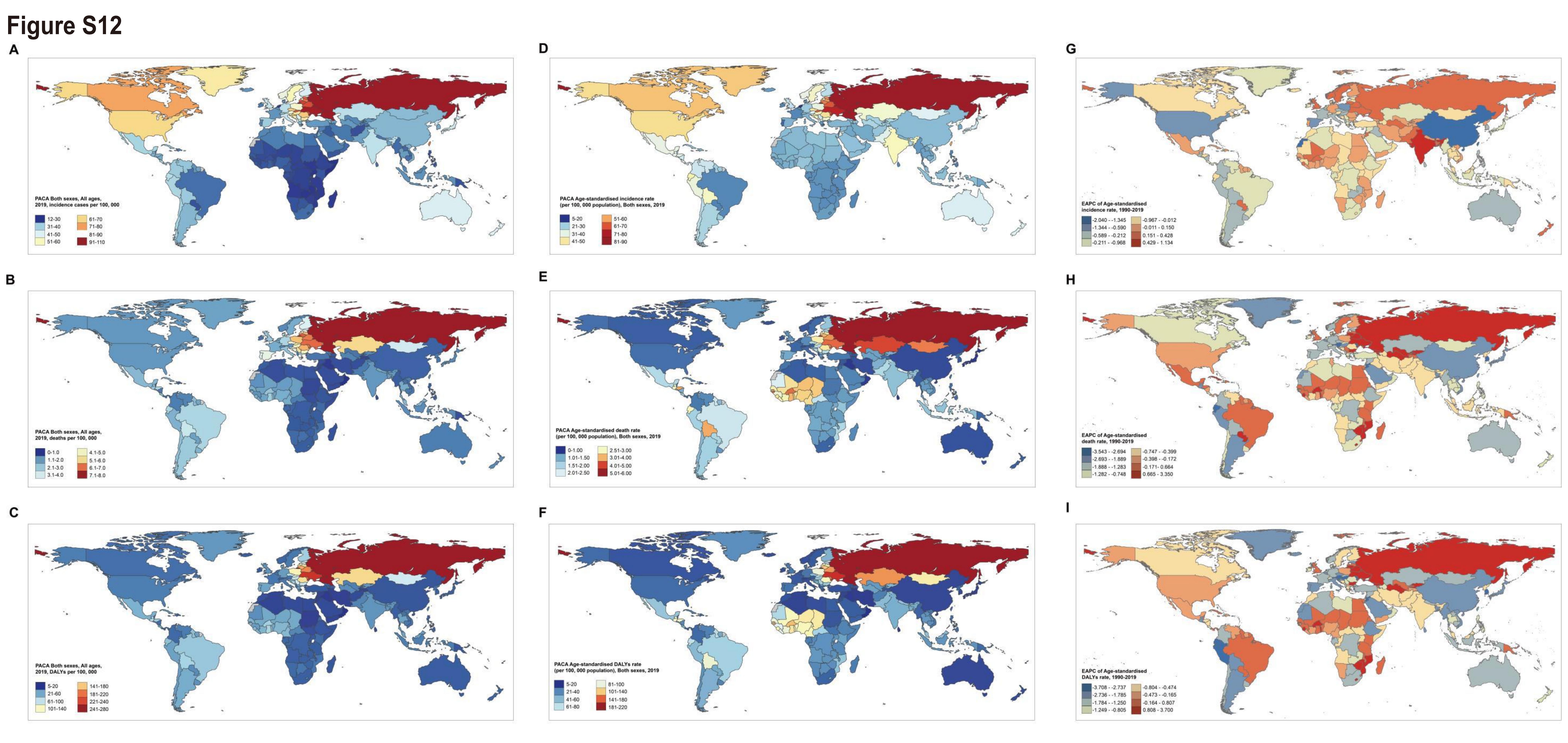

Supplement: Supplementary file 22 [file Image_12.JPEG]

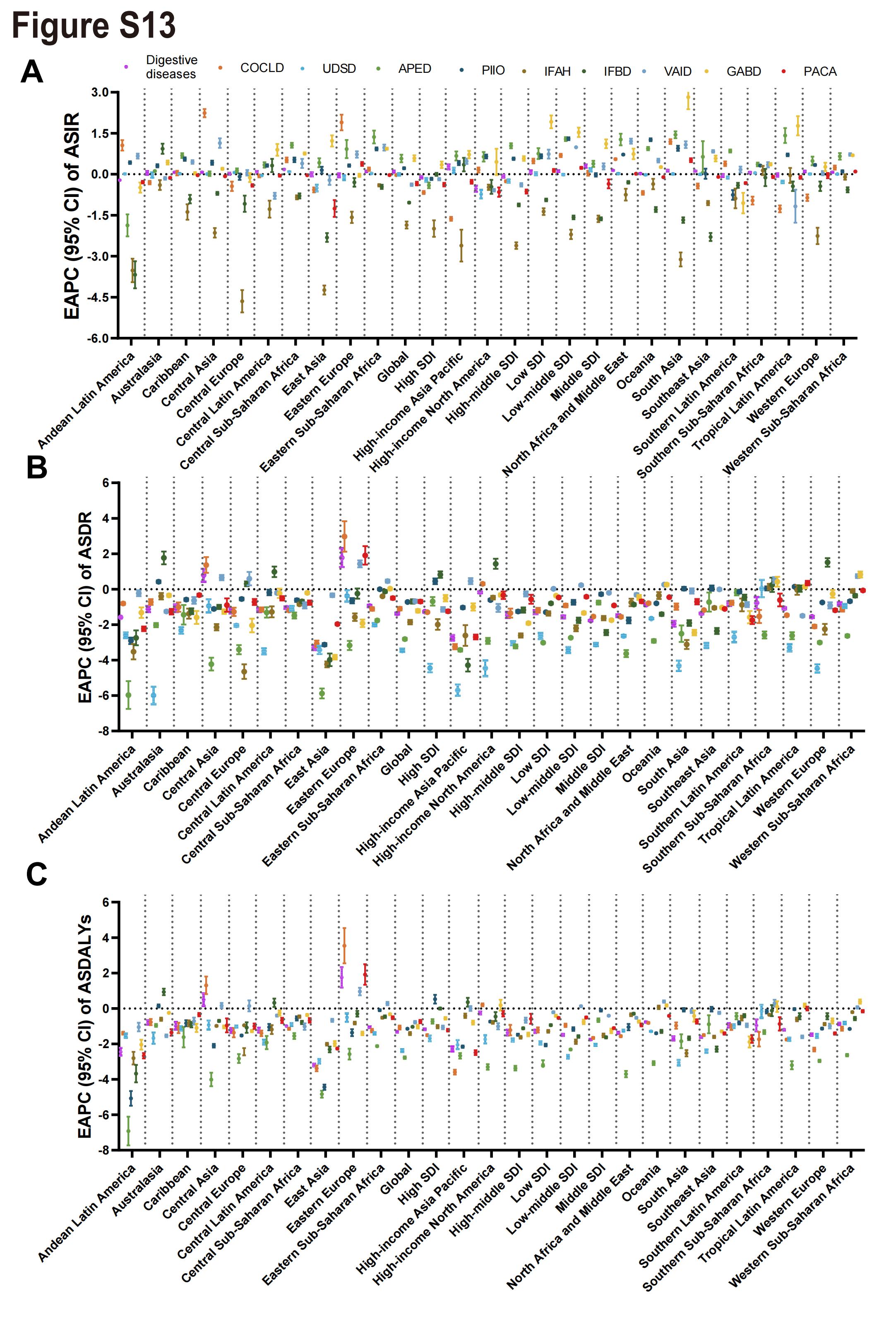

Supplement: Supplementary file 23 [file Image_13.JPEG]
